# Comparative Effectiveness and Safety of Oral Anticoagulants by Dementia Status in Older Patients With Atrial Fibrillation

**DOI:** 10.1001/jamanetworkopen.2023.4086

**Published:** 2023-03-28

**Authors:** Kueiyu Joshua Lin, Daniel E. Singer, Katsiaryna Bykov, Lily G. Bessette, Julianna M. Mastrorilli, Alexander Cervone, Dae Hyun Kim

**Affiliations:** 1 Division of Pharmacoepidemiology and Pharmacoeconomics, Department of Medicine, Brigham and Women’s Hospital and Harvard Medical School, Boston, Massachusetts; 2 Division of General Internal Medicine, Department of Medicine, Massachusetts General Hospital, Boston; 3 Hinda and Arthur Marcus Institute for Aging Research, Hebrew SeniorLife, Boston, Massachusetts; 4 Division of Gerontology, Department of Medicine, Beth Israel Deaconess Medical Center, Boston, Massachusetts

## Abstract

**Importance:**

The development of an optimal stroke prevention strategy, including the use of oral anticoagulant (OAC) therapy, is particularly important for patients with atrial fibrillation (AF) who are living with dementia, a condition that increases the risk of adverse outcomes. However, data on the role of dementia in the safety and effectiveness of OACs are limited.

**Objective:**

To assess the comparative safety and effectiveness of specific OACs by dementia status among older patients with AF.

**Design, Setting, and Participants:**

This retrospective comparative effectiveness study used 1:1 propensity score matching among 1 160 462 patients 65 years or older with AF. Data were obtained from the Optum Clinformatics Data Mart (January 1, 2013, to June 30, 2021), IBM MarketScan Research Database (January 1, 2013, to December 31, 2020), and Medicare claims databases maintained by the Centers for Medicare & Medicaid Services (inpatient, outpatient, and pharmacy; January 1, 2013, to December 31, 2017). Data analysis was performed from September 1, 2021, to May 24, 2022.

**Exposures:**

Apixaban, dabigatran, rivaroxaban, or warfarin.

**Main Outcomes and Measures:**

Composite end point of ischemic stroke or major bleeding events over the 6-month period after OAC initiation, pooled across databases using random-effects meta-analyses.

**Results:**

Among 1 160 462 patients with AF, the mean (SD) age was 77.4 (7.2) years; 50.2% were male, 80.5% were White, and 7.9% had dementia. Three comparative new-user cohorts were established: warfarin vs apixaban (501 990 patients; mean [SD] age, 78.1 [7.4] years; 50.2% female), dabigatran vs apixaban (126 718 patients; mean [SD] age, 76.5 [7.1] years; 52.0% male), and rivaroxaban vs apixaban (531 754 patients; mean [SD] age, 76.9 [7.2] years; 50.2% male). Among patients with dementia, compared with apixaban users, a higher rate of the composite end point was observed in warfarin users (95.7 events per 1000 person-years [PYs] vs 64.2 events per 1000 PYs; adjusted hazard ratio [aHR], 1.5; 95% CI, 1.3-1.7), dabigatran users (84.5 events per 1000 PYs vs 54.9 events per 1000 PYs; aHR, 1.5; 95% CI, 1.2-2.0), and rivaroxaban users (87.4 events per 1000 PYs vs 68.5 events per 1000 PYs; aHR, 1.3; 95% CI, 1.1-1.5). In all 3 comparisons, the magnitude of the benefits associated with apixaban was similar regardless of dementia diagnosis on the HR scale but differed substantially on the rate difference (RD) scale. The adjusted RD of the composite outcome per 1000 PYs for warfarin vs apixaban users was 29.8 (95% CI, 18.4-41.1) events in patients with dementia vs 16.0 (95% CI, 13.6-18.4) events in patients without dementia. The corresponding adjusted RD estimates of the composite outcome were 29.6 (95% CI, 11.6-47.6) events per 1000 PYs in patients with dementia vs 5.8 (95% CI, 1.1-10.4) events per 1000 PYs in patients without dementia for dabigatran vs apixaban users and 20.5 (95% CI, 9.9-31.1) events per 1000 PYs in patients with dementia vs 15.9 (95% CI, 11.4-20.3) events per 1000 PYs in patients without dementia for rivaroxaban vs apixaban users. The pattern was more distinct for major bleeding than for ischemic stroke.

**Conclusions and Relevance:**

In this comparative effectiveness study, apixaban was associated with lower rates of major bleeding and ischemic stroke compared with other OACs. The increased absolute risks associated with other OACs compared with apixaban were greater among patients with dementia than those without dementia, particularly for major bleeding. These findings support the use of apixaban for anticoagulation therapy in patients living with dementia who have AF.

## Key Points

**Question:**

What are the comparative effectiveness and safety of oral anticoagulants (OACs) by dementia status in older patients with atrial fibrillation (AF)?

**Findings:**

In this comparative effectiveness study of 1 160 462 patients with AF with and without dementia, apixaban was associated with net clinical benefits compared with other OACs and a greater absolute risk reduction in patients with vs without dementia. The rate difference of the composite outcome of ischemic stroke or major bleeding events per 1000 person-years over 6 months was 29.8 vs 16.0 for warfarin vs apixaban, 29.6 vs 5.8 for dabigatran vs apixaban, and 20.5 vs 15.9 for rivaroxaban vs apixaban in patients with vs without dementia.

**Meaning:**

These results support the use of apixaban for OAC therapy in persons living with dementia who have AF.

## Teaser

This comparative effectiveness study uses data from 3 US claims databases to assess the comparative safety and effectiveness of specific oral anticoagulant therapies by dementia status among older patients with atrial fibrillation.

## Introduction

Nonvalvular atrial fibrillation (AF) is highly prevalent in older adults^1^ and is associated with a 5-fold increased risk of embolic stroke.^2^ Anticoagulation can reduce this risk by approximately 70%, and clinical guidelines^3,4^ recommend that patients with AF who have an estimated annual stroke risk of 2.2% or greater (based on patient characteristics) receive anticoagulation therapy,^5,6^ which means the majority of older adults with AF should receive an oral anticoagulant (OAC).^7,8^ However, the prescribing decisions for OACs need to be individualized according to comorbidities and frailty because the risk-benefit profiles of OACs can vary substantially in different risk groups.^9^ The presence of dementia often complicates such a decision because persons living with dementia are at higher risk of falls, traumatic intracranial bleeding, medication errors, and low adherence to treatment regimens.^10,11^ Because dementia affects more than 6 million persons in the US^12^ and approximately 11% of older adults with AF,^13,14^ it is important to have an optimal stroke prevention strategy for persons living with dementia who have AF.

While the efficacy and safety of direct oral anticoagulants (DOACs) vs warfarin in patients with AF has been well established in randomized clinical trials (RCTs),^15,16,17,18^ persons living with dementia and older adults with frailty were substantially underrepresented in these trials. The small number of persons living with dementia who can consent and adhere to the study protocol of an RCT is unlikely to be representative of those living with dementia who are receiving routine care in terms of severity of cognitive and mobility impairment.^19,20^ In the absence of RCT evidence, analyses of routinely collected clinical data must be relied on to inform optimal choices for stroke prevention strategies among persons living with dementia who have AF. However, there are few RCT data on the role of dementia in the safety and effectiveness of OACs.^9^ We aimed to examine the comparative safety and effectiveness of specific OACs by assessing the risks of ischemic stroke and major bleeding events by dementia status among older patients with AF.

## Methods

### Study Population Based on OAC Exposure

We conducted a comparative effectiveness study using data from 3 large US claims databases: Optum Clinformatics Data Mart (January 1, 2013, to June 30, 2021), IBM MarketScan Research Database (January 1, 2013, to December 31, 2020), and Medicare fee-for-service Part A (inpatient claims), Part B (outpatient claims), and Part D (pharmacy claims; January 1, 2013, to December 31, 2017) databases maintained by the Centers for Medicare & Medicaid Services. Among adults 65 years or older, we established 3 comparative cohorts of new users of warfarin vs apixaban, dabigatran vs apixaban, and rivaroxaban vs apixaban who filled a prescription for the specific OAC without use of any OAC in the preceding 183 days from January 1, 2013, through June 30, 2021. Data analysis was performed from September 1, 2021, to May 24, 2022. This study was approved by the institutional review board of Brigham and Women’s Hospital in Boston, Massachusetts. A waiver of informed consent was granted because the study was a secondary analysis of routinely collected data that were unidentified. This study followed the Strengthening the Reporting of Observational Studies in Epidemiology (STROBE) reporting guideline for cohort studies.^21^

We used apixaban as the referent group because this medication has become the preferred OAC in older adults.^22,23,24^ The cohort entry (index) date was the OAC dispensing date, and the 365-day period before the index date was the baseline assessment period. We further applied the following inclusion criteria: (1) 1 or more diagnosis of AF in the baseline assessment period and (2) enrollment in medical and pharmacy coverage for at least 365 days. We excluded those with the following conditions in the baseline assessment period: (1) received hospice care, (2) had other indications for OAC (venous thromboembolism or joint replacement), (3) had major bleeding within the past 30 days, and (4) had valvular heart diseases or end-stage kidney or liver disease in the previous year (definitions are provided in eMethods in Supplement 1).

### Measurement of Baseline Covariates

We measured 79 covariates, including patient demographic characteristics, comorbidities, prescription drug use, and health care use, during the baseline assessment period. Because absence of code is assumed to indicate absence of condition, missing data (eg, missing a definitive diagnosis or procedure code) would have resulted in misclassification of the covariates. Therefore, we included only participants with baseline enrollment for at least 365 days before study entry to reduce this type of misclassification.^25^ Race and ethnicity were included as covariates because they could have been potential confounders of the analysis; a missing indicator was used for those missing race and ethnicity information. We computed the CHA_2_DS_2_-VASc (congestive heart failure, hypertension, age ≥75 years, diabetes, stroke or transient ischemic attack, vascular disease, age 65-74 years, and sex category) score^5,6^ for ischemic stroke risk and the modified HAS-BLED (hypertension, abnormal liver and/or kidney function, stroke history, bleeding history or predisposition, labile international normalized ratio, elderly, drug and alcohol use concomitantly) score for major bleeding risk.^6,26,27^

We used validated algorithms with positive predictive values of 78% to 92% to define dementia.^28^ Using data from the 365 days before cohort entry, we identified patients through *International Classification of Diseases, Ninth Revision* (*ICD-9*) and *International Classification of Diseases, Tenth Revision* (*ICD-10*) codes for dementia validated by Taylor et al,^29^ which has also been adopted as the Chronic Conditions Data Warehouse definition by the Centers for Medicare & Medicaid Services. In a sensitivity analysis, we further subdivided patients with a dementia diagnosis into those using dementia drugs (ie, donepezil, rivastigmine, galantamine, and memantine) vs those not using dementia drugs because persons living with dementia who are receiving these medications are more likely to have more clinically severe dementia.^30,31^

The burden of comorbidity was quantified using a combined comorbidity score based on 20 chronic medical conditions; this combined score has outperformed 2 widely used comorbidity scores, the Charlson Comorbidity Index and the Elixhauser Comorbidity Index, in estimating 1-year mortality.^32^ Frailty was measured using a claims-based frailty index (CFI) that was validated against clinical measures of frailty.^33,34,35,36^ The CFI has been validated against gait speed,^33^ grip strength,^33^ frailty phenotype,^35^ the deficit-accumulation frailty index,^35^ and severe disability.^35^ Using accepted cutoffs,^37,38,39^ we defined nonfrailty as a CFI score of lower than 0.15, prefrailty as a CFI score of 0.15 to 0.24, and frailty as a CFI score of 0.25 or higher.

### Outcomes and Follow-up

The primary outcome was a composite end point of ischemic stroke or major bleeding. Secondary outcomes included individual components of the primary outcome (ie, ischemic stroke and components of the major bleeding events, including gastrointestinal [GI] and other extracranial bleeding and intracranial hemorrhage [ICH]) and all-cause mortality. Because death ascertainment in the Optum and MarketScan databases was inadequate (data are only based on discharge status), the death outcome was only assessed in the Medicare population (which also contains death information from Social Security files). Clinical events were defined using validated algorithms with high performance (positive predictive values of 85%-90% for ischemic stroke^40,41^ and 86%-96%^42^ for major bleeding outcomes) (definitions are available in eMethods in Supplement 1). Follow-up began 1 day after the index date and continued for a maximum of 183 days or the earliest of the following events: occurrence of the study outcome, disenrollment from insurance, or end of data availability. We used intention-to-treat analysis in the primary analysis and as-treated analysis in the sensitivity analysis. In the sensitivity analysis, study participants were also censored at the date on which they discontinued use of the index drug or switched to another OAC. To assess the robustness of findings for discontinuation of the index drug, 3 definitions of discontinuation were used based on gaps in OAC dispensing of more than 7 days, more than 14 days, and more than 30 days.

### Statistical Analysis

We compared clinical characteristics between participants using apixaban vs participants using each of the other OACs (dabigatran, rivaroxaban, or warfarin). We used 1:1 propensity score (PS) matching to adjust for confounding. We estimated the PS using a logistic regression analysis that modeled the probability of initiating apixaban vs another AOC as a function of the 79 covariates, including calendar time by quarters (PS variables are shown in eTables 1-3 in Supplement 1). The PS model development and PS matching were conducted within each database and dementia subgroup (dementia or no dementia). In the matched cohort, we estimated hazard ratios (HRs) and 95% CIs using Cox proportional hazards models. We checked the proportionality assumption by inspecting the Kaplan-Meier curves; this assumption was satisfied in our analyses. We estimated rate differences (RDs) using generalized linear models, assuming Poisson distributions. We used random effects meta-analysis to pool database-specific estimates. We also conducted a meta-analysis of the dementia subgroup–specific HR and RD estimates and examined heterogeneity across subgroups.^43^

We conducted several sensitivity analyses. First, to account for unmeasured confounding, we also used the high-dimensional propensity score (HDPS),^44^ which semiautomatically identifies, prioritizes, and adjusts for thousands of codes empirically associated with the exposure and the outcome.^44,45,46,47,48^ Second, we excluded those with ischemic stroke in the 60 days before OAC initiation (to avoid misclassifying diagnoses of previous stroke as incident events during follow-up). Third, we excluded those admitted to a short-term skilled nursing facility (SNF) at baseline to avoid misclassification of OAC exposure given that Medicare Part D does not cover medications during short-term SNF stays. Fourth, to account for variations in clinical practice, we adjusted the PS model for the 50 US states. Fifth, we excluded those receiving reduced doses of a DOAC (75.0 mg of dabigatran, 15.0 mg of rivaroxaban, or 2.5 mg of apixaban) because the reasons underlying the reduced dosing may be underrecorded in the claims data. Sixth, to assess the potential impact of competing risks due to death, we assessed a composite outcome of ischemic stroke, major bleeding, and death. Analyses were conducted through the Aetion Evidence Generation Platform (Aetion) using R software, version 3.4.2 (R Foundation for Statistical Computing).^49,50,51^ The significance threshold was 2-tailed *P* = .05.

## Results

### Characteristics of Study Populations

Among 1 160 462 patients with AF, the mean (SD) age was 77.4 (7.2) years; 49.8% were female, 50.2% were male, 4.3% were Black, 80.5% were White, and 61% were of other races and/or ethnicities (including Asian, Hispanic, North American Native, other, unknown, and missing). In total, 7.9% of patients had dementia and 92.1% did not. We established 3 new-user cohorts comprising patients using warfarin vs apixaban (501 990 total patients from the 3 databases; mean [SD] age, 78.1 [7.4] years; 50.2% female) (eFigure 1 in Supplement 1), dabigatran vs apixaban (126 718 total patients from the 3 databases; mean [SD] age, 76.5 [7.1] years; 48.0% female) (eFigure 2 in Supplement 1), and rivaroxaban vs apixaban (531 754 total patients from the 3 databases; mean [SD] age, 76.9 [7.2] years; 49.8% female) (eFigure 3 in Supplement 1). The pooled estimates of patient characteristics based on the 3 databases combined (after PS matching) are shown in the Table (database-specific estimates are shown in eTables 4-15 in Supplement 1). Before PS matching, compared with apixaban users, dabigatran and rivaroxaban users were younger (dabigatran vs apixaban group: mean [SD] age, 76.4 [7.1] years vs 78.1 [7.4] years; rivaroxaban vs apixaban group: mean [SD] age, 76.7 [7.1] years vs 77.8. [7.4] years). Dabigatran and rivaroxaban users were also healthier than apixaban users, as observed in lower CHA_2_DS_2_-VASc scores (mean [SD], 4.40 [1.69] among dabigatran users and 4.41 [1.70] among rivaroxaban users vs 4.63 [1.70] among apixaban users), lower comorbidity scores (mean [SD], 2.85 [2.58] among dabigatran users and 3.02 [2.66] among rivaroxaban users vs 3.50 [2.90] among apixaban users), and lower prevalence of comorbidities (eg, hypertension: 84.6% of dabigatran users and 84.9% of rivaroxaban users vs 87.1% of apixaban users) (eTables 8-15 in Supplement 1). In contrast, warfarin vs apixaban users had similar ages (mean [SD], 78.2 [7.3] years vs 77.8 [7.4] years), CHA_2_DS_2_-VASc scores (mean [SD], 4.80 [1.70] vs 4.60 [1.70]), and comorbidity scores (mean [SD], 3.50 [2.80] vs 3.50 [2.90]) (eTables 4-7 in Supplement 1). After PS matching, all characteristics were adequately balanced between exposure groups in all 3 cohorts (Table) and within dementia status (eTables 16-27 in Supplement 1). Compared with patients without dementia, those living with dementia were older, more likely to be female, and more likely to have higher CFI scores, higher comorbidity scores, higher CHA_2_DS_2_-VASc scores, higher HAS-BLED scores, and greater health care use (eTables 16-27 in Supplement 1).

**Table.** Participant Characteristics^a^

Characteristic	Participants, No. (%)					
	Warfarin cohort		Dabigatran cohort		Rivaroxaban cohort	
	Apixaban (n = 250 995)	Warfarin (n = 250 995)	Apixaban (n = 63 359)	Dabigatran (n = 63 359)	Apixaban (n = 265 877)	Rivaroxaban (n = 265 877)
Age, mean (SD), y	78.1 (7.4)	78.1 (7.3)	76.5 (7.1)	76.6 (7.1)	76.8 (7.2)	76.9 (7.2)
Sex						
Female	125 863 (50.1)	126 130 (50.3)	30 448 (48.1)	30 261 (47.8)	132 320 (49.8)	132 423 (49.8)
Male	125 132 (49.9)	124 865 (49.7)	32 911 (51.9)	33 098 (52.2)	133 557 (50.2)	133 454 (50.2)
Race and ethnicity						
Black	10 827 (4.7)	10 818 (4.7)	2487 (4.4)	2428 (4.3)	11 828 (4.9)	11 853 (4.9)
White	206 224 (89.5)	206 187 (89.4)	50 350 (88.8)	50 444 (89.0)	210 296 (87.5)	210 296 (87.5)
Other^b^	13 487 (5.9)	13 533 (5.9)	3834 (6.8)	3799 (6.7)	18 084 (7.5)	18 059 (7.5)
Dual insurance status^c^	11 430 (5.9)	11 457 (5.9)	2633 (5.3)	2583 (5.2)	10 882 (5.6)	10 704 (5.6)
CHA_2_DS_2_-VASc score, mean (SD)	4.71 (1.70)	4.71 (1.66)	4.42 (1.69)	4.41 (1.70)	4.43 (1.69)	4.42 (1.70)
HAS-BLED score, mean (SD)	2.30 (0.70)	2.30 (0.74)	2.22 (0.72)	2.22 (0.70)	2.23 (0.71)	2.23 (0.70)
CCI score, mean (SD)	3.48 (2.90)	3.48 (2.83)	2.90 (2.60)	2.88 (2.60)	3.11 (2.71)	3.08 (2.70)
CFI score, mean (SD)	0.21 (0.10)	0.21 (0.07)	0.20 (0.07)	0.20 (0.10)	0.20 (0.07)	0.20 (0.10)
Cardiovascular condition						
Acute myocardial infarction	17 199 (6.9)	17 277 (6.9)	2898 (4.6)	2866 (4.5)	14 572 (5.5)	14 427 (5.4)
Cardioablation	1286 (0.5)	1260 (0.5)	411 (0.6)	403 (0.6)	1708 (0.6)	1699 (0.6)
Cardioversion	10 679 (4.3)	10 414 (4.1)	3998 (6.3)	4021 (6.3)	17 755 (6.7)	17 691 (6.7)
Cerebrovascular disease	70 312 (28.0)	70 459 (28.1)	16 593 (26.2)	16 594 (26.2)	65 070 (24.5)	64 393 (24.2)
Congestive heart failure						
Inpatient	51 717 (20.6)	51 651 (20.6)	9880 (15.6)	9577 (15.1)	45 065 (16.9)	44 516 (16.7)
Outpatient	86 644 (34.5)	86 440 (34.4)	18 812 (29.7)	18 789 (29.7)	77 540 (29.2)	77 249 (29.1)
Coronary revascularization	11 564 (4.6)	11 485 (4.6)	1622 (2.6)	1589 (2.5)	7461 (2.8)	7433 (2.8)
Hypertension	215 906 (86.0)	215 775 (86.0)	53 643 (84.7)	53 642 (84.7)	226 620 (85.2)	226 481 (85.2)
Ischemic heart disease	117 173 (46.7)	116 858 (46.6)	27 410 (43.3)	27 616 (43.6)	113 388 (42.6)	113 118 (42.5)
PVD or PVD surgical procedure	37 345 (14.9)	37 351 (14.9)	8182 (12.9)	8111 (12.8)	35 389 (13.3)	35 243 (13.3)
Stroke						
Inpatient	22 655 (9.0)	22 863 (9.1)	4879 (7.7)	4803 (7.6)	18 749 (7.1)	18 282 (6.9)
Outpatient	31 251 (12.5)	31 324 (12.5)	6890 (10.9)	6915 (10.9)	29 610 (11.1)	29 254 (11.0)
Syncope	24 758 (9.9)	24 920 (9.9)	6197 (9.8)	6290 (9.9)	27 617 (10.4)	27 595 (10.4)
Noncardiovascular condition						
Acute kidney failure	36 212 (14.4)	36 240 (14.4)	5743 (9.1)	5665 (8.9)	28 822 (10.8)	28 373 (10.7)
Alcohol misuse or dependence	4783 (1.9)	4799 (1.9)	1179 (1.9)	1196 (1.9)	5635 (2.1)	5642 (2.1)
Anemia	72 201 (28.8)	72 184 (28.8)	15 198 (24.0)	15 251 (24.1)	65 428 (24.6)	64 976 (24.4)
CKD stage 3, 4, or unspecified	47 757 (19.0)	47 525 (18.9)	7757 (12.2)	7676 (12.1)	37 598 (14.1)	37 044 (13.9)
COPD	57 603 (22.9)	57 315 (22.8)	12 994 (20.5)	12 753 (20.1)	56 712 (21.3)	56 494 (21.2)
Dementia	21 079 (8.4)	21 190 (8.4)	4338 (6.8)	4239 (6.7)	20 229 (7.6)	20 073 (7.5)
Diabetes	94 441 (37.6)	94 494 (37.6)	22 987 (36.3)	23 017 (36.3)	93 060 (35.0)	93 275 (35.1)
Endoscopy	5630 (2.2)	5754 (2.3)	1057 (1.7)	1069 (1.7)	4505 (1.7)	4486 (1.7)
Falls	15 256 (6.1)	15 324 (6.1)	2830 (4.5)	2836 (4.5)	14 772 (5.6)	14 569 (5.5)
Fractures	22 711 (9.0)	22 817 (9.1)	4946 (7.8)	5016 (7.9)	22 651 (8.5)	22 484 (8.5)
GI bleeding						
Inpatient	14 792 (5.9)	14 750 (5.9)	2923 (4.6)	2848 (4.5)	11 940 (4.5)	11 913 (4.5)
Outpatient	33 501 (13.3)	33 629 (13.4)	8001 (12.6)	8082 (12.8)	33 578 (12.6)	33 615 (12.6)
Liver disease	15 573 (6.2)	15 508 (6.2)	3738 (5.9)	3644 (5.8)	16 460 (6.2)	16 377 (6.2)
Cancer	45 657 (18.2)	45 764 (18.2)	11 225 (17.7)	11 228 (17.7)	47 014 (17.7)	46 769 (17.6)
Obesity	48 879 (19.5)	48 953 (19.5)	12 222 (19.3)	12 129 (19.1)	53 387 (20.1)	53 565 (20.1)
Peptic ulcer	5622 (2.2)	5637 (2.2)	1077 (1.7)	1087 (1.7)	5312 (2.0)	5214 (2.0)
Ever smoked	77 467 (30.9)	77 226 (30.8)	17 619 (27.8)	17 632 (27.8)	81537 (30.7)	81 186 (30.5)
Cardiovascular medication						
ACE inhibitor	72 535 (28.9)	72 450 (28.9)	16 933 (26.7)	16 840 (26.6)	71 166 (26.8)	71 131 (26.8)
Angiotensin 2 receptor blocker	13 661 (5.4)	13 623 (5.4)	4085 (6.4)	4141 (6.5)	15 985 (6.0)	15 891 (6.0)
Antiarrhythmic agent	47 005 (18.7)	46 726 (18.6)	13 731 (21.7)	13 684 (21.6)	51 923 (19.5)	51 877 (19.5)
Injectable anticoagulant	2673 (1.1)	2733 (1.1)	465 (0.7)	467 (0.7)	1836 (0.7)	1857 (0.7)
Antiplatelet agent	37 072 (14.8)	36 842 (14.7)	8726 (13.8)	8863 (14.0)	39 437 (14.8)	39 282 (14.8)
β-blocker	171 519 (68.3)	171 238 (68.2)	41 245 (65.1)	41 157 (65.0)	175 911 (66.2)	175 740 (66.1)
Calcium channel blocker	4418 (1.8)	4425 (1.8)	1073 (1.7)	1049 (1.7)	4273 (1.6)	4294 (1.6)
Diuretic	143 919 (57.3)	143 827 (57.3)	33 426 (52.8)	33 405 (52.7)	138 452 (52.1)	138 451 (52.1)
Fibrate	11 428 (4.6)	11 430 (4.6)	2930 (4.6)	2935 (4.6)	11 527 (4.3)	11 445 (4.3)
Nitrate	33 127 (13.2)	32 813 (13.1)	7245 (11.4)	7375 (11.6)	29 109 (10.9)	28 907 (10.9)
Statin	164 076 (65.4)	164 017 (65.3)	40 400 (63.8)	40 472 (63.9)	168 276 (63.3)	168 050 (63.2)
Other medication						
Anticonvulsant	41 224 (16.4)	41 414 (16.5)	9738 (15.4)	9800 (15.5)	42 094 (15.8)	42 098 (15.8)
Antidepressant						
SSRI or SNRI	50 898 (20.3)	50 832 (20.3)	12 783 (20.2)	12 915 (20.4)	53 510 (20.1)	53 296 (20.0)
Tricyclic	6709 (2.7)	6750 (2.7)	1627 (2.6)	1644 (2.6)	6952 (2.6)	6968 (2.6)
Other	21 171 (8.4)	21 200 (8.4)	4951 (7.8)	4878 (7.7)	22 242 (8.4)	22 214 (8.4)
Antipsychotic agent	7120 (2.8)	7289 (2.9)	1791 (2.8)	1783 (2.8)	8012 (3.0)	7930 (3.0)
Anxiolytic (except benzodiazepine)	2819 (1.1)	2843 (1.1)	699 (1.1)	685 (1.1)	3295 (1.2)	3400 (1.3)
Anxiolytic (benzodiazepine)	43 070 (17.2)	42 997 (17.1)	11 225 (17.7)	11 345 (17.9)	47 560 (17.9)	47 574 (17.9)
Bronchodilator	47 843 (19.1)	47 725 (19.0)	11 914 (18.8)	11 917 (18.8)	51 917 (19.5)	51 838 (19.5)
Corticosteroid						
Inhaled	52 557 (20.9)	52 626 (21.0)	13 923 (22.0)	14 178 (22.4)	59 412 (22.3)	59 287 (22.3)
Oral	77 799 (31.0)	77 813 (31.0)	19 970 (31.5)	19 909 (31.4)	85 193 (32.0)	85 226 (32.1)
Dementia medication	12 672 (5.0)	12 798 (5.1)	3152 (5.0)	3086 (4.9)	13 218 (5.0)	13 082 (4.9)
Diabetes medication						
Insulin	20 364 (8.1)	20 365 (8.1)	4616 (7.3)	4569 (7.2)	18 254 (6.9)	18 217 (6.9)
Metformin	40 724 (16.2)	40 688 (16.2)	10 687 (16.9)	10 686 (16.9)	43 459 (16.3)	43 851 (16.5)
Other	13 439 (5.4)	13 486 (5.4)	3576 (5.6)	3481 (5.5)	14 335 (5.4)	14 327 (5.4)
Sulfonylurea	25 654 (10.2)	25 591 (10.2)	6071 (9.6)	6049 (9.5)	23 584 (8.9)	23 533 (8.9)
Estrogen	6624 (2.6)	6730 (2.7)	2086 (3.3)	2036 (3.2)	8580 (3.2)	8536 (3.2)
GI						
Histamine type 2 receptor blocker	19 015 (7.6)	19 093 (7.6)	4499 (7.1)	4543 (7.2)	19 827 (7.5)	19 636 (7.4)
Proton pump inhibitor	80 061 (31.9)	79 915 (31.8)	19 592 (30.9)	19 832 (31.3)	83 026 (31.2)	82 760 (31.1)
Sucralfate	4669 (1.9)	4588 (1.8)	1203 (1.9)	1229 (1.9)	5027 (1.9)	4954 (1.9)
Hypnotic	19 257 (7.7)	19 167 (7.6)	5563 (8.8)	5552 (8.8)	22 039 (8.3)	22 182 (8.3)
NSAID	34 955 (13.9)	34 885 (13.9)	10 330 (16.3)	10 263 (16.2)	45 815 (17.2)	45 816 (17.2)
Opioid	96 939 (38.6)	96 727 (38.5)	24 184 (38.2)	24 135 (38.1)	99 554 (37.4)	99 953 (37.6)
Parkinsonism medication	9566 (3.8)	9687 (3.9)	2443 (3.9)	2444 (3.9)	9900 (3.7)	9921 (3.7)
Thyroid hormone replacement	56 383 (22.5)	56 525 (22.5)	13 489 (21.3)	13 353 (21.1)	56 783 (21.4)	56 666 (21.3)
Health care use						
ED visit	104 777 (41.7)	105 108 (41.9)	24 831 (39.2)	24 792 (39.1)	113 964 (42.9)	113 434 (42.7)
Home health services (in days)	7119 (3.7)	7239 (3.7)	2738 (5.5)	2720 (5.5)	6756 (3.5)	6829 (3.5)
Home oxygen use	8294 (3.3)	8362 (3.3)	1708 (2.7)	1653 (2.6)	8029 (3.0)	7947 (3.0)
Hospitalization	123 433 (49.2)	123 123 (49.1)	27 250 (43.0)	26 701 (42.1)	123 353 (46.4)	122 468 (46.1)
Geographic region						
Northeast	50 722 (20.2)	51 118 (20.4)	13 778 (21.7)	13 909 (22.0)	50 882 (19.1)	50 711 (19.1)
Midwest	66 024 (26.3)	65 599 (26.1)	13 688 (21.6)	13 398 (21.1)	60 070 (22.6)	60 053 (22.6)
South	87 165 (34.7)	87 114 (34.7)	24 577 (38.8)	24 779 (39.1)	103 420 (38.9)	103 250 (38.8)
West	46 822 (18.7)	46 892 (18.7)	11 236 (17.7)	11 190 (17.7)	51 227 (19.3)	51 567 (19.4)
Other	262 (0.1)	272 (0.1)	80 (0.1)	83 (0.1)	278 (0.1)	296 (0.1)

Abbreviations: ACE, angiotensin-converting enzyme; CCI, combined comorbidity index; CFI, claims-based frailty index; CHA_2_DS_2_-VASc, congestive heart failure, hypertension, age ≥75 years, diabetes, stroke or transient ischemic attack, vascular disease, age 65-74 years, and sex category; CKD, chronic kidney disease; COPD, chronic obstructive pulmonary disease; ED, emergency department; GI, gastrointestinal; HAS-BLED, hypertension, abnormal liver and/or kidney function, stroke history, bleeding history or predisposition, labile international normalized ratio, elderly, drug and alcohol use concomitantly; NSAID, nonsteroidal anti-inflammatory drug; PVD, peripheral vascular disease; SNRI, serotonin-norepinephrine reuptake inhibitor; SSRI, selective serotonin reuptake inhibitor.

^a^ Among a propensity score–matched population with atrial fibrillation who received treatment with oral anticoagulants, pooled across Medicare, Optum Clinformatics Data Mart, and IBM MarketScan Research Database populations.

^b^ Other races and ethnicities include Asian, Hispanic, North American Native, other, unknown, and missing categories in the Medicare database; Asian, Hispanic, unknown, and missing categories in the Optum Clinformatics Data Mart; and unavailable category in the IBM MarketScan Research Database.

^c^ With both Medicare and Medicaid enrollment eligibility.

### Clinical Outcomes Associated With Warfarin vs Apixaban by Dementia Status

**Figure 1.**
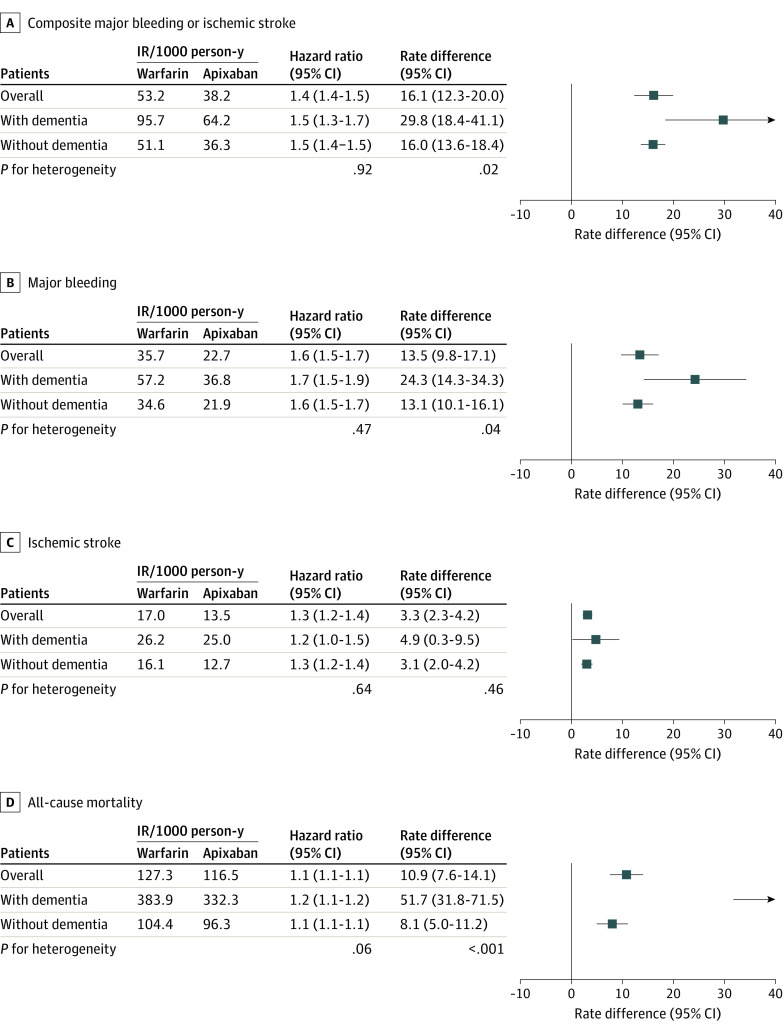
Association of Warfarin vs Apixaban With Clinical Outcomes Among a propensity score–matched US Medicare population with atrial fibrillation pooled across Medicare, Optum Clinformatics Data Mart, and IBM MarketScan Research Database populations. The estimates are pooled results across the 3 databases and were calculated using a random effects meta-analysis of the weighted mean of database-specific estimates. Mortality results were based on the Medicare population due to incomplete death ascertainment in the other 2 databases. IR indicates incidence rate.

Over a mean (SD) follow-up of 165.8 (43.0) days, the pooled rate of the composite end point per 1000 person-years (PYs) from the 3 databases combined was 53.2 events for warfarin users and 38.2 events for apixaban users (adjusted HR [aHR], 1.4 [95% CI, 1.4-1.5]; RD per 1000 PYs, 16.1 [95% CI, 12.3-20.0] events). Compared with apixaban users, warfarin users had higher rates of major bleeding (aHR, 1.6 [95% CI, 1.5-1.7]; RD per 1000 PYs, 13.5 [95% CI, 9.8-17.1] events), ischemic stroke (aHR, 1.3 [95% CI, 1.2-1.4]; RD per 1000 PYs, 3.3 [95% CI, 2.3-4.2] events), and death (aHR, 1.1 [95% CI, 1.1-1.1]; RD per 1000 PYs, 10.9 [95% CI, 7.6-14.1] events). The magnitude of the benefit associated with apixaban was consistent across dementia status on the HR scale (1.5 [95% CI, 1.3-1.7] for those with dementia vs 1.5 [95% CI, 1.4-1.5] for those without dementia; *P* = .92 for heterogeneity) but greater for those with vs without dementia on the RD scale (RD per 1000 PYs, 29.8 [95% CI, 18.4-41.1] events vs 16.0 [95% CI, 13.6-18.4] events; *P* = .02 for heterogeneity). The treatment effect heterogeneity in RD by dementia groups was evident for death and major bleeding, including both intracranial and extracranial bleeding, but not for ischemic stroke (pooled results are shown in Figure 1 and eTable 28 in Supplement 1, and database-specific estimates are shown in eTables 29-31 in Supplement 1).

### Clinical Outcomes Associated With Dabigatran vs Apixaban by Dementia Status

**Figure 2.**
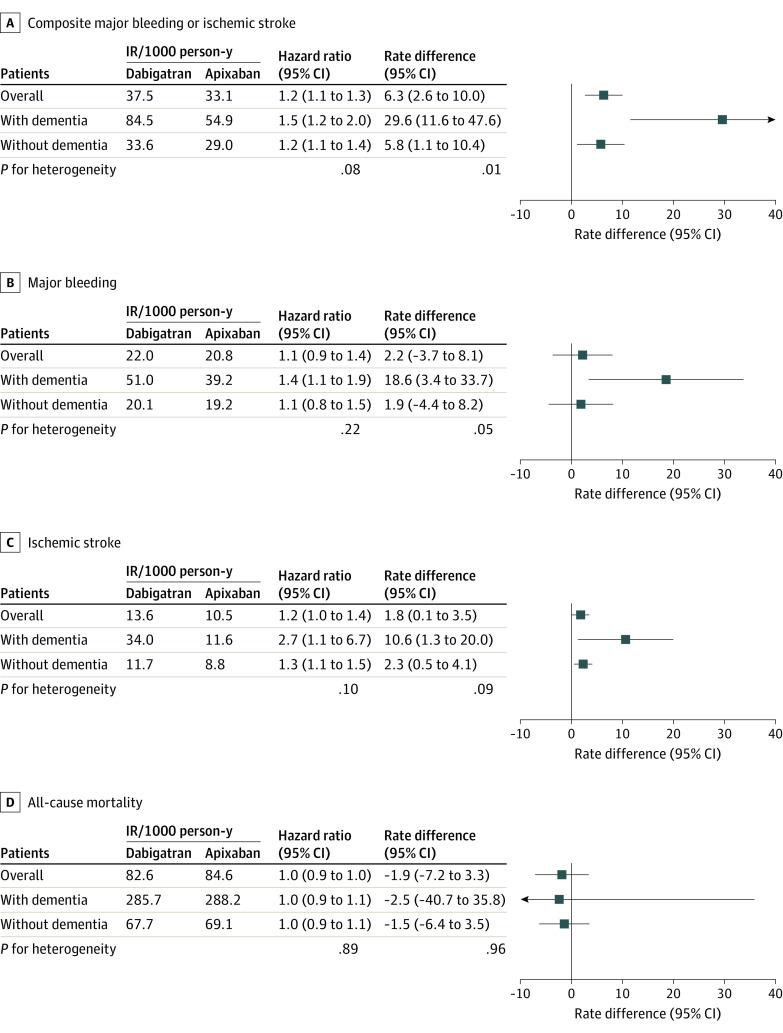
Association of Dabigatran vs Apixaban With Clinical Outcomes Among a propensity score–matched US Medicare population with atrial fibrillation pooled across Medicare, Optum Clinformatics Data Mart, and IBM MarketScan Research Database populations. The estimates are pooled results across the 3 databases and were calculated using a random effects meta-analysis of the weighted mean of database-specific estimates. Mortality results were based on the Medicare population due to incomplete death ascertainment in the other 2 databases. IR indicates incidence rate.

Over a mean (SD) follow-up of 169.5 (38.5) days, the pooled rate of the composite end point per 1000 PYs was 37.5 events for dabigatran users and 33.1 events for apixaban users (aHR, 1.2 [95% CI, 1.1-1.3]; RD per 1000 PYs, 6.3 [95% CI, 2.6-10.0] events). Compared with apixaban users, dabigatran users had higher rates of ischemic stroke (aHR, 1.2 [95% CI, 1.0-1.4]; RD per 1000 PYs, 1.8 [95% CI, 0.1-3.5] events). The magnitude of the benefit associated with apixaban was consistent across dementia subgroups on the HR scale (1.5 [95% CI, 1.2-2.0] for those with dementia vs 1.2 [95% CI, 1.1-1.4] for those without dementia; *P* = .08 for heterogeneity) but greater on the RD scale for those with vs without dementia (29.6 [95% CI, 11.6-47.6] events per 1000 PYs vs 5.8 [95% CI, 1.1-10.4] events per 1000 PYs; *P* = .001 for heterogeneity). The hemorrhagic risk reduction associated with apixaban was particularly distinct for GI bleeding but not evident for ICH, a pattern consistent across dementia subgroups (pooled results are shown in Figure 2 and eTable 32 in Supplement 1, and database-specific estimates are shown in eTables 33-35 in Supplement 1).

### Clinical Outcomes Associated With Rivaroxaban vs Apixaban by Dementia Status

**Figure 3.**
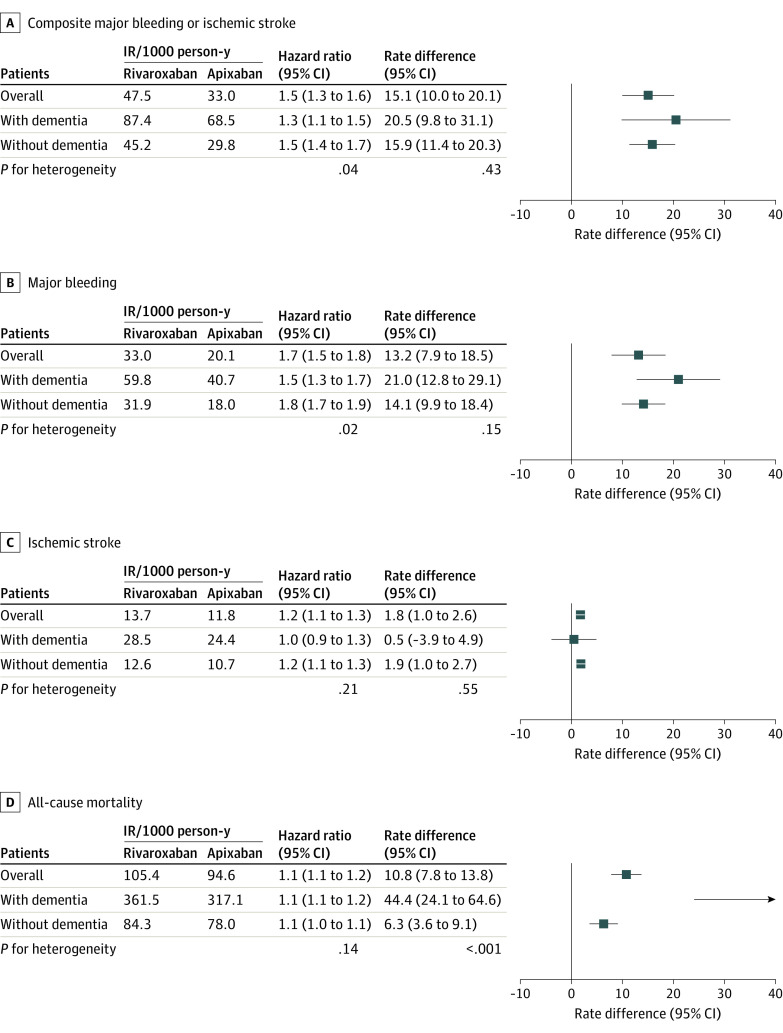
Association of Rivaroxaban vs Apixaban with Clinical Outcomes Among a propensity score–matched US Medicare population with atrial fibrillation pooled across Medicare, Optum Clinformatics Data Mart, and IBM MarketScan Research Database populations. The estimates are pooled results across the 3 databases and were calculated using a random effects meta-analysis of the weighted mean of database-specific estimates. Mortality results were based on the Medicare population due to incomplete death ascertainment in the other 2 databases. IR indicates incidence rate.

Over a mean (SD) follow-up of 164.0 (44.7) days, the rate of the composite end point per 1000 PYs was 47.5 events for rivaroxaban users and 33.0 events for apixaban users (aHR, 1.5 [95% CI, 1.3-1.6]; RD per 1000 PYs, 15.1 [95% CI, 10.0-20.1] events). Compared with apixaban users, rivaroxaban users had higher rates of major bleeding (aHR, 1.7 [95% CI, 1.5-1.8]; RD per 1000 PYs, 13.2 [95% CI, 7.9-18.5] events), ischemic stroke (aHR, 1.2 [95% CI, 1.1-1.3]; RD per 1000 PYs, 1.8 [95% CI, 1.0-2.6] events), and death (aHR, 1.1 [95% CI, 1.1-1.2]; RD per 1000 PYs, 10.8 [95% CI, 7.8-13.8] events). The absolute mortality reduction associated with apixaban was greater in persons living with dementia vs those without dementia (RD per 1000 PYs, 44.4 [95% CI, 24.1-64.6] events vs 6.3 [95% CI, 3.6-9.1] events; *P* < .001 for heterogeneity). The risk reduction in major bleeding associated with apixaban was particularly distinct for GI bleeding but not evident for ICH, a pattern consistent across dementia subgroups (pooled results are shown in Figure 3 and eTable 36 in Supplement 1, and database-specific estimates are shown in eTables 37-39 in Supplement 1).

### Sensitivity Analyses

In all of the prespecified sensitivity analyses, we observed a consistent pattern; apixaban users had lower rates of the composite outcomes than users of other OACs, which was primarily due to fewer major bleeding outcomes. The magnitude of the benefit associated with apixaban was consistent across dementia status on the HR scale but substantially greater on the RD scale in those with vs without dementia. This pattern was observed when patients with dementia were further subdivided by use vs nonuse of dementia medications (eFigures 4-6 in Supplement 1), when death was included in the composite outcome, when HDPS was used for confounding adjustment, when as-treated analyses were conducted using various gaps between prescriptions to define discontinuation, when those with recent stroke were excluded, when those with a short-term SNF stay at baseline were excluded, when those receiving a reduced dose of a DOAC were excluded, and when the model was adjusted for US states (eTable 40 in Supplement 1).

## Discussion

In this comparative effectiveness study of adults 65 years or older with AF who were identified in 3 US nationwide health insurance databases, we observed that apixaban users had lower rates of ischemic strokes and intracranial and extracranial major bleeding compared with users of other OACs in the general population. The magnitude of the benefit associated with apixaban was similar regardless of dementia status on the relative scale but greater in terms of absolute rate reduction in patients with vs without dementia when compared with specific OACs for certain outcomes (particularly warfarin in relation to major bleeding). These increases in the absolute benefits of apixaban among patients with dementia reflect the substantially higher rates of poor outcomes in these patients.

Routine-care database studies of anticoagulation therapy among patients with AF using Medicare^52,53,54,55,56^ and commercial insurance claims^56,57,58,59,60,61^ have reported results that are generally consistent with those of RCTs.^15,16,17^ However, there are limited data comparing the benefits of OACs among persons living with dementia. None of the RCTs evaluated subgroup effects among persons living with dementia. One small observational study^62^ based on 2399 patients in the UK reported that DOAC use was associated with 2-fold increases in the risk of GI bleeding and overall death compared with warfarin. In this UK study,^62^ DOAC users were noted to be substantially older with more severe illness at baseline, and residual confounding was acknowledged as a possible explanation for the findings. Given the limited sample size, the previous study^62^ was not able to compare clinical outcomes by specific DOACs, which is suboptimal because patient characteristics and safety and effectiveness outcome incidence were all substantially different across DOACs in our analysis. We found that dabigatran and rivaroxaban users were younger and had a smaller comorbidity burden compared with apixaban users, who had baseline demographic and comorbidity profiles similar to warfarin users. In contrast to results from the UK study,^62^ our findings suggest apixaban is associated with lower major bleeding risks compared with all other OACs, which is consistent with findings from an RCT^17^ and other routine care database analyses^52,53,54,55,56,57,58,59,60,61^ conducted in general populations.

Examining the large number of major bleeding events associated with other OACs vs apixaban among persons living with dementia, the majority of major bleeding events involved extracranial bleeding, although intracranial bleeding is associated with a substantially higher risk (or incidence) of death and disability.^63^ It has been postulated that persons living with dementia could have an increased risk of cerebral amyloid angiopathy–mediated intracerebral hemorrhage.^64,65^ Yet, the ways in which different OACs may interact with this type of vulnerability is unclear. Among persons living with dementia, compared with apixaban users, warfarin users had a higher risk of ICH, but this higher risk was not observed in dabigatran or rivaroxaban users. In contrast, the risk reduction in major bleeding associated with apixaban was observed when compared with both warfarin and other DOACs (eTables 28-39 in Supplement 1). This pattern was similar across dementia subgroups.

Our results among persons living with dementia have provided important data about this vulnerable population of patients who have been substantially underrepresented in clinical trials. The association of OACs with significantly higher risks of major bleeding and ischemic stroke on the RD scale was proportional to the absolute event rates across dementia subgroups. Despite a substantially higher baseline rate of all outcomes among persons living with dementia, apixaban use was associated with a similar relative rate reduction regardless of dementia diagnosis, yielding a substantially higher absolute rate reduction compared with the other OACs. The fact that the absolute net clinical benefits associated with apixaban in comparison with other OACs were greater among persons living with dementia than those without dementia supports the use of apixaban in highly vulnerable patients with frailty.

### Limitations

This study has several strengths, including the use of large nationally representative databases of older adults in the US to achieve sufficient power that enabled detailed subgroup analysis by dementia status. We implemented a rigorous analytical framework with a new-user active-comparator design, which has been reported to successfully replicate RCT findings.^66^ In addition, we conducted a variety of sensitivity analyses and observed consistent results, which suggests our findings are robust.

This study also has limitations. First, the dementia subgroups could have been misclassified. We conducted a sensitivity analysis assessing the treatment effects of OACs among persons living with dementia who were using dementia medications and found consistent results. In addition, because this study was based on claims data, we were unable to measure clinical variables (eg, laboratory results) that may have implications for the effectiveness and safety of OACs or may confound associations. Therefore, unmeasured confounding remains possible. To mitigate unmeasured confounding, we applied a validated proxy confounding adjustment algorithm, HDPS,^44^ and found similar results. We also applied a validated CFI to account for frailty in our analysis. Another possible source of bias is misclassification of the OAC exposure during follow-up. Using a fixed gap between prescriptions to define treatment discontinuation can produce differential misclassification of the drug exposure given that the prescribing routine may be different for DOACs vs warfarin.^67^ Therefore, we conducted as-treated analyses varying the allowable gaps between prescriptions as sensitivity analyses, which yielded consistent results.

## Conclusions

In this comparative effectiveness study of OAC use among 3 US nationwide cohorts of older adults with AF, apixaban was consistently associated with lower rates of major bleeding and ischemic stroke compared with other OACs. The substantial risks associated with other OACs compared with apixaban were greater among patients with dementia than those without dementia on the absolute scale, particularly for major bleeding events. These results support the use of apixaban for anticoagulation therapy in vulnerable patients with frailty, particularly those living with dementia.

## Notes

Supplement 1. **eMethods.** Definitions of Exposures, Outcomes, Inclusions and Exclusions, Covariates, and Comorbidity Scores **eFigure 1.** Selection of Study Population for the Warfarin vs Apixaban Cohort Pooled Across Medicare, Optum, and MarketScan Populations **eFigure 2.** Selection of Study Population for the Dabigatran vs Apixaban Cohort Pooled Across Medicare, Optum, and MarketScan Populations **eFigure 3.** Selection of Study Population for the Rivaroxaban vs Apixaban Cohort Pooled Across Medicare, Optum, and MarketScan Populations **eFigure 4.** Sensitivity Analyses for Warfarin vs Apixaban in Older Adults With Atrial Fibrillation in the Medicare Population **eFigure 5.** Sensitivity Analyses for Dabigatran vs Apixaban in Older Adults With Atrial Fibrillation In the Medicare Population **eFigure 6.** Sensitivity Analyses for Rivaroxaban vs Apixaban in Older Adults With Atrial Fibrillation In the Medicare Population **eTable 1.** Propensity Score Models for Each Oral Anticoagulant vs Apixaban in the Medicare Population **eTable 2.** Propensity Score Models for Each Oral Anticoagulant vs Apixaban in the Optum Population **eTable 3.** Propensity Score Models for Each Oral Anticoagulant vs Apixaban in the MarketScan Population **eTable 4.** Study Population With Atrial Fibrillation Treated With Warfarin vs Apixaban Before and After 1:1 Propensity Score Matching Pooled Across Medicare, Optum, and MarketScan Populations **eTable 5.** Study Population With Atrial Fibrillation Treated With Warfarin vs Apixaban Before and After 1:1 Propensity Score Matching in the Medicare Population **eTable 6.** Study Population With Atrial Fibrillation Treated With Warfarin vs Apixaban Before and After 1:1 Propensity Score Matching in the Optum Population **eTable 7.** Study Population With Atrial Fibrillation Treated With Warfarin vs Apixaban Before and After 1:1 Propensity Score Matching in the MarketScan Population **eTable 8.** Study Population With Atrial Fibrillation Treated With Dabigatran vs Apixaban Before and After 1:1 Propensity Score Matching Pooled Across Medicare, Optum, and MarketScan Populations **eTable 9.** Study Population With Atrial Fibrillation Treated With Dabigatran vs Apixaban Before and After 1:1 Propensity Score Matching in the Medicare Population **eTable 10.** Study Population With Atrial Fibrillation Treated With Dabigatran vs Apixaban Before and After 1:1 Propensity Score Matching in the Optum Population **eTable 11.** Study Population With Atrial Fibrillation Treated With Dabigatran vs Apixaban Before and After 1:1 Propensity Score Matching in the MarketScan Population **eTable 12.** Study Population With Atrial Fibrillation Treated With Rivaroxaban vs Apixaban Before and After 1:1 Propensity Score Matching Pooled Across Medicare, Optum, and MarketScan Populations **eTable 13.** Study Population With Atrial Fibrillation Treated With Rivaroxaban vs Apixaban Before and After 1:1 Propensity Score Matching in the Medicare Population **eTable 14.** Study Population With Atrial Fibrillation Treated With Rivaroxaban vs Apixaban Before and After 1:1 Propensity Score Matching in the Optum Population **eTable 15.** Study Population With Atrial Fibrillation Treated With Rivaroxaban vs Apixaban Before and After 1:1 Propensity Score Matching in the MarketScan Population **eTable 16.** Selected Characteristics of Propensity Score-Matched Population With Atrial Fibrillation Treated With Warfarin vs Apixaban by Dementia Diagnosis Pooled Across Medicare, Optum, and MarketScan Populations **eTable 17.** Selected Characteristics of Propensity Score–Matched Population With Atrial Fibrillation Treated With Warfarin vs Apixaban by Dementia Diagnosis in the Medicare Population **eTable 18.** Selected Characteristics of Propensity Score–Matched Population With Atrial Fibrillation Treated With Warfarin vs Apixaban by Dementia Diagnosis in the Optum Population **eTable 19.** Selected Characteristics of Propensity Score–Matched Population With Atrial Fibrillation Treated With Warfarin vs Apixaban by Dementia Diagnosis in the MarketScan Population **eTable 20.** Selected Characteristics of Propensity Score–Matched Population With Atrial Fibrillation Treated With Dabigatran vs Apixaban by Dementia Diagnosis Pooled Across Medicare, Optum, and MarketScan Populations **eTable 21.** Selected Characteristics of Propensity Score–Matched Population With Atrial Fibrillation Treated With Dabigatran vs Apixaban by Dementia Diagnosis in the Medicare Population **eTable 22.** Selected Characteristics of Propensity Score–Matched Population With Atrial Fibrillation Treated With Dabigatran vs Apixaban by Dementia Diagnosis in the Optum Population **eTable 23.** Selected Characteristics of Propensity Score–Matched Population With Atrial Fibrillation Treated With Dabigatran vs Apixaban by Dementia Diagnosis in the MarketScan Population **eTable 24.** Selected Characteristics of Propensity Score–Matched Population With Atrial Fibrillation Treated With Rivaroxaban vs Apixaban by Dementia Diagnosis Pooled Across Medicare, Optum, and MarketScan Populations **eTable 25.** Selected Characteristics of Propensity Score–Matched Population With Atrial Fibrillation Treated With Rivaroxaban vs Apixaban by Dementia Diagnosis in the Medicare Population **eTable 26.** Selected Characteristics of Propensity Score–Matched Population With Atrial Fibrillation Treated With Rivaroxaban vs Apixaban by Dementia Diagnosis in the Optum Population **eTable 27.** Selected Characteristics of Propensity Score–Matched Population With Atrial Fibrillation Treated With Rivaroxaban vs Apixaban by Dementia Diagnosis in the MarketScan Population **eTable 28.** Dementia and Adverse Outcomes in the US Medicare Population With Atrial Fibrillation Treated With Warfarin vs Apixaban After 1:1 Propensity Score Matching Pooled Across Medicare, Optum, and MarketScan Populations **eTable 29.** Dementia and Adverse Outcomes in the US Medicare Population With Atrial Fibrillation Treated With Warfarin vs Apixaban After 1:1 Propensity Score Matching in the Medicare Population **eTable 30.** Dementia and Adverse Outcomes in the US Medicare Population With Atrial Fibrillation Treated With Warfarin vs Apixaban After 1:1 Propensity Score Matching in the Optum Population **eTable 31.** Dementia and Adverse Outcomes in the US Medicare Population With Atrial Fibrillation Treated With Warfarin vs Apixaban After 1:1 Propensity Score Matching in the MarketScan Population **eTable 32.** Dementia and Adverse Outcomes in the US Medicare Population With Atrial Fibrillation Treated With Warfarin vs Dabigatran After 1:1 Propensity Score Matching Pooled Across Medicare, Optum, and MarketScan Population **eTable 33.** Dementia and Adverse Outcomes in the US Medicare Population With Atrial Fibrillation Treated With Dabigatran vs Apixaban After 1:1 Propensity Score Matching in the Medicare Population **eTable 34.** Dementia and Adverse Outcomes in the US Medicare Population With Atrial Fibrillation Treated With Dabigatran vs Apixaban After 1:1 Propensity Score Matching in the Optum Population **eTable 35.** Dementia and Adverse Outcomes in the US Medicare Population With Atrial Fibrillation Treated With Dabigatran vs Apixaban After 1:1 Propensity Score Matching in the MarketScan Population **eTable 36.** Dementia and Adverse Outcomes in the US Medicare Population With Atrial Fibrillation Treated With Rivaroxaban vs Dabigatran After 1:1 Propensity Score Matching Pooled Across Medicare, Optum, and MarketScan Population **eTable 37.** Dementia and Adverse Outcomes in the US Medicare Population With Atrial Fibrillation Treated With Rivaroxaban vs Apixaban After 1:1 Propensity Score Matching in the Medicare Population **eTable 38.** Dementia and Adverse Outcomes in the US Medicare Population With Atrial Fibrillation Treated With Rivaroxaban vs Apixaban After 1:1 Propensity Score Matching in the Optum Population **eTable 39.** Dementia and Adverse Outcomes in the US Medicare Population With Atrial Fibrillation Treated With Rivaroxaban vs Apixaban After 1:1 Propensity Score Matching in the MarketScan Population **eTable 40.** Sensitivity Analyses for the Primary Outcome in Older Adults With Atrial Fibrillation in the Medicare Population

Supplement 2.
Data Sharing Statement
