# Supplementary material for: Comparative Effectiveness and Safety of Oral Anticoagulants by Dementia Status in Older Patients With Atrial Fibrillation
Source: JAMA Netw Open. 2023 Mar 28;6(3):e234086. doi: 10.1001/jamanetworkopen.2023.4086 (PMC10051113; doi:10.1001/jamanetworkopen.2023.4086)
Supplement: Supplement 1. — eMethods. Definitions of Exposures, Outcomes, Inclusions and Exclusions, Covariates, and Comorbidity Scores eFigure 1. Selection of Study Population for the Warfarin vs Apixaban Cohort Pooled Across Medicare, Optum, and MarketScan Populations eFigure 2. Selection of Study Population for the Dabigatran vs Apixaban Cohort Pooled Across Medicare, Optum, and MarketScan Populations eFigure 3. Selection of Study Population for the Rivaroxaban vs Apixaban Cohort Pooled Across Medicare, Optum, and MarketScan Populations eFigure 4. Sensitivity Analyses for Warfarin vs Apixaban in Older Adults With Atrial Fibrillation in the Medicare Population eFigure 5. Sensitivity Analyses for Dabigatran vs Apixaban in Older Adults With Atrial Fibrillation In the Medicare Population eFigure 6. Sensitivity Analyses for Rivaroxaban vs Apixaban in Older Adults With Atrial Fibrillation In the Medicare Population eTable 1. Propensity Score Models for Each Oral Anticoagulant vs Apixaban in the Medicare Population eTable 2. Propensity Score Models for Each Oral Anticoagulant vs Apixaban in the Optum Population eTable 3. Propensity Score Models for Each Oral Anticoagulant vs Apixaban in the MarketScan Population eTable 4. Study Population With Atrial Fibrillation Treated With Warfarin vs Apixaban Before and After 1:1 Propensity Score Matching Pooled Across Medicare, Optum, and MarketScan Populations eTable 5. Study Population With Atrial Fibrillation Treated With Warfarin vs Apixaban Before and After 1:1 Propensity Score Matching in the Medicare Population eTable 6. Study Population With Atrial Fibrillation Treated With Warfarin vs Apixaban Before and After 1:1 Propensity Score Matching in the Optum Population eTable 7. Study Population With Atrial Fibrillation Treated With Warfarin vs Apixaban Before and After 1:1 Propensity Score Matching in the MarketScan Population eTable 8. Study Population With Atrial Fibrillation Treated With Dabigatran vs Apixaban Before and After 1:1 Propensity Score Matc [file jamanetwopen-e234086-s001.pdf]

Supplementary Online Content

Lin KJ, Singer DE, Bykov K, et al. Comparative effectiveness and safety of oral anticoagulants by dementia status in older patients with atrial fibrillation. *JAMA Netw Open*. 2023;6(3):e234086. doi:10.1001/jamanetworkopen.2023.4086

eMethods. Definitions of Exposures, Outcomes, Inclusions and Exclusions, Covariates, and Comorbidity Scores

eFigure 1. Selection of Study Population for the Warfarin vs Apixaban Cohort Pooled Across Medicare, Optum, and MarketScan Populations

eFigure 2. Selection of Study Population for the Dabigatran vs Apixaban Cohort Pooled Across Medicare, Optum, and MarketScan Populations

eFigure 3. Selection of Study Population for the Rivaroxaban vs Apixaban Cohort Pooled Across Medicare, Optum, and MarketScan Populations

eFigure 4. Sensitivity Analyses for Warfarin vs Apixaban in Older Adults With Atrial Fibrillation in the Medicare Population

eFigure 5. Sensitivity Analyses for Dabigatran vs Apixaban in Older Adults With Atrial Fibrillation In the Medicare Population

eFigure 6. Sensitivity Analyses for Rivaroxaban vs Apixaban in Older Adults With Atrial Fibrillation In the Medicare Population

eTable 1. Propensity Score Models for Each Oral Anticoagulant vs Apixaban in the Medicare Population

eTable 2. Propensity Score Models for Each Oral Anticoagulant vs Apixaban in the Optum Population

eTable 3. Propensity Score Models for Each Oral Anticoagulant vs Apixaban in the MarketScan Population

eTable 4. Study Population With Atrial Fibrillation Treated With Warfarin vs Apixaban Before and After 1:1 Propensity Score Matching Pooled Across Medicare, Optum, and MarketScan Populations

eTable 5. Study Population With Atrial Fibrillation Treated With Warfarin vs Apixaban Before and After 1:1 Propensity Score Matching in the Medicare Population

eTable 6. Study Population With Atrial Fibrillation Treated With Warfarin vs Apixaban Before and After 1:1 Propensity Score Matching in the Optum Population

eTable 7. Study Population With Atrial Fibrillation Treated With Warfarin vs Apixaban Before and After 1:1 Propensity Score Matching in the MarketScan Population

eTable 8. Study Population With Atrial Fibrillation Treated With Dabigatran vs Apixaban Before and After 1:1 Propensity Score Matching Pooled Across Medicare, Optum, and MarketScan Populations

eTable 9. Study Population With Atrial Fibrillation Treated With Dabigatran vs Apixaban Before and After 1:1 Propensity Score Matching in the Medicare Population

eTable 10. Study Population With Atrial Fibrillation Treated With Dabigatran vs Apixaban Before and After 1:1 Propensity Score Matching in the Optum Population

© 2023 Lin KJ et al. *JAMA Network Open*.

eTable 11. Study Population With Atrial Fibrillation Treated With Dabigatran vs Apixaban Before and After 1:1 Propensity Score Matching in the MarketScan Population

eTable 12. Study Population With Atrial Fibrillation Treated With Rivaroxaban vs Apixaban Before and After 1:1 Propensity Score Matching Pooled Across Medicare, Optum, and MarketScan Populations

eTable 13. Study Population With Atrial Fibrillation Treated With Rivaroxaban vs Apixaban Before and After 1:1 Propensity Score Matching in the Medicare Population

eTable 14. Study Population With Atrial Fibrillation Treated With Rivaroxaban vs Apixaban Before and After 1:1 Propensity Score Matching in the Optum Population

eTable 15. Study Population With Atrial Fibrillation Treated With Rivaroxaban vs Apixaban Before and After 1:1 Propensity Score Matching in the MarketScan Population

eTable 16. Selected Characteristics of Propensity Score-Matched Population With Atrial Fibrillation Treated With Warfarin vs Apixaban by Dementia Diagnosis Pooled Across Medicare, Optum, and MarketScan Populations

eTable 17. Selected Characteristics of Propensity Score–Matched Population With Atrial Fibrillation Treated With Warfarin vs Apixaban by Dementia Diagnosis in the Medicare Population

eTable 18. Selected Characteristics of Propensity Score–Matched Population With Atrial Fibrillation Treated With Warfarin vs Apixaban by Dementia Diagnosis in the Optum Population

eTable 19. Selected Characteristics of Propensity Score–Matched Population With Atrial Fibrillation Treated With Warfarin vs Apixaban by Dementia Diagnosis in the MarketScan Population

eTable 20. Selected Characteristics of Propensity Score–Matched Population With Atrial Fibrillation Treated With Dabigatran vs Apixaban by Dementia Diagnosis Pooled Across Medicare, Optum, and MarketScan Populations

eTable 21. Selected Characteristics of Propensity Score–Matched Population With Atrial Fibrillation Treated With Dabigatran vs Apixaban by Dementia Diagnosis in the Medicare Population

eTable 22. Selected Characteristics of Propensity Score–Matched Population With Atrial Fibrillation Treated With Dabigatran vs Apixaban by Dementia Diagnosis in the Optum Population

eTable 23. Selected Characteristics of Propensity Score–Matched Population With Atrial Fibrillation Treated With Dabigatran vs Apixaban by Dementia Diagnosis in the MarketScan Population

eTable 24. Selected Characteristics of Propensity Score–Matched Population With Atrial Fibrillation Treated With Rivaroxaban vs Apixaban by Dementia Diagnosis Pooled Across Medicare, Optum, and MarketScan Populations

eTable 25. Selected Characteristics of Propensity Score–Matched Population With Atrial Fibrillation Treated With Rivaroxaban vs Apixaban by Dementia Diagnosis in the Medicare Population

eTable 26. Selected Characteristics of Propensity Score–Matched Population With Atrial Fibrillation Treated With Rivaroxaban vs Apixaban by Dementia Diagnosis in the Optum Population

© 2023 Lin KJ et al. *JAMA Network Open*.

eTable 27. Selected Characteristics of Propensity Score–Matched Population With Atrial Fibrillation Treated With Rivaroxaban vs Apixaban by Dementia Diagnosis in the MarketScan Population

eTable 28. Dementia and Adverse Outcomes in the US Medicare Population With Atrial Fibrillation Treated With Warfarin vs Apixaban After 1:1 Propensity Score Matching Pooled Across Medicare, Optum, and MarketScan Populations

eTable 29. Dementia and Adverse Outcomes in the US Medicare Population With Atrial Fibrillation Treated With Warfarin vs Apixaban After 1:1 Propensity Score Matching in the Medicare Population

eTable 30. Dementia and Adverse Outcomes in the US Medicare Population With Atrial Fibrillation Treated With Warfarin vs Apixaban After 1:1 Propensity Score Matching in the Optum Population

eTable 31. Dementia and Adverse Outcomes in the US Medicare Population With Atrial Fibrillation Treated With Warfarin vs Apixaban After 1:1 Propensity Score Matching in the MarketScan Population

eTable 32. Dementia and Adverse Outcomes in the US Medicare Population With Atrial Fibrillation Treated With Warfarin vs Dabigatran After 1:1 Propensity Score Matching Pooled Across Medicare, Optum, and MarketScan Population

eTable 33. Dementia and Adverse Outcomes in the US Medicare Population With Atrial Fibrillation Treated With Dabigatran vs Apixaban After 1:1 Propensity Score Matching in the Medicare Population

eTable 34. Dementia and Adverse Outcomes in the US Medicare Population With Atrial Fibrillation Treated With Dabigatran vs Apixaban After 1:1 Propensity Score Matching in the Optum Population

eTable 35. Dementia and Adverse Outcomes in the US Medicare Population With Atrial Fibrillation Treated With Dabigatran vs Apixaban After 1:1 Propensity Score Matching in the MarketScan Population

eTable 36. Dementia and Adverse Outcomes in the US Medicare Population With Atrial Fibrillation Treated With Rivaroxaban vs Dabigatran After 1:1 Propensity Score Matching Pooled Across Medicare, Optum, and MarketScan Population

eTable 37. Dementia and Adverse Outcomes in the US Medicare Population With Atrial Fibrillation Treated With Rivaroxaban vs Apixaban After 1:1 Propensity Score Matching in the Medicare Population

eTable 38. Dementia and Adverse Outcomes in the US Medicare Population With Atrial Fibrillation Treated With Rivaroxaban vs Apixaban After 1:1 Propensity Score Matching in the Optum Population

eTable 39. Dementia and Adverse Outcomes in the US Medicare Population With Atrial Fibrillation Treated With Rivaroxaban vs Apixaban After 1:1 Propensity Score Matching in the MarketScan Population

eTable 40. Sensitivity Analyses for the Primary Outcome in Older Adults With Atrial Fibrillation in the Medicare Population

This supplemental material has been provided by the authors to give readers additional information about their work.

© 2023 Lin KJ et al. *JAMA Network Open*.

eMethods. Definitions of Exposures, Outcomes, Inclusions and Exclusions, Covariates, and Comorbidity Scores

Exposure Definitions

Exposure	NDC Generic Name
Apixaban	APIXABAN
Dabigatran	DABIGATRAN ETEXILATE MESYLATE
Rivaroxaban	RIVAROXABAN
Warfarin	WARFARIN SODIUM

Outcome Definitions

Outcome	Codes/Definition
Composite Major Bleeding or Ischemic Stroke	The occurrence of either of the following:<br>• Ischemic stroke<br>• Major bleeding
Major Bleeding	**Primary position, inpatient ICD-9 Diagnosis Code is any of:** 336.1, 363.61, 363.72, 376.32, 430, 432, 432.9, 568.81, 719.1, 719.10, 719.15, 719.18, 852, 852.0, 852.00, 852.04, 852.05, 852.10, 852.11, 852.12, 852.16, 852.20, 852.21, 852.22, 852.23, 852.25, 852.29, 852.3, 852.31, 852.32, 852.35, 852.36, 852.40, 852.43, 852.5, 852.51, 852.52, 852.53, 852.54, 852.59, 853.0, 853.00, 853.04, 853.05, 853.09, 853.10, 853.11, 853.13, 853.19, 866.02, 866.11, 866.12, 363.62, 377.42, 379.23, 423.0, 431, 432.0, 432.1, 719.11, 719.12, 719.13, 719.14, 719.16, 719.17, 719.19, 729.92, 852.01, 852.02, 852.03, 852.06, 852.09, 852.1, 852.13, 852.14, 852.15, 852.19, 852.2, 852.24, 852.26, 852.30, 852.33, 852.34, 852.39, 852.4, 852.41, 852.42, 852.44, 852.45, 852.46, 852.49, 852.50, 852.55, 852.56, 853, 853.01, 853.02, 853.03, 853.06, 853.1, 853.12, 853.14, 853.15, 853.16, 866.01<br><br>**Primary position, inpatient ICD-10 Diagnosis Code is any of:** G95.19, H05.23, H05.231, H05.232, H05.239, H31.303, H31.311, H31.312, H31.319, H31.412, H31.419, H43.11, H43.12, H47.029, I31.2, I60.0, I60.01, I60.10, I60.11, I60.3, I60.31, I60.5, I60.51, I60.52, I60.6, I60.8, I61, I61.4, I61.8, I62.1, K66.1, M25.011, M25.012, M25.02, M25.022, M25.03, M25.032, M25.04, M25.041, M25.042, M25.049, M25.05, M25.059, M25.06, M25.061, M25.062, M25.069, M25.071, M25.074, M25.076, M25.08, S06.4X0D, S06.4X0S, S06.4X1, S06.4X1S, S06.4X2, S06.4X2D, S06.4X3, S06.4X3A, S06.4X4, S06.4X4A, S06.4X5S, S06.4X6, S06.4X6A, S06.4X6D, S06.4X6S, S06.4X7, S06.4X7A, S06.4X8A, S06.5X, S06.5X0, S06.5X0S, S06.5X1, S06.5X1A, S06.5X1S, S06.5X2, S06.5X2D, S06.5X2S, S06.5X3, S06.5X3A, S06.5X3D, S06.5X3S, S06.5X4, S06.5X4A, S06.5X4D, S06.5X5D, S06.5X6A, S06.5X6S, S06.5X8, S06.5X8A, S06.5X9A, S06.6, S06.6X0, S06.6X0D, S06.6X1, S06.6X1A, S06.6X1S, S06.6X2, S06.6X2D, S06.6X2S, S06.6X3, S06.6X3A, S06.6X3D, S06.6X4S, S06.6X5A, S06.6X5S, S06.6X6D, S06.6X7A, S06.6X8, S06.6X8A, S06.6X9S, S26.00, S26.00XS, S26.01, S26.01XS, S26.020D, S26.021, S26.021D, S26.021S, S26.022, S26.022A, S26.022S, S26.09XA, S26.09XD, S37.01, S37.011S, S37.012D, S37.021, S37.021A, S37.021D, S37.022, S37.029D, S37.029S, S37.03, S37.031, S37.031A, S37.031S, S37.032S, S37.039D, S37.041S, S37.042, S37.042A, S37.049A, S37.051A, S37.051D, S37.051S, S37.052D, S37.052S, S37.059, S37.059D, S37.06, S37.061, S37.061D, S37.062, S37.062A, S37.062D, S37.062S, S37.069, S37.069A, S37.069S, H05.233, H31.30, H31.301, H31.302, H31.309, H31.31, H31.313, H31.41, H31.411, H31.413, H43.1, H43.10, H43.13, H47.02, H47.021, H47.022, H47.023, I23.0, I60, I60.00, I60.02, I60.1, I60.12, I60.2, I60.30, I60.32, I60.4, I60.50, I60.7, I60.9, I61.0, I61.1, I61.2, I61.3, I61.5, I61.6, I61.9, I62, I62.0, I62.00, I62.01, I62.02, I62.03, I62.9, M25.0, M25.00, M25.01, M25.019, M25.021, M25.029, M25.031, M25.039, M25.051, M25.052, M25.07, M25.072, M25.073, M25.075, S06.4, S06.4X, S06.4X0, S06.4X0A, S06.4X1A, S06.4X1D, S06.4X2A, S06.4X2S, S06.4X3D, S06.4X3S, S06.4X4D, S06.4X4S, S06.4X5, S06.4X5A, S06.4X5D, S06.4X8, S06.4X9, S06.4X9A, S06.4X9D, S06.4X9S, S06.5, S06.5X0A, S06.5X0D, S06.5X1D, S06.5X2A, S06.5X4S, S06.5X5, S06.5X5A, S06.5X5S, S06.5X6, S06.5X6D, S06.5X7, S06.5X7A, S06.5X9, S06.5X9D, S06.5X9S, S06.6X, S06.6X0A, S06.6X0S, S06.6X1D, S06.6X2A, S06.6X3S, S06.6X4, S06.6X4A, S06.6X4D, S06.6X5, S06.6X5D, S06.6X6, S06.6X6A, S06.6X6S, S06.6X7, S06.6X9, S06.6X9A, S06.6X9D, S26.0, S26.00XA, S26.00XD, S26.01XA, S26.01XD, S26.02, S26.020, S26.020A, S26.020S, S26.021A, S26.022D, S26.09, S26.09XS, S37.011, S37.011A, S37.011D, S37.012, S37.012A, S37.012S, S37.019, S37.019A, S37.019D,

© 2023 Lin KJ et al. *JAMA Network Open*.

	S37.019S, S37.02, S37.021S, S37.022A, S37.022D, S37.022S, S37.029, S37.029A, S37.031D, S37.032, S37.032A, S37.032D, S37.039, S37.039A, S37.039S, S37.04, S37.041, S37.041A, S37.041D, S37.042D, S37.042S, S37.049, S37.049D, S37.049S, S37.05, S37.051, S37.052, S37.052A, S37.059A, S37.059S, S37.061A, S37.061S, S37.069D, M79.81<br><br>**ICD-9 Procedure Code (any position) is any of:** 99.04, 99.05, 99.03, 99.06 **with Primary ICD-9 Diagnosis Code is any of:** 285.1, 455.2, 455.5, 455.6, 455.7, 455.8, 456.0, 530.1, 530.11, 530.13, 530.19, 530.7, 531.3, 531.40, 531.5, 531.60, 531.61, 532.20, 532.3, 532.4, 532.41, 532.5, 532.6, 532.61, 532.9, 533.0, 533.00, 533.01, 533.20, 533.3, 533.40, 533.6, 533.60, 533.61, 533.7, 534.01, 534.2, 534.3, 534.4, 534.40, 534.41, 534.5, 534.6, 534.9, 535.00, 535.11, 535.20, 535.30, 535.41, 535.50, 535.61, 562.00, 562.02, 562.03, 562.10, 562.11, 562.12, 562.13, 569.3, 569.85, 578.1, 599.70, 599.71, 623.6, 623.8, 626.6, 626.8, 784.7, 784.8, 786.3, 786.30, 786.39, 280.0, 455.1, 455.4, 455.9, 456.20, 459.0, 530.10, 530.12, 530.82, 531.0, 531.00, 531.01, 531.1, 531.2, 531.20, 531.21, 531.4, 531.41, 531.6, 531.7, 531.9, 532.0, 532.00, 532.01, 532.1, 532.2, 532.21, 532.40, 532.60, 532.7, 533.1, 533.2, 533.21, 533.4, 533.41, 533.5, 533.9, 534.0, 534.00, 534.1, 534.20, 534.21, 534.60, 534.61, 534.7, 535.01, 535.10, 535.21, 535.31, 535.40, 535.51, 535.60, 537.83, 562.01, 578.0, 578.9, 593.81, 786.31<br><br>**HCPCS Procedure Code (any position) is any of:** P9010, P9016, P9017, P9019, P9022, P9032, P9036, P9038, P9039, P9051, P9052, P9054, P9055, P9056, P9058, P9059, P9060, P9011, P9020, P9021, P9023, P9031, P9033, P9034, P9035, P9037, P9040, P9044, P9053, P9057, P9070, P9071 **with Primary ICD-9 Diagnosis Code is any of:** 285.1, 455.2, 455.5, 455.6, 455.7, 455.8, 456.0, 530.1, 530.11, 530.13, 530.19, 530.7, 531.3, 531.40, 531.5, 531.60, 531.61, 532.20, 532.3, 532.4, 532.41, 532.5, 532.6, 532.61, 532.9, 533.0, 533.00, 533.01, 533.20, 533.3, 533.40, 533.6, 533.60, 533.61, 533.7, 534.01, 534.2, 534.3, 534.4, 534.40, 534.41, 534.5, 534.6, 534.9, 535.00, 535.11, 535.20, 535.30, 535.41, 535.50, 535.61, 562.00, 562.02, 562.03, 562.10, 562.11, 562.12, 562.13, 569.3, 569.85, 578.1, 599.70, 599.71, 623.6, 623.8, 626.6, 626.8, 784.7, 784.8, 786.3, 786.30, 786.39, 280.0, 455.1, 455.4, 455.9, 456.20, 459.0, 530.10, 530.12, 530.82, 531.0, 531.00, 531.01, 531.1, 531.2, 531.20, 531.21, 531.4, 531.41, 531.6, 531.7, 531.9, 532.0, 532.00, 532.01, 532.1, 532.2, 532.21, 532.40, 532.60, 532.7, 533.1, 533.2, 533.21, 533.4, 533.41, 533.5, 533.9, 534.0, 534.00, 534.1, 534.20, 534.21, 534.60, 534.61, 534.7, 535.01, 535.10, 535.21, 535.31, 535.40, 535.51, 535.60, 537.83, 562.01, 578.0, 578.9, 593.81, 786.31<br><br>**Revenue Center Code is any of:** 0387, 0391, 0381, 0382, 0383, 0384 **with Primary ICD-9 Diagnosis Code is any of:** 285.1, 455.2, 455.5, 455.6, 455.7, 455.8, 456.0, 530.1, 530.11, 530.13, 530.19, 530.7, 531.3, 531.40, 531.5, 531.60, 531.61, 532.20, 532.3, 532.4, 532.41, 532.5, 532.6, 532.61, 532.9, 533.0, 533.00, 533.01, 533.20, 533.3, 533.40, 533.6, 533.60, 533.61, 533.7, 534.01, 534.2, 534.3, 534.4, 534.40, 534.41, 534.5, 534.6, 534.9, 535.00, 535.11, 535.20, 535.30, 535.41, 535.50, 535.61, 562.00, 562.02, 562.03, 562.10, 562.11, 562.12, 562.13, 569.3, 569.85, 578.1, 599.70, 599.71, 623.6, 623.8, 626.6, 626.8, 784.7, 784.8, 786.3, 786.30, 786.39, 280.0, 455.1, 455.4, 455.9, 456.20, 459.0, 530.10, 530.12, 530.82, 531.0, 531.00, 531.01, 531.1, 531.2, 531.20, 531.21, 531.4, 531.41, 531.6, 531.7, 531.9, 532.0, 532.00, 532.01, 532.1, 532.2, 532.21, 532.40, 532.60, 532.7, 533.1, 533.2, 533.21, 533.4, 533.41, 533.5, 533.9, 534.0, 534.00, 534.1, 534.20, 534.21, 534.60, 534.61, 534.7, 535.01, 535.10, 535.21, 535.31, 535.40, 535.51, 535.60, 537.83, 562.01, 578.0, 578.9, 593.81, 786.31<br><br>**ICD-10 Procedure Code (any position) is any of:** 30230P1, 30230T1, 30233H1, 30233M1, 30233R1, 30233W1, 30240K1, 30240W1, 30243H1, 30243M1, 30243N1, 30243R1, 30243T1, 30243V1, 30230H1, 30230K1, 30230L1, 30230M1, 30230N1, 30230R1, 30230V1, 30230W1, 30233K1, 30233L1, 30233N1, 30233P1, 30233T1, 30233V1, 30240H1, 30240L1, 30240M1, 30240N1, 30240P1, 30240R1, 30240T1, 30240V1, 30243K1, 30243L1, 30243P1, 30243W1 **with Primary ICD-10 Diagnosis Code is any of:** D50.0, D62, I85.11, K20, K20.8, K21.0, K22.11, K22.6, K25.0, K25.4, K25.6, K26.1, K26.4, K26.5, K26.6, K27.2, K27.5, K27.6, K28.0, K28.4, K28.5, K29.01, K29.21, K29.41, K31.811, K55.21, K57.01, K57.21, K57.51, K57.53, K57.93, K64.0, K64.5, K64.8, K64.9, K92.0, N89.7, N92.1, N93.9, R04.0, R04.1, R04.2, R31.0, R31.9, R58, I85.01, K20.0, K20.9, K25.1, K25.2, K25.5, K26.0, K26.2, K27.0, K27.1, K27.4, K28.1, K28.2, K28.6,

© 2023 Lin KJ et al. *JAMA Network Open*.

	K29.31, K29.51, K29.61, K29.71, K29.81, K29.91, K57.11, K57.31, K57.41, K57.81, K57.91, K62.5, K64.1, K64.2, K64.3, K64.4, K92.1, K92.2, N89.8, N93.8<br><br>**HCPCS Procedure Code (any position) is any of:** P9010, P9016, P9017, P9019, P9022, P9032, P9036, P9038, P9039, P9051, P9052, P9054, P9055, P9056, P9058, P9059, P9060, P9011, P9020, P9021, P9023, P9031, P9033, P9034, P9035, P9037, P9040, P9044, P9053, P9057, P9070, P9071 **with Primary ICD-10 Diagnosis Code is any of:** D50.0, D62, I85.11, K20, K20.8, K21.0, K22.11, K22.6, K25.0, K25.4, K25.6, K26.1, K26.4, K26.5, K26.6, K27.2, K27.5, K27.6, K28.0, K28.4, K28.5, K29.01, K29.21, K29.41, K31.811, K55.21, K57.01, K57.21, K57.51, K57.53, K57.93, K64.0, K64.5, K64.8, K64.9, K92.0, N89.7, N92.1, N93.9, R04.0, R04.1, R04.2, R31.0, R31.9, R58, I85.01, K20.0, K20.9, K25.1, K25.2, K25.5, K26.0, K26.2, K27.0, K27.1, K27.4, K28.1, K28.2, K28.6, K29.31, K29.51, K29.61, K29.71, K29.81, K29.91, K57.11, K57.31, K57.41, K57.81, K57.91, K62.5, K64.1, K64.2, K64.3, K64.4, K92.1, K92.2, N89.8, N93.8<br><br>**Revenue Center Code is any of:** 0387, 0391, 0381, 0382, 0383, 0384 **with Primary ICD-10 Diagnosis Code is any of:** D50.0, D62, I85.11, K20, K20.8, K21.0, K22.11, K22.6, K25.0, K25.4, K25.6, K26.1, K26.4, K26.5, K26.6, K27.2, K27.5, K27.6, K28.0, K28.4, K28.5, K29.01, K29.21, K29.41, K31.811, K55.21, K57.01, K57.21, K57.51, K57.53, K57.93, K64.0, K64.5, K64.8, K64.9, K92.0, N89.7, N92.1, N93.9, R04.0, R04.1, R04.2, R31.0, R31.9, R58, I85.01, K20.0, K20.9, K25.1, K25.2, K25.5, K26.0, K26.2, K27.0, K27.1, K27.4, K28.1, K28.2, K28.6, K29.31, K29.51, K29.61, K29.71, K29.81, K29.91, K57.11, K57.31, K57.41, K57.81, K57.91, K62.5, K64.1, K64.2, K64.3, K64.4, K92.1, K92.2, N89.8, N93.8
Ischemic Stroke	**Primary position, inpatient** ICD-9 Diagnosis Code is any of: 433.01, 433.11, 433.21, 433.31, 434.01, 434.11, 362.31, 362.32, 362.33, 362.34, 433.81, 433.91, 436<br>Primary ICD-10 Diagnosis Code is any of: H34.0, H34.00, H34.01, H34.02, H34.03, H34.1, H34.10, H34.11, H34.13, H34.2, H34.21, H34.213, H34.23, H34.231, H34.239, I63.011, I63.012, I63.031, I63.112, I63.12, I63.132, I63.139, I63.19, I63.2, I63.21, I63.212, I63.213, I63.219, I63.22, I63.231, I63.232, I63.233, I63.239, I63.29, I63.30, I63.31, I63.311, I63.32, I63.322, I63.329, I63.33, I63.339, I63.34, I63.341, I63.342, I63.349, I63.39, I63.40, I63.411, I63.419, I63.422, I63.429, I63.431, I63.433, I63.49, I63.50, I63.51, I63.52, I63.523, I63.529, I63.531, I63.533, I63.541, I63.542, I63.543, I63.8, I63.9, H34.12, H34.211, H34.212, H34.219, H34.232, H34.233, I63, I63.0, I63.00, I63.01, I63.013, I63.019, I63.02, I63.03, I63.032, I63.033, I63.039, I63.09, I63.1, I63.10, I63.11, I63.111, I63.113, I63.119, I63.13, I63.131, I63.133, I63.20, I63.211, I63.23, I63.3, I63.312, I63.313, I63.319, I63.321, I63.323, I63.331, I63.332, I63.333, I63.343, I63.4, I63.41, I63.412, I63.413, I63.42, I63.421, I63.423, I63.43, I63.432, I63.439, I63.44, I63.441, I63.442, I63.443, I63.449, I63.5, I63.511, I63.512, I63.513, I63.519, I63.521, I63.522, I63.53, I63.532, I63.539, I63.54, I63.549, I63.59, I63.6
All-Cause Mortality	**Death** recorded by **Master Beneficiary Summary File** or **Inpatient** Discharge Status Code is any of: 20, 40, 41, 42
Major GI Bleeding	ICD-9 Procedure Code (any position) is any of: 99.04, 99.05, 99.03, 99.06 with Primary ICD-9 Diagnosis Code is any of: 455.2, 455.5, 455.6, 455.7, 455.8, 456.0, 530.1, 530.11, 530.13, 530.19, 530.7, 531.3, 531.40, 531.5, 531.60, 531.61, 532.20, 532.3, 532.4, 532.41, 532.5, 532.6, 532.61, 532.9, 533.0, 533.00, 533.01, 533.20, 533.3, 533.40, 533.6, 533.60, 533.61, 533.7, 534.01, 534.2, 534.3, 534.4, 534.40, 534.41, 534.5, 534.6, 534.9, 535.00, 535.11, 535.20, 535.30, 535.41, 535.50, 535.61, 562.00, 562.02, 562.03, 562.10, 562.11, 562.12, 562.13, 569.3, 569.85, 578.1, 455.1, 455.4, 455.9, 456.20, 530.10, 530.12, 530.82, 531.0, 531.00, 531.01, 531.1, 531.2, 531.20, 531.21, 531.4, 531.41, 531.6, 531.7, 531.9, 532.0, 532.00, 532.01, 532.1, 532.2, 532.21, 532.40, 532.60, 532.7, 533.1, 533.2, 533.21, 533.4, 533.41, 533.5, 533.9, 534.0, 534.00, 534.1, 534.20, 534.21, 534.60, 534.61, 534.7, 535.01, 535.10, 535.21, 535.31, 535.40, 535.51, 535.60, 537.83, 562.01, 578.0, 578.9<br><br>HCPCS Procedure Code (any position) is any of: P9010, P9016, P9017, P9019, P9022, P9032, P9036, P9038, P9039, P9051, P9052, P9054, P9055, P9056, P9058, P9059, P9060, P9011, P9020, P9021, P9023, P9031, P9033, P9034, P9035, P9037, P9040, P9044, P9053, P9057, P9070, P9071 with Primary ICD-9 Diagnosis Code is any of: 455.2, 455.5, 455.6, 455.7, 455.8, 456.0, 530.1, 530.11, 530.13, 530.19, 530.7, 531.3, 531.40, 531.5, 531.60, 531.61, 532.20, 532.3, 532.4, 532.41, 532.5, 532.6, 532.61, 532.9, 533.0, 533.00, 533.01, 533.20, 533.3, 533.40, 533.6, 533.60, 533.61, 533.7, 534.01, 534.2, 534.3, 534.4, 534.40, 534.41, 534.5, 534.6, 534.9, 535.00, 535.11, 535.20,

© 2023 Lin KJ et al. *JAMA Network Open*.

	535.30, 535.41, 535.50, 535.61, 562.00, 562.02, 562.03, 562.10, 562.11, 562.12, 562.13, 569.3, 569.85, 578.1, 455.1, 455.4, 455.9, 456.20, 530.10, 530.12, 530.82, 531.0, 531.00, 531.01, 531.1, 531.2, 531.20, 531.21, 531.4, 531.41, 531.6, 531.7, 531.9, 532.0, 532.00, 532.01, 532.1, 532.2, 532.21, 532.40, 532.60, 532.7, 533.1, 533.2, 533.21, 533.4, 533.41, 533.5, 533.9, 534.0, 534.00, 534.1, 534.20, 534.21, 534.60, 534.61, 534.7, 535.01, 535.10, 535.21, 535.31, 535.40, 535.51, 535.60, 537.83, 562.01, 578.0, 578.9<br><br>Revenue Center Code is any of: 0387, 0391, 0381, 0382, 0383, 0384 with Primary ICD-9 Diagnosis Code is any of: 455.2, 455.5, 455.6, 455.7, 455.8, 456.0, 530.1, 530.11, 530.13, 530.19, 530.7, 531.3, 531.40, 531.5, 531.60, 531.61, 532.20, 532.3, 532.4, 532.41, 532.5, 532.6, 532.61, 532.9, 533.0, 533.00, 533.01, 533.20, 533.3, 533.40, 533.6, 533.60, 533.61, 533.7, 534.01, 534.2, 534.3, 534.4, 534.40, 534.41, 534.5, 534.6, 534.9, 535.00, 535.11, 535.20, 535.30, 535.41, 535.50, 535.61, 562.00, 562.02, 562.03, 562.10, 562.11, 562.12, 562.13, 569.3, 569.85, 578.1, 455.1, 455.4, 455.9, 456.20, 530.10, 530.12, 530.82, 531.0, 531.00, 531.01, 531.1, 531.2, 531.20, 531.21, 531.4, 531.41, 531.6, 531.7, 531.9, 532.0, 532.00, 532.01, 532.1, 532.2, 532.21, 532.40, 532.60, 532.7, 533.1, 533.2, 533.21, 533.4, 533.41, 533.5, 533.9, 534.0, 534.00, 534.1, 534.20, 534.21, 534.60, 534.61, 534.7, 535.01, 535.10, 535.21, 535.31, 535.40, 535.51, 535.60, 537.83, 562.01, 578.0, 578.9<br><br>ICD-10 Procedure Code (any position) is any of: 30230P1, 30230T1, 30233H1, 30233M1, 30233R1, 30233W1, 30240K1, 30240W1, 30243H1, 30243M1, 30243N1, 30243R1, 30243T1, 30243V1, 30230H1, 30230K1, 30230L1, 30230M1, 30230N1, 30230R1, 30230V1, 30230W1, 30233K1, 30233L1, 30233N1, 30233P1, 30233T1, 30233V1, 30240H1, 30240L1, 30240M1, 30240N1, 30240P1, 30240R1, 30240T1, 30240V1, 30243K1, 30243L1, 30243P1, 30243W1 with Primary ICD-10 Diagnosis Code is any of: I85.11, K20, K20.8, K21.0, K22.11, K22.6, K25.0, K25.4, K25.6, K26.1, K26.4, K26.5, K26.6, K27.2, K27.5, K27.6, K28.0, K28.4, K28.5, K29.01, K29.21, K29.41, K31.811, K55.21, K57.01, K57.21, K57.51, K57.53, K57.93, K64.0, K64.5, K64.8, K64.9, K92.0, I85.01, K20.0, K20.9, K25.1, K25.2, K25.5, K26.0, K26.2, K27.0, K27.1, K27.4, K28.1, K28.2, K28.6, K29.31, K29.51, K29.61, K29.71, K29.81, K29.91, K57.11, K57.31, K57.41, K57.81, K57.91, K62.5, K64.1, K64.2, K64.3, K64.4, K92.1, K92.2<br><br>HCPCS Procedure Code (any position) is any of: P9010, P9016, P9017, P9019, P9022, P9032, P9036, P9038, P9039, P9051, P9052, P9054, P9055, P9056, P9058, P9059, P9060, P9011, P9020, P9021, P9023, P9031, P9033, P9034, P9035, P9037, P9040, P9044, P9053, P9057, P9070, P9071 with Primary ICD-10 Diagnosis Code is any of: I85.11, K20, K20.8, K21.0, K22.11, K22.6, K25.0, K25.4, K25.6, K26.1, K26.4, K26.5, K26.6, K27.2, K27.5, K27.6, K28.0, K28.4, K28.5, K29.01, K29.21, K29.41, K31.811, K55.21, K57.01, K57.21, K57.51, K57.53, K57.93, K64.0, K64.5, K64.8, K64.9, K92.0, I85.01, K20.0, K20.9, K25.1, K25.2, K25.5, K26.0, K26.2, K27.0, K27.1, K27.4, K28.1, K28.2, K28.6, K29.31, K29.51, K29.61, K29.71, K29.81, K29.91, K57.11, K57.31, K57.41, K57.81, K57.91, K62.5, K64.1, K64.2, K64.3, K64.4, K92.1, K92.2<br><br>Revenue Center Code is any of: 0387, 0391, 0381, 0382, 0383, 0384 with Primary ICD-10 Diagnosis Code is any of: I85.11, K20, K20.8, K21.0, K22.11, K22.6, K25.0, K25.4, K25.6, K26.1, K26.4, K26.5, K26.6, K27.2, K27.5, K27.6, K28.0, K28.4, K28.5, K29.01, K29.21, K29.41, K31.811, K55.21, K57.01, K57.21, K57.51, K57.53, K57.93, K64.0, K64.5, K64.8, K64.9, K92.0, I85.01, K20.0, K20.9, K25.1, K25.2, K25.5, K26.0, K26.2, K27.0, K27.1, K27.4, K28.1, K28.2, K28.6, K29.31, K29.51, K29.61, K29.71, K29.81, K29.91, K57.11, K57.31, K57.41, K57.81, K57.91, K62.5, K64.1, K64.2, K64.3, K64.4, K92.1, K92.2
Intracranial bleed	**Primary position, inpatient** ICD-9 Diagnosis Code is any of: 430, 432, 432.9, 852, 852.0, 852.00, 852.04, 852.05, 852.10, 852.11, 852.12, 852.16, 852.20, 852.21, 852.22, 852.23, 852.25, 852.29, 852.3, 852.31, 852.32, 852.35, 852.36, 852.40, 852.43, 852.5, 852.51, 852.52, 852.53, 852.54, 852.59, 853.0, 853.00, 853.04, 853.05, 853.09, 853.10, 853.11, 853.13, 853.19, 431, 432.0, 432.1, 852.01, 852.02, 852.03, 852.06, 852.09, 852.1, 852.13, 852.14, 852.15, 852.19, 852.2, 852.24, 852.26, 852.30, 852.33, 852.34, 852.39, 852.4, 852.41, 852.42, 852.44, 852.45, 852.46, 852.49, 852.50, 852.55, 852.56, 853, 853.01, 853.02, 853.03, 853.06, 853.1, 853.12, 853.14, 853.15, 853.16<br>**Primary position, inpatient** ICD-10 Diagnosis Code is any of: I60.0, I60.01, I60.10, I60.11, I60.3, I60.31, I60.5, I60.51, I60.52, I60.6, I60.8, I61, I61.4, I61.8, I62.1, S06.4X0D, S06.4X0S, S06.4X1, S06.4X1S, S06.4X2, S06.4X2D, S06.4X3, S06.4X3A, S06.4X4,

© 2023 Lin KJ et al. *JAMA Network Open*.

	S06.4X4A, S06.4X5S, S06.4X6, S06.4X6A, S06.4X6D, S06.4X6S, S06.4X7, S06.4X7A, S06.4X8A, S06.5X, S06.5X0, S06.5X0S, S06.5X1, S06.5X1A, S06.5X1S, S06.5X2, S06.5X2D, S06.5X2S, S06.5X3, S06.5X3A, S06.5X3D, S06.5X3S, S06.5X4, S06.5X4A, S06.5X4D, S06.5X5D, S06.5X6A, S06.5X6S, S06.5X8, S06.5X8A, S06.5X9A, S06.6, S06.6X0, S06.6X0D, S06.6X1, S06.6X1A, S06.6X1S, S06.6X2, S06.6X2D, S06.6X2S, S06.6X3, S06.6X3A, S06.6X3D, S06.6X4S, S06.6X5A, S06.6X5S, S06.6X6D, S06.6X7A, S06.6X8, S06.6X8A, S06.6X9S, I60, I60.00, I60.02, I60.1, I60.12, I60.2, I60.30, I60.32, I60.4, I60.50, I60.7, I60.9, I61.0, I61.1, I61.2, I61.3, I61.5, I61.6, I61.9, I62, I62.0, I62.00, I62.01, I62.02, I62.03, I62.9, S06.4, S06.4X, S06.4X0, S06.4X0A, S06.4X1A, S06.4X1D, S06.4X2A, S06.4X2S, S06.4X3D, S06.4X3S, S06.4X4D, S06.4X4S, S06.4X5, S06.4X5A, S06.4X5D, S06.4X8, S06.4X9, S06.4X9A, S06.4X9D, S06.4X9S, S06.5, S06.5X0A, S06.5X0D, S06.5X1D, S06.5X2A, S06.5X4S, S06.5X5, S06.5X5A, S06.5X5S, S06.5X6, S06.5X6D, S06.5X7, S06.5X7A, S06.5X9, S06.5X9D, S06.5X9S, S06.6X, S06.6X0A, S06.6X0S, S06.6X1D, S06.6X2A, S06.6X3S, S06.6X4, S06.6X4A, S06.6X4D, S06.6X5, S06.6X5D, S06.6X6, S06.6X6A, S06.6X6S, S06.6X7, S06.6X9, S06.6X9A, S06.6X9D

Inclusion/Exclusion Definitions

Inclusion/Exclusion	Codes/Definition
Atrial fibrillation and flutter	ICD-9 Diagnosis Code (any position) is any of: 427.3, 427.31, 427.32<br>ICD-10 Diagnosis Code (any position) is any of: I48.0, I48.3, I48.92, I48, I48.1, I48.2, I48.4, I48.9, I48.91
Hospice Stay	Hospice claim or Inpatient stay with Patient Discharge Status = 50 or 51
Joint replacement	ICD-9 Procedure Code (any position) is any of: 00.70, 00.71, 00.72, 00.73, 81.51, 81.52, 81.53, 00.80, 00.81, 00.82, 00.83, 00.84, 81.54, 81.55, 81.80, 81.81, 81.88, 81.5, 81.59, 81.97<br>ICD-10 Procedure Code (any position) is any of: 0RRE0KZ, 0RRJ07Z, 0RRK0J6, 0RRK0KZ, 0RWL3JZ, 0RWL4JZ, 0RWN3JZ, 0RWQ3JZ, 0RWR0JZ, 0RWU0JZ, 0RWW0JZ, 0SPA0JZ, 0SPS0JZ, 0SPW0JZ, 0SR906Z, 0SR90KZ, 0SRA039, 0SRA07Z, 0SRA0KZ, 0SRB02Z, 0SRB06Z, 0SRB07Z, 0SRB0J9, 0SRB0JZ, 0SRC0JZ, 0SRD069, 0SRE00Z, 0SRE01A, 0SRE0J9, 0SRE0JA, 0SRR03A, 0SRS01Z, 0SRS03A, 0SRS03Z, 0SRS0JA, 0SRT0KZ, 0SRU0JA, 0SRV0JA, 0SRW0JA, 0SRW0JZ, 0SW94JZ, 0SWC0JZ, 0SWC4JC, 0SWD3JC, 0SWD4JC, 0SWG0JZ, 0SWG3JZ, 0SWP4JZ, 0SWS3JZ, 0SWT4JZ, 0SWU0JZ, 0SWV0JZ, 0SWW0JZ, 0SWW4JZ, 0RRE0JZ, 0RRF07Z, 0RRF0KZ, 0RRJ0J6, 0RRJ0J7, 0RRK0J7, 0RRK0JZ, 0RWH4JZ, 0RWK0JZ, 0RWK3JZ, 0RWK4JZ, 0RWM3JZ, 0RWM4JZ, 0RWN4JZ, 0RWP4JZ, 0RWQ0JZ, 0RWT0JZ, 0RWU3JZ, 0RWV3JZ, 0SPC0JZ, 0SPU0JZ, 0SR9039, 0SR907Z, 0SRA00Z, 0SRA01A, 0SRA0JZ, 0SRB019, 0SRB01A, 0SRC069, 0SRC07Z, 0SRC0LZ, 0SRD0LA, 0SRD0LZ, 0SRE009, 0SRE0JZ, 0SRS019, 0SW93JZ, 0SWA3JZ, 0SWA4JZ, 0SWC0JC, 0SWC3JC, 0SWD3JZ, 0SWF0JZ, 0SWF3JZ, 0SWF4JZ, 0SWJ3JZ, 0SWK0JZ, 0SWK3JZ, 0SWL3JZ, 0SWM0JZ, 0SWM4JZ, 0SWN0JZ, 0SWR0JZ, 0SWW3JZ, 0RRF0JZ, 0RRG0KZ, 0RRH0JZ, 0RRJ00Z, 0RRK00Z, 0RRK07Z, 0RWG0JZ, 0RWG3JZ, 0RWH3JZ, 0RWJ0JZ, 0RWL0JZ, 0RWM0JZ, 0RWP0JZ, 0RWT4JZ, 0RWV4JZ, 0SPC48Z, 0SPC4JZ, 0SPD08Z, 0SPD0JZ, 0SPD4JZ, 0SPR0JZ, 0SPT0JZ, 0SR902A, 0SR902Z, 0SR903A, 0SR903Z, 0SR904Z, 0SR90JZ, 0SRA009, 0SRA0J9, 0SRB01Z, 0SRB03A, 0SRB04Z, 0SRB069, 0SRC06A, 0SRD06Z, 0SRD07Z, 0SRD0J9, 0SRE01Z, 0SRE039, 0SRE03A, 0SRR03Z, 0SRS0JZ, 0SRS0KZ, 0SRT0JZ, 0SRU07Z, 0SRU0J9, 0SRU0JZ, 0SRU0KZ, 0SRV07Z, 0SRV0J9, 0SRW07Z, 0SRW0J9, 0SRW0KZ, 0SWB3JZ, 0SWB4JZ, 0SWD0JZ, 0SWD4JZ, 0SWH0JZ, 0SWH4JZ, 0SWL0JZ, 0SWL4JZ, 0SWM3JZ, 0SWP0JZ, 0SWQ0JZ, 0SWQ3JZ, 0SWQ4JZ, 0SWR3JZ, 0SWR4JZ, 0SWV3JZ, 0SWV4JZ, 0RRE07Z, 0RRH0KZ, 0RRJ0JZ, 0RWJ3JZ, 0RWJ4JZ, 0RWS3JZ, 0RWW4JZ, 0RWX0JZ, 0SPD48Z, 0SPV0JZ, 0SR9019, 0SR901Z, 0SR904A, 0SR906A, 0SRA019, 0SRA03A, 0SRB029, 0SRB03Z, 0SRB049, 0SRB06A, 0SRB0JA, 0SRC0LA, 0SRD06A, 0SRD0JA, 0SRD0KZ, 0SRD0L9, 0SRE019, 0SRE03Z, 0SRR01A, 0SRR01Z, 0SRR0J9, 0SRR0JA, 0SRR0KZ, 0SRS01A, 0SRS039, 0SRT07Z, 0SRT0JA, 0SRV0KZ, 0SWB0JZ, 0SWC4JZ, 0SWE4JZ, 0SWG4JZ, 0SWH3JZ, 0SWJ0JZ, 0SWK4JZ, 0SWN3JZ, 0SWP3JZ, 0SWS0JZ, 0SWT3JZ, 0SWU4JZ, 0RRG07Z, 0RRG0JZ, 0RRH07Z, 0RRJ0KZ, 0RWG4JZ, 0RWH0JZ, 0RWN0JZ, 0RWP3JZ, 0RWQ4JZ, 0RWR3JZ, 0RWR4JZ, 0RWS0JZ, 0RWS4JZ, 0RWT3JZ, 0RWU4JZ, 0RWV0JZ, 0RWW3JZ, 0RWX3JZ, 0RWX4JZ, 0SPC08Z, 0SPE0JZ, 0SR901A, 0SR9029, 0SR9049, 0SR9069, 0SR90J9, 0SR90JA, 0SRA00A, 0SRA01Z, 0SRA03Z, 0SRA0JA, 0SRB02A, 0SRB039, 0SRB04A, 0SRB0KZ, 0SRC06Z, 0SRC0J9, 0SRC0JA, 0SRC0KZ, 0SRC0L9, 0SRD0JZ, 0SRE00A, 0SRE07Z, 0SRE0KZ, 0SRR019, 0SRR039, 0SRR07Z, 0SRR0JZ, 0SRS07Z, 0SRS0J9, 0SRT0J9, 0SRV0JZ, 0SW90JZ, 0SWA0JZ, 0SWC3JZ, 0SWD0JC, 0SWE0JZ, 0SWE3JZ, 0SWJ4JZ, 0SWN4JZ, 0SWS4JZ, 0SWT0JZ, 0SWU3JZ

© 2023 Lin KJ et al. *JAMA Network Open*.

Pulmonary Embolism	ICD-9 Diagnosis Code (any position) is any of: 415.11, 415.12, 415.19, 415.1, 415.10, 415.199, 415.15, 415.16, 415.17, 415.13<br>ICD-10 Diagnosis Code (any position) is any of: I26.01, I26.09, I26.92, I26.0, I26.99, I26.02, I26.9, I26.90
Deep Vein Thrombosis	ICD-9 Diagnosis Code (any position) is any of: 451.0, 451.1, 451.11, 451.19, 451.2, 451.8, 451.81, 451.82, 451.83, 451.84, 451.89, 451.9, 453.0, 453.1, 453.2, 453.3, 453.4, 453.40, 453.41, 453.42, 453.5, 453.50, 453.51, 453.52, 453.6, 453.7, 453.71, 453.72, 453.73, 453.74, 453.75, 453.76, 453.77, 453.79, 453.8, 453.81, 453.82, 453.83, 453.84, 453.85, 453.86, 453.87, 453.89, 453.9<br>ICD-10 Diagnosis Code (any position) is any of: I80.222, I80.229, I80.232, I80.233, I80.29, I80.293, I82.21, I82.220, I82.29, I82.290, I82.402, I82.43, I82.432, I82.443, I82.491, I82.4Y3, I82.602, I82.611, I82.90, I82.A19, I82.B1, I80.21, I80.9, I82.401, I82.409, I82.411, I82.419, I82.42, I82.421, I82.422, I82.429, I82.439, I82.44, I82.441, I82.492, I82.4Z, I82.4Z3, I82.612, I82.619, I82.622, I82.B11, I82.C11, I82.C13, I82.C19, I80.201, I80.203, I80.211, I80.212, I80.213, I80.22, I80.223, I80.23, I80.231, I82.22, I82.4, I82.403, I82.412, I82.442, I82.493, I82.4Z2, I82.60, I82.601, I82.613, I82.623, I82.890, I82.A11, I82.A12, I82.A13, I80.2, I80.20, I80.219, I80.221, I80.291, I82.1, I82.2, I82.40, I82.413, I82.431, I82.433, I82.4Y1, I82.4Y9, I82.4Z9, I82.61, I82.62, I82.B13, I82.B19, I82.C12, I80.202, I80.209, I80.239, I80.292, I80.299, I80.3, I82.0, I82.210, I82.41, I82.423, I82.449, I82.49, I82.499, I82.4Y, I82.4Y2, I82.4Z1, I82.6, I82.603, I82.609, I82.621, I82.629, I82.A1, I82.B12, I82.C1<br><br>ICD-10 Diagnosis Code (any position) is any of: T81.718A, T80.0XXA, T81.72XA, T82.817A, T82.818A with ICD-10 Diagnosis Code (any position) is any of: I26.99, I26.90
Evidence of Valvular Disease	ICD-9 Diagnosis Code (any position) is any of: 394.0, 394.1, 394.2, 394.9, 395.0, 395.1, 395.2, 395.9, 396.0, 396.1, 396.2, 396.3, 396.8, 396.9, 397.0, 397.1, 397.9, 398.90, 398.91, 398.99, V42.2, V43.3<br>ICD-10 Diagnosis Code (any position) is any of: I05.2, I06.9, I07.2, I08.0, I08.3, I05.1, I05.8, I06.0, I07.8, I08.8, I09.89, I05.9, I06.1, I06.2, I07.9, I09.9, I05.0, I07.0, I08.1, I08.2, I08.9, I09.81, Z95.2, Z95.3, I06.8, I07.1, I09.1, Z95.4<br>HCPCS Procedure Code (any position) is any of: 0257T, 0258T, 0259T, 0262T, 33400, 33401, 33403, 33420, 33422, 33425, 33426, 33427, 33430, 33460, 33463, 33464, 33465, 33468, 33475, 33496, 33660, 33665
Stage 5 Chronic Kidney Disease, End Stage Renal Disease, or Dialysis	ICD-9 Diagnosis Code (any position) is any of: 403.11, 403.91, V45.1, V56.1, V56.31, V56.8, 403.01, 585.5, 585.6, V45.11, V45.12, V56, V56.0, V56.2, V56.3, V56.32<br>ICD-10 Diagnosis Code (any position) is any of: I12.0, I13.11, Y84.1, Z49, Z49.0, Z49.02, Z49.32, I13.2, N18.5, Z49.01, Z49.3, Z49.31, Z91.15, Z99.2<br>ICD-9 Procedure Code (any position) is any of: 39.95, 54.98<br>ICD-10 Procedure Code (any position) is any of: 3E1M39Z, 5A1D70Z, 5A1D90Z, 5A1D80Z<br>HCPCS Procedure Code (any position) is any of: 90935, 90940, 90941, 90942, 90945, 90955, 90959, 90962, 90963, 90964, 90965, 90969, 90985, 99559, 90937, 90939, 90947, 90951, 90952, 90953, 90954, 90956, 90957, 90958, 90960, 90961, 90966, 90967, 90968, 90970, 90989, 90990, 90993, 99512
Liver Disease	ICD-9 Diagnosis Code (any position) is any of: 571.5, 571.6, 570, 571.2, 572.8 with ICD-9 Diagnosis Code (any position) is any of: 456.0, 456.21, 567.21, 567.89, 572.4, 789.59, 456.1, 456.20, 567.0, 567.29, 567.9, 572.2<br><br>ICD-10 Diagnosis Code (any position) is any of: K70.2, K70.31, K72.9, K72.90, K74.69, K70.30, K72.1, K74.0, K74.3, K74.4, K74.5, K74.60 with ICD-10 Diagnosis Code (any position) is any of: I85.11, I86.4, K65.8, K65.9, K70.41, K71.11, K72.01, K72.91, K76.7, R18.8, I85.00, I85.01, I85.10, K65.0, K67, K72.11<br><br>ICD-9 Diagnosis Code (any position) is any of: 570<br>ICD-10 Diagnosis Code (any position) is any of: K72, K72.0, K72.00, K72.01, K72.9, K72.90, K72.91, K76.2
Major Bleeding	Primary ICD-9 Diagnosis Code is any of: 336.1, 363.61, 363.63, 372.72, 376.32, 719.15, 719.18, 866.02, 866.11, 866.12, 363.62, 377.42, 379.23, 719.11, 719.12, 719.13, 719.14, 719.16, 719.17, 719.19, 729.92, 866.01, 432, 852.0, 852.00, 852.10, 852.20, 852.3, 852.40, 852.5, 853.0, 853.00, 853.10, 432.0, 852.1, 852.2, 852.30, 852.4, 852.50, 853.1, 430, 432.9, 852.04, 852.05, 852.11, 852.12, 852.16, 852.21, 852.22, 852.23, 852.25, 852.29, 852.31, 852.32, 852.35, 852.36, 852.43, 852.51, 852.52, 852.53, 852.54, 852.59, 853.04, 853.05, 853.09, 853.11, 853.13, 853.19, 431, 432.1, 852.01, 852.02, 852.03, 852.06, 852.09, 852.13, 852.14, 852.15, 852.19, 852.24,

© 2023 Lin KJ et al. *JAMA Network Open*.

	852.26, 852.33, 852.34, 852.39, 852.41, 852.42, 852.44, 852.45, 852.46, 852.49, 852.55, 852.56, 853.01, 853.02, 853.03, 853.06, 853.12, 853.14, 853.15, 853.16<br><br>Primary ICD-10 Diagnosis Code is any of: G95.19, H05.231, H05.232, H05.239, H11.30, H31.303, H31.311, H31.312, H31.319, H31.322, H31.323, H43.11, H43.12, H47.029, M25.011, M25.012, M25.022, M25.032, M25.041, M25.042, M25.049, M25.059, M25.061, M25.062, M25.069, M25.071, M25.074, M25.076, M25.08, M79.81, S31.001A, S37.031A, S37.042A, S37.049A, S37.051A, H05.233, H11.31, H11.32, H11.33, H31.301, H31.302, H31.309, H31.313, H31.321, H31.329, H43.10, H43.13, H47.021, H47.022, H47.023, M25.00, M25.019, M25.021, M25.029, M25.031, M25.039, M25.051, M25.052, M25.072, M25.073, M25.075, S37.032A, S37.039A, S37.041A, S37.052A, S37.059A, I60.01, I60.10, I60.11, I60.31, I60.51, I60.52, I60.6, I60.8, I61.4, I61.8, S06.340A, S06.341A, S06.344A, S06.345A, S06.346A, S06.347A, S06.349A, S06.351A, S06.353A, S06.354A, S06.357A, S06.358A, S06.361A, S06.364A, S06.365A, S06.366A, S06.368A, S06.369A, S06.4X3A, S06.4X4A, S06.4X6A, S06.4X7A, S06.4X8A, S06.5X1A, S06.5X3A, S06.5X4A, S06.5X6A, S06.5X8A, S06.5X9A, S06.6X1A, S06.6X3A, S06.6X5A, S06.6X7A, S06.6X8A, I60.00, I60.02, I60.12, I60.2, I60.30, I60.32, I60.4, I60.50, I60.7, I60.9, I61.0, I61.1, I61.2, I61.3, I61.5, I61.6, I61.9, I62.00, I62.01, I62.02, I62.03, I62.9, S06.342A, S06.343A, S06.348A, S06.350A, S06.352A, S06.355A, S06.356A, S06.359A, S06.360A, S06.362A, S06.363A, S06.367A, S06.4X0A, S06.4X1A, S06.4X2A, S06.4X5A, S06.4X9A, S06.5X0A, S06.5X2A, S06.5X5A, S06.5X7A, S06.6X0A, S06.6X2A, S06.6X4A, S06.6X6A, S06.6X9A<br><br>HCPCS Procedure Code (any position) is any of: P9010, P9011, P9016, P9017, P9019, P9020, P9021, P9022, P9023, P9044, P9051, P9052, P9053, P9054, P9055, P9056, P9057, P9058, P9059, P9060, P9031, P9032, P9033, P9034, P9035, P9036, P9037, P9038, P9039, P9040 with Primary ICD-9 Diagnosis Code is any of: 280.0, 285.1, 285.9, 423.0, 455.0, 455.1, 455.2, 455.3, 455.4, 455.5, 455.6, 455.7, 455.8, 455.9, 456.0, 456.20, 459.0, 530.1, 530.7, 530.82, 531.00, 531.01, 531.1, 531.20, 531.21, 531.3, 531.40, 531.41, 531.5, 531.60, 531.61, 531.7, 531.9, 532.00, 532.01, 532.1, 532.20, 532.21, 532.3, 532.40, 532.41, 532.5, 532.60, 532.61, 532.7, 532.9, 533.00, 533.01, 533.1, 533.20, 533.21, 533.3, 533.40, 533.41, 533.5, 533.60, 533.61, 533.7, 533.9, 534.00, 534.01, 534.1, 534.20, 534.21, 534.3, 534.40, 534.41, 534.5, 534.60, 534.61, 534.7, 534.9, 535.00, 535.01, 535.10, 535.11, 535.20, 535.21, 535.30, 535.31, 535.40, 535.41, 535.50, 535.51, 535.60, 535.61, 537.83, 562.00, 562.01, 562.02, 562.03, 562.10, 562.11, 562.12, 562.13, 568.81, 569.3, 569.85, 578.0, 578.1, 578.9, 599.7, 623.8, 626.2, 626.6, 784.7, 784.8, 786.3, 790.92<br><br>ICD-9 Procedure Code (any position) is any of: 99.03, 99.04, 99.05, 99.06 with Primary ICD-9 Diagnosis Code is any of: 280.0, 285.1, 285.9, 423.0, 455.0, 455.1, 455.2, 455.3, 455.4, 455.5, 455.6, 455.7, 455.8, 455.9, 456.0, 456.20, 459.0, 530.1, 530.7, 530.82, 531.00, 531.01, 531.1, 531.20, 531.21, 531.3, 531.40, 531.41, 531.5, 531.60, 531.61, 531.7, 531.9, 532.00, 532.01, 532.1, 532.20, 532.21, 532.3, 532.40, 532.41, 532.5, 532.60, 532.61, 532.7, 532.9, 533.00, 533.01, 533.1, 533.20, 533.21, 533.3, 533.40, 533.41, 533.5, 533.60, 533.61, 533.7, 533.9, 534.00, 534.01, 534.1, 534.20, 534.21, 534.3, 534.40, 534.41, 534.5, 534.60, 534.61, 534.7, 534.9, 535.00, 535.01, 535.10, 535.11, 535.20, 535.21, 535.30, 535.31, 535.40, 535.41, 535.50, 535.51, 535.60, 535.61, 537.83, 562.00, 562.01, 562.02, 562.03, 562.10, 562.11, 562.12, 562.13, 568.81, 569.3, 569.85, 578.0, 578.1, 578.9, 599.7, 623.8, 626.2, 626.6, 784.7, 784.8, 786.3, 790.92<br><br>Revenue Center Code is any of: 0381, 0382, 0383, 0384, 0387 with Primary ICD-9 Diagnosis Code is any of: 280.0, 285.1, 285.9, 423.0, 455.2, 455.5, 455.8, 456.0, 456.20, 459.0, 530.1, 530.7, 530.82, 531.1, 531.3, 531.5, 531.7, 531.9, 532.1, 532.3, 532.5, 532.7, 532.9, 533.1, 533.3, 533.5, 533.7, 533.9, 534.1, 534.3, 534.5, 534.7, 534.9, 535.00, 535.01, 535.10, 535.11, 535.20, 535.21, 535.30, 535.31, 535.40, 535.41, 535.50, 535.51, 535.60, 535.61, 537.83, 562.00, 562.01, 562.02, 562.03, 562.10, 562.11, 562.12, 562.13, 568.81, 569.3, 569.85, 578.0, 578.1, 578.9, 599.7, 623.8, 626.2, 626.6, 784.7, 784.8, 786.3, 790.92, 455.0, 455.1, 455.3, 455.4, 455.6, 455.7, 455.9, 531.00, 531.01, 531.20, 531.21, 531.40, 531.41, 531.60, 531.61, 532.00, 532.01, 532.20, 532.21, 532.40, 532.41, 532.60, 532.61, 533.00, 533.01, 533.20, 533.21, 533.40, 533.41, 533.60, 533.61, 534.00, 534.01, 534.20, 534.21, 534.40, 534.41, 534.60, 534.61

© 2023 Lin KJ et al. *JAMA Network Open*.

	ICD-10 Procedure Code (any position) is any of: 30230P1, 30230R1, 30233K1, 30233M1, 30233N1, 30233P1, 30233V1, 30233W1, 30240M1, 30240N1, 30240V1, 30240W1, 30243L1, 30243T1, 30243V1, 30243W1, 30250K1, 30250L1, 30250N1, 30250P1, 30250V1, 30250W1, 30253H1, 30253P1, 30253R1, 30253W1, 30260L1, 30260M1, 30260N1, 30260P1, 30260V1, 30260W1, 30263N1, 30263P1, 30263T1, 30230H1, 30230K1, 30230L1, 30230M1, 30230N1, 30230T1, 30230V1, 30230W1, 30233H1, 30233L1, 30233Q1, 30233R1, 30233T1, 30240H1, 30240K1, 30240L1, 30240P1, 30240R1, 30240T1, 30243H1, 30243K1, 30243M1, 30243N1, 30243P1, 30243Q1, 30243R1, 30250H1, 30250M1, 30250R1, 30250T1, 30253K1, 30253L1, 30253M1, 30253N1, 30253T1, 30253V1, 30260H1, 30260K1, 30260R1, 30260T1, 30263H1, 30263K1, 30263L1, 30263M1, 30263R1, 30263V1, 30263W1 with Primary ICD-10 Diagnosis Code is any of: D62, K22.6, K25.0, K25.4, K25.6, K26.4, K26.6, K27.2, K27.6, K28.0, K28.4, K29.01, K29.21, K29.41, K31.811, K55.21, K57.01, K57.12, K57.21, K57.33, K57.40, K57.51, K57.52, K57.53, K57.93, K64.0, K64.8, K64.9, K66.1, N92.0, N92.1, R04.0, R04.1, D64.9, K22.8, K25.2, K26.0, K26.2, K27.0, K27.4, K28.2, K28.6, K29.31, K29.51, K29.61, K29.71, K29.81, K29.91, K57.00, K57.11, K57.13, K57.20, K57.31, K57.32, K57.41, K57.80, K57.81, K57.91, K57.92, K62.5, K64.1, K64.2, K64.3, K64.4, K92.1, K92.2, R79.1<br><br>HCPCS Procedure Code (any position) is any of: P9016, P9017, P9019, P9020, P9039, P9052, P9053, P9057, P9059, P9060, P9010, P9031, P9032, P9035, P9037, P9038, P9055, P9021, P9033, P9036, P9056, P9011, P9023, P9034, P9054, P9058, P9022, P9040, P9044, P9051 with Primary ICD-10 Diagnosis Code is any of: D62, K22.6, K25.0, K25.4, K25.6, K26.4, K26.6, K27.2, K27.6, K28.0, K28.4, K29.01, K29.21, K29.41, K31.811, K55.21, K57.01, K57.12, K57.21, K57.33, K57.40, K57.51, K57.52, K57.53, K57.93, K64.0, K64.8, K64.9, K66.1, N92.0, N92.1, R04.0, R04.1, D64.9, K22.8, K25.2, K26.0, K26.2, K27.0, K27.4, K28.2, K28.6, K29.31, K29.51, K29.61, K29.71, K29.81, K29.91, K57.00, K57.11, K57.13, K57.20, K57.31, K57.32, K57.41, K57.80, K57.81, K57.91, K57.92, K62.5, K64.1, K64.2, K64.3, K64.4, K92.1, K92.2, R79.1<br><br>Revenue Center Code is any of: 0387, 0381, 0382, 0383, 0384 with Primary ICD-10 Diagnosis Code is any of: D62, K22.6, K25.0, K25.4, K25.6, K26.4, K26.6, K27.2, K27.6, K28.0, K28.4, K29.01, K29.21, K29.41, K31.811, K55.21, K57.01, K57.12, K57.21, K57.33, K57.40, K57.51, K57.52, K57.53, K57.93, K64.0, K64.8, K64.9, K66.1, N92.0, N92.1, R04.0, R04.1, D64.9, K22.8, K25.2, K26.0, K26.2, K27.0, K27.4, K28.2, K28.6, K29.31, K29.51, K29.61, K29.71, K29.81, K29.91, K57.00, K57.11, K57.13, K57.20, K57.31, K57.32, K57.41, K57.80, K57.81, K57.91, K57.92, K62.5, K64.1, K64.2, K64.3, K64.4, K92.1, K92.2, R79.1

Covariate Definitions

Covariate	Codes/Definition
Dementia	ICD-9 Diagnosis Code (any position) is any of: 290.0, 290.1, 290.11, 290.13, 290.20, 290.21, 290.42, 290.43, 294.11, 294.21, 294.8, 331.7, 290.12, 290.3, 290.40, 290.41, 294.0, 294.10, 294.20, 331.0, 331.11, 331.19, 331.2, 797<br>ICD-10 Diagnosis Code (any position) is any of: F01.50, F01.51, F03.91, G30.1, G30.8, G30.9, G31.01, G31.09, F02.80, F02.81, F03.90, G30.0, F01.5, F02.8, F03.9, G31.0
Dual Status	Categorized as true if Dual Status Code is 03, 04, or 08
Acute renal failure	ICD-9 Diagnosis Code (any position) is any of: 584, 584.5, 584.6, 584.7, 584.8, 584.9<br>ICD-10 Diagnosis Code (any position) is any of: N17.8, N17.9, N17.2, N17.0, N19, N17.1
Acute Myocardial Infarction	ICD-9 Diagnosis Code (any position) is any of: 410, 410.0, 410.00, 410.01, 410.02, 410.1, 410.10, 410.11, 410.12, 410.2, 410.20, 410.21, 410.22, 410.3, 410.30, 410.31, 410.32, 410.4, 410.40, 410.41, 410.42, 410.5, 410.50, 410.51, 410.52, 410.6, 410.60, 410.61, 410.62, 410.7, 410.70, 410.71, 410.72, 410.8, 410.80, 410.81, 410.82, 410.9, 410.90, 410.91, 410.92<br>ICD-10 Diagnosis Code is any of: I21.09, I22.1, I22.8, I21.29, I21.01, I21.19, I21.02, I21.3, I22.0, I22.2, I21.11, I21.21, I21.4, I22.9
Alcohol Abuse or Dependence	ICD-9 Diagnosis Code (any position) is any of: 291.2, 291.5, 291.89, 305.03, 571.2, 571.3, 291.9, 303.02, 305.01, 571.1, 291.1, 291.8, 291.81, 305.02, 571.0, E860.0, 291.0, 291.4, 291.82, 303.0, 303.00, 303.03, 303.90, 303.92, 305.0, 305.00, 425.5, 291.3, 303.01, 303.9, 303.91, 303.93, 357.5, V11.3<br>ICD-10 Diagnosis Code (any position) is any of: F10.129, F10.150, F10.19, F10.20, F10.221, F10.230, F10.250, F10.921, F10.96, F10.982, F10.99, T51.0X1D, T51.0X1S, V11.3, F10.180, F10.21, F10.220, F10.229, F10.231, F10.232, F10.26, F10.121, F10.182,

© 2023 Lin KJ et al. *JAMA Network Open*.

	F10.188, F10.251, F10.259, F10.94, F10.959, F10.97, F10.981, K70.10, K70.41, F10.10, F10.120, F10.151, F10.239, F10.27, F10.280, F10.282, F10.920, F10.951, G62.1, I42.6, K70.0, K70.11, K70.2, K70.9, F10.14, F10.159, F10.181, F10.24, F10.281, F10.288, F10.29, F10.929, F10.950, F10.980, F10.988, K70.30, K70.31, K70.40, T51.0X1A
Anemia	ICD-9 Diagnosis Code (any position) is any of: 281.4, 282, 283.9, 284.0, 285.29, 280, 281.1, 281.2, 282.2, 282.8, 283, 283.1, 283.19, 284, 284.89, 285.1, 285.2, 285.9, 280.1, 281.3, 281.8, 282.3, 283.10, 280.0, 280.8, 280.9, 281.0, 281.9, 283.0, 284.09, 285, 285.21, 285.22, 285.3, 285.8, 281, 282.9, 284.8, 284.9, 285.0<br>ICD-10 Diagnosis Code (any position) is any of: D50, D50.0, D50.8, D51.2, D52, D52.0, D56.1, D57.0, D57.4, D57.411, D57.81, D58.2, D60.1, D61.1, D61.82, D62, D64, D64.1, D51.9, D52.9, D53.0, D53.8, D55.2, D55.9, D56.5, D56.9, D57.21, D57.219, D57.812, D57.819, D58.0, D58.1, D59.0, D59.4, D59.5, D60.0, D60.8, D61.81, D61.810, D61.811, D61.89, D64.2, D51.1, D51.3, D52.1, D52.8, D55.3, D56, D57.20, D57.211, D57.212, D57.412, D58.8, D59.1, D59.8, D59.9, D61.2, D64.0, D64.3, D64.81, D50.1, D50.9, D51, D55, D55.8, D56.2, D56.3, D57.00, D57.01, D57.02, D57.2, D57.41, D57.419, D57.8, D57.80, D57.811, D58.9, D59, D60, D61, D61.0, D61.01, D61.3, D61.8, D61.818, D61.9, D51.0, D51.8, D53, D53.1, D53.2, D53.9, D55.0, D55.1, D56.0, D56.4, D56.8, D57, D57.1, D57.3, D57.40, D58, D59.2, D59.3, D59.6, D60.9, D61.09, D64.4, D64.8, D64.89, D64.9
Cardio-ablation	ICD-9 Procedure Code (any position) is any of: 37.34<br>ICD-10 Procedure Code (any position) is any of: 02593ZZ, 025J3ZZ, 025K3ZZ, 025M3ZZ, 02B83ZZ, 02T83ZZ, 025H3ZZ, 02B73ZZ, 02BG3ZZ, 025L3ZZ, 02BK3ZZ, 02BL3ZZ, 02563ZZ, 02573ZZ, 025F3ZZ, 025G3ZZ, 02B93ZZ, 02BH3ZZ, 02BJ3ZZ, 02553ZZ, 02583ZZ, 02B53ZZ, 02B63ZZ, 02BF3ZZ, 02BM3ZZ
Cardioversion	ICD-9 Procedure Code (any position) is any of: 99.61<br>ICD-10 Procedure Code (any position) is any of: 5A2204Z<br>HCPCS Procedure Code (any position) is any of: 92960, 92961
Chronic Kidney Disease	ICD-9 Diagnosis Code (any position) is any of: 585.3, 585.4, 585.9<br>ICD-10 Diagnosis Code (any position) is any of: N18.3, N18.9, N18.4
Congestive heart failure	ICD-9 Diagnosis Code (any position) is any of: 398.91, 402.01, 402.11, 402.91, 404.01, 404.03, 404.11, 404.13, 404.91, 404.93, 428.0, 428.1, 428.2, 428.20, 428.21, 428.22, 428.23, 428.3, 428.30, 428.31, 428.32, 428.33, 428.4, 428.40, 428.41, 428.42, 428.43, 428.9, 425.0, 425.9, 429.3, 425.18, 428, 425.1, 425.3, 425.5, 425.7, 425, 425.11, 425.2, 425.4, 425.8<br>ICD-10 Diagnosis Code (any position) is any of: I42.0, I42.4, I43, I50.1, I50.4, I50.42, I42, I42.2, I42.3, I42.6, I42.7, I42.8, I50.32, I11.0, I42.5, I42.9, I50.2, I50.20, I50.21, I50.43, A18.84, I50, I50.23, I50.3, I50.30, I50.40, I50.41, I50.9, I42.1, I50.22, I50.31, I50.33
Coronary revascularization (PTCA, stenting, CABG)	ICD-9 Procedure Code (any position) is any of: 00.66, 36.03, 36.11, 36.12, 36.32, 36.10, 36.17, 36.34, 36.06, 36.07, 36.09, 36.14, 36.15, 36.16, 36.19, 36.2, 36.33, 36.1, 36.13, 36.31<br>ICD-10 Procedure Code (any position) is any of: 021L0Z5, 0270056, 0270066, 0270076, 027007Z, 02700DZ, 02700GZ, 02700T6, 02700Z6, 027034Z, 0270376, 02703FZ, 02703Z6, 027045Z, 0270466, 02704F6, 02704T6, 0271066, 0271076, 027137Z, 02713F6, 02713GZ, 02713T6, 02713TZ, 02713ZZ, 0271446, 027144Z, 027145Z, 02714G6, 02714TZ, 02714Z6, 027206Z, 02720GZ, 0272356, 027235Z, 027237Z, 02723FZ, 02723T6, 027244Z, 02724E6, 02724T6, 0273046, 0273056, 02730E6, 02730G6, 02730ZZ, 0273376, 02733DZ, 0273446, 02734D6, 02C03ZZ, 02C23Z6, 02C30Z6, 02C34Z6, 021K0Z5, 027006Z, 02700E6, 02700FZ, 02700ZZ, 0270366, 02703D6, 02703E6, 02703ZZ, 0270446, 0270476, 02704GZ, 02704Z6, 02710E6, 02710FZ, 02710T6, 02710Z6, 0271346, 02713E6, 0271456, 0271476, 027147Z, 02714DZ, 02714F6, 027204Z, 0272056, 0272066, 02720E6, 02720EZ, 02720FZ, 02720G6, 02720T6, 02720ZZ, 0272366, 02723ZZ, 0272446, 0272466, 0272476, 02724FZ, 027304Z, 0273076, 027307Z, 02730DZ, 02730T6, 02733TZ, 027344Z, 0273456, 0273466, 02734E6, 02734EZ, 02734G6, 02734TZ, 02C03Z6, 02QA3ZZ, 0270046, 0270356, 027035Z, 027036Z, 02703F6, 02703G6, 02703TZ, 027044Z, 0270456, 02704E6, 02704G6, 0271046, 027105Z, 02710G6, 02710GZ, 02710TZ, 0271356, 02713D6, 02713DZ, 02713EZ, 0271466, 02714D6, 02714E6, 02714FZ, 027207Z, 02720F6, 02720Z6, 027234Z, 0272376, 02723D6, 02723E6, 02723EZ, 02723F6, 02723G6, 02723GZ, 02723Z6, 027246Z, 027247Z, 02724G6, 02724TZ, 02724ZZ, 027305Z, 02730F6, 02730FZ, 027334Z, 027337Z, 02733EZ, 02733G6, 02733GZ, 02733ZZ, 027347Z, 02734DZ, 02C13Z6, 02C14Z6, 02C23ZZ, 02C33ZZ, 02QB3ZZ, 02QC3ZZ, 02700EZ, 02700F6, 02700G6, 02700TZ, 027037Z, 027047Z, 02704ZZ, 027106Z, 02710DZ, 02710EZ, 027135Z, 02713FZ, 02713G6, 02714EZ, 02714T6, 027205Z, 0272076, 02720D6, 027236Z, 02724D6, 02724F6, 02724GZ,

© 2023 Lin KJ et al. *JAMA Network Open*.

	0273066, 02730Z6, 0273346, 0273356, 027335Z, 0273366, 02733E6, 02733F6, 02733FZ, 02733T6, 02733Z6, 02C00Z6, 02C10Z6, 021K4Z5, 021L4Z5, 027004Z, 027005Z, 02700D6, 0270346, 02703DZ, 02703EZ, 02703GZ, 02703T6, 027046Z, 02704D6, 02704DZ, 02704EZ, 02704FZ, 02704TZ, 027104Z, 0271056, 027107Z, 02710D6, 02710F6, 02710ZZ, 027134Z, 0271366, 027136Z, 0271376, 02713Z6, 027146Z, 02714GZ, 02714ZZ, 0272046, 02720DZ, 02720TZ, 0272346, 02723DZ, 02723TZ, 0272456, 027245Z, 02724DZ, 02724EZ, 02724Z6, 027306Z, 02730D6, 02730EZ, 02730GZ, 02730TZ, 027336Z, 02733D6, 027345Z, 027346Z, 0273476, 02734F6, 02734FZ, 02734GZ, 02734T6, 02734Z6, 02734ZZ, 02C04Z6, 02C13ZZ, 02C20Z6, 02C24Z6, 02C33Z6, 02QA4ZZ, 02QB4ZZ, 02QC4ZZ<br>HCPCS Procedure Code (any position) is any of: 33510, 33511, 33517, 92921, 92924, 92938, 92941, 92973, 92995, 33516, 33518, 33519, 33521, 33534, 33572, 92944, 92984, 33513, 33535, 92982, 92996, 33140, 33141, 33512, 33514, 33523, 33545, 92920, 92937, 92943, 33522, 33530, 33533, 33536, 92925
COPD	ICD-9 Diagnosis Code (any position) is any of: 491.0, 491.9, 491.8, 492.0, 492.8, 491.1, 491.2, 496, 491.20, 491.21, 491.22<br>ICD-10 Diagnosis Code (any position) is any of: J44.9, J43.0, J41.8, J43.8, J43.9, J41.0, J42, J41.1, J43.1, J43.2, J44.0, J44.1
Diabetes	ICD-9 Diagnosis Code (any position) is any of: 249.00, 249.10, 249.11, 249.40, 249.41, 249.50, 249.51, 249.60, 249.61, 249.81, 249.91, 250.01, 250.02, 250.10, 250.11, 250.13, 250.20, 250.32, 250.42, 250.51, 250.61, 250.63, 250.70, 250.72, 250.73, 250.83, 249.01, 249.20, 249.21, 249.30, 249.31, 249.70, 249.71, 249.80, 249.90, 250.00, 250.03, 250.12, 250.21, 250.22, 250.23, 250.30, 250.31, 250.33, 250.40, 250.41, 250.43, 250.50, 250.52, 250.53, 250.60, 250.62, 250.71, 250.80, 250.81, 250.82, 250.90, 362.01, 362.02, 362.03, 362.06, 250.91, 250.92, 250.93, 357.2, 362.04, 362.05, 366.41<br>ICD-10 Diagnosis Code (any position) is any of: E08.00, E08.10, E08.11, E08.21, E08.29, E08.311, E08.319, E08.321, E08.3211, E08.3212, E08.329, E08.3291, E08.3292, E08.331, E08.339, E08.3392, E08.3393, E08.3413, E08.3419, E08.3491, E08.3493, E08.3499, E08.351, E08.3512, E08.3513, E08.3522, E08.3523, E08.3529, E08.3533, E08.3539, E08.3543, E08.3549, E08.3551, E08.3552, E08.36, E08.37X3, E08.39, E08.42, E08.52, E08.610, E08.620, E08.621, E08.622, E08.628, E08.630, E08.641, E08.8, E09.01, E09.29, E09.321, E09.3212, E09.3213, E09.3219, E09.3291, E09.3292, E09.3293, E09.331, E09.3313, E09.339, E09.3419, E09.3493, E09.3499, E09.351, E09.3511, E09.3512, E09.3513, E09.3519, E09.3522, E09.3523, E09.3532, E09.3542, E09.3552, E09.3553, E09.359, E09.3593, E09.36, E09.37X1, E09.39, E09.42, E09.43, E09.51, E09.52, E09.59, E09.610, E09.620, E09.622, E09.630, E09.649, E09.69, E09.8, E10.22, E10.29, E10.321, E10.3211, E10.3212, E10.329, E10.3291, E10.3299, E10.3311, E10.3312, E10.3319, E10.339, E10.3391, E10.3392, E10.3399, E10.3419, E10.349, E10.3491, E10.3492, E10.351, E10.3511, E10.3512, E10.3519, E10.37X1, E10.37X3, E10.37X9, E10.39, E10.40, E10.43, E10.49, E10.51, E10.52, E10.59, E10.610, E10.620, E10.622, E10.628, E08.01, E08.22, E08.3213, E08.3219, E08.3293, E08.3299, E08.3311, E08.3312, E08.3313, E08.3319, E08.3391, E08.3399, E08.341, E08.3411, E08.3412, E08.349, E08.3492, E08.3511, E08.3519, E08.3521, E08.3531, E08.3532, E08.3541, E08.3542, E08.3553, E08.3559, E08.359, E08.3591, E08.3592, E08.3593, E08.3599, E08.37X1, E08.37X2, E08.37X9, E08.40, E08.41, E08.43, E08.44, E08.49, E08.51, E08.59, E08.618, E08.638, E08.649, E08.65, E08.69, E08.9, E09.00, E09.10, E09.11, E09.21, E09.22, E09.311, E09.319, E09.3211, E09.329, E09.3299, E09.3311, E09.3312, E09.3319, E09.3391, E09.3393, E09.3399, E09.341, E09.3412, E09.3413, E09.349, E09.3491, E09.3533, E09.3551, E09.3591, E09.3592, E09.3599, E09.37X9, E09.40, E09.41, E09.44, E09.49, E09.618, E09.621, E09.628, E09.638, E09.641, E09.65, E09.9, E10.10, E10.11, E10.21, E10.311, E10.319, E10.3213, E10.3219, E10.3292, E10.3293, E10.331, E10.3313, E10.3393, E10.341, E10.3411, E10.3412, E10.3413, E10.3493, E10.3499, E10.3513, E10.359, E10.36, E10.37X2, E10.41, E10.42, E10.44, E10.618, E10.621, E10.630, E10.649, E10.69, E10.8, E10.9, E11.00, E11.10, E11.11, E11.22, E11.319, E11.321, E11.3212, E11.329, E11.3293, E11.331, E11.3312, E11.3313, E11.339, E11.3391, E11.3393, E11.3399, E11.3411, E11.3413, E11.3419, E11.349, E11.3491, E11.3492, E11.3511, E11.3512, E11.3521, E11.3522, E11.3523, E11.3529, E11.3531, E11.3532, E11.3533, E11.3551, E11.3552, E11.3553, E11.3593, E11.37X1, E11.37X2, E11.37X3, E11.37X9, E11.40, E11.43, E11.44, E11.49, E11.52, E11.59, E11.618, E11.622, E11.628, E11.630, E11.641, E11.649, E11.9, E13.00, E13.21, E13.22, E13.29, E13.311, E13.319, E13.3211, E13.3291, E13.3292, E13.331, E13.3312, E13.3319, E13.339, E13.3391, E13.3392, E13.3399, E13.3411, E13.3412, E13.3493,

© 2023 Lin KJ et al. *JAMA Network Open*.

	E13.3511, E13.3521, E13.3522, E13.3523, E13.3531, E13.3533, E13.3539, E13.3542, E13.3549, E13.3551, E13.3552, E13.3553, E13.3559, E13.36, E13.40, E13.51, E13.52, E13.59, E13.610, E13.618, E13.621, E13.622, E13.628, E13.630, E13.641, E13.649, E13.65, E10.638, E10.641, E10.65, E11.01, E11.21, E11.29, E11.311, E11.3211, E11.3213, E11.3219, E11.3291, E11.3292, E11.3299, E11.3311, E11.3319, E11.3392, E11.341, E11.3412, E11.3493, E11.3499, E11.351, E11.3513, E11.3519, E11.3539, E11.3541, E11.3542, E11.3543, E11.3549, E11.3559, E11.359, E11.3591, E11.3592, E11.3599, E11.36, E11.39, E11.41, E11.42, E11.51, E11.610, E11.620, E11.621, E11.638, E11.65, E11.69, E11.8, E13.01, E13.10, E13.11, E13.321, E13.3212, E13.3213, E13.3219, E13.329, E13.3293, E13.3299, E13.3311, E13.3313, E13.3393, E13.341, E13.3413, E13.3419, E13.349, E13.3491, E13.3492, E13.3499, E13.351, E13.3512, E13.3513, E13.3519, E13.3529, E13.3532, E13.3541, E13.3543, E13.359, E13.39, E13.41, E13.42, E13.43, E13.44, E13.49, E13.620, E13.638, E13.69, E13.8, E13.9
Falls	ICD-9 Diagnosis Code (any position) is any of: E880.9, E883.2, E883.9, E888.0, E880, E880.1, E884, E884.5, E884.9, E885.2, E886, E886.9, E881.0, E883, E884.0, E885.1, E888.1, V15.88, E881, E882, E884.1, E884.2, E884.3, E884.4, E884.6, E885, E885.9, E887, E888, E888.9, E880.0, E881.1, E883.0, E883.1, E885.0, E885.3, E885.4, E886.0, E888.8<br>ICD-10 Diagnosis Code (any position) is any of: V00.118D, V00.122D, V00.131D, V00.141D, V00.142A, V00.148D, V00.151A, V00.182A, V00.188A, V00.212D, V00.221D, V00.228A, V00.228D, V00.288A, V00.312D, V00.318A, V00.321A, V00.381D, V00.382D, V00.388D, V00.812A, V00.821A, V00.821D, V00.822D, V00.828A, V00.831A, V00.832A, V00.892D, W00.1XXA, W00.2XXA, W00.9XXD, W01.10XD, W01.110A, W01.118D, W01.119D, W01.198D, W05.2XXA, W06.XXXD, W09.0XXA, W09.2XXA, W09.8XXA, W09.8XXD, W10.0XXD, W10.1XXA, W10.2XXD, W12.XXXD, W13.1XXA, W13.2XXA, W13.3XXA, W13.8XXA, W13.9XXD, W16.021D, W16.022D, W16.222A, W16.311D, W16.312D, W16.322D, W16.331A, W16.332A, W16.42XD, W16.522D, W16.531A, W16.621A, W16.712A, W16.712D, W16.722A, W16.822A, W16.822D, W16.831D, W16.92XD, W17.1XXD, W17.3XXA, W17.81XD, W17.89XA, W18.02XA, W18.12XD, W18.40XA, W18.49XA, V00.112A, V00.112D, V00.142D, V00.152D, V00.158D, V00.211D, V00.212A, V00.218A, V00.282D, V00.311D, V00.328A, V00.822A, V00.832D, V00.898A, W00.0XXA, W00.1XXD, W08.XXXD, W09.2XXD, W10.9XXA, W13.0XXD, W16.011A, W16.021A, W16.032D, W16.121D, W16.211A, W16.212D, W16.222D, W16.42XA, W16.522A, W16.611D, W16.621D, W16.722D, W16.811A, W16.832A, W16.91XD, W17.1XXA, W17.82XD, W18.01XA, W18.09XD, W18.12XA, W18.31XA, W18.39XD, W18.42XA, W18.42XD, W18.43XD, W19.XXXA, V00.118A, V00.122A, V00.131A, V00.148A, V00.152A, V00.158A, V00.181A, V00.181D, V00.182D, V00.188D, V00.218D, V00.222A, V00.222D, V00.281D, V00.311A, V00.312A, V00.321D, V00.328D, V00.811D, V00.831D, V00.891D, W00.9XXA, W01.111A, W01.111D, W01.118A, W01.198A, W05.1XXA, W06.XXXA, W07.XXXA, W09.1XXA, W10.8XXA, W10.8XXD, W10.9XXD, W11.XXXA, W13.0XXA, W13.3XXD, W16.022A, W16.112A, W16.112D, W16.131A, W16.132A, W16.132D, W16.211D, W16.322A, W16.521A, W16.711D, W16.811D, W16.831A, W16.832D, W16.91XA, W18.00XD, W18.2XXD, W18.30XD, W18.40XD, V00.111A, V00.128D, V00.132A, V00.138A, V00.151D, V00.211A, V00.221A, V00.281A, V00.282A, V00.322A, V00.322D, V00.381A, V00.811A, V00.818A, V00.838A, V00.891A, V00.892A, W00.0XXD, W00.2XXD, W01.0XXA, W01.119A, W01.190A, W03.XXXD, W05.2XXD, W07.XXXD, W08.XXXA, W09.0XXD, W11.XXXD, W12.XXXA, W13.1XXD, W13.2XXD, W13.4XXD, W14.XXXD, W16.011D, W16.012D, W16.031A, W16.032A, W16.111A, W16.122D, W16.221A, W16.221D, W16.311A, W16.312A, W16.331D, W16.332D, W16.41XA, W16.41XD, W16.511D, W16.512A, W16.532A, W16.532D, W16.721A, W16.721D, W16.812A, W16.812D, W16.821A, W16.92XA, W17.0XXA, W17.2XXA, W17.2XXD, W17.89XD, W18.00XA, W18.01XD, W18.02XD, W18.11XD, W18.30XA, W18.31XD, W18.41XA, V00.111D, V00.121A, V00.121D, V00.128A, V00.132D, V00.138D, V00.141A, V00.288D, V00.318D, V00.382A, V00.388A, V00.812D, V00.818D, V00.828D, V00.838D, V00.898D, W01.0XXD, W01.10XA, W01.110D, W01.190D, W03.XXXA, W04.XXXA, W04.XXXD, W05.0XXA, W05.0XXD, W05.1XXD, W09.1XXD, W10.0XXA, W10.1XXD, W10.2XXA, W13.4XXA, W13.8XXD, W13.9XXA, W14.XXXA, W15.XXXA, W15.XXXD, W16.012A, W16.031D, W16.111D, W16.121A, W16.122A, W16.131D, W16.212A, W16.321A, W16.321D, W16.511A, W16.512D, W16.521D, W16.531D, W16.611A, W16.612A, W16.612D, W16.622A, W16.622D, W16.711A, W16.821D, W17.0XXD, W17.3XXD, W17.4XXA, W17.4XXD, W17.81XA, W17.82XA, W18.11XA, W18.2XXA, W18.39XA, W18.41XD, W18.43XA, W18.49XD, W19.XXXD, Z91.81
Fractures	ICD-9 Diagnosis Code (any position) is any of: 733.13, 733.15, 733.19, 800.03, 800.05, 800.22, 800.26, 800.46, 800.53, 800.55, 800.59, 800.60, 800.64, 800.66, 800.7, 800.74,

© 2023 Lin KJ et al. *JAMA Network Open*.

	800.79, 800.8, 800.80, 800.91, 800.95, 801.02, 801.04, 801.15, 801.16, 801.2, 801.21, 801.26, 801.29, 801.49, 801.51, 801.54, 801.82, 801.86, 801.93, 802, 802.21, 802.25, 802.30, 802.31, 802.39, 802.6, 803, 803.00, 803.03, 803.09, 803.10, 803.13, 803.14, 803.2, 803.26, 803.36, 803.40, 803.42, 803.45, 803.46, 803.50, 803.53, 803.60, 803.64, 803.7, 803.8, 803.83, 803.89, 804.0, 804.01, 804.09, 804.11, 804.12, 804.19, 804.26, 804.30, 804.41, 804.42, 804.45, 804.70, 804.74, 804.76, 804.81, 804.85, 804.89, 804.9, 805.01, 805.06, 805.07, 805.12, 805.16, 805.3, 805.6, 805.8, 806.0, 806.13, 806.19, 806.24, 806.25, 806.30, 806.37, 806.4, 806.69, 806.71, 806.9, 807.02, 807.06, 807.07, 807.14, 807.15, 807.16, 807.19, 807.2, 808, 808.4, 808.43, 808.44, 808.5, 808.59, 810.0, 810.02, 811.0, 811.00, 811.19, 812.30, 812.31, 812.41, 812.50, 813.0, 813.08, 813.11, 813.2, 813.83, 813.9, 815.0, 815.12, 820, 820.01, 820.03, 820.13, 820.3, 821.0, 821.10, 821.29, 821.31, 821.33, 822.0, 822.1, 823, 823.01, 823.32, 823.82, 823.91, 824.2, 824.7, 825.1, 825.34, 828.0, 828.1, 733.1, 733.16, 800.04, 800.1, 800.10, 800.11, 800.12, 800.13, 800.16, 800.21, 800.23, 800.29, 800.30, 800.31, 800.4, 800.43, 800.45, 800.50, 800.54, 800.71, 800.81, 800.84, 800.89, 800.9, 800.92, 800.94, 801.00, 801.4, 801.41, 801.43, 801.63, 801.73, 801.75, 801.76, 801.8, 801.80, 801.83, 801.84, 801.91, 802.20, 802.26, 803.0, 803.05, 803.12, 803.24, 803.3, 803.34, 803.39, 803.4, 803.49, 803.55, 803.56, 803.6, 803.65, 803.71, 803.73, 803.74, 803.91, 803.93, 803.95, 804.05, 804.10, 804.13, 804.20, 804.32, 804.34, 804.39, 804.40, 804.63, 804.65, 804.7, 804.71, 804.75, 804.79, 804.8, 804.80, 804.82, 804.83, 804.84, 804.92, 804.99, 805.02, 805.10, 805.13, 805.15, 805.17, 805.4, 805.5, 806.00, 806.03, 806.05, 806.11, 806.12, 806.22, 806.26, 806.3, 806.31, 806.34, 806.36, 806.61, 806.7, 807.3, 808.0, 808.2, 808.49, 808.9, 810.03, 811.02, 812, 812.2, 812.20, 812.21, 812.43, 813.06, 813.12, 813.20, 813.30, 813.32, 813.41, 813.50, 813.53, 813.8, 813.81, 813.91, 814.0, 814.10, 814.13, 814.18, 815, 815.03, 815.10, 815.11, 815.19, 819.1, 820.09, 820.20, 820.22, 820.31, 821.01, 821.11, 821.22, 821.32, 823.11, 823.20, 823.80, 823.90, 823.92, 824.8, 825, 825.2, 825.31, 825.32, 827, 827.0, 829.0, 733.11, 733.14, 800, 800.06, 800.09, 800.14, 800.3, 800.32, 800.42, 800.5, 800.51, 800.52, 800.56, 800.61, 800.69, 800.73, 800.82, 800.86, 800.90, 800.93, 801, 801.0, 801.06, 801.12, 801.19, 801.23, 801.24, 801.33, 801.35, 801.45, 801.55, 801.56, 801.6, 801.61, 801.62, 801.64, 801.66, 801.69, 801.74, 801.90, 801.94, 801.99, 802.1, 802.2, 802.22, 802.24, 802.36, 802.37, 802.5, 802.7, 802.8, 803.01, 803.06, 803.16, 803.25, 803.33, 803.43, 803.44, 803.5, 803.54, 803.59, 803.72, 803.75, 803.79, 803.80, 803.82, 803.85, 803.9, 803.94, 803.99, 804.02, 804.03, 804.16, 804.22, 804.23, 804.3, 804.35, 804.36, 804.44, 804.46, 804.52, 804.54, 804.61, 804.62, 804.69, 804.72, 804.90, 804.94, 805, 805.04, 805.05, 805.14, 805.18, 806.07, 806.08, 806.15, 806.16, 806.2, 806.20, 806.27, 806.28, 806.32, 806.35, 806.5, 806.72, 806.8, 807.01, 807.04, 807.11, 807.4, 807.5, 808.53, 808.8, 809.0, 810, 810.10, 810.12, 811.01, 811.03, 811.10, 811.12, 812.02, 812.09, 812.1, 812.11, 812.40, 812.5, 812.53, 813, 813.01, 813.02, 813.04, 813.05, 813.1, 813.15, 813.16, 813.43, 813.47, 814, 814.02, 814.03, 814.05, 814.06, 814.08, 814.17, 814.19, 815.00, 815.04, 816.03, 816.11, 816.12, 816.13, 817, 818.0, 820.0, 820.2, 820.32, 820.9, 821.20, 821.23, 821.3, 822, 823.00, 823.10, 823.12, 823.2, 823.21, 823.3, 823.42, 823.9, 824.0, 824.3, 825.22, 825.23, 825.24, 825.29, 825.33, 825.39, 826.0, 800.0, 800.00, 800.20, 800.33, 800.35, 800.40, 800.49, 800.70, 800.76, 800.83, 800.85, 800.96, 800.99, 801.03, 801.05, 801.1, 801.11, 801.13, 801.20, 801.3, 801.39, 801.42, 801.5, 801.50, 801.53, 801.71, 801.72, 801.79, 801.95, 802.0, 802.23, 802.28, 802.29, 802.33, 802.38, 802.9, 803.02, 803.19, 803.21, 803.22, 803.30, 803.31, 803.32, 803.41, 803.51, 803.52, 803.62, 803.63, 803.76, 803.81, 803.84, 803.86, 803.90, 803.92, 803.96, 804, 804.04, 804.06, 804.14, 804.21, 804.25, 804.29, 804.31, 804.33, 804.4, 804.43, 804.49, 804.51, 804.55, 804.56, 804.6, 804.60, 804.66, 804.73, 804.93, 804.95, 804.96, 805.00, 805.7, 805.9, 806.01, 806.02, 806.09, 806.1, 806.14, 806.18, 806.21, 806.29, 806.33, 806.38, 806.6, 806.60, 806.62, 806.70, 806.79, 807.03, 807.12, 807.6, 808.42, 809, 809.1, 810.00, 810.01, 811, 811.11, 811.13, 812.00, 812.19, 812.51, 812.54, 813.00, 813.03, 813.07, 813.13, 813.18, 813.23, 813.3, 813.4, 813.40, 813.44, 813.45, 813.52, 813.54, 813.80, 813.82, 813.92, 813.93, 814.04, 814.07, 814.1, 814.11, 814.14, 814.16, 815.01, 815.09, 815.1, 816, 816.00, 816.01, 816.02, 816.1, 816.10, 817.1, 818.1, 819.0, 820.02, 820.10, 820.12, 820.8, 821, 821.1, 821.2, 821.39, 823.02, 823.22, 823.30, 823.31, 823.41, 823.8, 824, 824.1, 824.4, 824.5, 824.6, 825.20, 825.25, 825.3, 825.30, 825.35, 829.1, 733.10, 733.12, 800.01, 800.02, 800.15, 800.19, 800.2, 800.24, 800.25, 800.34, 800.36, 800.39, 800.41, 800.44, 800.6, 800.62, 800.63, 800.65, 800.72, 800.75, 801.01, 801.09, 801.10, 801.14, 801.22, 801.25, 801.30, 801.31, 801.32, 801.34, 801.36, 801.40, 801.44, 801.46, 801.52, 801.59, 801.60, 801.65, 801.7, 801.70, 801.81, 801.85, 801.89, 801.9, 801.92, 801.96, 802.27, 802.3, 802.32, 802.34, 802.35, 802.4, 803.04, 803.1,

© 2023 Lin KJ et al. *JAMA Network Open*.

	803.11, 803.15, 803.20, 803.23, 803.29, 803.35, 803.61, 803.66, 803.69, 803.70, 804.00, 804.1, 804.15, 804.2, 804.24, 804.5, 804.50, 804.53, 804.59, 804.64, 804.86, 804.91, 805.0, 805.03, 805.08, 805.1, 805.11, 805.2, 806, 806.04, 806.06, 806.10, 806.17, 806.23, 806.39, 807, 807.0, 807.00, 807.05, 807.08, 807.09, 807.1, 807.10, 807.13, 807.17, 807.18, 808.1, 808.3, 808.41, 808.51, 808.52, 808.54, 810.1, 810.11, 810.13, 811.09, 811.1, 812.0, 812.01, 812.03, 812.10, 812.12, 812.13, 812.3, 812.4, 812.42, 812.44, 812.49, 812.52, 812.59, 813.10, 813.14, 813.17, 813.21, 813.22, 813.31, 813.33, 813.42, 813.46, 813.5, 813.51, 813.90, 814.00, 814.01, 814.09, 814.12, 814.15, 815.02, 815.13, 815.14, 816.0, 817.0, 818, 819, 820.00, 820.1, 820.11, 820.19, 820.21, 820.30, 821.00, 821.21, 821.30, 823.0, 823.1, 823.4, 823.40, 823.81, 824.9, 825.0, 825.21, 826, 826.1, 827.1, 828, 829<br>ICD-10 Diagnosis Code (any position) is any of: M48.51XA, M48.57XA, M80.011A, M80.019A, M80.051A, M80.052A, M80.079A, M80.811A, M80.839A, M80.849A, M80.852A, M80.871A, M80.872A, M80.88XA, M84.433A, M84.463A, M84.469A, M84.472A, M84.473A, M84.478A, M84.519A, M84.542A, M84.552A, M84.569A, M84.575A, M84.58XA, M84.621A, M84.631A, M84.649A, M84.652A, M84.68XA, S02.110B, S02.119A, S02.2XXB, S02.411A, S06.335A, S06.361A, S06.4X4A, S06.5X3A, S06.5X4A, S06.6X1A, S06.6X4A, S06.6X8A, S06.892A, S06.899A, S12.041A, S12.101A, S12.101B, S12.111A, S12.112A, S12.120B, S12.131A, S12.150B, S12.190A, S12.190B, S12.191A, S12.201A, S12.231B, S12.251B, S12.290B, S12.300A, S12.301A, S12.350A, S12.351B, S12.400B, S12.450A, S12.491B, S12.500B, S12.501B, S12.550B, S14.109A, S14.113A, S14.117A, S14.124A, S14.135A, S14.151A, S14.155A, S22.011A, S22.012B, S22.018B, S22.019A, S22.022B, S22.028A, S22.031A, S22.038A, S22.039A, S22.042A, S22.049A, S22.049B, S22.051A, S22.051B, S22.052A, S22.060A, S22.061A, S22.069A, S22.071A, S22.072B, S22.078A, S22.080B, S22.081B, S22.088B, S22.20XA, S22.24XB, S22.32XB, S22.43XB, S22.49XA, S22.9XXA, S22.9XXB, S24.102A, S24.104A, S24.134A, S32.002A, S32.030A, S32.031B, S32.032B, S32.040B, S32.041B, S32.052A, S32.052B, S32.112B, S32.119A, S32.122A, S32.130B, S32.15XA, S32.15XB, S32.19XA, S32.314B, S32.315A, S32.392B, S32.399A, S32.401A, S32.402A, S32.409A, S32.412A, S32.414B, S32.422B, S32.426A, S32.436A, S32.444A, S32.445A, S32.446B, S32.451B, S32.454B, S32.456A, S32.471B, S32.473B, S32.475A, S32.484A, S32.485A, S32.509B, S32.511A, S32.612B, S32.615A, S32.615B, S32.691A, S32.691B, S32.692A, S32.810B, S32.811A, S32.89XA, S32.9XXB, S34.101A, S34.111A, S34.119A, S34.122A, S34.129A, S34.131A, S34.139A, S42.015B, S42.016B, S42.018A, S42.019B, S42.031A, S42.034A, S42.102A, S42.109A, S42.111A, S42.112A, S42.114B, S42.116A, S42.121A, S42.122B, S42.133A, S42.134B, S42.142A, S42.143A, S42.152A, S42.153A, S42.155A, S42.192B, S42.199B, S42.211B, S42.212A, S42.212B, S42.214B, S42.223B, S42.225A, S42.239A, S42.242B, S42.255A, S42.261A, S42.263B, S42.266A, S42.266B, S42.292A, S42.301A, S42.309A, S42.311A, S42.321B, S42.323A, S42.324B, S42.332A, S42.335A, S42.336B, S42.342A, S42.343A, S42.351B, S42.353B, S42.366A, S42.399A, S42.401A, S42.412B, S42.415A, S42.416A, S42.423A, S42.424A, S42.425B, S42.432A, S42.443A, S42.445A, S42.447A, S42.453B, S42.456A, S42.466A, S42.466B, S42.472B, S42.474B, S42.475B, S42.489A, S42.491B, S42.492A, S42.493A, S42.493B, S42.494A, S42.495A, S42.496A, S42.496B, S42.90XA, S49.009A, S49.041A, S49.092A, S52.001A, S52.001B, S52.001C, S52.009A, S52.009C, S52.019A, S52.022A, S52.024C, S52.025C, S52.026C, S52.032B, S52.045A, S52.046B, S52.046C, S52.091B, S52.092C, S52.099C, S52.122C, S52.124C, S52.125A, S52.131A, S52.132A, S52.133B, S52.134C, S52.135C, S52.136A, S52.221C, S52.223C, S52.225B, S52.231A, S52.231C, S52.232A, S52.233C, S52.235B, S52.242A, S52.243C, S52.246B, S52.252A, S52.253A, S52.262B, S52.263B, S52.266A, S52.272A, S52.272B, S52.279B, S52.279C, S52.281A, S52.283C, S52.302C, S52.321A, S52.321C, S52.322B, S52.325A, S52.325B, S52.326A, S52.331B, S52.332A, S52.334A, S52.336A, S52.341A, S52.341B, S52.342A, S52.345A, S52.351A, S52.352C, S52.354A, S52.355C, S52.356B, S52.362A, S52.362B, S52.363A, S52.365C, S52.366C, S52.372A, S52.372B, S52.379B, S52.391A, S52.391B, S52.399A, S52.501A, S52.501C, S52.509C, S52.511C, S52.513A, S52.514A, S52.515A, S52.529A, S52.531C, S52.532A, S52.532C, S52.541A, S52.551A, S52.551C, S52.552A, S52.552B, S52.561A, S52.561B, S52.562C, S52.569C, S52.571A, S52.599C, S52.609C, S52.611C, S52.613A, S52.691B, S52.92XB, S59.029A, S59.032A, S59.042A, S59.102A, S59.111A, S59.121A, S59.191A, S59.199A, S59.201A, S59.209A, S59.219A, S59.221A, S62.013A, S62.013B, S62.021A, S62.021B, S62.024B, S62.031A, S62.031B, S62.033B, S62.035B, S62.036A, S62.109A, S62.113B, S62.114A, S62.116B, S62.136B, S62.141B, S62.153B, S62.155B, S62.161A, S62.162B, S62.164A, S62.164B, S62.166B, S62.172A, S62.174A, S62.175A, S62.183A, S62.185A, S62.186B, S62.201B, S62.202A, S62.209A, S62.212A, S62.213A, S62.221B, S62.224B, S62.225A, S62.232B, S62.236A, S62.236B,

© 2023 Lin KJ et al. *JAMA Network Open*.

	S62.242A, S62.242B, S62.244A, S62.246A, S62.251A, S62.251B, S62.252A, S62.254A, S62.255A, S62.292B, S62.303A, S62.304B, S62.305A, S62.306A, S62.307B, S62.316B, S62.321A, S62.322B, S62.323A, S62.324A, S62.325B, S62.327A, S62.329B, S62.334A, S62.334B, S62.335A, S62.335B, S62.337A, S62.337B, S62.344A, S62.344B, S62.346A, S62.347A, S62.349B, S62.350B, S62.351A, S62.351B, S62.357B, S62.358B, S62.359A, S62.359B, S62.369B, S62.394B, S62.395A, S62.397B, S62.501B, S62.511B, S62.512A, S62.516B, S62.600B, S62.601B, S62.606B, S62.607B, S62.619A, S62.625B, S62.629B, S62.632B, S62.638B, S62.641A, S62.644A, S62.644B, S62.653B, S62.655A, S62.662A, S62.666B, S62.668A, S62.669B, S62.92XA, S62.92XB, S72.001A, S72.002B, S72.019A, S72.019B, S72.022A, S72.035A, S72.036A, S72.042C, S72.043C, S72.044A, S72.045B, S72.046B, S72.059C, S72.062B, S72.062C, S72.063B, S72.063C, S72.065A, S72.092A, S72.114C, S72.121A, S72.122B, S72.124C, S72.125C, S72.126B, S72.126C, S72.131B, S72.132A, S72.133A, S72.136A, S72.136B, S72.142A, S72.142C, S72.146C, S72.22XA, S72.26XB, S72.26XC, S72.301C, S72.302A, S72.324A, S72.325B, S72.325C, S72.331B, S72.332B, S72.333A, S72.333B, S72.333C, S72.334B, S72.335C, S72.336A, S72.341B, S72.343B, S72.346B, S72.351A, S72.351B, S72.352C, S72.354A, S72.362A, S72.392B, S72.392C, S72.401C, S72.402A, S72.413C, S72.421C, S72.422B, S72.432A, S72.433B, S72.436A, S72.436C, S72.441B, S72.445B, S72.445C, S72.446C, S72.453A, S72.463B, S72.499B, S72.8X9A, S72.90XE, S72.91XE, S72.92XA, S79.011A, S79.091A, S79.099A, S79.131A, S79.139A, S82.012C, S82.013B, S82.015A, S82.016B, S82.021A, S82.021B, S82.022A, S82.023C, S82.025A, S82.026C, S82.031B, S82.032A, S82.032C, S82.033A, S82.042A, S82.042B, S82.043B, S82.045A, S82.092A, S82.092B, S82.099A, S82.099C, S82.101C, S82.102B, S82.111B, S82.112C, S82.114C, S82.115B, S82.122B, S82.124B, S82.132B, S82.133C, S82.134B, S82.135B, S82.136A, S82.141A, S82.143A, S82.145A, S82.146B, S82.161A, S82.202A, S82.209C, S82.222A, S82.223A, S82.224A, S82.232B, S82.243A, S82.245A, S82.251A, S82.254A, S82.256B, S82.256C, S82.291B, S82.292A, S82.299A, S82.391C, S82.392C, S82.421A, S82.425A, S82.431C, S82.432B, S82.434C, S82.436A, S82.436B, S82.443B, S82.444A, S82.452A, S82.452B, S82.453A, S82.454A, S82.455B, S82.456A, S82.456B, S82.461B, S82.464A, S82.464B, S82.466C, S82.499A, S82.53XA, S82.54XB, S82.54XC, S82.56XC, S82.61XA, S82.62XA, S82.65XB, S82.66XA, S82.66XC, S82.832B, S82.843A, S82.843B, S82.845B, S82.851C, S82.852A, S82.852B, S82.853A, S82.853B, S82.854B, S82.856A, S82.863B, S82.864B, S82.871A, S82.871B, S82.871C, S82.875B, S82.876A, S82.899A, S82.899C, S82.90XB, S82.91XC, S82.92XA, S82.92XB, S89.039A, S89.092A, S89.099A, S89.112A, S89.139A, S89.199A, S89.221A, S89.291A, S89.301A, S92.021A, S92.022A, S92.023A, S92.025A, S92.032B, S92.033A, S92.034B, S92.036A, S92.041B, S92.043B, S92.044A, S92.046A, S92.055A, S92.063A, S92.065B, S92.109B, S92.116A, S92.123B, S92.124A, S92.125B, S92.133B, S92.135A, S92.136A, S92.136B, S92.142A, S92.142B, S92.144A, S92.144B, S92.145A, S92.153A, S92.153B, S92.155A, S92.156A, S92.202A, S92.202B, S92.212A, S92.213A, S92.215A, S92.224A, S92.225A, S92.225B, S92.226A, S92.231B, S92.233A, S92.233B, S92.235B, S92.243B, S92.244B, S92.252B, S92.253A, S92.256A, S92.312B, S92.313B, S92.316A, S92.323A, S92.331B, S92.336A, S92.341B, S92.342A, S92.344B, S92.346A, S92.352A, S92.354A, S92.355B, S92.403A, S92.403B, S92.404B, S92.412B, S92.413A, S92.415B, S92.424B, S92.491B, S92.492B, S92.503A, S92.512A, S92.512B, S92.531A, S92.901A, S92.911B, T14.8, M48.52XA, M80.022A, M80.032A, M80.039A, M80.042A, M80.049A, M80.059A, M80.061A, M80.062A, M80.069A, M80.071A, M80.072A, M80.821A, M80.829A, M80.831A, M80.842A, M80.861A, M80.869A, M80.879A, M84.412A, M84.419A, M84.429A, M84.432A, M84.442A, M84.444A, M84.451A, M84.459A, M84.476A, M84.50XA, M84.529A, M84.551A, M84.559A, M84.561A, M84.632A, M84.634A, M84.639A, M84.642A, M84.653A, M84.671A, S02.0XXB, S02.119B, S02.19XA, S02.400A, S02.400B, S02.402A, S02.412A, S02.413A, S02.92XA, S02.92XB, S06.337A, S06.339A, S06.4X0A, S06.4X5A, S06.6X3A, S06.6X7A, S06.896A, S06.9X1A, S06.9X5A, S06.9X9A, S12.000A, S12.02XA, S12.02XB, S12.030A, S12.090B, S12.091A, S12.091B, S12.100B, S12.110B, S12.131B, S12.14XB, S12.200B, S12.201B, S12.231A, S12.24XA, S12.300B, S12.331A, S12.390B, S12.431A, S12.44XA, S12.451A, S12.490A, S12.490B, S12.491A, S12.531A, S12.601A, S12.630A, S12.64XA, S12.64XB, S12.651B, S14.104A, S14.107A, S14.114A, S14.133A, S14.152A, S22.001A, S22.002A, S22.008A, S22.012A, S22.021B, S22.030A, S22.031B, S22.032A, S22.032B, S22.039B, S22.048A, S22.060B, S22.062B, S22.068A, S22.069B, S22.082B, S22.089A, S22.20XB, S22.21XB, S22.39XB, S24.112A, S24.154A, S32.000A, S32.001B, S32.022A, S32.028A, S32.049A, S32.050B, S32.059B, S32.10XB, S32.110A, S32.111B, S32.119B, S32.132A, S32.14XB, S32.17XB, S32.2XXB, S32.301A, S32.301B, S32.311A, S32.312B, S32.313A, S32.391A, S32.391B, S32.409B, S32.412B, S32.413A, S32.415B, S32.416A, S32.421A, S32.421B, S32.432A,

© 2023 Lin KJ et al. *JAMA Network Open*.

	S32.433B, S32.434B, S32.435B, S32.436B, S32.441A, S32.441B, S32.444B, S32.445B, S32.452B, S32.455A, S32.455B, S32.464B, S32.465B, S32.472A, S32.472B, S32.473A, S32.476B, S32.482B, S32.483A, S32.485B, S32.486A, S32.491B, S32.492A, S32.511B, S32.512A, S32.591A, S32.599A, S32.599B, S32.612A, S32.810A, S32.811B, S32.82XB, S32.89XB, S34.104A, S34.109A, S34.125A, S42.001A, S42.002A, S42.002B, S42.009A, S42.015A, S42.021A, S42.021B, S42.022A, S42.023A, S42.024A, S42.033A, S42.101A, S42.109B, S42.112B, S42.115B, S42.134A, S42.136A, S42.141B, S42.151A, S42.191A, S42.191B, S42.201B, S42.202A, S42.202B, S42.213B, S42.214A, S42.216A, S42.222A, S42.223A, S42.224A, S42.226B, S42.239B, S42.251A, S42.251B, S42.254B, S42.256A, S42.262A, S42.264B, S42.291B, S42.293B, S42.296A, S42.309B, S42.325A, S42.325B, S42.326B, S42.343B, S42.346B, S42.354A, S42.355B, S42.362A, S42.364A, S42.364B, S42.366B, S42.391A, S42.392A, S42.411A, S42.412A, S42.413B, S42.414A, S42.414B, S42.415B, S42.426A, S42.431B, S42.434A, S42.435B, S42.442A, S42.448B, S42.449A, S42.452A, S42.453A, S42.455A, S42.463B, S42.464A, S42.475A, S42.481A, S49.021A, S49.102A, S49.109A, S49.111A, S49.112A, S49.131A, S52.002C, S52.022B, S52.022C, S52.025B, S52.026B, S52.032A, S52.033B, S52.035B, S52.036A, S52.042C, S52.045C, S52.102B, S52.112A, S52.119A, S52.121A, S52.123B, S52.125B, S52.131B, S52.131C, S52.134A, S52.136C, S52.181B, S52.182A, S52.189A, S52.201C, S52.209C, S52.211A, S52.225C, S52.235C, S52.236A, S52.241C, S52.242B, S52.245A, S52.246C, S52.251C, S52.252B, S52.254A, S52.255C, S52.262C, S52.265A, S52.265C, S52.271A, S52.281C, S52.282A, S52.282B, S52.292C, S52.299B, S52.299C, S52.301A, S52.301B, S52.301C, S52.309A, S52.312A, S52.319A, S52.325C, S52.326C, S52.332C, S52.333A, S52.335B, S52.335C, S52.336C, S52.344C, S52.345C, S52.354B, S52.354C, S52.355A, S52.361A, S52.363B, S52.365A, S52.382A, S52.382C, S52.389B, S52.391C, S52.392A, S52.392B, S52.399C, S52.509A, S52.512A, S52.513C, S52.514B, S52.515C, S52.516C, S52.532B, S52.539B, S52.541C, S52.561C, S52.569A, S52.569B, S52.571C, S52.572C, S52.579B, S52.591B, S52.592C, S52.609A, S52.611B, S52.613C, S52.614B, S52.616A, S52.621A, S52.622A, S52.629A, S52.692A, S52.692C, S52.91XA, S52.92XA, S59.002A, S59.011A, S59.021A, S59.022A, S59.091A, S59.122A, S59.131A, S59.231A, S62.009B, S62.011B, S62.012A, S62.012B, S62.015A, S62.016B, S62.022B, S62.023B, S62.032A, S62.034A, S62.109B, S62.111A, S62.115A, S62.123B, S62.124A, S62.125B, S62.126B, S62.132B, S62.144A, S62.145B, S62.151B, S62.152B, S62.154B, S62.155A, S62.181B, S62.182B, S62.186A, S62.202B, S62.223B, S62.224A, S62.231B, S62.234B, S62.235A, S62.235B, S62.243A, S62.243B, S62.254B, S62.292A, S62.299A, S62.299B, S62.300B, S62.301B, S62.305B, S62.307A, S62.311A, S62.314A, S62.315A, S62.320A, S62.320B, S62.324B, S62.326B, S62.327B, S62.328A, S62.329A, S62.333A, S62.338A, S62.341A, S62.345B, S62.349A, S62.352B, S62.353A, S62.353B, S62.354A, S62.355A, S62.360A, S62.361A, S62.362A, S62.363A, S62.364A, S62.368B, S62.369A, S62.391A, S62.394A, S62.395B, S62.396B, S62.399B, S62.501A, S62.502B, S62.509A, S62.521A, S62.523A, S62.523B, S62.524A, S62.600A, S62.602A, S62.604A, S62.604B, S62.609B, S62.613A, S62.613B, S62.614A, S62.618A, S62.618B, S62.622B, S62.632A, S62.636A, S62.636B, S62.639B, S62.643A, S62.645A, S62.646A, S62.647B, S62.648B, S62.650A, S62.651A, S62.654B, S62.658B, S62.661A, S62.661B, S62.663A, S62.664A, S62.665B, S62.90XA, S72.001C, S72.021C, S72.022B, S72.025A, S72.025C, S72.031B, S72.031C, S72.032A, S72.033C, S72.034B, S72.041C, S72.042A, S72.043A, S72.044B, S72.044C, S72.052B, S72.061B, S72.063A, S72.064C, S72.066B, S72.109B, S72.109C, S72.112B, S72.113B, S72.114B, S72.121C, S72.122A, S72.124A, S72.124B, S72.126A, S72.132B, S72.134A, S72.135A, S72.141A, S72.143A, S72.144A, S72.145A, S72.145B, S72.146A, S72.22XC, S72.24XC, S72.25XA, S72.302C, S72.309C, S72.321B, S72.325A, S72.331A, S72.332A, S72.332C, S72.341C, S72.342A, S72.342B, S72.343C, S72.352B, S72.355C, S72.356A, S72.356C, S72.363A, S72.364A, S72.364B, S72.364C, S72.365B, S72.366A, S72.392A, S72.399A, S72.409C, S72.412C, S72.413A, S72.414B, S72.421A, S72.422A, S72.426B, S72.426C, S72.431A, S72.434A, S72.434B, S72.434C, S72.441A, S72.446B, S72.451A, S72.452C, S72.453B, S72.453C, S72.454C, S72.455B, S72.456A, S72.462A, S72.463C, S72.464B, S72.465A, S72.465C, S72.466A, S72.466B, S72.466C, S72.492B, S72.8X2B, S72.8X9C, S72.90XC, S72.91XB, S72.92XE, S79.009A, S79.092A, S79.119A, S79.132A, S79.142A, S79.199A, S82.009A, S82.011B, S82.012B, S82.014A, S82.021C, S82.022B, S82.022C, S82.026B, S82.035C, S82.036B, S82.045B, S82.046A, S82.046B, S82.091B, S82.092C, S82.099B, S82.101A, S82.109A, S82.112B, S82.113A, S82.114A, S82.115C, S82.121C, S82.123A, S82.123C, S82.125A, S82.125B, S82.126B, S82.126C, S82.131B, S82.136B, S82.144C, S82.146A, S82.152C, S82.154A, S82.191B, S82.199A, S82.199B, S82.201C, S82.209A, S82.225A, S82.226C, S82.241A, S82.241B, S82.242B, S82.252B, S82.252C, S82.253A, S82.254B, S82.254C, S82.255A, S82.255C, S82.262A, S82.264A, S82.264C,

© 2023 Lin KJ et al. *JAMA Network Open*.

	S82.266B, S82.292B, S82.292C, S82.302B, S82.311A, S82.391A, S82.422B, S82.422C, S82.423A, S82.423C, S82.424C, S82.425C, S82.432A, S82.442C, S82.443C, S82.451A, S82.451B, S82.452C, S82.453B, S82.462A, S82.462B, S82.462C, S82.463C, S82.465C, S82.491A, S82.491B, S82.499C, S82.53XC, S82.55XC, S82.64XB, S82.64XC, S82.839B, S82.841C, S82.844A, S82.852C, S82.855A, S82.855C, S82.856B, S82.861C, S82.862A, S82.863A, S82.863C, S82.866A, S82.866B, S82.874C, S82.875A, S82.876B, S82.892A, S82.899B, S82.91XA, S82.91XB, S89.009A, S89.019A, S89.021A, S89.111A, S89.119A, S89.192A, S89.212A, S89.222A, S89.229A, S89.312A, S89.322A, S89.329A, S89.391A, S89.399A, S92.009A, S92.011A, S92.012A, S92.012B, S92.013A, S92.014A, S92.014B, S92.015A, S92.023B, S92.024B, S92.031A, S92.033B, S92.035A, S92.041A, S92.045A, S92.052A, S92.052B, S92.053B, S92.054A, S92.056A, S92.065A, S92.066B, S92.101A, S92.111A, S92.113B, S92.122B, S92.123A, S92.124B, S92.132B, S92.134A, S92.134B, S92.135B, S92.141A, S92.143A, S92.143B, S92.152B, S92.191A, S92.191B, S92.192A, S92.192B, S92.199A, S92.199B, S92.215B, S92.216B, S92.226B, S92.244A, S92.255A, S92.302B, S92.315B, S92.322A, S92.332A, S92.332B, S92.344A, S92.351B, S92.356A, S92.356B, S92.412A, S92.421A, S92.425A, S92.426B, S92.506B, S92.511A, S92.513A, S92.513B, S92.515B, S92.516B, S92.522A, S92.523A, S92.524B, S92.531B, S92.533B, S92.902B, S92.912A, S92.919A, M48.54XA, M48.56XA, M80.00XA, M80.819A, M80.832A, M80.862A, M84.411A, M84.434A, M84.443A, M84.452A, M84.475A, M84.511A, M84.532A, M84.533A, M84.534A, M84.539A, M84.541A, M84.550A, M84.60XA, M84.612A, M84.629A, M84.641A, M84.659A, M84.662A, M84.663A, M84.669A, M84.674A, S02.110A, S02.112B, S02.401A, S02.401B, S02.411B, S02.412B, S02.413B, S02.42XA, S02.609A, S02.66XA, S02.66XB, S02.69XA, S06.330A, S06.331A, S06.362A, S06.363A, S06.367A, S06.4X2A, S06.4X9A, S06.6X2A, S06.6X9A, S06.894A, S06.898A, S06.9X2A, S06.9X8A, S12.01XA, S12.040B, S12.041B, S12.090A, S12.110A, S12.111B, S12.120A, S12.121A, S12.14XA, S12.150A, S12.151A, S12.191B, S12.250B, S12.301B, S12.34XB, S12.391A, S12.401A, S12.401B, S12.44XB, S12.500A, S12.501A, S12.54XA, S12.550A, S12.600A, S12.600B, S12.631B, S12.650A, S12.651A, S12.690B, S12.9XXA, S14.105A, S14.106A, S14.112A, S14.121A, S14.122A, S14.131A, S14.137A, S14.153A, S14.157A, S22.000A, S22.009A, S22.009B, S22.011B, S22.020A, S22.020B, S22.021A, S22.029B, S22.030B, S22.038B, S22.041A, S22.042B, S22.048B, S22.050A, S22.052B, S22.058A, S22.058B, S22.059A, S22.062A, S22.068B, S22.070A, S22.070B, S22.079B, S22.21XA, S22.23XA, S22.32XA, S22.41XA, S22.42XA, S24.109A, S24.113A, S24.153A, S32.000B, S32.001A, S32.002B, S32.008A, S32.008B, S32.009A, S32.011A, S32.018A, S32.028B, S32.030B, S32.032A, S32.039A, S32.042B, S32.051A, S32.051B, S32.058A, S32.058B, S32.10XA, S32.110B, S32.120B, S32.121B, S32.130A, S32.16XB, S32.19XB, S32.309B, S32.316B, S32.399B, S32.411B, S32.413B, S32.414A, S32.415A, S32.423A, S32.435A, S32.443A, S32.451A, S32.461A, S32.462A, S32.463A, S32.465A, S32.466B, S32.471A, S32.474B, S32.476A, S32.481A, S32.482A, S32.486B, S32.501A, S32.502A, S32.601A, S32.609A, S32.614A, S32.699A, S32.699B, S34.105A, S34.113A, S34.114A, S34.115A, S42.009B, S42.011A, S42.011B, S42.012B, S42.013B, S42.017B, S42.019A, S42.022B, S42.023B, S42.025B, S42.026B, S42.035A, S42.036A, S42.036B, S42.113A, S42.114A, S42.116B, S42.123B, S42.124A, S42.124B, S42.125A, S42.126A, S42.131A, S42.132A, S42.132B, S42.144B, S42.146A, S42.151B, S42.152B, S42.154B, S42.156A, S42.192A, S42.201A, S42.209A, S42.215A, S42.221B, S42.241B, S42.249A, S42.253B, S42.255B, S42.262B, S42.263A, S42.264A, S42.265A, S42.265B, S42.294A, S42.321A, S42.322A, S42.323B, S42.324A, S42.331A, S42.331B, S42.334A, S42.341B, S42.342B, S42.344A, S42.345B, S42.355A, S42.356B, S42.361B, S42.365A, S42.409B, S42.413A, S42.421B, S42.422B, S42.423B, S42.425A, S42.431A, S42.435A, S42.441A, S42.443B, S42.446B, S42.448A, S42.452B, S42.454A, S42.464B, S42.465A, S42.472A, S42.473B, S42.482A, S42.494B, S49.029A, S49.039A, S49.049A, S49.119A, S49.121A, S49.122A, S49.129A, S49.139A, S49.141A, S49.142A, S49.192A, S52.002B, S52.011A, S52.021B, S52.021C, S52.023A, S52.024A, S52.025A, S52.026A, S52.033A, S52.033C, S52.034C, S52.043A, S52.043B, S52.043C, S52.044C, S52.045B, S52.091A, S52.091C, S52.092A, S52.099A, S52.102A, S52.111A, S52.121C, S52.122A, S52.123A, S52.125C, S52.126B, S52.126C, S52.133A, S52.133C, S52.134B, S52.136B, S52.189C, S52.201A, S52.212A, S52.222B, S52.223A, S52.224A, S52.224C, S52.225A, S52.226A, S52.226B, S52.231B, S52.232C, S52.234B, S52.234C, S52.236B, S52.241A, S52.242C, S52.244B, S52.245C, S52.246A, S52.253C, S52.255A, S52.256B, S52.256C, S52.261A, S52.261C, S52.265B, S52.266C, S52.271B, S52.271C, S52.272C, S52.292B, S52.324A, S52.333C, S52.343C, S52.344B, S52.346B, S52.351B, S52.351C, S52.352A, S52.352B, S52.353A, S52.356A, S52.361B, S52.364A, S52.365B, S52.366A, S52.371C, S52.379C, S52.381A, S52.389C, S52.501B, S52.512C, S52.514C, S52.521A, S52.531A, S52.542A, S52.542B, S52.552C,

© 2023 Lin KJ et al. *JAMA Network Open*.

	S52.562A, S52.572A, S52.572B, S52.592B, S52.601C, S52.602A, S52.609B, S52.612A, S52.612C, S52.615C, S52.616B, S52.691A, S52.699C, S52.90XA, S52.90XB, S52.90XC, S52.92XC, S59.001A, S59.039A, S59.049A, S59.099A, S59.109A, S59.132A, S59.142A, S59.212A, S59.249A, S62.001B, S62.011A, S62.014A, S62.015B, S62.016A, S62.026B, S62.101A, S62.101B, S62.102A, S62.123A, S62.124B, S62.131A, S62.132A, S62.133A, S62.133B, S62.144B, S62.146B, S62.151A, S62.153A, S62.156A, S62.161B, S62.162A, S62.163A, S62.165B, S62.171B, S62.175B, S62.181A, S62.184A, S62.211B, S62.213B, S62.233A, S62.233B, S62.241A, S62.241B, S62.252B, S62.291A, S62.291B, S62.300A, S62.301A, S62.302A, S62.309B, S62.312B, S62.314B, S62.317A, S62.318B, S62.319A, S62.326A, S62.328B, S62.331A, S62.331B, S62.332B, S62.333B, S62.339B, S62.340A, S62.342A, S62.343A, S62.343B, S62.345A, S62.352A, S62.360B, S62.363B, S62.366A, S62.366B, S62.367B, S62.390A, S62.393B, S62.502A, S62.509B, S62.514A, S62.514B, S62.515B, S62.524B, S62.525B, S62.526A, S62.603B, S62.612A, S62.615B, S62.616A, S62.623A, S62.623B, S62.624B, S62.625A, S62.626A, S62.627A, S62.628A, S62.629A, S62.634A, S62.634B, S62.635A, S62.637B, S62.640B, S62.642A, S62.646B, S62.648A, S62.649A, S62.649B, S62.652B, S62.653A, S62.655B, S62.656B, S62.658A, S62.659A, S62.660B, S62.667A, S62.91XB, S72.001B, S72.002C, S72.011B, S72.012B, S72.022C, S72.024A, S72.024B, S72.025B, S72.032B, S72.033A, S72.035C, S72.036C, S72.043B, S72.045A, S72.046A, S72.061C, S72.066A, S72.091A, S72.092B, S72.111A, S72.112A, S72.112C, S72.114A, S72.115A, S72.116B, S72.121B, S72.123B, S72.134C, S72.135B, S72.135C, S72.141C, S72.146B, S72.301B, S72.302B, S72.321A, S72.322B, S72.322C, S72.335A, S72.336C, S72.343A, S72.344C, S72.345B, S72.345C, S72.346C, S72.353B, S72.354B, S72.354C, S72.355B, S72.356B, S72.362B, S72.363B, S72.363C, S72.391B, S72.402B, S72.411A, S72.411B, S72.411C, S72.412A, S72.414C, S72.415A, S72.415C, S72.416B, S72.422C, S72.423A, S72.423B, S72.425B, S72.431B, S72.432B, S72.432C, S72.435B, S72.435C, S72.442C, S72.444A, S72.444B, S72.445A, S72.446A, S72.455A, S72.455C, S72.461B, S72.491A, S72.491C, S72.492A, S72.8X1A, S72.8X1C, S72.8X2A, S72.8X9B, S72.90XA, S72.91XA, S72.92XC, S79.111A, S79.122A, S79.141A, S79.191A, S79.192A, S82.002C, S82.009B, S82.012A, S82.016C, S82.023A, S82.023B, S82.025B, S82.032B, S82.034C, S82.044B, S82.101B, S82.102C, S82.109B, S82.109C, S82.114B, S82.115A, S82.121A, S82.121B, S82.124A, S82.124C, S82.132C, S82.133A, S82.134A, S82.143B, S82.144A, S82.152A, S82.152B, S82.153B, S82.154C, S82.155C, S82.191C, S82.192B, S82.201A, S82.201B, S82.221B, S82.222B, S82.223C, S82.226B, S82.231B, S82.232C, S82.234A, S82.236C, S82.241C, S82.242C, S82.243C, S82.246A, S82.246B, S82.251C, S82.255B, S82.261C, S82.264B, S82.299B, S82.301A, S82.302A, S82.309A, S82.309B, S82.309C, S82.312A, S82.319A, S82.401A, S82.421B, S82.423B, S82.426A, S82.433A, S82.433C, S82.434B, S82.435B, S82.436C, S82.441C, S82.455A, S82.456C, S82.461C, S82.463B, S82.465A, S82.465B, S82.492B, S82.492C, S82.51XA, S82.52XA, S82.53XB, S82.55XA, S82.55XB, S82.56XA, S82.56XB, S82.61XB, S82.63XC, S82.819A, S82.829A, S82.839C, S82.841B, S82.842B, S82.844B, S82.853C, S82.856C, S82.862B, S82.864C, S82.866C, S82.873B, S82.891A, S82.891B, S82.891C, S82.892B, S82.90XC, S82.92XC, S89.002A, S89.012A, S89.032A, S89.041A, S89.049A, S89.102A, S89.109A, S89.122A, S89.149A, S89.201A, S89.209A, S89.219A, S89.309A, S89.392A, S92.002B, S92.009B, S92.015B, S92.016B, S92.031B, S92.035B, S92.036B, S92.042A, S92.043A, S92.044B, S92.045B, S92.056B, S92.061A, S92.102B, S92.109A, S92.111B, S92.114B, S92.116B, S92.121B, S92.146A, S92.151B, S92.155B, S92.201A, S92.211A, S92.211B, S92.216A, S92.222A, S92.222B, S92.223B, S92.241B, S92.246B, S92.253B, S92.254A, S92.254B, S92.255B, S92.301B, S92.302A, S92.312A, S92.314A, S92.321A, S92.322B, S92.323B, S92.324A, S92.324B, S92.325B, S92.333A, S92.336B, S92.342B, S92.343B, S92.345A, S92.345B, S92.346B, S92.353A, S92.355A, S92.401A, S92.402B, S92.405B, S92.414A, S92.414B, S92.415A, S92.416B, S92.422A, S92.504A, S92.505A, S92.514B, S92.515A, S92.521B, S92.523B, S92.525B, S92.526A, S92.534B, S92.536A, S92.592A, S92.599A, S92.909A, S92.909B, S92.912B, M48.55XA, M48.58XA, M80.021A, M80.031A, M80.041A, M80.08XA, M80.812A, M80.851A, M84.40XA, M84.422A, M84.431A, M84.439A, M84.441A, M84.445A, M84.446A, M84.453A, M84.454A, M84.461A, M84.462A, M84.464A, M84.471A, M84.479A, M84.48XA, M84.521A, M84.531A, M84.553A, M84.563A, M84.564A, M84.573A, M84.619A, M84.633A, M84.651A, M84.661A, M84.673A, M84.675A, M84.676A, S02.0XXA, S02.111A, S02.111B, S02.112A, S02.113B, S02.19XB, S02.402B, S02.600B, S06.332A, S06.336A, S06.338A, S06.4X3A, S06.4X6A, S06.4X7A, S06.5X1A, S06.5X6A, S06.5X8A, S06.6X0A, S06.6X5A, S06.6X6A, S06.890A, S06.893A, S06.9X3A, S06.9X6A, S12.000B, S12.001A, S12.031B, S12.112B, S12.121B, S12.130A, S12.151B, S12.230A, S12.230B, S12.24XB, S12.250A, S12.331B, S12.34XA, S12.350B, S12.390A, S12.391B, S12.450B, S12.451B, S12.54XB, S12.551A,

© 2023 Lin KJ et al. *JAMA Network Open*.

	S12.551B, S12.590A, S12.590B, S12.601B, S12.631A, S12.650B, S12.691B, S12.8XXA, S14.116A, S14.123A, S14.125A, S14.126A, S14.134A, S14.154A, S22.001B, S22.010A, S22.010B, S22.022A, S22.040B, S22.041B, S22.050B, S22.059B, S22.061B, S22.071B, S22.072A, S22.078B, S22.081A, S22.22XA, S22.23XB, S22.24XA, S22.39XA, S22.41XB, S22.43XA, S22.49XB, S24.101A, S24.111A, S24.131A, S32.009B, S32.010A, S32.012A, S32.018B, S32.019B, S32.021A, S32.029A, S32.029B, S32.038A, S32.042A, S32.048A, S32.048B, S32.050A, S32.112A, S32.120A, S32.121A, S32.122B, S32.129B, S32.131A, S32.16XA, S32.302A, S32.302B, S32.392A, S32.401B, S32.402B, S32.416B, S32.422A, S32.424A, S32.425A, S32.426B, S32.431A, S32.431B, S32.433A, S32.442B, S32.443B, S32.446A, S32.453B, S32.454A, S32.456B, S32.464A, S32.474A, S32.483B, S32.484B, S32.499B, S32.501B, S32.502B, S32.509A, S32.519A, S32.602B, S32.613B, S32.614B, S32.616A, S32.82XA, S34.112A, S34.123A, S42.013A, S42.014A, S42.016A, S42.024B, S42.025A, S42.026A, S42.032B, S42.033B, S42.034B, S42.035B, S42.115A, S42.121B, S42.122A, S42.123A, S42.125B, S42.126B, S42.135A, S42.135B, S42.141A, S42.143B, S42.145B, S42.146B, S42.153B, S42.213A, S42.222B, S42.231A, S42.231B, S42.232B, S42.242A, S42.271A, S42.291A, S42.293A, S42.294B, S42.295A, S42.295B, S42.301B, S42.312A, S42.326A, S42.333B, S42.334B, S42.335B, S42.336A, S42.341A, S42.345A, S42.346A, S42.351A, S42.352A, S42.352B, S42.353A, S42.354B, S42.356A, S42.362B, S42.365B, S42.399B, S42.401B, S42.411B, S42.416B, S42.433A, S42.436A, S42.436B, S42.441B, S42.442B, S42.444A, S42.444B, S42.449B, S42.451A, S42.454B, S42.456B, S42.462A, S42.463A, S42.474A, S42.476B, S42.491A, S42.492B, S42.495B, S42.91XA, S42.92XA, S42.92XB, S49.002A, S49.019A, S49.032A, S49.091A, S49.101A, S49.132A, S52.002A, S52.009B, S52.024B, S52.031B, S52.031C, S52.035A, S52.036C, S52.041A, S52.041C, S52.042A, S52.042B, S52.044B, S52.046A, S52.101C, S52.109A, S52.121B, S52.122B, S52.123C, S52.124A, S52.126A, S52.132B, S52.132C, S52.135A, S52.135B, S52.181A, S52.181C, S52.182B, S52.202A, S52.209B, S52.221A, S52.221B, S52.222C, S52.223B, S52.232B, S52.233B, S52.235A, S52.241B, S52.243A, S52.243B, S52.251B, S52.253B, S52.254B, S52.255B, S52.256A, S52.262A, S52.263A, S52.264A, S52.264C, S52.266B, S52.282C, S52.283A, S52.283B, S52.291C, S52.299A, S52.302A, S52.309B, S52.311A, S52.323B, S52.324C, S52.334B, S52.336B, S52.341C, S52.344A, S52.346A, S52.346C, S52.353B, S52.353C, S52.355B, S52.363C, S52.371A, S52.371B, S52.381B, S52.381C, S52.382B, S52.389A, S52.399B, S52.502B, S52.502C, S52.511A, S52.511B, S52.512B, S52.515B, S52.516A, S52.516B, S52.522A, S52.531B, S52.541B, S52.542C, S52.559C, S52.562B, S52.591A, S52.599A, S52.601A, S52.602B, S52.611A, S52.615A, S52.615B, S52.691C, S52.692B, S52.699B, S52.91XB, S59.019A, S59.031A, S59.092A, S59.119A, S59.139A, S59.141A, S59.192A, S59.202A, S59.222A, S59.232A, S59.241A, S59.242A, S59.292A, S62.002A, S62.002B, S62.022A, S62.024A, S62.025A, S62.033A, S62.034B, S62.111B, S62.112A, S62.114B, S62.116A, S62.125A, S62.134A, S62.135A, S62.141A, S62.142B, S62.143A, S62.143B, S62.145A, S62.146A, S62.165A, S62.166A, S62.171A, S62.172B, S62.173A, S62.174B, S62.176A, S62.176B, S62.184B, S62.201A, S62.211A, S62.212B, S62.221A, S62.222A, S62.223A, S62.226A, S62.226B, S62.234A, S62.245A, S62.245B, S62.253A, S62.253B, S62.256B, S62.309A, S62.310A, S62.310B, S62.312A, S62.313A, S62.313B, S62.315B, S62.316A, S62.318A, S62.319B, S62.322A, S62.323B, S62.330A, S62.330B, S62.338B, S62.339A, S62.341B, S62.350A, S62.354B, S62.358A, S62.364B, S62.365A, S62.368A, S62.390B, S62.392A, S62.392B, S62.393A, S62.396A, S62.397A, S62.398B, S62.513A, S62.516A, S62.522B, S62.525A, S62.526B, S62.606A, S62.608A, S62.610A, S62.611A, S62.616B, S62.617A, S62.617B, S62.619B, S62.621B, S62.622A, S62.626B, S62.627B, S62.630A, S62.631A, S62.633A, S62.633B, S62.635B, S62.638A, S62.641B, S62.650B, S62.651B, S62.654A, S62.656A, S62.657B, S62.663B, S62.664B, S62.666A, S62.668B, S62.90XB, S72.002A, S72.009C, S72.011A, S72.011C, S72.021A, S72.023C, S72.033B, S72.034A, S72.041A, S72.041B, S72.045C, S72.046C, S72.051A, S72.051B, S72.052A, S72.052C, S72.059A, S72.062A, S72.064B, S72.066C, S72.091C, S72.092C, S72.099A, S72.099B, S72.101C, S72.102B, S72.111B, S72.111C, S72.113C, S72.115C, S72.123A, S72.123C, S72.125B, S72.131A, S72.133B, S72.134B, S72.141B, S72.144B, S72.21XA, S72.21XB, S72.21XC, S72.22XB, S72.23XA, S72.23XC, S72.24XA, S72.301A, S72.321C, S72.322A, S72.323A, S72.323C, S72.331C, S72.334A, S72.334C, S72.335B, S72.342C, S72.344A, S72.344B, S72.345A, S72.351C, S72.352A, S72.361A, S72.361C, S72.362C, S72.366B, S72.366C, S72.391A, S72.399B, S72.401B, S72.402C, S72.409B, S72.413B, S72.416A, S72.416C, S72.421B, S72.425A, S72.425C, S72.426A, S72.433A, S72.433C, S72.436B, S72.441C, S72.442B, S72.443A, S72.461A, S72.462C, S72.463A, S72.464C, S72.465B, S72.472A, S72.479A, S72.492C, S72.8X2C, S72.90XB, S79.002A, S79.102A, S79.109A, S79.129A, S79.149A, S82.001B, S82.002A, S82.011A, S82.011C, S82.013C, S82.016A, S82.024B, S82.024C, S82.031A,

© 2023 Lin KJ et al. *JAMA Network Open*.

	S82.031C, S82.033B, S82.033C, S82.034B, S82.035A, S82.036C, S82.041A, S82.041C, S82.042C, S82.043A, S82.044A, S82.044C, S82.046C, S82.091A, S82.091C, S82.113B, S82.113C, S82.116A, S82.131A, S82.132A, S82.134C, S82.135A, S82.142B, S82.142C, S82.144B, S82.146C, S82.153A, S82.153C, S82.154B, S82.155B, S82.156A, S82.156C, S82.169A, S82.192A, S82.199C, S82.202B, S82.209B, S82.221A, S82.223B, S82.224B, S82.225B, S82.225C, S82.231A, S82.233C, S82.234B, S82.235A, S82.235B, S82.235C, S82.236A, S82.236B, S82.242A, S82.243B, S82.244A, S82.244B, S82.245B, S82.245C, S82.246C, S82.251B, S82.252A, S82.253C, S82.261A, S82.261B, S82.263B, S82.265A, S82.265B, S82.265C, S82.291C, S82.299C, S82.301B, S82.301C, S82.392B, S82.399B, S82.401B, S82.402B, S82.409B, S82.409C, S82.421C, S82.422A, S82.424A, S82.426C, S82.432C, S82.442A, S82.445B, S82.446A, S82.446B, S82.451C, S82.453C, S82.454B, S82.454C, S82.455C, S82.461A, S82.464C, S82.466B, S82.492A, S82.51XB, S82.51XC, S82.63XA, S82.63XB, S82.65XA, S82.65XC, S82.66XB, S82.821A, S82.831A, S82.831C, S82.832A, S82.832C, S82.841A, S82.842A, S82.843C, S82.844C, S82.845A, S82.845C, S82.846C, S82.851A, S82.851B, S82.855B, S82.864A, S82.865A, S82.872C, S82.874B, S89.011A, S89.042A, S89.101A, S89.142A, S89.202A, S89.311A, S89.321A, S92.001A, S92.011B, S92.013B, S92.016A, S92.022B, S92.025B, S92.051B, S92.053A, S92.054B, S92.055B, S92.061B, S92.062A, S92.064A, S92.064B, S92.101B, S92.102A, S92.113A, S92.115A, S92.115B, S92.125A, S92.126B, S92.131A, S92.131B, S92.132A, S92.133A, S92.141B, S92.145B, S92.152A, S92.154A, S92.154B, S92.156B, S92.201B, S92.212B, S92.214B, S92.221A, S92.223A, S92.232B, S92.234B, S92.235A, S92.242A, S92.242B, S92.245B, S92.246A, S92.251B, S92.256B, S92.311B, S92.313A, S92.314B, S92.321B, S92.326A, S92.331A, S92.335B, S92.341A, S92.343A, S92.353B, S92.401B, S92.402A, S92.404A, S92.405A, S92.406B, S92.411A, S92.413B, S92.422B, S92.423A, S92.424A, S92.425B, S92.491A, S92.492A, S92.499A, S92.501A, S92.501B, S92.502A, S92.504B, S92.506A, S92.514A, S92.516A, S92.521A, S92.524A, S92.532A, S92.533A, S92.591A, S92.592B, S92.599B, S92.901B, S92.902A, S92.919B, M48.53XA, M80.012A, M80.029A, M80.80XA, M80.822A, M80.841A, M80.859A, M84.421A, M84.474A, M84.477A, M84.512A, M84.522A, M84.549A, M84.562A, M84.571A, M84.572A, M84.574A, M84.576A, M84.611A, M84.622A, M84.650A, M84.664A, M84.672A, S02.113A, S02.118A, S02.118B, S02.2XXA, S02.42XB, S02.600A, S02.609B, S02.69XB, S02.91XA, S02.91XB, S06.333A, S06.334A, S06.360A, S06.364A, S06.365A, S06.366A, S06.368A, S06.369A, S06.4X1A, S06.4X8A, S06.5X0A, S06.5X2A, S06.5X5A, S06.5X7A, S06.5X9A, S06.891A, S06.895A, S06.897A, S06.9X0A, S06.9X4A, S06.9X7A, S12.001B, S12.01XB, S12.030B, S12.031A, S12.040A, S12.100A, S12.130B, S12.200A, S12.251A, S12.290A, S12.291A, S12.291B, S12.330A, S12.330B, S12.351A, S12.400A, S12.430A, S12.430B, S12.431B, S12.530A, S12.530B, S12.531B, S12.591A, S12.591B, S12.630B, S12.690A, S12.691A, S14.101A, S14.102A, S14.103A, S14.111A, S14.115A, S14.127A, S14.132A, S14.136A, S14.156A, S22.000B, S22.002B, S22.008B, S22.018A, S22.019B, S22.028B, S22.029A, S22.040A, S22.079A, S22.080A, S22.082A, S22.088A, S22.089B, S22.22XB, S22.31XA, S22.31XB, S22.42XB, S22.5XXA, S22.5XXB, S24.103A, S24.114A, S24.132A, S24.133A, S24.151A, S24.152A, S32.010B, S32.011B, S32.012B, S32.019A, S32.020A, S32.020B, S32.021B, S32.022B, S32.031A, S32.038B, S32.039B, S32.040A, S32.041A, S32.049B, S32.059A, S32.111A, S32.129A, S32.131B, S32.132B, S32.139A, S32.139B, S32.14XA, S32.17XA, S32.2XXA, S32.309A, S32.311B, S32.312A, S32.313B, S32.314A, S32.315B, S32.316A, S32.411A, S32.423B, S32.424B, S32.425B, S32.432B, S32.434A, S32.442A, S32.452A, S32.453A, S32.461B, S32.462B, S32.463B, S32.466A, S32.475B, S32.481B, S32.491A, S32.492B, S32.499A, S32.512B, S32.519B, S32.591B, S32.592A, S32.592B, S32.601B, S32.602A, S32.609B, S32.611A, S32.611B, S32.613A, S32.616B, S32.692B, S32.9XXA, S34.102A, S34.103A, S34.121A, S34.124A, S34.132A, S34.3XXA, S42.001B, S42.012A, S42.014B, S42.017A, S42.018B, S42.031B, S42.032A, S42.101B, S42.102B, S42.111B, S42.113B, S42.131B, S42.133B, S42.136B, S42.142B, S42.144A, S42.145A, S42.154A, S42.155B, S42.156B, S42.199A, S42.209B, S42.211A, S42.215B, S42.216B, S42.221A, S42.224B, S42.225B, S42.226A, S42.232A, S42.241A, S42.249B, S42.252A, S42.252B, S42.253A, S42.254A, S42.256B, S42.261B, S42.272A, S42.279A, S42.292B, S42.296B, S42.302A, S42.302B, S42.319A, S42.322B, S42.332B, S42.333A, S42.344B, S42.361A, S42.363A, S42.363B, S42.391B, S42.392B, S42.402A, S42.402B, S42.409A, S42.421A, S42.422A, S42.424B, S42.426B, S42.432B, S42.433B, S42.434B, S42.445B, S42.446A, S42.447B, S42.451B, S42.455B, S42.461A, S42.461B, S42.462B, S42.465B, S42.471A, S42.471B, S42.473A, S42.476A, S42.90XB, S42.91XB, S49.001A, S49.011A, S49.012A, S49.022A, S49.031A, S49.042A, S49.099A, S49.149A, S49.191A, S49.199A, S52.012A, S52.021A, S52.023B, S52.023C, S52.031A, S52.032C, S52.034A, S52.034B, S52.035C, S52.036B, S52.041B, S52.044A, S52.092B, S52.099B, S52.101A, S52.101B,

© 2023 Lin KJ et al. *JAMA Network Open*.

	S52.102C, S52.109B, S52.109C, S52.124B, S52.182C, S52.189B, S52.201B, S52.202B, S52.202C, S52.209A, S52.219A, S52.222A, S52.224B, S52.226C, S52.233A, S52.234A, S52.236C, S52.244A, S52.244C, S52.245B, S52.251A, S52.252C, S52.254C, S52.261B, S52.263C, S52.264B, S52.279A, S52.281B, S52.291A, S52.291B, S52.292A, S52.302B, S52.309C, S52.321B, S52.322A, S52.322C, S52.323A, S52.323C, S52.324B, S52.326B, S52.331A, S52.331C, S52.332B, S52.333B, S52.334C, S52.335A, S52.342B, S52.342C, S52.343A, S52.343B, S52.345B, S52.356C, S52.361C, S52.362C, S52.364B, S52.364C, S52.366B, S52.372C, S52.379A, S52.392C, S52.502A, S52.509B, S52.513B, S52.539A, S52.539C, S52.549A, S52.549B, S52.549C, S52.551B, S52.559A, S52.559B, S52.571B, S52.579A, S52.579C, S52.591C, S52.592A, S52.599B, S52.601B, S52.602C, S52.612B, S52.613B, S52.614A, S52.614C, S52.616C, S52.699A, S52.91XC, S59.009A, S59.012A, S59.041A, S59.101A, S59.112A, S59.129A, S59.149A, S59.211A, S59.229A, S59.239A, S59.291A, S59.299A, S62.001A, S62.009A, S62.014B, S62.023A, S62.025B, S62.026A, S62.032B, S62.035A, S62.036B, S62.102B, S62.112B, S62.113A, S62.115B, S62.121A, S62.121B, S62.122A, S62.122B, S62.126A, S62.131B, S62.134B, S62.135B, S62.136A, S62.142A, S62.152A, S62.154A, S62.156B, S62.163B, S62.173B, S62.182A, S62.183B, S62.185B, S62.209B, S62.222B, S62.225B, S62.231A, S62.232A, S62.244B, S62.246B, S62.255B, S62.256A, S62.302B, S62.303B, S62.304A, S62.306B, S62.308A, S62.308B, S62.311B, S62.317B, S62.321B, S62.325A, S62.332A, S62.336A, S62.336B, S62.340B, S62.342B, S62.346B, S62.347B, S62.348A, S62.348B, S62.355B, S62.356A, S62.356B, S62.357A, S62.361B, S62.362B, S62.365B, S62.367A, S62.391B, S62.398A, S62.399A, S62.511A, S62.512B, S62.513B, S62.515A, S62.521B, S62.522A, S62.601A, S62.602B, S62.603A, S62.605A, S62.605B, S62.607A, S62.608B, S62.609A, S62.610B, S62.611B, S62.612B, S62.614B, S62.615A, S62.620A, S62.620B, S62.621A, S62.624A, S62.628B, S62.630B, S62.631B, S62.637A, S62.639A, S62.640A, S62.642B, S62.643B, S62.645B, S62.647A, S62.652A, S62.657A, S62.659B, S62.660A, S62.662B, S62.665A, S62.667B, S62.669A, S62.91XA, S72.009A, S72.009B, S72.012A, S72.012C, S72.019C, S72.021B, S72.023A, S72.023B, S72.024C, S72.026A, S72.026B, S72.026C, S72.031A, S72.032C, S72.034C, S72.035B, S72.036B, S72.042B, S72.051C, S72.059B, S72.061A, S72.064A, S72.065B, S72.065C, S72.091B, S72.099C, S72.101A, S72.101B, S72.102A, S72.102C, S72.109A, S72.113A, S72.115B, S72.116A, S72.116C, S72.122C, S72.125A, S72.131C, S72.132C, S72.133C, S72.136C, S72.142B, S72.143B, S72.143C, S72.144C, S72.145C, S72.23XB, S72.24XB, S72.25XB, S72.25XC, S72.26XA, S72.309A, S72.309B, S72.323B, S72.324B, S72.324C, S72.326A, S72.326B, S72.326C, S72.336B, S72.341A, S72.346A, S72.353A, S72.353C, S72.355A, S72.361B, S72.365A, S72.365C, S72.391C, S72.399C, S72.401A, S72.409A, S72.412B, S72.414A, S72.415B, S72.423C, S72.424A, S72.424B, S72.424C, S72.431C, S72.435A, S72.442A, S72.443B, S72.443C, S72.444C, S72.451B, S72.451C, S72.452A, S72.452B, S72.454A, S72.454B, S72.456B, S72.456C, S72.461C, S72.462B, S72.464A, S72.471A, S72.491B, S72.499A, S72.499C, S72.8X1B, S72.91XC, S72.92XB, S79.001A, S79.012A, S79.019A, S79.101A, S79.112A, S79.121A, S82.001A, S82.001C, S82.002B, S82.009C, S82.013A, S82.014B, S82.014C, S82.015B, S82.015C, S82.024A, S82.025C, S82.026A, S82.034A, S82.035B, S82.036A, S82.041B, S82.043C, S82.045C, S82.102A, S82.111A, S82.111C, S82.112A, S82.116B, S82.116C, S82.122A, S82.122C, S82.123B, S82.125C, S82.126A, S82.131C, S82.133B, S82.135C, S82.136C, S82.141B, S82.141C, S82.142A, S82.143C, S82.145B, S82.145C, S82.151A, S82.151B, S82.151C, S82.155A, S82.156B, S82.162A, S82.191A, S82.192C, S82.202C, S82.221C, S82.222C, S82.224C, S82.226A, S82.231C, S82.232A, S82.233A, S82.233B, S82.234C, S82.244C, S82.253B, S82.256A, S82.262B, S82.262C, S82.263A, S82.263C, S82.266A, S82.266C, S82.291A, S82.302C, S82.391B, S82.392A, S82.399A, S82.399C, S82.401C, S82.402A, S82.402C, S82.409A, S82.424B, S82.425B, S82.426B, S82.431A, S82.431B, S82.433B, S82.434A, S82.435A, S82.435C, S82.441A, S82.441B, S82.442B, S82.443A, S82.444B, S82.444C, S82.445A, S82.445C, S82.446C, S82.463A, S82.466A, S82.491C, S82.499B, S82.52XB, S82.52XC, S82.54XA, S82.61XC, S82.62XB, S82.62XC, S82.64XA, S82.811A, S82.812A, S82.822A, S82.831B, S82.839A, S82.842C, S82.846A, S82.846B, S82.854A, S82.854C, S82.861A, S82.861B, S82.862C, S82.865B, S82.865C, S82.872A, S82.872B, S82.873A, S82.873C, S82.874A, S82.875C, S82.876C, S82.892C, S82.90XA, S89.001A, S89.022A, S89.029A, S89.031A, S89.091A, S89.121A, S89.129A, S89.131A, S89.132A, S89.141A, S89.191A, S89.211A, S89.292A, S89.299A, S89.302A, S89.319A, S92.001B, S92.002A, S92.021B, S92.024A, S92.026A, S92.026B, S92.032A, S92.034A, S92.042B, S92.046B, S92.051A, S92.062B, S92.063B, S92.066A, S92.112A, S92.112B, S92.114A, S92.121A, S92.122A, S92.126A, S92.146B, S92.151A, S92.209A, S92.209B, S92.213B, S92.214A, S92.221B, S92.224B, S92.231A, S92.232A, S92.234A, S92.236A, S92.236B, S92.241A, S92.243A, S92.245A, S92.251A, S92.252A, S92.301A, S92.309A,

© 2023 Lin KJ et al. *JAMA Network Open*.

	S92.309B, S92.311A, S92.315A, S92.316B, S92.325A, S92.326B, S92.333B, S92.334A, S92.334B, S92.335A, S92.351A, S92.352B, S92.354B, S92.406A, S92.411B, S92.416A, S92.421B, S92.423B, S92.426A, S92.499B, S92.502B, S92.503B, S92.505B, S92.511B, S92.522B, S92.525A, S92.526B, S92.532B, S92.534A, S92.535A, S92.535B, S92.536B, S92.591B, S92.911A
Major GI Bleeding	ICD-9 Diagnosis Code (any position) is any of: 430, 432.9, 568.81, 569.83, 569.86, 599.70, 599.71, 719.1, 719.10, 719.15, 719.18, 784.7, 784.8, 786.3, 786.30, 786.39, 423.0, 431, 432.0, 432.1, 459.0, 599.7, 599.72, 719.11, 719.12, 719.13, 719.14, 719.16, 719.17, 719.19, 786.31, 455.2, 455.5, 455.6, 455.7, 455.8, 456.0, 530.1, 530.11, 530.13, 530.19, 530.7, 531.3, 531.40, 531.5, 531.60, 531.61, 532.20, 532.3, 532.4, 532.41, 532.5, 532.6, 532.61, 532.9, 533.0, 533.00, 533.01, 533.20, 533.3, 533.40, 533.6, 533.60, 533.61, 533.7, 534.01, 534.2, 534.3, 534.4, 534.40, 534.41, 534.5, 534.6, 534.9, 535.00, 535.11, 535.20, 535.30, 535.41, 535.50, 535.61, 562.00, 562.02, 562.03, 562.10, 562.11, 562.12, 562.13, 569.3, 569.85, 578.1, 455.1, 455.4, 455.9, 456.20, 530.10, 530.12, 530.82, 531.0, 531.00, 531.01, 531.1, 531.2, 531.20, 531.21, 531.4, 531.41, 531.6, 531.7, 531.9, 532.0, 532.00, 532.01, 532.1, 532.2, 532.21, 532.40, 532.60, 532.7, 533.1, 533.2, 533.21, 533.4, 533.41, 533.5, 533.9, 534.0, 534.00, 534.1, 534.20, 534.21, 534.60, 534.61, 534.7, 535.01, 535.10, 535.21, 535.31, 535.40, 535.51, 535.60, 537.83, 562.01, 578.0, 578.9<br>ICD-10 Diagnosis Code (any position) is any of: I31.2, I60.01, I60.10, I60.11, I60.31, I60.51, I60.52, I60.6, I60.8, I61.4, I61.8, I62.1, K56.60, K57.33, K63.1, K66.1, M25.011, M25.012, M25.022, M25.032, M25.041, M25.042, M25.049, M25.059, M25.061, M25.062, M25.069, M25.071, M25.074, M25.076, M25.08, R04, R04.0, R04.1, R04.2, R04.8, R04.9, R31.0, R31.1, R31.2, R31.21, R31.9, R58, I60.00, I60.02, I60.12, I60.2, I60.30, I60.32, I60.4, I60.50, I60.7, I60.9, I61.0, I61.1, I61.2, I61.3, I61.5, I61.6, I61.9, I62.00, I62.01, I62.02, I62.03, I62.9, K57.13, K63.81, M25.00, M25.019, M25.021, M25.029, M25.031, M25.039, M25.051, M25.052, M25.072, M25.073, M25.075, R04.81, R04.89, R31, R31.29, I85.11, K20, K20.8, K21.0, K22.11, K22.6, K25.0, K25.4, K25.6, K26.1, K26.4, K26.5, K26.6, K27.2, K27.5, K27.6, K28.0, K28.4, K28.5, K29.01, K29.21, K29.41, K31.811, K55.21, K57.01, K57.21, K57.51, K57.53, K57.93, K64.0, K64.5, K64.8, K64.9, K92.0, I85.01, K20.0, K20.9, K25.1, K25.2, K25.5, K26.0, K26.2, K27.0, K27.1, K27.4, K28.1, K28.2, K28.6, K29.31, K29.51, K29.61, K29.71, K29.81, K29.91, K57.11, K57.31, K57.41, K57.81, K57.91, K62.5, K64.1, K64.2, K64.3, K64.4, K92.1, K92.2<br>ICD-9 Procedure Code (any position) is any of: 44.43<br>ICD-10 Procedure Code (any position) is any of: 0W3P8ZZ<br>HCPCS Procedure Code (any position) is any of: 43255
Home Oxygen Use	ICD-9 Diagnosis Code (any position) is any of: V46.2<br>ICD-10 Diagnosis Code (any position) is any of: Z99.81
Hypertension	ICD-9 Diagnosis Code (any position) is any of: 401.0, 401.1, 401.9, 402.0, 402.00, 402.01, 402.1, 402.10, 402.11, 402.9, 402.90, 402.91, 403.0, 403.00, 403.01, 403.1, 403.10, 403.11, 403.9, 403.90, 403.91, 404.0, 404.00, 404.01, 404.02, 404.03, 404.1, 404.10, 404.11, 404.12, 404.13, 404.9, 404.90, 404.91, 404.92, 404.93, 405.0, 405.01, 405.09, 405.1, 405.11, 405.19, 405.9, 405.91, 405.99<br>ICD-10 Diagnosis Code (any position) is any of: I10, I13.2, I15.0, I15.8, I13.11, I13.10, I12.0, I13.0, I11.9, I12.9
Malignancy	ICD-9 Diagnosis Code (any position) is any of: 140.0, 140.1, 140.3, 140.4, 140.5, 140.6, 140.8, 140.9, 141.0, 141.1, 141.2, 141.3, 141.4, 141.5, 141.6, 141.8, 141.9, 142.0, 142.1, 142.2, 142.8, 142.9, 143.0, 143.1, 143.8, 143.9, 144.0, 144.1, 144.8, 144.9, 145.0, 145.1, 145.2, 145.3, 145.4, 145.5, 145.6, 145.8, 145.9, 146.0, 146.1, 146.2, 146.3, 146.4, 146.5, 146.6, 146.7, 146.8, 146.9, 147.0, 147.1, 147.2, 147.3, 147.8, 147.9, 148.0, 148.1, 148.2, 148.3, 148.8, 148.9, 149.0, 149.1, 149.8, 149.9, 150.0, 150.1, 150.2, 150.3, 150.4, 150.5, 150.8, 150.9, 151.0, 151.1, 151.2, 151.3, 151.4, 151.5, 151.6, 151.8, 151.9, 152.0, 152.1, 152.2, 152.3, 152.8, 152.9, 153.0, 153.1, 153.2, 153.3, 153.4, 153.5, 153.6, 153.7, 153.8, 153.9, 154.0, 154.1, 154.2, 154.3, 154.8, 155.0, 155.1, 155.2, 156.0, 156.1, 156.2, 156.8, 156.9, 157.0, 157.1, 157.2, 157.3, 157.4, 157.8, 157.9, 158.0, 158.8, 158.9, 159.0, 159.1, 159.8, 159.9, 160.0, 160.1, 160.2, 160.3, 160.4, 160.5, 160.8, 160.9, 161.0, 161.1, 161.2, 161.3, 161.8, 161.9, 162.0, 162.2, 162.3, 162.4, 162.5, 162.8, 162.9, 163.0, 163.1, 163.8, 163.9, 164.0, 164.1, 164.2, 164.3, 164.8, 164.9, 165.0, 165.8, 165.9, 170.0, 170.1, 170.2, 170.3, 170.4, 170.5, 170.6, 170.7, 170.8, 170.9, 171.0, 171.2, 171.3, 171.4, 171.5, 171.6, 171.7, 171.8, 171.9, 172.0, 172.1, 172.2, 172.3, 172.4, 172.5, 172.6, 172.7, 172.8, 172.9, 173.0, 173.00, 173.01, 173.02, 173.09, 173.1, 173.10, 173.11, 173.12, 173.19, 173.2, 173.20, 173.21, 173.22, 173.29, 173.3, 173.30, 173.31, 173.32, 173.39,

© 2023 Lin KJ et al. *JAMA Network Open*.

	173.4, 173.40, 173.41, 173.42, 173.49, 173.5, 173.50, 173.51, 173.52, 173.59, 173.6, 173.60, 173.61, 173.62, 173.69, 173.7, 173.70, 173.71, 173.72, 173.79, 173.8, 173.80, 173.81, 173.82, 173.89, 173.9, 173.90, 173.91, 173.92, 173.99, 174.0, 174.1, 174.2, 174.3, 174.4, 174.5, 174.6, 174.8, 174.9, 175.0, 175.9, 176.0, 176.1, 176.2, 176.3, 176.4, 176.5, 176.8, 176.9, 180.0, 180.1, 180.8, 180.9, 182.0, 182.1, 182.8, 183.0, 183.2, 183.3, 183.4, 183.5, 183.8, 183.9, 184.0, 184.1, 184.2, 184.3, 184.4, 184.8, 184.9, 186.0, 186.9, 187.1, 187.2, 187.3, 187.4, 187.5, 187.6, 187.7, 187.8, 187.9, 188.0, 188.1, 188.2, 188.3, 188.4, 188.5, 188.6, 188.7, 188.8, 188.9, 189.0, 189.1, 189.2, 189.3, 189.4, 189.8, 189.9, 190.0, 190.1, 190.2, 190.3, 190.4, 190.5, 190.6, 190.7, 190.8, 190.9, 191.0, 191.1, 191.2, 191.3, 191.4, 191.5, 191.6, 191.7, 191.8, 191.9, 192.0, 192.1, 192.2, 192.3, 192.8, 192.9, 194.0, 194.1, 194.3, 194.4, 194.5, 194.6, 194.8, 194.9, 195.0, 195.1, 195.2, 195.3, 195.4, 195.5, 195.8, 196.0, 196.1, 196.2, 196.3, 196.5, 196.6, 196.8, 196.9, 197.0, 197.1, 197.2, 197.3, 197.4, 197.5, 197.6, 197.7, 197.8, 198.0, 198.1, 198.2, 198.3, 198.4, 198.5, 198.6, 198.7, 198.8, 198.81, 198.82, 198.89, 199.0, 199.1, 199.2, 200.0, 200.00, 200.01, 200.02, 200.03, 200.04, 200.05, 200.06, 200.07, 200.08, 200.1, 200.10, 200.11, 200.12, 200.13, 200.14, 200.15, 200.16, 200.17, 200.18, 200.2, 200.20, 200.21, 200.22, 200.23, 200.24, 200.25, 200.26, 200.27, 200.28, 200.3, 200.30, 200.31, 200.32, 200.33, 200.34, 200.35, 200.36, 200.37, 200.38, 200.4, 200.40, 200.41, 200.42, 200.43, 200.44, 200.45, 200.46, 200.47, 200.48, 200.5, 200.50, 200.51, 200.52, 200.53, 200.54, 200.55, 200.56, 200.57, 200.58, 200.6, 200.60, 200.61, 200.62, 200.63, 200.64, 200.65, 200.66, 200.67, 200.68, 200.7, 200.70, 200.71, 200.72, 200.73, 200.74, 200.75, 200.76, 200.77, 200.78, 200.8, 200.80, 200.81, 200.82, 200.83, 200.84, 200.85, 200.86, 200.87, 200.88, 201.0, 201.00, 201.01, 201.02, 201.03, 201.04, 201.05, 201.06, 201.07, 201.08, 201.1, 201.10, 201.11, 201.12, 201.13, 201.14, 201.15, 201.16, 201.17, 201.18, 201.2, 201.20, 201.21, 201.22, 201.23, 201.24, 201.25, 201.26, 201.27, 201.28, 201.4, 201.40, 201.41, 201.42, 201.43, 201.44, 201.45, 201.46, 201.47, 201.48, 201.5, 201.50, 201.51, 201.52, 201.53, 201.54, 201.55, 201.56, 201.57, 201.58, 201.6, 201.60, 201.61, 201.62, 201.63, 201.64, 201.65, 201.66, 201.67, 201.68, 201.7, 201.70, 201.71, 201.72, 201.73, 201.74, 201.75, 201.76, 201.77, 201.78, 201.9, 201.90, 201.91, 201.92, 201.93, 201.94, 201.95, 201.96, 201.97, 201.98, 202.0, 202.00, 202.01, 202.02, 202.03, 202.04, 202.05, 202.06, 202.07, 202.08, 202.1, 202.10, 202.11, 202.12, 202.13, 202.14, 202.15, 202.16, 202.17, 202.18, 202.2, 202.20, 202.21, 202.22, 202.23, 202.24, 202.25, 202.26, 202.27, 202.28, 202.3, 202.30, 202.31, 202.32, 202.33, 202.34, 202.35, 202.36, 202.37, 202.38, 202.4, 202.40, 202.41, 202.42, 202.43, 202.44, 202.45, 202.46, 202.47, 202.48, 202.5, 202.50, 202.51, 202.52, 202.53, 202.54, 202.55, 202.56, 202.57, 202.58, 202.6, 202.60, 202.61, 202.62, 202.63, 202.64, 202.65, 202.66, 202.67, 202.68, 202.7, 202.70, 202.71, 202.72, 202.73, 202.74, 202.75, 202.76, 202.77, 202.78, 202.8, 202.80, 202.81, 202.82, 202.83, 202.84, 202.85, 202.86, 202.87, 202.88, 202.9, 202.90, 202.91, 202.92, 202.93, 202.94, 202.95, 202.96, 202.97, 202.98, 203.0, 203.00, 203.01, 203.02, 203.1, 203.10, 203.11, 203.12, 203.8, 203.80, 203.81, 203.82, 204.0, 204.00, 204.01, 204.02, 204.1, 204.10, 204.11, 204.12, 204.2, 204.20, 204.21, 204.22, 204.8, 204.80, 204.81, 204.82, 204.9, 204.90, 204.91, 204.92, 205.0, 205.00, 205.01, 205.02, 205.1, 205.10, 205.11, 205.12, 205.2, 205.20, 205.21, 205.22, 205.3, 205.30, 205.31, 205.32, 205.8, 205.80, 205.81, 205.82, 205.9, 205.90, 205.91, 205.92, 206.0, 206.00, 206.01, 206.02, 206.1, 206.10, 206.11, 206.12, 206.2, 206.20, 206.21, 206.22, 206.8, 206.80, 206.81, 206.82, 206.9, 206.90, 206.91, 206.92, 207.0, 207.00, 207.01, 207.02, 207.1, 207.10, 207.11, 207.12, 207.2, 207.20, 207.21, 207.22, 207.8, 207.80, 207.81, 207.82, 208.0, 208.00, 208.01, 208.02, 208.1, 208.10, 208.11, 208.12, 208.2, 208.20, 208.21, 208.22, 208.8, 208.80, 208.81, 208.82, 208.9, 208.90, 208.91, 208.92<br>ICD-10 Diagnosis Code (any position) is any of: C00.1, C00.6, C02.0, C02.1, C02.3, C04.9, C05, C05.0, C05.1, C06, C07, C09.0, C09.9, C10, C10.0, C10.2, C10.3, C10.4, C11.8, C13.0, C15.3, C16, C16.0, C16.4, C16.5, C17.9, C26, C26.9, C32, C32.9, C34.0, C34.00, C34.11, C34.12, C34.2, C34.3, C34.80, C34.82, C34.91, C38.1, C38.3, C38.8, C40.02, C40.10, C40.3, C40.91, C41.2, C41.3, C43.11, C43.21, C43.30, C43.4, C43.60, C43.62, C45.7, C46.4, C46.5, C46.52, C47, C47.20, C47.6, C48.0, C48.1, C49.1, C49.10, C49.11, C49.12, C49.20, C50, C50.012, C50.02, C50.112, C50.12, C50.121, C50.129, C50.229, C50.31, C50.319, C50.41, C50.411, C50.422, C50.611, C50.91, C50.912, C50.919, C50.921, C51.9, C54.8, C56, C56.9, C57.00, C57.01, C57.1, C57.11, C57.2, C57.22, C57.3, C57.4, C57.9, C60.8, C61, C62.00, C62.01, C62.02, C62.10, C63.7, C66.9, C67, C67.9, C69.00, C69.11, C69.31, C69.32, C69.4, C69.40, C69.5, C69.51, C69.62, C69.9, C69.90, C70.9, C71.6, C71.9, C72.0, C72.22, C72.3, C74.0, C74.91, C75, C76, C76.2, C76.3, C76.50, C81.0, C81.05, C81.10, C81.12, C81.20, C81.21, C81.28, C81.93, C82.00, C82.01, C82.02,

© 2023 Lin KJ et al. *JAMA Network Open*.

	C82.07, C82.08, C82.21, C82.29, C82.3, C82.31, C82.32, C82.41, C82.44, C82.45, C82.47, C82.48, C82.60, C82.80, C82.84, C82.88, C82.95, C82.97, C83.04, C83.13, C83.16, C83.18, C83.19, C83.30, C83.34, C83.37, C83.50, C83.51, C83.56, C83.57, C83.58, C83.59, C83.8, C83.80, C83.92, C84.0, C84.08, C84.09, C84.42, C84.46, C84.74, C84.75, C84.76, C84.92, C84.96, C85.20, C85.27, C85.80, C85.83, C85.86, C85.89, C85.90, C85.97, C90.2, C95, C95.0, C95.02, C96.2, C96.9, C96.Z, D45, C00.2, C00.4, C00.5, C02.2, C02.9, C03, C04, C06.0, C06.8, C08.0, C08.9, C09, C09.1, C10.1, C10.9, C13.2, C13.9, C14.0, C15.4, C16.1, C16.3, C17.1, C17.3, C18.1, C18.8, C18.9, C20, C21.2, C22.1, C22.2, C22.8, C24, C24.0, C24.8, C25.2, C26.0, C31.0, C31.9, C32.2, C34.02, C34.8, C34.9, C34.90, C34.92, C37, C38.2, C39, C39.0, C40.0, C40.92, C41.0, C43.3, C43.39, C43.51, C43.52, C43.72, C43.8, C45.1, C45.2, C46.0, C47.10, C47.2, C48.2, C49.2, C49.8, C50.019, C50.11, C50.221, C50.32, C50.321, C50.519, C50.529, C50.629, C50.81, C50.82, C50.822, C50.829, C51.2, C53, C54.0, C55, C57.21, C57.8, C62.11, C63.00, C63.10, C63.11, C64.1, C65.1, C65.2, C66.1, C67.3, C67.6, C67.7, C68.0, C68.1, C69.2, C69.52, C69.82, C69.91, C71.4, C71.8, C72.1, C72.20, C72.21, C72.4, C72.40, C73, C74.1, C74.90, C75.0, C75.2, C75.5, C75.8, C75.9, C76.40, C76.42, C81.22, C81.23, C81.31, C81.36, C81.44, C81.70, C81.94, C81.96, C81.97, C82.0, C82.03, C82.14, C82.15, C82.25, C82.33, C82.38, C82.40, C82.42, C82.46, C82.53, C82.6, C82.62, C82.65, C82.89, C82.98, C82.99, C83, C83.07, C83.09, C83.12, C83.32, C83.39, C83.53, C83.7, C83.70, C83.74, C83.79, C83.84, C83.85, C83.89, C83.96, C84.01, C84.03, C84.1, C84.11, C84.13, C84.14, C84.19, C84.61, C84.62, C84.71, C84.72, C84.77, C84.79, C84.97, C84.A1, C84.A7, C84.A9, C84.Z3, C84.Z6, C84.Z7, C85.1, C85.10, C85.12, C85.14, C85.15, C85.21, C85.25, C85.28, C85.85, C85.88, C85.91, C88.8, C88.9, C90.0, C94.32, C94.8, C94.80, C94.82, C95.90, C96.4, C96.A, C00, C02.8, C03.9, C04.0, C04.8, C06.80, C08, C11, C11.0, C11.2, C13, C14, C15.8, C17.2, C18.0, C18.3, C18.4, C18.6, C18.7, C21.1, C22.0, C24.9, C25.0, C25.1, C25.8, C26.1, C30.1, C31.2, C31.8, C32.0, C32.1, C32.3, C32.8, C33, C34.10, C34.31, C34.32, C38, C38.0, C40.00, C40.12, C40.20, C40.21, C40.30, C40.31, C40.82, C41, C41.9, C43.10, C43.5, C43.59, C43.6, C43.9, C45.9, C46.2, C47.21, C47.22, C47.5, C47.8, C48, C49, C49.0, C49.4, C50.0, C50.011, C50.021, C50.029, C50.111, C50.122, C50.211, C50.219, C50.22, C50.3, C50.311, C50.322, C50.329, C50.4, C50.419, C50.42, C50.5, C50.522, C50.612, C50.622, C50.819, C50.821, C50.9, C50.911, C50.922, C51, C51.1, C52, C54, C54.1, C56.1, C57, C57.12, C57.20, C57.7, C58, C60, C60.9, C62.1, C63.0, C63.01, C63.8, C64, C64.2, C64.9, C67.0, C67.5, C68.8, C68.9, C69, C69.0, C69.3, C69.41, C69.60, C69.80, C69.81, C69.92, C70.0, C71.1, C71.3, C71.7, C72.2, C72.42, C72.9, C74.10, C75.3, C76.41, C76.5, C76.52, C81.02, C81.04, C81.06, C81.18, C81.19, C81.27, C81.29, C81.40, C81.41, C81.43, C81.45, C81.72, C81.73, C81.74, C81.76, C81.91, C81.95, C81.98, C82.05, C82.09, C82.10, C82.19, C82.20, C82.26, C82.27, C82.36, C82.52, C82.54, C82.55, C82.56, C82.61, C82.66, C82.69, C82.8, C82.86, C82.87, C82.90, C83.0, C83.02, C83.08, C83.1, C83.11, C83.31, C83.81, C83.86, C83.88, C83.90, C83.97, C83.98, C84.04, C84.05, C84.12, C84.47, C84.65, C84.67, C84.78, C84.9, C84.A3, C84.A4, C84.A5, C84.A8, C84.Z0, C84.Z4, C84.Z9, C85.13, C85.17, C85.24, C85.81, C85.87, C85.94, C85.99, C88.2, C88.3, C94.3, C95.1, C95.10, C95.9, C95.92, D89, C02, C02.4, C03.0, C05.2, C05.9, C06.1, C06.2, C06.89, C06.9, C08.1, C11.1, C11.3, C11.9, C14.2, C15, C15.5, C15.9, C16.2, C16.8, C17, C18, C18.5, C21.0, C21.8, C22.3, C22.7, C23, C25.3, C25.9, C30, C31.1, C38.4, C40.1, C40.22, C40.8, C40.9, C41.1, C41.4, C43.1, C43.12, C43.20, C43.22, C43.31, C43.71, C45.0, C46.1, C46.3, C46.51, C47.0, C47.1, C47.12, C47.9, C49.21, C49.5, C49.9, C50.022, C50.21, C50.212, C50.222, C50.312, C50.429, C50.511, C50.52, C50.6, C50.61, C50.621, C50.92, C50.929, C51.0, C51.8, C53.1, C53.9, C57.10, C60.0, C60.1, C60.2, C62.90, C62.92, C63.02, C63.1, C63.2, C63.9, C65.9, C66, C67.1, C67.2, C67.4, C68, C69.01, C69.1, C69.21, C69.22, C69.61, C69.8, C71, C71.2, C72.30, C72.31, C72.50, C72.59, C74.02, C74.11, C75.1, C75.4, C76.1, C76.51, C81.00, C81.07, C81.11, C81.13, C81.14, C81.15, C81.16, C81.17, C81.2, C81.25, C81.38, C81.39, C81.42, C81.46, C81.48, C81.49, C81.7, C81.71, C81.75, C81.77, C81.99, C82, C82.04, C82.06, C82.13, C82.17, C82.22, C82.23, C82.30, C82.59, C82.64, C82.67, C82.81, C82.83, C82.85, C82.91, C82.92, C82.93, C82.96, C83.00, C83.01, C83.05, C83.15, C83.17, C83.3, C83.33, C83.36, C83.38, C83.5, C83.54, C83.71, C83.73, C83.77, C83.78, C83.87, C83.95, C83.99, C84, C84.00, C84.02, C84.17, C84.41, C84.44, C84.48, C84.6, C84.63, C84.66, C84.70, C84.91, C84.93, C84.95, C84.A, C84.A2, C85.18, C85.19, C85.22, C85.23, C85.8, C85.82, C85.84, C85.9, C85.92, C85.93, C85.96, C85.98, C94.30, C94.31, C95.00, C95.12, C00.0, C00.3, C00.8, C00.9, C01, C03.1, C04.1, C05.8, C09.8, C10.8, C12, C13.1, C13.8, C14.8, C16.6, C16.9, C17.0, C17.8, C18.2, C19, C21, C22, C22.4, C22.9, C24.1, C25, C25.4, C25.7, C30.0, C31, C31.3, C34, C34.01, C34.1, C34.30, C34.81, C39.9, C40, C40.01, C40.11, C40.2, C40.32, C40.80, C40.81, C40.90, C43.0, C43.2, C43.61,

© 2023 Lin KJ et al. *JAMA Network Open*.

	C43.7, C43.70, C46, C46.50, C46.7, C46.9, C47.11, C47.3, C47.4, C48.8, C49.22, C49.3, C49.6, C50.01, C50.1, C50.119, C50.2, C50.412, C50.421, C50.51, C50.512, C50.521, C50.619, C50.62, C50.8, C50.811, C50.812, C53.0, C53.8, C54.2, C54.3, C54.9, C56.2, C57.0, C57.02, C62, C62.0, C62.12, C62.9, C62.91, C63, C63.12, C65, C66.2, C67.8, C69.02, C69.10, C69.12, C69.20, C69.30, C69.42, C69.50, C69.6, C70, C70.1, C71.0, C71.5, C72, C72.32, C72.41, C72.5, C74, C74.00, C74.01, C74.12, C74.9, C74.92, C76.0, C76.4, C76.8, C81, C81.01, C81.03, C81.08, C81.09, C81.1, C81.24, C81.26, C81.3, C81.30, C81.32, C81.33, C81.34, C81.35, C81.37, C81.4, C81.47, C81.78, C81.79, C81.9, C81.90, C81.92, C82.1, C82.11, C82.12, C82.16, C82.18, C82.2, C82.24, C82.28, C82.34, C82.35, C82.37, C82.39, C82.4, C82.43, C82.49, C82.5, C82.50, C82.51, C82.57, C82.58, C82.63, C82.68, C82.82, C82.9, C82.94, C83.03, C83.06, C83.10, C83.14, C83.35, C83.52, C83.55, C83.72, C83.75, C83.76, C83.82, C83.83, C83.9, C83.91, C83.93, C83.94, C84.06, C84.07, C84.10, C84.15, C84.16, C84.18, C84.4, C84.40, C84.43, C84.45, C84.49, C84.60, C84.64, C84.68, C84.69, C84.7, C84.73, C84.90, C84.94, C84.98, C84.99, C84.A0, C84.A6, C84.Z, C84.Z1, C84.Z2, C84.Z5, C84.Z8, C85.11, C85.16, C85.2, C85.26, C85.29, C85.95, C88.0, C88.4, C94.81, C95.01, C95.11, C95.91, C96.0, C96.5, C96.6, Z85.46
Ischemic Heart Disease	ICD-9 Diagnosis Code (any position) is any of: 410.1, 410.10, 410.11, 410.20, 410.31, 410.7, 410.70, 410.71, 410.9, 410.92, 411.1, 411.81, 413.0, 414.0, 414.00, 414.01, 414.02, 414.03, 414.06, 410.21, 410.22, 410.41, 410.61, 410.72, 411.89, 412, 413.9, 414.12, 414.2, 410.0, 410.00, 410.3, 410.32, 410.4, 410.42, 410.5, 410.62, 410.80, 410.81, 410.82, 411.0, 414.11, 414.3, 414.8, 410.01, 410.02, 410.12, 410.2, 410.30, 410.51, 410.52, 410.90, 410.91, 411.8, 414.05, 414.07, 414.10, 414.19, 414.4, 414.9, 410.40, 410.50, 410.6, 410.60, 410.8, 413.1, 414.04, 414.1<br>ICD-10 Diagnosis Code (any position) is any of: I20.0, I21.02, I21.09, I21.19, I21.29, I22.2, I22.8, I24.9, I25.111, I25.119, I25.5, I25.708, I25.710, I25.738, I25.751, I25.84, I20.8, I21.11, I24.1, I25.2, I25.719, I25.721, I25.728, I25.731, I25.758, I25.760, I25.761, I25.791, I25.811, I25.82, I25.89, I25.9, I21.4, I22.9, I24.0, I24.8, I25.3, I25.42, I25.6, I25.750, I25.812, I25.83, I20.9, I21.3, I22.0, I22.1, I25.118, I25.701, I25.709, I25.718, I25.730, I25.768, I25.769, I25.790, I25.798, I25.799, I20.1, I21.01, I21.21, I25.10, I25.110, I25.41, I25.700, I25.711, I25.720, I25.729, I25.739, I25.759, I25.810
Peripheral Vascular Disease	HCPCS Procedure Code (any position) is any of: 35256, 35286, 35363, 35459, 35492, 35556, 35582, 35583, 35646, 35661, 35681, 37207, 37222, 37226, 37227, 37229, 37230, 35351, 35371, 35454, 35470, 35485, 35495, 35533, 35546, 35551, 35585, 35647, 35666, 35671, 35682, 37228, 35355, 35473, 35541, 35548, 35563, 35637, 35641, 35651, 35654, 35656, 37208, 37225, 37235, 35549, 35558, 35565, 35566, 35570, 35571, 35587, 35638, 35663, 35683, 37220, 37232, 37234, 35361, 35372, 35381, 35456, 35474, 35482, 35483, 35493, 35521, 35621, 35623, 35879, 37221, 37223, 37224, 37231, 37233<br>ICD-9 Procedure Code (any position) is any of: 38.18, 38.49, 39.50, 39.55, 39.59, 39.9, 39.91, 39.94, 39.97, 39.29, 39.51, 39.52, 39.53, 39.54, 39.58, 39.90, 39.92, 39.99, 38.08, 38.09, 38.38, 38.48, 39.5, 39.56, 39.93, 39.96, 38.39, 39.25, 39.57, 39.95, 39.98<br>ICD-10 Procedure Code (any position) is any of: 0410098, 041009B, 041009K, 04100A8, 04100AG, 04100J6, 04100JH, 04100JJ, 04100KB, 04100KJ, 04100Z7, 04100Z8, 04100ZC, 04100ZD, 04100ZJ, 0410497, 041049C, 041049D, 041049H, 041049J, 04104A7, 04104A8, 04104AR, 04104J6, 04104JB, 04104JK, 04104K8, 04104Z9, 04104ZD, 04104ZK, 041C0JH, 041C0KK, 041C49K, 041C4AH, 041C4KJ, 041D09J, 041D0AK, 041D0JJ, 041D0KJ, 041D49H, 041D4AK, 041E0KJ, 041E0ZH, 041E49K, 041E4JH, 041E4JJ, 041E4JK, 041E4ZK, 041F0AH, 041F0AK, 041F49H, 041F4AJ, 041F4JH, 041F4KH, 041F4ZJ, 041F4ZK, 041H09H, 041H0AJ, 041H0JK, 041H0ZK, 041H4AH, 041H4JJ, 041H4JK, 041H4ZH, 041J09H, 041J09J, 041J0AJ, 041J0ZJ, 041J4KK, 041K09H, 041K09J, 041K09M, 041K0AH, 041K0AK, 041K0AQ, 041K0JJ, 041K0JK, 041K0JN, 041K0KK, 041K0KM, 041K0KQ, 041K0ZH, 041K49H, 041K49N, 041K4AH, 041K4AN, 041K4AP, 041K4AQ, 041K4AS, 041K4KH, 041K4ZH, 041K4ZL, 041L0AH, 041L0JS, 041L0KH, 041L0KK, 041L0KP, 041L0ZM, 041L49K, 041L4AL, 041L4AM, 041L4AQ, 041L4JP, 041L4KK, 041L4KS, 041L4ZQ, 041M0ZS, 041M4AS, 041M4ZQ, 041M4ZS, 041N09S, 041N0AM, 041N0AS, 041N49P, 041N4AM, 041N4JM, 041N4KP, 041N4ZM, 0470046, 0470056, 0470066, 047007Z, 0470346, 04703GZ, 047044Z, 0470456, 04704F6, 04704FZ, 04704GZ, 047C05Z, 047C07Z, 047C376, 047C3E6, 047C3EZ, 047C3G6, 047C4D6, 047C4DZ, 047C4E6, 047C4EZ, 047C4GZ, 047C4Z6, 047C4ZZ, 047D0F6, 047D0G6, 047D3DZ, 047D3EZ, 047D4E6, 047D4G6, 047E07Z, 047E0EZ, 047E0F6, 047E0FZ, 047E0Z6, 047E346, 047E37Z, 047E3E6, 047E3EZ, 047E3FZ, 047E44Z, 047E466, 047E4EZ, 047E4F6, 047E4GZ, 047E4ZZ, 047F066, 047F07Z, 047F0DZ, 047F0G6, 047F0Z6, 047F35Z, 047F376, 047F446, 047F476, 047F4Z6,

© 2023 Lin KJ et al. *JAMA Network Open*.

	047F4ZZ, 047H046, 047H05Z, 047H06Z, 047H0Z6, 047H0ZZ, 047H3DZ, 047H3E6, 047H466, 047H4G6, 047H4Z6, 047J066, 047J06Z, 047J0DZ, 047J0FZ, 047J34Z, 047J3FZ, 047J3G6, 047J3GZ, 047J3ZZ, 047J466, 047K056, 047K06Z, 047K0E6, 047K0G6, 047K0Z1, 047K0Z6, 047K0ZZ, 047K341, 047K376, 047K3D6, 047K3E6, 047K3FZ, 047K3Z6, 047K45Z, 047K46Z, 047K476, 047K4D1, 047K4DZ, 047K4E6, 047K4EZ, 047K4F6, 047L041, 047L0D1, 047L0E6, 047L0GZ, 047L0Z6, 047L341, 047L34Z, 047L36Z, 047L3Z1, 047L446, 047L4D1, 047L4GZ, 047L4ZZ, 047M05Z, 047M0D1, 047M0EZ, 047M35Z, 047M3D6, 047M3Z1, 047M3Z6, 047M3ZZ, 047M466, 047M4EZ, 047M4G6, 047M4Z1, 047M4Z6, 047N056, 047N346, 047N36Z, 047N3E6, 047N3GZ, 047N3Z6, 047N45Z, 047N4DZ, 047N4E6, 047N4Z1, 047P04Z, 047P066, 047P076, 047P07Z, 047P36Z, 047P3DZ, 047P3E6, 047P3G6, 047P3GZ, 047P476, 047P4DZ, 047P4G6, 047P4ZZ, 047Q066, 047Q06Z, 047Q3FZ, 047Q47Z, 047Q4D6, 047Q4Z6, 047R066, 047R0EZ, 047R0F6, 047R0GZ, 047R376, 047R3D6, 047R3G6, 047R3GZ, 047R446, 047R456, 047R4ZZ, 047S076, 047S07Z, 047S0EZ, 047S0G6, 047S34Z, 047S356, 047S35Z, 047S37Z, 047S3D6, 047S3FZ, 047S476, 047S47Z, 047S4Z6, 047T046, 047T0D6, 047T0EZ, 047T0G6, 047T0Z6, 047T356, 047T3DZ, 047T456, 047T476, 047T4E6, 047U056, 047U0E6, 047U0FZ, 047U34Z, 047U376, 047U3D6, 047U3EZ, 047U47Z, 047U4FZ, 047V076, 047V07Z, 047V0F6, 047V0Z6, 047V34Z, 047V3E6, 047V3EZ, 047V44Z, 047V46Z, 047V476, 047V4FZ, 047W06Z, 047W0DZ, 047W0EZ, 047W0F6, 047W0GZ, 047W36Z, 047W3EZ, 047W4D6, 047W4GZ, 047Y046, 047Y06Z, 047Y356, 047Y37Z, 047Y3D6, 047Y3E6, 047Y476, 047Y47Z, 047Y4EZ, 04BM0ZZ, 04BP4ZZ, 04BQ0ZZ, 04BS4ZZ, 04BU4ZZ, 04BY4ZZ, 04CM0Z6, 04CN3Z6, 04CP4Z6, 04CQ0ZZ, 04CQ3Z6, 04CR3Z6, 04CR3ZZ, 04CS3Z6, 04CS3ZZ, 04CS4ZZ, 04CT0Z6, 04CT4ZZ, 04CU4ZZ, 04CV3ZZ, 04CV4Z6, 04CW3ZZ, 04CY4Z6, 04HC0DZ, 04HF0DZ, 04HH0DZ, 04HH4DZ, 04HK0DZ, 04HK3DZ, 04HK4DZ, 04HN3DZ, 04HQ4DZ, 04HT4DZ, 04HU3DZ, 04HY0DZ, 04ND3ZZ, 04NK0ZZ, 04NK4ZZ, 04NM0ZZ, 04NQ4ZZ, 04NR3ZZ, 04NR4ZZ, 04NS0ZZ, 04NU3ZZ, 04RK0JZ, 04RK0KZ, 04RL0KZ, 04RL4JZ, 04RL4KZ, 04RM47Z, 04RM4JZ, 04RN47Z, 04RN4KZ, 04RP47Z, 04RQ47Z, 04RQ4JZ, 04RR07Z, 04RR0JZ, 04RR4JZ, 04RS4KZ, 04RT4KZ, 04RU4KZ, 04RV0JZ, 04RW47Z, 04RW4JZ, 04RY0JZ, 04RY47Z, 04UD0KZ, 04UD3JZ, 04UE3JZ, 04UF0JZ, 04UF37Z, 04UH0KZ, 04UH37Z, 04UH47Z, 04UH4KZ, 04UJ4KZ, 04UK07Z, 04UL07Z, 04UL47Z, 04UL4JZ, 04UM37Z, 04UM3JZ, 04UN0JZ, 04UP3JZ, 04UP3KZ, 04UP47Z, 04UP4KZ, 04UQ47Z, 04UR07Z, 04UR3JZ, 04UR4JZ, 04UT0JZ, 04UT37Z, 04UU4KZ, 04UV07Z, 04UW37Z, 04UW3JZ, 04UY4KZ, 04VC4CZ, 04VC4ZZ, 04VD3CZ, 04VD4CZ, 04VH0CZ, 04VH3CZ, 04VK0ZZ, 04VK4ZZ, 04VM4ZZ, 04VQ3CZ, 04VS0CZ, 04VT3CZ, 04VU4CZ, 04VV0ZZ, 04WY02Z, 04WY32Z, 04WY42Z, 04WY4DZ, 041009F, 041009J, 041009R, 04100JQ, 04100KD, 041049R, 04104A9, 04104AF, 04104AJ, 04104J8, 04104JG, 04104JH, 04104JR, 04104K6, 04104K7, 04104Z6, 041C0KH, 041C0ZK, 041C4AJ, 041C4JH, 041D09H, 041D0JK, 041D0ZJ, 041D0ZK, 041E4AK, 041E4KJ, 041F09K, 041F0JH, 041F0JJ, 041F0KK, 041F49J, 041H0KH, 041H4AJ, 041H4AK, 041H4KK, 041H4ZJ, 041J0AK, 041J0JH, 041J0JJ, 041J0KJ, 041J0ZK, 041J49K, 041J4AH, 041K09K, 041K09P, 041K09S, 041K0AJ, 041K0AN, 041K0JS, 041K0KL, 041K0KP, 041K0ZL, 041K0ZQ, 041K49J, 041K49P, 041K4KP, 041K4ZM, 041L09J, 041L09L, 041L09M, 041L09N, 041L09P, 041L0AL, 041L0AM, 041L0JH, 041L0ZK, 041L49H, 041L49S, 041L4AK, 041L4AS, 041L4JM, 041L4KH, 041L4ZL, 041L4ZP, 041M09L, 041M09P, 041M09Q, 041M0AL, 041M0AM, 041M0JM, 041M0ZM, 041M0ZQ, 041M4AM, 041M4AP, 041M4JP, 041M4JQ, 041M4ZP, 041N09L, 041N09M, 041N09P, 041N0AP, 041N0JP, 041N0JQ, 041N0KM, 041N4JL, 041N4KL, 041N4KM, 047004Z, 047035Z, 04703D6, 04703E6, 04703ZZ, 0470446, 0470466, 04704E6, 04704ZZ, 047C046, 047C04Z, 047C066, 047C076, 047C0ZZ, 047C356, 047C366, 047C3D6, 047C3F6, 047C3FZ, 047C3GZ, 047C3ZZ, 047C44Z, 047C47Z, 047D046, 047D0E6, 047D0FZ, 047D34Z, 047D3D6, 047D3GZ, 047D446, 047D466, 047D4D6, 047E0D6, 047E35Z, 047E3D6, 047E3Z6, 047E46Z, 047E476, 047E47Z, 047E4DZ, 047E4FZ, 047F0E6, 047F0F6, 047F34Z, 047F36Z, 047F3DZ, 047F3GZ, 047F3Z6, 047F3ZZ, 047F44Z, 047F456, 047F46Z, 047F4E6, 047H04Z, 047H07Z, 047H0DZ, 047H0E6, 047H3D6, 047H3Z6, 047H45Z, 047H4DZ, 047J046, 047J04Z, 047J076, 047J0D6, 047J0EZ, 047J0F6, 047J0GZ, 047J0ZZ, 047J35Z, 047J3EZ, 047J3F6, 047J44Z, 047J456, 047J476, 047J4FZ, 047J4GZ, 047J4ZZ, 047K076, 047K0D1, 047K36Z, 047K37Z, 047K3D1, 047K3G6, 047K456, 047K47Z, 047K4FZ, 047K4G6, 047K4GZ, 047K4Z1, 047K4Z6, 047L076, 047L0FZ, 047L0Z1, 047L37Z, 047L3FZ, 047L3GZ, 047L3ZZ, 047L4E6, 047L4EZ, 047L4Z1, 047M066, 047M076, 047M0Z1, 047M346, 047M356, 047M37Z, 047M3GZ, 047M44Z, 047M46Z, 047M476, 047M4DZ, 047M4E6, 047M4GZ, 047N041, 047N046, 047N04Z, 047N05Z, 047N066, 047N076, 047N0D1, 047N0EZ, 047N0F6, 047N0GZ, 047N0Z1,

© 2023 Lin KJ et al. *JAMA Network Open*.

	047N341, 047N34Z, 047N366, 047N3D1, 047N3FZ, 047N3G6, 047N3Z1, 047N46Z, 047N47Z, 047N4D6, 047N4F6, 047N4G6, 047N4Z6, 047N4ZZ, 047P056, 047P05Z, 047P0D6, 047P0EZ, 047P0G6, 047P0ZZ, 047P35Z, 047P3EZ, 047P456, 047P45Z, 047P4D6, 047P4EZ, 047P4FZ, 047Q046, 047Q056, 047Q0FZ, 047Q0GZ, 047Q0ZZ, 047Q346, 047Q34Z, 047Q366, 047Q466, 047Q4EZ, 047Q4F6, 047Q4FZ, 047R046, 047R056, 047R05Z, 047R06Z, 047R076, 047R0Z6, 047R0ZZ, 047R356, 047R36Z, 047R3ZZ, 047R476, 047R4E6, 047R4G6, 047R4GZ, 047S046, 047S06Z, 047S0D6, 047S0E6, 047S0FZ, 047S366, 047S3Z6, 047S3ZZ, 047S456, 047S466, 047S4DZ, 047S4E6, 047S4GZ, 047T04Z, 047T06Z, 047T076, 047T0DZ, 047T346, 047T366, 047T37Z, 047T3D6, 047T3EZ, 047T3FZ, 047T3G6, 047T466, 047T4D6, 047T4EZ, 047T4FZ, 047U066, 047U0D6, 047U346, 047U366, 047U3DZ, 047U3E6, 047U3F6, 047U44Z, 047U456, 047U4F6, 047V046, 047V04Z, 047V066, 047V0DZ, 047V356, 047V366, 047V3GZ, 047V456, 047V4EZ, 047V4F6, 047V4GZ, 047V4ZZ, 047W046, 047W066, 047W376, 047W37Z, 047W3D6, 047W3G6, 047W3Z6, 047W3ZZ, 047W44Z, 047W46Z, 047W47Z, 047W4G6, 047Y0DZ, 047Y346, 047Y3EZ, 047Y3F6, 047Y44Z, 047Y4D6, 047Y4F6, 04BK0ZZ, 04BV4ZZ, 04BW0ZZ, 04BY0ZZ, 04CK0ZZ, 04CK4ZZ, 04CL3ZZ, 04CM0ZZ, 04CM4ZZ, 04CP0ZZ, 04CP3ZZ, 04CT0ZZ, 04CU0ZZ, 04CU3Z6, 04CV4ZZ, 04CW0Z6, 04CW0ZZ, 04CW4Z6, 04HC3DZ, 04HF3DZ, 04HH3DZ, 04HJ4DZ, 04HN4DZ, 04HS3DZ, 04HT3DZ, 04HU4DZ, 04HV4DZ, 04HW0DZ, 04HY3DZ, 04HY42Z, 04ND0ZZ, 04NE3ZZ, 04NF4ZZ, 04NH3ZZ, 04NP0ZZ, 04NP3ZZ, 04NU0ZZ, 04NW4ZZ, 04QY3ZZ, 04RK07Z, 04RK4KZ, 04RL0JZ, 04RM0JZ, 04RN4JZ, 04RP4JZ, 04RP4KZ, 04RR0KZ, 04RR47Z, 04RR4KZ, 04RS07Z, 04RS0JZ, 04RS4JZ, 04RT07Z, 04RT4JZ, 04RU4JZ, 04RV07Z, 04RY07Z, 04RY4KZ, 04UC0KZ, 04UD3KZ, 04UD4JZ, 04UE3KZ, 04UE4JZ, 04UH3KZ, 04UJ3JZ, 04UJ47Z, 04UK37Z, 04UK3KZ, 04UK4KZ, 04UL3JZ, 04UL3KZ, 04UM07Z, 04UM4KZ, 04UN4JZ, 04UN4KZ, 04UP37Z, 04UP4JZ, 04UQ4JZ, 04UR0KZ, 04UR37Z, 04UR4KZ, 04US3JZ, 04UT3KZ, 04UT4KZ, 04UU07Z, 04UU3KZ, 04UV0JZ, 04UV3KZ, 04UV4KZ, 04UW0KZ, 04UY07Z, 04UY37Z, 04VC0CZ, 04VD0CZ, 04VE3CZ, 04VF0ZZ, 04VJ0ZZ, 04VK3CZ, 04VL3CZ, 04VM0CZ, 04VM3CZ, 04VN3CZ, 04VN4CZ, 04VP3CZ, 04VP4ZZ, 04VQ0ZZ, 04VQ4CZ, 04VR0CZ, 04VR4CZ, 04VR4ZZ, 04VS0ZZ, 04VS4CZ, 04VU0CZ, 04VU0ZZ, 04VU3CZ, 04VV4ZZ, 04VY3CZ, 04VY4CZ, 04WY00Z, 04WY3CZ, 04WY40Z, 04WY43Z, 04100A6, 04100AB, 04100AD, 04100AQ, 04100AR, 04100J7, 04100J8, 04100J9, 04100JF, 04100K6, 04100K7, 04100K8, 04100KQ, 04100Z9, 04100ZF, 04100ZK, 0410496, 0410499, 041049F, 041049Q, 04104AD, 04104AK, 04104J7, 04104JC, 04104JQ, 04104KH, 04104KK, 04104KR, 04104Z8, 04104ZJ, 04104ZR, 041C09J, 041C0AH, 041C4AK, 041C4JK, 041C4KH, 041C4KK, 041C4ZH, 041C4ZJ, 041D0AJ, 041D0JH, 041D0KK, 041D49J, 041D4AJ, 041D4KJ, 041E09H, 041E09J, 041E0JJ, 041E4AJ, 041E4KH, 041E4ZH, 041E4ZJ, 041F09J, 041F0KJ, 041F0ZH, 041F0ZK, 041F4KK, 041F4ZH, 041H0KJ, 041H0KK, 041H0ZJ, 041H49J, 041H4JH, 041H4KH, 041J0AH, 041J0JK, 041J49J, 041J4KH, 041J4KJ, 041K09Q, 041K0JM, 041K0JP, 041K0JQ, 041K0KJ, 041K0KN, 041K49K, 041K49L, 041K49M, 041K4AM, 041K4JJ, 041K4JL, 041K4JN, 041K4ZJ, 041L09H, 041L09S, 041L0AJ, 041L0AS, 041L0JJ, 041L0JL, 041L0JM, 041L0JN, 041L0JP, 041L0KQ, 041L0KS, 041L0ZL, 041L0ZN, 041L49L, 041L49N, 041L4AJ, 041L4AN, 041L4AP, 041L4JJ, 041L4JK, 041L4JN, 041L4JQ, 041L4KL, 041L4KN, 041L4KP, 041L4KQ, 041L4ZM, 041L4ZN, 041M0AP, 041M0AS, 041M0JL, 041M0JP, 041M0KQ, 041M0ZL, 041M49M, 041M49Q, 041M49S, 041M4AQ, 041M4JL, 041M4KQ, 041M4ZL, 041N09Q, 041N0KP, 041N0KQ, 041N0KS, 041N4AL, 041N4AP, 041N4ZQ, 047006Z, 0470076, 04700D6, 04700DZ, 04700E6, 04700F6, 04700GZ, 04700ZZ, 0470366, 04703G6, 047046Z, 0470476, 04704D6, 04704DZ, 04704EZ, 04704G6, 047C06Z, 047C0GZ, 047C0Z6, 047C346, 047C34Z, 047C3DZ, 047C456, 047D05Z, 047D0D6, 047D346, 047D35Z, 047D3F6, 047D3FZ, 047D3G6, 047D3ZZ, 047D456, 047D47Z, 047D4DZ, 047D4EZ, 047D4Z6, 047E046, 047E05Z, 047E06Z, 047E076, 047E0E6, 047E0G6, 047E0GZ, 047E0ZZ, 047E376, 047E3G6, 047E3GZ, 047E3ZZ, 047E446, 047E456, 047E45Z, 047E4Z6, 047F056, 047F05Z, 047F0D6, 047F0EZ, 047F0FZ, 047F0GZ, 047F3D6, 047F3E6, 047F3EZ, 047F45Z, 047F47Z, 047F4D6, 047H076, 047H0EZ, 047H0F6, 047H35Z, 047H37Z, 047H3EZ, 047H446, 047H46Z, 047H47Z, 047H4D6, 047H4E6, 047H4FZ, 047J056, 047J05Z, 047J0G6, 047J376, 047J3DZ, 047J3E6, 047J3Z6, 047J47Z, 047J4D6, 047J4DZ, 047J4EZ, 047J4F6, 047J4Z6, 047K041, 047K05Z, 047K07Z, 047K366, 047K3EZ, 047K3ZZ, 047K441, 047K44Z, 047L05Z, 047L066, 047L06Z, 047L0DZ, 047L0EZ, 047L3D6, 047L44Z, 047L466, 047L46Z, 047L47Z, 047L4D6, 047L4Z6, 047M041, 047M04Z, 047M07Z, 047M0G6, 047M341, 047M36Z, 047M3D1, 047M3E6, 047M456, 047M45Z, 047M4ZZ, 047N0DZ, 047N0E6, 047N0FZ, 047N0G6, 047N356, 047N35Z, 047N376, 047N37Z, 047N3EZ, 047N44Z, 047N4FZ, 047P06Z, 047P0FZ, 047P0Z6, 047P346,

© 2023 Lin KJ et al. *JAMA Network Open*.

	047P37Z, 047P3ZZ, 047P46Z, 047P4Z6, 047Q05Z, 047Q0D6, 047Q0DZ, 047Q3D6, 047Q3DZ, 047Q3E6, 047Q446, 047Q44Z, 047Q456, 047Q476, 047Q4DZ, 047Q4G6, 047R07Z, 047R0DZ, 047R0FZ, 047R35Z, 047R3F6, 047R3Z6, 047R44Z, 047R45Z, 047R4F6, 047R4Z6, 047S04Z, 047S056, 047S05Z, 047S066, 047S0GZ, 047S0Z6, 047S346, 047S446, 047S44Z, 047S46Z, 047S4D6, 047S4EZ, 047S4F6, 047S4G6, 047T0FZ, 047T0GZ, 047T34Z, 047T35Z, 047T3E6, 047T3GZ, 047T446, 047T4DZ, 047T4GZ, 047U04Z, 047U05Z, 047U076, 047U07Z, 047U0G6, 047U0GZ, 047U356, 047U36Z, 047U37Z, 047U3FZ, 047U476, 047U4GZ, 047V05Z, 047V06Z, 047V0E6, 047V36Z, 047V3D6, 047V3F6, 047V3FZ, 047V3ZZ, 047V446, 047V4D6, 047V4E6, 047V4Z6, 047W076, 047W0G6, 047W34Z, 047W3E6, 047W3FZ, 047W446, 047W456, 047W45Z, 047W4DZ, 047W4FZ, 047W4ZZ, 047Y066, 047Y0D6, 047Y0EZ, 047Y35Z, 047Y3DZ, 047Y3ZZ, 047Y456, 047Y46Z, 047Y4Z6, 04BN0ZZ, 04BP0ZZ, 04BV0ZZ, 04BW4ZZ, 04CK4Z6, 04CL0ZZ, 04CM3ZZ, 04CN4ZZ, 04CQ4Z6, 04CR4ZZ, 04CT3Z6, 04CT3ZZ, 04CT4Z6, 04CU4Z6, 04CV3Z6, 04CW4ZZ, 04CY3Z6, 04CY3ZZ, 04CY4ZZ, 04HD3DZ, 04HD4DZ, 04HE3DZ, 04HJ0DZ, 04HL0DZ, 04HM3DZ, 04HP3DZ, 04HQ0DZ, 04HR3DZ, 04HR4DZ, 04HU0DZ, 04HV0DZ, 04HW3DZ, 04HY4DZ, 04ND4ZZ, 04NE4ZZ, 04NF3ZZ, 04NH0ZZ, 04NH4ZZ, 04NJ0ZZ, 04NK3ZZ, 04NL0ZZ, 04NL4ZZ, 04NM4ZZ, 04NS4ZZ, 04NT0ZZ, 04NT3ZZ, 04NU4ZZ, 04NV0ZZ, 04NV4ZZ, 04NY0ZZ, 04QY0ZZ, 04RS0KZ, 04RS47Z, 04RU0JZ, 04RU0KZ, 04RV47Z, 04RV4JZ, 04RV4KZ, 04RW0KZ, 04RW4KZ, 04RY4JZ, 04UC07Z, 04UC0JZ, 04UC37Z, 04UC3JZ, 04UC4JZ, 04UD07Z, 04UE37Z, 04UE4KZ, 04UF07Z, 04UF4JZ, 04UH0JZ, 04UH3JZ, 04UH4JZ, 04UJ0KZ, 04UJ37Z, 04UK0JZ, 04UK0KZ, 04UL37Z, 04UM0JZ, 04UM3KZ, 04UN37Z, 04UN3JZ, 04UP0JZ, 04US0JZ, 04US4JZ, 04UT07Z, 04UV0KZ, 04UV47Z, 04UW07Z, 04UW47Z, 04UW4JZ, 04UY0KZ, 04UY47Z, 04UY4JZ, 04VE4ZZ, 04VF0CZ, 04VF4ZZ, 04VH4ZZ, 04VJ3CZ, 04VJ4CZ, 04VL0CZ, 04VL4ZZ, 04VM4CZ, 04VN0CZ, 04VN0ZZ, 04VN4ZZ, 04VP4CZ, 04VR0ZZ, 04VR3CZ, 04VU4ZZ, 04VW4ZZ, 04WY03Z, 0410096, 041009D, 041009G, 041009Q, 04100A7, 04100A9, 04100AH, 04100AK, 04100JB, 04100JC, 04100JG, 04100JK, 04100JR, 04100K9, 04100KC, 04100ZG, 04100ZQ, 0410498, 041049G, 041049K, 04104A6, 04104AC, 04104AG, 04104AH, 04104AQ, 04104KD, 04104KF, 04104KG, 04104KJ, 04104KQ, 04104ZF, 04104ZG, 04104ZH, 041C0KJ, 041C4ZK, 041D09K, 041D4JH, 041D4JJ, 041D4KH, 041D4ZJ, 041D4ZK, 041E09K, 041E0AH, 041E0AJ, 041E0JH, 041E0KH, 041E0KK, 041E0ZK, 041E4AH, 041F09H, 041F0JK, 041F4AH, 041F4AK, 041F4KJ, 041H09J, 041H0JH, 041H0ZH, 041H49H, 041H4ZK, 041J09K, 041J0KH, 041J0KK, 041J0ZH, 041J4AK, 041J4JH, 041J4ZH, 041J4ZJ, 041J4ZK, 041K0AM, 041K0AS, 041K0KH, 041K0KS, 041K0ZJ, 041K0ZP, 041K49Q, 041K49S, 041K4AK, 041K4JH, 041K4JK, 041K4JM, 041K4JP, 041K4JS, 041K4KJ, 041K4KK, 041K4KL, 041K4KM, 041K4KS, 041K4ZK, 041K4ZN, 041K4ZP, 041K4ZS, 041L09Q, 041L0AP, 041L0AQ, 041L0JQ, 041L0KM, 041L0ZQ, 041L49P, 041L4JH, 041L4JL, 041L4KJ, 041L4ZH, 041L4ZJ, 041L4ZK, 041M09M, 041M0AQ, 041M0JQ, 041M0KL, 041M0KM, 041M0KP, 041M0KS, 041M0ZP, 041M49L, 041M49P, 041M4KS, 041N0AQ, 041N0KL, 041N0ZM, 041N0ZS, 041N4AQ, 041N4AS, 041N4JP, 041N4JQ, 041N4JS, 041N4KQ, 047005Z, 04700G6, 047037Z, 04703F6, 04703FZ, 047047Z, 047C0DZ, 047C0E6, 047C0F6, 047C0FZ, 047C0G6, 047C35Z, 047C36Z, 047C37Z, 047C3Z6, 047C466, 047C476, 047C4F6, 047C4G6, 047D056, 047D066, 047D06Z, 047D076, 047D07Z, 047D0DZ, 047D0EZ, 047D356, 047D366, 047D36Z, 047D37Z, 047D476, 047D4FZ, 047D4GZ, 047E04Z, 047E056, 047E066, 047E0DZ, 047E3F6, 047E4D6, 047E4G6, 047F04Z, 047F076, 047F0ZZ, 047F346, 047F37Z, 047F3F6, 047F3G6, 047F466, 047F4DZ, 047F4FZ, 047F4GZ, 047H0D6, 047H0FZ, 047H0G6, 047H346, 047H34Z, 047H366, 047H3F6, 047H3ZZ, 047H44Z, 047H456, 047H476, 047H4F6, 047H4GZ, 047J0E6, 047J46Z, 047J4E6, 047J4G6, 047K046, 047K0D6, 047K0EZ, 047K0FZ, 047K34Z, 047K356, 047K3F6, 047K466, 047K4D6, 047L07Z, 047L0F6, 047L0G6, 047L0ZZ, 047L35Z, 047L366, 047L3D1, 047L3DZ, 047L3EZ, 047L3G6, 047L456, 047L4F6, 047L4G6, 047M056, 047M06Z, 047M0DZ, 047M0F6, 047M0ZZ, 047M3DZ, 047M3F6, 047M446, 047M47Z, 047M4D1, 047M4D6, 047M4FZ, 047N07Z, 047N0D6, 047N3D6, 047N3DZ, 047N456, 047N466, 047N4EZ, 047N4GZ, 047P046, 047P0E6, 047P0GZ, 047P34Z, 047P376, 047P3D6, 047P3F6, 047P446, 047P466, 047P47Z, 047P4E6, 047P4F6, 047Q04Z, 047Q07Z, 047Q0E6, 047Q0F6, 047Q376, 047Q3F6, 047Q3GZ, 047Q3Z6, 047Q3ZZ, 047Q45Z, 047Q46Z, 047Q4E6, 047R04Z, 047R0D6, 047R366, 047R37Z, 047R3DZ, 047R3EZ, 047R466, 047R46Z, 047R47Z, 047R4DZ, 047S0F6, 047S36Z, 047S3DZ, 047S3EZ, 047S3GZ, 047S4FZ, 047S4ZZ, 047T05Z, 047T0E6, 047T376, 047T3F6, 047T44Z, 047T45Z, 047T46Z, 047T47Z, 047T4F6, 047T4G6, 047T4ZZ, 047U06Z, 047U0DZ, 047U0EZ, 047U0F6, 047U0Z6, 047U446, 047U46Z, 047U4EZ, 047U4ZZ, 047V056, 047V0D6, 047V0EZ, 047V0FZ, 047V0ZZ, 047V346,

© 2023 Lin KJ et al. *JAMA Network Open*.

	047V35Z, 047V37Z, 047V466, 047V47Z, 047V4DZ, 047W04Z, 047W056, 047W0D6, 047W0FZ, 047W0Z6, 047W0ZZ, 047W356, 047W35Z, 047W366, 047W3F6, 047W3GZ, 047W466, 047W4Z6, 047Y0E6, 047Y0F6, 047Y0FZ, 047Y0ZZ, 047Y36Z, 047Y376, 047Y3FZ, 047Y3GZ, 047Y446, 047Y45Z, 047Y466, 047Y4DZ, 047Y4E6, 047Y4GZ, 047Y4ZZ, 04BL4ZZ, 04BN4ZZ, 04BR0ZZ, 04BS0ZZ, 04BT4ZZ, 04CL0Z6, 04CL4Z6, 04CL4ZZ, 04CM4Z6, 04CN0Z6, 04CP3Z6, 04CP4ZZ, 04CQ0Z6, 04CQ4ZZ, 04CR0Z6, 04CS0ZZ, 04CV0ZZ, 04CY0ZZ, 04HC4DZ, 04HE4DZ, 04HF4DZ, 04HL3DZ, 04HN0DZ, 04HP0DZ, 04HQ3DZ, 04HS0DZ, 04HS4DZ, 04HT0DZ, 04HV3DZ, 04HW4DZ, 04HY02Z, 04NC0ZZ, 04NC3ZZ, 04NC4ZZ, 04NN0ZZ, 04NQ0ZZ, 04NR0ZZ, 04NS3ZZ, 04NT4ZZ, 04NV3ZZ, 04NW3ZZ, 04RK47Z, 04RK4JZ, 04RM07Z, 04RM0KZ, 04RM4KZ, 04RP0KZ, 04RQ0JZ, 04RQ0KZ, 04RT0JZ, 04RU07Z, 04RV0KZ, 04UC47Z, 04UC4KZ, 04UD0JZ, 04UD37Z, 04UD47Z, 04UE47Z, 04UF4KZ, 04UH07Z, 04UJ07Z, 04UJ0JZ, 04UJ3KZ, 04UK3JZ, 04UK4JZ, 04UL4KZ, 04UM4JZ, 04UN07Z, 04UN0KZ, 04UN47Z, 04UP07Z, 04UQ0JZ, 04UQ0KZ, 04UQ37Z, 04US07Z, 04US37Z, 04US4KZ, 04UT3JZ, 04UT4JZ, 04UU4JZ, 04UV37Z, 04UV3JZ, 04UW4KZ, 04UY0JZ, 04UY3JZ, 04UY3KZ, 04VC0ZZ, 04VC3CZ, 04VE0CZ, 04VF3CZ, 04VF4CZ, 04VH0ZZ, 04VH4CZ, 04VK0CZ, 04VL0ZZ, 04VL4CZ, 04VM0ZZ, 04VP0ZZ, 04VQ0CZ, 04VS4ZZ, 04VT0CZ, 04VT4ZZ, 04VV0CZ, 04VW0CZ, 04VW3CZ, 04VW4CZ, 04VY0ZZ, 04VY4ZZ, 04WY0CZ, 04WY3DZ, 04WY4CZ, 0410097, 0410099, 041009C, 041009H, 04100AC, 04100AF, 04100AJ, 04100JD, 04100KF, 04100KG, 04100KH, 04100KK, 04100KR, 04100Z6, 04100ZB, 04100ZH, 04100ZR, 041049B, 04104AB, 04104J9, 04104JD, 04104JF, 04104JJ, 04104K9, 04104KB, 04104KC, 04104Z7, 04104ZB, 04104ZC, 04104ZQ, 041C09H, 041C09K, 041C0AJ, 041C0AK, 041C0JJ, 041C0JK, 041C0ZH, 041C0ZJ, 041C49H, 041C49J, 041C4JJ, 041D0AH, 041D0KH, 041D0ZH, 041D49K, 041D4AH, 041D4JK, 041D4KK, 041D4ZH, 041E0AK, 041E0JK, 041E0ZJ, 041E49H, 041E49J, 041E4KK, 041F0AJ, 041F0KH, 041F0ZJ, 041F49K, 041F4JJ, 041F4JK, 041H09K, 041H0AH, 041H0AK, 041H0JJ, 041H49K, 041H4KJ, 041J49H, 041J4AJ, 041J4JJ, 041J4JK, 041K09L, 041K09N, 041K0AL, 041K0AP, 041K0JH, 041K0JL, 041K0ZK, 041K0ZM, 041K0ZN, 041K0ZS, 041K4AJ, 041K4AL, 041K4JQ, 041K4KN, 041K4KQ, 041K4ZQ, 041L09K, 041L0AK, 041L0AN, 041L0JK, 041L0KJ, 041L0KL, 041L0KN, 041L0ZH, 041L0ZJ, 041L0ZP, 041L0ZS, 041L49J, 041L49M, 041L49Q, 041L4AH, 041L4JS, 041L4KM, 041L4ZS, 041M09S, 041M0JS, 041M4AL, 041M4JM, 041M4JS, 041M4KL, 041M4KM, 041M4KP, 041M4ZM, 041N0AL, 041N0JL, 041N0JM, 041N0JS, 041N0ZL, 041N0ZP, 041N0ZQ, 041N49L, 041N49M, 041N49Q, 041N49S, 041N4KS, 041N4ZL, 041N4ZP, 041N4ZS, 04700EZ, 04700FZ, 04700Z6, 047034Z, 0470356, 047036Z, 0470376, 04703DZ, 04703EZ, 04703Z6, 047045Z, 04704Z6, 047C056, 047C0D6, 047C0EZ, 047C446, 047C45Z, 047C46Z, 047C4FZ, 047D04Z, 047D0GZ, 047D0Z6, 047D0ZZ, 047D376, 047D3E6, 047D3Z6, 047D44Z, 047D45Z, 047D46Z, 047D4F6, 047D4ZZ, 047E34Z, 047E356, 047E366, 047E36Z, 047E3DZ, 047E4E6, 047F046, 047F06Z, 047F356, 047F366, 047F3FZ, 047F4EZ, 047F4F6, 047F4G6, 047H056, 047H066, 047H0GZ, 047H356, 047H36Z, 047H376, 047H3FZ, 047H3G6, 047H3GZ, 047H4EZ, 047H4ZZ, 047J07Z, 047J0Z6, 047J346, 047J356, 047J366, 047J36Z, 047J37Z, 047J3D6, 047J446, 047J45Z, 047K04Z, 047K066, 047K0DZ, 047K0F6, 047K0GZ, 047K346, 047K35Z, 047K3DZ, 047K3GZ, 047K3Z1, 047K446, 047K4ZZ, 047L046, 047L04Z, 047L056, 047L0D6, 047L346, 047L356, 047L376, 047L3E6, 047L3F6, 047L3Z6, 047L441, 047L45Z, 047L476, 047L4DZ, 047L4FZ, 047M046, 047M0D6, 047M0E6, 047M0FZ, 047M0GZ, 047M0Z6, 047M34Z, 047M366, 047M376, 047M3EZ, 047M3FZ, 047M3G6, 047M441, 047M4F6, 047N06Z, 047N0Z6, 047N0ZZ, 047N3F6, 047N3ZZ, 047N441, 047N446, 047N476, 047N4D1, 047P0DZ, 047P0F6, 047P356, 047P366, 047P3FZ, 047P3Z6, 047P44Z, 047P4GZ, 047Q076, 047Q0EZ, 047Q0G6, 047Q0Z6, 047Q356, 047Q35Z, 047Q36Z, 047Q37Z, 047Q3EZ, 047Q3G6, 047Q4GZ, 047Q4ZZ, 047R0E6, 047R0G6, 047R346, 047R34Z, 047R3E6, 047R3FZ, 047R4D6, 047R4EZ, 047R4FZ, 047S0DZ, 047S0ZZ, 047S376, 047S3E6, 047S3F6, 047S3G6, 047S45Z, 047T056, 047T066, 047T07Z, 047T0F6, 047T0ZZ, 047T36Z, 047T3Z6, 047T3ZZ, 047T4Z6, 047U046, 047U0ZZ, 047U35Z, 047U3G6, 047U3GZ, 047U3Z6, 047U3ZZ, 047U45Z, 047U466, 047U4D6, 047U4DZ, 047U4E6, 047U4G6, 047U4Z6, 047V0G6, 047V0GZ, 047V376, 047V3DZ, 047V3G6, 047V3Z6, 047V45Z, 047V4G6, 047W05Z, 047W07Z, 047W0E6, 047W346, 047W3DZ, 047W476, 047W4E6, 047W4EZ, 047W4F6, 047Y04Z, 047Y056, 047Y05Z, 047Y076, 047Y07Z, 047Y0G6, 047Y0GZ, 047Y0Z6, 047Y34Z, 047Y366, 047Y3G6, 047Y3Z6, 047Y4FZ, 047Y4G6, 04BK4ZZ, 04BL0ZZ, 04BM4ZZ, 04BQ4ZZ, 04BR4ZZ, 04BT0ZZ, 04BU0ZZ, 04CK0Z6, 04CK3Z6, 04CK3ZZ, 04CL3Z6, 04CM3Z6, 04CN0ZZ, 04CN3ZZ, 04CN4Z6, 04CP0Z6, 04CQ3ZZ, 04CR0ZZ, 04CR4Z6, 04CS0Z6, 04CS4Z6, 04CU0Z6, 04CU3ZZ, 04CV0Z6, 04CW3Z6, 04CY0Z6, 04HD0DZ, 04HE0DZ, 04HJ3DZ, 04HL4DZ,

© 2023 Lin KJ et al. *JAMA Network Open*.

	04HM0DZ, 04HM4DZ, 04HP4DZ, 04HR0DZ, 04NE0ZZ, 04NF0ZZ, 04NJ3ZZ, 04NJ4ZZ, 04NL3ZZ, 04NM3ZZ, 04NN3ZZ, 04NN4ZZ, 04NP4ZZ, 04NQ3ZZ, 04NW0ZZ, 04NY3ZZ, 04NY4ZZ, 04QY4ZZ, 04RL07Z, 04RL47Z, 04RN07Z, 04RN0JZ, 04RN0KZ, 04RP07Z, 04RP0JZ, 04RQ07Z, 04RQ4KZ, 04RT0KZ, 04RT47Z, 04RU47Z, 04RW07Z, 04RW0JZ, 04RY0KZ, 04UC3KZ, 04UD4KZ, 04UE07Z, 04UE0JZ, 04UE0KZ, 04UF0KZ, 04UF3JZ, 04UF3KZ, 04UF47Z, 04UJ4JZ, 04UK47Z, 04UL0JZ, 04UL0KZ, 04UM0KZ, 04UM47Z, 04UN3KZ, 04UP0KZ, 04UQ07Z, 04UQ3JZ, 04UQ3KZ, 04UQ4KZ, 04UR0JZ, 04UR3KZ, 04UR47Z, 04US0KZ, 04US3KZ, 04US47Z, 04UT0KZ, 04UT47Z, 04UU0JZ, 04UU0KZ, 04UU37Z, 04UU3JZ, 04UU47Z, 04UV4JZ, 04UW0JZ, 04UW3KZ, 04VD0ZZ, 04VD4ZZ, 04VE0ZZ, 04VE4CZ, 04VJ0CZ, 04VJ4ZZ, 04VK4CZ, 04VP0CZ, 04VQ4ZZ, 04VS3CZ, 04VT0ZZ, 04VT4CZ, 04VV3CZ, 04VV4CZ, 04VW0ZZ, 04VY0CZ, 04WY0DZ, 04WY30Z, 04WY33Z<br>ICD-9 Diagnosis Code (any position) is any of: 440.20, 440.23, 440.31, 440.32, 440.22, 440.30, 443.9, 440.29, 440.4, 440.24, 440.21<br>ICD-10 Diagnosis Code is any of: I70.219, I70.231, I70.232, I70.233, I70.242, I70.244, I70.25, I70.268, I70.269, I70.291, I70.299, I70.303, I70.311, I70.313, I70.343, I70.345, I70.398, I70.401, I70.444, I70.492, I70.499, I70.502, I70.508, I70.513, I70.522, I70.531, I70.532, I70.535, I70.542, I70.545, I70.55, I70.568, I70.592, I70.608, I70.621, I70.628, I70.631, I70.641, I70.642, I70.649, I70.663, I70.668, I70.735, I70.761, I70.762, I70.793, I73.9, I70.211, I70.212, I70.222, I70.223, I70.234, I70.262, I70.292, I70.298, I70.334, I70.338, I70.342, I70.362, I70.368, I70.403, I70.418, I70.432, I70.442, I70.445, I70.462, I70.469, I70.503, I70.511, I70.529, I70.538, I70.544, I70.548, I70.549, I70.591, I70.599, I70.633, I70.634, I70.639, I70.661, I70.691, I70.692, I70.699, I70.711, I70.712, I70.718, I70.719, I70.742, I70.743, I70.744, I70.748, I70.791, I70.203, I70.208, I70.213, I70.229, I70.239, I70.245, I70.261, I70.301, I70.302, I70.318, I70.321, I70.323, I70.328, I70.329, I70.332, I70.333, I70.341, I70.344, I70.348, I70.35, I70.361, I70.391, I70.393, I70.411, I70.412, I70.413, I70.419, I70.422, I70.433, I70.443, I70.461, I70.512, I70.518, I70.519, I70.521, I70.523, I70.528, I70.543, I70.561, I70.563, I70.598, I70.602, I70.611, I70.618, I70.619, I70.635, I70.644, I70.645, I70.648, I70.662, I70.669, I70.702, I70.708, I70.713, I70.728, I70.731, I70.733, I70.738, I70.739, I70.745, I70.749, I70.799, I70.201, I70.209, I70.218, I70.221, I70.235, I70.241, I70.243, I70.293, I70.308, I70.309, I70.312, I70.319, I70.322, I70.331, I70.339, I70.349, I70.363, I70.369, I70.408, I70.409, I70.421, I70.428, I70.434, I70.438, I70.441, I70.448, I70.449, I70.45, I70.491, I70.493, I70.509, I70.533, I70.534, I70.562, I70.593, I70.603, I70.612, I70.623, I70.643, I70.65, I70.693, I70.701, I70.721, I70.729, I70.741, I70.75, I70.769, I70.792, I70.92, I70.202, I70.228, I70.238, I70.248, I70.249, I70.263, I70.335, I70.392, I70.399, I70.402, I70.423, I70.429, I70.431, I70.435, I70.439, I70.463, I70.468, I70.498, I70.501, I70.539, I70.541, I70.569, I70.601, I70.609, I70.613, I70.622, I70.629, I70.632, I70.638, I70.698, I70.703, I70.709, I70.722, I70.723, I70.732, I70.734, I70.763, I70.768, I70.798
Smoking	ICD-9 Diagnosis Code (any position) is any of: 989.84, 305.1, 649.0, 649.00, 649.04, 649.01, 649.02, 649.03, V15.82<br>ICD-10 Diagnosis Code (any position) is any of: F17.200, O99.332, T65.221A, T65.292A, F17.221, F17.291, F17.201, O99.331, O99.333, O99.334, T65.212A, T65.213A, T65.214A, T65.222A, T65.223A, T65.293A, T65.294A, F17.210, F17.211, F17.220, F17.290, O99.335, T65.211A, T65.224A, T65.291A, Z87.891, O99.330<br>HCPCS Procedure Code is any of: 1034F, 4001F, 99406, G9016, G9458, S4995, S9453, 4004F, 99407, G9276, G0437, G0436, S9075<br>NDC Generic Name is any of: NICOTINE BITARTRATE, VARENICLINE TARTRATE, NICOTINE, NICOTINE POLACRILEX
Stroke	ICD-9 Diagnosis Code (any position) is any of: 433.00, 433.20, 433.30, 433.90, 434.1, 434.9, 434.91, 438.19, 438.30, 438.40, 438.41, 438.51, 438.7, 438.85, 433.01, 433.1, 433.11, 433.3, 434.00, 434.10, 438.11, 438.21, 438.50, 438.6, 438.89, 433.2, 433.8, 433.80, 433.9, 436, 437.1, 438.1, 438.12, 438.13, 438.22, 438.31, 438.32, 438.42, 438.53, 438.82, 438.83, 433.10, 433.31, 433.81, 434.90, 438.10, 438.2, 438.20, 438.5, 438.52, 438.8, 438.81, 438.84, 431, 433.0, 433.21, 433.91, 434.0, 434.01, 434.11, 438.0, 438.14, 438.3, 438.4, 438.9<br>ICD-10 Diagnosis Code (any position) is any of: G45.9, G46.6, I63.013, I63.031, I63.033, I63.232, I63.323, I63.331, I63.342, I63.349, I63.50, I63.513, I63.521, I63.522, I63.523, I63.529, I63.541, I63.8, I65.21, I65.23, I66.02, I67.1, I67.7, G46.2, I63.00, I63.012, I63.12, I63.211, I63.213, I63.219, I63.239, I63.29, I63.313, I63.319, I63.329, I63.341, I63.39, I63.411, I63.413, I63.419, I63.432, I63.433, I63.449, I63.511, I63.532, I63.543, I63.549, I63.59, I65.9, I66.03, I66.22, I66.29, I66.9, I67.4, I67.89, I67.9, I68.8, G45.4, G45.8, G46.7, I63.019, I63.032, I63.112, I63.113, I63.131, I63.20, I63.233, I63.322, I63.333, I63.339, I63.40, I63.421, I63.422, I63.429, I63.431, I63.442, I63.443, I63.533, I63.539, I63.6,

© 2023 Lin KJ et al. *JAMA Network Open*.

	I63.9, I65.01, I65.02, I65.03, I65.1, I65.29, I65.8, I66.09, I66.12, I66.23, I66.3, I66.8, I67.2, I67.5, I67.6, I67.848, G45.0, G45.1, G45.2, G46.4, G46.5, G46.8, I63.02, I63.111, I63.133, I63.139, I63.19, I63.212, I63.231, I63.30, I63.312, I63.332, I63.343, I63.49, I63.512, I63.531, I63.542, I66.19, I66.21, I67.81, I68.2, G46.0, G46.1, G46.3, I63.011, I63.039, I63.09, I63.10, I63.119, I63.132, I63.22, I63.311, I63.321, I63.412, I63.423, I63.439, I63.441, I63.519, I65.09, I65.22, I66.01, I66.11, I66.13, I67.82, I67.841, I68.0
Syncope	ICD-9 Diagnosis Code (any position) is any of: 780.2, 992.1<br>ICD-10 Diagnosis Code (any position) is any of: R55
ACE inhibitors	NDC Generic Name is any of: CAPTOPRIL, LISINOPRIL, RAMIPRIL, TRANDOLAPRIL
ARBs	NDC Generic Name is any of: IRBESARTAN, TELMISARTAN, VALSARTAN
Antiarrhythmic agents	NDC Generic Name is any of: AMIODARONE HCL, DOFETILIDE, DRONEDARONE HCL, FLECAINIDE ACETATE, IBUTILIDE FUMARATE, MEXILETINE HCL, MORICIZINE HCL, PROCAINAMIDE HCL, PROPAFENONE HCL, QUINIDINE HCL, TOCAINIDE HCL
Injectable Anticoagulants	NDC Generic Name is any of: DALTEPARIN SODIUM,PORCINE, ENOXAPARIN SODIUM, FONDAPARINUX SODIUM
Antiplatelet agents	NDC Generic Name is any of: ASPIRIN, TICAGRELOR, CLOPIDOGREL BISULFATE, ASPIRIN (CALCIUM CARB & MAGNESIUM BUFFERS)/PRAVASTATIN, ASPIRIN/ACETAMINOPHEN, ASPIRIN/ACETAMINOPHEN/CAFFEINE, ASPIRIN/ACETAMINOPHEN/CAFFEINE/CALCIUM, ASPIRIN/ACETAMINOPHEN/CAFFEINE/POTASSIUM, ASPIRIN/ACETAMINOPHEN/CALCIUM CARBONATE, ASPIRIN/ACETAMINOPHEN/MAGNESIUM/ALUMINUM HYDROXIDE/CAFFEINE, ASPIRIN/CAFFEINE, ASPIRIN/CALCIUM CARBONATE, ASPIRIN/CALCIUM CARBONATE/MAGNESIUM, ASPIRIN/CALCIUM CARBONATE/MAGNESIUM/ALUMINUM HYDROXIDE, ASPIRIN/CODEINE PHOSPHATE, ASPIRIN/DIPHENHYDRAMINE CITRATE, ASPIRIN/DIPHENHYDRAMINE HCL, ASPIRIN/DIPHENHYDRAMINE/SODIUM BICARBONATE/CITRIC ACID, ASPIRIN/DIPYRIDAMOLE, ASPIRIN/MAGNESIUM CARBONATE/DIHYDROXYALUMINUM AMINOACETATE, ASPIRIN/MAGNESIUM HYDROXIDE/ALUMINUM HYDROXIDE, ASPIRIN/MAGNESIUM HYDROXIDE/ALUMINUM HYDROXIDE/CAFFEINE, ASPIRIN/MEPROBAMATE, ASPIRIN/SALICYLAMIDE/ACETAMINOPHEN/CAFFEINE, ASPIRIN/SALICYLAMIDE/CAFFEINE, ASPIRIN/SODIUM BICARBONATE/CITRIC ACID, BUTALBITAL/ASPIRIN/CAFFEINE, CARISOPRODOL/ASPIRIN, CHLORPHENIRAMINE MAL/PHENYLEPHRINE/D-METHORPHAN HB/ASPIRIN, CHLORPHENIRAMINE MALEATE/PHENYLEPHRINE BITARTRATE/ASPIRIN, CINNAMEDRINE HCL/ASPIRIN/CAFFEINE, CODEINE PHOSPHATE/BUTALBITAL/ASPIRIN/CAFFEINE, CODEINE PHOSPHATE/CARISOPRODOL/ASPIRIN, CODEINE/ASPIRIN/SALICYLAMIDE/ACETAMINOPHEN/CAFFEINE, DIHYDROCODEINE BITARTRATE/ASPIRIN/CAFFEINE, DIHYDROCODEINE/ASPIRIN/CAFFEINE, EPHEDRINE/ASPIRIN/ACETANILIDE/CAFFEINE, HYDROCODONE BITARTRATE/ASPIRIN, METHOCARBAMOL/ASPIRIN, ORPHENADRINE CITRATE/ASPIRIN/CAFFEINE, OXYCODONE HCL/ASPIRIN, OXYCODONE HCL/OXYCODONE TEREPHTHALATE/ASPIRIN, OXYCODONE/ASPIRIN, PENTAZOCINE HCL/ASPIRIN, PHENYLEPHRINE HCL/ASPIRIN, PHENYLPROPANOLAMINE BITARTRATE/ASPIRIN, PHENYLPROPANOLAMINE BITARTRATE/ASPIRIN/CHLORPHENIRAMINE, PHENYLPROPANOLAMINE HCL/ASPIRIN, PHENYLPROPANOLAMINE HCL/ASPIRIN/CHLORPHENIRAMINE, PHENYLPROPANOLAMINE HCL/ASPIRIN/CHLORPHENIRAMINE/CAFFEINE, PHENYLPROPANOLAMINE HCL/ASPIRIN/DIPHENHYDRAMINE, PROPOXYPHENE HCL/ASPIRIN/CAFFEINE, PSEUDOEPHEDRINE HCL/ASPIRIN/CHLORPHENIRAMINE, PRASUGREL HCL
Beta-Blockers	NDC Generic Name is any of: ATENOLOL, CARVEDILOL, METOPROLOL SUCCINATE, METOPROLOL TARTRATE, NADOLOL, PINDOLOL
Calcium Channel Blockers	NDC Generic Name is any of: ISRADIPINE, NIFEDIPINE, NIMODIPINE, NISOLDIPINE<br>NDC Brand Name is any of: ADALAT, CALAN, CARDENE, CARDIZEM, CLEVIPREX, COVERA-HS, DILT-CD, DYNACIRC, ISRADIPINE, NIFEDIPINE, NIMODIPINE, NISOLDIPINE, NORVASC, NYMALIZE, PLENDIL, PROCARDIA, SULAR, TIAZAC, VASCOR, VERELAN
Diuretics	NDC Brand Name is any of: BUMETANIDE, BUMEX, DELONE, DEMADEX, DETUE, DIAQUA-2, EDECRIN, ETHACRYNIC ACID, FUMIDE, FUROBEN 40, FUROMIDE, FUROSEMIDE, FUROSEMIDE-0.9 % NACL, FUROSEMIDE-0.9% NACL, LASAJECT-10, LASAJECT-2, LASIMIDE, LASIX, LO-AQUA, ROSE-40, TORSEMIDE, AMILORIDE HCL, AMILORIDE HCL W/HCTZ, AMILORIDE HCL-HCTZ, AMILORIDE HYDROCHLORIDE, AMILORIDE-HYDROCHLOROTHIAZIDE, EPLERENONE, SPIRONOLACTONE, SPIRONOLACTONE W/HCTZ, SPIRONOLACTONE-HCTZ, TRIAMTERENE, TRIAMTERENE W/HCTZ, TRIAMTERENE-HCTZ, TRIAMTERENE-HYDROCHLOROTHIAZID, ATENOLOL W/CHLORTHALIDONE, ATENOLOL-CHLORTHALIDONE, BENAZEPRIL-HYDROCHLOROTHIAZIDE, BENDROFLUMETHIAZIDE, BISOPROLOL-

© 2023 Lin KJ et al. *JAMA Network Open*.

	HYDROCHLOROTHIAZIDE, CAPTOPRIL-HYDROCHLOROTHIAZIDE, CAPTOPRIL/HYDROCHLOROTHIAZIDE, CHLOROTHIAZIDE, CHLOROTHIAZIDE SODIUM, CHLOROTHIAZIDE W/RESERPINE, CHLOROTHIAZIDE/RESERPINE, CHLORTHALIDONE, CLONIDINE HCL W/CHLORTHALIDONE, CLONIDINE W/CHLORTHALIDONE, ENALAPRIL-HYDROCHLOROTHIAZIDE, FOSINOPRIL-HYDROCHLOROTHIAZIDE, HYDROCHLOROTHIAZIDE, HYDROCHLOROTHIAZIDE PLUS, HYDROCHLOROTHIAZIDE/RESERPINE, HYDROFLUMETHIAZIDE, HYDROFLUMETHIAZIDE W/RESERPINE, HYDROFLUMETHIAZIDE/RESERPINE, INDAPAMIDE, IRBESARTAN-HYDROCHLOROTHIAZIDE, LISINOPRIL-HYDROCHLOROTHIAZIDE, LOSARTAN-HYDROCHLOROTHIAZIDE, METHYCLOTHIAZIDE, METHYCLOTHIAZIDE W/DESERPIDINE, METHYLDOPA W/CHLOROTHIAZIDE, METHYLDOPA-HYDROCHLOROTHIAZIDE, METHYLDOPA/HYDROCHLOROTHIAZIDE, METOLAZONE, METOPROLOL-HYDROCHLOROTHIAZIDE, MOEXIPRIL-HYDROCHLOROTHIAZIDE, NADOLOL-BENDROFLUMETHIAZIDE, QUINAPRIL-HYDROCHLOROTHIAZIDE, RAUWOLFIA/BENDROFLUMETHIAZIDE, RESERPINE/HYDROCHLOROTHIAZIDE, SK-CHLOROTHIAZIDE, SK-HYDROCHLOROTHIAZIDE, VALSARTAN-HYDROCHLOROTHIAZIDE
Fibrates	NDC Generic Name is any of: FENOFIBRATE, FENOFIBRATE NANOCRYSTALLIZED, FENOFIBRATE,MICRONIZED, FENOFIBRIC ACID, FENOFIBRIC ACID (CHOLINE), GEMFIBROZIL
Statins	NDC Generic Name is any of: LOVASTATIN, SIMVASTATIN, AMLODIPINE BESYLATE/ATORVASTATIN CALCIUM, ASPIRIN (CALCIUM CARB & MAGNESIUM BUFFERS)/PRAVASTATIN, ATORVASTATIN CALCIUM, CERIVASTATIN SODIUM, EZETIMIBE/ATORVASTATIN CALCIUM, EZETIMIBE/SIMVASTATIN, FLUVASTATIN SODIUM, IMIPENEM/CILASTATIN SODIUM, NIACIN/LOVASTATIN, NIACIN/SIMVASTATIN, NYSTATIN, NYSTATIN/EMOLLIENT COMBINATION NO.54, NYSTATIN/TRIAMCINOLONE ACETONIDE, PENTOSTATIN, PITAVASTATIN CALCIUM, PRAVASTATIN SODIUM, ROSUVASTATIN CALCIUM, SITAGLIPTIN PHOSPHATE/SIMVASTATIN
Nitrates	NDC Generic Name is any of: ISOSORBIDE DINITRATE, ISOSORBIDE DINITRATE/HYDRALAZINE HCL, ISOSORBIDE MONONITRATE, NITROGLYCERIN, NITROGLYCERIN/DEXTROSE 5 % IN WATER
Insulin	NDC Generic Name is any of: DILUENT,INSULIN ASPART COMBINATION #1, INSULIN ASPART, INSULIN ASPART PROTAMINE HUMAN/INSULIN ASPART, INSULIN DETEMIR, INSULIN GLARGINE,HUMAN RECOMBINANT ANALOG, INSULIN GLULISINE, INSULIN LISPRO, INSULIN LISPRO PROTAMINE & INSULIN LISPRO
Metformin	NDC Generic Name is any of: METFORMIN HCL, METFORMIN/AMINO ACIDS COMB. #7/HERBAL COMB.#125/CHOLINE, METFORMIN/CAFFEINE/AMINO ACIDS#7/HERBAL COMB#125/CHOLINE BIT, ALOGLIPTIN BENZOATE/METFORMIN HCL, CANAGLIFLOZIN/METFORMIN HCL, DAPAGLIFLOZIN PROPANEDIOL/METFORMIN HCL, GLIPIZIDE/METFORMIN HCL, GLYBURIDE/METFORMIN HCL, LINAGLIPTIN/METFORMIN HCL, PIOGLITAZONE HCL/METFORMIN HCL, REPAGLINIDE/METFORMIN HCL, ROSIGLITAZONE MALEATE/METFORMIN HCL, SAXAGLIPTIN HCL/METFORMIN HCL, SITAGLIPTIN PHOSPHATE/METFORMIN HCL
Sulfonylurea	NDC Generic Name is any of: CHLORPROPAMIDE, GLIMEPIRIDE, GLIPIZIDE, GLIPIZIDE/METFORMIN HCL, GLYBURIDE, GLYBURIDE,MICRONIZED, GLYBURIDE/METFORMIN HCL, PIOGLITAZONE HCL/GLIMEPIRIDE, ROSIGLITAZONE MALEATE/GLIMEPIRIDE, TOLAZAMIDE, TOLBUTAMIDE
Other oral hypoglycemic agents	NDC Generic Name is any of: ACARBOSE, ALOGLIPTIN BENZOATE, ALOGLIPTIN BENZOATE/PIOGLITAZONE HCL, EMPAGLIFLOZIN/LINAGLIPTIN, LINAGLIPTIN, MIGLITOL, NATEGLINIDE, PIOGLITAZONE HCL, REPAGLINIDE, ROSIGLITAZONE MALEATE, ROSIGLITAZONE MALEATE/GLIMEPIRIDE, SAXAGLIPTIN HCL, SITAGLIPTIN PHOSPHATE, SITAGLIPTIN PHOSPHATE/SIMVASTATIN, TROGLITAZONE, PIOGLITAZONE HCL/GLIMEPIRIDE, PIOGLITAZONE HCL/METFORMIN HCL
Corticosteroids	NDC Generic Name is any of: ACETIC ACID/HYDROCORTISONE, ACYCLOVIR/HYDROCORTISONE, BENZOYL PEROXIDE/HYDROCORTISONE, BENZOYL PEROXIDE/HYDROCORTISONE/SKIN CLEANSER COMB NO.14, CHLORCYCLIZINE HCL/HYDROCORTISONE ACETATE, CHLOROXYLENOL/BENZOCAINE/HYDROCORTISONE ACETATE, CIPROFLOXACIN HCL/DEXAMETHASONE, CIPROFLOXACIN HCL/HYDROCORTISONE, CLIOQUINOL/HYDROCORTISONE, CLIOQUINOL/HYDROCORTISONE/EMOLLIENT COMBINATION NO.88, CLIOQUINOL/HYDROCORTISONE/PRAMOXINE, CORTISONE ACETATE, DEXAMETHASONE, DEXAMETHASONE ACETATE, DEXAMETHASONE ACETATE, MICRONIZED, DEXAMETHASONE ISONICOTINATE, DEXAMETHASONE PHOSPHATE, DEXAMETHASONE PHOSPHATE/LIDOCAINE HCL, DEXAMETHASONE SOD PHOSPHATE, DEXAMETHASONE SODIUM PHOSPHATE IN 0.9 % SODIUM CHLORIDE, DEXAMETHASONE SODIUM PHOSPHATE/PF, DEXAMETHASONE, MICRONIZED, DIPHENHYDRAMINE

© 2023 Lin KJ et al. *JAMA Network Open*.

	HCL/HYDROCORTISONE, FLUDROCORTISONE ACETATE, GENTAMICIN SULFATE/PREDNISOLONE ACETATE, HYDROCORTISONE, HYDROCORTISONE ACETATE, HYDROCORTISONE ACETATE/ALOE POLYSACCHARIDE/IODOQUINOL, HYDROCORTISONE ACETATE/ALOE VERA, HYDROCORTISONE ACETATE/IODOQUINOL/ALOE POLYSACCHARIDES #2, HYDROCORTISONE ACETATE/IODOQUINOL/ALOE VERA, HYDROCORTISONE ACETATE/LIDOCAINE HCL/ALOE VERA, HYDROCORTISONE ACETATE/LIDOCAINE HCL/SKIN CLEANSER NO.6, HYDROCORTISONE ACETATE/PRAMOXINE HCL, HYDROCORTISONE ACETATE/PRAMOXINE HCL/ALOE POLYSACCHARIDE, HYDROCORTISONE ACETATE/PRAMOXINE HCL/EMOLLIENT BASE, HYDROCORTISONE ACETATE/PRAMOXINE HCL/SKIN CLEANSER NO.16, HYDROCORTISONE ACETATE/PRAMOXINE/EMOLLIENT/PRAMOXINE COMB#1, HYDROCORTISONE ACETATE/UREA, HYDROCORTISONE BUTYRATE, HYDROCORTISONE BUTYRATE/EMOLLIENT BASE, HYDROCORTISONE CYPIONATE, HYDROCORTISONE HEMISUCCINATE, HYDROCORTISONE PROBUTATE, HYDROCORTISONE SOD PHOSPHATE, HYDROCORTISONE SOD SUCCINATE, HYDROCORTISONE SODIUM SUCCINATE/PF, HYDROCORTISONE VALERATE, HYDROCORTISONE/ALOE POLYSACCHARIDE/IODOQUINOL, HYDROCORTISONE/ALOE VERA, HYDROCORTISONE/ALOE VERA/VITAMIN E ACETATE/VITAMINS A AND D, HYDROCORTISONE/BENZOCAINE/CHLOROXYLENOL, HYDROCORTISONE/COLLOIDAL OATMEAL/ALOE/VITAMIN E, HYDROCORTISONE/EMOLLIENT COMBINATION NO.45, HYDROCORTISONE/IODOQUINOL, HYDROCORTISONE/MINERAL OIL/PETROLATUM,WHITE, HYDROCORTISONE/PRAMOXINE HCL/CHLOROXYLENOL, HYDROCORTISONE/PRAMOXINE HCL/CHLOROXYLENOL/WATER, HYDROCORTISONE/PRAMOXINE/CHLOROXYLENOL/BENZALKONIUM, HYDROCORTISONE/RESORCINOL/BISMUTH SUBGALLATE/ZINC OXIDE, HYDROCORTISONE/SALICYLIC ACID/SULFUR, HYDROCORTISONE/SALICYLIC ACID/SULFUR/SHAMPOO CMB 1, HYDROCORTISONE/SKIN CLEANSER COMBINATION NO.25, HYDROCORTISONE/SKIN CLEANSER COMBINATION NO.35, HYDROCORTISONE/YERBA SANTA, KETOCONAZOLE/HYDROCORTISONE, LIDOCAINE HCL/HYDROCORTISONE ACETATE, LIDOCAINE HCL/HYDROCORTISONE ACETATE/PSYLLIUM HUSK, METHYLPREDNISOLONE, METHYLPREDNISOLONE ACETATE, METHYLPREDNISOLONE ACETATE, MICRONIZED, METHYLPREDNISOLONE ACETATE/BUPIVACAINE HCL, METHYLPREDNISOLONE SODIUM SUCCINATE, METHYLPREDNISOLONE SODIUM SUCCINATE/PF, METHYLPREDNISOLONE, MICRONIZED, NEOMYCIN SULFATE/BACITRACIN ZINC/POLYMYXIN B/HYDROCORTISONE, NEOMYCIN SULFATE/COLISTIN SULFATE/HYDROCORTISONE, NEOMYCIN SULFATE/COLISTIN SULFATE/HYDROCORTISONE/THONZONIUM, NEOMYCIN SULFATE/DEXAMETHASONE SOD PHOSPHATE, NEOMYCIN SULFATE/HYDROCORTISONE, NEOMYCIN SULFATE/HYDROCORTISONE ACETATE, NEOMYCIN SULFATE/POLYMYXIN B SULFATE/BUFFERS/HYDROCORTISONE, NEOMYCIN SULFATE/POLYMYXIN B SULFATE/HYDROCORTISONE, NEOMYCIN SULFATE/POLYMYXIN B SULFATE/PREDNISOLONE, NEOMYCIN/BACITRACIN/POLYMYXIN B/HYDROCORTISONE, NEOMYCIN/POLYMYXIN B SULFATE/DEXAMETHASONE, OXYTETRACYCLINE HCL/HYDROCORTISONE ACETATE, POLYMYXIN B SULFATE/HYDROCORTISONE, PREDNISOLONE, PREDNISOLONE ACETATE, PREDNISOLONE ACETATE, MICRONIZED, PREDNISOLONE SOD PHOSPHATE, PREDNISOLONE SODIUM PHOSPHATE/PEAK FLOW METER, PREDNISOLONE, MICRONIZED, PREDNISONE, PREDNISONE MICRONIZED, SULFACETAMIDE SODIUM/PREDNISOLONE ACETATE, SULFACETAMIDE SODIUM/PREDNISOLONE SODIUM PHOSPHATE, TOBRAMYCIN/DEXAMETHASONE
Estrogen	NDC Generic Name is any of: 2-METHOXYESTRADIOL, DESOGESTREL-ETHINYL ESTRADIOL, DESOGESTREL-ETHINYL ESTRADIOL/ETHINYL ESTRADIOL, DROSPIRENONE/ESTRADIOL, DROSPIRENONE/ETHINYL ESTRADIOL/LEVOMEFOLATE CALCIUM, ESTRADIOL, ESTRADIOL ACETATE, ESTRADIOL BENZOATE, ESTRADIOL CYPIONATE, ESTRADIOL CYPIONATE/MEDROXYPROGESTERONE ACETATE, ESTRADIOL HEMIHYDRATE, MICRONIZED, ESTRADIOL MICRONIZED, ESTRADIOL VALERATE, ESTRADIOL VALERATE/DIENOGEST, ESTRADIOL/LEVONORGESTREL, ESTRADIOL/NORETHINDRONE ACETATE, ESTRADIOL/NORGESTIMATE, ESTROGENS, CONJUGATED, ESTROGENS, CONJUGATED/BAZEDOXIFENE ACETATE, ESTROGENS, CONJUGATED/MEDROXYPROGESTERONE ACETATE, ESTROGENS, CONJUGATED/MEPROBAMATE, ESTROGENS,CONJ.,SYNTHETIC A, ESTROGENS,CONJ.,SYNTHETIC B, ESTROGENS,ESTERIFIED, ESTROGENS,ESTERIFIED/METHYLTESTOSTERONE, ESTROPIPATE, ETHINYL ESTRADIOL, ETHINYL ESTRADIOL/DROSPIRENONE, ETHYNODIOL DIACETATE-ETHINYL ESTRADIOL, ETONOGESTREL/ETHINYL ESTRADIOL, LEVONORGESTREL-ETH ESTRA/ETHINYL ESTRADIOL, LEVONORGESTREL-ETHINYL ESTRADIOL, LEVONORGESTREL-ETHINYL

© 2023 Lin KJ et al. *JAMA Network Open*.

	ESTRADIOL/PREGNANCY TEST KIT, NORELGESTROMIN/ETHINYL ESTRADIOL, NORETHINDRONE ACETATE-ETHINYL ESTRADIOL, NORETHINDRONE ACETATE-ETHINYL ESTRADIOL/FERROUS FUMARATE, NORETHINDRONE-ETHINYL ESTRADIOL, NORETHINDRONE-ETHINYL ESTRADIOL/FERROUS FUMARATE, NORGESTIMATE-ETHINYL ESTRADIOL, NORGESTREL-ETHINYL ESTRADIOL, TESTOSTERONE CYPIONATE/ESTRADIOL CYPIONATE, TESTOSTERONE ENANTHATE/ESTRADIOL VALERATE
Thyroid hormone replacement	NDC Generic Name is any of: LEVOTHYROXINE SODIUM, LIOTHYRONINE SODIUM, LIOTHYRONINE SODIUM, MICRONIZED, LIOTRIX
H2 blockers	NDC Generic Name is any of: CIMETIDINE, CIMETIDINE HCL, CIMETIDINE HCL IN 0.9 % SODIUM CHLORIDE, FAMOTIDINE, FAMOTIDINE IN 0.9 % SODIUM CHLORIDE, FAMOTIDINE IN SODIUM CHLORIDE, ISO-OSMOTIC/PF, FAMOTIDINE/CALCIUM CARBONATE/MAGNESIUM HYDROXIDE, FAMOTIDINE/PF, IBUPROFEN/FAMOTIDINE, NIZATIDINE, RANITIDINE BISMUTH CITRATE, RANITIDINE HCL, RANITIDINE HCL IN 0.45 % SODIUM CHLORIDE, RANITIDINE HCL/DIETARY SUPPLEMENT,MISC COMB17, RANITIDINE HCL/DIETARY SUPPLEMENT,MISC.COMBO8
Proton-pump inhibitors	NDC Generic Name is any of: ESOMEPRAZOLE MAGNESIUM, ESOMEPRAZOLE SODIUM, ESOMEPRAZOLE STRONTIUM, LANSOPRAZOLE, LANSOPRAZOLE/AMOXICILLIN TRIHYDRATE/CLARITHROMYCIN, LANSOPRAZOLE/NAPROXEN, NAPROXEN/ESOMEPRAZOLE MAGNESIUM, OMEPRAZOLE, OMEPRAZOLE MAGNESIUM, OMEPRAZOLE/CLARITHROMYCIN/AMOXICILLIN TRIHYDRATE, OMEPRAZOLE/SODIUM BICARBONATE, PANTOPRAZOLE SODIUM, RABEPRAZOLE SODIUM
Other antacids: sucralfate	NDC Generic Name is any of: SUCRALFATE, SUCRALFATE MALATE, POLYMERIZED
Anticonvulsants	NDC Generic Name is any of: AMINOPHYLLINE/EPHEDRINE/POTASSIUM IODIDE/PHENOBARBITAL, BELLADONNA ALKALOIDS/PHENOBARBITAL, CARBAMAZEPINE, ERGOTAMINE TARTRATE/BELLADONNA ALKALOIDS/PHENOBARBITAL, ETHOSUXIMIDE, EZOGABINE, FELBAMATE, GABAPENTIN, GABAPENTIN ENACARBIL, GABAPENTIN/DIETARY SUPPLEMENT, MISC COMBO NO.11, GUAIFENESIN/DYPHYLLINE/EPHEDRINE/PHENOBARBITAL, HYOSCYAMINE SULFATE/PHENOBARBITAL, LACOSAMIDE, LAMOTRIGINE, LEVETIRACETAM, LEVETIRACETAM IN SODIUM CHLORIDE, ISO-OSMOTIC, OXCARBAZEPINE, PHENOBARBITAL, PHENOBARBITAL SODIUM, PHENOBARBITAL SODIUM IN 0.9 % SODIUM CHLORIDE, PHENOBARBITAL/HYOSCYAMINE SULF/ATROPINE SULF/SCOPOLAMINE HB, PHENTERMINE HCL/TOPIRAMATE, PHENYTOIN, PHENYTOIN SODIUM, PHENYTOIN SODIUM EXTENDED, PREGABALIN, PRIMIDONE, PROPANTHELINE BROMIDE/PHENOBARBITAL, RUFINAMIDE, THEOPHYLLINE/EPHEDRINE HCL/PHENOBARBITAL, THEOPHYLLINE/EPHEDRINE/POTASSIUM IODIDE/PHENOBARBITAL, TIAGABINE HCL, TOPIRAMATE, VALPROIC ACID (AS SODIUM SALT) (VALPROATE SODIUM), VIGABATRIN, ZONISAMIDE
Dementia drugs	NDC Generic Name is any of: DONEPEZIL HCL, MEMANTINE HCL/DONEPEZIL HCL, RIVASTIGMINE, RIVASTIGMINE TARTRATE, GALANTAMINE HBR, MEMANTINE HCL
Parkinsonism drugs	NDC Generic Name is any of: LEVODOPA, CARBIDOPA/LEVODOPA, CARBIDOPA/LEVODOPA/ENTACAPONE, ROPINIROLE HCL, PRAMIPEXOLE DI-HCL, ROTIGOTINE, AMANTADINE HCL, TRIHEXYPHENIDYL HCL, BENZTROPINE MESYLATE, SELEGILINE, SELEGILINE HCL, RASAGILINE MESYLATE, TOLCAPONE, ENTACAPONE, APOMORPHINE HCL
NSAIDs	NDC Generic Name is any of: CELECOXIB, DIFLUNISAL, ETODOLAC, FLURBIPROFEN, IBUPROFEN, INDOMETHACIN, KETOPROFEN, MELOXICAM, NABUMETONE, NAPROXEN, OXAPROZIN, PIROXICAM, ROFECOXIB, SULINDAC, VALDECOXIB
Opioids	NDC Generic Name is any of: ACETAMINOPHEN WITH CODEINE PHOSPHATE, AMMONIUM CHL/POTASSIUM GUAICO/CODEINE/DIPHEN/BROMODIPHENHYD, AMMONIUM CHLORIDE/PHENYLEPHRINE HCL/CODEINE/CHLORPHENIRAMINE, ASPIRIN/CODEINE PHOSPHATE, BROMPHENIRAMINE MALEATE/CODEINE PHOSPHATE, BROMPHENIRAMINE MALEATE/PHENYLEPHRINE HCL/CODEINE PHOSPHATE, BROMPHENIRAMINE MALEATE/PHENYLEPHRINE HCL/DIHYDROCODEINE BT, BROMPHENIRAMINE MALEATE/PSEUDOEPHEDRINE HCL/CODEINE, BROMPHENIRAMINE/PSEUDOEPHEDRINE HCL/DIHYDROCODEINE, BUTALBITAL/ACETAMINOPHEN/CAFFEINE/CODEINE PHOSPHATE, CHLORCYCLIZINE HCL/CODEINE PHOSPHATE, CHLORCYCLIZINE HCL/PHENYLEPHRINE HCL/CODEINE PHOSPHATE, CHLORCYCLIZINE HCL/PSEUDOEPHEDRINE HCL/CODEINE PHOSPHATE, CHLORPHENIRAMINE MALEATE/CODEINE PHOSPHATE, CHLORPHENIRAMINE MALEATE/CODEINE PHOSPHATE/ACETAMINOPHEN, CHLORPHENIRAMINE MALEATE/PHENYLEPHRINE HCL/CODEINE PHOSPHATE, CHLORPHENIRAMINE MALEATE/PSEUDOEPHEDRINE HCL/CODEINE, CODEINE PHOSPHATE, CODEINE PHOSPHATE/BROMODIPHENHYDRAMINE HCL, CODEINE

© 2023 Lin KJ et al. *JAMA Network Open*.

	PHOSPHATE/BUTALBITAL/ASPIRIN/CAFFEINE, CODEINE PHOSPHATE/CARISOPRODOL/ASPIRIN, CODEINE PHOSPHATE/PYRILAMINE MALEATE, CODEINE POLISTIREX/CHLORPHENIRAMINE POLISTIREX, CODEINE SULFATE, CODEINE/ASPIRIN/SALICYLAMIDE/ACETAMINOPHEN/CAFFEINE, CODEINE/CALCIUM IODIDE, DEXBROMPHENIRAMINE MALEATE/PSEUDOEPHEDRINE HCL/CODEINE PHOS, DEXCHLORPHENIRAMINE MALEATE/PHENYLEPHRINE HCL/CODEINE, DIHYDROCODEINE BITARTRATE, DIHYDROCODEINE BITARTRATE/ACETAMINOPHEN/CAFFEINE, DIHYDROCODEINE BITARTRATE/ASPIRIN/CAFFEINE, DIHYDROCODEINE BITARTRATE/GUAIFENESIN, DIHYDROCODEINE/ASPIRIN/CAFFEINE, DIPHENHYDRAMINE HCL/PHENYLEPHRINE HCL/CODEINE PHOSPHATE, FENTANYL, FENTANYL CITRATE, FENTANYL CITRATE IN 0.9 % SODIUM CHLORIDE/PF, FENTANYL CITRATE/BUPIVACAINE HCL IN 0.9 % SODIUM CHLORIDE/PF, FENTANYL CITRATE/DEXTROSE 5%-WATER/PF, FENTANYL CITRATE/DROPERIDOL, FENTANYL CITRATE/PF, FENTANYL CITRATE/ROPIVACAINE HCL/SODIUM CHLORIDE 0.9%/PF, FENTANYL HCL, GUAIFENESIN/CODEINE PHOSPHATE, GUAIFENESIN/HYDROCODONE BITARTRATE, GUAIFENESIN/HYDROCODONE BITARTRATE/BROMPHENIRAMINE, GUAIFENESIN/PHENYLEPHRINE HCL/HYDROCODONE, GUAIFENESIN/PHENYLEPHRINE HCL/PHENYLPROP/HYDROCODONE/PHENIR, GUAIFENESIN/PHENYLPROPANOLAMINE HCL/CODEINE, GUAIFENESIN/PHENYLPROPANOLAMINE HCL/HYDROCODONE/PYRIL/PHENIR, GUAIFENESIN/PSEUDOEPHEDRINE HCL/DIHYDROCODEINE, GUAIFENESIN/PSEUDOEPHEDRINE HCL/HYDROCODONE/CHLORPHENIRAMINE, HYDROCODONE BITARTRATE, HYDROCODONE BITARTRATE/ACETAMINOPHEN, HYDROCODONE BITARTRATE/ACETAMINOPHEN/DIETARY SUPPLEMENT #11, HYDROCODONE BITARTRATE/ASPIRIN, HYDROCODONE BITARTRATE/CHLORPHENIRAMINE MALEATE, HYDROCODONE BITARTRATE/HOMATROPINE, HYDROCODONE BITARTRATE/HOMATROPINE METHYLBROMIDE, HYDROCODONE BITARTRATE/PSEUDOEPHEDRINE HCL/GUAIFENESIN, HYDROCODONE POLISTIREX/CHLORPHENIRAMINE POLISTIREX, HYDROCODONE TANNATE/CHLORPHENIRAMINE TANNATE, HYDROCODONE/IBUPROFEN, HYDROMORPHONE HCL, HYDROMORPHONE HCL IN 0.9 % SODIUM CHLORIDE, HYDROMORPHONE HCL IN 0.9 % SODIUM CHLORIDE/PF, HYDROMORPHONE HCL IN DEXTROSE 5 %-WATER/PF, HYDROMORPHONE HCL/BUPIVACAINE HCL IN 0.9% SODIUM CHLORIDE/PF, HYDROMORPHONE HCL/GUAIFENESIN, HYDROMORPHONE HCL/PF, IBUPROFEN/OXYCODONE HCL, LEVORPHANOL TARTRATE, MEPERIDINE HCL, MEPERIDINE HCL IN 0.9 % SODIUM CHLORIDE, MEPERIDINE HCL/ATROPINE SULFATE, MEPERIDINE HCL/PF, MEPERIDINE HCL/PROMETHAZINE HCL, METHADONE HCL, METHADONE HYDROCHLORIDE IN 0.9 % SODIUM CHLORIDE, MORPHINE SULFATE, MORPHINE SULFATE IN 0.9 % SODIUM CHLORIDE, MORPHINE SULFATE IN 0.9 % SODIUM CHLORIDE/PF, MORPHINE SULFATE IN SODIUM CHLORIDE, ISO-OSMOTIC/PF, MORPHINE SULFATE LIPOSOMAL/PF, MORPHINE SULFATE/DEXTROSE 5 % IN WATER, MORPHINE SULFATE/DEXTROSE 5%-WATER/PF, MORPHINE SULFATE/NALTREXONE HCL, MORPHINE SULFATE/PF, OXYCODONE HCL, OXYCODONE HCL/ACETAMINOPHEN, OXYCODONE HCL/ASPIRIN, OXYCODONE HCL/OXYCODONE TEREPHTHALATE/ASPIRIN, OXYCODONE/ASPIRIN, OXYMORPHONE HCL, PENTAZOCINE HCL/ACETAMINOPHEN, PENTAZOCINE HCL/ASPIRIN, PENTAZOCINE HCL/NALOXONE HCL, PENTAZOCINE LACTATE, PHENYLEPHRINE HCL/CODEINE PHOSPHATE, PHENYLEPHRINE HCL/CODEINE PHOSPHATE/ACETAMINOPHEN/GUAIFEN, PHENYLEPHRINE HCL/CODEINE PHOSPHATE/GUAIFENESIN, PHENYLEPHRINE HCL/CODEINE PHOSPHATE/PHENIRAMINE/SODIUM CIT, PHENYLEPHRINE HCL/CODEINE/ACETAMINOPHEN/CHLORPHENIRAMINE, PHENYLEPHRINE HCL/CODEINE/CHLORPHENIRAMINE, PHENYLEPHRINE HCL/CODEINE/CHLORPHENIRAMINE/POTASSIUM IODIDE, PHENYLEPHRINE HCL/CODEINE/PYRILAMINE, PHENYLEPHRINE HCL/DIHYDROCODEINE BITARTRATE, PHENYLEPHRINE HCL/DIHYDROCODEINE BITARTRATE/CHLORPHENIRAMINE, PHENYLEPHRINE HCL/DIHYDROCODEINE BITARTRATE/GUAIFENESIN, PHENYLEPHRINE HCL/HYDROCODONE BITARTRATE, PHENYLEPHRINE HCL/HYDROCODONE BITARTRATE/BROMPHENIRAMINE, PHENYLEPHRINE HCL/HYDROCODONE BITARTRATE/CARBINOXAMINE, PHENYLEPHRINE HCL/HYDROCODONE BITARTRATE/CHLORPHENIRAMINE, PHENYLEPHRINE HCL/HYDROCODONE BITARTRATE/DEXBROMPHENIRAMINE, PHENYLEPHRINE HCL/HYDROCODONE BITARTRATE/DEXCHLORPHENIRAMINE, PHENYLEPHRINE HCL/HYDROCODONE BITARTRATE/DIPHENHYDRAMINE, PHENYLEPHRINE HCL/HYDROCODONE BITARTRATE/PYRIL/CHLORPHENIR, PHENYLEPHRINE HCL/HYDROCODONE BITARTRATE/PYRILAMINE, PHENYLEPHRINE HCL/PPA HCL/DIHYDROCODEINE/CHLORPHENIRAMINE, PHENYLEPHRINE TANNATE/HYDROCODONE

© 2023 Lin KJ et al. *JAMA Network Open*.

	TANNATE/DIPHENHYDRAMINE, PHENYLEPHRINE/HYDROCODONE BIT/ACETAMINOPHEN/CHLORPHENIRAMINE, PHENYLEPHRINE/HYDROCODONE/BROMPHENIRAMINE TANNATES, PHENYLEPHRINE/HYDROCODONE/DEXCHLORPHENIRAMINE TANNATES, PHENYLEPHRINE/PSEUDOEPHED/HYDROCODONE/PYRILAMINE/CHLORPHENIR, PHENYLPROPANOLAMINE HCL/CODEINE/BROMPHENIRAMINE, PHENYLPROPANOLAMINE HCL/HYDROCODONE BITARTRATE, POTASSIUM GUAIACOLSULFONATE/HYDROCODONE BITARTRATE, POTASSIUM GUAIACOLSULFONATE/PSEUDOEPHEDRINE HCL/HYDROCODONE, PROMETHAZINE HCL/CODEINE, PROMETHAZINE/PHENYLEPHRINE HCL/CODEINE, PROPOXYPHENE HCL, PROPOXYPHENE HCL/ACETAMINOPHEN, PROPOXYPHENE HCL/ASPIRIN/CAFFEINE, PROPOXYPHENE NAPSYLATE, PROPOXYPHENE NAPSYLATE/ACETAMINOPHEN, PSEUDOEPHEDRINE HCL/CODEINE PHOSPHATE, PSEUDOEPHEDRINE HCL/CODEINE PHOSPHATE/ACETAMINOPHEN/GUAIFEN, PSEUDOEPHEDRINE HCL/CODEINE PHOSPHATE/GUAIFENESIN, PSEUDOEPHEDRINE HCL/CODEINE PHOSPHATE/TRIPROLIDINE, PSEUDOEPHEDRINE HCL/CODEINE/CHLORPHENIRAMINE, PSEUDOEPHEDRINE HCL/DIHYDROCODEINE BITARTRATE/CHLORPHENIR, PSEUDOEPHEDRINE HCL/HYDROCODONE BITARTRATE, PSEUDOEPHEDRINE HCL/HYDROCODONE BITARTRATE/BROMPHENIRAMINE, PSEUDOEPHEDRINE HCL/HYDROCODONE BITARTRATE/CARBINOXAMINE, PSEUDOEPHEDRINE HCL/HYDROCODONE BITARTRATE/CHLORPHENIRAMINE, PSEUDOEPHEDRINE HCL/HYDROCODONE BITARTRATE/TRIPROLIDINE, PSEUDOEPHEDRINE TANNATE/HYDROCODONE TANNATE, PSEUDOEPHEDRINE/HYDROCODONE/BROMPHENIRAMINE TANNATES, PSEUDOEPHEDRINE/HYDROCODONE/CHLORPHENIRAMINE TANNATES, PYRILAMINE MALEATE/PHENYLEPHRINE HCL/CODEINE PHOSPHATE, PYRILAMINE MALEATE/PHENYLEPHRINE HCL/DIHYDROCODEINE BT, PYRILAMINE MALEATE/PSEUDOEPHEDRINE HCL/CODEINE PHOSPHATE, TAPENTADOL HCL, TRAMADOL HCL, TRAMADOL HCL/ACETAMINOPHEN, TRAMADOL HCL/DIETARY SUPPLEMENT,MISC. CB.11
Antipsychotic agents	NDC Generic Name is any of: ACEPROMAZINE MALEATE, ARIPIPRAZOLE, ASENAPINE MALEATE, CHLORPROMAZINE HCL, CLOZAPINE, FLUPHENAZINE DECANOATE, FLUPHENAZINE ENANTHATE, FLUPHENAZINE HCL, HALOPERIDOL, HALOPERIDOL DECANOATE, HALOPERIDOL LACTATE, ILOPERIDONE, LOXAPINE, LOXAPINE HCL, LOXAPINE SUCCINATE, LURASIDONE HCL, MESORIDAZINE BESYLATE, MOLINDONE HCL, OLANZAPINE, OLANZAPINE PAMOATE, OLANZAPINE/FLUOXETINE HCL, PALIPERIDONE, PALIPERIDONE PALMITATE, PERPHENAZINE, PERPHENAZINE/AMITRIPTYLINE HCL, PIMOZIDE, PROMAZINE HCL, QUETIAPINE FUMARATE, RISPERIDONE, RISPERIDONE MICROSPHERES, THIORIDAZINE HCL, THIOTHIXENE, THIOTHIXENE HCL, TRIFLUOPERAZINE HCL, TRIFLUPROMAZINE HCL, ZIPRASIDONE HCL, ZIPRASIDONE MESYLATE
Anxiolytics (except benzodiazepines)	NDC Generic Name is any of: BUSPIRONE HCL, MEPROBAMATE, ASPIRIN/MEPROBAMATE, ESTROGENS, CONJUGATED/MEPROBAMATE
Benzodiazepines	NDC Generic Name is any of: ALPRAZOLAM, ALPRAZOLAM/DIETARY SUPPLEMENT,MISC COMBO NO.17, AMITRIPTYLINE HCL/CHLORDIAZEPOXIDE, CHLORDIAZEPOXIDE, CHLORDIAZEPOXIDE HCL, CHLORDIAZEPOXIDE HCL/METHSCOPOLAMINE NITRATE, CHLORDIAZEPOXIDE/CLIDINIUM BROMIDE, CLOBAZAM, CLONAZEPAM, CLORAZEPATE DIPOTASSIUM, DIAZEPAM, DIAZEPAM/SOYBEAN OIL, ESTAZOLAM, FLURAZEPAM HCL, LORAZEPAM, LORAZEPAM IN 0.9 % SODIUM CHLORIDE, LORAZEPAM/DEXTROSE 5 % IN WATER, MIDAZOLAM, MIDAZOLAM HCL, MIDAZOLAM HCL IN 0.9 % SODIUM CHLORIDE, MIDAZOLAM HCL IN 0.9 % SODIUM CHLORIDE/PF, MIDAZOLAM HCL IN DEXTROSE 5% IN WATER, MIDAZOLAM HCL/DEXTROSE 5%-WATER/PF, MIDAZOLAM HCL/PF, OXAZEPAM, QUAZEPAM, TEMAZEPAM, TEMAZEPAM/DIETARY SUPPLEMENT,MISC.COMBO8, TRIAZOLAM
Hypnotics	NDC Generic Name is any of: ACETAMINOPHEN/DIPHENHYDRAMINE HCL, AMINOGLUTETHIMIDE, ASPIRIN/DIPHENHYDRAMINE HCL, ASPIRIN/DIPHENHYDRAMINE/SODIUM BICARBONATE/CITRIC ACID, ASPIRIN/MEPROBAMATE, BROMPHENIRAMINE MAL/DIPHENHYDRAMINE HCL/PHENYLEPHRINE HCL, BROMPHENIRAMINE MALEATE/DIPHENHYDRAMINE HCL, BUSPIRONE HCL, CARBETAPENTANE TANNATE/PHENYLEPHRINE TANNATE/DIPHENHYDRAMINE, CHLORAL HYDRATE, DEXTROMETHORPHAN HBR/ACETAMINOPHEN/DIPHENHYDRAMINE HCL, DIPHENHYDRAMINE HCL, DIPHENHYDRAMINE HCL IN 0.9 % SODIUM CHLORIDE, DIPHENHYDRAMINE HCL/ALLANTOIN, DIPHENHYDRAMINE HCL/BENZETHONIUM CHLORIDE/ZINC ACETATE, DIPHENHYDRAMINE HCL/CALAMINE, DIPHENHYDRAMINE HCL/CAMPHOR/CALAMINE, DIPHENHYDRAMINE HCL/HYDROCORTISONE, DIPHENHYDRAMINE HCL/MENTHOL, DIPHENHYDRAMINE HCL/PHENYLEPHRINE HCL/ACETAMINOPHEN, DIPHENHYDRAMINE HCL/PHENYLEPHRINE HCL/ACETAMINOPHEN/GUAIFEN, DIPHENHYDRAMINE

© 2023 Lin KJ et al. *JAMA Network Open*.

	HCL/PHENYLEPHRINE HCL/CODEINE PHOSPHATE, DIPHENHYDRAMINE HCL/PHENYLEPHRINE HCL/DEXTROMETHORPHAN HBR, DIPHENHYDRAMINE HCL/TRIPELENNAMINE HCL/MENTHOL, DIPHENHYDRAMINE HCL/ZINC ACETATE, DIPHENHYDRAMINE/PHENYLEPHRIN/DEXTROMETHORPH/ACETAMINOPHEN/GG, ESTROGENS, CONJUGATED/MEPROBAMATE, ESZOPICLONE, ETHCHLORVYNOL, HYDROXYZINE HCL, HYDROXYZINE PAMOATE, IBUPROFEN/DIPHENHYDRAMINE HCL, MAGNESIUM SALICYLATE/DIPHENHYDRAMINE HCL, MEPROBAMATE, NAPROXEN SODIUM/DIPHENHYDRAMINE HCL, PHENYLEPHRINE HCL/DIPHENHYDRAMINE HCL, PHENYLEPHRINE HCL/HYDROCODONE BITARTRATE/DIPHENHYDRAMINE, PHENYLEPHRINE TANNATE/HYDROCODONE TANNATE/DIPHENHYDRAMINE, PHENYLEPHRINE/ACETAMINOPHEN/DIPHENHYDRAMINE/CHLORPHENIRAMINE, PHENYLPROPANOLAMINE HCL/ASPIRIN/DIPHENHYDRAMINE, PRAMOXINE HCL/DIPHENHYDRAMINE HCL, PSEUDOEPHEDRINE HCL/ACETAMINOPHEN/DIPHENHYDRAMINE, PSEUDOEPHEDRINE HCL/DIPHENHYDRAMINE HCL, THEOPHYLLINE/EPHEDRINE/HYDROXYZINE, ZALEPLON, ZOLPIDEM TARTRATE
SSRI/SNRI antidepressants	NDC Generic Name is any of: CITALOPRAM HYDROBROMIDE, DULOXETINE HCL, ESCITALOPRAM OXALATE, FLUOXETINE, FLUOXETINE HCL, FLUOXETINE HCL/DIETARY SUPPLEMENT,MISC COMB17, FLUOXETINE HCL/DIETARY SUPPLEMENT,MISC.COMBO8, FLUVOXAMINE MALEATE, OLANZAPINE/FLUOXETINE HCL, PAROXETINE HCL, PAROXETINE MESYLATE, SERTRALINE HCL, DESVENLAFAXINE, DESVENLAFAXINE FUMARATE, DESVENLAFAXINE SUCCINATE, VENLAFAXINE HCL, LEVOMILNACIPRAN HYDROCHLORIDE
Tricyclic antidepressants	NDC Generic Name is any of: AMITRIPTYLINE HCL, AMITRIPTYLINE HCL/CHLORDIAZEPOXIDE, AMOXAPINE, DESIPRAMINE HCL, DOXEPIN HCL, IMIPRAMINE HCL, IMIPRAMINE PAMOATE, NORTRIPTYLINE HCL, PERPHENAZINE/AMITRIPTYLINE HCL, PROTRIPTYLINE HCL, TRIMIPRAMINE MALEATE, CLOMIPRAMINE HCL
Other antidepressants	NDC Generic Name is any of: BUPROPION HBR, BUPROPION HCL, BUPROPION HCL/DIETARY SUPPLEMENT,MISC COMB15, BUPROPION HCL/DIETARY SUPPLEMENT,MISC COMB16, ISOCARBOXAZID, MIRTAZAPINE, NALTREXONE HCL/BUPROPION HCL, NEFAZODONE HCL, PHENELZINE SULFATE, TRAZODONE HCL, TRAZODONE HCL/DIETARY SUPPLEMENT,MISC.COMBO8, VILAZODONE HYDROCHLORIDE, VORTIOXETINE HYDROBROMIDE, MAPROTILINE HCL
Bronchodilators	NDC Generic Name is any of: ALBUTEROL, ALBUTEROL SULFATE, IPRATROPIUM BROMIDE/ALBUTEROL SULFATE, LEVALBUTEROL HCL, LEVALBUTEROL TARTRATE, METAPROTERENOL SULFATE, PIRBUTEROL ACETATE, SALMETEROL XINAFOATE, FLUTICASONE PROPIONATE/SALMETEROL XINAFOATE, FORMOTEROL FUMARATE, ARFORMOTEROL TARTRATE, BUDESONIDE/FORMOTEROL FUMARATE, MOMETASONE FUROATE/FORMOTEROL FUMARATE
Corticosteroids, inhaled	NDC Generic Name is any of: AZELASTINE HCL/FLUTICASONE PROPIONATE, BECLOMETHASONE DIPROPIONATE, BUDESONIDE, BUDESONIDE, MICRONIZED, BUDESONIDE/FORMOTEROL FUMARATE, CICLESONIDE, FLUNISOLIDE, FLUNISOLIDE/MENTHOL, FLUTICASONE FUROATE, FLUTICASONE FUROATE/VILANTEROL TRIFENATATE, FLUTICASONE PROPIONATE, FLUTICASONE PROPIONATE/SALMETEROL XINAFOATE, MOMETASONE FUROATE, MOMETASONE FUROATE/AMMONIUM LACTATE, MOMETASONE FUROATE/FORMOTEROL FUMARATE
Endoscopy	ICD-9 Procedure Code (any position) is any of: 45.1, 45.11, 45.12, 45.13, 45.14, 45.15, 45.16, 45.19, 45.2, 45.21, 45.22, 45.23, 45.24, 45.25, 45.26, 45.27, 45.28, 45.29<br>ICD-10 Procedure Code (any position) is any of: 0D927ZX, 0D958ZX, 0D987ZX, 0D9A7ZX, 0D9A8ZX, 0D9B7ZX, 0DB28ZX, 0DB47ZX, 0DB67ZX, 0DJ08ZZ, 0D928ZX, 0D947ZX, 0D957ZX, 0D988ZX, 0D9B8ZX, 0DB27ZX, 0DB57ZX, 0DB77ZX, 0DJ68ZZ, 0D917ZX, 0D938ZX, 0D948ZX, 0D9C4ZX, 0DB17ZX, 0DB48ZX, 0D918ZX, 0D967ZX, 0D977ZX, 0D997ZX, 0D998ZX, 0D9C7ZX, 0D9C8ZX, 0DB18ZX, 0D937ZX, 0D968ZX, 0D978ZX, 0DB37ZX, 0DB38ZX, 0DB58ZX, 0DB68ZX, 0DB78ZX, 0DB97ZX, 0DB98ZX
Liver disease	ICD-9 Diagnosis Code (any position) is any of: 070.2, 070.21, 070.52, 070.70, 456.21, 573.0, 573.1, 573.2, 573.5, 573.9, 576.8, 782.4, 070.0, 070.1, 070.20, 070.22, 070.23, 070.3, 070.41, 070.5, 070.51, 070.53, 070.71, 070.30, 070.4, 070.54, 070.59, 070.7, 570, 573.3, 573.4, 573.8, 070.31, 070.32, 070.33, 070.44, 070.49, 070.6, 070.9, 456.1, 456.2, 070.42, 070.43, 185.0, 456.0, 789.51, 789.57, 789.58, 789.59, 185.05, 198.30, 456.20, 789.5, 571.0, 571.1, 571.3, 571.40, 571.49, 571.5, 571.6, 571.8, 572, 572.0, 572.3, 572.4, 571, 571.2, 571.4, 571.41, 571.42, 571.9, 572.1, 572.2, 572.8<br>ICD-10 Diagnosis Code (any position) is any of: B16.1, B17.11, B17.2, B17.9, B18.2, B18.8, B19.0, B19.9, I85.11, K87, B15.9, B16.0, B16.2, B16.9, B18.0, B18.9, B19.10, B19.11, K76.2, K83.8, R17, B15.0, B19.21, K72.00, B17.0, B17.10, B17.8, B18.1, B19.20, I85.10, K83.5, I85.0, K74.6, R18, R18.0, R18.8, I85.00, I85.01, K70.10, K70.11, K70.2, K70.31,

© 2023 Lin KJ et al. *JAMA Network Open*.

	K70.40, K70.41, K71.0, K71.10, K71.11, K71.2, K71.3, K71.51, K71.6, K72.01, K72.10, K72.90, K72.91, K73.2, K73.8, K74.1, K74.2, K74.69, K75.0, K75.1, K75.4, K75.81, K75.89, K76.0, K76.3, K76.4, K76.5, K76.6, K76.7, K76.9, K77, B25.1, K70.0, K70.30, K70.9, K71.4, K71.50, K71.7, K71.8, K71.9, K72.11, K73.0, K73.1, K73.9, K74.0, K74.3, K74.4, K74.5, K74.60, K75.2, K75.3, K75.9, K76.1, K76.81, K76.89<br>ICD-9 Procedure Code (any position) is any of: 39.1, 42.91<br>ICD-10 Procedure Code (any position) is any of: 06100Z5, 0610496, 06104K5, 06104Z5, 06104ZY, 061107B, 0611099, 06110AY, 06110J9, 0611479, 061147Y, 06114J9, 06120ZY, 06140AY, 061507Y, 061509Y, 061549Y, 06154AY, 061647Y, 061709Y, 061807Y, 061809B, 061809Y, 06180J9, 06180Z9, 0618479, 061849B, 06184AB, 06184JB, 06184ZB, 06190ZY, 061947Y, 061949Y, 061B0ZY, 061B4AY, 061B4KY, 061J09Y, 061J0KY, 06L30ZZ, 061007Y, 06100A5, 06100J5, 06100K6, 06110JB, 06110KB, 06114JY, 061207Y, 061407Y, 061409Y, 061449Y, 06144AY, 06150KY, 06154ZY, 061607Y, 06160ZY, 06164AY, 061749Y, 06174AY, 06174KY, 0618099, 06180K9, 06184J9, 06184JY, 06184Z9, 061909Y, 061J4JY, 06L34CZ, 0610075, 06100A6, 06100K5, 06100KY, 06100ZY, 061049Y, 06104A5, 06104A6, 06104JY, 06104Z6, 061107Y, 06110KY, 06110ZB, 061149Y, 06114A9, 06114AB, 06114AY, 06114ZB, 06140KY, 06140ZY, 06144ZY, 06150AY, 06150ZY, 061609Y, 061649Y, 06164JY, 06164KY, 06164ZY, 06170KY, 061807B, 06180JB, 06180KY, 06180ZY, 061847Y, 06184DY, 06184KY, 06190AY, 06194JY, 061B07Y, 061B0AY, 061B4ZY, 061J07Y, 061J4ZY, 06L33DZ, 06L34DZ, 0610096, 061009Y, 06100Z6, 0610476, 06104AY, 06104J5, 06104J6, 06104KY, 06110JY, 06110K9, 06110ZY, 0611499, 06114JB, 06114K9, 06114KB, 06114KY, 06114Z9, 06114ZY, 06120KY, 061247Y, 061249Y, 06124AY, 06150JY, 06154JY, 06160KY, 061747Y, 06174ZY, 0618079, 06180AY, 06180JY, 06180KB, 06180ZB, 06183DY, 061847B, 0618499, 06184AY, 06184K9, 06184ZY, 061907Y, 06194ZY, 061B0JY, 061B0KY, 061B47Y, 061B4JY, 061J0AY, 061J0ZY, 061J47Y, 06L30CZ, 06L30DZ, 06L33CZ, 06L33ZZ, 0610076, 0610095, 06100AY, 06100J6, 06100JY, 0610475, 061047Y, 0610495, 06104K6, 0611079, 061109B, 061109Y, 06110A9, 06110AB, 06110Z9, 061147B, 061149B, 061209Y, 06120AY, 06120JY, 06124JY, 06124KY, 06124ZY, 06140JY, 061447Y, 06144JY, 06144KY, 061547Y, 06154KY, 06160AY, 06160JY, 061707Y, 06170AY, 06170JY, 06170ZY, 06174JY, 06180A9, 06180AB, 061849Y, 06184A9, 06184KB, 06190JY, 06190KY, 06194AY, 06194KY, 061B09Y, 061B49Y, 061J0JY, 061J49Y, 061J4AY, 061J4KY, 06L34ZZ
Peptic Ulcer	ICD-9 Diagnosis Code (any position) is any of: 533, 533.0, 533.00, 533.01, 533.11, 533.20, 533.3, 533.31, 533.40, 533.6, 533.60, 533.61, 533.7, 533.71, 533.90, V12.71, 533.1, 533.10, 533.2, 533.21, 533.30, 533.4, 533.41, 533.5, 533.50, 533.51, 533.70, 533.9, 533.91<br>ICD-10 Diagnosis Code (any position) is any of: K27, Z87.11, K25.4, K25.7, K25.9, K26.1, K26.4, K26.5, K26.6, K26.7, K27.2, K27.5, K27.6, K27.7, K28.0, K28.4, K28.5, K28.7, K56.699, K25.1, K25.5, K26.0, K26.2, K26.3, K26.9, K27.0, K27.1, K27.3, K27.4, K27.9, K28.1, K28.2, K28.3, K28.6, K28.9
Obesity	HCPCS Procedure Code (any position) is any of: 43659, 43999, 43842, S2082, 43644, 43645, 43770, 43843, 43845, 43846, 43847<br>ICD-9 Procedure Code (any position) is any of: 43.89, 44.69, 44.31, 44.39, 44.95, 45.51, 45.91, 43.82, 44.38, 44.68<br>ICD-10 Procedure Code (any position) is any of: 0D160JB, 0D160KL, 0D160Z9, 0D164JL, 0D1687L, 0D168JB, 0D168Z9, 0D168ZL, 0D190ZA, 0D194J9, 0D194K9, 0D194ZA, 0D198K9, 0D198ZA, 0D1A0JB, 0D1A0ZB, 0D1A87A, 0D1A8JB, 0D1A8KA, 0D1A8KB, 0D1B4JB, 0D1B4KB, 0D1B8JB, 0D760DZ, 0DB63Z3, 0DB67ZZ, 0DB90ZZ, 0DM64ZZ, 0DN67ZZ, 0DQ64ZZ, 0DV60CZ, 0DV63CZ, 0D16079, 0D1607A, 0D1607L, 0D160ZL, 0D164J9, 0D164KA, 0D164KL, 0D1687A, 0D168J9, 0D168ZA, 0D190K9, 0D190KB, 0D19479, 0D1947B, 0D194JA, 0D194KB, 0D1987A, 0D198ZB, 0D1A07A, 0D1A0KB, 0D1A47A, 0D1A4KA, 0D1A4ZB, 0D1A8ZA, 0D1A8ZB, 0D1B0JB, 0D763DZ, 0D764ZZ, 0DB60Z3, 0DB67Z3, 0DB68Z3, 0DQ68ZZ, 0DU64KZ, 0DU677Z, 0DU67KZ, 0DU687Z, 0DU68JZ, 0DU68KZ, 0DV63ZZ, 0DV64CZ, 0DV64DZ, 0D164Z9, 0D168JL, 0D168KA, 0D168KB, 0D168ZB, 0D190Z9, 0D1947A, 0D194KA, 0D194ZB, 0D1987B, 0D1A07B, 0D1A0JA, 0D1A4JA, 0D1A4KB, 0D763ZZ, 0D764DZ, 0DB63ZZ, 0DBB0ZZ, 0DF67ZZ, 0DF68ZZ, 0DN60ZZ, 0DQ60ZZ, 0DU60KZ, 0DU647Z, 0DU64JZ, 0DV60ZZ, 0DV64ZZ, 0D1607B, 0D160JL, 0D160K9, 0D160KA, 0D164JB, 0D1687B, 0D1907B, 0D190J9, 0D190JB, 0D190ZB, 0D194Z9, 0D198J9, 0D198KA, 0D1A0ZA, 0D1A47B, 0D1A4JB, 0D1A4ZA, 0D1A8JA, 0D1B47B, 0D1B4ZB, 0D1B8KB, 0D1B8ZB, 0D760ZZ, 0DB64Z3, 0DM60ZZ, 0DN63ZZ, 0DN64ZZ, 0DN68ZZ, 0DU607Z, 0DU60JZ, 0DV60DZ, 0DV63DZ, 0D160J9, 0D160JA, 0D160KB, 0D160ZA, 0D160ZB, 0D16479, 0D1647A, 0D1647B,

© 2023 Lin KJ et al. *JAMA Network Open*.

	0D1647L, 0D164JA, 0D164K9, 0D164KB, 0D164ZA, 0D164ZB, 0D164ZL, 0D16879, 0D168JA, 0D168K9, 0D168KL, 0D19079, 0D1907A, 0D190JA, 0D190KA, 0D194JB, 0D19879, 0D198JA, 0D198JB, 0D198KB, 0D198Z9, 0D1A0KA, 0D1A87B, 0D1A8ZH, 0D1B07B, 0D1B0KB, 0D1B0ZB, 0D1B87B, 0D1B8ZH, 0DB60ZZ, 0DB80ZZ, 0DF60ZZ, 0DF63ZZ, 0DF64ZZ, 0DQ63ZZ, 0DQ67ZZ, 0DU67JZ, 0DV67ZZ, 0DV68ZZ<br>ICD-9 Diagnosis Code (any position) is any of: 539.89, 649.1, V85.36, V85.41, V85.42, 649.10, 649.11, 649.20, 649.23, V85.37, V85.44, 278.01, 278.03, 539, 649.13, 649.14, 649.2, 649.22, 649.24, V85.31, V85.34, V85.35, V85.43, 278.0, 278.00, 539.09, 539.8, 539.81, 649.12, 649.21, V85.38, V85.39, 539.0, 539.01, V85.30, V85.32, V85.33, V85.45<br>ICD-10 Diagnosis Code (any position) is any of: Z68.41, O99.210, O99.214, O99.844, Z68.35, Z68.38, Z68.43, Z68.45, O99.845, Z68.32, Z68.33, Z68.36, O99.212, O99.841, O99.842, Z68.34, Z68.37, Z68.42, O99.211, O99.213, O99.840, O99.843, Z68.30, Z68.31, Z68.39, Z68.44, O99.215, E66.01, E66.2<br>NDC Generic Name is any of: DEXFENFLURAMINE HCL, NALTREXONE HCL/BUPROPION HCL, PHENTERMINE HCL/TOPIRAMATE, LORCASERIN HCL, ORLISTAT, SIBUTRAMINE HCL M-HYDRATE, BENZPHETAMINE HCL, FENFLURAMINE HCL, DIETHYLPROPION HCL, PHENDIMETRAZINE TARTRATE, PHENTERMINE HCL
Cerebrovascular disease	ICD-9 Diagnosis Code (any position) is any of: 430, 431, 432, 432.0, 432.1, 432.9, 433, 433.0, 433.00, 433.01, 433.1, 433.10, 433.11, 433.2, 433.20, 433.21, 433.3, 433.30, 433.31, 433.8, 433.80, 433.81, 433.9, 433.90, 433.91, 434, 434.0, 434.00, 434.01, 434.1, 434.10, 434.11, 434.9, 434.90, 434.91, 435, 435.0, 435.1, 435.2, 435.3, 435.8, 435.9, 436, 437, 437.0, 437.1, 437.2, 437.3, 437.4, 437.5, 437.6, 437.7, 437.8, 437.9, 438, 438.0, 438.1, 438.10, 438.11, 438.12, 438.13, 438.14, 438.19, 438.2, 438.20, 438.21, 438.22, 438.3, 438.30, 438.31, 438.32, 438.4, 438.40, 438.41, 438.42, 438.5, 438.50, 438.51, 438.52, 438.53, 438.6, 438.7, 438.8, 438.81, 438.82, 438.83, 438.84, 438.85, 438.89, 438.9<br>ICD-10 Diagnosis Code (any position) is any of: I60.0, I60.01, I60.10, I60.11, I60.21, I60.22, I60.3, I60.31, I60.5, I60.51, I60.52, I60.6, I60.8, I61, I61.4, I61.8, I62.1, I63.011, I63.012, I63.031, I63.112, I63.12, I63.132, I63.139, I63.19, I63.2, I63.21, I63.212, I63.213, I63.219, I63.22, I63.231, I63.232, I63.233, I63.239, I63.29, I63.30, I63.31, I63.311, I63.32, I63.322, I63.329, I63.33, I63.339, I63.34, I63.341, I63.342, I63.349, I63.39, I63.40, I63.411, I63.419, I63.422, I63.429, I63.431, I63.49, I63.50, I63.51, I63.52, I63.523, I63.529, I63.531, I63.533, I63.541, I63.542, I63.543, I63.8, I63.9, I65.01, I65.09, I65.2, I65.22, I65.23, I65.29, I65.9, I66, I66.0, I66.02, I66.09, I66.11, I66.12, I66.13, I66.19, I66.2, I66.21, I66.22, I66.8, I67.3, I67.6, I67.7, I67.8, I67.81, I67.84, I67.841, I67.848, I67.9, I68, I68.0, I68.8, I69.01, I69.02, I69.021, I69.022, I69.03, I69.031, I69.032, I69.04, I69.041, I69.042, I69.043, I69.051, I69.052, I69.053, I69.059, I69.06, I69.061, I69.063, I69.064, I69.065, I69.069, I69.09, I69.090, I69.092, I69.098, I69.12, I69.121, I69.128, I69.132, I69.133, I69.141, I69.143, I69.15, I69.151, I69.153, I69.154, I69.159, I69.16, I69.163, I69.165, I69.19, I69.190, I69.192, I69.193, I69.2, I69.21, I69.222, I69.228, I69.233, I69.234, I69.239, I69.241, I69.242, I69.244, I69.252, I69.253, I69.259, I69.26, I69.262, I69.263, I69.264, I69.265, I69.29, I69.291, I69.292, I69.298, I69.31, I69.32, I69.320, I69.323, I69.331, I69.332, I69.333, I69.334, I69.34, I69.344, I69.349, I69.35, I69.353, I69.362, I69.363, I69.364, I69.365, I69.391, I69.392, I69.393, I69.8, I69.81, I69.820, I69.822, I69.823, I69.831, I69.833, I69.834, I69.84, I69.841, I69.843, I69.844, I69.849, I69.851, I69.852, I69.853, I69.861, I69.862, I69.864, I69.869, I69.89, I69.890, I69.898, I69.9, I69.91, I69.921, I69.922, I69.93, I69.931, I69.932, I69.934, I69.939, I69.94, I69.941, I69.942, I69.943, I69.949, I69.951, I69.952, I69.953, I69.959, I69.963, I69.964, I69.969, I69.991, I69.992, G45.0, G45.9, I60, I60.00, I60.02, I60.1, I60.12, I60.2, I60.20, I60.30, I60.32, I60.4, I60.50, I60.7, I60.9, I61.0, I61.1, I61.2, I61.3, I61.5, I61.6, I61.9, I62, I62.0, I62.00, I62.01, I62.02, I62.03, I62.9, I63, I63.0, I63.00, I63.01, I63.013, I63.019, I63.02, I63.03, I63.032, I63.033, I63.039, I63.09, I63.1, I63.10, I63.11, I63.111, I63.113, I63.119, I63.13, I63.131, I63.133, I63.20, I63.211, I63.23, I63.3, I63.312, I63.319, I63.321, I63.331, I63.332, I63.4, I63.41, I63.412, I63.42, I63.421, I63.43, I63.432, I63.439, I63.44, I63.441, I63.442, I63.449, I63.5, I63.511, I63.512, I63.513, I63.519, I63.521, I63.522, I63.53, I63.532, I63.539, I63.54, I63.549, I63.59, I63.6, I65, I65.0, I65.02, I65.03, I65.1, I65.21, I65.8, I66.01, I66.03, I66.1, I66.23, I66.29, I66.3, I66.9, I67, I67.0, I67.1, I67.2, I67.4, I67.5, I67.82, I67.83, I67.89, I68.2, I69, I69.0, I69.00, I69.020, I69.023, I69.028, I69.033, I69.034, I69.039, I69.044, I69.049, I69.05, I69.054, I69.062, I69.091, I69.093, I69.1, I69.10, I69.11, I69.120, I69.122, I69.123, I69.13, I69.131, I69.134, I69.139, I69.14, I69.142, I69.144, I69.149, I69.152, I69.161, I69.162, I69.164, I69.169, I69.191, I69.198, I69.20, I69.22, I69.220, I69.221, I69.223, I69.23, I69.231, I69.232, I69.24, I69.243, I69.249, I69.25, I69.251, I69.254, I69.261,

	I69.269, I69.290, I69.293, I69.3, I69.30, I69.321, I69.322, I69.328, I69.33, I69.339, I69.341, I69.342, I69.343, I69.351, I69.352, I69.354, I69.359, I69.36, I69.361, I69.369, I69.39, I69.390, I69.398, I69.80, I69.82, I69.821, I69.828, I69.83, I69.832, I69.839, I69.842, I69.85, I69.854, I69.859, I69.86, I69.863, I69.865, I69.891, I69.892, I69.893, I69.90, I69.92, I69.920, I69.923, I69.928, I69.933, I69.944, I69.95, I69.954, I69.96, I69.961, I69.962, I69.965, I69.99, I69.990, I69.993, I69.998
ED Visit	HCPCS Procedure Code (any position) is any of: 99281, 99282, 99283, 99284, 99285, 99288
Home health day	The occurrence of **Home Health Agency** claim
Hospitalizations	The occurrence of **Inpatient** claim

Comorbidity Scores

Claims-based Frailty Index: https://dataverse.harvard.edu/dataverse/cfi

Combined Comorbidity Score: https://www.drugepi.org/dope/software#Combined1

CHADS-VASc Score Component	Codes/Definition	Weight
Congestive Heart Failure	ICD-9 Diagnosis Code in any position: 398.91, 404.01, 404.13, 428.0, 428.1, 428.2, 428.21, 428.3, 428.31, 428.33, 428.4, 428.41, 428.9, 402.01, 402.11, 402.91, 404.93, 428, 428.22, 428.23, 428.32, 428.42, 428.43<br>ICD-10 Diagnosis Code in any position: I50.20, I50.22, I50.33, I50.82, I50.89, I52, I50.1, I50.21, I50.23, I50.30, I50.31, I50.32, I50.40, I50.41, I50.42, I50.43, I50.810, I50.811, I50.812, I50.813, I50.814, I50.83, I50.84, I50.9, I51.9	1
Hypertension	ICD-9 Diagnosis Code in any position: 401, 401.9, 403.0, 403.1, 404.1, 404.91, 405.0, 405.09, 405.11, 405.19, 405.9, 405.99, 401.0, 401.1, 402.01, 402.11, 402.91, 403.9, 404.0, 404.03, 404.04, 404.11, 404.9, 405, 405.01, 405.1, 405.91<br>ICD-10 Diagnosis Code in any position: I09.81, I15.0, I15.8, I16.0, I16.1, I26.01, I26.99, I70.211, I70.219, I70.221, I70.223, I70.228, I70.229, I70.231, I70.233, I70.234, I70.238, I70.239, I70.242, I70.245, I70.249, I70.25, I70.262, I70.268, I70.269, I70.293, I70.298, I70.299, I70.403, I70.411, I70.413, I70.419, I70.421, I70.429, I70.431, I70.432, I70.433, I70.434, I70.438, I70.439, I70.443, I70.444, I70.448, I70.449, I70.45, I70.461, I70.462, I70.468, I70.493, I70.499, I70.501, I70.502, I70.503, I70.508, I70.511, I70.513, I70.518, I70.519, I70.522, I70.529, I70.531, I70.533, I70.535, I70.542, I70.544, I70.548, I70.549, I70.55, I70.562, I70.563, I70.568, I70.569, I70.592, I70.593, I70.598, I70.599, I73.9, I74.11, I74.9, T80.0XXA, T81.718A, T81.72XA, T82.818A, I10, I11.0, I13.0, I13.2, I15.1, I15.2, I15.9, I16.9, I26.09, I26.90, I70.212, I70.213, I70.218, I70.222, I70.232, I70.235, I70.241, I70.243, I70.244, I70.248, I70.261, I70.263, I70.291, I70.292, I70.401, I70.402, I70.408, I70.409, I70.412, I70.418, I70.422, I70.423, I70.428, I70.435, I70.441, I70.442, I70.445, I70.463, I70.469, I70.491, I70.492, I70.498, I70.509, I70.512, I70.521, I70.523, I70.528, I70.532, I70.534, I70.538, I70.539, I70.541, I70.543, I70.545, I70.561, I70.591, N26.2, T82.817A	1
Age	Age 65 to 74	1
	Age >= 75	2
Diabetes	ICD-9 Diagnosis Code in any position: 249, 249.0, 249.00, 249.10, 249.11, 249.2, 249.3, 249.40, 249.41, 249.5, 249.50, 249.51, 249.60, 249.61, 249.81, 249.9, 249.91, 250, 250.0, 250.01, 250.02, 250.1, 250.10, 250.11, 250.13, 250.20, 250.32, 250.42, 250.51, 250.6, 250.61, 250.63, 250.70, 250.72, 250.73, 250.8, 250.83, 249.01, 249.1, 249.20, 249.21, 249.30, 249.31, 249.4, 249.6, 249.7, 249.70, 249.71, 249.8, 249.80, 249.90, 250.00, 250.03, 250.12, 250.2, 250.21, 250.22, 250.23, 250.3, 250.30, 250.31, 250.33, 250.4, 250.40, 250.41, 250.43, 250.5, 250.50, 250.52, 250.53, 250.60, 250.62, 250.7, 250.71, 250.80, 250.81, 250.82, 250.9, 250.90, 250.91, 250.92, 250.93	1

© 2023 Lin KJ et al. *JAMA Network Open*.

	ICD-10 Diagnosis Code in any position: E08.00, E08.10, E08.11, E08.21, E08.29, E08.311, E08.319, E08.3211, E08.3212, E08.3291, E08.3292, E08.3392, E08.3393, E08.3413, E08.3419, E08.3491, E08.3493, E08.3499, E08.3512, E08.3513, E08.3522, E08.3523, E08.3529, E08.3533, E08.3539, E08.3543, E08.3549, E08.3551, E08.3552, E08.36, E08.37X3, E08.39, E08.42, E08.52, E08.610, E08.620, E08.621, E08.622, E08.628, E08.630, E08.641, E08.8, E09.01, E09.29, E09.3212, E09.3213, E09.3219, E09.3291, E09.3292, E09.3293, E09.3313, E09.339, E09.3419, E09.3493, E09.3499, E09.3511, E09.3512, E09.3513, E09.3519, E09.3522, E09.3523, E09.3532, E09.3542, E09.3552, E09.3553, E09.3593, E09.36, E09.37X1, E09.39, E09.42, E09.43, E09.51, E09.52, E09.59, E09.610, E09.620, E09.622, E09.630, E09.649, E09.69, E09.8, E10.22, E10.29, E10.3211, E10.3212, E10.3291, E10.3299, E10.3311, E10.3312, E10.3319, E10.3391, E10.3392, E10.3399, E10.3419, E10.3491, E10.3492, E10.3511, E10.3512, E10.3519, E10.3521, E10.3522, E10.3523, E10.3529, E10.3532, E10.3541, E10.3543, E10.3552, E10.3553, E10.3592, E10.3599, E10.37X1, E10.37X3, E10.37X9, E10.39, E10.40, E10.43, E10.49, E10.51, E10.52, E10.59, E10.610, E10.620, E10.622, E10.628, E10.649, E10.69, E10.8, E10.9, E11.00, E11.10, E11.11, E11.22, E11.319, E11.3212, E11.3293, E11.3312, E11.3313, E11.3391, E11.3393, E11.3399, E11.3411, E11.3413, E11.3419, E11.3491, E11.3492, E11.3511, E11.3512, E11.3521, E11.3522, E11.3523, E11.3529, E11.3531, E11.3532, E11.3533, E11.3551, E11.3552, E11.3553, E11.3593, E11.37X1, E11.37X2, E11.37X3, E11.37X9, E11.40, E11.43, E11.44, E11.49, E11.52, E11.59, E11.618, E11.622, E11.628, E11.630, E11.641, E11.649, E11.9, E13.00, E13.21, E13.22, E13.29, E13.311, E13.319, E13.3211, E13.3291, E13.3292, E13.3312, E13.3319, E13.3391, E13.3392, E13.3399, E13.3411, E13.3412, E13.3493, E13.3511, E13.3521, E13.3522, E13.3523, E13.3531, E13.3533, E13.3539, E13.3542, E13.3549, E13.3551, E13.3552, E13.3553, E13.3559, E13.3592, E13.3599, E13.36, E13.37X1, E13.37X2, E13.37X9, E13.40, E13.51, E13.52, E13.59, E13.610, E13.618, E13.621, E13.622, E13.628, E13.630, E13.641, E13.649, E13.65, E08.01, E08.22, E08.3213, E08.3219, E08.3293, E08.3299, E08.3311, E08.3312, E08.3313, E08.3319, E08.3391, E08.3399, E08.3411, E08.3412, E08.3492, E08.3511, E08.3519, E08.3521, E08.3531, E08.3532, E08.3541, E08.3542, E08.3553, E08.3559, E08.3591, E08.3592, E08.3593, E08.3599, E08.37X1, E08.37X2, E08.37X9, E08.40, E08.41, E08.43, E08.44, E08.49, E08.51, E08.59, E08.618, E08.638, E08.649, E08.65, E08.69, E08.9, E09.00, E09.10, E09.11, E09.21, E09.22, E09.311, E09.319, E09.3211, E09.3299, E09.3311, E09.3312, E09.3319, E09.3391, E09.3393, E09.3399, E09.3412, E09.3413, E09.3491, E09.3533, E09.3551, E09.3591, E09.3592, E09.3599, E09.37X9, E09.40, E09.41, E09.44, E09.49, E09.618, E09.621, E09.628, E09.638, E09.641, E09.65, E09.9, E10.10, E10.11, E10.21, E10.311, E10.319, E10.3213, E10.3219, E10.3292, E10.3293, E10.3313, E10.3393, E10.3411, E10.3412, E10.3413, E10.3493, E10.3499, E10.3513, E10.3531, E10.3533, E10.3539, E10.3542, E10.3549, E10.3551, E10.3559, E10.3591, E10.3593, E10.36, E10.37X2, E10.41, E10.42, E10.44, E10.618, E10.621, E10.630, E10.638, E10.641, E10.65, E11.01, E11.21, E11.29, E11.311, E11.3211, E11.3213, E11.3219, E11.3291, E11.3292, E11.3299, E11.3311, E11.3319, E11.3392, E11.3412, E11.3493, E11.3499, E11.3513, E11.3519, E11.3539, E11.3541, E11.3542, E11.3543, E11.3549, E11.3559, E11.3591, E11.3592, E11.3599, E11.36, E11.39, E11.41, E11.42, E11.51, E11.610, E11.620, E11.621, E11.638, E11.65, E11.69, E11.8, E13.01, E13.10, E13.11, E13.3212, E13.3213, E13.3219, E13.3293, E13.3299, E13.3311, E13.3313, E13.3393, E13.3413, E13.3419, E13.3491, E13.3492, E13.3499, E13.3512, E13.3513, E13.3519, E13.3529, E13.3532, E13.3541, E13.3543, E13.3591, E13.3593, E13.37X3, E13.39, E13.41, E13.42, E13.43, E13.44, E13.49, E13.620, E13.638, E13.69, E13.8, E13.9	

© 2023 Lin KJ et al. *JAMA Network Open*.

Stroke/TIA/Thromboembolism	ICD-9 Diagnosis Code in any position: 430, 432, 432.9, 433, 433.00, 433.01, 433.11, 433.2, 433.21, 433.3, 433.30, 433.31, 433.9, 433.90, 434.01, 434.11, 435.1, 435.9, 437.1, 453.4, 453.50, 453.51, 453.8, 453.83, 453.85, 453.87, 634.61, 635.60, 635.61, 635.62, 636.60, 636.61, 636.62, 637.61, 637.62, 638.6, 639.6, 673.21, 673.23, 673.24, 673.81, 673.83, 997.02, 415.1, 415.11, 415.12, 415.13, 415.19, 431, 432.0, 432.1, 433.0, 433.1, 433.10, 433.20, 433.8, 433.80, 433.81, 433.91, 434.91, 435, 435.0, 435.2, 435.3, 435.8, 437.3, 451.1, 451.11, 451.19, 451.2, 453.40, 453.41, 453.42, 453.5, 453.52, 453.81, 453.82, 453.84, 453.86, 453.89, 453.9, 634.60, 634.62, 637.60, 673.20, 673.22, 673.80, 673.82, 673.84<br>ICD-10 Diagnosis Code in any position: G45.1, G45.2, G46.2, G97.31, G97.32, I26.01, I26.02, I26.99, I60.01, I60.10, I60.11, I60.31, I60.51, I60.52, I60.6, I60.8, I61.4, I61.8, I62.1, I63.011, I63.012, I63.031, I63.112, I63.12, I63.132, I63.139, I63.19, I63.212, I63.213, I63.219, I63.22, I63.231, I63.232, I63.233, I63.239, I63.29, I63.30, I63.311, I63.322, I63.329, I63.339, I63.341, I63.342, I63.349, I63.39, I63.40, I63.411, I63.419, I63.422, I63.429, I63.431, I63.433, I63.49, I63.50, I63.523, I63.529, I63.531, I63.533, I63.541, I63.542, I63.543, I63.8, I63.9, I65.01, I65.09, I65.22, I65.23, I65.29, I65.9, I67.81, I67.841, I67.848, I80.10, I80.11, I80.13, I80.209, I80.222, I80.223, I80.239, I80.291, I80.293, I80.299, I82.210, I82.401, I82.402, I82.411, I82.412, I82.413, I82.421, I82.429, I82.432, I82.433, I82.439, I82.491, I82.492, I82.493, I82.499, I82.4Y1, I82.4Z1, I82.4Z2, I82.4Z3, I82.4Z9, I82.503, I82.509, I82.511, I82.512, I82.519, I82.522, I82.523, I82.529, I82.531, I82.532, I82.533, I82.543, I82.549, I82.591, I82.592, I82.593, I82.5Y2, I82.5Y9, I82.5Z1, I82.602, I82.611, I82.613, I82.619, I82.621, I82.A19, I82.B11, I82.C11, I82.C19, I97.811, O03.7, O04.7, O88.219, O88.23, O88.811, O88.813, O88.82, O88.83, T80.0XXA, T81.718A, T81.72XA, T82.818A, G45.0, G45.8, G45.9, G46.0, G46.1, I26.09, I26.90, I26.92, I60.00, I60.02, I60.12, I60.2, I60.30, I60.32, I60.4, I60.50, I60.7, I60.9, I61.0, I61.1, I61.2, I61.3, I61.5, I61.6, I61.9, I62.00, I62.01, I62.02, I62.03, I62.9, I63.00, I63.013, I63.019, I63.02, I63.032, I63.033, I63.039, I63.09, I63.10, I63.111, I63.113, I63.119, I63.131, I63.133, I63.20, I63.211, I63.312, I63.313, I63.319, I63.321, I63.323, I63.331, I63.332, I63.333, I63.343, I63.412, I63.413, I63.421, I63.423, I63.432, I63.439, I63.441, I63.442, I63.443, I63.449, I63.511, I63.512, I63.513, I63.519, I63.521, I63.522, I63.532, I63.539, I63.549, I63.59, I63.6, I65.02, I65.03, I65.1, I65.21, I65.8, I67.1, I67.82, I67.89, I80.12, I80.201, I80.202, I80.203, I80.221, I80.229, I80.231, I80.232, I80.233, I80.292, I80.3, I82.290, I82.403, I82.409, I82.419, I82.422, I82.423, I82.431, I82.441, I82.442, I82.443, I82.449, I82.4Y2, I82.4Y3, I82.4Y9, I82.501, I82.502, I82.513, I82.521, I82.539, I82.541, I82.542, I82.599, I82.5Y1, I82.5Y3, I82.5Z2, I82.5Z3, I82.5Z9, I82.601, I82.603, I82.609, I82.612, I82.622, I82.623, I82.629, I82.890, I82.90, I82.91, I82.A11, I82.A12, I82.A13, I82.B12, I82.B13, I82.B19, I82.C12, I82.C13, I97.810, I97.820, I97.821, O03.2, O07.2, O08.2, O88.211, O88.212, O88.213, O88.22, O88.812, O88.819, T82.817A	2
Vascular disease	ICD-9 Diagnosis Code in any position: 250.70, 250.72, 250.73, 410, 410.0, 410.00, 410.01, 410.12, 410.22, 410.31, 410.40, 410.52, 410.60, 410.70, 410.71, 410.72, 410.8, 410.80, 410.82, 410.91, 412, 414, 414.04, 414.05, 414.1, 414.10, 414.4, 414.8, 440.20, 440.22, 440.24, 440.30, 443.81, 443.9, 444.0, 444.2, 444.9, 250.7, 250.71, 410.02, 410.1, 410.10, 410.11, 410.2, 410.20, 410.21, 410.3, 410.30, 410.32, 410.4, 410.41, 410.42, 410.5, 410.50, 410.51, 410.6, 410.61, 410.62, 410.7, 410.81, 410.9, 410.90, 410.92, 414.0, 414.00, 414.01, 414.02, 414.03, 414.06, 414.07, 414.11, 414.12, 414.19, 414.2, 414.3, 414.9, 440.0, 440.21, 440.23, 440.29, 440.31, 440.32, 444.1, 444.<br>ICD-10 Diagnosis Code in any position: E08.52, E09.51, E09.52, E10.51, E10.52, E10.59, E11.52, E11.59, E13.51, E13.52, E13.59, I21.01, I21.11, I21.4, I22.0, I22.1, I22.2, I22.8, I22.9, I25.10, I25.110, I25.119, I25.3, I25.41, I25.42, I25.5, I25.701, I25.709, I25.718, I25.720, I25.721,	1

© 2023 Lin KJ et al. *JAMA Network Open*.

	I25.728, I25.729, I25.730, I25.731, I25.750, I25.751, I25.760, I25.790, I25.798, I25.799, I25.811, I25.82, I25.83, I25.89, I73.9, I79.1, I79.8, E08.51, E10.65, E11.51, E11.65, I21.02, I21.09, I21.19, I21.21, I21.29, I21.3, I21.9, I21.A1, I21.A9, I25.111, I25.118, I25.2, I25.6, I25.700, I25.708, I25.710, I25.711, I25.719, I25.738, I25.739, I25.758, I25.759, I25.761, I25.768, I25.769, I25.791, I25.810, I25.812, I25.84, I25.9, I70.0	
Gender	Gender is recorded as **Female**	1

HAS-BLED Score Component	Codes/Definition	Weights
Age	Age > 65	1
Abnormal liver function	ICD-9 Diagnosis Code in any position: 794.8<br>ICD-10 Diagnosis Code in any position: R94.5	1
Abnormal renal function	ICD-9 Diagnosis Code in any position: 794.4<br>ICD-10 Diagnosis Code in any position: R94.4	1
Stroke	ICD-9 Diagnosis Code in any position: 433.01, 433.21, 433.81, 433.91, 434.11, 433.11, 433.31, 434.01, 434.91<br>ICD-10 Diagnosis Code in any position: I63.019, I63.032, I63.113, I63.119, I63.132, I63.133, I63.20, I63.212, I63.22, I63.231, I63.232, I63.233, I63.239, I63.30, I63.312, I63.321, I63.332, I63.341, I63.343, I63.349, I63.39, I63.411, I63.413, I63.419, I63.421, I63.432, I63.433, I63.439, I63.443, I63.49, I63.512, I63.521, I63.522, I63.523, I63.529, I63.531, I63.542, I63.549, I63.8, I63.00, I63.011, I63.012, I63.013, I63.02, I63.031, I63.033, I63.039, I63.09, I63.10, I63.111, I63.112, I63.12, I63.131, I63.139, I63.19, I63.211, I63.213, I63.219, I63.29, I63.311, I63.313, I63.319, I63.322, I63.323, I63.329, I63.331, I63.333, I63.339, I63.342, I63.40, I63.412, I63.422, I63.423, I63.429, I63.431, I63.441, I63.442, I63.449, I63.50, I63.511, I63.513, I63.519, I63.532, I63.533, I63.539, I63.541, I63.543, I63.59, I63.6, I63.9	1
Hypertension	ICD-9 Diagnosis Code in any position: 401.1, 402.0, 402.00, 402.91, 403.01, 403.11, 403.9, 403.90, 404.00, 404.01, 404.02, 404.03, 404.9, 404.90, 404.93, 405.09, 405.1, 405.11, 405.9, 405.91, 401.0, 401.9, 402.01, 402.1, 402.10, 402.11, 402.9, 402.90, 403.0, 403.00, 403.1, 403.10, 403.91, 404.0, 404.1, 404.10, 404.11, 404.12, 404.13, 404.91, 404.92, 405.0, 405.01, 405.19, 405.99<br>ICD-10 Diagnosis Code in any position: I15.1, I15.2, I15.8, I16.0, I16.1, I16.9, N26.2, I10, I11.0, I11.9, I12.0, I12.9, I13.0, I13.10, I13.11, I13.2, I15.0, I15.9	1
Bleeding	ICD-9 Diagnosis Code in any position: 280.0, 280.8, 281.0, 281.1, 281.2, 281.3, 281.8, 281.9, 282.1, 282.41, 282.49, 282.61, 282.62, 282.63, 282.69, 282.8, 283.2, 283.9, 284.0, 284.09, 284.1, 284.19, 285.0, 285.1, 285.2, 285.3, 285.8, 285.9, 280.1, 280.9, 281.4, 282.0, 282.2, 282.3, 282.4, 282.40, 282.42, 282.43, 282.44, 282.45, 282.46, 282.47, 282.5, 282.6, 282.60, 282.64, 282.68, 282.7, 282.9, 283.0, 283.1, 283.10, 283.11, 283.19, 284.01, 284.11, 284.12, 284.2, 284.8, 284.81, 284.89, 284.9, 285.21, 285.22, 285.29<br>ICD-10 Diagnosis Code in any position: D50.0, D50.9, D51.0, D51.1, D51.2, D51.3, D51.8, D51.9, D52.8, D53.0, D55.2, D55.8, D56.0, D57.01, D57.1, D57.219, D57.411, D57.811, D57.812, D58.1, D59.0, D59.3, D59.4, D60.0, D60.1, D61.01, D61.1, D61.818, D61.82, D63.8, D64.0, D64.1, D64.4, D64.81, D64.89, D50.1, D50.8, D52.0, D52.1, D52.9, D53.1, D53.2, D53.8, D53.9, D55.0, D55.1, D55.3, D55.9, D56.1, D56.2, D56.3, D56.4, D56.5, D56.8, D56.9, D57.00, D57.02, D57.20, D57.211, D57.212, D57.3, D57.40, D57.412, D57.419, D57.80, D57.819, D58.0, D58.2, D58.8, D58.9, D59.1, D59.2, D59.5, D59.6, D59.8, D59.9, D60.8, D60.9, D61.09, D61.2, D61.3, D61.810, D61.811, D61.89, D61.9, D63.0, D63.1, D64.2, D64.3, D64.9, D62	1
Drug or alcohol abuse	ICD-9 Diagnosis Code in any position: 303.93, 305.03, V11.3, 303.00, 303.01, 303.02, 303.03, 303.90, 303.91, 303.92, 305.00, 305.01, 305.02	1

	ICD-10 Diagnosis Code in any position: F10.11, F10.129, F10.20, F10.21, F10.10, F10.120, F10.220, F10.229, Z65.8	

© 2023 Lin KJ et al. *JAMA Network Open*.

eFigure 1. Selection of Study Population for the Warfarin vs Apixaban Cohort Pooled Across Medicare, Optum, and MarketScan Populations

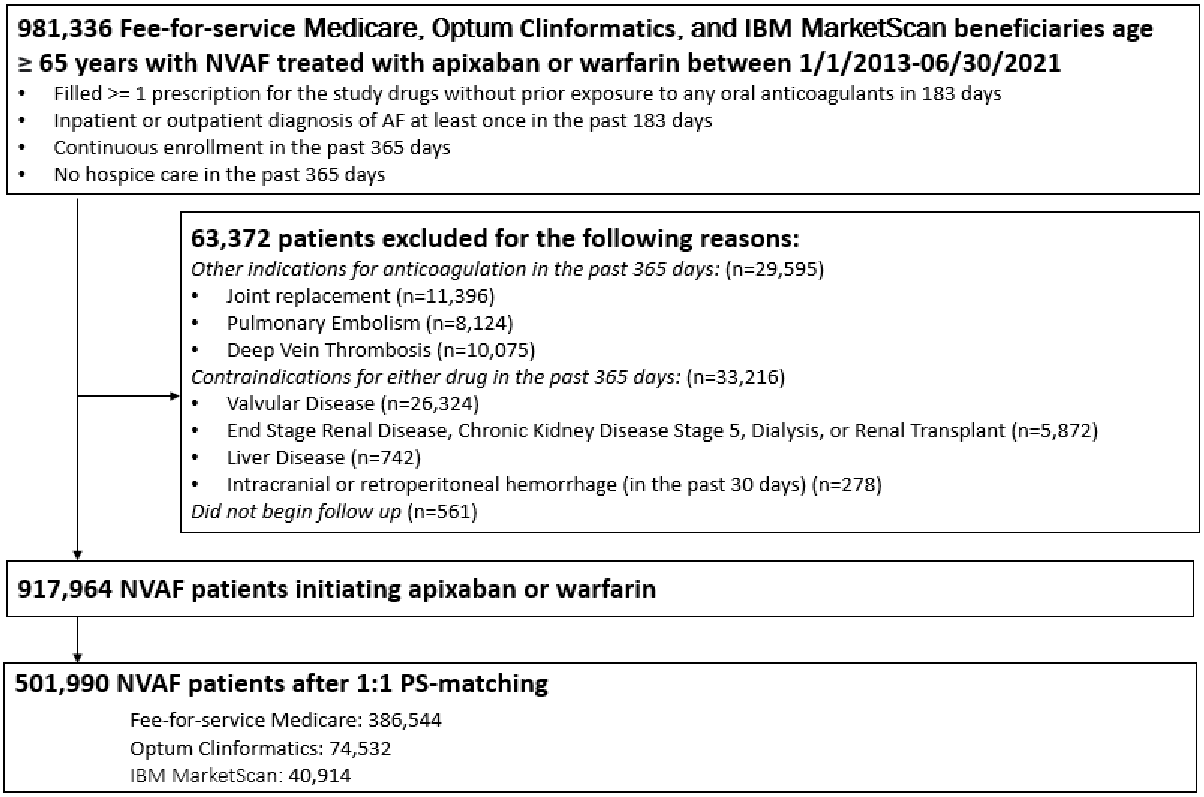

Abbreviations: NVAF, nonvalvular atrial fibrillation; PS, propensity score

© 2023 Lin KJ et al. *JAMA Network Open*.

eFigure 2. Selection of Study Population for the Dabigatran vs Apixaban Cohort Pooled Across Medicare, Optum, and MarketScan Populations

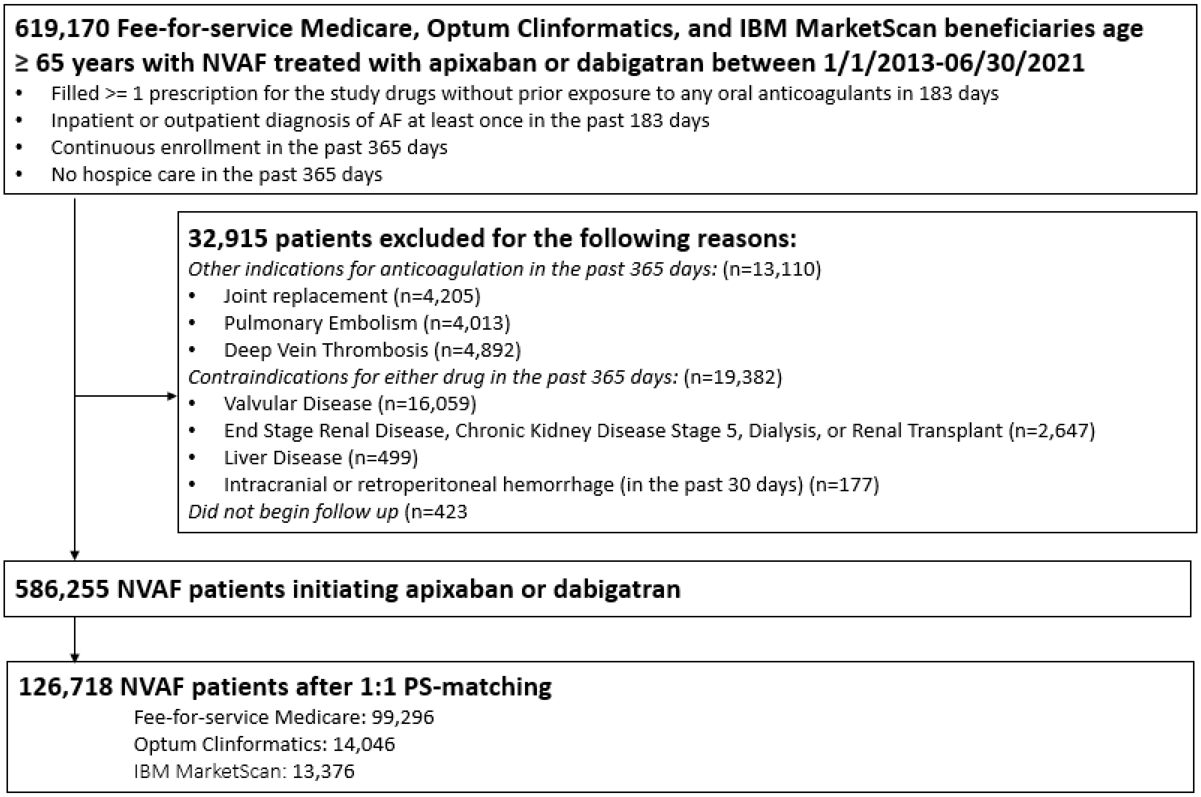

Abbreviations: NVAF, nonvalvular atrial fibrillation; PS, propensity score

© 2023 Lin KJ et al. *JAMA Network Open*.

eFigure 3. Selection of Study Population for the Rivaroxaban vs Apixaban Cohort Pooled Across Medicare, Optum, and MarketScan Populations

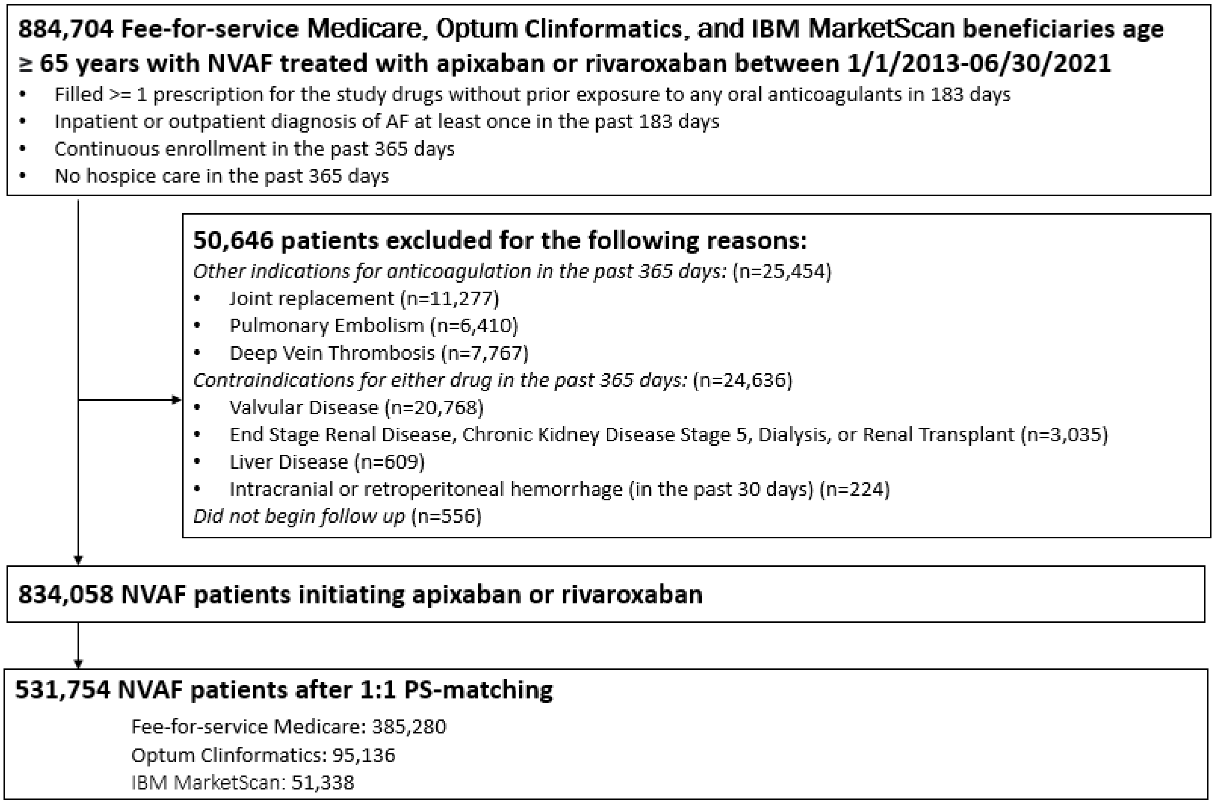

Abbreviations: NVAF, nonvalvular atrial fibrillation; PS, propensity score

© 2023 Lin KJ et al. *JAMA Network Open*.

eFigure 4. Sensitivity Analyses for Warfarin vs Apixaban in Older Adults With Atrial Fibrillation in the Medicare Population

Composite Major Bleeding or Ischemic Stroke

	Warfarin IR/1000 PY	Apixaban IR/1000 PY	Hazard Ratio (95% CI)	Rate Difference (95% CI)
Overall	57.4	38.7	1.5 (1.4–1.6)	18.7 (16.6–20.7)
Dementia with RX	85.0	55.9	1.5 (1.2–1.9)	29.1 (13.5–44.8)
Dementia without RX	101.9	69.7	1.5 (1.3–1.7)	32.3 (20.3–44.2)
No Dementia	53.8	36.6	1.5 (1.4–1.5)	17.2 (15.1–19.2)
P heterogeneity			0.95	0.02

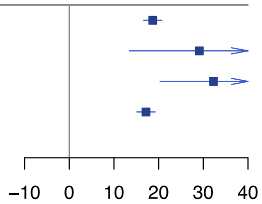

Major Bleeding

	Warfarin IR/1000 PY	Apixaban IR/1000 PY	Hazard Ratio (95% CI)	Rate Difference (95% CI)
Overall	42.7	26.4	1.6 (1.5–1.7)	16.3 (14.6–18.0)
Dementia with RX	60.6	40.6	1.5 (1.1–1.9)	20.0 (6.7–33.2)
Dementia without RX	71.7	44.9	1.6 (1.3–1.9)	26.8 (17.0–36.7)
No Dementia	40.2	25.1	1.6 (1.5–1.7)	15.2 (13.4–16.9)
P heterogeneity			0.87	0.06

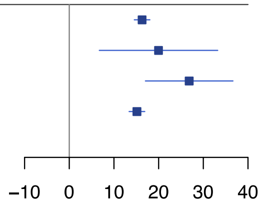

Ischemic Stroke

	Warfarin IR/1000 PY	Apixaban IR/1000 PY	Hazard Ratio (95% CI)	Rate Difference (95% CI)
Overall	14.2	11.1	1.3 (1.2–1.4)	3.1 (2.1–4.2)
Dementia with RX	21.7	14.6	1.5 (1.0–2.3)	7.0 (–0.9–14.9)
Dementia without RX	28.1	22.4	1.3 (1.0–1.6)	5.7 (–0.8–12.1)
No Dementia	13.2	10.4	1.3 (1.2–1.4)	2.8 (1.8–3.9)
P heterogeneity			0.79	0.41

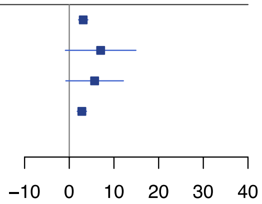

All–Cause Mortality

	Warfarin IR/1000 PY	Apixaban IR/1000 PY	Hazard Ratio (95% CI)	Rate Difference (95% CI)
Overall	127.3	116.5	1.1 (1.1–1.1)	10.9 (7.6–14.1)
Dementia with RX	315.1	268.8	1.2 (1.1–1.3)	46.3 (14.7–77.9)
Dementia without RX	414.5	375.6	1.1 (1.0–1.2)	38.9 (13.6–64.3)
No Dementia	104.4	96.3	1.1 (1.1–1.1)	8.1 (5.0–11.2)
P heterogeneity			0.40	<0.01

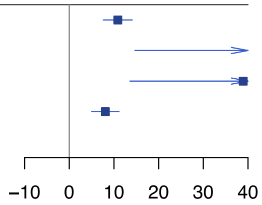

Abbreviations: CI, confidence interval; IR, incidence rate; PY, person-years; Rx, medications for dementia, including donepezil, rivastigmine, galantamine, and memantine.

© 2023 Lin KJ et al. *JAMA Network Open*.

eFigure 5. Sensitivity Analyses for Dabigatran vs Apixaban in Older Adults With Atrial Fibrillation In the Medicare Population

Composite Major Bleeding or Ischemic Stroke

	Dabigatran IR/1000 PY	Apixaban IR/1000 PY	Hazard Ratio (95% CI)	Rate Difference (95% CI)
Overall	41.5	33.6	1.2 (1.1–1.4)	7.9 (4.4–11.4)
Dementia with RX	83.6	47.6	1.8 (1.1–2.8)	36.0 (5.5–66.5)
Dementia without RX	83.3	59.1	1.4 (1.0–2.0)	24.3 (0.2–48.4)
No Dementia	39.0	30.9	1.3 (1.1–1.4)	8.1 (4.6–11.7)
P heterogeneity			0.90	0.09

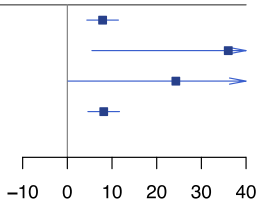

Major Bleeding

	Dabigatran IR/1000 PY	Apixaban IR/1000 PY	Hazard Ratio (95% CI)	Rate Difference (95% CI)
Overall	29.6	22.7	1.3 (1.2–1.5)	6.9 (4.0–9.9)
Dementia with RX	64.8	32.9	2.0 (1.1–3.5)	31.9 (5.6–58.2)
Dementia without RX	59.5	38.9	1.5 (1.0–2.3)	20.6 (0.6–40.6)
No Dementia	27.7	20.6	1.3 (1.2–1.5)	7.1 (4.2–10.0)
P heterogeneity			0.91	0.08

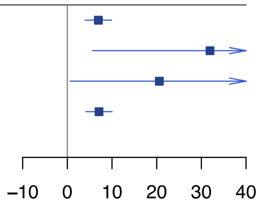

Ischemic Stroke

	Dabigatran IR/1000 PY	Apixaban IR/1000 PY	Hazard Ratio (95% CI)	Rate Difference (95% CI)
Overall	11.3	9.9	1.1 (1.0–1.4)	1.4 (–0.5–3.3)
Dementia with RX	22.0	9.1	2.4 (0.9–6.9)	12.9 (–1.9–27.7)
Dementia without RX	23.2	16.7	1.4 (0.7–2.6)	6.4 (–6.3–19.1)
No Dementia	10.6	8.9	1.2 (1.0–1.5)	1.7 (–0.1–3.6)
P heterogeneity			0.89	0.27

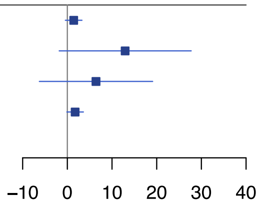

All–Cause Mortality

	Dabigatran IR/1000 PY	Apixaban IR/1000 PY	Hazard Ratio (95% CI)	Rate Difference (95% CI)
Overall	82.6	84.6	1.0 (0.9–1.0)	–1.9 (–7.2–3.3)
Dementia with RX	228.4	205.1	1.1 (0.9–1.4)	23.3 (–31.7–78.4)
Dementia without RX	311.8	308.0	1.0 (0.9–1.2)	3.9 (–46.0–53.8)
No Dementia	67.7	69.1	1.0 (0.9–1.1)	–1.5 (–6.4–3.5)
P heterogeneity			0.92	0.67

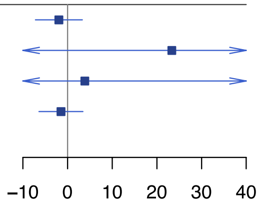

Abbreviations: CI, confidence interval; IR, incidence rate; PY, person-years; Rx, medications for dementia, including donepezil, rivastigmine, galantamine, and memantine.

© 2023 Lin KJ et al. *JAMA Network Open*.

eFigure 6. Sensitivity Analyses for Rivaroxaban vs Apixaban in Older Adults With Atrial Fibrillation In the Medicare Population

Composite Major Bleeding or Ischemic Stroke

	Rivaroxaban IR/1000 PY	Apixaban IR/1000 PY	Hazard Ratio (95% CI)	Rate Difference (95% CI)
Overall	54.1	34.5	1.6 (1.5–1.6)	19.6 (17.6–21.6)
Dementia with RX	92.0	52.4	1.8 (1.4–2.2)	39.7 (23.8–55.5)
Dementia without RX	86.4	71.1	1.2 (1.0–1.4)	15.4 (3.0–27.7)
No Dementia	51.2	31.4	1.6 (1.6–1.7)	19.7 (17.7–21.7)
P heterogeneity			<0.01	0.04

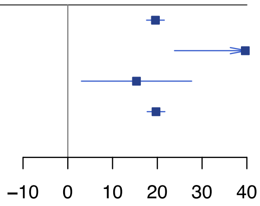

Major Bleeding

	Rivaroxaban IR/1000 PY	Apixaban IR/1000 PY	Hazard Ratio (95% CI)	Rate Difference (95% CI)
Overall	41.9	23.7	1.8 (1.7–1.9)	18.2 (16.5–19.9)
Dementia with RX	69.5	35.4	2.0 (1.5–2.6)	34.0 (20.6–47.5)
Dementia without RX	66.7	48.2	1.4 (1.2–1.7)	18.5 (8.0–29.0)
No Dementia	39.7	21.7	1.8 (1.7–1.9)	18.0 (16.3–19.7)
P heterogeneity			0.01	0.07

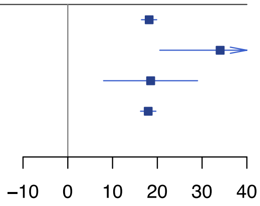

Ischemic Stroke

	Rivaroxaban IR/1000 PY	Apixaban IR/1000 PY	Hazard Ratio (95% CI)	Rate Difference (95% CI)
Overall	11.4	9.7	1.2 (1.1–1.3)	1.7 (0.7–2.7)
Dementia with RX	20.6	15.4	1.3 (0.9–2.1)	5.2 (−2.6–13.1)
Dementia without RX	19.7	20.9	0.9 (0.7–1.3)	−1.3 (−7.5–5.0)
No Dementia	10.6	8.7	1.2 (1.1–1.3)	1.9 (0.9–2.9)
P heterogeneity			0.25	0.43

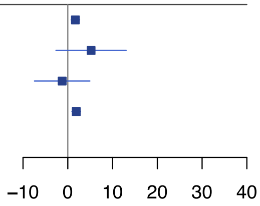

All–Cause Mortality

	Rivaroxaban IR/1000 PY	Apixaban IR/1000 PY	Hazard Ratio (95% CI)	Rate Difference (95% CI)
Overall	105.4	94.6	1.1 (1.1–1.2)	10.8 (7.8–13.8)
Dementia with RX	308.0	253.2	1.2 (1.1–1.4)	54.8 (23.9–85.7)
Dementia without RX	396.1	342.7	1.2 (1.1–1.2)	53.4 (26.9–79.8)
No Dementia	84.3	78.0	1.1 (1.0–1.1)	6.3 (3.6–9.1)
P heterogeneity			0.06	<0.01

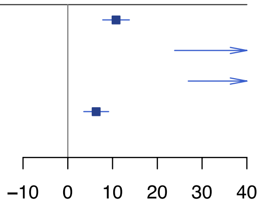

Abbreviations: CI, confidence interval; IR, incidence rate; PY, person-years; Rx, medications for dementia, including donepezil, rivastigmine, galantamine, and memantine.

© 2023 Lin KJ et al. *JAMA Network Open*.

eTable 1. Propensity Score Models for Each Oral Anticoagulant vs Apixaban in the Medicare Population

Characteristics	Odds Ratio (95% Confidence Interval) from Logistic Model		
	Warfarin vs Apixaban	Dabigatran vs Apixaban	Rivaroxaban vs. Apixaban
**Quarter/Year of Cohort Entry Date**			
Q2 2013	0.11 (0.10, 0.12)	0.11 (0.09, 0.12)	0.13 (0.11, 0.15)
Q3 2013	0.07 (0.06, 0.07)	0.06 (0.05, 0.07)	0.09 (0.08, 0.10)
Q4 2013	0.04 (0.04, 0.05)	0.03 (0.03, 0.04)	0.06 (0.05, 0.07)
Q1 2014	0.04 (0.03, 0.04)	0.02 (0.02, 0.03)	0.05 (0.04, 0.06)
Q2 2014	0.02 (0.02, 0.03)	0.02 (0.01, 0.02)	0.04 (0.03, 0.04)
Q3 2014	0.02 (0.02, 0.02)	0.01 (0.01, 0.01)	0.03 (0.03, 0.04)
Q4 2014	0.02 (0.02, 0.02)	0.01 (0.01, 0.01)	0.02 (0.02, 0.03)
Q1 2015	0.01 (0.01, 0.02)	0.01 (0.01, 0.01)	0.02 (0.02, 0.02)
Q2 2015	0.01 (0.01, 0.01)	0.01 (0.01, 0.01)	0.02 (0.02, 0.02)
Q3 2015	0.01 (0.01, 0.01)	0.00 (0.00, 0.01)	0.02 (0.01, 0.02)
Q4 2015	0.01 (0.01, 0.01)	0.01 (0.01, 0.01)	0.01 (0.01, 0.01)
Q1 2016	0.01 (0.01, 0.01)	0.01 (0.01, 0.01)	0.01 (0.01, 0.01)
Q2 2016	0.01 (0.01, 0.01)	0.01 (0.01, 0.01)	0.01 (0.01, 0.01)
Q3 2016	0.01 (0.01, 0.01)	0.01 (0.00, 0.01)	0.01 (0.01, 0.01)
Q4 2016	0.01 (0.01, 0.01)	0.01 (0.00, 0.01)	0.01 (0.01, 0.01)
Q1 2017	0.01 (0.01, 0.01)	0.01 (0.00, 0.01)	0.01 (0.01, 0.02)
Q2 2017	0.01 (0.00, 0.01)	0.00 (0.00, 0.01)	0.01 (0.01, 0.02)
Q3 2017	0.01 (0.00, 0.01)	0.00 (0.00, 0.01)	0.01 (0.01, 0.02)
Q4 2017	0.00 (0.00, 0.01)	0.00 (0.00, 0.00)	0.01 (0.01, 0.02)
**Patient Characteristics**			
Age	1.00 (0.99, 1.00)	0.97 (0.97, 0.98)	0.98 (0.98, 0.98)
Female	0.81 (0.80, 0.82)	0.85 (0.83, 0.87)	0.87 (0.86, 0.89)
Black (vs white)	1.03 (1.00, 1.06)	1.06 (1.01, 1.12)	1.11 (1.08, 1.14)
Other (vs white)	0.89 (0.86, 0.91)	1.17 (1.12, 1.23)	1.19 (1.15, 1.22)
Dual eligibility[a]	1.07 (1.04, 1.10)	1.03 (0.99, 1.08)	1.09 (1.07, 1.12)
**Cardiovascular Conditions**			
Acute myocardial infarction	0.93 (0.91, 0.96)	0.86 (0.82, 0.91)	0.94 (0.91, 0.96)
Cardio-ablation	0.66 (0.62, 0.72)	0.83 (0.73, 0.94)	0.90 (0.84, 0.96)
Cardioversion	0.46 (0.45, 0.48)	0.81 (0.78, 0.85)	0.87 (0.85, 0.90)
Cerebrovascular disease	0.93 (0.91, 0.95)	1.05 (1.01, 1.09)	0.92 (0.90, 0.94)
Congestive heart failure (inpatient)	0.98 (0.96, 1.00)	0.98 (0.94, 1.01)	0.97 (0.95, 0.99)
Congestive heart failure (outpatient)	1.03 (1.02, 1.05)	1.04 (1.01, 1.07)	0.97 (0.95, 0.99)
Coronary revascularization	2.74 (2.66, 2.83)	1.11 (1.04, 1.18)	1.02 (0.99, 1.06)
Hypertension	0.86 (0.83, 0.88)	0.93 (0.89, 0.98)	1.01 (0.98, 1.04)
Ischemic Heart Disease	0.94 (0.93, 0.96)	0.98 (0.96, 1.01)	0.91 (0.90, 0.93)
PVD or PVD Surgery	1.01 (0.99, 1.02)	1.04 (1.01, 1.07)	1.03 (1.01, 1.05)
Stroke (inpatient)	1.06 (1.04, 1.09)	1.06 (1.01, 1.11)	0.89 (0.87, 0.92)
Stroke (outpatient)	1.04 (1.02, 1.06)	0.97 (0.93, 1.01)	0.93 (0.90, 0.95)
Syncope	0.81 (0.79, 0.82)	0.92 (0.88, 0.95)	0.95 (0.93, 0.97)
CHA_2DS_2-VASc score (per point)	1.07 (1.06, 1.08)	1.00 (0.98, 1.02)	1.04 (1.03, 1.05)
HAS-BLED score (per point)	0.97 (0.95, 0.99)	1.03 (0.99, 1.06)	0.93 (0.91, 0.94)
**Non-cardiovascular Conditions**			
Acute renal failure	1.05 (1.03, 1.07)	0.86 (0.82, 0.89)	0.90 (0.88, 0.92)
Alcohol abuse or dependence	0.98 (0.93, 1.02)	1.11 (1.02, 1.20)	1.12 (1.07, 1.17)
Anemia	1.09 (1.07, 1.12)	0.98 (0.94, 1.02)	1.07 (1.04, 1.10)
CKD: stage 3, 4 or unspecified	1.08 (1.06, 1.10)	0.83 (0.80, 0.87)	0.86 (0.84, 0.87)
COPD	1.07 (1.05, 1.09)	1.06 (1.03, 1.09)	1.02 (1.01, 1.04)
Dementia	0.90 (0.88, 0.93)	0.96 (0.91, 1.01)	1.07 (1.04, 1.10)
Diabetes	1.00 (0.98, 1.02)	1.06 (1.03, 1.10)	0.98 (0.96, 1.00)
Endoscopy	1.06 (1.02, 1.10)	0.94 (0.87, 1.02)	0.95 (0.90, 0.99)
Falls	1.02 (0.99, 1.05)	0.98 (0.93, 1.03)	0.98 (0.95, 1.01)
Fractures	1.09 (1.07, 1.12)	1.05 (1.01, 1.09)	1.08 (1.05, 1.10)
GI bleeding events (inpatient)	1.15 (1.12, 1.18)	1.07 (1.02, 1.12)	1.00 (0.98, 1.03)
GI bleeding events (outpatient)	1.11 (1.09, 1.13)	1.01 (0.97, 1.04)	0.98 (0.96, 1.00)
Liver disease	0.99 (0.97, 1.01)	1.04 (0.99, 1.08)	1.01 (0.99, 1.04)
Malignancy	0.91 (0.90, 0.93)	0.98 (0.96, 1.01)	1.00 (0.98, 1.01)

© 2023 Lin KJ et al. *JAMA Network Open*.

Characteristics	Odds Ratio (95% Confidence Interval) from Logistic Model		
	Warfarin vs Apixaban	Dabigatran vs Apixaban	Rivaroxaban vs. Apixaban
Obesity	0.99 (0.98, 1.01)	1.05 (1.03, 1.08)	1.02 (1.01, 1.04)
Peptic ulcer disease	0.99 (0.95, 1.03)	0.89 (0.83, 0.96)	0.96 (0.92, 1.00)
Smoking	0.95 (0.94, 0.97)	0.96 (0.94, 0.99)	0.99 (0.97, 1.00)
Combined comorbidity index (per point)	1.02 (1.02, 1.03)	0.98 (0.98, 0.99)	0.99 (0.98, 0.99)
Claims-based frailty index (per 0.1 point)	4.28 (3.71, 4.95)	1.97 (1.51, 2.57)	1.72 (1.48, 2.00)
**Cardiovascular Medications**			
ACE inhibitors	1.08 (1.07, 1.10)	0.98 (0.96, 1.01)	1.01 (0.99, 1.02)
Angiotensin receptor blockers	0.81 (0.79, 0.83)	1.01 (0.97, 1.05)	0.98 (0.95, 1.00)
Antiarrhythmic agents	0.81 (0.80, 0.82)	1.02 (0.99, 1.04)	0.90 (0.89, 0.92)
Anticoagulants, injectable	12.09 (11.50, 12.71)	1.39 (1.24, 1.56)	1.35 (1.26, 1.44)
Antiplatelet agent	0.64 (0.63, 0.65)	0.79 (0.77, 0.82)	0.94 (0.92, 0.95)
Beta-blockers	0.99 (0.97, 1.00)	0.91 (0.89, 0.93)	0.94 (0.93, 0.95)
Calcium channel blockers	0.89 (0.85, 0.92)	0.93 (0.87, 0.99)	0.98 (0.94, 1.02)
Diuretics	1.18 (1.17, 1.20)	1.04 (1.02, 1.06)	1.00 (0.98, 1.01)
Fibrates	1.08 (1.05, 1.11)	1.01 (0.96, 1.06)	1.01 (0.98, 1.04)
Nitrates	1.04 (1.03, 1.06)	1.02 (0.99, 1.06)	0.96 (0.94, 0.98)
Statins	0.99 (0.97, 1.00)	0.97 (0.95, 0.99)	0.96 (0.95, 0.97)
**Non-cardiovascular Medications**			
Anticonvulsants	1.03 (1.02, 1.05)	1.02 (0.99, 1.05)	1.00 (0.98, 1.02)
Antidepressants - SSRIs/SNRIs	0.99 (0.97, 1.01)	1.00 (0.97, 1.04)	1.00 (0.97, 1.02)
Antidepressants - Tricyclics	1.00 (0.99, 1.02)	1.04 (1.02, 1.07)	1.01 (0.99, 1.02)
Antidepressants - Other	1.01 (0.98, 1.05)	0.93 (0.87, 0.99)	0.97 (0.94, 1.01)
Antipsychotic agents	0.97 (0.94, 1.01)	1.04 (0.98, 1.11)	1.12 (1.08, 1.16)
Anxiolytics - Benzodiazepines	0.91 (0.90, 0.93)	0.97 (0.94, 1.00)	1.00 (0.98, 1.01)
Anxiolytics - Other	1.05 (0.99, 1.11)	1.02 (0.92, 1.12)	1.03 (0.97, 1.09)
Bronchodilators	0.94 (0.93, 0.96)	0.99 (0.96, 1.02)	1.02 (1.00, 1.04)
Corticosteroids, inhaled	0.86 (0.85, 0.88)	0.95 (0.92, 0.97)	0.97 (0.95, 0.98)
Corticosteroids, oral	0.95 (0.94, 0.96)	1.00 (0.98, 1.02)	0.99 (0.98, 1.00)
Dementia drugs	0.93 (0.90, 0.96)	1.09 (1.04, 1.15)	1.02 (0.99, 1.05)
Diabetes agents - Insulin	0.97 (0.95, 0.99)	0.98 (0.94, 1.02)	0.94 (0.92, 0.96)
Diabetes agents - Metformin	0.95 (0.93, 0.97)	1.02 (0.98, 1.05)	1.02 (1.00, 1.04)
Diabetes agents - Sulfonylurea	0.78 (0.76, 0.81)	0.98 (0.94, 1.03)	0.98 (0.96, 1.01)
Diabetes agents - Other	1.11 (1.09, 1.13)	1.04 (1.00, 1.08)	0.98 (0.96, 1.01)
Estrogen	0.78 (0.75, 0.80)	0.93 (0.88, 0.99)	0.94 (0.91, 0.97)
GI - H2 blockers	0.99 (0.97, 1.01)	1.00 (0.96, 1.04)	0.99 (0.97, 1.02)
GI - Proton-pump inhibitors	0.95 (0.94, 0.96)	0.98 (0.95, 1.00)	0.98 (0.97, 0.99)
GI - Sucralfate	0.94 (0.90, 0.98)	1.04 (0.97, 1.12)	1.02 (0.98, 1.06)
Hypnotics	0.87 (0.85, 0.89)	0.95 (0.92, 0.99)	0.99 (0.97, 1.01)
NSAIDs	0.76 (0.75, 0.78)	0.94 (0.91, 0.96)	1.03 (1.02, 1.05)
Opioids	1.10 (1.08, 1.11)	1.05 (1.03, 1.07)	1.02 (1.01, 1.04)
Parkinsonism drugs	0.95 (0.93, 0.98)	1.03 (0.98, 1.09)	0.98 (0.95, 1.01)
Thyroid hormone replacement	1.01 (1.00, 1.03)	1.00 (0.98, 1.03)	0.98 (0.97, 1.00)
**Health care utilization in past 183 days**			
Emergency department visits	0.90 (0.89, 0.91)	0.93 (0.91, 0.95)	1.01 (1.00, 1.03)
Home health day[b]	1.22 (1.18, 1.26)	1.28 (1.22, 1.35)	1.16 (1.12, 1.20)
Home oxygen use	0.97 (0.94, 1.00)	0.94 (0.88, 1.00)	0.97 (0.94, 1.00)
Hospitalizations	0.74 (0.73, 0.75)	0.79 (0.77, 0.81)	0.96 (0.94, 0.97)
**Geographic region**			
Midwest	1.20 (1.18, 1.22)	0.89 (0.86, 0.91)	1.04 (1.02, 1.05)
South	0.65 (0.64, 0.66)	0.75 (0.73, 0.77)	0.86 (0.85, 0.87)
West	0.97 (0.95, 0.99)	0.85 (0.82, 0.88)	1.10 (1.08, 1.12)
Other	1.71 (1.41, 2.06)	1.32 (0.96, 1.81)	1.52 (1.26, 1.84)

Abbreviations: ACE, angiotensin converting enzyme; CCI, combined comorbidity; CFI, claims-based frailty index; CKD, chronic kidney disease; COPD, chronic obstructive pulmonary disease; GI, gastrointestinal; NSAID, non-steroidal anti-inflammatory drug; PVD, peripheral vascular disease; SNRI, serotonin-norepinephrine reuptake inhibitor; SSRI, selective serotonin reuptake inhibitor.

[a] With both Medicare and Medicaid enrollment eligibility

[b] Home health services received (days)

© 2023 Lin KJ et al. *JAMA Network Open*.

eTable 2. Propensity Score Models for Each Oral Anticoagulant vs Apixaban in the Optum Population

Characteristics	Odds Ratio (95% Confidence Interval) from Logistic Model		
	Warfarin vs Apixaban	Dabigatran vs Apixaban	Rivaroxaban vs. Apixaban
Quarter/Year of Cohort Entry Date			
Q2 2013	0.16 (0.11, 0.23)	0.14 (0.10, 0.21)	0.20 (0.14, 0.28)
Q3 2013	0.12 (0.08, 0.16)	0.10 (0.07, 0.14)	0.15 (0.11, 0.22)
Q4 2013	0.06 (0.04, 0.08)	0.05 (0.03, 0.07)	0.09 (0.07, 0.13)
Q1 2014	0.03 (0.02, 0.05)	0.03 (0.02, 0.04)	0.07 (0.05, 0.09)
Q2 2014	0.03 (0.02, 0.04)	0.02 (0.01, 0.03)	0.05 (0.04, 0.07)
Q3 2014	0.02 (0.02, 0.03)	0.01 (0.01, 0.02)	0.04 (0.03, 0.06)
Q4 2014	0.02 (0.01, 0.03)	0.01 (0.01, 0.02)	0.03 (0.02, 0.05)
Q1 2015	0.02 (0.01, 0.02)	0.01 (0.01, 0.01)	0.03 (0.02, 0.04)
Q2 2015	0.01 (0.01, 0.02)	0.01 (0.00, 0.01)	0.02 (0.02, 0.03)
Q3 2015	0.01 (0.01, 0.02)	0.01 (0.00, 0.01)	0.02 (0.02, 0.03)
Q4 2015	0.01 (0.01, 0.02)	0.01 (0.00, 0.01)	0.02 (0.01, 0.03)
Q1 2016	0.01 (0.01, 0.01)	0.01 (0.01, 0.01)	0.02 (0.01, 0.03)
Q2 2016	0.01 (0.01, 0.01)	0.01 (0.01, 0.01)	0.02 (0.01, 0.02)
Q3 2016	0.01 (0.00, 0.01)	0.01 (0.00, 0.01)	0.02 (0.01, 0.02)
Q4 2016	0.01 (0.00, 0.01)	0.01 (0.00, 0.01)	0.01 (0.01, 0.02)
Q1 2017	0.01 (0.00, 0.01)	0.00 (0.00, 0.01)	0.02 (0.01, 0.02)
Q2 2017	0.00 (0.00, 0.01)	0.00 (0.00, 0.00)	0.02 (0.01, 0.02)
Q3 2017	0.00 (0.00, 0.01)	0.00 (0.00, 0.00)	0.02 (0.01, 0.02)
Q4 2017	0.00 (0.00, 0.01)	0.00 (0.00, 0.00)	0.02 (0.01, 0.02)
Q1 2018	0.00 (0.00, 0.00)	0.00 (0.00, 0.00)	0.01 (0.01, 0.02)
Q2 2018	0.00 (0.00, 0.00)	0.00 (0.00, 0.00)	0.01 (0.01, 0.02)
Q3 2018	0.00 (0.00, 0.00)	0.00 (0.00, 0.00)	0.01 (0.01, 0.02)
Q4 2018	0.00 (0.00, 0.00)	0.00 (0.00, 0.00)	0.01 (0.01, 0.01)
Q1 2019	0.00 (0.00, 0.00)	0.00 (0.00, 0.00)	0.01 (0.01, 0.01)
Q2 2019	0.00 (0.00, 0.00)	insufficient numbers	0.01 (0.01, 0.01)
Q3 2019	0.00 (0.00, 0.00)	0.00 (0.00, 0.00)	0.01 (0.01, 0.01)
Q4 2019	0.00 (0.00, 0.00)	insufficient numbers	0.01 (0.01, 0.01)
Q1 2020	0.00 (0.00, 0.00)	insufficient numbers	0.01 (0.01, 0.01)
Q2 2020	insufficient numbers	insufficient numbers	0.01 (0.01, 0.01)
Q3 2020	insufficient numbers	insufficient numbers	0.01 (0.00, 0.01)
Q4 2020	insufficient numbers	insufficient numbers	0.01 (0.00, 0.01)
Q1 2021	0.00 (0.00, 0.00)	insufficient numbers	0.01 (0.01, 0.01)
Q2 2021	insufficient numbers	insufficient numbers	0.01 (0.00, 0.01)
**Patient Characteristics**			
Age	1.00 (1.00, 1.01)	0.98 (0.97, 0.99)	0.98 (0.98, 0.98)
Female	0.80 (0.77, 0.83)	0.80 (0.74, 0.86)	0.88 (0.85, 0.91)
Black (vs white)	0.98 (0.94, 1.03)	0.98 (0.88, 1.08)	1.01 (0.97, 1.05)
Other (vs white)	0.94 (0.90, 0.97)	1.19 (1.11, 1.28)	1.11 (1.08, 1.14)
**Cardiovascular Conditions**			
Acute myocardial infarction	0.90 (0.85, 0.95)	0.81 (0.71, 0.93)	0.95 (0.91, 1.00)
Cardio-ablation	0.65 (0.54, 0.78)	1.01 (0.74, 1.40)	0.88 (0.76, 1.03)
Cardioversion	0.47 (0.44, 0.49)	0.72 (0.65, 0.80)	0.90 (0.86, 0.94)
Cerebrovascular disease	1.10 (1.00, 1.21)	1.00 (0.82, 1.23)	0.92 (0.84, 1.01)
Congestive heart failure (inpatient)	1.00 (0.95, 1.04)	0.97 (0.88, 1.07)	0.96 (0.92, 1.00)
Congestive heart failure (outpatient)	1.18 (1.13, 1.22)	1.18 (1.09, 1.28)	1.07 (1.03, 1.11)
Coronary revascularization	2.58 (2.41, 2.77)	1.08 (0.90, 1.31)	0.90 (0.84, 0.97)
Hypertension	0.90 (0.85, 0.96)	1.15 (1.00, 1.31)	1.07 (1.02, 1.13)
Ischemic Heart Disease	1.00 (0.97, 1.03)	1.02 (0.95, 1.09)	0.95 (0.93, 0.98)
PVD or PVD Surgery	1.01 (0.98, 1.05)	1.02 (0.94, 1.10)	1.12 (1.08, 1.16)
Stroke (inpatient)	0.96 (0.90, 1.02)	1.05 (0.91, 1.20)	0.81 (0.77, 0.86)
Stroke (outpatient)	0.88 (0.80, 0.97)	0.98 (0.80, 1.20)	1.05 (0.96, 1.16)
Syncope	0.80 (0.77, 0.84)	0.91 (0.84, 0.99)	0.95 (0.92, 0.99)
CHA_2DS_2-VASc score (per point)	1.02 (0.99, 1.04)	1.00 (0.96, 1.05)	0.99 (0.97, 1.01)
HAS-BLED score (per point)	1.00 (0.96, 1.04)	0.97 (0.88, 1.06)	0.91 (0.88, 0.95)
**Non-cardiovascular Conditions**			
Acute renal failure	1.05 (1.01, 1.09)	0.81 (0.73, 0.89)	0.84 (0.81, 0.87)
Alcohol abuse or dependence	0.90 (0.83, 0.98)	1.08 (0.91, 1.30)	1.11 (1.03, 1.19)

© 2023 Lin KJ et al. *JAMA Network Open*.

Characteristics	Odds Ratio (95% Confidence Interval) from Logistic Model		
	Warfarin vs Apixaban	Dabigatran vs Apixaban	Rivaroxaban vs. Apixaban
Anemia	1.05 (1.00, 1.10)	1.06 (0.95, 1.19)	1.12 (1.07, 1.18)
CKD: stage 3, 4 or unspecified	1.07 (1.04, 1.11)	0.96 (0.88, 1.04)	0.98 (0.95, 1.01)
COPD	1.18 (1.14, 1.22)	1.02 (0.95, 1.10)	1.07 (1.03, 1.10)
Dementia	0.85 (0.80, 0.91)	0.94 (0.82, 1.09)	1.11 (1.05, 1.18)
Diabetes	1.15 (1.10, 1.20)	1.06 (0.97, 1.15)	1.04 (1.00, 1.08)
Endoscopy	1.19 (1.07, 1.31)	0.91 (0.71, 1.16)	0.96 (0.86, 1.07)
Falls	1.07 (1.03, 1.12)	1.09 (0.99, 1.20)	1.02 (0.98, 1.06)
Fractures	1.11 (1.05, 1.16)	1.09 (0.98, 1.20)	1.12 (1.07, 1.17)
GI bleeding events (inpatient)	1.25 (1.16, 1.36)	1.11 (0.92, 1.33)	1.04 (0.96, 1.13)
GI bleeding events (outpatient)	1.01 (0.98, 1.04)	1.12 (1.05, 1.19)	1.03 (1.00, 1.06)
Liver disease	0.91 (0.87, 0.96)	1.04 (0.93, 1.15)	0.97 (0.93, 1.02)
Malignancy	0.91 (0.87, 0.94)	1.00 (0.93, 1.08)	0.99 (0.95, 1.02)
Obesity	1.02 (0.99, 1.06)	1.01 (0.94, 1.08)	1.03 (1.00, 1.06)
Peptic ulcer disease	1.01 (0.93, 1.10)	1.05 (0.87, 1.26)	0.89 (0.83, 0.96)
Smoking	0.95 (0.92, 0.98)	0.92 (0.86, 0.99)	0.95 (0.93, 0.98)
Combined comorbidity index (per point)	1.01 (1.00, 1.01)	0.97 (0.95, 0.99)	0.98 (0.97, 0.99)
Claims-based frailty index (per 0.1 point)	2.27 (1.63, 3.17)	8.64 (4.23, 17.64)	1.56 (1.14, 2.12)
**Cardiovascular Medications**			
ACE inhibitors	1.07 (1.04, 1.10)	0.96 (0.90, 1.02)	0.98 (0.96, 1.01)
Angiotensin receptor blockers	0.78 (0.74, 0.83)	0.92 (0.82, 1.03)	1.02 (0.97, 1.07)
Antiarrhythmic agents	0.81 (0.78, 0.83)	1.06 (1.00, 1.14)	0.94 (0.91, 0.97)
Anticoagulants, injectable	10.17 (9.21, 11.23)	0.88 (0.60, 1.29)	1.14 (0.98, 1.33)
Antiplatelet agent	0.66 (0.63, 0.69)	0.74 (0.68, 0.81)	1.00 (0.97, 1.04)
Beta-blockers	0.96 (0.93, 0.98)	0.87 (0.83, 0.92)	0.94 (0.92, 0.96)
Calcium channel blockers	0.84 (0.49, 1.43)	1.53 (0.71, 3.31)	1.35 (0.86, 2.10)
Diuretics	1.11 (1.08, 1.14)	0.95 (0.90, 1.01)	0.99 (0.97, 1.01)
Fibrates	1.01 (0.95, 1.08)	1.06 (0.94, 1.20)	1.00 (0.94, 1.05)
Nitrates	1.05 (1.00, 1.09)	1.00 (0.92, 1.10)	0.94 (0.90, 0.97)
Statins	0.96 (0.94, 0.99)	0.93 (0.88, 0.98)	0.96 (0.93, 0.98)
**Non-cardiovascular Medications**			
Anticonvulsants	1.09 (1.05, 1.12)	1.04 (0.96, 1.12)	0.99 (0.96, 1.02)
Antidepressants - SSRIs/SNRIs	1.04 (1.00, 1.09)	0.98 (0.89, 1.08)	1.02 (0.98, 1.06)
Antidepressants - Tricyclics	1.02 (0.99, 1.06)	1.04 (0.97, 1.12)	0.97 (0.95, 1.00)
Antidepressants - Other	1.03 (0.95, 1.12)	0.87 (0.73, 1.05)	0.93 (0.87, 1.01)
Antipsychotic agents	0.91 (0.84, 1.00)	1.05 (0.88, 1.26)	1.02 (0.95, 1.10)
Anxiolytics - Benzodiazepines	0.88 (0.85, 0.92)	1.00 (0.93, 1.08)	0.99 (0.96, 1.02)
Anxiolytics - Other	1.02 (0.90, 1.16)	0.91 (0.70, 1.20)	1.00 (0.90, 1.11)
Bronchodilators	0.90 (0.87, 0.93)	1.03 (0.95, 1.12)	1.00 (0.97, 1.04)
Corticosteroids, inhaled	0.80 (0.78, 0.83)	0.88 (0.82, 0.95)	0.97 (0.94, 1.00)
Corticosteroids, oral	0.89 (0.87, 0.92)	1.01 (0.95, 1.07)	0.97 (0.95, 0.99)
Dementia drugs	0.99 (0.92, 1.06)	1.17 (1.01, 1.36)	1.05 (0.98, 1.12)
Diabetes agents - Insulin	0.97 (0.92, 1.01)	1.10 (0.99, 1.23)	0.94 (0.90, 0.99)
Diabetes agents - Metformin	0.95 (0.91, 0.99)	1.07 (0.98, 1.17)	1.03 (0.99, 1.07)
Diabetes agents - Other	0.79 (0.74, 0.84)	0.98 (0.86, 1.11)	1.07 (1.01, 1.13)
Diabetes agents - Sulfonylurea	1.13 (1.08, 1.18)	0.96 (0.87, 1.06)	0.97 (0.93, 1.02)
Estrogen	0.71 (0.65, 0.78)	0.94 (0.79, 1.11)	0.91 (0.84, 0.98)
GI - H2 blockers	0.99 (0.94, 1.04)	1.01 (0.91, 1.12)	1.01 (0.97, 1.05)
GI - Proton-pump inhibitors	0.94 (0.91, 0.96)	0.93 (0.87, 0.99)	0.98 (0.95, 1.00)
GI - Sucralfate	0.93 (0.84, 1.03)	0.89 (0.72, 1.11)	0.98 (0.90, 1.07)
Hypnotics	0.90 (0.85, 0.96)	1.06 (0.95, 1.19)	1.02 (0.97, 1.07)
NSAIDs	0.75 (0.72, 0.77)	0.85 (0.79, 0.91)	1.01 (0.98, 1.04)
Opioids	1.03 (1.01, 1.06)	0.97 (0.92, 1.03)	1.00 (0.97, 1.02)
Parkinsonism drugs	0.96 (0.89, 1.02)	0.99 (0.86, 1.15)	0.95 (0.90, 1.01)
Thyroid hormone replacement	1.00 (0.97, 1.03)	0.98 (0.92, 1.05)	0.99 (0.96, 1.02)
**Health care utilization in past 183 days**			
Emergency department visits	0.78 (0.76, 0.80)	0.85 (0.80, 0.90)	0.91 (0.88, 0.93)
Home oxygen use	0.97 (0.91, 1.04)	0.84 (0.72, 0.99)	0.91 (0.86, 0.97)
Hospitalizations	0.76 (0.73, 0.79)	0.74 (0.69, 0.80)	0.82 (0.79, 0.84)
**Geographic Region**			
Midwest	1.07 (1.03, 1.12)	0.71 (0.65, 0.78)	0.91 (0.87, 0.94)

Characteristics	Odds Ratio (95% Confidence Interval) from Logistic Model		
	Warfarin vs Apixaban	Dabigatran vs Apixaban	Rivaroxaban vs. Apixaban
South	0.61 (0.59, 0.64)	0.72 (0.67, 0.78)	0.83 (0.80, 0.86)
West	0.94 (0.90, 0.98)	0.71 (0.66, 0.78)	0.97 (0.94, 1.01)
Other	1.02 (0.65, 1.60)	0.99 (0.43, 2.25)	1.06 (0.70, 1.60)
**Business Type Code**			
Medicare	1.66 (1.58, 1.75)	0.89 (0.82, 0.97)	0.99 (0.95, 1.03)

Abbreviations: ACE, angiotensin converting enzyme; CCI, combined comorbidity; CFI, claims-based frailty index; CKD, chronic kidney disease; COPD, chronic obstructive pulmonary disease; GI, gastrointestinal; NSAID, non-steroidal anti-inflammatory drug; PVD, peripheral vascular disease; SNRI, serotonin-norepinephrine reuptake inhibitor; SSRI, selective serotonin reuptake inhibitor.

© 2023 Lin KJ et al. *JAMA Network Open*.

eTable 3. Propensity Score Models for Each Oral Anticoagulant vs Apixaban in the MarketScan Population

Characteristics	Odds Ratio (95% Confidence Interval) from Logistic Model		
	Warfarin vs Apixaban	Dabigatran vs Apixaban	Rivaroxaban vs. Apixaban
Quarter/Year of Cohort Entry Date			
Q2 2013	0.15 (0.12, 0.18)	0.15 (0.12, 0.19)	0.20 (0.16, 0.25)
Q3 2013	0.09 (0.07, 0.11)	0.09 (0.07, 0.12)	0.14 (0.11, 0.17)
Q4 2013	0.06 (0.05, 0.07)	0.05 (0.04, 0.07)	0.10 (0.08, 0.12)
Q1 2014	0.05 (0.04, 0.06)	0.04 (0.03, 0.05)	0.10 (0.08, 0.12)
Q2 2014	0.03 (0.03, 0.04)	0.03 (0.02, 0.03)	0.08 (0.06, 0.10)
Q3 2014	0.03 (0.03, 0.04)	0.02 (0.02, 0.03)	0.07 (0.06, 0.09)
Q4 2014	0.02 (0.02, 0.03)	0.01 (0.01, 0.02)	0.05 (0.04, 0.07)
Q1 2015	0.02 (0.01, 0.02)	0.01 (0.01, 0.02)	0.04 (0.03, 0.05)
Q2 2015	0.02 (0.01, 0.02)	0.01 (0.01, 0.01)	0.04 (0.03, 0.05)
Q3 2015	0.01 (0.01, 0.02)	0.01 (0.01, 0.01)	0.03 (0.03, 0.04)
Q4 2015	0.01 (0.01, 0.01)	0.01 (0.01, 0.02)	0.03 (0.02, 0.04)
Q1 2016	0.01 (0.01, 0.01)	0.01 (0.01, 0.01)	0.02 (0.02, 0.03)
Q2 2016	0.01 (0.01, 0.01)	0.01 (0.01, 0.01)	0.02 (0.02, 0.03)
Q3 2016	0.01 (0.01, 0.01)	0.01 (0.01, 0.01)	0.02 (0.02, 0.02)
Q4 2016	0.01 (0.01, 0.01)	0.01 (0.01, 0.01)	0.02 (0.01, 0.02)
Q1 2017	0.01 (0.00, 0.01)	0.01 (0.00, 0.01)	0.02 (0.02, 0.02)
Q2 2017	0.01 (0.00, 0.01)	0.01 (0.00, 0.01)	0.02 (0.02, 0.02)
Q3 2017	0.00 (0.00, 0.01)	0.00 (0.00, 0.01)	0.02 (0.02, 0.02)
Q4 2017	0.00 (0.00, 0.00)	0.00 (0.00, 0.01)	0.02 (0.01, 0.02)
Q1 2018	0.00 (0.00, 0.00)	0.00 (0.00, 0.01)	0.02 (0.01, 0.02)
Q2 2018	0.00 (0.00, 0.00)	0.00 (0.00, 0.00)	0.02 (0.01, 0.02)
Q3 2018	0.00 (0.00, 0.00)	0.00 (0.00, 0.00)	0.01 (0.01, 0.02)
Q4 2018	0.00 (0.00, 0.00)	0.00 (0.00, 0.00)	0.01 (0.01, 0.02)
Q1 2019	0.00 (0.00, 0.00)	0.00 (0.00, 0.00)	0.01 (0.01, 0.01)
Q2 2019	0.00 (0.00, 0.00)	0.00 (0.00, 0.00)	0.01 (0.01, 0.01)
Q3 2019	0.00 (0.00, 0.00)	0.00 (0.00, 0.00)	0.01 (0.01, 0.01)
Q4 2019	0.00 (0.00, 0.00)	0.00 (0.00, 0.00)	0.01 (0.01, 0.01)
Q1 2020	0.00 (0.00, 0.00)	insufficient numbers	0.01 (0.01, 0.01)
Q2 2020	0.00 (0.00, 0.00)	insufficient numbers	0.01 (0.01, 0.01)
Q3 2020	0.00 (0.00, 0.00)	insufficient numbers	0.01 (0.01, 0.01)
Q4 2020	0.00 (0.00, 0.00)	insufficient numbers	0.01 (0.01, 0.01)
Q2 2013	0.15 (0.12, 0.18)	0.15 (0.12, 0.19)	0.20 (0.16, 0.25)
Q3 2013	0.09 (0.07, 0.11)	0.09 (0.07, 0.12)	0.14 (0.11, 0.17)
**Patient Characteristics**			
Age	1.02 (1.02, 1.02)	0.98 (0.98, 0.99)	0.98 (0.98, 0.99)
Female	0.88 (0.84, 0.92)	0.95 (0.88, 1.02)	0.98 (0.94, 1.02)
**Cardiovascular Conditions**			
Acute myocardial infarction	0.92 (0.86, 0.99)	0.89 (0.77, 1.02)	0.96 (0.90, 1.03)
Cardio-ablation	0.66 (0.51, 0.86)	1.43 (1.03, 1.98)	1.00 (0.82, 1.23)
Cardioversion	0.45 (0.41, 0.48)	0.79 (0.71, 0.88)	0.87 (0.82, 0.93)
Cerebrovascular disease	1.07 (0.98, 1.17)	1.20 (1.03, 1.40)	1.08 (0.99, 1.18)
Congestive heart failure (inpatient)	1.07 (1.01, 1.13)	1.01 (0.92, 1.12)	1.09 (1.04, 1.15)
Congestive heart failure (outpatient)	1.14 (1.09, 1.19)	1.16 (1.08, 1.26)	1.02 (0.98, 1.06)
Coronary revascularization	3.28 (2.98, 3.61)	0.91 (0.74, 1.12)	1.04 (0.94, 1.15)
Hypertension	0.88 (0.81, 0.95)	1.17 (1.02, 1.35)	1.22 (1.13, 1.32)
Ischemic Heart Disease	1.03 (0.99, 1.07)	1.03 (0.96, 1.10)	0.97 (0.93, 1.01)
PVD or PVD Surgery	1.12 (1.07, 1.18)	1.17 (1.07, 1.28)	1.09 (1.03, 1.14)
Stroke (inpatient)	1.12 (1.03, 1.21)	1.01 (0.88, 1.17)	1.06 (0.98, 1.15)
Stroke (outpatient)	0.95 (0.87, 1.04)	0.88 (0.75, 1.02)	0.93 (0.85, 1.01)
Syncope	0.77 (0.73, 0.82)	0.88 (0.80, 0.96)	0.95 (0.91, 1.00)
CHA_2DS_2-VASc score (per point)	0.96 (0.93, 0.98)	0.91 (0.88, 0.95)	0.92 (0.90, 0.94)
HAS-BLED score (per point)	1.01 (0.95, 1.08)	0.99 (0.88, 1.10)	0.85 (0.80, 0.91)
**Non-cardiovascular Conditions**			
Acute renal failure	1.13 (1.07, 1.20)	0.85 (0.76, 0.95)	0.91 (0.86, 0.97)
Alcohol abuse or dependence	1.02 (0.88, 1.17)	1.11 (0.87, 1.40)	1.31 (1.15, 1.48)
Anemia	1.09 (1.01, 1.17)	1.06 (0.93, 1.22)	1.16 (1.07, 1.24)

© 2023 Lin KJ et al. *JAMA Network Open*.

Characteristics	Odds Ratio (95% Confidence Interval) from Logistic Model		
	Warfarin vs Apixaban	Dabigatran vs Apixaban	Rivaroxaban vs. Apixaban
CKD: stage 3, 4 or unspecified	1.05 (0.99, 1.10)	0.89 (0.80, 0.98)	0.88 (0.83, 0.92)
COPD	1.02 (0.97, 1.07)	1.05 (0.96, 1.15)	1.04 (0.99, 1.09)
Dementia	0.91 (0.84, 1.00)	0.91 (0.77, 1.07)	1.05 (0.96, 1.15)
Diabetes	1.23 (1.17, 1.29)	1.14 (1.04, 1.24)	1.10 (1.04, 1.15)
Endoscopy	1.01 (0.87, 1.18)	1.06 (0.79, 1.42)	0.91 (0.78, 1.07)
Falls	1.13 (1.06, 1.21)	1.05 (0.92, 1.19)	1.05 (0.98, 1.12)
Fractures	1.09 (1.02, 1.16)	0.91 (0.81, 1.02)	1.07 (1.01, 1.14)
GI bleeding events (inpatient)	1.44 (1.31, 1.59)	1.11 (0.92, 1.33)	1.14 (1.03, 1.25)
GI bleeding events (outpatient)	0.96 (0.91, 1.01)	0.95 (0.88, 1.04)	0.97 (0.93, 1.02)
Liver disease	1.04 (0.97, 1.12)	1.05 (0.93, 1.19)	0.99 (0.93, 1.06)
Malignancy	0.94 (0.90, 0.98)	1.01 (0.93, 1.08)	1.02 (0.98, 1.07)
Obesity	0.94 (0.89, 0.99)	1.00 (0.92, 1.09)	1.00 (0.95, 1.04)
Peptic ulcer disease	0.97 (0.83, 1.13)	0.89 (0.67, 1.19)	1.02 (0.89, 1.16)
Smoking	0.92 (0.87, 0.97)	1.01 (0.92, 1.10)	1.00 (0.96, 1.05)
Combined comorbidity index (per point)	1.03 (1.02, 1.05)	0.98 (0.96, 1.00)	0.98 (0.97, 0.99)
Claims-based frailty index (per 0.1 point)	3.54 (2.22, 5.65)	4.02 (1.78, 9.08)	1.47 (0.94, 2.30)
**Cardiovascular Medications**			
ACE inhibitors	1.03 (0.99, 1.07)	0.95 (0.89, 1.01)	0.97 (0.94, 1.01)
Angiotensin receptor blockers	0.88 (0.82, 0.95)	1.01 (0.91, 1.13)	1.00 (0.94, 1.07)
Antiarrhythmic agents	0.94 (0.90, 0.99)	1.04 (0.97, 1.12)	0.92 (0.89, 0.96)
Anticoagulants, injectable	15.94 (13.74, 18.49)	1.51 (1.08, 2.11)	1.37 (1.12, 1.67)
Antiplatelet agent	0.61 (0.58, 0.64)	0.78 (0.72, 0.85)	0.93 (0.89, 0.97)
Beta-blockers	1.01 (0.97, 1.05)	0.94 (0.88, 0.99)	0.95 (0.92, 0.98)
Calcium channel blockers	0.97 (0.68, 1.36)	0.68 (0.35, 1.29)	0.91 (0.65, 1.27)
Diuretics	1.22 (1.17, 1.26)	0.99 (0.93, 1.05)	0.99 (0.96, 1.02)
Fibrates	1.08 (1.00, 1.17)	0.90 (0.79, 1.03)	1.00 (0.93, 1.08)
Nitrates	1.00 (0.95, 1.05)	0.92 (0.83, 1.01)	0.93 (0.88, 0.98)
Statins	1.02 (0.98, 1.06)	0.96 (0.91, 1.02)	0.95 (0.92, 0.98)
**Non-cardiovascular Medications**			
Anticonvulsants	1.05 (1.00, 1.11)	1.03 (0.95, 1.12)	1.00 (0.95, 1.04)
Antidepressants - SSRIs/SNRIs	1.02 (0.97, 1.07)	1.03 (0.96, 1.12)	0.97 (0.93, 1.01)
Antidepressants - Tricyclics	1.04 (0.93, 1.16)	0.89 (0.74, 1.07)	1.04 (0.94, 1.14)
Antidepressants - Other	1.03 (0.96, 1.10)	1.01 (0.90, 1.14)	1.05 (0.99, 1.12)
Antipsychotic agents	0.93 (0.83, 1.05)	1.16 (0.96, 1.41)	1.07 (0.96, 1.19)
Anxiolytics - Benzodiazepines	0.91 (0.86, 0.95)	0.95 (0.88, 1.03)	1.00 (0.96, 1.05)
Anxiolytics - Other	0.96 (0.80, 1.16)	1.03 (0.77, 1.38)	1.03 (0.88, 1.21)
Bronchodilators	0.99 (0.94, 1.05)	0.94 (0.86, 1.02)	0.99 (0.95, 1.04)
Corticosteroids, inhaled	0.90 (0.85, 0.94)	0.93 (0.87, 1.01)	0.96 (0.92, 1.00)
Corticosteroids, oral	0.98 (0.94, 1.01)	1.01 (0.95, 1.08)	1.02 (0.98, 1.05)
Dementia drugs	1.08 (0.99, 1.19)	1.28 (1.09, 1.51)	1.04 (0.95, 1.14)
Diabetes agents - Insulin	1.00 (0.93, 1.07)	1.06 (0.94, 1.19)	0.97 (0.90, 1.03)
Diabetes agents - Metformin	0.87 (0.82, 0.92)	1.02 (0.92, 1.12)	1.01 (0.96, 1.07)
Diabetes agents - Other	0.84 (0.78, 0.90)	1.01 (0.89, 1.14)	1.00 (0.93, 1.07)
Diabetes agents - Sulfonylurea	1.10 (1.03, 1.17)	0.98 (0.88, 1.09)	1.01 (0.95, 1.07)
Estrogen	0.81 (0.74, 0.90)	0.90 (0.77, 1.04)	0.92 (0.84, 1.00)
GI - H2 blockers	0.94 (0.88, 1.01)	0.97 (0.86, 1.09)	0.97 (0.91, 1.03)
GI - Proton-pump inhibitors	0.93 (0.89, 0.96)	0.94 (0.88, 1.00)	0.95 (0.92, 0.98)
GI - Sucralfate	0.94 (0.83, 1.08)	1.18 (0.95, 1.47)	0.95 (0.84, 1.08)
Hypnotics	0.92 (0.86, 0.99)	1.01 (0.90, 1.12)	0.99 (0.94, 1.06)
NSAIDs	0.71 (0.68, 0.75)	0.87 (0.81, 0.94)	1.00 (0.96, 1.04)
Opioids	1.06 (1.02, 1.10)	1.05 (0.99, 1.12)	1.02 (0.98, 1.05)
Parkinsonism drugs	0.99 (0.91, 1.09)	1.01 (0.87, 1.18)	1.01 (0.93, 1.10)
Thyroid hormone replacement	1.04 (0.99, 1.08)	1.02 (0.95, 1.09)	0.98 (0.94, 1.02)
**Health care utilization in past 183 days**			
Emergency department visits	0.81 (0.78, 0.84)	0.90 (0.84, 0.95)	1.00 (0.97, 1.04)
Hospitalizations	0.77 (0.73, 0.80)	0.86 (0.80, 0.93)	0.95 (0.91, 0.99)
**Geographic Region**			
Midwest	1.04 (0.98, 1.09)	0.74 (0.67, 0.81)	0.97 (0.92, 1.03)
South	0.67 (0.63, 0.70)	0.77 (0.71, 0.83)	0.88 (0.84, 0.92)
West	0.96 (0.90, 1.03)	0.89 (0.80, 0.99)	1.01 (0.95, 1.08)
Other	0.76 (0.55, 1.04)	0.70 (0.43, 1.16)	0.75 (0.56, 1.02)

© 2023 Lin KJ et al. *JAMA Network Open*.

Characteristics	Odds Ratio (95% Confidence Interval) from Logistic Model		
	Warfarin vs Apixaban	Dabigatran vs Apixaban	Rivaroxaban vs. Apixaban
**Geographic Region**			
Midwest	1.04 (0.98, 1.09)	0.74 (0.67, 0.81)	0.97 (0.92, 1.03)
South	0.67 (0.63, 0.70)	0.77 (0.71, 0.83)	0.88 (0.84, 0.92)
West	0.96 (0.90, 1.03)	0.89 (0.80, 0.99)	1.01 (0.95, 1.08)
Other	0.76 (0.55, 1.04)	0.70 (0.43, 1.16)	0.75 (0.56, 1.02)
**Geographic Region**			
Midwest	1.04 (0.98, 1.09)	0.74 (0.67, 0.81)	0.97 (0.92, 1.03)
South	0.67 (0.63, 0.70)	0.77 (0.71, 0.83)	0.88 (0.84, 0.92)
West	0.96 (0.90, 1.03)	0.89 (0.80, 0.99)	1.01 (0.95, 1.08)
Other	0.76 (0.55, 1.04)	0.70 (0.43, 1.16)	0.75 (0.56, 1.02)
**Employee Classification**			
Salary Union	1.03 (0.91, 1.16)	1.03 (0.84, 1.26)	0.97 (0.87, 1.09)
Salary Other	0.83 (0.73, 0.94)	0.94 (0.77, 1.16)	1.00 (0.89, 1.13)
Hourly Non-Union	1.05 (0.96, 1.15)	1.07 (0.92, 1.24)	1.01 (0.93, 1.10)
Hourly Union	1.09 (1.02, 1.17)	1.00 (0.90, 1.11)	1.02 (0.96, 1.09)
Hourly Other	0.90 (0.72, 1.12)	1.30 (0.96, 1.76)	1.14 (0.94, 1.39)
Non-Union	0.78 (0.73, 0.85)	0.98 (0.87, 1.11)	1.01 (0.94, 1.08)
Union	0.95 (0.80, 1.12)	1.10 (0.86, 1.40)	0.99 (0.86, 1.13)
Unknown	1.03 (0.94, 1.12)	1.28 (1.12, 1.47)	1.29 (1.19, 1.39)
**Employment Status**			
Active Part Time or Seasonal	0.76 (0.52, 1.11)	0.88 (0.48, 1.61)	0.88 (0.65, 1.18)
Early Retiree	0.95 (0.78, 1.16)	0.73 (0.54, 0.98)	1.03 (0.88, 1.19)
Medicare Eligible Retiree	1.00 (0.91, 1.09)	0.91 (0.80, 1.04)	0.91 (0.84, 0.98)
Retiree	1.53 (1.31, 1.80)	1.23 (0.98, 1.56)	0.92 (0.79, 1.08)
COBRA Continuee	1.57 (0.74, 3.36)	1.33 (0.43, 4.16)	0.99 (0.52, 1.92)
Long Term Disability	1.76 (1.01, 3.06)	0.93 (0.37, 2.37)	1.23 (0.75, 2.00)
Surviving Spouse/Dependent	1.01 (0.90, 1.12)	0.87 (0.74, 1.04)	0.91 (0.83, 1.00)
Other/Unknown/Missing	1.17 (1.00, 1.38)	0.88 (0.67, 1.17)	1.06 (0.92, 1.22)
**Health Plan Indicator**			
Health Plan	0.87 (0.74, 1.02)	0.80 (0.61, 1.07)	0.68 (0.59, 0.79)
**MHSA Coverage Indicator**			
Covered/Possible MHSA Claims	1.04 (0.95, 1.14)	1.37 (1.18, 1.61)	0.90 (0.83, 0.98)
Missing	1.43 (1.27, 1.61)	1.04 (0.85, 1.28)	1.04 (0.93, 1.15)
**Plan Indicator**			
Comprehensive	0.79 (0.68, 0.92)	1.10 (0.82, 1.47)	insufficient numbers
EPO	1.15 (0.79, 1.69)	1.23 (0.64, 2.36)	insufficient numbers
HMO	0.87 (0.74, 1.03)	1.14 (0.84, 1.55)	insufficient numbers
POS	0.68 (0.57, 0.81)	1.20 (0.88, 1.65)	insufficient numbers
PPO	0.77 (0.66, 0.89)	1.10 (0.82, 1.47)	insufficient numbers
POS with capitation	0.80 (0.62, 1.03)	2.48 (1.69, 3.64)	insufficient numbers
CDHP	0.73 (0.56, 0.96)	0.86 (0.54, 1.36)	insufficient numbers
HDHP	0.97 (0.70, 1.35)	1.05 (0.61, 1.79)	insufficient numbers
Missing	-	-	insufficient numbers

Abbreviations: ACE, angiotensin converting enzyme; CCI, combined comorbidity; CFI, claims-based frailty index; CKD, chronic kidney disease; COPD, chronic obstructive pulmonary disease; GI, gastrointestinal; NSAID, non-steroidal anti-inflammatory drug; PVD, peripheral vascular disease; SNRI, serotonin-norepinephrine reuptake inhibitor; SSRI, selective serotonin reuptake inhibitor.

© 2023 Lin KJ et al. *JAMA Network Open*.

eTable 4. Study Population With Atrial Fibrillation Treated With Warfarin vs Apixaban Before and After 1:1 Propensity Score Matching Pooled Across Medicare, Optum, and MarketScan Populations*

Characteristics<br>Number of Patients (%)	Before PS Matching			After PS Matching		
	Apixaban (n=508,016)	Warfarin (n=409,948)	St. Diff	Apixaban (n=250,995)	Warfarin (n=250,995)	St. Diff
**Quarter/Year of Cohort Entry Date**						
Q1 2013	377 (0.1%)	27,852 (6.8%)	-0.38	377 (0.2%)	417 (0.2%)	0.00
Q2 2013	2,619 (0.5%)	23,561 (5.7%)	-0.30	2,618 (1.0%)	2,627 (1.0%)	0.00
Q3 2013	3,801 (0.7%)	21,351 (5.2%)	-0.26	3,797 (1.5%)	3,818 (1.5%)	0.00
Q4 2013	5,852 (1.2%)	20,583 (5.0%)	-0.23	5,806 (2.3%)	5,766 (2.3%)	0.00
Q1 2014	8,606 (1.7%)	26,617 (6.5%)	-0.24	8,523 (3.4%)	8,411 (3.4%)	0.00
Q2 2014	11,406 (2.2%)	23,759 (5.8%)	-0.18	10,997 (4.4%)	10,924 (4.4%)	0.00
Q3 2014	12,220 (2.4%)	22,421 (5.5%)	-0.16	11,489 (4.6%)	11,498 (4.6%)	0.00
Q4 2014	15,185 (3.0%)	22,194 (5.4%)	-0.12	13,695 (5.5%)	13,557 (5.4%)	0.00
Q1 2015	18,910 (3.7%)	23,226 (5.7%)	-0.09	15,892 (6.3%)	15,869 (6.3%)	0.00
Q2 2015	20,474 (4.0%)	20,883 (5.1%)	-0.05	16,079 (6.4%)	16,042 (6.4%)	0.00
Q3 2015	21,088 (4.2%)	18,888 (4.6%)	-0.02	15,349 (6.1%)	15,345 (6.1%)	0.00
Q4 2015	23,583 (4.6%)	18,621 (4.5%)	0.00	15,698 (6.3%)	15,789 (6.3%)	0.00
Q1 2016	28,913 (5.7%)	19,988 (4.9%)	0.04	17,667 (7.0%)	17,771 (7.1%)	0.00
Q2 2016	29,527 (5.8%)	17,430 (4.3%)	0.07	15,863 (6.3%)	15,872 (6.3%)	0.00
Q3 2016	28,848 (5.7%)	15,723 (3.8%)	0.09	14,546 (5.8%)	14,582 (5.8%)	0.00
Q4 2016	30,820 (6.1%)	15,449 (3.8%)	0.11	14,309 (5.7%)	14,395 (5.7%)	0.00
Q1 2017	31,413 (6.2%)	15,581 (3.8%)	0.11	14,464 (5.8%)	14,675 (5.8%)	0.00
Q2 2017	31,121 (6.1%)	13,721 (3.3%)	0.13	12,996 (5.2%)	12,965 (5.2%)	0.00
Q3 2017	30,035 (5.9%)	12,680 (3.1%)	0.14	12,192 (4.9%)	12,055 (4.8%)	0.00
Q4 2017	31,447 (6.2%)	12,307 (3.0%)	0.15	11,819 (4.7%)	11,737 (4.7%)	0.00
Q1 2018	8,343 (1.6%)	1,842 (0.4%)	0.12	1,728 (0.7%)	1,804 (0.7%)	0.00
Q2 2018	7,394 (1.5%)	1,491 (0.4%)	0.12	1,478 (0.6%)	1,458 (0.6%)	0.00
Q3 2018	7,007 (1.4%)	1,329 (0.3%)	0.12	1,302 (0.5%)	1,304 (0.5%)	0.00
Q4 2018	7,354 (1.4%)	1,325 (0.3%)	0.12	1,353 (0.5%)	1,301 (0.5%)	0.00
Q1 2019	9,039 (1.8%)	1,418 (0.3%)	0.14	1,466 (0.6%)	1,398 (0.6%)	0.00
Q2 2019	9,012 (1.8%)	1,181 (0.3%)	0.15	1,124 (0.4%)	1,162 (0.5%)	0.00
Q3 2019	8,646 (1.7%)	1,194 (0.3%)	0.14	1,158 (0.5%)	1,175 (0.5%)	0.00
Q4 2019	8,768 (1.7%)	1,026 (0.3%)	0.15	1,012 (0.4%)	1,010 (0.4%)	0.00
Q1 2020	9,876 (1.9%)	1,101 (0.3%)	0.16	1,068 (0.4%)	1,092 (0.4%)	0.00
Q2 2020	7,512 (1.5%)	759 (0.2%)	0.14	766 (0.3%)	755 (0.3%)	0.00
Q3 2020	8,609 (1.7%)	827 (0.2%)	0.15	813 (0.3%)	823 (0.3%)	0.00
Q4 2020	8,803 (1.7%)	764 (0.2%)	0.16	706 (0.3%)	758 (0.3%)	0.00
Q1 2021	12,511 (2.5%)	1,897 (0.5%)	0.17	1,881 (0.7%)	1,887 (0.8%)	0.00
Q2 2021	8,897 (1.8%)	959 (0.2%)	0.15	964 (0.4%)	953 (0.4%)	0.00
**Sociodemographic**						
Mean Age (SD)	77.79 (7.35)	78.17 (7.29)	-0.05	78.09 (7.4)	78.10 (7.30)	0.00
Male	243,147 (47.9%)	207,991 (50.7%)	-0.06	125,132 (49.9%)	124,865 (49.7%)	0.00
Female	264,869 (52.1%)	201,957 (49.3%)	0.06	125,863 (50.1%)	126,130 (50.3%)	0.00
Black	393,640 (86.4%)	331,637 (89.6%)	-0.10	206,224 (89.5%)	206,187 (89.4%)	0.00
White	25,825 (5.7%)	17,443 (4.7%)	0.04	10,827 (4.7%)	10,818 (4.7%)	0.00
Other[A]	36,023 (7.9%)	21,049 (5.7%)	0.09	13,487 (5.9%)	13,533 (5.9%)	0.00
Dual Status[B]	17,495 (5.5%)	20,775 (6.6%)	-0.05	11,430 (5.9%)	11,457 (5.9%)	0.00
**Cardiovascular conditions**						
Acute Myocardial infarction	34,869 (6.9%)	29,466 (7.2%)	-0.01	17,199 (6.9%)	17,277 (6.9%)	0.00
Cardio-ablation	3,528 (0.7%)	1,975 (0.5%)	0.03	1,286 (0.5%)	1,260 (0.5%)	0.00
Cardioversion	37,787 (7.4%)	13,868 (3.4%)	0.18	10,679 (4.3%)	10,414 (4.1%)	0.01
Cerebrovascular disease	136,905 (26.9%)	120,516 (29.4%)	-0.05	70,312 (28.0%)	70,459 (28.1%)	0.00
Congestive heart failure (inpatient)	99,313 (19.5%)	88,595 (21.6%)	-0.05	51,717 (20.6%)	51,651 (20.6%)	0.00
Congestive heart failure (outpatient)	161,398 (31.8%)	152,455 (37.2%)	-0.11	86,644 (34.5%)	86,440 (34.4%)	0.00
Coronary revascularization	17,207 (3.4%)	23,064 (5.6%)	-0.11	11,564 (4.6%)	11,485 (4.6%)	0.00
Hypertension	442,183 (87.0%)	350,670 (85.5%)	0.04	215,906 (86.0%)	215,775 (86.0%)	0.00
Ischemic Heart Disease	232,708 (45.8%)	194,845 (47.5%)	-0.03	117,173 (46.7%)	116,858 (46.6%)	0.00
PVD or PVD Surgery	73,027 (14.4%)	64,720 (15.8%)	-0.04	37,345 (14.9%)	37,351 (14.9%)	0.00
Stroke (inpatient)	45,161 (8.9%)	39,024 (9.5%)	-0.02	22,655 (9.0%)	22,863 (9.1%)	0.00

© 2023 Lin KJ et al. *JAMA Network Open*.

Characteristics Number of Patients (%)	Before PS Matching			After PS Matching		
	Apixaban (n=508,016)	Warfarin (n=409,948)	St. Diff	Apixaban (n=250,995)	Warfarin (n=250,995)	St. Diff
Stroke (outpatient)	74,589 (14.7%)	51,822 (12.6%)	0.06	31,251 (12.5%)	31,324 (12.5%)	0.00
Syncope	58,512 (11.5%)	38,209 (9.3%)	0.07	24,758 (9.9%)	24,920 (9.9%)	0.00
Mean CHA2DS2-VASc score (SD)	4.63 (1.70)	4.77 (1.67)	-0.09	4.71 (1.7)	4.71 (1.66)	0.00
Mean HAS-BLED score (SD)	2.31 (0.74)	2.31 (0.74)	-0.01	2.30 (0.7)	2.30 (0.74)	0.00
**Non-cardiovascular conditions**						
Acute renal failure	72,743 (14.3%)	59,825 (14.6%)	-0.01	36,212 (14.4%)	36,240 (14.4%)	0.00
Alcohol abuse or dependence	11,431 (2.3%)	6,856 (1.7%)	0.04	4,783 (1.9%)	4,799 (1.9%)	0.00
Anemia	136,541 (26.9%)	123,535 (30.1%)	-0.07	72,201 (28.8%)	72,184 (28.8%)	0.00
CKD: stage 3, 4 or unspecified	94,548 (18.6%)	79,429 (19.4%)	-0.02	47,757 (19.0%)	47,525 (18.9%)	0.00
COPD	115,105 (22.7%)	96,339 (23.5%)	-0.02	57,603 (22.9%)	57,315 (22.8%)	0.00
Dementia	41,850 (8.2%)	36,080 (8.8%)	-0.02	21,079 (8.4%)	21,190 (8.4%)	0.00
Diabetes	181,250 (35.7%)	160,233 (39.1%)	-0.07	94,441 (37.6%)	94,494 (37.6%)	0.00
Endoscopy	9,675 (1.9%)	10,440 (2.5%)	-0.04	5,630 (2.2%)	5,754 (2.3%)	0.00
Falls	38,093 (7.5%)	23,421 (5.7%)	0.07	15,256 (6.1%)	15,324 (6.1%)	0.00
Fractures	44,054 (8.7%)	37,805 (9.2%)	-0.02	22,711 (9.0%)	22,817 (9.1%)	0.00
GI bleeding (inpatient)	23,813 (4.7%)	26,941 (6.6%)	-0.08	14,792 (5.9%)	14,750 (5.9%)	0.00
GI bleeding (outpatient)	70,250 (13.8%)	57,813 (14.1%)	-0.01	33,501 (13.3%)	33,629 (13.4%)	0.00
Liver disease	34,267 (6.7%)	25,205 (6.1%)	0.02	15,573 (6.2%)	15,508 (6.2%)	0.00
Malignancy	90,649 (17.8%)	75,273 (18.4%)	-0.01	45,657 (18.2%)	45,764 (18.2%)	0.00
Obesity	103,801 (20.4%)	77,289 (18.9%)	0.04	48,879 (19.5%)	48,953 (19.5%)	0.00
Peptic Ulcer	12,622 (2.5%)	8,520 (2.1%)	0.03	5,622 (2.2%)	5,637 (2.2%)	0.00
Smoking	165,682 (32.6%)	116,578 (28.4%)	0.09	77,467 (30.9%)	77,226 (30.8%)	0.00
Mean CCI score (SD)	3.51 (2.88)	3.54 (2.84)	-0.01	3.48 (2.9)	3.48 (2.83)	0.00
Mean CFI (SD)	0.20 (0.07)	0.21 (0.07)	-0.13	0.21 (0.1)	0.21 (0.07)	0.00
**Cardiovascular medications**						
ACE inhibitors	136,785 (26.9%)	123,812 (30.2%)	-0.07	72,535 (28.9%)	72,450 (28.9%)	0.00
Angiotensin II receptor blockers	30,716 (6.0%)	21,983 (5.4%)	0.03	13,661 (5.4%)	13,623 (5.4%)	0.00
Antiarrhythmic agents	103,472 (20.4%)	72,898 (17.8%)	0.07	47,005 (18.7%)	46,726 (18.6%)	0.00
Anticoagulants, injectable	2,745 (0.5%)	25,323 (6.2%)	-0.32	2,673 (1.1%)	2,733 (1.1%)	0.00
Antiplatelet agent	83,105 (16.4%)	57,072 (13.9%)	0.07	37,072 (14.8%)	36,842 (14.7%)	0.00
Beta-blockers	345,752 (68.1%)	279,473 (68.2%)	0.00	171,519 (68.3%)	171,238 (68.2%)	0.00
Calcium channel blockers	7,609 (1.5%)	7,220 (1.8%)	-0.02	4,418 (1.8%)	4,425 (1.8%)	0.00
Diuretics	271,967 (53.5%)	242,825 (59.2%)	-0.12	143,919 (57.3%)	143,827 (57.3%)	0.00
Fibrates	21,039 (4.1%)	20,289 (4.9%)	-0.04	11,428 (4.6%)	11,430 (4.6%)	0.00
Nitrates	60,964 (12.0%)	56,737 (13.8%)	-0.05	33,127 (13.2%)	32,813 (13.1%)	0.00
Statins	330,451 (65.0%)	266,361 (65.0%)	0.00	164,076 (65.4%)	164,017 (65.3%)	0.00
**Other medications**						
Anticonvulsants	85,439 (16.8%)	65,538 (16.0%)	0.02	41,224 (16.4%)	41,414 (16.5%)	0.00
Antidepressants - Other	45,310 (8.9%)	33,257 (8.1%)	0.03	21,171 (8.4%)	21,200 (8.4%)	0.00
Antidepressants - SSRI/SNRI	105,299 (20.7%)	81,531 (19.9%)	0.02	50,898 (20.3%)	50,832 (20.3%)	0.00
Antidepressants - Tricyclics	13,445 (2.6%)	11,447 (2.8%)	-0.01	6,709 (2.7%)	6,750 (2.7%)	0.00
Antipsychotic agents	14,696 (2.9%)	12,112 (3.0%)	0.00	7,120 (2.8%)	7,289 (2.9%)	0.00
Anxiolytics (except benzodiazepine)	6,875 (1.4%)	4,144 (1.0%)	0.03	2,819 (1.1%)	2,843 (1.1%)	0.00
Anxiolytics - Benzodiazepines	89,772 (17.7%)	66,154 (16.1%)	0.04	43,070 (17.2%)	42,997 (17.1%)	0.00
Bronchodilators	101,364 (20.0%)	77,515 (18.9%)	0.03	47,843 (19.1%)	47,725 (19.0%)	0.00
Corticosteroids, inhaled	113,461 (22.3%)	82,919 (20.2%)	0.05	52,557 (20.9%)	52,626 (21.0%)	0.00
Corticosteroids, oral	166,352 (32.7%)	123,516 (30.1%)	0.06	77,799 (31.0%)	77,813 (31.0%)	0.00
Dementia drugs	25,692 (5.1%)	21,096 (5.1%)	0.00	12,672 (5.0%)	12,798 (5.1%)	0.00
Diabetes agents - Insulin	37,737 (7.4%)	35,562 (8.7%)	-0.05	20,364 (8.1%)	20,365 (8.1%)	0.00
Diabetes agents - Metformin	82,143 (16.2%)	66,437 (16.2%)	0.00	40,724 (16.2%)	40,688 (16.2%)	0.00
Diabetes agents - Other	27,885 (5.5%)	21,739 (5.3%)	0.01	13,439 (5.4%)	13,486 (5.4%)	0.00
Diabetes agents - Sulfonylurea	46,032 (9.1%)	45,829 (11.2%)	-0.07	25,654 (10.2%)	25,591 (10.2%)	0.00
Estrogen	16,493 (3.2%)	10,289 (2.5%)	0.04	6,624 (2.6%)	6,730 (2.7%)	0.00
GI - H2 blockers	40,601 (8.0%)	30,706 (7.5%)	0.02	19,015 (7.6%)	19,093 (7.6%)	0.00
GI - Proton-pump inhibitors	164,437 (32.4%)	128,842 (31.4%)	0.02	80,061 (31.9%)	79,915 (31.8%)	0.00
GI - Sucralfate	10,361 (2.0%)	7,241 (1.8%)	0.02	4,669 (1.9%)	4,588 (1.8%)	0.00
Hypnotics	39,940 (7.9%)	32,234 (7.9%)	0.00	19,257 (7.7%)	19,167 (7.6%)	0.00
NSAIDs	83,312 (16.4%)	53,379 (13.0%)	0.10	34,955 (13.9%)	34,885 (13.9%)	0.00
Opioids	186,427 (36.7%)	163,688 (39.9%)	-0.07	96,939 (38.6%)	96,727 (38.5%)	0.00
Parkinsonism drugs	19,471 (3.8%)	15,593 (3.8%)	0.00	9,566 (3.8%)	9,687 (3.9%)	0.00

© 2023 Lin KJ et al. *JAMA Network Open*.

Characteristics Number of Patients (%)	Before PS Matching			After PS Matching		
	Apixaban (n=508,016)	Warfarin (n=409,948)	St. Diff	Apixaban (n=250,995)	Warfarin (n=250,995)	St. Diff
Thyroid hormone replacement	113,898 (22.4%)	91,086 (22.2%)	0.00	56,383 (22.5%)	56,525 (22.5%)	0.00
**Health care utilization**						
Emergency department visits	236,274 (46.5%)	167,836 (40.9%)	0.11	104,777 (41.7%)	105,108 (41.9%)	0.00
Home Health Day [C]	7,365 (2.3%)	23,566 (7.5%)	-0.24	7,119 (3.7%)	7,239 (3.7%)	0.00
Home oxygen use	18,434 (3.6%)	13,700 (3.3%)	0.02	8,294 (3.3%)	8,362 (3.3%)	0.00
Hospitalizations	259,600 (51.1%)	204,998 (50.0%)	0.02	123,433 (49.2%)	123,123 (49.1%)	0.00
**Geographic Region**						
Northeast	92,453 (18.2%)	86,228 (21.0%)	-0.07	50,722 (20.2%)	51,118 (20.4%)	0.00
Midwest	110,185 (21.7%)	116,770 (28.5%)	-0.16	66,024 (26.3%)	65,599 (26.1%)	0.00
South	213,777 (42.1%)	128,246 (31.3%)	0.23	87,165 (34.7%)	87,114 (34.7%)	0.00
West	91,208 (18.0%)	78,204 (19.1%)	-0.03	46,822 (18.7%)	46,892 (18.7%)	0.00
Other	393 (0.1%)	500 (0.1%)	-0.01	262 (0.1%)	272 (0.1%)	0.00

Abbreviations: ACE, angiotensin converting enzyme; CCI, combined comorbidity; CFI, claims-based frailty index; CKD, chronic kidney disease; COPD, chronic obstructive pulmonary disease; GI, gastrointestinal; NSAID, non-steroidal anti-inflammatory drug; PVD, peripheral vascular disease; SNRI, serotonin-norepinephrine reuptake inhibitor; SSRI, selective serotonin reuptake inhibitor.

[A] Other race category includes Unknown, Other, Asian, Hispanic, North American Native, and Missing Race Categories in Medicare. Asian, Hispanic, Unknown, and Missing categories are included for race in Optum. Race is unavailable in the MarketScan database.

[B] With both Medicare are Medicaid enrollment eligibility

[C] Home Health services received (days)

eTable 5. Study Population With Atrial Fibrillation Treated With Warfarin vs Apixaban Before and After 1:1 Propensity Score Matching in the Medicare Population

Characteristics<br>Number of Patients (%)	Before PS Matching			After PS Matching		
	Apixaban (n=316,772)	Warfarin (n=313,193)	St. Diff	Apixaban (n=193,272)	Warfarin (n=193,272)	St. Diff
**Quarter/Year of Cohort Entry Date**						
Q1 2013	243 (0.1%)	19,537 (6.2%)	-0.35	243 (0.1%)	270 (0.1%)	0.00
Q2 2013	1,914 (0.6%)	16,905 (5.4%)	-0.28	1,914 (1.0%)	1,889 (1.0%)	0.00
Q3 2013	2,791 (0.9%)	14,994 (4.8%)	-0.24	2,789 (1.4%)	2,800 (1.4%)	0.00
Q4 2013	4,310 (1.4%)	14,601 (4.7%)	-0.19	4,280 (2.2%)	4,152 (2.1%)	0.01
Q1 2014	6,905 (2.2%)	21,574 (6.9%)	-0.23	6,857 (3.5%)	6,771 (3.5%)	0.00
Q2 2014	9,269 (2.9%)	19,476 (6.2%)	-0.16	8,986 (4.6%)	8,919 (4.6%)	0.00
Q3 2014	9,924 (3.1%)	18,190 (5.8%)	-0.13	9,383 (4.9%)	9,363 (4.8%)	0.00
Q4 2014	12,265 (3.9%)	18,051 (5.8%)	-0.09	11,199 (5.8%)	11,053 (5.7%)	0.00
Q1 2015	15,741 (5.0%)	19,036 (6.1%)	-0.05	13,339 (6.9%)	13,306 (6.9%)	0.00
Q2 2015	17,184 (5.4%)	17,368 (5.5%)	0.00	13,569 (7.0%)	13,519 (7.0%)	0.00
Q3 2015	17,555 (5.5%)	15,708 (5.0%)	0.02	12,900 (6.7%)	12,892 (6.7%)	0.00
Q4 2015	19,843 (6.3%)	15,554 (5.0%)	0.06	13,196 (6.8%)	13,305 (6.9%)	0.00
Q1 2016	23,901 (7.5%)	16,362 (5.2%)	0.09	14,545 (7.5%)	14,626 (7.6%)	0.00
Q2 2016	24,679 (7.8%)	14,526 (4.6%)	0.13	13,247 (6.9%)	13,297 (6.9%)	0.00
Q3 2016	23,809 (7.5%)	13,053 (4.2%)	0.14	12,071 (6.2%)	12,145 (6.3%)	0.00
Q4 2016	25,593 (8.1%)	12,860 (4.1%)	0.17	11,890 (6.2%)	12,032 (6.2%)	0.00
Q1 2017	25,373 (8.0%)	12,792 (4.1%)	0.16	11,833 (6.1%)	12,020 (6.2%)	0.00
Q2 2017	25,440 (8.0%)	11,468 (3.7%)	0.18	10,848 (5.6%)	10,831 (5.6%)	0.00
Q3 2017	24,400 (7.7%)	10,719 (3.4%)	0.19	10,241 (5.3%)	10,163 (5.3%)	0.00
Q4 2017	25,633 (8.1%)	10,419 (3.3%)	0.21	9,942 (5.1%)	9,919 (5.1%)	0.00
**Sociodemographic**						
Median Age (SD)	78.09 (7.48)	78.26 (7.39)	-0.02	78.18 (7.47)	78.20 (7.39)	0.00
Male	146,196 (46.2%)	154,539 (49.3%)	-0.06	93,757 (48.5%)	93,721 (48.5%)	0.00
Female	170,576 (53.8%)	158,654 (50.7%)	0.06	99,515 (51.5%)	99,551 (51.5%)	0.00
Black	12,989 (4.1%)	13,293 (4.2%)	-0.01	7,972 (4.1%)	7,963 (4.1%)	0.00
White	289,674 (91.4%)	287,459 (91.8%)	-0.01	177,341 (91.8%)	177,353 (91.8%)	0.00
Other[A]	14,109 (4.5%)	12,441 (4.0%)	0.02	7,959 (4.1%)	7,956 (4.1%)	0.00
Dual Status[B]	17,495 (5.5%)	20,775 (6.6%)	-0.05	11,430 (5.9%)	11,457 (5.9%)	0.00
**Cardiovascular conditions**						
Acute Myocardial infarction	21,143 (6.7%)	22,418 (7.2%)	-0.02	13,199 (6.8%)	13,138 (6.8%)	0.00
Cardio-ablation	2,409 (0.8%)	1,591 (0.5%)	0.04	1,048 (0.5%)	1,019 (0.5%)	0.00
Cardioversion	23,734 (7.5%)	9,918 (3.2%)	0.19	7,983 (4.1%)	7,721 (4.0%)	0.01
Cerebrovascular disease	88,182 (27.8%)	95,265 (30.4%)	-0.06	55,561 (28.7%)	55,687 (28.8%)	0.00
Congestive heart failure (inpatient)	66,939 (21.1%)	71,093 (22.7%)	-0.04	41,739 (21.6%)	41,702 (21.6%)	0.00
Congestive heart failure (outpatient)	97,096 (30.7%)	114,617 (36.6%)	-0.13	65,113 (33.7%)	64,997 (33.6%)	0.00
Coronary revascularization	10,768 (3.4%)	18,167 (5.8%)	-0.11	8,950 (4.6%)	8,868 (4.6%)	0.00
Hypertension	273,296 (86.3%)	267,575 (85.4%)	0.03	165,455 (85.6%)	165,336 (85.5%)	0.00
Ischemic Heart Disease	146,122 (46.1%)	149,939 (47.9%)	-0.04	90,488 (46.8%)	90,197 (46.7%)	0.00
PVD or PVD Surgery	44,046 (13.9%)	49,597 (15.8%)	-0.05	28,498 (14.7%)	28,411 (14.7%)	0.00
Stroke (inpatient)	30,193 (9.5%)	31,159 (9.9%)	-0.01	18,239 (9.4%)	18,404 (9.5%)	0.00
Stroke (outpatient)	29,215 (9.2%)	29,701 (9.5%)	-0.01	17,978 (9.3%)	18,027 (9.3%)	0.00
Syncope	36,070 (11.4%)	29,084 (9.3%)	0.07	18,983 (9.8%)	19,061 (9.9%)	0.00
Mean CHA_2DS_2-VASc score (SD)	4.74 (1.72)	4.85 (1.70)	-0.06	4.78 (1.74)	4.78 (1.68)	0.00
Mean HAS-BLED score (SD)	2.30 (0.74)	2.33 (0.75)	-0.04	2.31 (0.75)	2.31 (0.74)	0.00
**Non-cardiovascular conditions**						
Acute renal failure	44,672 (14.1%)	46,883 (15.0%)	-0.03	28,235 (14.6%)	28,230 (14.6%)	0.00
Alcohol abuse or dependence	6,115 (1.9%)	4,871 (1.6%)	0.02	3,461 (1.8%)	3,480 (1.8%)	0.00
Anemia	88,976 (28.1%)	99,130 (31.7%)	-0.08	57,689 (29.8%)	57,756 (29.9%)	0.00
CKD: stage 3, 4 or unspecified	52,355 (16.5%)	58,389 (18.6%)	-0.06	34,677 (17.9%)	34,486 (17.8%)	0.00
COPD	71,283 (22.5%)	74,010 (23.6%)	-0.03	44,323 (22.9%)	44,023 (22.8%)	0.00
Dementia	27,292 (8.6%)	29,106 (9.3%)	-0.02	16,832 (8.7%)	16,987 (8.8%)	0.00
Diabetes	112,950 (35.7%)	123,363 (39.4%)	-0.08	72,789 (37.7%)	72,831 (37.7%)	0.00
Endoscopy	6,849 (2.2%)	8,608 (2.7%)	-0.03	4,642 (2.4%)	4,752 (2.5%)	-0.01
Falls	14,613 (4.6%)	14,216 (4.5%)	0.00	8,949 (4.6%)	9,027 (4.7%)	0.00
Fractures	28,377 (9.0%)	29,521 (9.4%)	-0.01	17,725 (9.2%)	17,863 (9.2%)	0.00
GI bleeding (inpatient)	18,533 (5.9%)	23,086 (7.4%)	-0.06	12,777 (6.6%)	12,730 (6.6%)	0.00
GI bleeding (outpatient)	32,742 (10.3%)	38,407 (12.3%)	-0.06	22,038 (11.4%)	22,083 (11.4%)	0.00
Liver disease	19,507 (6.2%)	19,409 (6.2%)	0.00	11,916 (6.2%)	11,839 (6.1%)	0.00
Malignancy	58,477 (18.5%)	58,359 (18.6%)	0.00	35,698 (18.5%)	35,866 (18.6%)	0.00

© 2023 Lin KJ et al. *JAMA Network Open*.

Characteristics<br>Number of Patients (%)	Before PS Matching			After PS Matching		
	Apixaban<br>(n=316,772)	Warfarin<br>(n=313,193)	St. Diff	Apixaban<br>(n=193,272)	Warfarin<br>(n=193,272)	St. Diff
Obesity	62,743 (19.8%)	61,859 (19.8%)	0.00	38,472 (19.9%)	38,463 (19.9%)	0.00
Peptic Ulcer	7,700 (2.4%)	7,000 (2.2%)	0.01	4,464 (2.3%)	4,495 (2.3%)	0.00
Smoking	110,949 (35.0%)	98,984 (31.6%)	0.07	64,517 (33.4%)	64,333 (33.3%)	0.00
Mean CCI score (SD)	3.30 (2.78)	3.52 (2.84)	-0.08	3.41 (2.85)	3.41 (2.80)	0.00
Mean CFI (SD)	0.20 (0.07)	0.21 (0.07)	-0.14	0.21 (0.07)	0.21 (0.07)	0.00
**Cardiovascular medications**						
ACE inhibitors	86,741 (27.4%)	95,184 (30.4%)	-0.07	56,014 (29.0%)	56,022 (29.0%)	0.00
Angiotensin II receptor blockers	20,184 (6.4%)	17,123 (5.5%)	0.04	10,813 (5.6%)	10,794 (5.6%)	0.00
Antiarrhythmic agents	69,083 (21.8%)	56,928 (18.2%)	0.09	37,095 (19.2%)	36,951 (19.1%)	0.00
Anticoagulants, injectable	1,857 (0.6%)	20,082 (6.4%)	-0.32	1,855 (1.0%)	1,914 (1.0%)	0.00
Antiplatelet agent	53,767 (17.0%)	44,395 (14.2%)	0.08	29,157 (15.1%)	28,940 (15.0%)	0.00
Beta-blockers	217,595 (68.7%)	215,128 (68.7%)	0.00	132,861 (68.7%)	132,711 (68.7%)	0.00
Calcium channel blockers	7,448 (2.4%)	7,075 (2.3%)	0.01	4,354 (2.3%)	4,356 (2.3%)	0.00
Diuretics	174,404 (55.1%)	188,447 (60.2%)	-0.10	112,349 (58.1%)	112,280 (58.1%)	0.00
Fibrates	13,767 (4.3%)	15,660 (5.0%)	-0.03	8,903 (4.6%)	8,938 (4.6%)	0.00
Nitrates	40,950 (12.9%)	44,879 (14.3%)	-0.04	26,342 (13.6%)	26,172 (13.5%)	0.00
Statins	208,381 (65.8%)	205,580 (65.6%)	0.00	127,509 (66.0%)	127,482 (66.0%)	0.00
**Other medications**						
Anticonvulsants	53,588 (16.9%)	51,610 (16.5%)	0.01	32,282 (16.7%)	32,466 (16.8%)	0.00
Antidepressants - Other	28,214 (8.9%)	26,153 (8.4%)	0.02	16,520 (8.5%)	16,526 (8.6%)	0.00
Antidepressants - SSRI/SNRI	67,638 (21.4%)	64,561 (20.6%)	0.02	40,342 (20.9%)	40,282 (20.8%)	0.00
Antidepressants - Tricyclics	9,062 (2.9%)	9,154 (2.9%)	0.00	5,432 (2.8%)	5,470 (2.8%)	0.00
Antipsychotic agents	9,820 (3.1%)	10,085 (3.2%)	-0.01	5,893 (3.0%)	6,029 (3.1%)	-0.01
Anxiolytics (except benzodiazepine)	60,798 (19.2%)	52,523 (16.8%)	0.06	34,715 (18.0%)	34,608 (17.9%)	0.00
Anxiolytics - Benzodiazepines	4,156 (1.3%)	3,312 (1.1%)	0.02	2,224 (1.2%)	2,252 (1.2%)	0.00
Bronchodilators	63,350 (20.0%)	59,797 (19.1%)	0.02	37,271 (19.3%)	37,081 (19.2%)	0.00
Corticosteroids, inhaled	74,053 (23.4%)	64,930 (20.7%)	0.07	41,741 (21.6%)	41,687 (21.6%)	0.00
Corticosteroids, oral	106,874 (33.7%)	97,188 (31.0%)	0.06	61,738 (31.9%)	61,619 (31.9%)	0.00
Dementia drugs	17,359 (5.5%)	16,859 (5.4%)	0.00	10,148 (5.3%)	10,296 (5.3%)	0.00
Diabetes agents - Insulin	24,010 (7.6%)	27,789 (8.9%)	-0.05	15,883 (8.2%)	15,933 (8.2%)	0.00
Diabetes agents - Metformin	49,679 (15.7%)	50,656 (16.2%)	-0.01	31,075 (16.1%)	31,028 (16.1%)	0.00
Diabetes agents - Other	17,837 (5.6%)	16,674 (5.3%)	0.01	10,455 (5.4%)	10,480 (5.4%)	0.00
Diabetes agents - Sulfonylurea	28,951 (9.1%)	35,148 (11.2%)	-0.07	19,695 (10.2%)	19,652 (10.2%)	0.00
Estrogen	11,432 (3.6%)	8,198 (2.6%)	0.06	5,400 (2.8%)	5,503 (2.8%)	0.00
GI - H2 blockers	26,293 (8.3%)	24,666 (7.9%)	0.01	15,309 (7.9%)	15,350 (7.9%)	0.00
GI - Proton-pump inhibitors	106,684 (33.7%)	101,982 (32.6%)	0.02	63,620 (32.9%)	63,400 (32.8%)	0.00
GI - Sucralfate	6,812 (2.2%)	5,881 (1.9%)	0.02	3,824 (2.0%)	3,727 (1.9%)	0.01
Hypnotics	27,508 (8.7%)	25,809 (8.2%)	0.02	15,698 (8.1%)	15,627 (8.1%)	0.00
NSAIDs	53,329 (16.8%)	41,976 (13.4%)	0.10	27,813 (14.4%)	27,606 (14.3%)	0.00
Opioids	122,499 (38.7%)	127,804 (40.8%)	-0.04	76,271 (39.5%)	76,205 (39.4%)	0.00
Parkinsonism drugs	12,816 (4.0%)	12,427 (4.0%)	0.00	7,597 (3.9%)	7,765 (4.0%)	-0.01
Thyroid hormone replacement	73,843 (23.3%)	70,993 (22.7%)	0.01	44,434 (23.0%)	44,495 (23.0%)	0.00
**Health care utilization**						
Emergency department visits	130,423 (41.2%)	120,201 (38.4%)	0.06	75,806 (39.2%)	75,954 (39.3%)	0.00
Home health day[C]	7,365 (2.3%)	23,566 (7.5%)	-0.24	7,119 (3.7%)	7,239 (3.7%)	0.00
Home oxygen use	11,045 (3.5%)	11,043 (3.5%)	0.00	6,490 (3.4%)	6,480 (3.4%)	0.00
Hospitalizations	169,800 (53.6%)	162,456 (51.9%)	0.03	98,484 (51.0%)	98,126 (50.8%)	0.00
**Geographic Region**						
Northeast	61,445 (19.4%)	68,777 (22.0%)	-0.06	40,788 (21.1%)	41,186 (21.3%)	0.00
Midwest	67,506 (21.3%)	89,273 (28.5%)	-0.17	50,176 (26.0%)	49,766 (25.7%)	0.01
South	136,973 (43.2%)	101,895 (32.5%)	0.22	69,439 (35.9%)	69,315 (35.9%)	0.00
West	50,633 (16.0%)	52,922 (16.9%)	-0.02	32,698 (16.9%)	32,828 (17.0%)	0.00
Other	215 (0.1%)	326 (0.1%)	0.00	171 (0.1%)	177 (0.1%)	0.00

Abbreviations: ACE, angiotensin converting enzyme; CCI, combined comorbidity; CFI, claims-based frailty index; CKD, chronic kidney disease; COPD, chronic obstructive pulmonary disease; GI, gastrointestinal; NSAID, non-steroidal anti-inflammatory drug; PVD, peripheral vascular disease; SNRI, serotonin-norepinephrine reuptake inhibitor; SSRI, selective serotonin reuptake inhibitor.

[A] Other race category includes Unknown, Other, Asian, Hispanic, North American Native, and Missing Race Categories in Medicare. Asian, Hispanic, Unknown, and Missing categories are included for race in Optum. Race is unavailable in the MarketScan database.

[B] With both Medicare are Medicaid enrollment eligibility

[C] Home Health services received (days)

© 2023 Lin KJ et al. *JAMA Network Open*.

eTable 6. Study Population With Atrial Fibrillation Treated With Warfarin vs Apixaban Before and After 1:1 Propensity Score Matching in the Optum Population

Characteristics Number of Patients (%)	Before PS Matching			After PS Matching		
	Apixaban (n=138,716)	Warfarin (n=56,936)	St. Diff	Apixaban (n=37,266)	Warfarin (n=37,266)	St. Diff
Quarter/Year of Cohort Entry Date						
Q1 2013	36 (0.0%)	3,352 (5.9%)	-0.35	36 (0.1%)	36 (0.1%)	0.00
Q2 2013	192 (0.1%)	2,795 (4.9%)	-0.31	191 (0.5%)	213 (0.6%)	-0.01
Q3 2013	248 (0.2%)	2,608 (4.6%)	-0.29	248 (0.7%)	256 (0.7%)	0.00
Q4 2013	433 (0.3%)	2,440 (4.3%)	-0.27	433 (1.2%)	454 (1.2%)	0.00
Q1 2014	668 (0.5%)	2,351 (4.1%)	-0.24	659 (1.8%)	664 (1.8%)	0.00
Q2 2014	816 (0.6%)	2,046 (3.6%)	-0.21	790 (2.1%)	823 (2.2%)	-0.01
Q3 2014	899 (0.6%)	2,023 (3.6%)	-0.21	862 (2.3%)	894 (2.4%)	-0.01
Q4 2014	1,062 (0.8%)	1,964 (3.4%)	-0.18	988 (2.7%)	1,012 (2.7%)	0.00
Q1 2015	1,412 (1.0%)	2,592 (4.6%)	-0.22	1,311 (3.5%)	1,302 (3.5%)	0.00
Q2 2015	1,513 (1.1%)	2,064 (3.6%)	-0.17	1,325 (3.6%)	1,331 (3.6%)	0.00
Q3 2015	1,615 (1.2%)	1,873 (3.3%)	-0.14	1,344 (3.6%)	1,330 (3.6%)	0.00
Q4 2015	1,697 (1.2%)	1,822 (3.2%)	-0.14	1,373 (3.7%)	1,370 (3.7%)	0.00
Q1 2016	2,599 (1.9%)	2,244 (3.9%)	-0.12	1,865 (5.0%)	1,891 (5.1%)	0.00
Q2 2016	2,327 (1.7%)	1,763 (3.1%)	-0.09	1,548 (4.2%)	1,517 (4.1%)	0.01
Q3 2016	2,376 (1.7%)	1,617 (2.8%)	-0.07	1,459 (3.9%)	1,449 (3.9%)	0.00
Q4 2016	2,510 (1.8%)	1,591 (2.8%)	-0.07	1,485 (4.0%)	1,431 (3.8%)	0.01
Q1 2017	4,118 (3.0%)	2,170 (3.8%)	-0.04	2,025 (5.4%)	2,063 (5.5%)	0.00
Q2 2017	3,781 (2.7%)	1,711 (3.0%)	-0.02	1,634 (4.4%)	1,616 (4.3%)	0.00
Q3 2017	3,685 (2.7%)	1,482 (2.6%)	0.01	1,463 (3.9%)	1,423 (3.8%)	0.01
Q4 2017	3,668 (2.6%)	1,436 (2.5%)	0.01	1,422 (3.8%)	1,387 (3.7%)	0.01
Q1 2018	6,888 (5.0%)	1,583 (2.8%)	0.11	1,500 (4.0%)	1,550 (4.2%)	-0.01
Q2 2018	5,950 (4.3%)	1,283 (2.3%)	0.11	1,268 (3.4%)	1,255 (3.4%)	0.00
Q3 2018	5,580 (4.0%)	1,125 (2.0%)	0.12	1,083 (2.9%)	1,106 (3.0%)	-0.01
Q4 2018	5,891 (4.2%)	1,149 (2.0%)	0.13	1,188 (3.2%)	1,131 (3.0%)	0.01
Q1 2019	7,356 (5.3%)	1,208 (2.1%)	0.17	1,246 (3.3%)	1,190 (3.2%)	0.01
Q2 2019	7,098 (5.1%)	975 (1.7%)	0.19	929 (2.5%)	960 (2.6%)	-0.01
Q3 2019	6,803 (4.9%)	976 (1.7%)	0.18	953 (2.6%)	960 (2.6%)	0.00
Q4 2019	6,977 (5.0%)	855 (1.5%)	0.20	853 (2.3%)	843 (2.3%)	0.00
Q1 2020	8,304 (6.0%)	976 (1.7%)	0.22	977 (2.6%)	969 (2.6%)	0.00
Q2 2020	6,447 (4.6%)	650 (1.1%)	0.21	652 (1.7%)	647 (1.7%)	0.00
Q3 2020	7,080 (5.1%)	710 (1.2%)	0.22	704 (1.9%)	708 (1.9%)	0.00
Q4 2020	7,279 (5.2%)	646 (1.1%)	0.24	607 (1.6%)	645 (1.7%)	-0.01
Q1 2021	12,511 (9.0%)	1,897 (3.3%)	0.24	1,881 (5.0%)	1,887 (5.1%)	0.00
Q2 2021	8,897 (6.4%)	959 (1.7%)	0.24	964 (2.6%)	953 (2.6%)	0.00
Patient Characteristics						
Mean age (SD)	77.14 (6.84)	77.35 (6.52)	-0.03	77.42 (6.67)	77.38 (6.64)	0.01
Male	69,298 (50.0%)	31,111 (54.6%)	-0.09	20,087 (53.9%)	19,945 (53.5%)	0.01
Female	69,418 (50.0%)	25,825 (45.4%)	0.09	17,179 (46.1%)	17,321 (46.5%)	-0.01
Black	12,836 (9.3%)	4,150 (7.3%)	0.07	2,855 (7.7%)	2,855 (7.7%)	0.00
White	103,966 (74.9%)	44,178 (77.6%)	-0.06	28,883 (77.5%)	28,834 (77.4%)	0.00
Other[A]	21,914 (15.8%)	8,608 (15.1%)	0.02	5,528 (14.8%)	5,577 (15.0%)	-0.01
Cardiovascular conditions						
Acute myocardial infarction	9,967 (7.2%)	4,169 (7.3%)	0.00	2,567 (6.9%)	2,670 (7.2%)	-0.01
Cardio-ablation	827 (0.6%)	228 (0.4%)	0.03	152 (0.4%)	156 (0.4%)	0.00
Cardioversion	9,914 (7.1%)	2,263 (4.0%)	0.14	1,668 (4.5%)	1,676 (4.5%)	0.00
Cerebrovascular disease	35,448 (25.6%)	14,643 (25.7%)	0.00	9,316 (25.0%)	9,414 (25.3%)	-0.01
Congestive heart failure (inpatient)	22,430 (16.2%)	8,927 (15.7%)	0.01	5,753 (15.4%)	5,825 (15.6%)	-0.01
Congestive heart failure (outpatient)	50,399 (36.3%)	24,207 (42.5%)	-0.13	14,951 (40.1%)	14,902 (40.0%)	0.00
Coronary revascularization	4,762 (3.4%)	2,822 (5.0%)	-0.08	1,702 (4.6%)	1,698 (4.6%)	0.00
Hypertension	123,751 (89.2%)	50,161 (88.1%)	0.03	32,953 (88.4%)	32,915 (88.3%)	0.00
Ischemic Heart Disease	62,952 (45.4%)	26,047 (45.7%)	-0.01	17,048 (45.7%)	17,011 (45.6%)	0.00
PVD or PVD Surgery	22,221 (16.0%)	9,198 (16.2%)	-0.01	5,929 (15.9%)	5,999 (16.1%)	-0.01
Stroke (inpatient)	9,924 (7.2%)	3,828 (6.7%)	0.02	2,441 (6.6%)	2,486 (6.7%)	0.00
Stroke (outpatient)	33,999 (24.5%)	13,585 (23.9%)	0.01	8,767 (23.5%)	8,858 (23.8%)	-0.01

© 2023 Lin KJ et al. *JAMA Network Open*.

Characteristics Number of Patients (%)	Before PS Matching			After PS Matching		
	Apixaban (n=138,716)	Warfarin (n=56,936)	St. Diff	Apixaban (n=37,266)	Warfarin (n=37,266)	St. Diff
Syncope	16,273 (11.7%)	5,373 (9.4%)	0.07	3,692 (9.9%)	3,769 (10.1%)	-0.01
Mean CHA_2DS_2-VASc score (SD)	4.78 (1.69)	4.76 (1.64)	0.01	4.76 (1.69)	4.77 (1.64)	-0.01
Mean HAS-BLED score (SD)	2.35 (0.74)	2.31 (0.72)	0.05	2.32 (0.73)	2.32 (0.73)	0.00
**Non-cardiovascular conditions**						
Acute renal failure	21,935 (15.8%)	8,139 (14.3%)	0.04	5,436 (14.6%)	5,504 (14.8%)	-0.01
Alcohol abuse or dependence	4,393 (3.2%)	1,398 (2.5%)	0.04	1,011 (2.7%)	995 (2.7%)	0.00
Anemia	36,595 (26.4%)	15,174 (26.7%)	-0.01	9,809 (26.3%)	9,824 (26.4%)	0.00
CKD: stage 3, 4 or unspecified	34,142 (24.6%)	14,249 (25.0%)	-0.01	9,543 (25.6%)	9,529 (25.6%)	0.00
COPD	33,325 (24.0%)	13,850 (24.3%)	-0.01	8,984 (24.1%)	8,985 (24.1%)	0.00
Dementia	11,259 (8.1%)	4,127 (7.2%)	0.03	2,776 (7.4%)	2,771 (7.4%)	0.00
Diabetes	51,685 (37.3%)	22,659 (39.8%)	-0.05	14,476 (38.8%)	14,542 (39.0%)	0.00
Endoscopy	2,179 (1.6%)	1,126 (2.0%)	-0.03	666 (1.8%)	680 (1.8%)	0.00
Falls	19,331 (13.9%)	6,421 (11.3%)	0.08	4,693 (12.6%)	4,685 (12.6%)	0.00
Fractures	11,377 (8.2%)	4,811 (8.4%)	-0.01	3,210 (8.6%)	3,187 (8.6%)	0.00
GI bleeding (inpatient)	3,581 (2.6%)	1,777 (3.1%)	-0.03	1,091 (2.9%)	1,122 (3.0%)	-0.01
GI bleeding (outpatient)	30,739 (22.2%)	13,528 (23.8%)	-0.04	8,545 (22.9%)	8,619 (23.1%)	0.00
Liver disease	11,568 (8.3%)	3,654 (6.4%)	0.07	2,498 (6.7%)	2,553 (6.9%)	-0.01
Malignancy	22,530 (16.2%)	9,141 (16.1%)	0.00	6,018 (16.1%)	5,977 (16.0%)	0.00
Obesity	33,425 (24.1%)	10,933 (19.2%)	0.12	7,754 (20.8%)	7,829 (21.0%)	0.00
Peptic Ulcer	4,081 (2.9%)	1,121 (2.0%)	0.06	893 (2.4%)	887 (2.4%)	0.00
Smoking	47,355 (34.1%)	13,592 (23.9%)	0.23	10,468 (28.1%)	10,497 (28.2%)	0.00
Mean CCI score (SD)	4.11 (3.14)	3.84 (2.96)	0.09	3.96 (3.08)	3.97 (3.03)	0.00
Mean CFI (SD)	0.20 (0.07)	0.20 (0.06)	0.00	0.20 (0.07)	0.20 (0.06)	0.00
**Cardiovascular medications**						
ACE inhibitors	36,759 (26.5%)	17,355 (30.5%)	-0.09	10,842 (29.1%)	10,804 (29.0%)	0.00
Angiotensin II receptor blockers	7,203 (5.2%)	2,419 (4.2%)	0.05	1,617 (4.3%)	1,621 (4.3%)	0.00
Antiarrhythmic agents	24,764 (17.9%)	8,764 (15.4%)	0.07	6,060 (16.3%)	5,985 (16.1%)	0.01
Anticoagulants, injectable	646 (0.5%)	2,815 (4.9%)	-0.27	580 (1.6%)	569 (1.5%)	0.01
Antiplatelet agent	19,954 (14.4%)	6,881 (12.1%)	0.07	4,574 (12.3%)	4,628 (12.4%)	0.00
Beta-blockers	92,778 (66.9%)	37,702 (66.2%)	0.01	24,803 (66.6%)	24,739 (66.4%)	0.00
Calcium channel blockers	58 (0.0%)	37 (0.1%)	-0.04	19 (0.1%)	21 (0.1%)	0.00
Diuretics	70,934 (51.1%)	31,450 (55.2%)	-0.08	20,136 (54.0%)	20,102 (53.9%)	0.00
Fibrates	5,010 (3.6%)	2,495 (4.4%)	-0.04	1,491 (4.0%)	1,487 (4.0%)	0.00
Nitrates	14,160 (10.2%)	6,489 (11.4%)	-0.04	4,126 (11.1%)	4,028 (10.8%)	0.01
Statins	88,401 (63.7%)	35,343 (62.1%)	0.03	23,407 (62.8%)	23,338 (62.6%)	0.00
**Other medications**						
Anticonvulsants	24,260 (17.5%)	8,517 (15.0%)	0.07	5,972 (16.0%)	6,010 (16.1%)	0.00
Antidepressants - Other	13,225 (9.5%)	4,526 (7.9%)	0.06	3,201 (8.6%)	3,221 (8.6%)	0.00
Antidepressants - SSRI/SNRI	27,904 (20.1%)	9,996 (17.6%)	0.06	6,796 (18.2%)	6,848 (18.4%)	-0.01
Antidepressants - Tricyclics	3,185 (2.3%)	1,302 (2.3%)	0.00	800 (2.1%)	809 (2.2%)	-0.01
Antipsychotic agents	3,674 (2.6%)	1,164 (2.0%)	0.04	762 (2.0%)	809 (2.2%)	-0.01
Anxiolytics - Benzodiazepines	20,777 (15.0%)	7,491 (13.2%)	0.05	5,113 (13.7%)	5,236 (14.1%)	-0.01
Anxiolytics (except benzodiazepine)	2,124 (1.5%)	513 (0.9%)	0.06	390 (1.0%)	396 (1.1%)	-0.01
Bronchodilators	27,842 (20.1%)	10,347 (18.2%)	0.05	6,724 (18.0%)	6,774 (18.2%)	-0.01
Corticosteroids, inhaled	27,639 (19.9%)	9,840 (17.3%)	0.07	6,473 (17.4%)	6,547 (17.6%)	-0.01
Corticosteroids, oral	42,581 (30.7%)	14,912 (26.2%)	0.10	9,965 (26.7%)	10,107 (27.1%)	-0.01
Dementia drugs	6,069 (4.4%)	2,236 (3.9%)	0.03	1,503 (4.0%)	1,499 (4.0%)	0.00
Diabetes agents - Insulin	10,020 (7.2%)	4,439 (7.8%)	-0.02	2,789 (7.5%)	2,788 (7.5%)	0.00
Diabetes agents - Metformin	24,320 (17.5%)	9,881 (17.4%)	0.00	6,493 (17.4%)	6,520 (17.5%)	0.00
Diabetes agents - Other	12,730 (9.2%)	6,335 (11.1%)	-0.06	3,868 (10.4%)	3,885 (10.4%)	0.00
Diabetes agents - Sulfonylurea	6,951 (5.0%)	2,587 (4.5%)	0.02	1,712 (4.6%)	1,712 (4.6%)	0.00
Estrogen	3,147 (2.3%)	988 (1.7%)	0.04	620 (1.7%)	604 (1.6%)	0.01
GI - H2 blockers	10,525 (7.6%)	3,637 (6.4%)	0.05	2,469 (6.6%)	2,483 (6.7%)	0.00
GI - Proton-pump inhibitors	41,701 (30.1%)	15,447 (27.1%)	0.07	10,329 (27.7%)	10,436 (28.0%)	-0.01
GI - Sucralfate	2,631 (1.9%)	770 (1.4%)	0.04	526 (1.4%)	540 (1.4%)	0.00

© 2023 Lin KJ et al. *JAMA Network Open*.

Characteristics<br>Number of Patients (%)	Before PS Matching			After PS Matching		
	Apixaban<br>(n=138,716)	Warfarin<br>(n=56,936)	St. Diff	Apixaban<br>(n=37,266)	Warfarin<br>(n=37,266)	St. Diff
Hypnotics	8,549 (6.2%)	3,416 (6.0%)	0.01	2,047 (5.5%)	2,036 (5.5%)	0.00
NSAIDs	21,518 (15.5%)	6,668 (11.7%)	0.11	4,478 (12.0%)	4,490 (12.0%)	0.00
Opioids	45,178 (32.6%)	19,983 (35.1%)	-0.05	12,660 (34.0%)	12,578 (33.8%)	0.00
Parkinsonism drugs	4,844 (3.5%)	1,762 (3.1%)	0.02	1,220 (3.3%)	1,194 (3.2%)	0.01
Thyroid hormone replacement	28,894 (20.8%)	11,494 (20.2%)	0.01	7,538 (20.2%)	7,585 (20.4%)	0.00
**Health care utilization**						
Emergency department visits	77,013 (55.5%)	27,806 (48.8%)	0.13	18,475 (49.6%)	18,626 (50.0%)	-0.01
Home oxygen use	6,598 (4.8%)	2,044 (3.6%)	0.06	1,486 (4.0%)	1,537 (4.1%)	-0.01
Hospitalizations	64,284 (46.3%)	23,920 (42.0%)	0.09	15,411 (41.4%)	15,566 (41.8%)	-0.01
**Geographic Region**						
Northeast	16,653 (12.0%)	8,041 (14.1%)	-0.06	5,188 (13.9%)	5,119 (13.7%)	0.01
Midwest	26,387 (19.0%)	12,784 (22.5%)	-0.09	8,328 (22.3%)	8,391 (22.5%)	0.00
South	60,490 (43.6%)	16,958 (29.8%)	0.29	12,164 (32.6%)	12,231 (32.8%)	0.00
West	35,113 (25.3%)	19,113 (33.6%)	-0.18	11,558 (31.0%)	11,494 (30.8%)	0.00
Other	73 (0.1%)	40 (0.1%)	0.00	28 (0.1%)	31 (0.1%)	0.00
**Business Type Code (Recategorized)**						
Commercial	8,722 (6.3%)	3,748 (6.6%)	-0.01	2,300 (6.2%)	2,338 (6.3%)	0.00
Medicare	129,994 (93.7%)	53,188 (93.4%)	0.01	34,966 (93.8%)	34,928 (93.7%)	0.00

Abbreviations: ACE, angiotensin converting enzyme; CCI, combined comorbidity; CFI, claims-based frailty index; CKD, chronic kidney disease; COPD, chronic obstructive pulmonary disease; GI, gastrointestinal; NSAID, non-steroidal anti-inflammatory drug; PVD, peripheral vascular disease; SNRI, serotonin-norepinephrine reuptake inhibitor; SSRI, selective serotonin reuptake inhibitor.

[A] Other race category includes Unknown, Other, Asian, Hispanic, North American Native, and Missing Race Categories in Medicare. Asian, Hispanic, Unknown, and Missing categories are included for race in Optum. Race is unavailable in the MarketScan database.

© 2023 Lin KJ et al. *JAMA Network Open*.

eTable 7. Study Population With Atrial Fibrillation Treated With Warfarin vs Apixaban Before and After 1:1 Propensity Score Matching in the MarketScan Population

Characteristics Number of Patients (%)	Before PS Matching			After PS Matching		
	Apixaban (n=52,528)	Warfarin (n=39,819)	St. Diff	Apixaban (n=20,457)	Warfarin (n=20,457)	St. Diff
**Quarter/Year of Cohort Entry Date**						
Q1 2013	98 (0.2%)	4,963 (12.5%)	-0.52	98 (0.5%)	111 (0.5%)	0.00
Q2 2013	513 (1.0%)	3,861 (9.7%)	-0.39	513 (2.5%)	525 (2.6%)	-0.01
Q3 2013	762 (1.5%)	3,749 (9.4%)	-0.35	760 (3.7%)	762 (3.7%)	0.00
Q4 2013	1,109 (2.1%)	3,542 (8.9%)	-0.30	1,093 (5.3%)	1,160 (5.7%)	-0.02
Q1 2014	1,033 (2.0%)	2,692 (6.8%)	-0.24	1,007 (4.9%)	976 (4.8%)	0.00
Q2 2014	1,321 (2.5%)	2,237 (5.6%)	-0.16	1,221 (6.0%)	1,182 (5.8%)	0.01
Q3 2014	1,397 (2.7%)	2,208 (5.5%)	-0.14	1,244 (6.1%)	1,241 (6.1%)	0.00
Q4 2014	1,858 (3.5%)	2,179 (5.5%)	-0.10	1,508 (7.4%)	1,492 (7.3%)	0.00
Q1 2015	1,757 (3.3%)	1,598 (4.0%)	-0.04	1,242 (6.1%)	1,261 (6.2%)	0.00
Q2 2015	1,777 (3.4%)	1,451 (3.6%)	-0.01	1,185 (5.8%)	1,192 (5.8%)	0.00
Q3 2015	1,918 (3.7%)	1,307 (3.3%)	0.02	1,105 (5.4%)	1,123 (5.5%)	0.00
Q4 2015	2,043 (3.9%)	1,245 (3.1%)	0.04	1,129 (5.5%)	1,114 (5.4%)	0.00
Q1 2016	2,413 (4.6%)	1,382 (3.5%)	0.06	1,257 (6.1%)	1,254 (6.1%)	0.00
Q2 2016	2,521 (4.8%)	1,141 (2.9%)	0.10	1,068 (5.2%)	1,058 (5.2%)	0.00
Q3 2016	2,663 (5.1%)	1,053 (2.6%)	0.13	1,016 (5.0%)	988 (4.8%)	0.01
Q4 2016	2,717 (5.2%)	998 (2.5%)	0.14	934 (4.6%)	932 (4.6%)	0.00
Q1 2017	1,922 (3.7%)	619 (1.6%)	0.13	606 (3.0%)	592 (2.9%)	0.01
Q2 2017	1,900 (3.6%)	542 (1.4%)	0.14	514 (2.5%)	518 (2.5%)	0.00
Q3 2017	1,950 (3.7%)	479 (1.2%)	0.16	488 (2.4%)	469 (2.3%)	0.01
Q4 2017	2,146 (4.1%)	452 (1.1%)	0.19	455 (2.2%)	431 (2.1%)	0.01
Q1 2018	1,455 (2.8%)	259 (0.7%)	0.16	228 (1.1%)	254 (1.2%)	-0.01
Q2 2018	1,444 (2.7%)	208 (0.5%)	0.18	210 (1.0%)	203 (1.0%)	0.00
Q3 2018	1,427 (2.7%)	204 (0.5%)	0.18	219 (1.1%)	198 (1.0%)	0.01
Q4 2018	1,463 (2.8%)	176 (0.4%)	0.19	165 (0.8%)	170 (0.8%)	0.00
Q1 2019	1,683 (3.2%)	210 (0.5%)	0.20	220 (1.1%)	208 (1.0%)	0.01
Q2 2019	1,914 (3.6%)	206 (0.5%)	0.22	195 (1.0%)	202 (1.0%)	0.00
Q3 2019	1,843 (3.5%)	218 (0.5%)	0.22	205 (1.0%)	215 (1.1%)	-0.01
Q4 2019	1,791 (3.4%)	171 (0.4%)	0.22	159 (0.8%)	167 (0.8%)	0.00
Q1 2020	1,572 (3.0%)	125 (0.3%)	0.21	91 (0.4%)	123 (0.6%)	-0.03
Q2 2020	1,065 (2.0%)	109 (0.3%)	0.16	114 (0.6%)	108 (0.5%)	0.01
Q3 2020	1,529 (2.9%)	117 (0.3%)	0.21	109 (0.5%)	115 (0.6%)	-0.01
Q4 2020	1,524 (2.9%)	118 (0.3%)	0.21	99 (0.5%)	113 (0.6%)	-0.01
**Sociodemographic**						
Mean Age (SD)	77.68 (7.81)	78.67 (7.52)	-0.13	78.45 (7.80)	78.46 (7.57)	0.00
Male	27,653 (52.6%)	22,341 (56.1%)	-0.07	11,288 (55.2%)	11,199 (54.7%)	0.01
Female	24,875 (47.4%)	17,478 (43.9%)	0.07	9,169 (44.8%)	9,258 (45.3%)	-0.01
**Cardiovascular conditions**						
Acute Myocardial infarction	3,759 (7.2%)	2,879 (7.2%)	0.00	1,433 (7.0%)	1,469 (7.2%)	-0.01
Cardio-ablation	292 (0.6%)	156 (0.4%)	0.03	86 (0.4%)	85 (0.4%)	0.00
Cardioversion	4,139 (7.9%)	1,687 (4.2%)	0.16	1,028 (5.0%)	1,017 (5.0%)	0.00
Cerebrovascular disease	13,275 (25.3%)	10,608 (26.6%)	-0.03	5,435 (26.6%)	5,358 (26.2%)	0.01
Congestive heart failure (inpatient)	9,944 (18.9%)	8,575 (21.5%)	-0.06	4,225 (20.7%)	4,124 (20.2%)	0.01
Congestive heart failure (outpatient)	13,903 (26.5%)	13,631 (34.2%)	-0.17	6,580 (32.2%)	6,541 (32.0%)	0.00
Coronary revascularization	1,677 (3.2%)	2,075 (5.2%)	-0.10	912 (4.5%)	919 (4.5%)	0.00
Hypertension	45,136 (85.9%)	32,934 (82.7%)	0.09	17,498 (85.5%)	17,524 (85.7%)	-0.01
Ischemic Heart Disease	23,634 (45.0%)	18,859 (47.4%)	-0.05	9,637 (47.1%)	9,650 (47.2%)	0.00
PVD or PVD Surgery	6,760 (12.9%)	5,925 (14.9%)	-0.06	2,918 (14.3%)	2,941 (14.4%)	0.00
Stroke (inpatient)	5,044 (9.6%)	4,037 (10.1%)	-0.02	1,975 (9.7%)	1,973 (9.6%)	0.00
Stroke (outpatient)	11,375 (21.7%)	8,536 (21.4%)	0.01	4,506 (22.0%)	4,439 (21.7%)	0.01
Syncope	6,169 (11.7%)	3,752 (9.4%)	0.07	2,083 (10.2%)	2,090 (10.2%)	0.00
Mean CHA_2DS_2-VASc score (SD)	3.53 (1.60)	4.16 (1.50)	-0.41	3.96 (1.57)	3.96 (1.53)	0.00
Mean HAS-BLED score (SD)	2.22 (0.70)	2.20 (0.72)	0.03	2.23 (0.71)	2.23 (0.70)	0.00
**Non-cardiovascular conditions**						
Acute renal failure	6,136 (11.7%)	4,803 (12.1%)	-0.01	2,541 (12.4%)	2,506 (12.3%)	0.00
Alcohol abuse or dependence	923 (1.8%)	587 (1.5%)	0.02	311 (1.5%)	324 (1.6%)	-0.01
Anemia	10,970 (20.9%)	9,231 (23.2%)	-0.06	4,703 (23.0%)	4,604 (22.5%)	0.01

© 2023 Lin KJ et al. *JAMA Network Open*.

Characteristics<br>Number of Patients (%)	Before PS Matching			After PS Matching		
	Apixaban (n=52,528)	Warfarin (n=39,819)	St. Diff	Apixaban (n=20,457)	Warfarin (n=20,457)	St. Diff
CKD: stage 3, 4 or unspecified	8,051 (15.3%)	6,791 (17.1%)	-0.05	3,537 (17.3%)	3,510 (17.2%)	0.00
COPD	10,497 (20.0%)	8,479 (21.3%)	-0.03	4,296 (21.0%)	4,307 (21.1%)	0.00
Dementia	3,299 (6.3%)	2,847 (7.1%)	-0.03	1,471 (7.2%)	1,432 (7.0%)	0.01
Diabetes	16,615 (31.6%)	14,211 (35.7%)	-0.09	7,176 (35.1%)	7,121 (34.8%)	0.01
Endoscopy	647 (1.2%)	706 (1.8%)	-0.05	322 (1.6%)	322 (1.6%)	0.00
Falls	4,149 (7.9%)	2,784 (7.0%)	0.03	1,614 (7.9%)	1,612 (7.9%)	0.00
Fractures	4,300 (8.2%)	3,473 (8.7%)	-0.02	1,776 (8.7%)	1,767 (8.6%)	0.00
GI bleeding (inpatient)	1,699 (3.2%)	2,078 (5.2%)	-0.10	924 (4.5%)	898 (4.4%)	0.00
GI bleeding (outpatient)	6,769 (12.9%)	5,878 (14.8%)	-0.06	2,918 (14.3%)	2,927 (14.3%)	0.00
Liver disease	3,192 (6.1%)	2,142 (5.4%)	0.03	1,159 (5.7%)	1,116 (5.5%)	0.01
Malignancy	9,642 (18.4%)	7,773 (19.5%)	-0.03	3,941 (19.3%)	3,921 (19.2%)	0.00
Obesity	7,633 (14.5%)	4,497 (11.3%)	0.10	2,653 (13.0%)	2,661 (13.0%)	0.00
Peptic Ulcer	841 (1.6%)	399 (1.0%)	0.05	265 (1.3%)	255 (1.2%)	0.01
Smoking	7,378 (14.0%)	4,002 (10.1%)	0.12	2,482 (12.1%)	2,396 (11.7%)	0.01
Mean CCI score (SD)	3.19 (2.71)	3.24 (2.68)	-0.02	3.27 (2.76)	3.24 (2.71)	0.01
Mean CFI (SD)	0.19 (0.06)	0.20 (0.06)	-0.17	0.20 (0.06)	0.20 (0.06)	0.00
**Cardiovascular medications**						
ACE inhibitors	13,285 (25.3%)	11,273 (28.3%)	-0.07	5,679 (27.8%)	5,624 (27.5%)	0.01
Angiotensin II receptor blockers	3,329 (6.3%)	2,441 (6.1%)	0.01	1,231 (6.0%)	1,208 (5.9%)	0.00
Antiarrhythmic agents	9,625 (18.3%)	7,206 (18.1%)	0.01	3,850 (18.8%)	3,790 (18.5%)	0.01
Anticoagulants, injectable	242 (0.5%)	2,426 (6.1%)	-0.32	238 (1.2%)	250 (1.2%)	0.00
Antiplatelet agent	9,384 (17.9%)	5,796 (14.6%)	0.09	3,341 (16.3%)	3,274 (16.0%)	0.01
Beta-blockers	35,379 (67.4%)	26,643 (66.9%)	0.01	13,855 (67.7%)	13,788 (67.4%)	0.01
Calcium channel blockers	103 (0.2%)	108 (0.3%)	-0.02	45 (0.2%)	48 (0.2%)	0.00
Diuretics	26,629 (50.7%)	22,928 (57.6%)	-0.14	11,434 (55.9%)	11,445 (55.9%)	0.00
Fibrates	2,262 (4.3%)	2,134 (5.4%)	-0.05	1,034 (5.1%)	1,005 (4.9%)	0.01
Nitrates	33,669 (64.1%)	25,438 (63.9%)	0.00	13,160 (64.3%)	13,197 (64.5%)	0.00
Statins	5,854 (11.1%)	5,369 (13.5%)	-0.07	2,659 (13.0%)	2,613 (12.8%)	0.01
**Other medications**						
Anticonvulsants	7,591 (14.5%)	5,411 (13.6%)	0.03	2,970 (14.5%)	2,938 (14.4%)	0.00
Antidepressants - Other	3,871 (7.4%)	2,578 (6.5%)	0.04	1,450 (7.1%)	1,453 (7.1%)	0.00
Antidepressants - SSRI/SNRI	9,757 (18.6%)	6,974 (17.5%)	0.03	3,760 (18.4%)	3,702 (18.1%)	0.01
Antidepressants - Tricyclics	1,198 (2.3%)	991 (2.5%)	-0.01	477 (2.3%)	471 (2.3%)	0.00
Antipsychotic agents	1,202 (2.3%)	863 (2.2%)	0.01	465 (2.3%)	451 (2.2%)	0.01
Anxiolytics (except benzodiazepine)	8,197 (15.6%)	6,140 (15.4%)	0.01	3,242 (15.8%)	3,153 (15.4%)	0.01
Anxiolytics - Benzodiazepines	595 (1.1%)	319 (0.8%)	0.03	205 (1.0%)	195 (1.0%)	0.00
Bronchodilators	10,172 (19.4%)	7,371 (18.5%)	0.02	3,848 (18.8%)	3,870 (18.9%)	0.00
Corticosteroids, inhaled	11,769 (22.4%)	8,149 (20.5%)	0.05	4,343 (21.2%)	4,392 (21.5%)	-0.01
Corticosteroids, oral	16,897 (32.2%)	11,416 (28.7%)	0.08	6,096 (29.8%)	6,087 (29.8%)	0.00
Dementia drugs	2,264 (4.3%)	2,001 (5.0%)	-0.03	1,021 (5.0%)	1,003 (4.9%)	0.00
Diabetes agents - Insulin	3,707 (7.1%)	3,334 (8.4%)	-0.05	1,692 (8.3%)	1,644 (8.0%)	0.01
Diabetes agents - Metformin	8,144 (15.5%)	5,900 (14.8%)	0.02	3,156 (15.4%)	3,140 (15.3%)	0.00
Diabetes agents - Other	3,097 (5.9%)	2,478 (6.2%)	-0.01	1,272 (6.2%)	1,294 (6.3%)	0.00
Diabetes agents - Sulfonylurea	4,351 (8.3%)	4,346 (10.9%)	-0.09	2,091 (10.2%)	2,054 (10.0%)	0.01
Estrogen	1,914 (3.6%)	1,103 (2.8%)	0.05	604 (3.0%)	623 (3.0%)	0.00
GI - H2 blockers	3,783 (7.2%)	2,403 (6.0%)	0.05	1,237 (6.0%)	1,260 (6.2%)	-0.01
GI - Proton-pump inhibitors	16,052 (30.6%)	11,413 (28.7%)	0.04	6,112 (29.9%)	6,079 (29.7%)	0.00
GI - Sucralfate	918 (1.7%)	590 (1.5%)	0.02	319 (1.6%)	321 (1.6%)	0.00
Hypnotics	3,883 (7.4%)	3,009 (7.6%)	-0.01	1,512 (7.4%)	1,504 (7.4%)	0.00
NSAIDs	8,465 (16.1%)	4,735 (11.9%)	0.12	2,664 (13.0%)	2,789 (13.6%)	-0.02
Opioids	18,750 (35.7%)	15,901 (39.9%)	-0.09	8,008 (39.1%)	7,944 (38.8%)	0.01
Parkinsonism drugs	1,811 (3.4%)	1,404 (3.5%)	-0.01	749 (3.7%)	728 (3.6%)	0.01
Thyroid hormone replacement	11,161 (21.2%)	8,599 (21.6%)	-0.01	4,411 (21.6%)	4,445 (21.7%)	0.00
**Health care utilization**						
Emergency department visits	28,838 (54.9%)	19,829 (49.8%)	0.10	10,496 (51.3%)	10,528 (51.5%)	0.00
Hospitalizations	25,516 (48.6%)	18,622 (46.8%)	0.04	9,538 (46.6%)	9,431 (46.1%)	0.01
**Geographic Region**						
Northeast	14,355 (27.3%)	9,410 (23.6%)	0.09	4,746 (23.2%)	4,813 (23.5%)	-0.01
Midwest	16,292 (31.0%)	14,713 (36.9%)	-0.12	7,520 (36.8%)	7,442 (36.4%)	0.01
South	16,314 (31.1%)	9,393 (23.6%)	0.17	5,562 (27.2%)	5,568 (27.2%)	0.00

© 2023 Lin KJ et al. *JAMA Network Open*.

Characteristics<br>Number of Patients (%)	Before PS Matching			After PS Matching		
	Apixaban<br>(n=52,528)	Warfarin<br>(n=39,819)	St. Diff	Apixaban<br>(n=20,457)	Warfarin<br>(n=20,457)	St. Diff
West	5,462 (10.4%)	6,169 (15.5%)	-0.15	2,566 (12.5%)	2,570 (12.6%)	0.00
Other	105 (0.2%)	134 (0.3%)	-0.02	63 (0.3%)	64 (0.3%)	0.00
**Employee Classification**						
Salary Non-union	4,709 (9.0%)	3,943 (9.9%)	-0.03	2,056 (10.1%)	2,033 (9.9%)	0.01
Salary Union	1,242 (2.4%)	800 (2.0%)	0.03	470 (2.3%)	481 (2.4%)	-0.01
Salary Other	921 (1.8%)	756 (1.9%)	-0.01	413 (2.0%)	412 (2.0%)	0.00
Hourly Non-Union	2,630 (5.0%)	2,394 (6.0%)	-0.04	1,205 (5.9%)	1,187 (5.8%)	0.00
Hourly Union	12,481 (23.8%)	12,954 (32.5%)	-0.19	6,732 (32.9%)	6,572 (32.1%)	0.02
Hourly Other	248 (0.5%)	271 (0.7%)	-0.03	127 (0.6%)	132 (0.6%)	0.00
Non-Union	14,476 (27.6%)	5,888 (14.8%)	0.32	3,597 (17.6%)	3,704 (18.1%)	-0.01
Union	784 (1.5%)	409 (1.0%)	0.05	242 (1.2%)	254 (1.2%)	0.00
Unknown	15,037 (28.6%)	12,404 (31.2%)	-0.06	5,615 (27.4%)	5,682 (27.8%)	-0.01
**Employment Status**						
Active Full Time	3,639 (6.9%)	1,414 (3.6%)	0.15	960 (4.7%)	931 (4.6%)	0.00
Active Part Time or Seasonal	164 (0.3%)	59 (0.1%)	0.04	39 (0.2%)	36 (0.2%)	0.00
Early Retiree	560 (1.1%)	290 (0.7%)	0.04	161 (0.8%)	175 (0.9%)	-0.01
Medicare Eligible Retiree	36,932 (70.3%)	27,248 (68.4%)	0.04	14,537 (71.1%)	14,534 (71.0%)	0.00
Retiree	510 (1.0%)	1,973 (5.0%)	-0.24	354 (1.7%)	373 (1.8%)	-0.01
COBRA Continuee	30 (0.1%)	14 (0.0%)	0.04	11 (0.1%)	9 (0.0%)	0.04
Long Term Disability	42 (0.1%)	35 (0.1%)	0.00	18 (0.1%)	22 (0.1%)	0.00
Surviving Spouse/Dependent	4,813 (9.2%)	3,881 (9.7%)	-0.02	2,100 (10.3%)	2,060 (10.1%)	0.01
Other/Unknown/Missing	5,838 (11.1%)	4,905 (12.3%)	-0.04	2,269 (11.1%)	2,303 (11.3%)	-0.01
**Health Plan Indicator**						
Employer	46,027 (87.6%)	34,544 (86.8%)	0.02	17,956 (87.8%)	17,909 (87.5%)	0.01
Health Plan	6,501 (12.4%)	5,275 (13.2%)	-0.02	2,501 (12.2%)	2,548 (12.5%)	-0.01
**MHSA Coverage Indicator**						
Not Covered/Claims Not Present	2,887 (5.5%)	2,357 (5.9%)	-0.02	1,239 (6.1%)	1,243 (6.1%)	0.00
Covered/Possible MHSA Claims	43,626 (83.1%)	33,354 (83.8%)	-0.02	17,100 (83.6%)	17,068 (83.4%)	0.01
Missing	6,015 (11.5%)	4,108 (10.3%)	0.04	2,118 (10.4%)	2,146 (10.5%)	0.00
**Plan Indicator**						
Basic/major medical	0 (0.0%)	0 (0.0%)	--	0 (0.0%)	0 (0.0%)	--
Comprehensive	17,115 (32.6%)	17,139 (43.0%)	-0.22	8,773 (42.9%)	8,625 (42.2%)	0.01
EPO	120 (0.2%)	85 (0.2%)	0.00	47 (0.2%)	47 (0.2%)	0.00
HMO	5,580 (10.6%)	4,360 (10.9%)	-0.01	1,954 (9.6%)	1,924 (9.4%)	0.01
POS	1,567 (3.0%)	1,285 (3.2%)	-0.01	709 (3.5%)	698 (3.4%)	0.01
PPO	26,199 (49.9%)	15,938 (40.0%)	0.20	8,408 (41.1%)	8,571 (41.9%)	-0.02
POS with capitation	696 (1.3%)	168 (0.4%)	0.10	124 (0.6%)	142 (0.7%)	-0.01
CDHP	383 (0.7%)	168 (0.4%)	0.04	112 (0.5%)	121 (0.6%)	-0.01
HDHP	243 (0.5%)	95 (0.2%)	0.05	60 (0.3%)	59 (0.3%)	0.00
Missing	625 (1.2%)	581 (1.5%)	-0.03	270 (1.3%)	270 (1.3%)	0.00

Abbreviations: ACE, angiotensin converting enzyme; CCI, combined comorbidity; CFI, claims-based frailty index; CKD, chronic kidney disease; COPD, chronic obstructive pulmonary disease; GI, gastrointestinal; NSAID, non-steroidal anti-inflammatory drug; PVD, peripheral vascular disease; SNRI, serotonin-norepinephrine reuptake inhibitor; SSRI, selective serotonin reuptake inhibitor.

eTable 8. Study Population With Atrial Fibrillation Treated With Dabigatran vs Apixaban Before and After 1:1 Propensity Score Matching Pooled Across Medicare, Optum, and MarketScan Populations*

Characteristics Number of Patients (%)	Before PS Matching			After PS Matching		
	Apixaban (n=512,004)	Dabigatran (n=74,251)	St. Diff	Apixaban (n=63,359)	Dabigatran (n=63,359)	St. Diff
**Quarter/Year of Cohort Entry Date**						
Q1 2013	377 (0.1%)	6,841 (9.2%)	-0.44	377 (0.6%)	384 (0.6%)	0.00
Q2 2013	2,619 (0.5%)	5,758 (7.8%)	-0.37	2,599 (4.1%)	2,776 (4.4%)	-0.01
Q3 2013	3,803 (0.7%)	4,843 (6.5%)	-0.31	3,558 (5.6%)	3,608 (5.7%)	0.00
Q4 2013	5,862 (1.1%)	4,360 (5.9%)	-0.26	4,231 (6.7%)	4,157 (6.6%)	0.00
Q1 2014	8,616 (1.7%)	4,423 (6.0%)	-0.22	4,483 (7.1%)	4,408 (7.0%)	0.00
Q2 2014	11,431 (2.2%)	4,029 (5.4%)	-0.17	4,145 (6.5%)	4,029 (6.4%)	0.01
Q3 2014	12,273 (2.4%)	3,153 (4.2%)	-0.10	3,183 (5.0%)	3,153 (5.0%)	0.00
Q4 2014	15,244 (3.0%)	2,742 (3.7%)	-0.04	2,738 (4.3%)	2,742 (4.3%)	0.00
Q1 2015	19,009 (3.7%)	2,909 (3.9%)	-0.01	2,937 (4.6%)	2,909 (4.6%)	0.00
Q2 2015	20,575 (4.0%)	2,483 (3.3%)	0.04	2,475 (3.9%)	2,483 (3.9%)	0.00
Q3 2015	21,203 (4.1%)	2,199 (3.0%)	0.06	2,248 (3.5%)	2,199 (3.5%)	0.00
Q4 2015	23,754 (4.6%)	2,882 (3.9%)	0.04	2,879 (4.5%)	2,882 (4.5%)	0.00
Q1 2016	29,143 (5.7%)	3,940 (5.3%)	0.02	3,983 (6.3%)	3,940 (6.2%)	0.00
Q2 2016	29,796 (5.8%)	3,838 (5.2%)	0.03	3,767 (5.9%)	3,838 (6.1%)	0.00
Q3 2016	29,144 (5.7%)	3,299 (4.4%)	0.06	3,369 (5.3%)	3,299 (5.2%)	0.00
Q4 2016	31,124 (6.1%)	3,286 (4.4%)	0.07	3,219 (5.1%)	3,286 (5.2%)	0.00
Q1 2017	31,751 (6.2%)	3,283 (4.4%)	0.08	3,225 (5.1%)	3,283 (5.2%)	0.00
Q2 2017	31,466 (6.1%)	2,826 (3.8%)	0.11	2,788 (4.4%)	2,826 (4.5%)	0.00
Q3 2017	30,296 (5.9%)	2,495 (3.4%)	0.12	2,550 (4.0%)	2,495 (3.9%)	0.00
Q4 2017	31,785 (6.2%)	2,326 (3.1%)	0.15	2,312 (3.6%)	2,326 (3.7%)	0.00
Q1 2018	8,446 (1.6%)	416 (0.6%)	0.10	413 (0.7%)	416 (0.7%)	0.00
Q2 2018	7,481 (1.5%)	287 (0.4%)	0.11	300 (0.5%)	287 (0.5%)	0.00
Q3 2018	7,062 (1.4%)	252 (0.3%)	0.11	258 (0.4%)	252 (0.4%)	0.00
Q4 2018	7,400 (1.4%)	233 (0.3%)	0.12	223 (0.4%)	233 (0.4%)	0.00
Q1 2019	9,131 (1.8%)	219 (0.3%)	0.15	207 (0.3%)	219 (0.3%)	0.00
Q2 2019	9,090 (1.8%)	164 (0.2%)	0.16	146 (0.2%)	164 (0.3%)	-0.01
Q3 2019	8,714 (1.7%)	179 (0.2%)	0.15	175 (0.3%)	179 (0.3%)	0.00
Q4 2019	8,838 (1.7%)	144 (0.2%)	0.16	139 (0.2%)	144 (0.2%)	0.00
Q1 2020	9,959 (1.9%)	80 (0.1%)	0.18	77 (0.1%)	80 (0.1%)	0.00
Q2 2020	7,576 (1.5%)	61 (0.1%)	0.16	58 (0.1%)	61 (0.1%)	0.00
Q3 2020	8,668 (1.7%)	50 (0.1%)	0.17	48 (0.1%)	50 (0.1%)	0.00
Q4 2020	8,844 (1.7%)	38 (0.1%)	0.18	39 (0.1%)	38 (0.1%)	0.00
Q1 2021	12,561 (2.5%)	179 (0.2%)	0.19	184 (0.3%)	179 (0.3%)	0.00
Q2 2021	8,963 (1.8%)	34 (0.0%)	0.18	26 (0.0%)	34 (0.1%)	-0.01
**Sociodemographic**						
Mean Age (SD)	77.81 (7.35)	76.44 (7.05)	0.19	76.52 (7.1)	76.55 (7.08)	0.00
Male	245,043 (47.9%)	38,764 (52.2%)	-0.09	33,098 (52.2%)	32,911 (51.9%)	0.01
Female	266,961 (52.1%)	35,487 (47.8%)	0.09	30,261 (47.8%)	30,448 (48.1%)	-0.01
Black	26,028 (5.7%)	2,906 (4.4%)	0.06	2,428 (4.3%)	2,487 (4.4%)	-0.01
White	396,736 (86.4%)	58,505 (88.7%)	-0.07	50,444 (89.0%)	50,350 (88.8%)	0.01
Other[A]	36,354 (7.9%)	4,526 (6.9%)	0.04	3,799 (6.7%)	3,834 (6.8%)	0.00
Dual Status[B]	17,725 (5.6%)	3,127 (5.4%)	0.01	2,583 (5.2%)	2,633 (5.3%)	0.00
**Cardiovascular conditions**						
Acute Myocardial infarction	35,166 (6.9%)	3,305 (4.5%)	0.10	2,866 (4.5%)	2,898 (4.6%)	0.00
Cardio-ablation	3,567 (0.7%)	521 (0.7%)	0.00	403 (0.6%)	411 (0.6%)	0.00
Cardioversion	38,074 (7.4%)	4,728 (6.4%)	0.04	4,021 (6.3%)	3,998 (6.3%)	0.00
Cerebrovascular disease	138,373 (27.0%)	19,442 (26.2%)	0.02	16,594 (26.2%)	16,593 (26.2%)	0.00
Congestive heart failure (inpatient)	100,411 (19.6%)	11,465 (15.4%)	0.11	9,577 (15.1%)	9,880 (15.6%)	-0.01
Congestive heart failure (outpatient)	163,200 (31.9%)	22,205 (29.9%)	0.04	18,789 (29.7%)	18,812 (29.7%)	0.00
Coronary revascularization	17,353 (3.4%)	1,884 (2.5%)	0.05	1,589 (2.5%)	1,622 (2.6%)	0.00
Hypertension	445,853 (87.1%)	62,848 (84.6%)	0.07	53,642 (84.7%)	53,643 (84.7%)	0.00
Ischemic Heart Disease	235,030 (45.9%)	32,065 (43.2%)	0.05	27,616 (43.6%)	27,410 (43.3%)	0.01
PVD or PVD Surgery	73,860 (14.4%)	9,502 (12.8%)	0.05	8,111 (12.8%)	8,182 (12.9%)	0.00
Stroke (inpatient)	45,677 (8.9%)	5,587 (7.5%)	0.05	4,803 (7.6%)	4,879 (7.7%)	0.00

© 2023 Lin KJ et al. *JAMA Network Open*.

Characteristics<br>Number of Patients (%)	Before PS Matching			After PS Matching		
	Apixaban<br>(n=512,004)	Dabigatran<br>(n=74,251)	St. Diff	Apixaban<br>(n=63,359)	Dabigatran<br>(n=63,359)	St. Diff
Stroke (outpatient)	75,381 (14.7%)	7,934 (10.7%)	0.12	6,915 (10.9%)	6,890 (10.9%)	0.00
Syncope	58,954 (11.5%)	7,123 (9.6%)	0.06	6,290 (9.9%)	6,197 (9.8%)	0.00
Mean CHA2DS2-VASc score (SD)	4.63 (1.70)	4.40 (1.69)	0.13	4.41 (1.7)	4.42 (1.69)	-0.01
Mean HAS-BLED score (SD)	2.31 (0.74)	2.22 (0.71)	0.12	2.22 (0.7)	2.22 (0.72)	0.00
**Non-cardiovascular conditions**						
Acute renal failure	73,502 (14.4%)	6,425 (8.7%)	0.18	5,665 (8.9%)	5,743 (9.1%)	0.00
Alcohol abuse or dependence	11,553 (2.3%)	1,306 (1.8%)	0.04	1,196 (1.9%)	1,179 (1.9%)	0.00
Anemia	137,958 (26.9%)	17,619 (23.7%)	0.07	15,251 (24.1%)	15,198 (24.0%)	0.00
CKD: stage 3, 4 or unspecified	95,693 (18.7%)	8,761 (11.8%)	0.19	7,676 (12.1%)	7,757 (12.2%)	0.00
COPD	116,315 (22.7%)	15,184 (20.4%)	0.06	12,753 (20.1%)	12,994 (20.5%)	-0.01
Dementia	42,394 (8.3%)	5,037 (6.8%)	0.06	4,239 (6.7%)	4,338 (6.8%)	-0.01
Diabetes	182,983 (35.7%)	26,979 (36.3%)	-0.01	23,017 (36.3%)	22,987 (36.3%)	0.00
Endoscopy	9,786 (1.9%)	1,227 (1.7%)	0.02	1,069 (1.7%)	1,057 (1.7%)	0.00
Falls	38,571 (7.5%)	3,148 (4.2%)	0.14	2,836 (4.5%)	2,830 (4.5%)	0.00
Fractures	44,549 (8.7%)	5,670 (7.6%)	0.04	5,016 (7.9%)	4,946 (7.8%)	0.00
GI bleeding (inpatient)	24,113 (4.7%)	3,394 (4.6%)	0.01	2,848 (4.5%)	2,923 (4.6%)	-0.01
GI bleeding (outpatient)	71,010 (13.9%)	9,516 (12.8%)	0.03	8,082 (12.8%)	8,001 (12.6%)	0.00
Liver disease	34,577 (6.8%)	4,280 (5.8%)	0.04	3,644 (5.8%)	3,738 (5.9%)	-0.01
Malignancy	91,407 (17.9%)	13,053 (17.6%)	0.01	11,228 (17.7%)	11,225 (17.7%)	0.00
Obesity	104,541 (20.4%)	14,031 (18.9%)	0.04	12,129 (19.1%)	12,222 (19.3%)	0.00
Peptic Ulcer	12,767 (2.5%)	1,219 (1.6%)	0.06	1,087 (1.7%)	1,077 (1.7%)	0.00
Smoking	167,160 (32.6%)	19,875 (26.8%)	0.13	17,632 (27.8%)	17,619 (27.8%)	0.00
Mean CCI score (SD)	3.52 (2.88)	2.85 (2.58)	0.25	2.88 (2.6)	2.90 (2.60)	-0.01
Mean CFI (SD)	0.21 (0.07)	0.20 (0.07)	0.11	0.20 (0.1)	0.20 (0.07)	0.00
**Cardiovascular medications**						
ACE inhibitors	138,034 (27.0%)	19,939 (26.9%)	0.00	16,840 (26.6%)	16,933 (26.7%)	0.00
Angiotensin II receptor blockers	30,870 (6.0%)	4,846 (6.5%)	-0.02	4,141 (6.5%)	4,085 (6.4%)	0.00
Antiarrhythmic agents	104,419 (20.4%)	16,269 (21.9%)	-0.04	13,684 (21.6%)	13,731 (21.7%)	0.00
Anticoagulants, injectable	2,861 (0.6%)	567 (0.8%)	-0.03	467 (0.7%)	465 (0.7%)	0.00
Antiplatelet agent	83,913 (16.4%)	10,136 (13.7%)	0.08	8,863 (14.0%)	8,726 (13.8%)	0.01
Beta-blockers	348,675 (68.1%)	48,095 (64.8%)	0.07	41,157 (65.0%)	41,245 (65.1%)	0.00
Calcium channel blockers	7,662 (1.5%)	1,272 (1.7%)	-0.02	1,049 (1.7%)	1,073 (1.7%)	0.00
Diuretics	274,398 (53.6%)	39,288 (52.9%)	0.01	33,405 (52.7%)	33,426 (52.8%)	0.00
Fibrates	21,234 (4.1%)	3,458 (4.7%)	-0.02	2,935 (4.6%)	2,930 (4.6%)	0.00
Nitrates	61,644 (12.0%)	8,545 (11.5%)	0.02	7,375 (11.6%)	7,245 (11.4%)	0.01
Statins	333,253 (65.1%)	47,105 (63.4%)	0.03	40,472 (63.9%)	40,400 (63.8%)	0.00
**Other medications**						
Anticonvulsants	86,235 (16.8%)	11,111 (15.0%)	0.05	9,800 (15.5%)	9,738 (15.4%)	0.00
Antidepressants - Other	45,804 (8.9%)	5,639 (7.6%)	0.05	4,878 (7.7%)	4,951 (7.8%)	0.00
Antidepressants - SSRI/SNRI	106,321 (20.8%)	14,765 (19.9%)	0.02	12,915 (20.4%)	12,783 (20.2%)	0.01
Antidepressants - Tricyclics	13,566 (2.6%)	1,951 (2.6%)	0.00	1,644 (2.6%)	1,627 (2.6%)	0.00
Antipsychotic agents	14,859 (2.9%)	2,073 (2.8%)	0.01	1,783 (2.8%)	1,791 (2.8%)	0.00
Anxiolytics (except benzodiazepine)	6,942 (1.4%)	790 (1.1%)	0.03	685 (1.1%)	699 (1.1%)	0.00
Anxiolytics - Benzodiazepines	90,600 (17.7%)	12,527 (16.9%)	0.02	11,345 (17.9%)	11,225 (17.7%)	0.00
Bronchodilators	102,283 (20.0%)	13,874 (18.7%)	0.03	11,917 (18.8%)	11,914 (18.8%)	0.00
Corticosteroids, inhaled	114,352 (22.3%)	16,323 (22.0%)	0.01	14,178 (22.4%)	13,923 (22.0%)	0.01
Corticosteroids, oral	167,724 (32.8%)	23,225 (31.3%)	0.03	19,909 (31.4%)	19,970 (31.5%)	0.00
Dementia drugs	25,989 (5.1%)	3,673 (4.9%)	0.01	3,086 (4.9%)	3,152 (5.0%)	0.00
Diabetes agents - Insulin	38,133 (7.4%)	5,338 (7.2%)	0.01	4,569 (7.2%)	4,616 (7.3%)	0.00
Diabetes agents - Metformin	82,824 (16.2%)	12,536 (16.9%)	-0.02	10,686 (16.9%)	10,687 (16.9%)	0.00
Diabetes agents - Other	28,103 (5.5%)	4,255 (5.7%)	-0.01	3,481 (5.5%)	3,576 (5.6%)	-0.01
Diabetes agents - Sulfonylurea	46,405 (9.1%)	7,255 (9.8%)	-0.02	6,049 (9.5%)	6,071 (9.6%)	0.00
Estrogen	16,571 (3.2%)	2,479 (3.3%)	-0.01	2,036 (3.2%)	2,086 (3.3%)	0.00
GI - H2 blockers	40,965 (8.0%)	5,202 (7.0%)	0.04	4,543 (7.2%)	4,499 (7.1%)	0.00
GI - Proton-pump inhibitors	165,995 (32.4%)	22,783 (30.7%)	0.04	19,832 (31.3%)	19,592 (30.9%)	0.01
GI - Sucralfate	10,479 (2.0%)	1,405 (1.9%)	0.01	1,229 (1.9%)	1,203 (1.9%)	0.00
Hypnotics	40,249 (7.9%)	6,728 (9.1%)	-0.04	5,552 (8.8%)	5,563 (8.8%)	0.00
NSAIDs	83,763 (16.4%)	12,214 (16.4%)	0.00	10,263 (16.2%)	10,330 (16.3%)	0.00
Opioids	188,087 (36.7%)	28,405 (38.3%)	-0.03	24,135 (38.1%)	24,184 (38.2%)	0.00
Parkinsonism drugs	19,666 (3.8%)	2,825 (3.8%)	0.00	2,444 (3.9%)	2,443 (3.9%)	0.00

© 2023 Lin KJ et al. *JAMA Network Open*.

Characteristics<br>Number of Patients (%)	Before PS Matching			After PS Matching		
	Apixaban (n=512,004)	Dabigatran (n=74,251)	St. Diff	Apixaban (n=63,359)	Dabigatran (n=63,359)	St. Diff
Thyroid hormone replacement	114,986 (22.5%)	15,588 (21.0%)	0.04	13,353 (21.1%)	13,489 (21.3%)	-0.01
**Health care utilization**						
Emergency department visits	238,440 (46.6%)	28,794 (38.8%)	0.16	24,792 (39.1%)	24,831 (39.2%)	0.00
Home Health Day[C]	7,486 (2.3%)	3,652 (6.3%)	-0.20	2,720 (5.5%)	2,738 (5.5%)	0.00
Home oxygen use	18,641 (3.6%)	1,958 (2.6%)	0.06	1,653 (2.6%)	1,708 (2.7%)	-0.01
Hospitalizations	261,794 (51.1%)	31,948 (43.0%)	0.16	26,701 (42.1%)	27,250 (43.0%)	-0.02
**Geographic Region**						
Northeast	93,124 (18.2%)	16,328 (22.0%)	-0.10	13,909 (22.0%)	13,778 (21.7%)	0.01
Midwest	111,174 (21.7%)	16,019 (21.6%)	0.00	13,398 (21.1%)	13,688 (21.6%)	-0.01
South	215,101 (42.0%)	28,403 (38.3%)	0.08	24,779 (39.1%)	24,577 (38.8%)	0.01
West	92,206 (18.0%)	13,407 (18.1%)	0.00	11,190 (17.7%)	11,236 (17.7%)	0.00
Other	399 (0.1%)	94 (0.1%)	-0.02	83 (0.1%)	80 (0.1%)	0.00

Abbreviations: ACE, angiotensin converting enzyme; CCI, combined comorbidity; CFI, claims-based frailty index; CKD, chronic kidney disease; COPD, chronic obstructive pulmonary disease; GI, gastrointestinal; NSAID, non-steroidal anti-inflammatory drug; PVD, peripheral vascular disease; SNRI, serotonin-norepinephrine reuptake inhibitor; SSRI, selective serotonin reuptake inhibitor.

[A] Other race category includes Unknown, Other, Asian, Hispanic, North American Native, and Missing Race Categories in Medicare. Asian, Hispanic, Unknown, and Missing categories are included for race in Optum. Race is unavailable in the MarketScan database.

[B] With both Medicare are Medicaid enrollment eligibility

[C] Home Health services received (days)

© 2023 Lin KJ et al. *JAMA Network Open*.

eTable 9. Study Population With Atrial Fibrillation Treated With Dabigatran vs Apixaban Before and After 1:1 Propensity Score Matching in the Medicare Population

Characteristics<br>Number of Patients (%)	Before PS Matching			After PS Matching		
	Apixaban (n=319,256)	Dabigatran (n=57,728)	St. Diff	Apixaban (n=49,648)	Dabigatran (n=49,648)	St. Diff
**Quarter/Year of Cohort Entry Date**						
Q1 2013	243 (0.1%)	5,089 (8.8%)	-0.43	243 (0.5%)	243 (0.5%)	0.00
Q2 2013	1,914 (0.6%)	4,331 (7.5%)	-0.36	1,911 (3.8%)	2,031 (4.1%)	-0.02
Q3 2013	2,793 (0.9%)	3,518 (6.1%)	-0.29	2,646 (5.3%)	2,686 (5.4%)	0.00
Q4 2013	4,319 (1.4%)	3,235 (5.6%)	-0.23	3,175 (6.4%)	3,140 (6.3%)	0.00
Q1 2014	6,910 (2.2%)	3,530 (6.1%)	-0.20	3,597 (7.2%)	3,523 (7.1%)	0.00
Q2 2014	9,284 (2.9%)	3,300 (5.7%)	-0.14	3,421 (6.9%)	3,300 (6.6%)	0.01
Q3 2014	9,963 (3.1%)	2,523 (4.4%)	-0.07	2,574 (5.2%)	2,523 (5.1%)	0.00
Q4 2014	12,309 (3.9%)	2,217 (3.8%)	0.01	2,182 (4.4%)	2,217 (4.5%)	0.00
Q1 2015	15,828 (5.0%)	2,378 (4.1%)	0.04	2,406 (4.8%)	2,378 (4.8%)	0.00
Q2 2015	17,267 (5.4%)	2,085 (3.6%)	0.09	2,094 (4.2%)	2,085 (4.2%)	0.00
Q3 2015	17,649 (5.5%)	1,812 (3.1%)	0.12	1,867 (3.8%)	1,812 (3.6%)	0.01
Q4 2015	19,982 (6.3%)	2,380 (4.1%)	0.10	2,333 (4.7%)	2,380 (4.8%)	0.00
Q1 2016	24,081 (7.5%)	3,224 (5.6%)	0.08	3,262 (6.6%)	3,224 (6.5%)	0.00
Q2 2016	24,892 (7.8%)	3,197 (5.5%)	0.09	3,143 (6.3%)	3,197 (6.4%)	0.00
Q3 2016	24,060 (7.5%)	2,720 (4.7%)	0.12	2,751 (5.5%)	2,720 (5.5%)	0.00
Q4 2016	25,847 (8.1%)	2,733 (4.7%)	0.14	2,653 (5.3%)	2,733 (5.5%)	-0.01
Q1 2017	25,654 (8.0%)	2,828 (4.9%)	0.13	2,760 (5.6%)	2,828 (5.7%)	0.00
Q2 2017	25,718 (8.1%)	2,443 (4.2%)	0.16	2,405 (4.8%)	2,443 (4.9%)	0.00
Q3 2017	24,627 (7.7%)	2,181 (3.8%)	0.17	2,240 (4.5%)	2,181 (4.4%)	0.00
Q4 2017	25,916 (8.1%)	2,004 (3.5%)	0.20	1,985 (4.0%)	2,004 (4.0%)	0.00
**Sociodemographic**						
Mean age (SD)	78.11 (7.48)	76.58 (7.08)	0.21	76.63 (7.16)	76.67 (7.12)	-0.01
Male	147,355 (46.2%)	29,366 (50.9%)	-0.09	25,295 (50.9%)	25,197 (50.8%)	0.00
Female	171,901 (53.8%)	28,362 (49.1%)	0.09	24,353 (49.1%)	24,451 (49.2%)	0.00
Black	13,098 (4.1%)	2,291 (4.0%)	0.01	1,915 (3.9%)	1,964 (4.0%)	-0.01
White	291,905 (91.4%)	52,322 (90.6%)	0.03	45,144 (90.9%)	45,070 (90.8%)	0.00
Other[A]	14,253 (4.5%)	3,115 (5.4%)	-0.04	2,589 (5.2%)	2,614 (5.3%)	0.00
Dual Status[B]	17,725 (5.6%)	3,127 (5.4%)	0.01	2,583 (5.2%)	2,633 (5.3%)	0.00
**Cardiovascular conditions**						
Acute myocardial infarction	21,328 (6.7%)	2,567 (4.4%)	0.10	2,253 (4.5%)	2,271 (4.6%)	0.00
Cardio-ablation	2,434 (0.8%)	398 (0.7%)	0.01	301 (0.6%)	315 (0.6%)	0.00
Cardioversion	23,932 (7.5%)	3,545 (6.1%)	0.06	3,061 (6.2%)	3,053 (6.1%)	0.00
Cerebrovascular disease	89,124 (27.9%)	15,597 (27.0%)	0.02	13,291 (26.8%)	13,371 (26.9%)	0.00
Congestive heart failure (inpatient)	67,688 (21.2%)	9,324 (16.2%)	0.13	7,904 (15.9%)	8,089 (16.3%)	-0.01
Congestive heart failure (outpatient)	98,144 (30.7%)	17,016 (29.5%)	0.03	14,453 (29.1%)	14,470 (29.1%)	0.00
Coronary revascularization	10,872 (3.4%)	1,541 (2.7%)	0.04	1,318 (2.7%)	1,330 (2.7%)	0.00
Hypertension	275,546 (86.3%)	48,566 (84.1%)	0.06	41,702 (84.0%)	41,757 (84.1%)	0.00
Ischemic Heart Disease	147,558 (46.2%)	25,009 (43.3%)	0.06	21,677 (43.7%)	21,518 (43.3%)	0.01
PVD or PVD Surgery	44,556 (14.0%)	7,398 (12.8%)	0.04	6,349 (12.8%)	6,401 (12.9%)	0.00
Stroke (inpatient)	30,529 (9.6%)	4,516 (7.8%)	0.06	3,902 (7.9%)	3,974 (8.0%)	0.00
Stroke (outpatient)	29,549 (9.3%)	4,597 (8.0%)	0.05	4,013 (8.1%)	4,058 (8.2%)	0.00
Syncope	36,373 (11.4%)	5,495 (9.5%)	0.06	4,873 (9.8%)	4,808 (9.7%)	0.00
Mean CHA_2DS_2-VASc score (SD)	4.74 (1.72)	4.49 (1.72)	0.15	4.49 (1.71)	4.50 (1.72)	-0.01
Mean HAS-BLED score (SD)	2.31 (0.74)	2.23 (0.72)	0.11	2.23 (0.72)	2.23 (0.73)	0.00
**Non-cardiovascular conditions**						
Acute renal failure	45,157 (14.1%)	5,158 (8.9%)	0.16	4,601 (9.3%)	4,639 (9.3%)	0.00
Alcohol abuse or dependence	6,180 (1.9%)	967 (1.7%)	0.02	917 (1.8%)	880 (1.8%)	0.00
Anemia	89,945 (28.2%)	14,363 (24.9%)	0.07	12,528 (25.2%)	12,448 (25.1%)	0.00
CKD: stage 3, 4 or unspecified	52,991 (16.6%)	6,407 (11.1%)	0.16	5,609 (11.3%)	5,709 (11.5%)	-0.01
COPD	72,047 (22.6%)	11,934 (20.7%)	0.05	10,159 (20.5%)	10,277 (20.7%)	0.00
Dementia	27,652 (8.7%)	4,070 (7.1%)	0.06	3,420 (6.9%)	3,518 (7.1%)	-0.01
Diabetes	114,032 (35.7%)	21,224 (36.8%)	-0.02	18,287 (36.8%)	18,215 (36.7%)	0.00
Endoscopy	6,915 (2.2%)	1,027 (1.8%)	0.03	897 (1.8%)	895 (1.8%)	0.00
Falls	14,811 (4.6%)	1,949 (3.4%)	0.06	1,734 (3.5%)	1,759 (3.5%)	0.00
Fractures	28,709 (9.0%)	4,515 (7.8%)	0.04	4,003 (8.1%)	3,957 (8.0%)	0.00
GI bleeding (inpatient)	18,748 (5.9%)	2,942 (5.1%)	0.04	2,480 (5.0%)	2,556 (5.1%)	0.00
GI bleeding (outpatient)	33,149 (10.4%)	6,245 (10.8%)	-0.01	5,324 (10.7%)	5,316 (10.7%)	0.00
Liver disease	19,699 (6.2%)	3,338 (5.8%)	0.02	2,839 (5.7%)	2,927 (5.9%)	-0.01
Malignancy	58,993 (18.5%)	10,232 (17.7%)	0.02	8,931 (18.0%)	8,872 (17.9%)	0.00
Obesity	63,205 (19.8%)	11,432 (19.8%)	0.00	9,926 (20.0%)	9,995 (20.1%)	0.00
Peptic Ulcer	7,790 (2.4%)	1,008 (1.7%)	0.05	897 (1.8%)	884 (1.8%)	0.00

© 2023 Lin KJ et al. *JAMA Network Open*.

Characteristics<br>Number of Patients (%)	Before PS Matching			After PS Matching		
	Apixaban (n=319,256)	Dabigatran (n=57,728)	St. Diff	Apixaban (n=49,648)	Dabigatran (n=49,648)	St. Diff
Smoking	111,963 (35.1%)	17,166 (29.7%)	0.12	15,232 (30.7%)	15,210 (30.6%)	0.00
Mean CCI score (SD)	3.31 (2.78)	2.84 (2.58)	0.18	2.86 (2.56)	2.88 (2.60)	-0.01
Mean CFI (SD)	0.21 (0.07)	0.20 (0.07)	0.14	0.20 (0.07)	0.20 (0.07)	0.00
**Cardiovascular medications**						
ACE inhibitors	87,540 (27.4%)	15,617 (27.1%)	0.01	13,290 (26.8%)	13,353 (26.9%)	0.00
Angiotensin II receptor blockers	20,287 (6.4%)	3,842 (6.7%)	-0.01	3,318 (6.7%)	3,245 (6.5%)	0.01
Antiarrhythmic agents	69,717 (21.8%)	12,862 (22.3%)	-0.01	10,911 (22.0%)	10,919 (22.0%)	0.00
Anticoagulants, injectable	1,939 (0.6%)	456 (0.8%)	-0.02	394 (0.8%)	391 (0.8%)	0.00
Antiplatelet agent	54,328 (17.0%)	8,045 (13.9%)	0.09	7,065 (14.2%)	6,970 (14.0%)	0.01
Beta-blockers	219,466 (68.7%)	37,659 (65.2%)	0.07	32,445 (65.4%)	32,554 (65.6%)	0.00
Calcium channel blockers	7,501 (2.3%)	1,247 (2.2%)	0.01	1,028 (2.1%)	1,053 (2.1%)	0.00
Diuretics	175,957 (55.1%)	31,113 (53.9%)	0.02	26,684 (53.7%)	26,668 (53.7%)	0.00
Fibrates	13,899 (4.4%)	2,684 (4.6%)	-0.01	2,292 (4.6%)	2,292 (4.6%)	0.00
Nitrates	41,422 (13.0%)	6,938 (12.0%)	0.03	5,995 (12.1%)	5,920 (11.9%)	0.01
Statins	210,128 (65.8%)	37,021 (64.1%)	0.04	32,066 (64.6%)	31,975 (64.4%)	0.00
**Other medications**						
Anticonvulsants	54,123 (17.0%)	8,889 (15.4%)	0.04	7,879 (15.9%)	7,854 (15.8%)	0.00
Antidepressants - Other	28,529 (8.9%)	4,513 (7.8%)	0.04	3,922 (7.9%)	3,988 (8.0%)	0.00
Antidepressants - SSRI/SNRI	68,287 (21.4%)	11,824 (20.5%)	0.02	10,451 (21.1%)	10,306 (20.8%)	0.01
Antidepressants - Tricyclics	9,143 (2.9%)	1,599 (2.8%)	0.01	1,352 (2.7%)	1,339 (2.7%)	0.00
Antipsychotic agents	9,930 (3.1%)	1,722 (3.0%)	0.01	1,488 (3.0%)	1,485 (3.0%)	0.00
Anxiolytics (except benzodiazepine)	61,362 (19.2%)	9,962 (17.3%)	0.05	9,113 (18.4%)	9,038 (18.2%)	0.01
Anxiolytics - Benzodiazepines	4,203 (1.3%)	634 (1.1%)	0.02	552 (1.1%)	564 (1.1%)	0.00
Bronchodilators	63,927 (20.0%)	10,927 (18.9%)	0.03	9,462 (19.1%)	9,401 (18.9%)	0.01
Corticosteroids, inhaled	74,662 (23.4%)	12,974 (22.5%)	0.02	11,400 (23.0%)	11,155 (22.5%)	0.01
Corticosteroids, oral	107,785 (33.8%)	18,462 (32.0%)	0.04	15,977 (32.2%)	15,984 (32.2%)	0.00
Dementia drugs	17,535 (5.5%)	2,933 (5.1%)	0.02	2,481 (5.0%)	2,542 (5.1%)	0.00
Diabetes agents - Insulin	24,251 (7.6%)	4,148 (7.2%)	0.02	3,554 (7.2%)	3,621 (7.3%)	0.00
Diabetes agents - Metformin	50,093 (15.7%)	9,759 (16.9%)	-0.03	8,414 (16.9%)	8,384 (16.9%)	0.00
Diabetes agents - Other	17,971 (5.6%)	3,320 (5.8%)	-0.01	2,743 (5.5%)	2,802 (5.6%)	0.00
Diabetes agents - Sulfonylurea	29,179 (9.1%)	5,727 (9.9%)	-0.03	4,821 (9.7%)	4,822 (9.7%)	0.00
Estrogen	11,493 (3.6%)	1,966 (3.4%)	0.01	1,638 (3.3%)	1,667 (3.4%)	-0.01
GI - H2 blockers	26,524 (8.3%)	4,242 (7.3%)	0.04	3,730 (7.5%)	3,686 (7.4%)	0.00
GI - Proton-pump inhibitors	107,739 (33.7%)	18,273 (31.7%)	0.04	16,012 (32.3%)	15,794 (31.8%)	0.01
GI - Sucralfates	6,894 (2.2%)	1,157 (2.0%)	0.01	1,019 (2.1%)	998 (2.0%)	0.01
Hypnotics	27,741 (8.7%)	5,417 (9.4%)	-0.02	4,548 (9.2%)	4,529 (9.1%)	0.00
NSAIDs	53,631 (16.8%)	9,777 (16.9%)	0.00	8,310 (16.7%)	8,335 (16.8%)	0.00
Opioids	123,605 (38.7%)	22,391 (38.8%)	0.00	19,165 (38.6%)	19,221 (38.7%)	0.00
Parkinsonism drugs	12,931 (4.1%)	2,282 (4.0%)	0.01	1,979 (4.0%)	1,992 (4.0%)	0.00
Thyroid hormone replacement	74,532 (23.3%)	12,339 (21.4%)	0.05	10,730 (21.6%)	10,772 (21.7%)	0.00
**Health care utilization**						
ED Visit	131,669 (41.2%)	21,090 (36.5%)	0.10	18,334 (36.9%)	18,408 (37.1%)	0.00
Home health day[C]	7,486 (2.3%)	3,652 (6.3%)	-0.20	2,720 (5.5%)	2,738 (5.5%)	0.00
Home oxygen use	11,176 (3.5%)	1,635 (2.8%)	0.04	1,384 (2.8%)	1,418 (2.9%)	-0.01
Hospitalizations	171,248 (53.6%)	25,696 (44.5%)	0.18	21,679 (43.7%)	22,106 (44.5%)	-0.02
**Geographic Region**						
Northeast	61,891 (19.4%)	12,717 (22.0%)	-0.06	10,900 (22.0%)	10,860 (21.9%)	0.00
Midwest	68,129 (21.3%)	12,457 (21.6%)	-0.01	10,505 (21.2%)	10,725 (21.6%)	-0.01
South	137,838 (43.2%)	22,676 (39.3%)	0.08	19,851 (40.0%)	19,671 (39.6%)	0.01
West	51,176 (16.0%)	9,820 (17.0%)	-0.03	8,345 (16.8%)	8,343 (16.8%)	0.00
Other	222 (0.1%)	58 (0.1%)	0.00	47 (0.1%)	49 (0.1%)	0.00

Abbreviations: ACE, angiotensin converting enzyme; CCI, combined comorbidity; CFI, claims-based frailty index; CKD, chronic kidney disease; COPD, chronic obstructive pulmonary disease; GI, gastrointestinal; NSAID, non-steroidal anti-inflammatory drug; PVD, peripheral vascular disease; SNRI, serotonin-norepinephrine reuptake inhibitor; SSRI, selective serotonin reuptake inhibitor.

[A] Other race category includes Unknown, Other, Asian, Hispanic, North American Native, and Missing Race Categories in Medicare. Asian, Hispanic, Unknown, and Missing categories are included for race in Optum. Race is unavailable in the MarketScan database.

[B] With both Medicare are Medicaid enrollment eligibility

[C] Home Health services received (days)

© 2023 Lin KJ et al. *JAMA Network Open*.

eTable 10. Study Population With Atrial Fibrillation Treated With Dabigatran vs Apixaban Before and After 1:1 Propensity Score Matching in the Optum Population

Characteristics<br>Number of Patients (%)	Before PS Matching			After PS Matching		
	Apixaban (n=139,862)	Dabigatran (n=8,209)	St. Diff	Apixaban (n=7,023)	Dabigatran (n=7,023)	St. Diff
**Quarter/Year of Cohort Entry Date**						
Q1 2013	36 (0.0%)	650 (7.9%)	-0.41	36 (0.5%)	39 (0.6%)	-0.01
Q2 2013	192 (0.1%)	518 (6.3%)	-0.36	190 (2.7%)	228 (3.2%)	-0.03
Q3 2013	248 (0.2%)	463 (5.6%)	-0.33	245 (3.5%)	252 (3.6%)	-0.01
Q4 2013	434 (0.3%)	397 (4.8%)	-0.29	352 (5.0%)	330 (4.7%)	0.01
Q1 2014	669 (0.5%)	405 (4.9%)	-0.27	412 (5.9%)	398 (5.7%)	0.01
Q2 2014	820 (0.6%)	308 (3.8%)	-0.22	291 (4.1%)	308 (4.4%)	-0.01
Q3 2014	906 (0.6%)	243 (3.0%)	-0.18	229 (3.3%)	243 (3.5%)	-0.01
Q4 2014	1,066 (0.8%)	222 (2.7%)	-0.15	256 (3.6%)	222 (3.2%)	0.02
Q1 2015	1,418 (1.0%)	284 (3.5%)	-0.17	273 (3.9%)	284 (4.0%)	-0.01
Q2 2015	1,529 (1.1%)	212 (2.6%)	-0.11	208 (3.0%)	212 (3.0%)	0.00
Q3 2015	1,624 (1.2%)	192 (2.3%)	-0.08	176 (2.5%)	192 (2.7%)	-0.01
Q4 2015	1,713 (1.2%)	227 (2.8%)	-0.11	251 (3.6%)	227 (3.2%)	0.02
Q1 2016	2,625 (1.9%)	400 (4.9%)	-0.17	394 (5.6%)	400 (5.7%)	0.00
Q2 2016	2,364 (1.7%)	314 (3.8%)	-0.13	298 (4.2%)	314 (4.5%)	-0.01
Q3 2016	2,403 (1.7%)	287 (3.5%)	-0.11	303 (4.3%)	287 (4.1%)	0.01
Q4 2016	2,535 (1.8%)	276 (3.4%)	-0.10	285 (4.1%)	276 (3.9%)	0.01
Q1 2017	4,164 (3.0%)	299 (3.6%)	-0.03	323 (4.6%)	299 (4.3%)	0.01
Q2 2017	3,825 (2.7%)	238 (2.9%)	-0.01	244 (3.5%)	238 (3.4%)	0.01
Q3 2017	3,713 (2.7%)	203 (2.5%)	0.01	197 (2.8%)	203 (2.9%)	-0.01
Q4 2017	3,702 (2.6%)	204 (2.5%)	0.01	202 (2.9%)	204 (2.9%)	0.00
Q1 2018	6,971 (5.0%)	324 (3.9%)	0.05	333 (4.7%)	324 (4.6%)	0.00
Q2 2018	6,026 (4.3%)	215 (2.6%)	0.09	224 (3.2%)	215 (3.1%)	0.01
Q3 2018	5,624 (4.0%)	209 (2.5%)	0.08	214 (3.0%)	209 (3.0%)	0.00
Q4 2018	5,937 (4.2%)	174 (2.1%)	0.12	166 (2.4%)	174 (2.5%)	-0.01
Q1 2019	7,430 (5.3%)	184 (2.2%)	0.16	169 (2.4%)	184 (2.6%)	-0.01
Q2 2019	7,166 (5.1%)	125 (1.5%)	0.20	111 (1.6%)	125 (1.8%)	-0.02
Q3 2019	6,854 (4.9%)	134 (1.6%)	0.19	136 (1.9%)	134 (1.9%)	0.00
Q4 2019	7,031 (5.0%)	120 (1.5%)	0.20	128 (1.8%)	120 (1.7%)	0.01
Q1 2020	8,367 (6.0%)	63 (0.8%)	0.29	62 (0.9%)	63 (0.9%)	0.00
Q2 2020	6,496 (4.6%)	50 (0.6%)	0.25	51 (0.7%)	50 (0.7%)	0.00
Q3 2020	7,133 (5.1%)	31 (0.4%)	0.29	33 (0.5%)	31 (0.4%)	0.01
Q4 2020	7,317 (5.2%)	25 (0.3%)	0.30	21 (0.3%)	25 (0.4%)	-0.02
Q1 2021	12,561 (9.0%)	179 (2.2%)	0.30	184 (2.6%)	179 (2.5%)	0.01
Q2 2021	8,963 (6.4%)	34 (0.4%)	0.34	26 (0.4%)	34 (0.5%)	-0.01
**Sociodemographic**						
Mean age (SD)	77.16 (6.84)	75.75 (6.52)	0.21	75.84 (6.63)	75.91 (6.57)	-0.01
Male	69,859 (49.9%)	4,587 (55.9%)	-0.12	3,988 (56.8%)	3,904 (55.6%)	0.02
Female	70,003 (50.1%)	3,622 (44.1%)	0.12	3,035 (43.2%)	3,119 (44.4%)	-0.02
Black	12,930 (9.2%)	615 (7.5%)	0.06	513 (7.3%)	523 (7.4%)	0.00
White	104,831 (75.0%)	6,183 (75.3%)	-0.01	5,300 (75.5%)	5,280 (75.2%)	0.01
Other[A]	22,101 (15.8%)	1,411 (17.2%)	-0.04	1,210 (17.2%)	1,220 (17.4%)	-0.01
**Cardiovascular conditions**						
Acute myocardial infarction	10,055 (7.2%)	370 (4.5%)	0.12	322 (4.6%)	325 (4.6%)	0.00
Cardio-ablation	839 (0.6%)	61 (0.7%)	-0.01	52 (0.7%)	45 (0.6%)	0.01
Cardioversion	9,981 (7.1%)	564 (6.9%)	0.01	449 (6.4%)	463 (6.6%)	-0.01
Cerebrovascular disease	35,853 (25.6%)	1,940 (23.6%)	0.05	1,700 (24.2%)	1,671 (23.8%)	0.01
Congestive heart failure (inpatient)	22,692 (16.2%)	934 (11.4%)	0.14	770 (11.0%)	822 (11.7%)	-0.02
Congestive heart failure (outpatient)	51,015 (36.5%)	2,975 (36.2%)	0.01	2,530 (36.0%)	2,543 (36.2%)	0.00
Coronary revascularization	4,789 (3.4%)	176 (2.1%)	0.08	152 (2.2%)	154 (2.2%)	0.00
Hypertension	124,827 (89.3%)	7,314 (89.1%)	0.01	6,267 (89.2%)	6,248 (89.0%)	0.01
Ischemic Heart Disease	63,623 (45.5%)	3,587 (43.7%)	0.04	3,075 (43.8%)	3,053 (43.5%)	0.01
PVD or PVD Surgery	22,483 (16.1%)	1,110 (13.5%)	0.07	951 (13.5%)	968 (13.8%)	-0.01
Stroke (inpatient)	10,050 (7.2%)	477 (5.8%)	0.06	388 (5.5%)	417 (5.9%)	-0.02
Stroke (outpatient)	34,362 (24.6%)	1,795 (21.9%)	0.06	1,576 (22.4%)	1,555 (22.1%)	0.01
Syncope	16,382 (11.7%)	844 (10.3%)	0.04	706 (10.1%)	728 (10.4%)	-0.01

© 2023 Lin KJ et al. *JAMA Network Open*.

Characteristics<br>Number of Patients (%)	Before PS Matching			After PS Matching		
	Apixaban<br>(n=139,862)	Dabigatran<br>(n=8,209)	St. Diff	Apixaban<br>(n=7,023)	Dabigatran<br>(n=7,023)	St. Diff
Mean CHA_2DS_2-VASc score (SD)	4.78 (1.69)	4.49 (1.65)	0.17	4.50 (1.65)	4.51 (1.67)	-0.01
Mean HAS-BLED score (SD)	2.35 (0.74)	2.27 (0.69)	0.11	2.27 (0.70)	2.28 (0.69)	-0.01
**Non-cardiovascular conditions**						
Acute renal failure	22,153 (15.8%)	721 (8.8%)	0.21	647 (9.2%)	648 (9.2%)	0.00
Alcohol abuse or dependence	4,439 (3.2%)	212 (2.6%)	0.04	188 (2.7%)	192 (2.7%)	0.00
Anemia	36,923 (26.4%)	1,859 (22.6%)	0.09	1,579 (22.5%)	1,606 (22.9%)	-0.01
CKD: stage 3, 4 or unspecified	34,552 (24.7%)	1,462 (17.8%)	0.17	1,347 (19.2%)	1,316 (18.7%)	0.01
COPD	33,687 (24.1%)	1,733 (21.1%)	0.07	1,416 (20.2%)	1,501 (21.4%)	-0.03
Dementia	11,400 (8.2%)	536 (6.5%)	0.07	477 (6.8%)	480 (6.8%)	0.00
Diabetes	52,190 (37.3%)	3,080 (37.5%)	0.00	2,603 (37.1%)	2,622 (37.3%)	0.00
Endoscopy	2,212 (1.6%)	108 (1.3%)	0.03	88 (1.3%)	91 (1.3%)	0.00
Falls	19,561 (14.0%)	792 (9.6%)	0.14	717 (10.2%)	715 (10.2%)	0.00
Fractures	11,493 (8.2%)	607 (7.4%)	0.03	530 (7.5%)	533 (7.6%)	0.00
GI bleeding (inpatient)	3,643 (2.6%)	191 (2.3%)	0.02	155 (2.2%)	163 (2.3%)	-0.01
GI bleeding (outpatient)	31,041 (22.2%)	2,084 (25.4%)	-0.08	1,782 (25.4%)	1,743 (24.8%)	0.01
Liver disease	11,661 (8.3%)	532 (6.5%)	0.07	460 (6.5%)	472 (6.7%)	-0.01
Malignancy	22,697 (16.2%)	1,293 (15.8%)	0.01	1,059 (15.1%)	1,115 (15.9%)	-0.02
Obesity	33,654 (24.1%)	1,592 (19.4%)	0.11	1,361 (19.4%)	1,389 (19.8%)	-0.01
Peptic Ulcer	4,122 (2.9%)	145 (1.8%)	0.07	119 (1.7%)	136 (1.9%)	-0.02
Smoking	47,768 (34.2%)	1,843 (22.5%)	0.26	1,655 (23.6%)	1,674 (23.8%)	0.00
Mean CCI score (SD)	4.12 (3.14)	3.24 (2.73)	0.30	3.31 (2.77)	3.35 (2.77)	-0.01
Mean CFI (SD)	0.20 (0.07)	0.19 (0.06)	0.15	0.19 (0.06)	0.19 (0.06)	0.00
**Cardiovascular medications**						
ACE inhibitors	37,088 (26.5%)	2,250 (27.4%)	-0.02	1,884 (26.8%)	1,905 (27.1%)	-0.01
Angiotensin II receptor blockers	7,252 (5.2%)	430 (5.2%)	0.00	355 (5.1%)	377 (5.4%)	-0.01
Antiarrhythmic agents	25,010 (17.9%)	1,696 (20.7%)	-0.07	1,413 (20.1%)	1,439 (20.5%)	-0.01
Anticoagulants, injectable	673 (0.5%)	41 (0.5%)	0.00	34 (0.5%)	32 (0.5%)	0.00
Antiplatelet agent	20,124 (14.4%)	937 (11.4%)	0.09	830 (11.8%)	803 (11.4%)	0.01
Beta-blockers	93,575 (66.9%)	5,173 (63.0%)	0.08	4,403 (62.7%)	4,420 (62.9%)	0.00
Calcium channel blockers	58 (0.0%)	11 (0.1%)	-0.04	7 (0.1%)	8 (0.1%)	0.00
Diuretics	71,614 (51.2%)	4,090 (49.8%)	0.03	3,432 (48.9%)	3,480 (49.6%)	-0.01
Fibrates	5,057 (3.6%)	400 (4.9%)	-0.06	344 (4.9%)	326 (4.6%)	0.01
Nitrates	14,297 (10.2%)	780 (9.5%)	0.02	705 (10.0%)	659 (9.4%)	0.02
Statins	89,206 (63.8%)	4,961 (60.4%)	0.07	4,285 (61.0%)	4,257 (60.6%)	0.01
**Other medications**						
Anticonvulsants	24,458 (17.5%)	1,166 (14.2%)	0.09	1,039 (14.8%)	1,020 (14.5%)	0.01
Antidepressants - Other	13,358 (9.6%)	615 (7.5%)	0.08	532 (7.6%)	539 (7.7%)	0.00
Antidepressants - SSRI/SNRI	28,189 (20.2%)	1,498 (18.2%)	0.05	1,301 (18.5%)	1,300 (18.5%)	0.00
Antidepressants - Tricyclics	3,213 (2.3%)	173 (2.1%)	0.01	136 (1.9%)	141 (2.0%)	-0.01
Antipsychotic agents	3,714 (2.7%)	170 (2.1%)	0.04	136 (1.9%)	157 (2.2%)	-0.02
Anxiolytics (except benzodiazepine)	20,976 (15.0%)	1,246 (15.2%)	-0.01	1,113 (15.8%)	1,104 (15.7%)	0.00
Anxiolytics - Benzodiazepines	2,138 (1.5%)	79 (1.0%)	0.05	64 (0.9%)	68 (1.0%)	-0.01
Bronchodilators	28,108 (20.1%)	1,517 (18.5%)	0.04	1,301 (18.5%)	1,325 (18.9%)	-0.01
Corticosteroids, inhaled	27,841 (19.9%)	1,584 (19.3%)	0.02	1,361 (19.4%)	1,349 (19.2%)	0.01
Corticosteroids, oral	42,942 (30.7%)	2,369 (28.9%)	0.04	2,013 (28.7%)	2,031 (28.9%)	0.00
Dementia drugs	6,148 (4.4%)	354 (4.3%)	0.00	304 (4.3%)	310 (4.4%)	0.00
Diabetes agents - Insulin	10,129 (7.2%)	599 (7.3%)	0.00	520 (7.4%)	515 (7.3%)	0.00
Diabetes agents - Metformin	24,522 (17.5%)	1,475 (18.0%)	-0.01	1,241 (17.7%)	1,251 (17.8%)	0.00
Diabetes agents - Other	12,835 (9.2%)	787 (9.6%)	-0.01	658 (9.4%)	662 (9.4%)	0.00
Diabetes agents - Sulfonylurea	7,016 (5.0%)	406 (4.9%)	0.00	325 (4.6%)	350 (5.0%)	-0.02
Estrogen	3,161 (2.3%)	216 (2.6%)	-0.02	158 (2.2%)	173 (2.5%)	-0.02
GI - H2 blockers	10,635 (7.6%)	503 (6.1%)	0.06	452 (6.4%)	441 (6.3%)	0.00
GI - Proton-pump inhibitors	42,072 (30.1%)	2,228 (27.1%)	0.07	1,910 (27.2%)	1,905 (27.1%)	0.00
GI - Sucralfates	2,657 (1.9%)	118 (1.4%)	0.04	103 (1.5%)	98 (1.4%)	0.01
Hypnotics	8,606 (6.2%)	611 (7.4%)	-0.05	463 (6.6%)	487 (6.9%)	-0.01
NSAIDs	21,634 (15.5%)	1,191 (14.5%)	0.03	948 (13.5%)	979 (13.9%)	-0.01
Opioids	45,592 (32.6%)	2,844 (34.6%)	-0.04	2,376 (33.8%)	2,402 (34.2%)	-0.01
Parkinsonism drugs	4,903 (3.5%)	271 (3.3%)	0.01	245 (3.5%)	228 (3.2%)	0.02
Thyroid hormone replacement	29,189 (20.9%)	1,582 (19.3%)	0.04	1,291 (18.4%)	1,375 (19.6%)	-0.03
**Health care utilization**						

© 2023 Lin KJ et al. *JAMA Network Open*.

Characteristics<br>Number of Patients (%)	Before PS Matching			After PS Matching		
	Apixaban (n=139,862)	Dabigatran (n=8,209)	St. Diff	Apixaban (n=7,023)	Dabigatran (n=7,023)	St. Diff
ED Visit	77,695 (55.6%)	3,861 (47.0%)	0.17	3,311 (47.1%)	3,298 (47.0%)	0.00
Home oxygen use	6,659 (4.8%)	219 (2.7%)	0.11	188 (2.7%)	205 (2.9%)	-0.01
Hospitalizations	64,839 (46.4%)	2,957 (36.0%)	0.21	2,438 (34.7%)	2,505 (35.7%)	-0.02
**Geographic Region**						
Northeast	16,798 (12.0%)	1,319 (16.1%)	-0.12	1,116 (15.9%)	1,098 (15.6%)	0.01
Midwest	26,594 (19.0%)	1,354 (16.5%)	0.07	1,158 (16.5%)	1,158 (16.5%)	0.00
South	60,867 (43.5%)	3,213 (39.1%)	0.09	2,825 (40.2%)	2,788 (39.7%)	0.01
West	35,530 (25.4%)	2,316 (28.2%)	-0.06	1,915 (27.3%)	1,973 (28.1%)	-0.02
Other	73 (0.1%)	7 (0.1%)	0.00	9 (0.1%)	6 (0.1%)	0.00
**Business Type Code (Recategorized)**						
Commercial	8,763 (6.3%)	1,056 (12.9%)	-0.23	913 (13.0%)	865 (12.3%)	0.02
Medicare	131,099 (93.7%)	7,153 (87.1%)	0.23	6,110 (87.0%)	6,158 (87.7%)	-0.02

Abbreviations: ACE, angiotensin converting enzyme; CCI, combined comorbidity; CFI, claims-based frailty index; CKD, chronic kidney disease; COPD, chronic obstructive pulmonary disease; GI, gastrointestinal; NSAID, non-steroidal anti-inflammatory drug; PVD, peripheral vascular disease; SNRI, serotonin-norepinephrine reuptake inhibitor; SSRI, selective serotonin reuptake inhibitor.

[A] Other race category includes Unknown, Other, Asian, Hispanic, North American Native, and Missing Race Categories in Medicare. Asian, Hispanic, Unknown, and Missing categories are included for race in Optum. Race is unavailable in the MarketScan database.

© 2023 Lin KJ et al. *JAMA Network Open*.

eTable 11. Study Population With Atrial Fibrillation Treated With Dabigatran vs Apixaban Before and After 1:1 Propensity Score Matching in the MarketScan Population

Characteristics<br>Number of Patients (%)	Before PS Matching<br>Apixaban<br>(n=52,886)	 Dabigatran<br>(n=8.,314)	 St. Diff	After PS Matching<br>Apixaban<br>(n=6,688)	 Warfarin<br>(n=6,688)	 St. Diff
**Quarter/Year of Cohort Entry Date**						
Q1 2013	98 (0.2%)	1,102 (13.3%)	-0.54	98 (1.5%)	103 (1.5%)	0.00
Q2 2013	513 (1.0%)	909 (10.9%)	-0.43	500 (7.5%)	518 (7.7%)	-0.01
Q3 2013	762 (1.4%)	862 (10.4%)	-0.39	663 (9.9%)	663 (9.9%)	0.00
Q4 2013	1,109 (2.1%)	728 (8.8%)	-0.30	694 (10.4%)	691 (10.3%)	0.00
Q1 2014	1,037 (2.0%)	488 (5.9%)	-0.20	485 (7.3%)	486 (7.3%)	0.00
Q2 2014	1,327 (2.5%)	421 (5.1%)	-0.14	432 (6.5%)	421 (6.3%)	0.01
Q3 2014	1,404 (2.7%)	387 (4.7%)	-0.11	373 (5.6%)	387 (5.8%)	-0.01
Q4 2014	1,869 (3.5%)	303 (3.6%)	-0.01	284 (4.2%)	303 (4.5%)	-0.01
Q1 2015	1,763 (3.3%)	247 (3.0%)	0.02	260 (3.9%)	247 (3.7%)	0.01
Q2 2015	1,779 (3.4%)	186 (2.2%)	0.07	179 (2.7%)	186 (2.8%)	-0.01
Q3 2015	1,930 (3.6%)	195 (2.3%)	0.08	204 (3.1%)	195 (2.9%)	0.01
Q4 2015	2,059 (3.9%)	275 (3.3%)	0.03	267 (4.0%)	275 (4.1%)	-0.01
Q1 2016	2,437 (4.6%)	316 (3.8%)	0.04	337 (5.0%)	316 (4.7%)	0.01
Q2 2016	2,540 (4.8%)	327 (3.9%)	0.04	324 (4.8%)	327 (4.9%)	0.00
Q3 2016	2,681 (5.1%)	292 (3.5%)	0.08	302 (4.5%)	292 (4.4%)	0.00
Q4 2016	2,742 (5.2%)	277 (3.3%)	0.09	311 (4.7%)	277 (4.1%)	0.03
Q1 2017	1,933 (3.7%)	156 (1.9%)	0.11	159 (2.4%)	156 (2.3%)	0.01
Q2 2017	1,923 (3.6%)	145 (1.7%)	0.12	146 (2.2%)	145 (2.2%)	0.00
Q3 2017	1,956 (3.7%)	111 (1.3%)	0.15	106 (1.6%)	111 (1.7%)	-0.01
Q4 2017	2,167 (4.1%)	118 (1.4%)	0.17	122 (1.8%)	118 (1.8%)	0.00
Q1 2018	1,475 (2.8%)	92 (1.1%)	0.12	86 (1.3%)	92 (1.4%)	-0.01
Q2 2018	1,455 (2.8%)	72 (0.9%)	0.14	69 (1.0%)	72 (1.1%)	-0.01
Q3 2018	1,438 (2.7%)	43 (0.5%)	0.18	41 (0.6%)	43 (0.6%)	0.00
Q4 2018	1,463 (2.8%)	59 (0.7%)	0.16	59 (0.9%)	59 (0.9%)	0.00
Q1 2019	1,701 (3.2%)	35 (0.4%)	0.21	42 (0.6%)	35 (0.5%)	0.01
Q2 2019	1,924 (3.6%)	39 (0.5%)	0.22	38 (0.6%)	39 (0.6%)	0.00
Q3 2019	1,860 (3.5%)	45 (0.5%)	0.22	40 (0.6%)	45 (0.7%)	-0.01
Q4 2019	1,807 (3.4%)	24 (0.3%)	0.23	23 (0.3%)	24 (0.4%)	-0.02
Q1 2020	1,592 (3.0%)	17 (0.2%)	0.22	12 (0.2%)	17 (0.3%)	-0.02
Q2 2020	1,080 (2.0%)	11 (0.1%)	0.19	5 (0.1%)	11 (0.2%)	-0.03
Q3 2020	1,535 (2.9%)	19 (0.2%)	0.22	15 (0.2%)	19 (0.3%)	-0.02
Q4 2020	1,527 (2.9%)	13 (0.2%)	0.22	10 (0.1%)	13 (0.2%)	-0.03
**Sociodemographic**						
Mean Age (SD)	77.70 (7.81)	76.12 (7.30)	0.21	76.37 (7.50)	76.31 (7.35)	0.01
Male	27,829 (52.6%)	4,811 (57.9%)	-0.11	3,802 (56.9%)	3,836 (57.4%)	-0.01
Female	25,057 (47.4%)	3,503 (42.1%)	0.11	2,884 (43.1%)	2,850 (42.6%)	0.01
**Cardiovascular conditions**						
Acute Myocardial infarction	3,783 (7.2%)	368 (4.4%)	0.12	318 (4.8%)	303 (4.5%)	0.01
Cardio-ablation	294 (0.6%)	62 (0.7%)	-0.01	46 (0.7%)	51 (0.8%)	-0.01
Cardioversion	4,161 (7.9%)	619 (7.4%)	0.02	455 (6.8%)	473 (7.1%)	-0.01
Cerebrovascular disease	13,396 (25.3%)	1,905 (22.9%)	0.06	1,561 (23.3%)	1,547 (23.1%)	0.00
Congestive heart failure (inpatient)	10,031 (19.0%)	1,207 (14.5%)	0.12	937 (14.0%)	963 (14.4%)	-0.01
Congestive heart failure (outpatient)	14,041 (26.5%)	2,214 (26.6%)	0.00	1,818 (27.2%)	1,795 (26.8%)	0.01
Coronary revascularization	1,692 (3.2%)	167 (2.0%)	0.08	132 (2.0%)	141 (2.1%)	-0.01
Hypertension	45,480 (86.0%)	6,968 (83.8%)	0.06	5,646 (84.4%)	5,643 (84.4%)	0.00
Ischemic Heart Disease	23,849 (45.1%)	3,469 (41.7%)	0.07	2,889 (43.2%)	2,840 (42.5%)	0.01
PVD or PVD Surgery	6,821 (12.9%)	994 (12.0%)	0.03	833 (12.5%)	819 (12.2%)	0.01
Stroke (inpatient)	5,098 (9.6%)	594 (7.1%)	0.09	463 (6.9%)	491 (7.3%)	-0.02
Stroke (outpatient)	11,470 (21.7%)	1,542 (18.5%)	0.08	1,299 (19.4%)	1,264 (18.9%)	0.01
Syncope	6,199 (11.7%)	784 (9.4%)	0.07	669 (10.0%)	649 (9.7%)	0.01
Mean CHA_2DS_2-VASc score (SD)	3.53 (1.60)	3.72 (1.49)	-0.12	3.70 (1.53)	3.68 (1.52)	0.01
Mean HAS-BLED score (SD)	2.22 (0.70)	2.12 (0.66)	0.15	2.13 (0.66)	2.13 (0.66)	0.00
**Non-cardiovascular conditions**						
Acute renal failure	6,192 (11.7%)	546 (6.6%)	0.18	458 (6.9%)	466 (7.0%)	0.00
Alcohol abuse or dependence	934 (1.8%)	127 (1.5%)	0.02	99 (1.5%)	106 (1.6%)	-0.01
Anemia	11,090 (21.0%)	1,397 (16.8%)	0.11	1,147 (17.2%)	1,139 (17.0%)	0.01

© 2023 Lin KJ et al. *JAMA Network Open*.

Characteristics Number of Patients (%)	Before PS Matching			After PS Matching		
	Apixaban (n=52,886)	Dabigatran (n=8.,314)	St. Diff	Apixaban (n=6,688)	Warfarin (n=6,688)	St. Diff
CKD: stage 3, 4 or unspecified	8,150 (15.4%)	892 (10.7%)	0.14	752 (11.2%)	754 (11.3%)	0.00
COPD	10,581 (20.0%)	1,517 (18.2%)	0.05	1,216 (18.2%)	1,233 (18.4%)	-0.01
Dementia	3,342 (6.3%)	431 (5.2%)	0.05	354 (5.3%)	342 (5.1%)	0.01
Diabetes	16,761 (31.7%)	2,675 (32.2%)	-0.01	2,212 (33.1%)	2,154 (32.2%)	0.02
Endoscopy	659 (1.2%)	92 (1.1%)	0.01	72 (1.1%)	71 (1.1%)	0.00
Falls	4,199 (7.9%)	407 (4.9%)	0.12	382 (5.7%)	359 (5.4%)	0.01
Fractures	4,347 (8.2%)	548 (6.6%)	0.06	439 (6.6%)	465 (7.0%)	-0.02
GI bleeding (inpatient)	1,722 (3.3%)	261 (3.1%)	0.01	185 (2.8%)	205 (3.1%)	-0.02
GI bleeding (outpatient)	6,820 (12.9%)	1,187 (14.3%)	-0.04	946 (14.1%)	951 (14.2%)	0.00
Liver disease	3,217 (6.1%)	410 (4.9%)	0.05	330 (4.9%)	332 (5.0%)	0.00
Malignancy	9,717 (18.4%)	1,528 (18.4%)	0.00	1,243 (18.6%)	1,234 (18.5%)	0.00
Obesity	7,682 (14.5%)	1,007 (12.1%)	0.07	846 (12.7%)	836 (12.5%)	0.01
Peptic Ulcer	855 (1.6%)	66 (0.8%)	0.07	44 (0.7%)	58 (0.9%)	-0.02
Smoking	7,429 (14.0%)	866 (10.4%)	0.11	731 (10.9%)	738 (11.0%)	0.00
Mean CCI score (SD)	3.20 (2.71)	2.53 (2.40)	0.26	2.61 (2.40)	2.59 (2.45)	0.01
Mean CFI (SD)	0.19 (0.06)	0.19 (0.06)	0.00	0.19 (0.06)	0.19 (0.06)	0.00
**Cardiovascular medications**						
ACE inhibitors	13,406 (25.3%)	2,072 (24.9%)	0.01	1,682 (25.2%)	1,691 (25.3%)	0.00
Angiotensin II receptor blockers	3,331 (6.3%)	574 (6.9%)	-0.02	448 (6.7%)	466 (7.0%)	-0.01
Antiarrhythmic agents	9,692 (18.3%)	1,711 (20.6%)	-0.06	1,366 (20.4%)	1,378 (20.6%)	0.00
Anticoagulants, injectable	249 (0.5%)	70 (0.8%)	-0.04	49 (0.7%)	51 (0.8%)	-0.01
Antiplatelet agent	9,461 (17.9%)	1,154 (13.9%)	0.11	978 (14.6%)	960 (14.4%)	0.01
Beta-blockers	35,634 (67.4%)	5,263 (63.3%)	0.09	4,254 (63.6%)	4,261 (63.7%)	0.00
Calcium channel blockers	103 (0.2%)	14 (0.2%)	0.00	16 (0.2%)	11 (0.2%)	0.00
Diuretics	26,827 (50.7%)	4,085 (49.1%)	0.03	3,285 (49.1%)	3,300 (49.4%)	-0.01
Fibrates	2,278 (4.3%)	374 (4.5%)	-0.01	303 (4.5%)	318 (4.8%)	-0.01
Nitrates	33,919 (64.1%)	5,123 (61.6%)	0.05	4,178 (62.5%)	4,169 (62.4%)	0.00
Statins	5,925 (11.2%)	827 (9.9%)	0.04	657 (9.8%)	672 (10.1%)	-0.01
**Other medications**						
Anticonvulsants	7,654 (14.5%)	1,056 (12.7%)	0.05	867 (13.0%)	856 (12.8%)	0.01
Antidepressants - Other	3,917 (7.4%)	511 (6.1%)	0.05	427 (6.4%)	419 (6.3%)	0.00
Antidepressants - SSRI/SNRI	9,845 (18.6%)	1,443 (17.4%)	0.03	1,137 (17.0%)	1,167 (17.5%)	-0.01
Antidepressants - Tricyclics	1,210 (2.3%)	179 (2.2%)	0.01	151 (2.3%)	145 (2.2%)	0.01
Antipsychotic agents	1,215 (2.3%)	181 (2.2%)	0.01	147 (2.2%)	146 (2.2%)	0.00
Anxiolytics (except benzodiazepine)	8,262 (15.6%)	1,319 (15.9%)	-0.01	1,098 (16.4%)	1,089 (16.3%)	0.00
Anxiolytics - Benzodiazepines	601 (1.1%)	77 (0.9%)	0.02	67 (1.0%)	63 (0.9%)	0.01
Bronchodilators	10,248 (19.4%)	1,430 (17.2%)	0.06	1,148 (17.2%)	1,196 (17.9%)	-0.02
Corticosteroids, inhaled	11,849 (22.4%)	1,765 (21.2%)	0.03	1,405 (21.0%)	1,437 (21.5%)	-0.01
Corticosteroids, oral	16,997 (32.1%)	2,394 (28.8%)	0.07	1,926 (28.8%)	1,973 (29.5%)	-0.02
Dementia drugs	2,306 (4.4%)	386 (4.6%)	-0.01	309 (4.6%)	304 (4.5%)	0.00
Diabetes agents - Insulin	3,753 (7.1%)	591 (7.1%)	0.00	507 (7.6%)	481 (7.2%)	0.02
Diabetes agents - Metformin	8,209 (15.5%)	1,302 (15.7%)	-0.01	1,052 (15.7%)	1,054 (15.8%)	0.00
Diabetes agents - Other	3,116 (5.9%)	529 (6.4%)	-0.02	426 (6.4%)	428 (6.4%)	0.00
Diabetes agents - Sulfonylurea	4,391 (8.3%)	741 (8.9%)	-0.02	585 (8.7%)	587 (8.8%)	0.00
Estrogen	1,917 (3.6%)	297 (3.6%)	0.00	245 (3.7%)	239 (3.6%)	0.01
GI - H2 blockers	3,806 (7.2%)	457 (5.5%)	0.07	372 (5.6%)	377 (5.6%)	0.00
GI - Proton-pump inhibitors	16,184 (30.6%)	2,282 (27.4%)	0.07	1,865 (27.9%)	1,892 (28.3%)	-0.01
GI - Sucralfate	928 (1.8%)	130 (1.6%)	0.02	105 (1.6%)	110 (1.6%)	0.00
Hypnotics	3,902 (7.4%)	700 (8.4%)	-0.04	529 (7.9%)	556 (8.3%)	-0.01
NSAIDs	8,498 (16.1%)	1,246 (15.0%)	0.03	1,027 (15.4%)	1,019 (15.2%)	0.01
Opioids	18,890 (35.7%)	3,170 (38.1%)	-0.05	2,570 (38.4%)	2,599 (38.9%)	-0.01
Parkinsonism drugs	1,832 (3.5%)	272 (3.3%)	0.01	219 (3.3%)	229 (3.4%)	-0.01
Thyroid hormone replacement	11,265 (21.3%)	1,667 (20.1%)	0.03	1,339 (20.0%)	1,336 (20.0%)	0.00
**Health care utilization**						
Emergency department visits	29,076 (55.0%)	3,843 (46.2%)	0.18	3,099 (46.4%)	3,129 (46.8%)	-0.01
Hospitalizations	25,707 (48.6%)	3,295 (39.6%)	0.18	2,546 (38.1%)	2,628 (39.3%)	-0.02
**Geographic Region**						
Northeast	14,435 (27.3%)	2,292 (27.6%)	-0.01	1,817 (27.2%)	1,815 (27.1%)	0.00
Midwest	16,451 (31.1%)	2,208 (26.6%)	0.10	1,808 (27.0%)	1,817 (27.2%)	0.00
South	16,396 (31.0%)	2,514 (30.2%)	0.02	2,166 (32.4%)	2,121 (31.7%)	0.02

© 2023 Lin KJ et al. *JAMA Network Open*.

Characteristics Number of Patients (%)	Before PS Matching			After PS Matching		
	Apixaban (n=52,886)	Dabigatran (n=8.,314)	St. Diff	Apixaban (n=6,688)	Warfarin (n=6,688)	St. Diff
West	5,500 (10.4%)	1,271 (15.3%)	-0.15	870 (13.0%)	909 (13.6%)	-0.02
Other	104 (0.2%)	29 (0.3%)	-0.02	25 (0.4%)	24 (0.4%)	0.00
**Employee Classification**						
Salary Non-union	4,737 (9.0%)	856 (10.3%)	-0.04	665 (9.9%)	679 (10.2%)	-0.01
Salary Union	1,250 (2.4%)	170 (2.0%)	0.03	131 (2.0%)	148 (2.2%)	-0.01
Salary Other	925 (1.7%)	177 (2.1%)	-0.03	150 (2.2%)	146 (2.2%)	0.00
Hourly Non-Union	2,645 (5.0%)	461 (5.5%)	-0.02	361 (5.4%)	390 (5.8%)	-0.02
Hourly Union	12,594 (23.8%)	2,367 (28.5%)	-0.11	1,917 (28.7%)	1,914 (28.6%)	0.00
Hourly Other	249 (0.5%)	82 (1.0%)	-0.06	74 (1.1%)	66 (1.0%)	0.01
Non-Union	14,542 (27.5%)	1,645 (19.8%)	0.18	1,448 (21.7%)	1,398 (20.9%)	0.02
Union	791 (1.5%)	128 (1.5%)	0.00	117 (1.7%)	110 (1.6%)	0.01
Unknown	15,153 (28.7%)	2,428 (29.2%)	-0.01	1,823 (27.3%)	1,835 (27.4%)	0.00
**Employment Status**						
Active Full Time	3,651 (6.9%)	482 (5.8%)	0.05	430 (6.4%)	432 (6.5%)	0.00
Active Part Time or Seasonal	162 (0.3%)	15 (0.2%)	0.02	12 (0.2%)	14 (0.2%)	0.00
Early Retiree	561 (1.1%)	77 (0.9%)	0.02	81 (1.2%)	70 (1.0%)	0.02
Medicare Eligible Retiree	37,201 (70.3%)	5,735 (69.0%)	0.03	4,757 (71.1%)	4,678 (70.0%)	0.02
Retiree	513 (1.0%)	403 (4.8%)	-0.23	197 (2.9%)	204 (3.1%)	-0.01
COBRA Continuee	30 (0.1%)	4 (0.0%)	0.04	2 (0.0%)	4 (0.1%)	-0.04
Long Term Disability	44 (0.1%)	6 (0.1%)	0.00	8 (0.1%)	6 (0.1%)	0.00
Surviving Spouse/Dependent	4,855 (9.2%)	594 (7.1%)	0.08	476 (7.1%)	505 (7.6%)	-0.02
Other/Unknown/Missing	5,869 (11.1%)	998 (12.0%)	-0.03	723 (10.8%)	773 (11.6%)	-0.03
**Health Plan Indicator**						
Employer	46,349 (87.6%)	7,245 (87.1%)	0.02	5,898 (88.2%)	5,852 (87.5%)	0.02
Health Plan	6,537 (12.4%)	1,069 (12.9%)	-0.02	788 (11.8%)	834 (12.5%)	-0.02
**MHSA Coverage Indicator**						
Not Covered/Claims Not Present	2,904 (5.5%)	407 (4.9%)	0.03	294 (4.4%)	327 (4.9%)	-0.02
Covered/Possible MHSA Claims	43,883 (83.0%)	7,362 (88.5%)	-0.16	5,935 (88.8%)	5,904 (88.3%)	0.02
Missing	6,099 (11.5%)	545 (6.6%)	0.17	457 (6.8%)	455 (6.8%)	0.00
**Plan Indicator**						
Basic/major medical	0 (0.0%)	0 (0.0%)	--	0 (0.0%)	0 (0.0%)	--
Comprehensive	17,252 (32.6%)	3,238 (38.9%)	-0.13	2,610 (39.0%)	2,592 (38.8%)	0.00
EPO	121 (0.2%)	17 (0.2%)	0.00	14 (0.2%)	15 (0.2%)	0.00
HMO	5,609 (10.6%)	708 (8.5%)	0.07	513 (7.7%)	538 (8.0%)	-0.01
POS	1,564 (3.0%)	343 (4.1%)	-0.06	298 (4.5%)	288 (4.3%)	0.01
PPO	26,380 (49.9%)	3,765 (45.3%)	0.09	3,034 (45.4%)	3,036 (45.4%)	0.00
POS with capitation	698 (1.3%)	100 (1.2%)	0.01	105 (1.6%)	92 (1.4%)	0.02
CDHP	385 (0.7%)	42 (0.5%)	0.03	42 (0.6%)	38 (0.6%)	0.00
HDHP	242 (0.5%)	26 (0.3%)	0.03	19 (0.3%)	25 (0.4%)	-0.02
Missing	625 (1.2%)	581 (1.5%)	-0.03	261 (1.3%)	263 (1.3%)	0.00

Abbreviations: ACE, angiotensin converting enzyme; CCI, combined comorbidity; CFI, claims-based frailty index; CKD, chronic kidney disease; COPD, chronic obstructive pulmonary disease; GI, gastrointestinal; NSAID, non-steroidal anti-inflammatory drug; PVD, peripheral vascular disease; SNRI, serotonin-norepinephrine reuptake inhibitor; SSRI, selective serotonin reuptake inhibitor.

© 2023 Lin KJ et al. *JAMA Network Open*.

eTable 12. Study Population With Atrial Fibrillation Treated With Rivaroxaban vs Apixaban Before and After 1:1 Propensity Score Matching Pooled Across Medicare, Optum, and MarketScan Populations*

Characteristics Number of Patients (%)	Before PS Matching			After PS Matching		
	Apixaban (n=506,769)	Rivaroxaban (n=327,289)	St. Diff	Apixaban (n=265,877)	Rivaroxaban (n=265,877)	St. Diff
**Quarter/Year of Cohort Entry Date**						
Q1 2013	377 (0.1%)	12,682 (3.9%)	-0.28	377 (0.1%)	380 (0.1%)	0.00
Q2 2013	2,619 (0.5%)	13,176 (4.0%)	-0.24	2,619 (1.0%)	2,923 (1.1%)	-0.01
Q3 2013	3,800 (0.7%)	13,294 (4.1%)	-0.22	3,800 (1.4%)	4,070 (1.5%)	-0.01
Q4 2013	5,854 (1.2%)	13,951 (4.3%)	-0.19	5,854 (2.2%)	5,997 (2.3%)	0.00
Q1 2014	8,613 (1.7%)	17,063 (5.2%)	-0.19	8,605 (3.2%)	8,719 (3.3%)	0.00
Q2 2014	11,408 (2.3%)	17,611 (5.4%)	-0.16	11,312 (4.3%)	11,271 (4.2%)	0.00
Q3 2014	12,227 (2.4%)	15,978 (4.9%)	-0.13	11,896 (4.5%)	11,721 (4.4%)	0.00
Q4 2014	15,179 (3.0%)	15,046 (4.6%)	-0.08	13,536 (5.1%)	13,346 (5.0%)	0.00
Q1 2015	18,863 (3.7%)	15,271 (4.7%)	-0.05	14,822 (5.6%)	14,610 (5.5%)	0.00
Q2 2015	20,396 (4.0%)	14,506 (4.4%)	-0.02	14,455 (5.4%)	14,292 (5.4%)	0.00
Q3 2015	20,992 (4.1%)	12,974 (4.0%)	0.01	12,989 (4.9%)	12,900 (4.9%)	0.00
Q4 2015	23,477 (4.6%)	12,538 (3.8%)	0.04	12,648 (4.8%)	12,516 (4.7%)	0.00
Q1 2016	28,813 (5.7%)	14,466 (4.4%)	0.06	14,466 (5.4%)	14,463 (5.4%)	0.00
Q2 2016	29,461 (5.8%)	13,890 (4.2%)	0.07	13,793 (5.2%)	13,884 (5.2%)	0.00
Q3 2016	28,754 (5.7%)	13,170 (4.0%)	0.08	13,018 (4.9%)	13,169 (5.0%)	0.00
Q4 2016	30,747 (6.1%)	13,286 (4.1%)	0.09	13,207 (5.0%)	13,286 (5.0%)	0.00
Q1 2017	31,290 (6.2%)	17,490 (5.3%)	0.04	17,548 (6.6%)	17,474 (6.6%)	0.00
Q2 2017	30,992 (6.1%)	17,013 (5.2%)	0.04	17,140 (6.4%)	16,993 (6.4%)	0.00
Q3 2017	29,915 (5.9%)	15,919 (4.9%)	0.05	15,988 (6.0%)	15,910 (6.0%)	0.00
Q4 2017	31,333 (6.2%)	15,949 (4.9%)	0.06	15,939 (6.0%)	15,938 (6.0%)	0.00
Q1 2018	8,375 (1.7%)	3,115 (1.0%)	0.06	3,111 (1.2%)	3,115 (1.2%)	0.00
Q2 2018	7,386 (1.5%)	2,671 (0.8%)	0.06	2,688 (1.0%)	2,671 (1.0%)	0.00
Q3 2018	7,000 (1.4%)	2,445 (0.7%)	0.06	2,464 (0.9%)	2,445 (0.9%)	0.00
Q4 2018	7,340 (1.4%)	2,222 (0.7%)	0.08	2,228 (0.8%)	2,222 (0.8%)	0.00
Q1 2019	9,054 (1.8%)	2,548 (0.8%)	0.09	2,602 (1.0%)	2,548 (1.0%)	0.00
Q2 2019	8,996 (1.8%)	2,385 (0.7%)	0.09	2,386 (0.9%)	2,385 (0.9%)	0.00
Q3 2019	8,630 (1.7%)	2,202 (0.7%)	0.10	2,158 (0.8%)	2,201 (0.8%)	0.00
Q4 2019	8,735 (1.7%)	2,019 (0.6%)	0.10	2,010 (0.8%)	2,019 (0.8%)	0.00
Q1 2020	9,889 (2.0%)	2,398 (0.7%)	0.11	2,321 (0.9%)	2,398 (0.9%)	0.00
Q2 2020	7,498 (1.5%)	1,698 (0.5%)	0.10	1,626 (0.6%)	1,698 (0.6%)	0.00
Q3 2020	8,582 (1.7%)	1,708 (0.5%)	0.11	1,701 (0.6%)	1,708 (0.6%)	0.00
Q4 2020	8,771 (1.7%)	1,730 (0.5%)	0.11	1,668 (0.6%)	1,730 (0.7%)	0.00
Q1 2021	12,518 (2.5%)	3,057 (0.9%)	0.12	3,128 (1.2%)	3,057 (1.1%)	0.00
Q2 2021	8,885 (1.8%)	1,818 (0.6%)	0.11	1,774 (0.7%)	1,818 (0.7%)	0.00
**Sociodemographic**						
Mean Age (SD)	77.80 (7.35)	76.72 (7.14)	0.15	76.82 (7.2)	76.89 (7.17)	-0.01
Male	242,456 (47.8%)	165,031 (50.4%)	-0.05	133,454 (50.2%)	133,557 (50.2%)	0.00
Female	264,313 (52.2%)	162,258 (49.6%)	0.05	132,423 (49.8%)	132,320 (49.8%)	0.00
Black	25,795 (5.7%)	14,073 (4.8%)	0.04	11,853 (4.9%)	11,828 (4.9%)	0.00
White	392,673 (86.4%)	256,809 (87.8%)	-0.04	210,296 (87.5%)	210,296 (87.5%)	0.00
Other[A]	35,995 (7.9%)	21,593 (7.4%)	0.02	18,059 (7.5%)	18,084 (7.5%)	0.00
Dual Status[B]	17,556 (5.6%)	13,568 (5.7%)	0.00	10,704 (5.6%)	10,882 (5.6%)	0.00
**Cardiovascular conditions**						
Acute Myocardial infarction	34,877 (6.9%)	17,225 (5.3%)	0.07	14,427 (5.4%)	14,572 (5.5%)	0.00
Cardio-ablation	3,520 (0.7%)	2,205 (0.7%)	0.00	1,699 (0.6%)	1,708 (0.6%)	0.00
Cardioversion	37,602 (7.4%)	21,099 (6.4%)	0.04	17,691 (6.7%)	17,755 (6.7%)	0.00
Cerebrovascular disease	136,840 (27.0%)	80,199 (24.5%)	0.06	64,393 (24.2%)	65,070 (24.5%)	-0.01
Congestive heart failure (inpatient)	99,312 (19.6%)	54,731 (16.7%)	0.07	44,516 (16.7%)	45,065 (16.9%)	-0.01
Congestive heart failure (outpatient)	161,328 (31.8%)	95,928 (29.3%)	0.05	77,249 (29.1%)	77,540 (29.2%)	0.00
Coronary revascularization	17,222 (3.4%)	8,933 (2.7%)	0.04	7,433 (2.8%)	7,461 (2.8%)	0.00
Hypertension	441,288 (87.1%)	277,941 (84.9%)	0.06	226,481 (85.2%)	226,620 (85.2%)	0.00
Ischemic Heart Disease	232,445 (45.9%)	139,187 (42.5%)	0.07	113,118 (42.5%)	113,388 (42.6%)	0.00
PVD or PVD Surgery	72,990 (14.4%)	43,384 (13.3%)	0.03	35,243 (13.3%)	35,389 (13.3%)	0.00
Stroke (inpatient)	45,174 (8.9%)	22,347 (6.8%)	0.08	18,282 (6.9%)	18,749 (7.1%)	-0.01

Characteristics Number of Patients (%)	Before PS Matching			After PS Matching		
	Apixaban (n=506,769)	Rivaroxaban (n=327,289)	St. Diff	Apixaban (n=265,877)	Rivaroxaban (n=265,877)	St. Diff
Stroke (outpatient)	74,549 (14.7%)	34,714 (10.6%)	0.12	29,254 (11.0%)	29,610 (11.1%)	0.00
Syncope	58,369 (11.5%)	33,544 (10.2%)	0.04	27,595 (10.4%)	27,617 (10.4%)	0.00
Mean CHA2DS2-VASc score (SD)	4.63 (1.70)	4.41 (1.68)	0.13	4.42 (1.7)	4.43 (1.69)	0.00
Mean HAS-BLED score (SD)	2.31 (0.74)	2.22 (0.70)	0.13	2.23 (0.7)	2.23 (0.71)	0.00
**Non-cardiovascular conditions**						
Acute renal failure	72,797 (14.4%)	33,052 (10.1%)	0.13	28,373 (10.7%)	28,822 (10.8%)	-0.01
Alcohol abuse or dependence	11,431 (2.3%)	6,320 (1.9%)	0.02	5,642 (2.1%)	5,635 (2.1%)	0.00
Anemia	136,273 (26.9%)	79,771 (24.4%)	0.06	64,976 (24.4%)	65,428 (24.6%)	0.00
CKD: stage 3, 4 or unspecified	94,706 (18.7%)	43,101 (13.2%)	0.15	37,044 (13.9%)	37,598 (14.1%)	-0.01
COPD	114,991 (22.7%)	69,564 (21.3%)	0.03	56,494 (21.2%)	56,712 (21.3%)	0.00
Dementia	42,038 (8.3%)	24,570 (7.5%)	0.03	20,073 (7.5%)	20,229 (7.6%)	0.00
Diabetes	181,205 (35.8%)	114,254 (34.9%)	0.02	93,275 (35.1%)	93,060 (35.0%)	0.00
Endoscopy	9,625 (1.9%)	5,529 (1.7%)	0.02	4,486 (1.7%)	4,505 (1.7%)	0.00
Falls	38,177 (7.5%)	16,806 (5.1%)	0.10	14,569 (5.5%)	14,772 (5.6%)	0.00
Fractures	43,994 (8.7%)	27,689 (8.5%)	0.01	22,484 (8.5%)	22,651 (8.5%)	0.00
GI bleeding (inpatient)	23,730 (4.7%)	14,882 (4.5%)	0.01	11,913 (4.5%)	11,940 (4.5%)	0.00
GI bleeding (outpatient)	69,985 (13.8%)	41,550 (12.7%)	0.03	33,615 (12.6%)	33,578 (12.6%)	0.00
Liver disease	34,200 (6.7%)	19,751 (6.0%)	0.03	16,377 (6.2%)	16,460 (6.2%)	0.00
Malignancy	90,440 (17.8%)	58,268 (17.8%)	0.00	46,769 (17.6%)	47,014 (17.7%)	0.00
Obesity	103,580 (20.4%)	63,631 (19.4%)	0.02	53,565 (20.1%)	53,387 (20.1%)	0.00
Peptic Ulcer	12,593 (2.5%)	6,191 (1.9%)	0.04	5,214 (2.0%)	5,312 (2.0%)	0.00
Smoking	165,305 (32.6%)	95,382 (29.1%)	0.08	81,186 (30.5%)	81,537 (30.7%)	0.00
Mean CCI score (SD)	3.52 (2.88)	3.02 (2.66)	0.18	3.08 (2.7)	3.11 (2.71)	-0.01
Mean CFI (SD)	0.21 (0.07)	0.20 (0.07)	0.12	0.20 (0.1)	0.20 (0.07)	0.00
**Cardiovascular medications**						
ACE inhibitors	136,779 (27.0%)	87,767 (26.8%)	0.00	71,131 (26.8%)	71,166 (26.8%)	0.00
Angiotensin II receptor blockers	30,577 (6.0%)	20,221 (6.2%)	-0.01	15,891 (6.0%)	15,985 (6.0%)	0.00
Antiarrhythmic agents	102,700 (20.3%)	65,015 (19.9%)	0.01	51,877 (19.5%)	51,923 (19.5%)	0.00
Anticoagulants, injectable	2,820 (0.6%)	2,482 (0.8%)	-0.02	1,857 (0.7%)	1,836 (0.7%)	0.00
Antiplatelet agent	83,020 (16.4%)	48,713 (14.9%)	0.04	39,282 (14.8%)	39,437 (14.8%)	0.00
Beta-blockers	345,100 (68.1%)	214,811 (65.6%)	0.05	175,740 (66.1%)	175,911 (66.2%)	0.00
Calcium channel blockers	7,580 (1.5%)	5,409 (1.7%)	-0.01	4,294 (1.6%)	4,273 (1.6%)	0.00
Diuretics	271,620 (53.6%)	169,748 (51.9%)	0.03	138,451 (52.1%)	138,452 (52.1%)	0.00
Fibrates	21,080 (4.2%)	14,451 (4.4%)	-0.01	11,445 (4.3%)	11,527 (4.3%)	0.00
Nitrates	60,947 (12.0%)	36,053 (11.0%)	0.03	28,907 (10.9%)	29,109 (10.9%)	0.00
Statins	329,975 (65.1%)	205,337 (62.7%)	0.05	168,050 (63.2%)	168,276 (63.3%)	0.00
**Other medications**						
Anticonvulsants	85,367 (16.8%)	50,311 (15.4%)	0.04	42,098 (15.8%)	42,094 (15.8%)	0.00
Antidepressants - Other	45,279 (8.9%)	26,415 (8.1%)	0.03	22,214 (8.4%)	22,242 (8.4%)	0.00
Antidepressants - SSRI/SNRI	105,226 (20.8%)	65,096 (19.9%)	0.02	53,296 (20.0%)	53,510 (20.1%)	0.00
Antidepressants - Tricyclics	13,405 (2.6%)	8,801 (2.7%)	0.00	6,968 (2.6%)	6,952 (2.6%)	0.00
Antipsychotic agents	14,718 (2.9%)	9,941 (3.0%)	-0.01	7,930 (3.0%)	8,012 (3.0%)	0.00
Anxiolytics (except benzodiazepine)	6,853 (1.4%)	3,909 (1.2%)	0.01	3,400 (1.3%)	3,295 (1.2%)	0.00
Anxiolytics - Benzodiazepines	89,478 (17.7%)	57,943 (17.7%)	0.00	47,574 (17.9%)	47,560 (17.9%)	0.00
Bronchodilators	101,141 (20.0%)	63,740 (19.5%)	0.01	51,838 (19.5%)	51,917 (19.5%)	0.00
Corticosteroids, inhaled	113,125 (22.3%)	73,271 (22.4%)	0.00	59,287 (22.3%)	59,412 (22.3%)	0.00
Corticosteroids, oral	165,903 (32.7%)	104,350 (31.9%)	0.02	85,226 (32.1%)	85,193 (32.0%)	0.00
Dementia drugs	25,785 (5.1%)	16,199 (4.9%)	0.01	13,082 (4.9%)	13,218 (5.0%)	0.00
Diabetes agents - Insulin	37,780 (7.5%)	21,964 (6.7%)	0.03	18,217 (6.9%)	18,254 (6.9%)	0.00
Diabetes agents - Metformin	82,067 (16.2%)	53,181 (16.2%)	0.00	43,851 (16.5%)	43,459 (16.3%)	0.00
Diabetes agents - Other	27,851 (5.5%)	17,730 (5.4%)	0.00	14,327 (5.4%)	14,335 (5.4%)	0.00
Diabetes agents - Sulfonylurea	46,009 (9.1%)	29,291 (8.9%)	0.00	23,533 (8.9%)	23,584 (8.9%)	0.00
Estrogen	16,394 (3.2%)	11,051 (3.4%)	-0.01	8,536 (3.2%)	8,580 (3.2%)	0.00
GI - H2 blockers	40,547 (8.0%)	23,702 (7.2%)	0.03	19,636 (7.4%)	19,827 (7.5%)	0.00
GI - Proton-pump inhibitors	164,072 (32.4%)	101,566 (31.0%)	0.03	82,760 (31.1%)	83,026 (31.2%)	0.00
GI - Sucralfate	10,298 (2.0%)	6,111 (1.9%)	0.01	4,954 (1.9%)	5,027 (1.9%)	0.00
Hypnotics	39,717 (7.8%)	28,672 (8.8%)	-0.03	22,182 (8.3%)	22,039 (8.3%)	0.00
NSAIDs	82,927 (16.4%)	57,507 (17.6%)	-0.03	45,816 (17.2%)	45,815 (17.2%)	0.00
Opioids	185,957 (36.7%)	124,375 (38.0%)	-0.03	99,953 (37.6%)	99,554 (37.4%)	0.00
Parkinsonism drugs	19,445 (3.8%)	12,064 (3.7%)	0.01	9,921 (3.7%)	9,900 (3.7%)	0.00

© 2023 Lin KJ et al. *JAMA Network Open*.

Characteristics<br>Number of Patients (%)	Before PS Matching			After PS Matching		
	Apixaban (n=506,769)	Rivaroxaban (n=327,289)	St. Diff	Apixaban (n=265,877)	Rivaroxaban (n=265,877)	St. Diff
Thyroid hormone replacement	113,691 (22.4%)	69,019 (21.1%)	0.03	56,666 (21.3%)	56,783 (21.4%)	0.00
**Health care utilization**						
Emergency department visits	235,890 (46.5%)	137,698 (42.1%)	0.09	113,434 (42.7%)	113,964 (42.9%)	0.00
Home Health Day[C]	7,390 (2.3%)	12,618 (5.3%)	-0.15	6,829 (3.5%)	6,756 (3.5%)	0.00
Home oxygen use	18,434 (3.6%)	9,689 (3.0%)	0.04	7,947 (3.0%)	8,029 (3.0%)	0.00
Hospitalizations	259,313 (51.2%)	151,801 (46.4%)	0.10	122,468 (46.1%)	123,353 (46.4%)	-0.01
**Geographic Region**						
Northeast	92,310 (18.2%)	62,727 (19.2%)	-0.02	50,711 (19.1%)	50,882 (19.1%)	0.00
Midwest	110,221 (21.7%)	74,046 (22.6%)	-0.02	60,053 (22.6%)	60,070 (22.6%)	0.00
South	212,792 (42.0%)	126,499 (38.7%)	0.07	103,250 (38.8%)	103,420 (38.9%)	0.00
West	91,052 (18.0%)	63,567 (19.4%)	-0.04	51,567 (19.4%)	51,227 (19.3%)	0.00
Other	394 (0.1%)	450 (0.1%)	-0.02	296 (0.1%)	278 (0.1%)	0.00

Abbreviations: ACE, angiotensin converting enzyme; CCI, combined comorbidity; CFI, claims-based frailty index; CKD, chronic kidney disease; COPD, chronic obstructive pulmonary disease; GI, gastrointestinal; NSAID, non-steroidal anti-inflammatory drug; PVD, peripheral vascular disease; SNRI, serotonin-norepinephrine reuptake inhibitor; SSRI, selective serotonin reuptake inhibitor.

[A] Other race category includes Unknown, Other, Asian, Hispanic, North American Native, and Missing Race Categories in Medicare. Asian, Hispanic, Unknown, and Missing categories are included for race in Optum. Race is unavailable in the MarketScan database.

[B] With both Medicare are Medicaid enrollment eligibility

[C] Home Health services received (days)

© 2023 Lin KJ et al. *JAMA Network Open*.

eTable 13. Study Population With Atrial Fibrillation Treated With Rivaroxaban vs Apixaban Before and After 1:1 Propensity Score Matching in the Medicare Population

Characteristics Number of Patients (%)	Before PS Matching			After PS Matching		
	Apixaban (n=315,660)	Rivaroxaban (n=239,600)	St. Diff	Apixaban (n=192,640)	Rivaroxaban (n=192,640)	St. Diff
**Quarter/Year of Cohort Entry Date**						
Q1 2013	243 (0.1%)	9,618 (4.0%)	-0.28	243 (0.1%)	243 (0.1%)	0.00
Q2 2013	1,914 (0.6%)	9,979 (4.2%)	-0.24	1,914 (1.0%)	2,086 (1.1%)	-0.01
Q3 2013	2,791 (0.9%)	9,992 (4.2%)	-0.21	2,791 (1.4%)	2,961 (1.5%)	-0.01
Q4 2013	4,310 (1.4%)	10,466 (4.4%)	-0.18	4,310 (2.2%)	4,427 (2.3%)	-0.01
Q1 2014	6,903 (2.2%)	13,627 (5.7%)	-0.18	6,897 (3.6%)	7,043 (3.7%)	-0.01
Q2 2014	9,266 (2.9%)	14,149 (5.9%)	-0.15	9,187 (4.8%)	9,137 (4.7%)	0.00
Q3 2014	9,921 (3.1%)	12,736 (5.3%)	-0.11	9,643 (5.0%)	9,548 (5.0%)	0.00
Q4 2014	12,264 (3.9%)	11,881 (5.0%)	-0.05	10,882 (5.6%)	10,739 (5.6%)	0.00
Q1 2015	15,702 (5.0%)	12,664 (5.3%)	-0.01	12,351 (6.4%)	12,179 (6.3%)	0.00
Q2 2015	17,114 (5.4%)	12,053 (5.0%)	0.02	12,050 (6.3%)	11,933 (6.2%)	0.00
Q3 2015	17,472 (5.5%)	10,646 (4.4%)	0.05	10,690 (5.5%)	10,615 (5.5%)	0.00
Q4 2015	19,749 (6.3%)	10,379 (4.3%)	0.09	10,465 (5.4%)	10,376 (5.4%)	0.00
Q1 2016	23,803 (7.5%)	11,893 (5.0%)	0.10	11,897 (6.2%)	11,892 (6.2%)	0.00
Q2 2016	24,599 (7.8%)	11,442 (4.8%)	0.12	11,374 (5.9%)	11,441 (5.9%)	0.00
Q3 2016	23,715 (7.5%)	10,827 (4.5%)	0.13	10,614 (5.5%)	10,826 (5.6%)	0.00
Q4 2016	25,525 (8.1%)	11,106 (4.6%)	0.14	10,984 (5.7%)	11,106 (5.8%)	0.00
Q1 2017	25,260 (8.0%)	14,678 (6.1%)	0.07	14,750 (7.7%)	14,662 (7.6%)	0.00
Q2 2017	25,317 (8.0%)	14,520 (6.1%)	0.07	14,620 (7.6%)	14,500 (7.5%)	0.00
Q3 2017	24,279 (7.7%)	13,429 (5.6%)	0.08	13,447 (7.0%)	13,422 (7.0%)	0.00
Q4 2017	25,513 (8.1%)	13,515 (5.6%)	0.10	13,531 (7.0%)	13,504 (7.0%)	0.00
**Sociodemographic**						
Mean age (SD)	78.10 (7.49)	76.92 (7.20)	0.16	77.02 (7.26)	77.09 (7.24)	-0.01
Male	145,655 (46.1%)	117,046 (48.9%)	-0.06	93,764 (48.7%)	93,751 (48.7%)	0.00
Female	170,005 (53.9%)	122,554 (51.1%)	0.06	98,876 (51.3%)	98,889 (51.3%)	0.00
Black	12,956 (4.1%)	9,846 (4.1%)	0.00	8,010 (4.2%)	7,966 (4.1%)	0.01
White	288,647 (91.4%)	217,037 (90.6%)	0.03	174,589 (90.6%)	174,691 (90.7%)	0.00
Other[A]	14,057 (4.5%)	12,717 (5.3%)	-0.04	10,041 (5.2%)	9,983 (5.2%)	0.00
Dual Status[B]	17,556 (5.6%)	13,568 (5.7%)	0.00	10,704 (5.6%)	10,882 (5.6%)	0.00
**Cardiovascular conditions**						
Acute myocardial infarction	21,142 (6.7%)	12,624 (5.3%)	0.06	10,538 (5.5%)	10,625 (5.5%)	0.00
Cardio-ablation	2,399 (0.8%)	1,721 (0.7%)	0.01	1,302 (0.7%)	1,329 (0.7%)	0.00
Cardioversion	23,598 (7.5%)	14,818 (6.2%)	0.05	12,558 (6.5%)	12,603 (6.5%)	0.00
Cerebrovascular disease	88,067 (27.9%)	61,230 (25.6%)	0.05	48,549 (25.2%)	48,982 (25.4%)	0.00
Congestive heart failure (inpatient)	66,903 (21.2%)	42,760 (17.8%)	0.09	34,669 (18.0%)	35,070 (18.2%)	-0.01
Congestive heart failure (outpatient)	96,886 (30.7%)	69,552 (29.0%)	0.04	55,131 (28.6%)	55,267 (28.7%)	0.00
Coronary revascularization	10,779 (3.4%)	6,806 (2.8%)	0.03	5,618 (2.9%)	5,651 (2.9%)	0.00
Hypertension	272,471 (86.3%)	202,723 (84.6%)	0.05	163,096 (84.7%)	163,252 (84.7%)	0.00
Ischemic Heart Disease	145,751 (46.2%)	103,013 (43.0%)	0.06	82,770 (43.0%)	82,982 (43.1%)	0.00
PVD or PVD Surgery	43,995 (13.9%)	31,455 (13.1%)	0.02	25,147 (13.1%)	25,152 (13.1%)	0.00
Stroke (inpatient)	30,180 (9.6%)	17,315 (7.2%)	0.09	14,102 (7.3%)	14,505 (7.5%)	-0.01
Stroke (outpatient)	29,162 (9.2%)	17,801 (7.4%)	0.07	14,874 (7.7%)	15,037 (7.8%)	0.00
Syncope	35,991 (11.4%)	24,599 (10.3%)	0.04	20,050 (10.4%)	20,019 (10.4%)	0.00
Mean CHA_2DS_2-VASc score (SD)	4.74 (1.72)	4.52 (1.71)	0.13	4.53 (1.70)	4.54 (1.71)	-0.01
Mean HAS-BLED score (SD)	2.31 (0.74)	2.23 (0.71)	0.11	2.24 (0.72)	2.24 (0.72)	0.00
**Non-cardiovascular conditions**						
Acute renal failure	44,681 (14.2%)	24,954 (10.4%)	0.12	21,268 (11.0%)	21,547 (11.2%)	-0.01
Alcohol abuse or dependence	6,112 (1.9%)	4,199 (1.8%)	0.01	3,778 (2.0%)	3,762 (2.0%)	0.00
Anemia	88,726 (28.1%)	61,827 (25.8%)	0.05	49,578 (25.7%)	49,876 (25.9%)	0.00
CKD: stage 3, 4 or unspecified	52,383 (16.6%)	29,060 (12.1%)	0.13	24,427 (12.7%)	24,939 (12.9%)	-0.01
COPD	71,132 (22.5%)	51,501 (21.5%)	0.02	41,207 (21.4%)	41,389 (21.5%)	0.00
Dementia	27,394 (8.7%)	19,149 (8.0%)	0.03	15,353 (8.0%)	15,457 (8.0%)	0.00
Diabetes	112,815 (35.7%)	84,760 (35.4%)	0.01	68,131 (35.4%)	68,000 (35.3%)	0.00
Endoscopy	6,800 (2.2%)	4,532 (1.9%)	0.02	3,648 (1.9%)	3,670 (1.9%)	0.00
Falls	14,634 (4.6%)	9,106 (3.8%)	0.04	7,584 (3.9%)	7,741 (4.0%)	-0.01
Fractures	28,300 (9.0%)	20,885 (8.7%)	0.01	16,721 (8.7%)	16,879 (8.8%)	0.00
GI bleeding (inpatient)	18,453 (5.8%)	12,678 (5.3%)	0.02	10,173 (5.3%)	10,170 (5.3%)	0.00
GI bleeding (outpatient)	32,567 (10.3%)	25,090 (10.5%)	-0.01	19,934 (10.3%)	19,850 (10.3%)	0.00
Liver disease	19,467 (6.2%)	14,351 (6.0%)	0.01	11,675 (6.1%)	11,720 (6.1%)	0.00
Malignancy	58,319 (18.5%)	43,655 (18.2%)	0.01	34,821 (18.1%)	35,046 (18.2%)	0.00
Obesity	62,572 (19.8%)	47,415 (19.8%)	0.00	39,121 (20.3%)	38,925 (20.2%)	0.00
Peptic Ulcer	7,663 (2.4%)	4,807 (2.0%)	0.03	4,008 (2.1%)	4,036 (2.1%)	0.00

© 2023 Lin KJ et al. *JAMA Network Open*.

Characteristics<br>Number of Patients (%)	Before PS Matching			After PS Matching		
	Apixaban<br>(n=315,660)	Rivaroxaban<br>(n=239,600)	St. Diff	Apixaban<br>(n=192,640)	Rivaroxaban<br>(n=192,640)	St. Diff
Smoking	110,595 (35.0%)	76,644 (32.0%)	0.06	64,190 (33.3%)	64,408 (33.4%)	0.00
Mean CCI score (SD)	3.31 (2.78)	2.97 (2.64)	0.13	3.01 (2.65)	3.04 (2.68)	-0.01
Mean CFI (SD)	0.21 (0.07)	0.20 (0.07)	0.14	0.20 (0.07)	0.20 (0.07)	0.00
**Cardiovascular medications**						
ACE inhibitors	86,682 (27.5%)	65,124 (27.2%)	0.01	52,288 (27.1%)	52,281 (27.1%)	0.00
Angiotensin II receptor blockers	20,079 (6.4%)	15,166 (6.3%)	0.00	11,810 (6.1%)	11,874 (6.2%)	0.00
Antiarrhythmic agents	68,450 (21.7%)	49,254 (20.6%)	0.03	39,072 (20.3%)	39,177 (20.3%)	0.00
Anticoagulants, injectable	1,912 (0.6%)	1,978 (0.8%)	-0.02	1,458 (0.8%)	1,443 (0.7%)	0.01
Antiplatelet agent	53,737 (17.0%)	36,533 (15.2%)	0.05	29,102 (15.1%)	29,400 (15.3%)	-0.01
Beta-blockers	217,000 (68.7%)	158,421 (66.1%)	0.06	128,488 (66.7%)	128,484 (66.7%)	0.00
Calcium channel blockers	7,421 (2.4%)	5,303 (2.2%)	0.01	4,213 (2.2%)	4,186 (2.2%)	0.00
Diuretics	173,973 (55.1%)	126,993 (53.0%)	0.04	102,463 (53.2%)	102,529 (53.2%)	0.00
Fibrates	13,789 (4.4%)	10,732 (4.5%)	0.00	8,427 (4.4%)	8,518 (4.4%)	0.00
Nitrates	40,904 (13.0%)	27,984 (11.7%)	0.04	22,261 (11.6%)	22,379 (11.6%)	0.00
Statins	207,860 (65.8%)	152,075 (63.5%)	0.05	123,224 (64.0%)	123,367 (64.0%)	0.00
**Other medications**						
Anticonvulsants	53,523 (17.0%)	38,043 (15.9%)	0.03	31,312 (16.3%)	31,368 (16.3%)	0.00
Antidepressants - Other	28,167 (8.9%)	19,764 (8.2%)	0.03	16,350 (8.5%)	16,372 (8.5%)	0.00
Antidepressants - SSRI/SNRI	67,487 (21.4%)	49,737 (20.8%)	0.01	40,111 (20.8%)	40,324 (20.9%)	0.00
Antidepressants - Tricyclics	9,024 (2.9%)	6,846 (2.9%)	0.00	5,384 (2.8%)	5,393 (2.8%)	0.00
Antipsychotic agents	9,837 (3.1%)	8,043 (3.4%)	-0.02	6,309 (3.3%)	6,354 (3.3%)	0.00
Anxiolytics (except benzodiazepine)	60,549 (19.2%)	44,469 (18.6%)	0.02	36,409 (18.9%)	36,419 (18.9%)	0.00
Anxiolytics - Benzodiazepines	4,148 (1.3%)	2,952 (1.2%)	0.01	2,478 (1.3%)	2,440 (1.3%)	0.00
Bronchodilators	63,101 (20.0%)	47,469 (19.8%)	0.01	38,073 (19.8%)	38,177 (19.8%)	0.00
Corticosteroids, inhaled	73,755 (23.4%)	55,391 (23.1%)	0.01	44,408 (23.1%)	44,561 (23.1%)	0.00
Corticosteroids, oral	106,488 (33.7%)	78,565 (32.8%)	0.02	63,486 (33.0%)	63,555 (33.0%)	0.00
Dementia drugs	17,393 (5.5%)	12,672 (5.3%)	0.01	10,085 (5.2%)	10,196 (5.3%)	0.00
Diabetes agents - Insulin	24,012 (7.6%)	16,486 (6.9%)	0.03	13,560 (7.0%)	13,544 (7.0%)	0.00
Diabetes agents - Metformin	49,576 (15.7%)	38,670 (16.1%)	-0.01	31,316 (16.3%)	31,118 (16.2%)	0.00
Diabetes agents - Other	17,801 (5.6%)	13,075 (5.5%)	0.00	10,452 (5.4%)	10,462 (5.4%)	0.00
Diabetes agents - Sulfonylurea	28,905 (9.2%)	21,688 (9.1%)	0.00	17,198 (8.9%)	17,220 (8.9%)	0.00
Estrogen	11,370 (3.6%)	8,578 (3.6%)	0.00	6,603 (3.4%)	6,660 (3.5%)	-0.01
GI - H2 blockers	26,231 (8.3%)	18,300 (7.6%)	0.03	14,920 (7.7%)	15,038 (7.8%)	0.00
GI - Proton-pump inhibitors	106,405 (33.7%)	77,317 (32.3%)	0.03	62,250 (32.3%)	62,468 (32.4%)	0.00
GI - Sucralfate	6,758 (2.1%)	4,807 (2.0%)	0.01	3,841 (2.0%)	3,918 (2.0%)	0.00
Hypnotics	27,332 (8.7%)	22,465 (9.4%)	-0.02	17,227 (8.9%)	17,168 (8.9%)	0.00
NSAIDs	53,051 (16.8%)	43,315 (18.1%)	-0.03	34,114 (17.7%)	34,080 (17.7%)	0.00
Opioids	122,067 (38.7%)	93,926 (39.2%)	-0.01	74,867 (38.9%)	74,647 (38.7%)	0.00
Parkinsonism drugs	12,787 (4.1%)	9,360 (3.9%)	0.01	7,636 (4.0%)	7,576 (3.9%)	0.01
Thyroid hormone replacement	73,606 (23.3%)	51,828 (21.6%)	0.04	42,292 (22.0%)	42,326 (22.0%)	0.00
**Health care utilization**						
Emergency department visits	130,035 (41.2%)	94,381 (39.4%)	0.04	77,184 (40.1%)	77,420 (40.2%)	0.00
Home health day[C]	7,390 (2.3%)	12,618 (5.3%)	-0.16	6,829 (3.5%)	6,756 (3.5%)	0.00
Home oxygen use	11,022 (3.5%)	7,528 (3.1%)	0.02	6,012 (3.1%)	6,059 (3.1%)	0.00
Hospitalizations	169,477 (53.7%)	117,226 (48.9%)	0.10	94,033 (48.8%)	94,589 (49.1%)	-0.01
**Geographic Region**						
Northeast	61,382 (19.4%)	47,157 (19.7%)	-0.01	38,142 (19.8%)	38,149 (19.8%)	0.00
Midwest	67,521 (21.4%)	53,318 (22.3%)	-0.02	43,036 (22.3%)	43,001 (22.3%)	0.00
South	136,109 (43.1%)	95,141 (39.7%)	0.07	76,433 (39.7%)	76,682 (39.8%)	0.00
West	50,432 (16.0%)	43,695 (18.2%)	-0.06	34,844 (18.1%)	34,634 (18.0%)	0.00
Other	216 (0.1%)	289 (0.1%)	0.00	185 (0.1%)	174 (0.1%)	0.00

Abbreviations: ACE, angiotensin converting enzyme; CCI, combined comorbidity; CFI, claims-based frailty index; CKD, chronic kidney disease; COPD, chronic obstructive pulmonary disease; GI, gastrointestinal; NSAID, non-steroidal anti-inflammatory drug; PVD, peripheral vascular disease; SNRI, serotonin-norepinephrine reuptake inhibitor; SSRI, selective serotonin reuptake inhibitor.

[A] Other race category includes Unknown, Other, Asian, Hispanic, North American Native, and Missing Race Categories in Medicare. Asian, Hispanic, Unknown, and Missing categories are included for race in Optum. Race is unavailable in the MarketScan database.

[B] With both Medicare are Medicaid enrollment eligibility

[C] Home Health services received (days)

© 2023 Lin KJ et al. *JAMA Network Open*.

eTable 14. Study Population With Atrial Fibrillation Treated With Rivaroxaban vs Apixaban Before and After 1:1 Propensity Score Matching in the Optum Population

Characteristics<br>Number of Patients (%)	Before PS Matching			After PS Matching		
	Apixaban (n=138,803)	Rivaroxaban (n=52,875)	St. Diff	Apixaban (n=47,568)	Rivaroxaban (n=47,568)	St. Diff
**Quarter/Year of Cohort Entry Date**						
Q1 2013	36 (0.0%)	1,079 (2.0%)	-0.20	36 (0.1%)	37 (0.1%)	0.00
Q2 2013	192 (0.1%)	1,142 (2.2%)	-0.20	192 (0.4%)	212 (0.4%)	0.00
Q3 2013	247 (0.2%)	1,119 (2.1%)	-0.18	247 (0.5%)	303 (0.6%)	-0.01
Q4 2013	433 (0.3%)	1,205 (2.3%)	-0.18	433 (0.9%)	452 (1.0%)	-0.01
Q1 2014	671 (0.5%)	1,346 (2.5%)	-0.17	669 (1.4%)	705 (1.5%)	-0.01
Q2 2014	822 (0.6%)	1,279 (2.4%)	-0.15	811 (1.7%)	848 (1.8%)	-0.01
Q3 2014	902 (0.6%)	1,214 (2.3%)	-0.14	866 (1.8%)	862 (1.8%)	0.00
Q4 2014	1,060 (0.8%)	1,119 (2.1%)	-0.11	946 (2.0%)	939 (2.0%)	0.00
Q1 2015	1,405 (1.0%)	1,262 (2.4%)	-0.11	1,153 (2.4%)	1,149 (2.4%)	0.00
Q2 2015	1,524 (1.1%)	1,123 (2.1%)	-0.08	1,143 (2.4%)	1,086 (2.3%)	0.01
Q3 2015	1,609 (1.2%)	1,041 (2.0%)	-0.06	1,025 (2.2%)	1,032 (2.2%)	0.00
Q4 2015	1,701 (1.2%)	949 (1.8%)	-0.05	999 (2.1%)	948 (2.0%)	0.01
Q1 2016	2,600 (1.9%)	1,414 (2.7%)	-0.05	1,409 (3.0%)	1,413 (3.0%)	0.00
Q2 2016	2,344 (1.7%)	1,175 (2.2%)	-0.04	1,164 (2.4%)	1,174 (2.5%)	-0.01
Q3 2016	2,382 (1.7%)	1,187 (2.2%)	-0.04	1,183 (2.5%)	1,187 (2.5%)	0.00
Q4 2016	2,511 (1.8%)	1,083 (2.0%)	-0.01	1,128 (2.4%)	1,083 (2.3%)	0.01
Q1 2017	4,127 (3.0%)	1,985 (3.8%)	-0.04	1,980 (4.2%)	1,985 (4.2%)	0.00
Q2 2017	3,787 (2.7%)	1,701 (3.2%)	-0.03	1,738 (3.7%)	1,701 (3.6%)	0.01
Q3 2017	3,696 (2.7%)	1,668 (3.2%)	-0.03	1,695 (3.6%)	1,668 (3.5%)	0.01
Q4 2017	3,681 (2.7%)	1,627 (3.1%)	-0.02	1,621 (3.4%)	1,627 (3.4%)	0.00
Q1 2018	6,925 (5.0%)	2,613 (4.9%)	0.00	2,615 (5.5%)	2,613 (5.5%)	0.00
Q2 2018	5,956 (4.3%)	2,155 (4.1%)	0.01	2,174 (4.6%)	2,155 (4.5%)	0.00
Q3 2018	5,585 (4.0%)	1,967 (3.7%)	0.02	2,005 (4.2%)	1,967 (4.1%)	0.01
Q4 2018	5,897 (4.2%)	1,785 (3.4%)	0.04	1,800 (3.8%)	1,785 (3.8%)	0.00
Q1 2019	7,380 (5.3%)	2,097 (4.0%)	0.06	2,151 (4.5%)	2,097 (4.4%)	0.00
Q2 2019	7,093 (5.1%)	1,895 (3.6%)	0.07	1,924 (4.0%)	1,895 (4.0%)	0.00
Q3 2019	6,791 (4.9%)	1,734 (3.3%)	0.08	1,727 (3.6%)	1,734 (3.6%)	0.00
Q4 2019	6,968 (5.0%)	1,587 (3.0%)	0.10	1,589 (3.3%)	1,587 (3.3%)	0.00
Q1 2020	8,316 (6.0%)	2,072 (3.9%)	0.10	1,974 (4.1%)	2,072 (4.4%)	-0.01
Q2 2020	6,435 (4.6%)	1,490 (2.8%)	0.10	1,438 (3.0%)	1,490 (3.1%)	-0.01
Q3 2020	7,069 (5.1%)	1,451 (2.7%)	0.12	1,450 (3.0%)	1,451 (3.1%)	-0.01
Q4 2020	7,255 (5.2%)	1,436 (2.7%)	0.13	1,381 (2.9%)	1,436 (3.0%)	-0.01
Q1 2021	12,518 (9.0%)	3,057 (5.8%)	0.12	3,128 (6.6%)	3,057 (6.4%)	0.01
Q2 2021	8,885 (6.4%)	1,818 (3.4%)	0.14	1,774 (3.7%)	1,818 (3.8%)	-0.01
**Sociodemographic**						
Mean age (SD)	77.15 (6.84)	76.05 (6.65)	0.16	76.14 (6.69)	76.17 (6.68)	0.00
Male	69,290 (49.9%)	28,580 (54.1%)	-0.08	25,531 (53.7%)	25,584 (53.8%)	0.00
Female	69,513 (50.1%)	24,295 (45.9%)	0.08	22,037 (46.3%)	21,984 (46.2%)	0.00
Black	12,839 (9.2%)	4,227 (8.0%)	0.04	3,843 (8.1%)	3,862 (8.1%)	0.00
White	104,026 (74.9%)	39,772 (75.2%)	-0.01	35,707 (75.1%)	35,605 (74.9%)	0.00
Other[A]	21,938 (15.8%)	8,876 (16.8%)	-0.03	8,018 (16.9%)	8,101 (17.0%)	0.00
**Cardiovascular conditions**						
Acute myocardial infarction	9,987 (7.2%)	2,758 (5.2%)	0.08	2,422 (5.1%)	2,508 (5.3%)	-0.01
Cardio-ablation	826 (0.6%)	271 (0.5%)	0.01	249 (0.5%)	224 (0.5%)	0.00
Cardioversion	9,896 (7.1%)	3,663 (6.9%)	0.01	3,178 (6.7%)	3,204 (6.7%)	0.00
Cerebrovascular disease	35,539 (25.6%)	11,254 (21.3%)	0.10	10,003 (21.0%)	10,180 (21.4%)	-0.01
Congestive heart failure (inpatient)	22,494 (16.2%)	6,306 (11.9%)	0.12	5,574 (11.7%)	5,744 (12.1%)	-0.01
Congestive heart failure (outpatient)	50,578 (36.4%)	17,704 (33.5%)	0.06	15,611 (32.8%)	15,749 (33.1%)	-0.01
Coronary revascularization	4,767 (3.4%)	1,253 (2.4%)	0.06	1,123 (2.4%)	1,141 (2.4%)	0.00
Hypertension	123,863 (89.2%)	46,231 (87.4%)	0.06	41,571 (87.4%)	41,615 (87.5%)	0.00
Ischemic Heart Disease	63,105 (45.5%)	21,765 (41.2%)	0.09	19,479 (40.9%)	19,572 (41.1%)	0.00
PVD or PVD Surgery	22,271 (16.0%)	7,879 (14.9%)	0.03	7,045 (14.8%)	7,145 (15.0%)	-0.01
Stroke (inpatient)	9,967 (7.2%)	2,385 (4.5%)	0.12	2,178 (4.6%)	2,198 (4.6%)	0.00
Stroke (outpatient)	34,058 (24.5%)	10,597 (20.0%)	0.11	9,501 (20.0%)	9,649 (20.3%)	-0.01
Syncope	16,264 (11.7%)	5,347 (10.1%)	0.05	4,769 (10.0%)	4,852 (10.2%)	-0.01

© 2023 Lin KJ et al. *JAMA Network Open*.

Characteristics<br>Number of Patients (%)	Before PS Matching			After PS Matching		
	Apixaban<br>(n=138,803)	Rivaroxaban<br>(n=52,875)	St. Diff	Apixaban<br>(n=47,568)	Rivaroxaban<br>(n=47,568)	St. Diff
Mean CHA_2DS_2-VASc score (SD)	4.78 (1.69)	4.44 (1.66)	0.20	4.46 (1.66)	4.47 (1.66)	-0.01
Mean HAS-BLED score (SD)	2.35 (0.74)	2.25 (0.69)	0.14	2.25 (0.70)	2.25 (0.70)	0.00
**Non-cardiovascular conditions**						
Acute renal failure	21,998 (15.8%)	5,352 (10.1%)	0.17	4,887 (10.3%)	5,045 (10.6%)	-0.01
Alcohol abuse or dependence	4,393 (3.2%)	1,521 (2.9%)	0.02	1,383 (2.9%)	1,410 (3.0%)	-0.01
Anemia	36,615 (26.4%)	11,922 (22.5%)	0.09	10,787 (22.7%)	10,862 (22.8%)	0.00
CKD: stage 3, 4 or unspecified	34,281 (24.7%)	10,303 (19.5%)	0.13	9,516 (20.0%)	9,596 (20.2%)	0.00
COPD	33,397 (24.1%)	11,490 (21.7%)	0.06	10,361 (21.8%)	10,471 (22.0%)	0.00
Dementia	11,330 (8.2%)	3,606 (6.8%)	0.05	3,289 (6.9%)	3,336 (7.0%)	0.00
Diabetes	51,834 (37.3%)	18,739 (35.4%)	0.04	17,012 (35.8%)	16,982 (35.7%)	0.00
Endoscopy	2,184 (1.6%)	622 (1.2%)	0.03	561 (1.2%)	552 (1.2%)	0.00
Falls	19,398 (14.0%)	5,633 (10.7%)	0.10	5,275 (11.1%)	5,337 (11.2%)	0.00
Fractures	11,412 (8.2%)	4,120 (7.8%)	0.01	3,749 (7.9%)	3,750 (7.9%)	0.00
GI bleeding (inpatient)	3,596 (2.6%)	1,053 (2.0%)	0.04	950 (2.0%)	953 (2.0%)	0.00
GI bleeding (outpatient)	30,697 (22.1%)	11,610 (22.0%)	0.00	10,236 (21.5%)	10,273 (21.6%)	0.00
Liver disease	11,565 (8.3%)	3,620 (6.8%)	0.06	3,327 (7.0%)	3,360 (7.1%)	0.00
Malignancy	22,507 (16.2%)	8,106 (15.3%)	0.02	7,252 (15.2%)	7,265 (15.3%)	0.00
Obesity	33,397 (24.1%)	11,606 (21.9%)	0.05	10,762 (22.6%)	10,825 (22.8%)	0.00
Peptic Ulcer	4,083 (2.9%)	1,005 (1.9%)	0.07	908 (1.9%)	963 (2.0%)	-0.01
Smoking	47,361 (34.1%)	14,530 (27.5%)	0.14	13,619 (28.6%)	13,742 (28.9%)	-0.01
Mean CCI score (SD)	4.12 (3.14)	3.46 (2.86)	0.22	3.52 (2.87)	3.56 (2.90)	-0.01
Mean CFI (SD)	0.20 (0.07)	0.19 (0.06)	0.15	0.19 (0.06)	0.19 (0.06)	0.00
**Cardiovascular medications**						
ACE inhibitors	36,817 (26.5%)	13,909 (26.3%)	0.00	12,375 (26.0%)	12,402 (26.1%)	0.00
Angiotensin II receptor blockers	7,192 (5.2%)	2,788 (5.3%)	0.00	2,455 (5.2%)	2,473 (5.2%)	0.00
Antiarrhythmic agents	24,720 (17.8%)	9,172 (17.3%)	0.01	8,092 (17.0%)	8,081 (17.0%)	0.00
Anticoagulants, injectable	663 (0.5%)	280 (0.5%)	0.00	238 (0.5%)	243 (0.5%)	0.00
Antiplatelet agent	19,935 (14.4%)	6,729 (12.7%)	0.05	6,076 (12.8%)	5,973 (12.6%)	0.01
Beta-blockers	92,873 (66.9%)	33,920 (64.2%)	0.06	30,552 (64.2%)	30,620 (64.4%)	0.00
Calcium channel blockers	57 (0.0%)	37 (0.1%)	-0.04	35 (0.1%)	29 (0.1%)	0.00
Diuretics	71,081 (51.2%)	25,828 (48.8%)	0.05	23,276 (48.9%)	23,272 (48.9%)	0.00
Fibrates	5,028 (3.6%)	2,068 (3.9%)	-0.02	1,814 (3.8%)	1,830 (3.8%)	0.00
Nitrates	14,175 (10.2%)	4,616 (8.7%)	0.05	4,003 (8.4%)	4,115 (8.7%)	-0.01
Statins	88,549 (63.8%)	31,984 (60.5%)	0.07	28,934 (60.8%)	29,017 (61.0%)	0.00
**Other medications**						
Anticonvulsants	24,297 (17.5%)	7,836 (14.8%)	0.07	7,386 (15.5%)	7,310 (15.4%)	0.00
Antidepressants - Other	13,254 (9.5%)	4,380 (8.3%)	0.04	4,079 (8.6%)	4,099 (8.6%)	0.00
Antidepressants - SSRI/SNRI	27,979 (20.2%)	9,474 (17.9%)	0.06	8,650 (18.2%)	8,678 (18.2%)	0.00
Antidepressants - Tricyclics	3,184 (2.3%)	1,109 (2.1%)	0.01	967 (2.0%)	969 (2.0%)	0.00
Antipsychotic agents	3,681 (2.7%)	1,178 (2.2%)	0.03	1,054 (2.2%)	1,105 (2.3%)	-0.01
Anxiolytics (except benzodiazepine)	20,773 (15.0%)	7,714 (14.6%)	0.01	6,982 (14.7%)	6,987 (14.7%)	0.00
Anxiolytics - Benzodiazepines	2,113 (1.5%)	639 (1.2%)	0.03	664 (1.4%)	611 (1.3%)	0.01
Bronchodilators	27,898 (20.1%)	9,881 (18.7%)	0.04	8,916 (18.7%)	8,964 (18.8%)	0.00
Corticosteroids, inhaled	27,635 (19.9%)	10,221 (19.3%)	0.02	9,154 (19.2%)	9,147 (19.2%)	0.00
Corticosteroids, oral	42,603 (30.7%)	15,153 (28.7%)	0.04	13,725 (28.9%)	13,744 (28.9%)	0.00
Dementia drugs	6,113 (4.4%)	2,132 (4.0%)	0.02	1,928 (4.1%)	1,960 (4.1%)	0.00
Diabetes agents - Insulin	10,047 (7.2%)	3,243 (6.1%)	0.04	2,943 (6.2%)	3,001 (6.3%)	0.00
Diabetes agents - Metformin	24,386 (17.6%)	9,129 (17.3%)	0.01	8,457 (17.8%)	8,327 (17.5%)	0.01
Diabetes agents - Other	12,772 (9.2%)	4,657 (8.8%)	0.01	4,145 (8.7%)	4,194 (8.8%)	0.00
Diabetes agents - Sulfonylurea	6,966 (5.0%)	2,609 (4.9%)	0.00	2,372 (5.0%)	2,367 (5.0%)	0.00
Estrogen	3,131 (2.3%)	1,209 (2.3%)	0.00	1,023 (2.2%)	1,029 (2.2%)	0.00
GI - H2 blockers	10,549 (7.6%)	3,407 (6.4%)	0.05	3,125 (6.6%)	3,180 (6.7%)	0.00
GI - Proton-pump inhibitors	41,692 (30.0%)	14,496 (27.4%)	0.06	13,023 (27.4%)	13,157 (27.7%)	-0.01
GI - Sucralfates	2,626 (1.9%)	804 (1.5%)	0.03	710 (1.5%)	732 (1.5%)	0.00
Hypnotics	8,522 (6.1%)	3,412 (6.5%)	-0.02	2,932 (6.2%)	2,902 (6.1%)	0.00
NSAIDs	21,467 (15.5%)	8,366 (15.8%)	-0.01	7,408 (15.6%)	7,473 (15.7%)	0.00
Opioids	45,213 (32.6%)	17,247 (32.6%)	0.00	15,392 (32.4%)	15,366 (32.3%)	0.00
Parkinsonism drugs	4,858 (3.5%)	1,583 (3.0%)	0.03	1,450 (3.0%)	1,465 (3.1%)	-0.01
Thyroid hormone replacement	28,941 (20.9%)	10,321 (19.5%)	0.03	9,241 (19.4%)	9,322 (19.6%)	-0.01
**Health care utilization**						

© 2023 Lin KJ et al. *JAMA Network Open*.

Characteristics<br>Number of Patients (%)	Before PS Matching			After PS Matching		
	Apixaban<br>(n=138,803)	Rivaroxaban<br>(n=52,875)	St. Diff	Apixaban<br>(n=47,568)	Rivaroxaban<br>(n=47,568)	St. Diff
ED Visit	77,116 (55.6%)	25,630 (48.5%)	0.14	23,051 (48.5%)	23,254 (48.9%)	-0.01
Home oxygen use	6,617 (4.8%)	1,727 (3.3%)	0.08	1,604 (3.4%)	1,634 (3.4%)	0.00
Hospitalizations	64,396 (46.4%)	19,399 (36.7%)	0.20	17,204 (36.2%)	17,523 (36.8%)	-0.01
**Geographic Region**						
Northeast	16,652 (12.0%)	7,078 (13.4%)	-0.04	6,142 (12.9%)	6,244 (13.1%)	-0.01
Midwest	26,429 (19.0%)	9,654 (18.3%)	0.02	8,778 (18.5%)	8,799 (18.5%)	0.00
South	60,469 (43.6%)	20,722 (39.2%)	0.09	18,890 (39.7%)	18,830 (39.6%)	0.00
West	35,179 (25.3%)	15,384 (29.1%)	-0.09	13,720 (28.8%)	13,660 (28.7%)	0.00
Other	74 (0.1%)	37 (0.1%)	0.00	38 (0.1%)	35 (0.1%)	0.00
**Business Type Code (Recategorized)**						
Commercial	8,666 (6.2%)	4,961 (9.4%)	-0.12	4,051 (8.5%)	4,020 (8.5%)	0.00
Medicare	130,137 (93.8%)	47,914 (90.6%)	0.12	43,517 (91.5%)	43,548 (91.5%)	0.00

Abbreviations: ACE, angiotensin converting enzyme; CCI, combined comorbidity; CFI, claims-based frailty index; CKD, chronic kidney disease; COPD, chronic obstructive pulmonary disease; GI, gastrointestinal; NSAID, non-steroidal anti-inflammatory drug; PVD, peripheral vascular disease; SNRI, serotonin-norepinephrine reuptake inhibitor; SSRI, selective serotonin reuptake inhibitor.

[A] Other race category includes Unknown, Other, Asian, Hispanic, North American Native, and Missing Race Categories in Medicare. Asian, Hispanic, Unknown, and Missing categories are included for race in Optum. Race is unavailable in the MarketScan database.

© 2023 Lin KJ et al. *JAMA Network Open*.

eTable 15. Study Population With Atrial Fibrillation Treated With Rivaroxaban vs Apixaban Before and After 1:1 Propensity Score Matching in the MarketScan Population

Characteristics Number of Patients (%)	Before PS Matching			After PS Matching		
	Apixaban (n=52,306)	Dabigatran (n=34,814)	St. Diff	Apixaban (n=25,669)	Warfarin (n=25,669)	St. Diff
**Quarter/Year of Cohort Entry Date**						
Q1 2013	98 (0.2%)	1,985 (5.7%)	-0.33	98 (0.4%)	100 (0.4%)	0.00
Q2 2013	513 (1.0%)	2,055 (5.9%)	-0.27	513 (2.0%)	625 (2.4%)	-0.03
Q3 2013	762 (1.5%)	2,183 (6.3%)	-0.25	762 (3.0%)	806 (3.1%)	-0.01
Q4 2013	1,111 (2.1%)	2,280 (6.5%)	-0.22	1,111 (4.3%)	1,118 (4.4%)	0.00
Q1 2014	1,039 (2.0%)	2,090 (6.0%)	-0.21	1,039 (4.0%)	971 (3.8%)	0.01
Q2 2014	1,320 (2.5%)	2,183 (6.3%)	-0.19	1,314 (5.1%)	1,286 (5.0%)	0.00
Q3 2014	1,404 (2.7%)	2,028 (5.8%)	-0.15	1,387 (5.4%)	1,311 (5.1%)	0.01
Q4 2014	1,855 (3.5%)	2,046 (5.9%)	-0.11	1,708 (6.7%)	1,668 (6.5%)	0.01
Q1 2015	1,756 (3.4%)	1,345 (3.9%)	-0.03	1,318 (5.1%)	1,282 (5.0%)	0.00
Q2 2015	1,758 (3.4%)	1,330 (3.8%)	-0.02	1,262 (4.9%)	1,273 (5.0%)	0.00
Q3 2015	1,911 (3.7%)	1,287 (3.7%)	0.00	1,274 (5.0%)	1,253 (4.9%)	0.00
Q4 2015	2,027 (3.9%)	1,210 (3.5%)	0.02	1,184 (4.6%)	1,192 (4.6%)	0.00
Q1 2016	2,410 (4.6%)	1,159 (3.3%)	0.07	1,160 (4.5%)	1,158 (4.5%)	0.00
Q2 2016	2,518 (4.8%)	1,273 (3.7%)	0.05	1,255 (4.9%)	1,269 (4.9%)	0.00
Q3 2016	2,657 (5.1%)	1,156 (3.3%)	0.09	1,221 (4.8%)	1,156 (4.5%)	0.01
Q4 2016	2,711 (5.2%)	1,097 (3.2%)	0.10	1,095 (4.3%)	1,097 (4.3%)	0.00
Q1 2017	1,903 (3.6%)	827 (2.4%)	0.07	818 (3.2%)	827 (3.2%)	0.00
Q2 2017	1,888 (3.6%)	792 (2.3%)	0.08	782 (3.0%)	792 (3.1%)	-0.01
Q3 2017	1,940 (3.7%)	822 (2.4%)	0.08	846 (3.3%)	820 (3.2%)	0.01
Q4 2017	2,139 (4.1%)	807 (2.3%)	0.10	787 (3.1%)	807 (3.1%)	0.00
Q1 2018	1,450 (2.8%)	502 (1.4%)	0.10	496 (1.9%)	502 (2.0%)	-0.01
Q2 2018	1,430 (2.7%)	516 (1.5%)	0.08	514 (2.0%)	516 (2.0%)	0.00
Q3 2018	1,415 (2.7%)	478 (1.4%)	0.09	459 (1.8%)	478 (1.9%)	-0.01
Q4 2018	1,443 (2.8%)	437 (1.3%)	0.11	428 (1.7%)	437 (1.7%)	0.00
Q1 2019	1,674 (3.2%)	451 (1.3%)	0.13	451 (1.8%)	451 (1.8%)	0.00
Q2 2019	1,903 (3.6%)	490 (1.4%)	0.14	462 (1.8%)	490 (1.9%)	-0.01
Q3 2019	1,839 (3.5%)	468 (1.3%)	0.14	431 (1.7%)	467 (1.8%)	-0.01
Q4 2019	1,767 (3.4%)	432 (1.2%)	0.15	421 (1.6%)	432 (1.7%)	-0.01
Q1 2020	1,573 (3.0%)	326 (0.9%)	0.15	347 (1.4%)	326 (1.3%)	0.01
Q2 2020	1,063 (2.0%)	208 (0.6%)	0.12	188 (0.7%)	208 (0.8%)	-0.01
Q3 2020	1,513 (2.9%)	257 (0.7%)	0.17	251 (1.0%)	257 (1.0%)	0.00
Q4 2020	1,516 (2.9%)	294 (0.8%)	0.16	287 (1.1%)	294 (1.1%)	0.00
**Sociodemographic**						
Mean Age (SD)	77.69 (7.81)	76.37 (7.41)	0.17	76.63 (7.65)	76.68 (7.47)	-0.01
Male	27,511 (52.6%)	19,405 (55.7%)	-0.06	14,159 (55.2%)	14,222 (55.4%)	0.00
Female	24,795 (47.4%)	15,409 (44.3%)	0.06	11,510 (44.8%)	11,447 (44.6%)	0.00
**Cardiovascular conditions**						
Acute Myocardial infarction	3,748 (7.2%)	1,843 (5.3%)	0.08	1,467 (5.7%)	1,439 (5.6%)	0.00
Cardio-ablation	295 (0.6%)	213 (0.6%)	0.00	148 (0.6%)	155 (0.6%)	0.00
Cardioversion	4,108 (7.9%)	2,618 (7.5%)	0.02	1,955 (7.6%)	1,948 (7.6%)	0.00
Cerebrovascular disease	13,234 (25.3%)	7,715 (22.2%)	0.07	5,841 (22.8%)	5,908 (23.0%)	0.00
Congestive heart failure (inpatient)	9,915 (19.0%)	5,665 (16.3%)	0.07	4,273 (16.6%)	4,251 (16.6%)	0.00
Congestive heart failure (outpatient)	13,864 (26.5%)	8,672 (24.9%)	0.04	6,507 (25.3%)	6,524 (25.4%)	0.00
Coronary revascularization	1,676 (3.2%)	874 (2.5%)	0.04	692 (2.7%)	669 (2.6%)	0.01
Hypertension	44,954 (85.9%)	28,987 (83.3%)	0.07	21,814 (85.0%)	21,753 (84.7%)	0.01
Ischemic Heart Disease	23,589 (45.1%)	14,409 (41.4%)	0.07	10,869 (42.3%)	10,834 (42.2%)	0.00
PVD or PVD Surgery	6,724 (12.9%)	4,050 (11.6%)	0.04	3,051 (11.9%)	3,092 (12.0%)	0.00
Stroke (inpatient)	5,027 (9.6%)	2,647 (7.6%)	0.07	2,002 (7.8%)	2,046 (8.0%)	-0.01
Stroke (outpatient)	11,329 (21.7%)	6,316 (18.1%)	0.09	4,879 (19.0%)	4,924 (19.2%)	-0.01
Syncope	6,114 (11.7%)	3,598 (10.3%)	0.04	2,776 (10.8%)	2,746 (10.7%)	0.00
Mean CHA_2DS_2-VASc score (SD)	3.53 (1.60)	3.62 (1.54)	-0.06	3.56 (1.57)	3.55 (1.59)	0.01
Mean HAS-BLED score (SD)	2.22 (0.70)	2.12 (0.66)	0.15	2.15 (0.66)	2.15 (0.67)	0.00
**Non-cardiovascular conditions**						
Acute renal failure	6,118 (11.7%)	2,746 (7.9%)	0.13	2,218 (8.6%)	2,230 (8.7%)	0.00
Alcohol abuse or dependence	926 (1.8%)	600 (1.7%)	0.01	481 (1.9%)	463 (1.8%)	0.01
Anemia	10,932 (20.9%)	6,022 (17.3%)	0.09	4,611 (18.0%)	4,690 (18.3%)	-0.01

© 2023 Lin KJ et al. *JAMA Network Open*.

Characteristics Number of Patients (%)	Before PS Matching			After PS Matching		
	Apixaban (n=52,306)	Dabigatran (n=34,814)	St. Diff	Apixaban (n=25,669)	Warfarin (n=25,669)	St. Diff
CKD: stage 3, 4 or unspecified	8,042 (15.4%)	3,738 (10.7%)	0.14	3,101 (12.1%)	3,063 (11.9%)	0.01
COPD	10,462 (20.0%)	6,573 (18.9%)	0.03	4,926 (19.2%)	4,852 (18.9%)	0.01
Dementia	3,314 (6.3%)	1,815 (5.2%)	0.05	1,431 (5.6%)	1,436 (5.6%)	0.00
Diabetes	16,556 (31.7%)	10,755 (30.9%)	0.02	8,132 (31.7%)	8,078 (31.5%)	0.00
Endoscopy	641 (1.2%)	375 (1.1%)	0.01	277 (1.1%)	283 (1.1%)	0.00
Falls	4,145 (7.9%)	2,067 (5.9%)	0.08	1,710 (6.7%)	1,694 (6.6%)	0.00
Fractures	4,282 (8.2%)	2,684 (7.7%)	0.02	2,014 (7.8%)	2,022 (7.9%)	0.00
GI bleeding (inpatient)	1,681 (3.2%)	1,151 (3.3%)	-0.01	790 (3.1%)	817 (3.2%)	-0.01
GI bleeding (outpatient)	6,721 (12.8%)	4,850 (13.9%)	-0.03	3,445 (13.4%)	3,455 (13.5%)	0.00
Liver disease	3,168 (6.1%)	1,780 (5.1%)	0.04	1,375 (5.4%)	1,380 (5.4%)	0.00
Malignancy	9,614 (18.4%)	6,507 (18.7%)	-0.01	4,696 (18.3%)	4,703 (18.3%)	0.00
Obesity	7,611 (14.6%)	4,610 (13.2%)	0.04	3,682 (14.3%)	3,637 (14.2%)	0.00
Peptic Ulcer	847 (1.6%)	379 (1.1%)	0.04	298 (1.2%)	313 (1.2%)	0.00
Smoking	7,349 (14.1%)	4,208 (12.1%)	0.06	3,377 (13.2%)	3,387 (13.2%)	0.00
Mean CCI score (SD)	3.20 (2.71)	2.65 (2.44)	0.21	2.79 (2.50)	2.80 (2.52)	0.00
Mean CFI (SD)	0.19 (0.06)	0.19 (0.06)	0.00	0.19 (0.06)	0.19 (0.06)	0.00
**Cardiovascular medications**						
ACE inhibitors	13,280 (25.4%)	8,734 (25.1%)	0.01	6,468 (25.2%)	6,483 (25.3%)	0.00
Angiotensin II receptor blockers	3,306 (6.3%)	2,267 (6.5%)	-0.01	1,626 (6.3%)	1,638 (6.4%)	0.00
Antiarrhythmic agents	9,530 (18.2%)	6,589 (18.9%)	-0.02	4,713 (18.4%)	4,665 (18.2%)	0.01
Anticoagulants, injectable	245 (0.5%)	224 (0.6%)	-0.01	161 (0.6%)	150 (0.6%)	0.00
Antiplatelet agent	9,348 (17.9%)	5,451 (15.7%)	0.06	4,104 (16.0%)	4,064 (15.8%)	0.01
Beta-blockers	35,227 (67.3%)	22,470 (64.5%)	0.06	16,700 (65.1%)	16,807 (65.5%)	-0.01
Calcium channel blockers	102 (0.2%)	69 (0.2%)	0.00	46 (0.2%)	58 (0.2%)	0.00
Diuretics	26,566 (50.8%)	16,927 (48.6%)	0.04	12,712 (49.5%)	12,651 (49.3%)	0.00
Fibrates	2,263 (4.3%)	1,651 (4.7%)	-0.02	1,204 (4.7%)	1,179 (4.6%)	0.00
Nitrates	33,566 (64.2%)	21,278 (61.1%)	0.06	15,892 (61.9%)	15,892 (61.9%)	0.00
Statins	5,868 (11.2%)	3,453 (9.9%)	0.04	2,643 (10.3%)	2,615 (10.2%)	0.00
**Other medications**						
Anticonvulsants	7,547 (14.4%)	4,432 (12.7%)	0.05	3,400 (13.2%)	3,416 (13.3%)	0.00
Antidepressants - Other	3,858 (7.4%)	2,271 (6.5%)	0.04	1,785 (7.0%)	1,771 (6.9%)	0.00
Antidepressants - SSRI/SNRI	9,760 (18.7%)	5,885 (16.9%)	0.05	4,535 (17.7%)	4,508 (17.6%)	0.00
Antidepressants - Tricyclics	1,197 (2.3%)	846 (2.4%)	-0.01	617 (2.4%)	590 (2.3%)	0.01
Antipsychotic agents	1,200 (2.3%)	720 (2.1%)	0.01	567 (2.2%)	553 (2.2%)	0.00
Anxiolytics (except benzodiazepine)	8,156 (15.6%)	5,760 (16.5%)	-0.02	4,183 (16.3%)	4,154 (16.2%)	0.00
Anxiolytics - Benzodiazepines	592 (1.1%)	318 (0.9%)	0.02	258 (1.0%)	244 (1.0%)	0.00
Bronchodilators	10,142 (19.4%)	6,390 (18.4%)	0.03	4,849 (18.9%)	4,776 (18.6%)	0.01
Corticosteroids, inhaled	11,735 (22.4%)	7,659 (22.0%)	0.01	5,725 (22.3%)	5,704 (22.2%)	0.00
Corticosteroids, oral	16,812 (32.1%)	10,632 (30.5%)	0.03	8,015 (31.2%)	7,894 (30.8%)	0.01
Dementia drugs	2,279 (4.4%)	1,395 (4.0%)	0.02	1,069 (4.2%)	1,062 (4.1%)	0.01
Diabetes agents - Insulin	3,721 (7.1%)	2,235 (6.4%)	0.03	1,714 (6.7%)	1,709 (6.7%)	0.00
Diabetes agents - Metformin	8,105 (15.5%)	5,382 (15.5%)	0.00	4,078 (15.9%)	4,014 (15.6%)	0.01
Diabetes agents - Other	3,084 (5.9%)	2,046 (5.9%)	0.00	1,503 (5.9%)	1,506 (5.9%)	0.00
Diabetes agents - Sulfonylurea	4,332 (8.3%)	2,946 (8.5%)	-0.01	2,190 (8.5%)	2,170 (8.5%)	0.00
Estrogen	1,893 (3.6%)	1,264 (3.6%)	0.00	910 (3.5%)	891 (3.5%)	0.00
GI - H2 blockers	3,767 (7.2%)	1,995 (5.7%)	0.06	1,591 (6.2%)	1,609 (6.3%)	0.00
GI - Proton-pump inhibitors	15,975 (30.5%)	9,753 (28.0%)	0.05	7,487 (29.2%)	7,401 (28.8%)	0.01
GI - Sucralfate	914 (1.7%)	500 (1.4%)	0.02	403 (1.6%)	377 (1.5%)	0.01
Hypnotics	3,863 (7.4%)	2,795 (8.0%)	-0.02	2,023 (7.9%)	1,969 (7.7%)	0.01
NSAIDs	8,409 (16.1%)	5,826 (16.7%)	-0.02	4,294 (16.7%)	4,262 (16.6%)	0.00
Opioids	18,677 (35.7%)	13,202 (37.9%)	-0.05	9,694 (37.8%)	9,541 (37.2%)	0.01
Parkinsonism drugs	1,800 (3.4%)	1,121 (3.2%)	0.01	835 (3.3%)	859 (3.3%)	0.00
Thyroid hormone replacement	11,144 (21.3%)	6,870 (19.7%)	0.04	5,133 (20.0%)	5,135 (20.0%)	0.00
**Health care utilization**						
Emergency department visits	28,739 (54.9%)	17,687 (50.8%)	0.08	13,199 (51.4%)	13,290 (51.8%)	-0.01
Hospitalizations	25,440 (48.6%)	15,176 (43.6%)	0.10	11,231 (43.8%)	11,241 (43.8%)	0.00
**Geographic Region**						
Northeast	14,276 (27.3%)	8,492 (24.4%)	0.07	6,427 (25.0%)	6,489 (25.3%)	-0.01
Midwest	16,271 (31.1%)	11,074 (31.8%)	-0.02	8,239 (32.1%)	8,270 (32.2%)	0.00
South	16,214 (31.0%)	10,636 (30.6%)	0.01	7,927 (30.9%)	7,908 (30.8%)	0.00

© 2023 Lin KJ et al. *JAMA Network Open*.

Characteristics<br>Number of Patients (%)	Before PS Matching			After PS Matching		
	Apixaban (n=52,306)	Dabigatran (n=34,814)	St. Diff	Apixaban (n=25,669)	Warfarin (n=25,669)	St. Diff
West	5,441 (10.4%)	4,488 (12.9%)	-0.08	3,003 (11.7%)	2,933 (11.4%)	0.01
Other	104 (0.2%)	124 (0.4%)	-0.04	73 (0.3%)	69 (0.3%)	0.00
**Employee Classification**						
Salary Non-union	4,700 (9.0%)	3,458 (9.9%)	-0.03	2,432 (9.5%)	2,409 (9.4%)	0.00
Salary Union	1,241 (2.4%)	734 (2.1%)	0.02	590 (2.3%)	574 (2.2%)	0.01
Salary Other	918 (1.8%)	711 (2.0%)	-0.01	504 (2.0%)	514 (2.0%)	0.00
Hourly Non-Union	2,627 (5.0%)	1,869 (5.4%)	-0.02	1,341 (5.2%)	1,341 (5.2%)	0.00
Hourly Union	12,460 (23.8%)	9,694 (27.8%)	-0.09	6,955 (27.1%)	6,967 (27.1%)	0.00
Hourly Other	248 (0.5%)	295 (0.8%)	-0.04	181 (0.7%)	176 (0.7%)	0.00
Non-Union	14,359 (27.5%)	7,089 (20.4%)	0.17	5,714 (22.3%)	5,772 (22.5%)	0.00
Union	784 (1.5%)	495 (1.4%)	0.01	380 (1.5%)	388 (1.5%)	0.00
Unknown	14,969 (28.6%)	10,469 (30.1%)	-0.03	7,572 (29.5%)	7,528 (29.3%)	0.00
**Employment Status**						
Active Full Time	3,615 (6.9%)	2,230 (6.4%)	0.02	1,751 (6.8%)	1,773 (6.9%)	0.00
Active Part Time or Seasonal	163 (0.3%)	79 (0.2%)	0.02	68 (0.3%)	62 (0.2%)	0.02
Early Retiree	559 (1.1%)	439 (1.3%)	-0.02	320 (1.2%)	333 (1.3%)	-0.01
Medicare Eligible Retiree	36,762 (70.3%)	24,399 (70.1%)	0.00	18,088 (70.5%)	18,096 (70.5%)	0.00
Retiree	508 (1.0%)	844 (2.4%)	-0.11	344 (1.3%)	350 (1.4%)	-0.01
COBRA Continuee	30 (0.1%)	18 (0.1%)	0.00	10 (0.0%)	12 (0.0%)	--
Long Term Disability	44 (0.1%)	35 (0.1%)	0.00	28 (0.1%)	24 (0.1%)	0.00
Surviving Spouse/Dependent	4,799 (9.2%)	2,811 (8.1%)	0.04	2,111 (8.2%)	2,145 (8.4%)	-0.01
Other/Unknown/Missing	5,826 (11.1%)	3,959 (11.4%)	-0.01	2,949 (11.5%)	2,874 (11.2%)	0.01
**Health Plan Indicator**						
Employer	45,823 (87.6%)	30,520 (87.7%)	0.00	22,439 (87.4%)	22,522 (87.7%)	-0.01
Health Plan	6,483 (12.4%)	4,294 (12.3%)	0.00	3,230 (12.6%)	3,147 (12.3%)	0.01
**MHSA Coverage Indicator**						
Not Covered/Claims Not Present	2,882 (5.5%)	2,039 (5.9%)	-0.02	1,464 (5.7%)	1,441 (5.6%)	0.00
Covered/Possible MHSA Claims	43,411 (83.0%)	29,204 (83.9%)	-0.02	21,456 (83.6%)	21,435 (83.5%)	0.00
Missing	6,013 (11.5%)	3,571 (10.3%)	0.04	2,749 (10.7%)	2,793 (10.9%)	-0.01
**Plan Indicator**						
Basic/major medical	0 (0.0%)	1 (0.0%)	--	0 (0.0%)	0 (0.0%)	--
Comprehensive	17,046 (32.6%)	13,177 (37.8%)	-0.11	9,360 (36.5%)	9,348 (36.4%)	0.00
EPO	120 (0.2%)	89 (0.3%)	-0.02	72 (0.3%)	73 (0.3%)	0.00
HMO	5,565 (10.6%)	3,054 (8.8%)	0.06	2,387 (9.3%)	2,310 (9.0%)	0.01
POS	1,568 (3.0%)	1,258 (3.6%)	-0.03	920 (3.6%)	926 (3.6%)	0.00
PPO	26,065 (49.8%)	16,039 (46.1%)	0.07	11,951 (46.6%)	12,036 (46.9%)	-0.01
POS with capitation	692 (1.3%)	436 (1.3%)	0.00	393 (1.5%)	396 (1.5%)	0.00
CDHP	383 (0.7%)	277 (0.8%)	-0.01	206 (0.8%)	209 (0.8%)	0.00
HDHP	241 (0.5%)	117 (0.3%)	0.03	101 (0.4%)	98 (0.4%)	0.00
Missing	626 (1.2%)	366 (1.1%)	0.01	279 (1.1%)	273 (1.1%)	0.00

Abbreviations: ACE, angiotensin converting enzyme; CCI, combined comorbidity; CFI, claims-based frailty index; CKD, chronic kidney disease; COPD, chronic obstructive pulmonary disease; GI, gastrointestinal; NSAID, non-steroidal anti-inflammatory drug; PVD, peripheral vascular disease; SNRI, serotonin-norepinephrine reuptake inhibitor; SSRI, selective serotonin reuptake inhibitor.

© 2023 Lin KJ et al. *JAMA Network Open*.

eTable 16. Selected Characteristics of Propensity Score-Matched Population With Atrial Fibrillation Treated With Warfarin vs Apixaban by Dementia Diagnosis Pooled Across Medicare, Optum, and MarketScan Populations

Characteristics Number of Patients (%)	With Dementia			Without Dementia		
	Apixaban (n=20,175)	Warfarin (n=20,175)	St. Diff	Apixaban (n=230,405)	Warfarin (n=230,405)	St. Diff
**Patient Characteristics**						
Mean Age (SD)	82.83 (6.89)	82.86 (6.90)	-0.01	77.67 (7.3)	77.67 (7.19)	0.00
Male	8,164 (40.5%)	8,170 (40.5%)	0.00	116,410 (50.5%)	116,429 (50.5%)	0.00
Female	12,011 (59.5%)	12,005 (59.5%)	0.00	113,995 (49.5%)	113,976 (49.5%)	0.00
Black	1,409 (7.5%)	1,405 (7.5%)	0.00	9,420 (4.5%)	9,427 (4.5%)	0.00
White	16,239 (86.3%)	16,291 (86.6%)	-0.01	189,685 (89.7%)	189,596 (89.7%)	0.00
Other[A]	1,174 (6.2%)	1,126 (6.0%)	0.01	12,275 (5.8%)	12,357 (5.8%)	0.00
Dual Status[B]	3,056 (18.9%)	3,044 (18.8%)	0.00	8,343 (4.7%)	8,294 (4.7%)	0.00
Mean CHA2DS2-VASc score (SD)	5.79 (1.60)	5.80 (1.60)	-0.01	4.62 (1.7)	4.61 (1.64)	0.01
Mean HAS-BLED score (SD)	2.69 (0.79)	2.70 (0.78)	0.00	2.27 (0.7)	2.27 (0.72)	0.00
Mean CCI score (SD)	6.33 (3.08)	6.31 (3.04)	0.01	3.23 (2.7)	3.23 (2.67)	0.00
Mean CFI (SD)	0.32 (0.08)	0.32 (0.07)	0.00	0.20 (0.1)	0.20 (0.06)	0.00
**Cardiovascular conditions**						
Acute Myocardial infarction	1,978 (9.8%)	1,993 (9.9%)	0.00	15,227 (6.6%)	15,211 (6.6%)	0.00
Cardio-ablation	91 (0.5%)	83 (0.4%)	0.01	1,213 (0.5%)	1,187 (0.5%)	0.00
Cardioversion	483 (2.4%)	462 (2.3%)	0.01	10,128 (4.4%)	9,946 (4.3%)	0.00
Cerebrovascular disease	10,194 (50.5%)	10,191 (50.5%)	0.00	59,926 (26.0%)	59,890 (26.0%)	0.00
Congestive heart failure (inpatient)	7,185 (35.6%)	7,169 (35.5%)	0.00	44,406 (19.3%)	44,040 (19.1%)	0.00
Congestive heart failure (outpatient)	9,658 (47.9%)	9,676 (48.0%)	0.00	76,823 (33.3%)	76,480 (33.2%)	0.00
Coronary revascularization	581 (2.9%)	593 (2.9%)	0.00	10,929 (4.7%)	10,861 (4.7%)	0.00
Hypertension	18,601 (92.2%)	18,621 (92.3%)	0.00	197,170 (85.6%)	196,735 (85.4%)	0.01
Ischemic Heart Disease	10,858 (53.8%)	10,869 (53.9%)	0.00	106,075 (46.0%)	105,548 (45.8%)	0.00
PVD or PVD Surgery	5,022 (24.9%)	5,065 (25.1%)	0.00	32,300 (14.0%)	32,124 (13.9%)	0.00
Stroke (inpatient)	4,293 (21.3%)	4,329 (21.5%)	0.00	18,379 (8.0%)	18,335 (8.0%)	0.00
Stroke (outpatient)	4,240 (21.0%)	4,280 (21.2%)	0.00	26,942 (11.7%)	26,970 (11.7%)	0.00
Syncope	3,635 (18.0%)	3,657 (18.1%)	0.00	21,223 (9.2%)	21,186 (9.2%)	0.00
**Non-cardiovascular conditions**						
Acute renal failure	5,534 (27.4%)	5,529 (27.4%)	0.00	30,521 (13.2%)	30,346 (13.2%)	0.00
Alcohol abuse or dependence	583 (2.9%)	576 (2.9%)	0.00	4,222 (1.8%)	4,205 (1.8%)	0.00
Anemia	9,063 (44.9%)	9,056 (44.9%)	0.00	62,864 (27.3%)	62,744 (27.2%)	0.00
CKD: stage 3, 4 or unspecified	5,854 (29.0%)	5,783 (28.7%)	0.01	41,785 (18.1%)	41,623 (18.1%)	0.00
COPD	6,134 (30.4%)	6,127 (30.4%)	0.00	51,255 (22.2%)	51,029 (22.1%)	0.00
Dementia	20,175 (100.0%)	20,175 (100.0%)		0 (0.0%)	0 (0.0%)	
Diabetes	8,657 (42.9%)	8,647 (42.9%)	0.00	85,676 (37.2%)	85,671 (37.2%)	0.00
Endoscopy	833 (4.1%)	876 (4.3%)	-0.01	4,857 (2.1%)	4,846 (2.1%)	0.00
Falls	3,673 (18.2%)	3,683 (18.3%)	0.00	11,460 (5.0%)	11,388 (4.9%)	0.00
Fractures	3,744 (18.6%)	3,749 (18.6%)	0.00	18,685 (8.1%)	18,956 (8.2%)	0.00
GI bleeding (inpatient)	2,291 (11.4%)	2,278 (11.3%)	0.00	12,509 (5.4%)	12,432 (5.4%)	0.00
GI bleeding (outpatient)	2,932 (14.5%)	2,903 (14.4%)	0.00	30,695 (13.3%)	30,533 (13.3%)	0.00
Liver disease	1,452 (7.2%)	1,455 (7.2%)	0.00	14,070 (6.1%)	14,079 (6.1%)	0.00
Malignancy	3,206 (15.9%)	3,184 (15.8%)	0.00	42,442 (18.4%)	42,550 (18.5%)	0.00
Obesity	3,046 (15.1%)	3,027 (15.0%)	0.00	45,784 (19.9%)	45,816 (19.9%)	0.00
Peptic Ulcer	670 (3.3%)	656 (3.3%)	0.00	4,927 (2.1%)	4,953 (2.1%)	0.00
Smoking	6,684 (33.1%)	6,677 (33.1%)	0.00	70,570 (30.6%)	70,251 (30.5%)	0.00
**Cardiovascular medications**						
ACE inhibitors	6,251 (31.0%)	6,221 (30.8%)	0.00	66,384 (28.8%)	65,946 (28.6%)	0.00
Angiotensin II receptor blockers	1,033 (5.1%)	1,011 (5.0%)	0.00	12,571 (5.5%)	12,674 (5.5%)	0.00
Antiarrhythmic agents	2,821 (14.0%)	2,882 (14.3%)	-0.01	44,202 (19.2%)	43,731 (19.0%)	0.01
Anticoagulants, injectable	388 (1.9%)	410 (2.0%)	-0.01	2,250 (1.0%)	2,306 (1.0%)	0.00
Antiplatelet agent	3,638 (18.0%)	3,656 (18.1%)	0.00	33,445 (14.5%)	33,128 (14.4%)	0.00
Beta-blockers	13,619 (67.5%)	13,612 (67.5%)	0.00	157,609 (68.4%)	157,446 (68.3%)	0.00
Calcium channel blockers	420 (2.1%)	418 (2.1%)	0.00	3,969 (1.7%)	4,010 (1.7%)	0.00
Diuretics	11,707 (58.0%)	11,697 (58.0%)	0.00	131,931 (57.3%)	131,927 (57.3%)	0.00
Fibrates	695 (3.4%)	699 (3.5%)	0.00	10,799 (4.7%)	10,825 (4.7%)	0.00
Nitrates	2,976 (14.8%)	3,006 (14.9%)	0.00	29,999 (13.0%)	29,800 (12.9%)	0.00

© 2023 Lin KJ et al. *JAMA Network Open*.

Characteristics Number of Patients (%)	With Dementia Apixaban (n=20,175)	With Dementia Warfarin (n=20,175)	St. Diff	Without Dementia Apixaban (n=230,405)	Without Dementia Warfarin (n=230,405)	St. Diff
Statins	13,394 (66.4%)	13,354 (66.2%)	0.00	150,374 (65.3%)	150,439 (65.3%)	0.00
**Other medications**						
Anticonvulsants	4,751 (23.5%)	4,734 (23.5%)	0.00	36,422 (15.8%)	36,480 (15.8%)	0.00
Antidepressants - Other	4,106 (20.4%)	4,069 (20.2%)	0.00	17,087 (7.4%)	16,889 (7.3%)	0.00
Antidepressants - SSRI/SNRI	7,866 (39.0%)	7,874 (39.0%)	0.00	42,727 (18.5%)	42,847 (18.6%)	0.00
Antidepressants - Tricyclics	598 (3.0%)	602 (3.0%)	0.00	6,130 (2.7%)	6,156 (2.7%)	0.00
Antipsychotic agents	3,334 (16.5%)	3,321 (16.5%)	0.00	3,789 (1.6%)	3,799 (1.6%)	0.00
Anxiolytics (except benzodiazepine)	486 (2.4%)	506 (2.5%)	-0.01	2,360 (1.0%)	2,324 (1.0%)	0.00
Anxiolytics - Benzodiazepines	4,637 (23.0%)	4,710 (23.3%)	-0.01	38,281 (16.6%)	38,188 (16.6%)	0.00
Bronchodilators	4,404 (21.8%)	4,378 (21.7%)	0.00	43,260 (18.8%)	43,287 (18.8%)	0.00
Corticosteroids, inhaled	3,931 (19.5%)	3,931 (19.5%)	0.00	48,655 (21.1%)	48,564 (21.1%)	0.00
Corticosteroids, oral	5,577 (27.6%)	5,621 (27.9%)	0.00	72,366 (31.4%)	72,280 (31.4%)	0.00
Dementia drugs	8,755 (43.4%)	8,697 (43.1%)	0.01	3,585 (1.6%)	3,645 (1.6%)	0.00
Diabetes agents - Insulin	2,450 (12.1%)	2,383 (11.8%)	0.01	17,884 (7.8%)	17,926 (7.8%)	0.00
Diabetes agents - Metformin	2,909 (14.4%)	2,910 (14.4%)	0.00	37,547 (16.3%)	37,710 (16.4%)	0.00
Diabetes agents - Other	1,174 (5.8%)	1,217 (6.0%)	-0.01	12,103 (5.3%)	12,246 (5.3%)	0.00
Diabetes agents - Sulfonylurea	2,032 (10.1%)	2,055 (10.2%)	0.00	23,507 (10.2%)	23,512 (10.2%)	0.00
Estrogen	425 (2.1%)	417 (2.1%)	0.00	6,294 (2.7%)	6,382 (2.8%)	0.00
GI - H2 blockers	2,348 (11.6%)	2,348 (11.6%)	0.00	16,630 (7.2%)	16,789 (7.3%)	0.00
GI - Proton-pump inhibitors	7,592 (37.6%)	7,628 (37.8%)	0.00	72,252 (31.4%)	72,032 (31.3%)	0.00
GI - Sucralfate	476 (2.4%)	491 (2.4%)	0.00	4,079 (1.8%)	4,132 (1.8%)	0.00
Hypnotics	1,789 (8.9%)	1,852 (9.2%)	-0.01	17,406 (7.6%)	17,348 (7.5%)	0.00
NSAIDs	2,645 (13.1%)	2,621 (13.0%)	0.00	32,437 (14.1%)	32,238 (14.0%)	0.00
Opioids	7,975 (39.5%)	8,097 (40.1%)	-0.01	88,738 (38.5%)	88,487 (38.4%)	0.00
Parkinsonism drugs	1,602 (7.9%)	1,609 (8.0%)	0.00	7,978 (3.5%)	7,948 (3.4%)	0.00
Thyroid hormone replacement	5,384 (26.7%)	5,410 (26.8%)	0.00	51,095 (22.2%)	51,227 (22.2%)	0.00
**Health care utilization**						
Emergency department visits	11,681 (57.9%)	11,652 (57.8%)	0.00	92,961 (40.3%)	92,968 (40.3%)	0.00
Home Health Day[C]	1,514 (9.3%)	1,524 (9.4%)	0.00	5,582 (3.2%)	5,657 (3.2%)	0.00
Home oxygen use	1,041 (5.2%)	1,057 (5.2%)	0.00	7,309 (3.2%)	7,259 (3.2%)	0.00
Hospitalizations	15,645 (77.5%)	15,628 (77.5%)	0.00	107,269 (46.6%)	106,874 (46.4%)	0.00
**Geographic Region**						
Northeast	4,450 (22.1%)	4,505 (22.3%)	-0.01	46,139 (20.0%)	46,402 (20.1%)	0.00
Midwest	5,163 (25.6%)	5,139 (25.5%)	0.00	60,713 (26.4%)	60,396 (26.2%)	0.00
South	7,436 (36.9%)	7,386 (36.6%)	0.01	79,798 (34.6%)	79,613 (34.6%)	0.00
West	3,104 (15.4%)	3,122 (15.5%)	0.00	43,518 (18.9%)	43,738 (19.0%)	0.00
Other	22 (0.1%)	23 (0.1%)	0.00	237 (0.1%)	256 (0.1%)	0.00

Abbreviations: ACE, angiotensin converting enzyme; CCI, combined comorbidity; CFI, claims-based frailty index; CKD, chronic kidney disease; COPD, chronic obstructive pulmonary disease; GI, gastrointestinal; NSAID, non-steroidal anti-inflammatory drug; PVD, peripheral vascular disease; SNRI, serotonin-norepinephrine reuptake inhibitor; SSRI, selective serotonin reuptake inhibitor.

[A] Other race category includes Unknown, Other, Asian, Hispanic, North American Native, and Missing Race Categories in Medicare. Asian, Hispanic, Unknown, and Missing categories are included for race in Optum. Race is unavailable in the MarketScan database.

[B] With both Medicare are Medicaid enrollment eligibility

[C] Home Health services received (days)

© 2023 Lin KJ et al. *JAMA Network Open*.

eTable 17. Selected Characteristics of Propensity Score–Matched Population With Atrial Fibrillation Treated With Warfarin vs Apixaban by Dementia Diagnosis in the Medicare Population

Characteristics Number of Patients (%)	With Dementia			Without Dementia		
	Apixaban (n=16,196)	Warfarin (n=16,196)	St. Diff	Apixaban (n=176,814)	Warfarin (n=176,814)	St. Diff
**Quarter/Year of Cohort Entry Date**						
Q1 2013	20 (0.1%)	20 (0.1%)	0.00	223 (0.1%)	256 (0.1%)	0.00
Q2 2013	126 (0.8%)	126 (0.8%)	0.00	1,787 (1.0%)	1,831 (1.0%)	0.00
Q3 2013	168 (1.0%)	170 (1.0%)	0.00	2,621 (1.5%)	2,577 (1.5%)	0.00
Q4 2013	305 (1.9%)	296 (1.8%)	0.01	3,978 (2.2%)	3,776 (2.1%)	0.01
Q1 2014	511 (3.2%)	505 (3.1%)	0.01	6,340 (3.6%)	6,372 (3.6%)	0.00
Q2 2014	705 (4.4%)	678 (4.2%)	0.01	8,235 (4.7%)	8,126 (4.6%)	0.00
Q3 2014	755 (4.7%)	771 (4.8%)	0.00	8,663 (4.9%)	8,601 (4.9%)	0.00
Q4 2014	959 (5.9%)	952 (5.9%)	0.00	10,208 (5.8%)	10,013 (5.7%)	0.00
Q1 2015	1,125 (6.9%)	1,097 (6.8%)	0.00	12,277 (6.9%)	12,264 (6.9%)	0.00
Q2 2015	1,141 (7.0%)	1,129 (7.0%)	0.00	12,307 (7.0%)	12,369 (7.0%)	0.00
Q3 2015	1,110 (6.9%)	1,134 (7.0%)	0.00	11,680 (6.6%)	11,707 (6.6%)	0.00
Q4 2015	1,187 (7.3%)	1,215 (7.5%)	-0.01	12,204 (6.9%)	12,125 (6.9%)	0.00
Q1 2016	1,259 (7.8%)	1,271 (7.8%)	0.00	13,338 (7.5%)	13,313 (7.5%)	0.00
Q2 2016	1,251 (7.7%)	1,246 (7.7%)	0.00	12,072 (6.8%)	12,050 (6.8%)	0.00
Q3 2016	1,039 (6.4%)	1,061 (6.6%)	-0.01	10,979 (6.2%)	11,066 (6.3%)	0.00
Q4 2016	988 (6.1%)	992 (6.1%)	0.00	10,849 (6.1%)	11,015 (6.2%)	0.00
Q1 2017	989 (6.1%)	990 (6.1%)	0.00	10,856 (6.1%)	10,991 (6.2%)	0.00
Q2 2017	875 (5.4%)	879 (5.4%)	0.00	9,858 (5.6%)	9,940 (5.6%)	0.00
Q3 2017	878 (5.4%)	870 (5.4%)	0.00	9,302 (5.3%)	9,317 (5.3%)	0.00
Q4 2017	805 (5.0%)	794 (4.9%)	0.00	9,037 (5.1%)	9,105 (5.1%)	0.00
**Patient Characteristics**						
Mean age (SD)	82.94 (7.07)	82.98 (7.08)	-0.01	77.74 (7.35)	77.75 (7.27)	0.00
Male	6,354 (39.2%)	6,344 (39.2%)	0.00	87,210 (49.3%)	87,173 (49.3%)	0.00
Female	9,842 (60.8%)	9,852 (60.8%)	0.00	89,604 (50.7%)	89,641 (50.7%)	0.00
White	14,317 (88.4%)	14,354 (88.6%)	-0.01	162,940 (92.2%)	162,779 (92.1%)	0.00
Black	1,140 (7.0%)	1,141 (7.0%)	0.00	6,734 (3.8%)	6,809 (3.9%)	-0.01
Other[A]	739 (4.6%)	701 (4.3%)	0.01	7,140 (4.0%)	7,226 (4.1%)	-0.01
Dual Status[B]	3,056 (18.9%)	3,044 (18.8%)	0.00	8,343 (4.7%)	8,294 (4.7%)	0.00
Mean CHA_2DS_2-VASc score (SD)	5.88 (1.61)	5.89 (1.61)	-0.01	4.68 (1.71)	4.67 (1.66)	0.01
Mean HAS-BLED score (SD)	2.71 (0.79)	2.71 (0.78)	0.00	2.27 (0.73)	2.27 (0.73)	0.00
Mean CCI score (SD)	6.22 (3.05)	6.21 (3.02)	0.00	3.15 (2.67)	3.15 (2.64)	0.00
Mean CFI (SD)	0.32 (0.08)	0.32 (0.07)	0.00	0.20 (0.06)	0.20 (0.06)	0.00
**Medical History**						
Acute renal failure	4,522 (27.9%)	4,524 (27.9%)	0.00	23,627 (13.4%)	23,429 (13.3%)	0.00
Acute myocardial infarction	1,593 (9.8%)	1,604 (9.9%)	0.00	11,621 (6.6%)	11,477 (6.5%)	0.00
Alcohol abuse or dependence	470 (2.9%)	457 (2.8%)	0.01	3,048 (1.7%)	3,021 (1.7%)	0.00
Anemia	7,563 (46.7%)	7,546 (46.6%)	0.00	49,935 (28.2%)	49,891 (28.2%)	0.00
Cardio-ablation	77 (0.5%)	69 (0.4%)	0.01	1,003 (0.6%)	964 (0.5%)	0.01
Cardioversion	381 (2.4%)	359 (2.2%)	0.01	7,546 (4.3%)	7,356 (4.2%)	0.00
Cerebrovascular disease	8,302 (51.3%)	8,301 (51.3%)	0.00	47,175 (26.7%)	47,102 (26.6%)	0.00
CKD: stage 3, 4 or unspecified	4,601 (28.4%)	4,544 (28.1%)	0.01	29,972 (17.0%)	29,965 (16.9%)	0.00
Congestive heart failure (inpatient)	6,118 (37.8%)	6,112 (37.7%)	0.00	35,601 (20.1%)	35,239 (19.9%)	0.01
Congestive heart failure (outpatient)	7,725 (47.7%)	7,726 (47.7%)	0.00	57,238 (32.4%)	57,043 (32.3%)	0.00
Coronary revascularization	480 (3.0%)	492 (3.0%)	0.00	8,431 (4.8%)	8,344 (4.7%)	0.00
COPD	4,996 (30.8%)	5,000 (30.9%)	0.00	39,109 (22.1%)	38,940 (22.0%)	0.00
Dementia	16,196 (100.0%)	16,196 (100.0%)	-	0 (0.0%)	0 (0.0%)	-
Diabetes	7,051 (43.5%)	7,051 (43.5%)	0.00	65,609 (37.1%)	65,670 (37.1%)	0.00
Endoscopy	719 (4.4%)	758 (4.7%)	-0.01	3,963 (2.2%)	3,968 (2.2%)	0.00
Falls	2,485 (15.3%)	2,473 (15.3%)	0.00	6,433 (3.6%)	6,377 (3.6%)	0.00
Fractures	3,036 (18.7%)	3,043 (18.8%)	0.00	14,532 (8.2%)	14,724 (8.3%)	0.00
GI bleeding events (inpatient)	2,016 (12.4%)	2,008 (12.4%)	0.00	10,722 (6.1%)	10,723 (6.1%)	0.00
GI bleeding events (outpatient)	1,952 (12.1%)	1,930 (11.9%)	0.01	20,075 (11.4%)	20,017 (11.3%)	0.00
Hypertension	14,947 (92.3%)	14,959 (92.4%)	0.00	150,419 (85.1%)	150,122 (84.9%)	0.01
Ischemic Heart Disease	8,777 (54.2%)	8,763 (54.1%)	0.00	81,534 (46.1%)	81,138 (45.9%)	0.00
Liver disease	1,173 (7.2%)	1,187 (7.3%)	0.00	10,678 (6.0%)	10,674 (6.0%)	0.00
Malignancy	2,566 (15.8%)	2,544 (15.7%)	0.00	33,196 (18.8%)	33,258 (18.8%)	0.00
Obesity	2,555 (15.8%)	2,548 (15.7%)	0.00	35,922 (20.3%)	35,843 (20.3%)	0.00
Peptic ulcer	547 (3.4%)	531 (3.3%)	0.01	3,911 (2.2%)	3,921 (2.2%)	0.00
PVD or PVD Surgery	4,017 (24.8%)	4,052 (25.0%)	0.00	24,522 (13.9%)	24,279 (13.7%)	0.01
Smoking	5,785 (35.7%)	5,758 (35.6%)	0.00	58,615 (33.2%)	58,245 (32.9%)	0.01

© 2023 Lin KJ et al. *JAMA Network Open*.

Characteristics<br>Number of Patients (%)	With Dementia			Without Dementia		
	Apixaban (n=16,196)	Warfarin (n=16,196)	St. Diff	Apixaban (n=176,814)	Warfarin (n=176,814)	St. Diff
Stroke (inpatient)	3,546 (21.9%)	3,571 (22.0%)	0.00	14,777 (8.4%)	14,664 (8.3%)	0.00
Stroke (outpatient)	2,543 (15.7%)	2,571 (15.9%)	-0.01	15,458 (8.7%)	15,470 (8.7%)	0.00
Syncope	2,853 (17.6%)	2,893 (17.9%)	-0.01	16,206 (9.2%)	16,150 (9.1%)	0.00
**Medications**						
ACE inhibitors	5,058 (31.2%)	5,042 (31.1%)	0.00	51,071 (28.9%)	50,714 (28.7%)	0.00
Angiotensin II receptor blockers	862 (5.3%)	835 (5.2%)	0.00	9,926 (5.6%)	9,998 (5.7%)	0.00
Antiarrhythmic agents	2,334 (14.4%)	2,400 (14.8%)	-0.01	34,842 (19.7%)	34,482 (19.5%)	0.01
Anticoagulants, injectable	299 (1.8%)	308 (1.9%)	-0.01	1,555 (0.9%)	1,587 (0.9%)	0.00
Anticonvulsants	3,927 (24.2%)	3,901 (24.1%)	0.00	28,371 (16.0%)	28,449 (16.1%)	0.00
Antidepressants - Other	3,398 (21.0%)	3,331 (20.6%)	0.01	13,138 (7.4%)	13,036 (7.4%)	0.00
Antidepressants - SSRI/SNRI	6,462 (39.9%)	6,460 (39.9%)	0.00	33,634 (19.0%)	33,736 (19.1%)	0.00
Antidepressants - Tricyclics	517 (3.2%)	521 (3.2%)	0.00	4,901 (2.8%)	4,937 (2.8%)	0.00
Antiplatelet agents	3,027 (18.7%)	3,043 (18.8%)	0.00	26,157 (14.8%)	25,854 (14.6%)	0.01
Antipsychotic agents	2,819 (17.4%)	2,780 (17.2%)	0.01	3,104 (1.8%)	3,126 (1.8%)	0.00
Anxiolytics - Benzodiazepines	3,932 (24.3%)	3,956 (24.4%)	0.00	30,593 (17.3%)	30,543 (17.3%)	0.00
Anxiolytics - Other	410 (2.5%)	420 (2.6%)	-0.01	1,849 (1.0%)	1,826 (1.0%)	0.00
Beta-blockers	11,012 (68.0%)	10,995 (67.9%)	0.00	121,785 (68.9%)	121,592 (68.8%)	0.00
Bronchodilators	3,642 (22.5%)	3,590 (22.2%)	0.01	33,394 (18.9%)	33,420 (18.9%)	0.00
Calcium channel blockers	418 (2.6%)	416 (2.6%)	0.00	3,910 (2.2%)	3,951 (2.2%)	0.00
Corticosteroids, inhaled	3,285 (20.3%)	3,264 (20.2%)	0.00	38,462 (21.8%)	38,348 (21.7%)	0.00
Corticosteroids, oral	4,643 (28.7%)	4,650 (28.7%)	0.00	57,195 (32.3%)	57,121 (32.3%)	0.00
Dementia drugs	7,044 (43.5%)	7,008 (43.3%)	0.00	2,893 (1.6%)	2,939 (1.7%)	-0.01
Diabetes agents - Insulin	2,036 (12.6%)	1,992 (12.3%)	0.01	13,867 (7.8%)	13,876 (7.8%)	0.00
Diabetes agents - Metformin	2,326 (14.4%)	2,359 (14.6%)	-0.01	28,525 (16.1%)	28,632 (16.2%)	0.00
Diabetes agents - Other	972 (6.0%)	1,012 (6.2%)	-0.01	9,359 (5.3%)	9,462 (5.4%)	0.00
Diabetes agents - Sulfonylurea	1,655 (10.2%)	1,680 (10.4%)	-0.01	17,958 (10.2%)	17,951 (10.2%)	0.00
Diuretics	9,595 (59.2%)	9,601 (59.3%)	0.00	102,526 (58.0%)	102,563 (58.0%)	0.00
Estrogen	355 (2.2%)	349 (2.2%)	0.00	5,122 (2.9%)	5,186 (2.9%)	0.00
Fibrates	576 (3.6%)	565 (3.5%)	0.01	8,364 (4.7%)	8,395 (4.7%)	0.00
GI - H2 blockers	1,961 (12.1%)	1,987 (12.3%)	-0.01	13,286 (7.5%)	13,389 (7.6%)	0.00
GI - Proton-pump inhibitors	6,311 (39.0%)	6,310 (39.0%)	0.00	57,052 (32.3%)	56,916 (32.2%)	0.00
GI - Sucralfate	404 (2.5%)	411 (2.5%)	0.00	3,299 (1.9%)	3,333 (1.9%)	0.00
Hypnotics	1,520 (9.4%)	1,569 (9.7%)	-0.01	14,143 (8.0%)	14,079 (8.0%)	0.00
Nitrates	2,472 (15.3%)	2,519 (15.6%)	-0.01	23,779 (13.4%)	23,648 (13.4%)	0.00
NSAIDs	2,219 (13.7%)	2,178 (13.4%)	0.01	25,687 (14.5%)	25,453 (14.4%)	0.00
Opioids	6,678 (41.2%)	6,720 (41.5%)	-0.01	69,528 (39.3%)	69,342 (39.2%)	0.00
Parkinsonism drugs	1,318 (8.1%)	1,319 (8.1%)	0.00	6,335 (3.6%)	6,307 (3.6%)	0.00
Statins	10,867 (67.1%)	10,841 (66.9%)	0.00	116,473 (65.9%)	116,492 (65.9%)	0.00
Thyroid hormone replacement	4,397 (27.1%)	4,430 (27.4%)	-0.01	39,975 (22.6%)	40,179 (22.7%)	0.00
**Health care use**						
Emergency department visits	8,778 (54.2%)	8,730 (53.9%)	0.01	66,945 (37.9%)	66,909 (37.8%)	0.00
Home health day[C]	1,514 (9.3%)	1,524 (9.4%)	0.00	5,582 (3.2%)	5,657 (3.2%)	0.00
Home oxygen use	841 (5.2%)	855 (5.3%)	0.00	5,678 (3.2%)	5,605 (3.2%)	0.00
Hospitalizations	12,887 (79.6%)	12,860 (79.4%)	0.00	85,307 (48.2%)	84,773 (47.9%)	0.01
**Geographic Region**						
Northeast	3,745 (23.1%)	3,778 (23.3%)	0.00	37,000 (20.9%)	37,211 (21.0%)	0.00
Midwest	3,973 (24.5%)	3,991 (24.6%)	0.00	46,055 (26.0%)	45,852 (25.9%)	0.00
South	6,167 (38.1%)	6,114 (37.8%)	0.01	63,330 (35.8%)	63,162 (35.7%)	0.00
West	2,293 (14.2%)	2,294 (14.2%)	0.00	30,274 (17.1%)	30,428 (17.2%)	0.00
Other	18 (0.1%)	19 (0.1%)	0.00	155 (0.1%)	161 (0.1%)	0.00

Abbreviations: ACE, angiotensin converting enzyme; CCI, combined comorbidity; CFI, claims-based frailty index; CKD, chronic kidney disease; COPD, chronic obstructive pulmonary disease; GI, gastrointestinal; NSAID, non-steroidal anti-inflammatory drug; PVD, peripheral vascular disease; SNRI, serotonin-norepinephrine reuptake inhibitor; SSRI, selective serotonin reuptake inhibitor.

[A] Other race category includes Unknown, Other, Asian, Hispanic, North American Native, and Missing Race Categories in Medicare. Asian, Hispanic, Unknown, and Missing categories are included for race in Optum. Race is unavailable in the MarketScan database.

[B] With both Medicare are Medicaid enrollment eligibility

[C] Home Health services received (days)

© 2023 Lin KJ et al. *JAMA Network Open*.

eTable 18. Selected Characteristics of Propensity Score–Matched Population With Atrial Fibrillation Treated With Warfarin vs Apixaban by Dementia Diagnosis in the Optum Population

Characteristics<br>Number of Patients (%)	With Dementia			Without Dementia		
	Apixaban (n=2,626)	Warfarin (n=2,626)	St. Diff	Apixaban (n=34,566)	Warfarin (n=34,566)	St. Diff
**Quarter/Year of Cohort Entry Date**						
Q1 2013	0 (0.0%)	0 (0.0%)	-	36 (0.1%)	36 (0.1%)	0.00
Q2 2013	10 (0.4%)	7 (0.3%)	0.02	182 (0.5%)	212 (0.6%)	-0.01
Q3 2013	12 (0.5%)	15 (0.6%)	-0.01	236 (0.7%)	227 (0.7%)	0.00
Q4 2013	17 (0.6%)	14 (0.5%)	0.01	414 (1.2%)	453 (1.3%)	-0.01
Q1 2014	35 (1.3%)	40 (1.5%)	-0.02	631 (1.8%)	601 (1.7%)	0.01
Q2 2014	54 (2.1%)	57 (2.2%)	-0.01	735 (2.1%)	776 (2.2%)	-0.01
Q3 2014	46 (1.8%)	39 (1.5%)	0.02	822 (2.4%)	828 (2.4%)	0.00
Q4 2014	63 (2.4%)	63 (2.4%)	0.00	938 (2.7%)	940 (2.7%)	0.00
Q1 2015	96 (3.7%)	96 (3.7%)	0.00	1,215 (3.5%)	1,250 (3.6%)	-0.01
Q2 2015	80 (3.0%)	89 (3.4%)	-0.02	1,228 (3.6%)	1,202 (3.5%)	0.01
Q3 2015	96 (3.7%)	102 (3.9%)	-0.01	1,229 (3.6%)	1,237 (3.6%)	0.00
Q4 2015	90 (3.4%)	91 (3.5%)	-0.01	1,268 (3.7%)	1,288 (3.7%)	0.00
Q1 2016	139 (5.3%)	137 (5.2%)	0.00	1,717 (5.0%)	1,711 (4.9%)	0.00
Q2 2016	111 (4.2%)	131 (5.0%)	-0.04	1,443 (4.2%)	1,413 (4.1%)	0.01
Q3 2016	105 (4.0%)	112 (4.3%)	-0.02	1,327 (3.8%)	1,321 (3.8%)	0.00
Q4 2016	118 (4.5%)	110 (4.2%)	0.01	1,361 (3.9%)	1,331 (3.9%)	0.00
Q1 2017	135 (5.1%)	138 (5.3%)	-0.01	1,952 (5.6%)	1,917 (5.5%)	0.00
Q2 2017	119 (4.5%)	107 (4.1%)	0.02	1,529 (4.4%)	1,494 (4.3%)	0.00
Q3 2017	120 (4.6%)	109 (4.2%)	0.02	1,334 (3.9%)	1,308 (3.8%)	0.01
Q4 2017	97 (3.7%)	96 (3.7%)	0.00	1,296 (3.7%)	1,292 (3.7%)	0.00
Q1 2018	112 (4.3%)	118 (4.5%)	-0.01	1,370 (4.0%)	1,432 (4.1%)	-0.01
Q2 2018	99 (3.8%)	94 (3.6%)	0.01	1,185 (3.4%)	1,166 (3.4%)	0.00
Q3 2018	97 (3.7%)	76 (2.9%)	0.04	1,059 (3.1%)	1,027 (3.0%)	0.01
Q4 2018	85 (3.2%)	77 (2.9%)	0.02	1,083 (3.1%)	1,050 (3.0%)	0.01
Q1 2019	104 (4.0%)	95 (3.6%)	0.02	1,109 (3.2%)	1,091 (3.2%)	0.00
Q2 2019	69 (2.6%)	64 (2.4%)	0.01	836 (2.4%)	895 (2.6%)	-0.01
Q3 2019	65 (2.5%)	72 (2.7%)	-0.01	902 (2.6%)	893 (2.6%)	0.00
Q4 2019	54 (2.1%)	63 (2.4%)	-0.02	767 (2.2%)	783 (2.3%)	-0.01
Q1 2020	50 (1.9%)	56 (2.1%)	-0.01	936 (2.7%)	911 (2.6%)	0.01
Q2 2020	41 (1.6%)	45 (1.7%)	-0.01	608 (1.8%)	600 (1.7%)	0.01
Q3 2020	58 (2.2%)	53 (2.0%)	0.01	624 (1.8%)	653 (1.9%)	-0.01
Q4 2020	56 (2.1%)	49 (1.9%)	0.01	577 (1.7%)	595 (1.7%)	0.00
Q1 2021	142 (5.4%)	155 (5.9%)	-0.02	1,737 (5.0%)	1,735 (5.0%)	0.00
Q2 2021	51 (1.9%)	56 (2.1%)	-0.01	880 (2.5%)	898 (2.6%)	-0.01
**Patient Characteristics**						
Mean age (SD)	81.79 (5.85)	81.80 (5.86)	0.00	77.11 (6.61)	77.06 (6.57)	0.01
Male	1,180 (44.9%)	1,180 (44.9%)	0.00	18,694 (54.1%)	18,695 (54.1%)	0.00
Female	1,446 (55.1%)	1,446 (55.1%)	0.00	15,872 (45.9%)	15,871 (45.9%)	0.00
Black	269 (10.2%)	264 (10.1%)	0.00	2,686 (7.8%)	2,618 (7.6%)	0.01
White	1,922 (73.2%)	1,937 (73.8%)	-0.01	26,745 (77.4%)	26,817 (77.6%)	0.00
Other[A]	435 (16.6%)	425 (16.2%)	0.01	5,135 (14.9%)	5,131 (14.8%)	0.00
Mean CHA2DS2-VASc score (SD)	5.74 (1.60)	5.77 (1.61)	-0.02	4.70 (1.66)	4.69 (1.62)	0.01
Mean HAS-BLED score (SD)	2.67 (0.81)	2.69 (0.80)	-0.02	2.30 (0.71)	2.29 (0.71)	0.01
Mean CCI score (SD)	7.11 (3.23)	7.10 (3.13)	0.00	3.71 (2.90)	3.71 (2.88)	0.00
Mean CFI (SD)	0.30 (0.07)	0.30 (0.07)	0.00	0.19 (0.06)	0.19 (0.06)	0.00
**Medical History**						
Acute myocardial infarction	251 (9.6%)	254 (9.7%)	0.00	2,287 (6.6%)	2,393 (6.9%)	-0.01
Acute renal failure	692 (26.4%)	691 (26.3%)	0.00	4,685 (13.6%)	4,753 (13.8%)	-0.01
Alcohol abuse or dependence	88 (3.4%)	84 (3.2%)	0.01	893 (2.6%)	902 (2.6%)	0.00
Anemia	1,044 (39.8%)	1,066 (40.6%)	-0.02	8,762 (25.3%)	8,716 (25.2%)	0.00
Cardio-ablation	13 (0.5%)	12 (0.5%)	0.00	130 (0.4%)	138 (0.4%)	0.00
Cardioversion	68 (2.6%)	64 (2.4%)	0.01	1,603 (4.6%)	1,603 (4.6%)	0.00
Cerebrovascular disease	1,227 (46.7%)	1,240 (47.2%)	-0.01	8,128 (23.5%)	8,134 (23.5%)	0.00
CKD: stage 3, 4 or unspecified	923 (35.1%)	922 (35.1%)	0.00	8,554 (24.7%)	8,482 (24.5%)	0.00
Congestive heart failure (inpatient)	630 (24.0%)	633 (24.1%)	0.00	5,062 (14.6%)	5,136 (14.9%)	-0.01

© 2023 Lin KJ et al. *JAMA Network Open*.

Characteristics<br>Number of Patients (%)	With Dementia			Without Dementia		
	Apixaban (n=2,626)	Warfarin (n=2,626)	St. Diff	Apixaban (n=34,566)	Warfarin (n=34,566)	St. Diff
Congestive heart failure (outpatient)	1,343 (51.1%)	1,369 (52.1%)	-0.02	13,576 (39.3%)	13,494 (39.0%)	0.01
Coronary revascularization	73 (2.8%)	68 (2.6%)	0.01	1,624 (4.7%)	1,623 (4.7%)	0.00
COPD	760 (28.9%)	772 (29.4%)	-0.01	8,219 (23.8%)	8,157 (23.6%)	0.00
Dementia	2,626 (100.0%)	2,626 (100.0%)	-	0 (0.0%)	0 (0.0%)	-
Diabetes	1,090 (41.5%)	1,095 (41.7%)	0.00	13,435 (38.9%)	13,433 (38.9%)	0.00
Endoscopy	81 (3.1%)	87 (3.3%)	-0.01	613 (1.8%)	606 (1.8%)	0.00
Falls	877 (33.4%)	878 (33.4%)	0.00	3,771 (10.9%)	3,765 (10.9%)	0.00
Fractures	462 (17.6%)	461 (17.6%)	0.00	2,686 (7.8%)	2,692 (7.8%)	0.00
GI bleeding events (inpatient)	170 (6.5%)	172 (6.5%)	0.00	975 (2.8%)	951 (2.8%)	0.00
GI bleeding events (outpatient)	772 (29.4%)	768 (29.2%)	0.00	7,907 (22.9%)	7,811 (22.6%)	0.01
Hypertension	2,423 (92.3%)	2,431 (92.6%)	-0.01	30,552 (88.4%)	30,418 (88.0%)	0.01
Ischemic Heart Disease	1,362 (51.9%)	1,382 (52.6%)	-0.01	15,599 (45.1%)	15,528 (44.9%)	0.00
Liver disease	196 (7.5%)	180 (6.9%)	0.02	2,334 (6.8%)	2,354 (6.8%)	0.00
Malignancy	406 (15.5%)	398 (15.2%)	0.01	5,549 (16.1%)	5,529 (16.0%)	0.00
Obesity	366 (13.9%)	348 (13.3%)	0.02	7,323 (21.2%)	7,466 (21.6%)	-0.01
Peptic ulcer	97 (3.7%)	102 (3.9%)	-0.01	783 (2.3%)	796 (2.3%)	0.00
PVD or PVD Surgery	677 (25.8%)	696 (26.5%)	-0.02	5,215 (15.1%)	5,275 (15.3%)	-0.01
Smoking	769 (29.3%)	786 (29.9%)	-0.01	9,685 (28.0%)	9,759 (28.2%)	0.00
Stroke (inpatient)	433 (16.5%)	462 (17.6%)	-0.03	1,951 (5.6%)	2,010 (5.8%)	-0.01
Stroke (outpatient)	1,146 (43.6%)	1,167 (44.4%)	-0.02	7,672 (22.2%)	7,670 (22.2%)	0.00
Syncope	505 (19.2%)	494 (18.8%)	0.01	3,215 (9.3%)	3,231 (9.3%)	0.00
**Medications**						
ACE inhibitors	798 (30.4%)	799 (30.4%)	0.00	10,029 (29.0%)	9,986 (28.9%)	0.00
Angiotensin II receptor blockers	87 (3.3%)	97 (3.7%)	-0.02	1,507 (4.4%)	1,514 (4.4%)	0.00
Antiarrhythmic agents	305 (11.6%)	307 (11.7%)	0.00	5,680 (16.4%)	5,644 (16.3%)	0.00
Anticoagulants, injectable	68 (2.6%)	75 (2.9%)	-0.02	485 (1.4%)	495 (1.4%)	0.00
Anticonvulsants	557 (21.2%)	570 (21.7%)	-0.01	5,396 (15.6%)	5,351 (15.5%)	0.00
Antidepressants - Other	474 (18.1%)	489 (18.6%)	-0.01	2,737 (7.9%)	2,668 (7.7%)	0.01
Antidepressants - SSRI/SNRI	893 (34.0%)	906 (34.5%)	-0.01	5,905 (17.1%)	5,916 (17.1%)	0.00
Antidepressants - Tricyclics	53 (2.0%)	52 (2.0%)	0.00	768 (2.2%)	753 (2.2%)	0.00
Antiplatelet agents	349 (13.3%)	360 (13.7%)	-0.01	4,307 (12.5%)	4,269 (12.4%)	0.00
Antipsychotic agents	322 (12.3%)	347 (13.2%)	-0.03	426 (1.2%)	422 (1.2%)	0.00
Anxiolytics - Benzodiazepines	447 (17.0%)	478 (18.2%)	-0.03	4,812 (13.9%)	4,732 (13.7%)	0.01
Anxiolytics - Other	51 (1.9%)	61 (2.3%)	-0.03	330 (1.0%)	341 (1.0%)	0.00
Beta-blockers	1,709 (65.1%)	1,717 (65.4%)	-0.01	22,958 (66.4%)	22,995 (66.5%)	0.00
Bronchodilators	500 (19.0%)	535 (20.4%)	-0.04	6,253 (18.1%)	6,251 (18.1%)	0.00
Calcium channel blockers	0 (0.0%)	1 (0.0%)	-	16 (0.0%)	19 (0.1%)	-0.04
Corticosteroids, inhaled	395 (15.0%)	427 (16.3%)	-0.04	6,113 (17.7%)	6,116 (17.7%)	0.00
Corticosteroids, oral	582 (22.2%)	626 (23.8%)	-0.04	9,441 (27.3%)	9,454 (27.4%)	0.00
Dementia drugs	1,086 (41.4%)	1,055 (40.2%)	0.02	377 (1.1%)	394 (1.1%)	0.00
Diabetes agents - Insulin	278 (10.6%)	256 (9.7%)	0.03	2,496 (7.2%)	2,539 (7.3%)	0.00
Diabetes agents - Metformin	392 (14.9%)	373 (14.2%)	0.02	6,072 (17.6%)	6,131 (17.7%)	0.00
Diabetes agents - Other	119 (4.5%)	122 (4.6%)	0.00	1,577 (4.6%)	1,588 (4.6%)	0.00
Diabetes agents - Sulfonylurea	247 (9.4%)	249 (9.5%)	0.00	3,608 (10.4%)	3,647 (10.6%)	-0.01
Diuretics	1,355 (51.6%)	1,350 (51.4%)	0.00	18,781 (54.3%)	18,766 (54.3%)	0.00
Estrogen	45 (1.7%)	44 (1.7%)	0.00	583 (1.7%)	592 (1.7%)	0.00
Fibrates	76 (2.9%)	88 (3.4%)	-0.03	1,431 (4.1%)	1,440 (4.2%)	-0.01
GI - H2 blockers	247 (9.4%)	247 (9.4%)	0.00	2,173 (6.3%)	2,233 (6.5%)	-0.01
GI - Proton-pump inhibitors	819 (31.2%)	859 (32.7%)	-0.03	9,617 (27.8%)	9,492 (27.5%)	0.01
GI - Sucralfate	51 (1.9%)	53 (2.0%)	-0.01	483 (1.4%)	495 (1.4%)	0.00
Hypnotics	167 (6.4%)	175 (6.7%)	-0.01	1,845 (5.3%)	1,871 (5.4%)	0.00
Nitrates	316 (12.0%)	313 (11.9%)	0.00	3,746 (10.8%)	3,748 (10.8%)	0.00
NSAIDs	260 (9.9%)	285 (10.9%)	-0.03	4,200 (12.2%)	4,203 (12.2%)	0.00
Opioids	817 (31.1%)	879 (33.5%)	-0.05	11,721 (33.9%)	11,682 (33.8%)	0.00
Parkinsonism drugs	183 (7.0%)	187 (7.1%)	0.00	1,024 (3.0%)	1,015 (2.9%)	0.01
Statins	1,661 (63.3%)	1,665 (63.4%)	0.00	21,608 (62.5%)	21,687 (62.7%)	0.00
Thyroid hormone replacement	638 (24.3%)	642 (24.4%)	0.00	6,955 (20.1%)	6,967 (20.2%)	0.00
**Health care use**						
Emergency department visits	1,869 (71.2%)	1,889 (71.9%)	-0.02	16,543 (47.9%)	16,683 (48.3%)	-0.01
Home oxygen use	153 (5.8%)	154 (5.9%)	0.00	1,337 (3.9%)	1,365 (3.9%)	0.00

© 2023 Lin KJ et al. *JAMA Network Open*.

Characteristics<br>Number of Patients (%)	With Dementia			Without Dementia		
	Apixaban (n=2,626)	Warfarin (n=2,626)	St. Diff	Apixaban (n=34,566)	Warfarin (n=34,566)	St. Diff
Hospitalizations	1,769 (67.4%)	1,795 (68.4%)	-0.02	13,499 (39.1%)	13,725 (39.7%)	-0.01
**Geographic Region**						
Northeast	421 (16.0%)	425 (16.2%)	-0.01	4,759 (13.8%)	4,710 (13.6%)	0.01
Midwest	621 (23.6%)	608 (23.2%)	0.01	7,713 (22.3%)	7,694 (22.3%)	0.00
South	905 (34.5%)	895 (34.1%)	0.01	11,268 (32.6%)	11,266 (32.6%)	0.00
West	678 (25.8%)	697 (26.5%)	-0.02	10,804 (31.3%)	10,869 (31.4%)	0.00
Other	1 (0.0%)	1 (0.0%)	-	22 (0.1%)	27 (0.1%)	0.00
**Business Type Code (Recategorized)**						
Commercial	98 (3.7%)	101 (3.8%)	-0.01	2,217 (6.4%)	2,211 (6.4%)	0.00
Medicare	2,528 (96.3%)	2,525 (96.2%)	0.01	32,349 (93.6%)	32,355 (93.6%)	0.00

Abbreviations: ACE, angiotensin converting enzyme; CCI, combined comorbidity; CFI, claims-based frailty index; CKD, chronic kidney disease; COPD, chronic obstructive pulmonary disease; GI, gastrointestinal; NSAID, non-steroidal anti-inflammatory drug; PVD, peripheral vascular disease; SNRI, serotonin-norepinephrine reuptake inhibitor; SSRI, selective serotonin reuptake inhibitor.

[A] Other race category includes Unknown, Other, Asian, Hispanic, North American Native, and Missing Race Categories in Medicare. Asian, Hispanic, Unknown, and Missing categories are included for race in Optum. Race is unavailable in the MarketScan database.

© 2023 Lin KJ et al. *JAMA Network Open*.

eTable 19. Selected Characteristics of Propensity Score–Matched Population With Atrial Fibrillation Treated With Warfarin vs Apixaban by Dementia Diagnosis in the MarketScan Population

Characteristics Number of Patients (%)	With Dementia			Without Dementia		
	Apixaban (n=1,375)	Warfarin (n=1,375)	St. Diff	Apixaban (n=19,234)	Warfarin (n=19,234)	St. Diff
**Quarter/Year of Cohort Entry Date**						
2013	125 (9.1%)	131 (9.5%)	0.00	2,354 (12.2%)	2,404 (12.5%)	0.00
2014	257 (18.7%)	252 (18.3%)	0.00	4,817 (25.0%)	4,809 (25.0%)	0.00
2015	360 (26.2%)	352 (25.6%)	0.01	4,390 (22.8%)	4,328 (22.5%)	0.00
2016	354 (25.7%)	355 (25.8%)	0.00	3,897 (20.3%)	3,903 (20.3%)	0.00
2017	159 (11.6%)	156 (11.3%)	0.00	1,863 (9.7%)	1,854 (9.6%)	0.00
2018	47 (3.4%)	48 (3.5%)	0.00	785 (4.1%)	768 (4.0%)	0.00
2019	48 (3.5%)	51 (3.7%)	0.00	736 (3.8%)	741 (3.9%)	0.00
2020	25 (1.8%)	30 (2.2%)	0.00	392 (2.0%)	427 (2.2%)	0.00
**Patient Characteristics**						
Mean age (SD)	83.49 (6.45)	83.50 (6.59)	-0.01	78.07 (7.71)	78.00 (7.52)	0.07
Male	643 (46.8%)	651 (47.3%)	-0.01	10,630 (55.3%)	10,652 (55.4%)	0.00
Female	732 (53.2%)	724 (52.7%)	0.01	8,604 (44.7%)	8,582 (44.6%)	0.00
Mean CHA2DS2-VASc score (SD)	4.75 (1.47)	4.71 (1.46)	0.04	3.90 (1.56)	3.89 (1.52)	0.00
Mean HAS-BLED score (SD)	2.57 (0.79)	2.57 (0.80)	0.00	2.20 (0.69)	2.20 (0.69)	0.00
Mean CCI score (SD)	5.86 (3.09)	5.96 (3.12)	-0.10	3.05 (2.62)	3.04 (2.57)	0.01
Mean CFI (SD)	0.30 (0.07)	0.30 (0.07)	0.00	0.19 (0.06)	0.19 (0.05)	0.00
**Medical History**						
Acute myocardial infarction	132 (9.6%)	139 (10.1%)	-0.01	1,340 (7.0%)	1,376 (7.2%)	0.00
Acute renal failure	311 (22.6%)	315 (22.9%)	0.00	2,237 (11.6%)	2,147 (11.2%)	0.01
Alcohol abuse or dependence	32 (2.3%)	35 (2.5%)	0.00	260 (1.4%)	284 (1.5%)	0.00
Anemia	463 (33.7%)	474 (34.5%)	-0.01	4,240 (22.0%)	4,205 (21.9%)	0.00
Cardio-ablation	2 (0.1%)	3 (0.2%)	0.00	84 (0.4%)	81 (0.4%)	0.00
Cardioversion	37 (2.7%)	39 (2.8%)	0.00	1,016 (5.3%)	1,006 (5.2%)	0.00
Cerebrovascular disease	699 (50.8%)	673 (48.9%)	0.02	4,716 (24.5%)	4,740 (24.6%)	0.00
CKD: stage 3, 4 or unspecified	319 (23.2%)	326 (23.7%)	-0.01	3,255 (16.9%)	3,170 (16.5%)	0.00
Congestive heart failure (inpatient)	422 (30.7%)	433 (31.5%)	-0.01	3,793 (19.7%)	3,689 (19.2%)	0.01
Congestive heart failure (outpatient)	584 (42.5%)	585 (42.5%)	0.00	5,972 (31.0%)	5,934 (30.9%)	0.00
Coronary revascularization	25 (1.8%)	33 (2.4%)	-0.01	880 (4.6%)	902 (4.7%)	0.00
COPD	364 (26.5%)	376 (27.3%)	-0.01	3,960 (20.6%)	3,922 (20.4%)	0.00
Dementia	1,375 (100.0%)	1,375 (100.0%)	0.00	0 (0.0%)	0 (0.0%)	0.00
Diabetes	503 (36.6%)	499 (36.3%)	0.00	6,586 (34.2%)	6,674 (34.7%)	-0.01
Endoscopy	35 (2.5%)	37 (2.7%)	0.00	279 (1.5%)	282 (1.5%)	0.00
Falls	317 (23.1%)	309 (22.5%)	0.01	1,270 (6.6%)	1,241 (6.5%)	0.00
Fractures	242 (17.6%)	250 (18.2%)	-0.01	1,513 (7.9%)	1,548 (8.0%)	0.00
GI bleeding events (inpatient)	103 (7.5%)	105 (7.6%)	0.00	802 (4.2%)	798 (4.1%)	0.00
GI bleeding events (outpatient)	214 (15.6%)	213 (15.5%)	0.00	2,770 (14.4%)	2,743 (14.3%)	0.00
Hypertension	1,244 (90.5%)	1,240 (90.2%)	0.00	16,353 (85.0%)	16,321 (84.9%)	0.00
Ischemic Heart Disease	700 (50.9%)	710 (51.6%)	-0.01	8,998 (46.8%)	8,975 (46.7%)	0.00
Liver disease	91 (6.6%)	84 (6.1%)	0.01	1,048 (5.4%)	1,063 (5.5%)	0.00
Malignancy	250 (18.2%)	241 (17.5%)	0.01	3,690 (19.2%)	3,760 (19.5%)	0.00
Obesity	123 (8.9%)	119 (8.7%)	0.00	2,597 (13.5%)	2,534 (13.2%)	0.00
Peptic ulcer	28 (2.0%)	25 (1.8%)	0.00	236 (1.2%)	238 (1.2%)	0.00
PVD or PVD Surgery	314 (22.8%)	321 (23.3%)	-0.01	2,615 (13.6%)	2,581 (13.4%)	0.00
Smoking	139 (10.1%)	138 (10.0%)	0.00	2,274 (11.8%)	2,253 (11.7%)	0.00
Stroke (inpatient)	320 (23.3%)	309 (22.5%)	0.01	1,680 (8.7%)	1,704 (8.9%)	0.00
Stroke (outpatient)	583 (42.4%)	560 (40.7%)	0.02	3,899 (20.3%)	3,913 (20.3%)	0.00
Syncope	278 (20.2%)	281 (20.4%)	0.00	1,818 (9.5%)	1,829 (9.5%)	0.00
**Medications**						
ACE inhibitors	397 (28.9%)	396 (28.8%)	0.00	5,332 (27.7%)	5,263 (27.4%)	0.00
Angiotensin II receptor blockers	71 (5.2%)	80 (5.8%)	-0.01	1,168 (6.1%)	1,182 (6.1%)	0.00
Antiarrhythmic agents	172 (12.5%)	184 (13.4%)	-0.01	3,660 (19.0%)	3,676 (19.1%)	0.00
Anticoagulants, injectable	24 (1.7%)	22 (1.6%)	0.00	211 (1.1%)	223 (1.2%)	0.00
Anticonvulsants	266 (19.3%)	270 (19.6%)	0.00	2,658 (13.8%)	2,681 (13.9%)	0.00
Antidepressants - Other	242 (17.6%)	249 (18.1%)	-0.01	1,166 (6.1%)	1,161 (6.0%)	0.00

© 2023 Lin KJ et al. *JAMA Network Open*.

Characteristics<br>Number of Patients (%)	With Dementia			Without Dementia		
	Apixaban<br>(n=1,375)	Warfarin<br>(n=1,375)	St. Diff	Apixaban<br>(n=19,234)	Warfarin<br>(n=19,234)	St. Diff
Antidepressants - SSRI/SNRI	531 (38.6%)	526 (38.3%)	0.00	3,174 (16.5%)	3,226 (16.8%)	0.00
Antidepressants - Tricyclics	30 (2.2%)	29 (2.1%)	0.00	450 (2.3%)	436 (2.3%)	0.00
Antiplatelet agents	270 (19.6%)	269 (19.6%)	0.00	3,048 (15.8%)	3,028 (15.7%)	0.00
Antipsychotic agents	193 (14.0%)	199 (14.5%)	0.00	247 (1.3%)	252 (1.3%)	0.00
Anxiolytics - Benzodiazepines	281 (20.4%)	263 (19.1%)	0.01	2,904 (15.1%)	2,937 (15.3%)	0.00
Anxiolytics - Other	28 (2.0%)	28 (2.0%)	0.00	156 (0.8%)	167 (0.9%)	0.00
Beta-blockers	911 (66.3%)	928 (67.5%)	-0.01	12,965 (67.4%)	13,004 (67.6%)	0.00
Bronchodilators	254 (18.5%)	255 (18.5%)	0.00	3,683 (19.1%)	3,639 (18.9%)	0.00
Calcium channel blockers	5 (0.4%)	2 (0.1%)	0.00	48 (0.2%)	44 (0.2%)	0.00
Corticosteroids, inhaled	243 (17.7%)	269 (19.6%)	-0.02	4,152 (21.6%)	4,141 (21.5%)	0.00
Corticosteroids, oral	353 (25.7%)	349 (25.4%)	0.00	5,789 (30.1%)	5,798 (30.1%)	0.00
Dementia drugs	668 (48.6%)	663 (48.2%)	0.00	328 (1.7%)	312 (1.6%)	0.00
Diabetes agents - Insulin	139 (10.1%)	135 (9.8%)	0.00	1,517 (7.9%)	1,529 (7.9%)	0.00
Diabetes agents - Metformin	187 (13.6%)	183 (13.3%)	0.00	2,930 (15.2%)	2,975 (15.5%)	0.00
Diabetes agents - Other	97 (7.1%)	79 (5.7%)	0.01	1,182 (6.1%)	1,201 (6.2%)	0.00
Diabetes agents - Sulfonylurea	127 (9.2%)	130 (9.5%)	0.00	1,899 (9.9%)	1,932 (10.0%)	0.00
Diuretics	747 (54.3%)	763 (55.5%)	-0.01	10,759 (55.9%)	10,664 (55.4%)	0.01
Estrogen	38 (2.8%)	31 (2.3%)	0.01	563 (2.9%)	576 (3.0%)	0.00
Fibrates	47 (3.4%)	46 (3.3%)	0.00	995 (5.2%)	972 (5.1%)	0.00
GI - H2 blockers	127 (9.2%)	132 (9.6%)	0.00	1,170 (6.1%)	1,182 (6.1%)	0.00
GI - Proton-pump inhibitors	462 (33.6%)	470 (34.2%)	-0.01	5,704 (29.7%)	5,649 (29.4%)	0.00
GI - Sucralfate	25 (1.8%)	28 (2.0%)	0.00	298 (1.5%)	294 (1.5%)	0.00
Hypnotics	113 (8.2%)	113 (8.2%)	0.00	1,342 (7.0%)	1,404 (7.3%)	0.00
Nitrates	169 (12.3%)	186 (13.5%)	-0.01	2,443 (12.7%)	2,404 (12.5%)	0.00
NSAIDs	144 (10.5%)	156 (11.3%)	-0.01	2,601 (13.5%)	2,642 (13.7%)	0.00
Opioids	504 (36.7%)	488 (35.5%)	0.01	7,520 (39.1%)	7,477 (38.9%)	0.00
Parkinsonism drugs	105 (7.6%)	102 (7.4%)	0.00	636 (3.3%)	624 (3.2%)	0.00
Statins	870 (63.3%)	877 (63.8%)	-0.01	12,288 (63.9%)	12,344 (64.2%)	0.00
Thyroid hormone replacement	356 (25.9%)	364 (26.5%)	-0.01	4,113 (21.4%)	4,092 (21.3%)	0.00
**Health care use**						
Emergency department visits	1,062 (77.2%)	1,045 (76.0%)	0.01	9,519 (49.5%)	9,534 (49.6%)	0.00
Hospitalizations	993 (72.2%)	1,001 (72.8%)	-0.01	8,542 (44.4%)	8,539 (44.4%)	0.00
**Geographic Region**						
Northeast	313 (22.8%)	308 (22.4%)	0.00	4,526 (23.5%)	4,523 (23.5%)	0.00
Midwest	548 (39.9%)	549 (39.9%)	0.00	6,981 (36.3%)	6,991 (36.3%)	0.00
South	380 (27.6%)	380 (27.6%)	0.00	5,219 (27.1%)	5,217 (27.1%)	0.00
West	132 (9.6%)	133 (9.7%)	0.00	2,451 (12.7%)	2,439 (12.7%)	0.00
Other						
**Employee Classification (Recategorized)**						
Salary Non-union	118 (8.6%)	125 (9.1%)	-0.01	1,870 (9.7%)	1,912 (9.9%)	0.00
Salary Union	34 (2.5%)	28 (2.0%)	0.00	432 (2.2%)	429 (2.2%)	0.00
Salary Other	23 (1.7%)	27 (2.0%)	0.00	383 (2.0%)	388 (2.0%)	0.00
Hourly Non-Union	72 (5.2%)	62 (4.5%)	0.01	1,137 (5.9%)	1,110 (5.8%)	0.00
Hourly Union	507 (36.9%)	511 (37.2%)	0.00	6,251 (32.5%)	6,172 (32.1%)	0.00
Hourly Other	8 (0.6%)	5 (0.4%)	0.00	126 (0.7%)	130 (0.7%)	0.00
Non-Union	265 (19.3%)	251 (18.3%)	0.01	3,446 (17.9%)	3,469 (18.0%)	0.00
Union	9 (0.7%)	9 (0.7%)	0.00	230 (1.2%)	233 (1.2%)	0.00
Unknown	339 (24.7%)	357 (26.0%)	-0.01	5,359 (27.9%)	5,391 (28.0%)	0.00
**Employment Status (Recategorized)**						
Active Full Time	13 (0.9%)	16 (1.2%)	0.00	920 (4.8%)	918 (4.8%)	0.00
Active Part Time or Seasonal	0 (0.0%)	2 (0.1%)	0.00	39 (0.2%)	37 (0.2%)	0.00
Early Retiree	4 (0.3%)	3 (0.2%)	0.00	158 (0.8%)	158 (0.8%)	0.00
Medicare Eligible Retiree	968 (70.4%)	949 (69.0%)	0.01	13,765 (71.6%)	13,712 (71.3%)	0.00
Retiree	25 (1.8%)	27 (2.0%)	0.00	331 (1.7%)	353 (1.8%)	0.00
COBRA Continuee	0 (0.0%)	0 (0.0%)	0.00	11 (0.1%)	8 (0.0%)	0.00
Long Term Disability	1 (0.1%)	1 (0.1%)	0.00	15 (0.1%)	21 (0.1%)	0.00
Surviving Spouse/Dependent	230 (16.7%)	237 (17.2%)	-0.01	1,838 (9.6%)	1,858 (9.7%)	0.00
Other/Unknown/Missing	134 (9.7%)	140 (10.2%)	0.00	2,157 (11.2%)	2,169 (11.3%)	0.00
**Health Plan Indicator**						

© 2023 Lin KJ et al. *JAMA Network Open*.

Characteristics Number of Patients (%)	With Dementia			Without Dementia		
	Apixaban (n=1,375)	Warfarin (n=1,375)	St. Diff	Apixaban (n=19,234)	Warfarin (n=19,234)	St. Diff
Employer	1,226 (89.2%)	1,219 (88.7%)	0.01	16,830 (87.5%)	16,827 (87.5%)	0.00
Health Plan	149 (10.8%)	156 (11.3%)	-0.01	2,404 (12.5%)	2,407 (12.5%)	0.00
**MHSA Coverage Indicator**						
Not Covered/Claims Not Present	84 (6.1%)	86 (6.3%)	0.00	1,188 (6.2%)	1,177 (6.1%)	0.00
Covered/Possible MHSA Claims	1,157 (84.1%)	1,137 (82.7%)	0.02	16,026 (83.3%)	16,026 (83.3%)	0.00
Missing	134 (9.7%)	152 (11.1%)	-0.01	2,020 (10.5%)	2,031 (10.6%)	0.00
**Plan Indicator**						
Basic/major medical	0 (0.0%)	0 (0.0%)	0.00	0 (0.0%)	0 (0.0%)	0.00
Comprehensive	670 (48.7%)	653 (47.5%)	0.01	8,088 (42.1%)	8,014 (41.7%)	0.00
EPO	0 (0.0%)	0 (0.0%)	0.00	52 (0.3%)	47 (0.2%)	0.00
HMO	96 (7.0%)	102 (7.4%)	0.00	1,909 (9.9%)	1,843 (9.6%)	0.00
POS	43 (3.1%)	44 (3.2%)	0.00	659 (3.4%)	660 (3.4%)	0.00
PPO	539 (39.2%)	545 (39.6%)	0.00	7,953 (41.3%)	8,118 (42.2%)	-0.01
POS with capitation	6 (0.4%)	10 (0.7%)	0.00	132 (0.7%)	126 (0.7%)	0.00
CDHP	1 (0.1%)	1 (0.1%)	0.00	125 (0.6%)	121 (0.6%)	0.00
HDHP	0 (0.0%)	0 (0.0%)	0.00	64 (0.3%)	65 (0.3%)	0.00
Missing	20 (1.5%)	20 (1.5%)	0.00	252 (1.3%)	240 (1.2%)	0.00

Abbreviations: ACE, angiotensin converting enzyme; CCI, combined comorbidity; CFI, claims-based frailty index; CKD, chronic kidney disease; COPD, chronic obstructive pulmonary disease; GI, gastrointestinal; NSAID, non-steroidal anti-inflammatory drug; PVD, peripheral vascular disease; SNRI, serotonin-norepinephrine reuptake inhibitor; SSRI, selective serotonin reuptake inhibitor.

© 2023 Lin KJ et al. *JAMA Network Open*.

eTable 20. Selected Characteristics of Propensity Score–Matched Population With Atrial Fibrillation Treated With Dabigatran vs Apixaban by Dementia Diagnosis Pooled Across Medicare, Optum, and MarketScan Populations

Characteristics<br>Number of Patients (%)	With Dementia			Without Dementia		
	Apixaban (n=4,173)	Dabigatran (n=4,173)	St. Diff	Apixaban (n=59,091)	Dabigatran (n=59,091)	St. Diff
**Patient Characteristics**						
Mean Age (SD)	81.89 (6.96)	81.91 (6.83)	0.00	76.14 (7.0)	76.17 (6.94)	0.00
Male	1,749 (41.9%)	1,707 (40.9%)	0.02	31,256 (52.9%)	31,203 (52.8%)	0.00
Female	2,424 (58.1%)	2,466 (59.1%)	-0.02	27,835 (47.1%)	27,888 (47.2%)	0.00
Black	250 (6.5%)	264 (6.9%)	-0.01	2,189 (4.1%)	2,231 (4.2%)	0.00
White	3,284 (85.5%)	3,287 (85.5%)	0.00	47,119 (89.3%)	47,000 (89.1%)	0.01
Other[A]	309 (8.0%)	292 (7.6%)	0.02	3,446 (6.5%)	3,523 (6.7%)	-0.01
Dual Status[B]	558 (16.4%)	544 (16.0%)	0.01	1,998 (4.3%)	2,050 (4.4%)	-0.01
Mean CHA2DS2-VASc score (SD)	5.72 (1.63)	5.71 (1.62)	0.01	4.32 (1.7)	4.32 (1.66)	0.00
Mean HAS-BLED score (SD)	2.65 (0.78)	2.65 (0.79)	0.00	2.20 (0.7)	2.20 (0.70)	0.00
Mean CCI score (SD)	5.79 (3.00)	5.80 (2.99)	0.00	2.68 (2.4)	2.70 (2.45)	-0.01
Mean CFI (SD)	0.31 (0.08)	0.31 (0.08)	-0.01	0.19 (0.1)	0.19 (0.06)	0.00
**Cardiovascular conditions**						
Acute Myocardial infarction	335 (8.0%)	295 (7.1%)	0.04	2,559 (4.3%)	2,604 (4.4%)	0.00
Cardio-ablation	17 (0.4%)	20 (0.5%)	-0.01	405 (0.7%)	391 (0.7%)	0.00
Cardioversion	131 (3.1%)	118 (2.8%)	0.02	3,797 (6.4%)	3,887 (6.6%)	-0.01
Cerebrovascular disease	2,237 (53.6%)	2,187 (52.4%)	0.02	14,478 (24.5%)	14,348 (24.3%)	0.01
Congestive heart failure (inpatient)	1,363 (32.7%)	1,344 (32.2%)	0.01	8,379 (14.2%)	8,511 (14.4%)	-0.01
Congestive heart failure (outpatient)	1,926 (46.2%)	1,880 (45.1%)	0.02	16,770 (28.4%)	16,883 (28.6%)	0.00
Coronary revascularization	112 (2.7%)	89 (2.1%)	0.04	1,494 (2.5%)	1,537 (2.6%)	0.00
Hypertension	3,844 (92.1%)	3,835 (91.9%)	0.01	49,681 (84.1%)	49,755 (84.2%)	0.00
Ischemic Heart Disease	2,282 (54.7%)	2,238 (53.6%)	0.02	25,133 (42.5%)	25,104 (42.5%)	0.00
PVD or PVD Surgery	968 (23.2%)	964 (23.1%)	0.00	7,166 (12.1%)	7,208 (12.2%)	0.00
Stroke (inpatient)	840 (20.1%)	841 (20.2%)	0.00	3,964 (6.7%)	3,998 (6.8%)	0.00
Stroke (outpatient)	895 (21.4%)	855 (20.5%)	0.02	6,114 (10.3%)	5,985 (10.1%)	0.01
Syncope	824 (19.7%)	820 (19.7%)	0.00	5,367 (9.1%)	5,361 (9.1%)	0.00
**Non-cardiovascular conditions**						
Acute renal failure	889 (21.3%)	870 (20.8%)	0.01	4,864 (8.2%)	4,855 (8.2%)	0.00
Alcohol abuse or dependence	148 (3.5%)	139 (3.3%)	0.01	1,034 (1.7%)	1,041 (1.8%)	0.00
Anemia	1,671 (40.0%)	1,692 (40.5%)	-0.01	13,416 (22.7%)	13,478 (22.8%)	0.00
CKD: stage 3, 4 or unspecified	936 (22.4%)	951 (22.8%)	-0.01	6,766 (11.5%)	6,783 (11.5%)	0.00
COPD	1,202 (28.8%)	1,201 (28.8%)	0.00	11,589 (19.6%)	11,738 (19.9%)	-0.01
Dementia	4,173 (100.0%)	4,173 (100.0%)		0 (0.0%)	0 (0.0%)	
Diabetes	1,815 (43.5%)	1,814 (43.5%)	0.00	21,066 (35.7%)	21,120 (35.7%)	0.00
Endoscopy	184 (4.4%)	171 (4.1%)	0.02	871 (1.5%)	874 (1.5%)	0.00
Falls	659 (15.8%)	640 (15.3%)	0.01	2,156 (3.6%)	2,172 (3.7%)	0.00
Fractures	710 (17.0%)	716 (17.2%)	0.00	4,268 (7.2%)	4,227 (7.2%)	0.00
GI bleeding (inpatient)	451 (10.8%)	417 (10.0%)	0.03	2,551 (4.3%)	2,482 (4.2%)	0.01
GI bleeding (outpatient)	580 (13.9%)	586 (14.0%)	0.00	7,423 (12.6%)	7,434 (12.6%)	0.00
Liver disease	299 (7.2%)	307 (7.4%)	-0.01	3,353 (5.7%)	3,418 (5.8%)	0.00
Malignancy	612 (14.7%)	642 (15.4%)	-0.02	10,620 (18.0%)	10,622 (18.0%)	0.00
Obesity	656 (15.7%)	633 (15.2%)	0.02	11,601 (19.6%)	11,607 (19.6%)	0.00
Peptic Ulcer	115 (2.8%)	122 (2.9%)	-0.01	906 (1.5%)	955 (1.6%)	-0.01
Smoking	1,320 (31.6%)	1,303 (31.2%)	0.01	16,403 (27.8%)	16,338 (27.6%)	0.00
**Cardiovascular medications**						
ACE inhibitors	1,230 (29.5%)	1,240 (29.7%)	-0.01	15,585 (26.4%)	15,622 (26.4%)	0.00
Angiotensin II receptor blockers	290 (6.9%)	268 (6.4%)	0.02	3,868 (6.5%)	3,816 (6.5%)	0.00
Antiarrhythmic agents	691 (16.6%)	680 (16.3%)	0.01	12,990 (22.0%)	12,985 (22.0%)	0.00
Anticoagulants, injectable	56 (1.3%)	47 (1.1%)	0.02	414 (0.7%)	410 (0.7%)	0.00
Antiplatelet agent	803 (19.2%)	738 (17.7%)	0.04	8,090 (13.7%)	7,960 (13.5%)	0.01
Beta-blockers	2,797 (67.0%)	2,775 (66.5%)	0.01	38,317 (64.8%)	38,390 (65.0%)	0.00
Calcium channel blockers	88 (2.1%)	88 (2.1%)	0.00	984 (1.7%)	991 (1.7%)	0.00
Diuretics	2,391 (57.3%)	2,387 (57.2%)	0.00	31,044 (52.5%)	31,063 (52.6%)	0.00
Fibrates	154 (3.7%)	156 (3.7%)	0.00	2,771 (4.7%)	2,786 (4.7%)	0.00
Nitrates	651 (15.6%)	630 (15.1%)	0.01	6,575 (11.1%)	6,604 (11.2%)	0.00

© 2023 Lin KJ et al. *JAMA Network Open*.

Characteristics Number of Patients (%)	With Dementia			Without Dementia		
	Apixaban (n=4,173)	Dabigatran (n=4,173)	St. Diff	Apixaban (n=59,091)	Dabigatran (n=59,091)	St. Diff
Statins	2,790 (66.9%)	2,771 (66.4%)	0.01	37,506 (63.5%)	37,553 (63.6%)	0.00
**Other medications**						
Anticonvulsants	979 (23.5%)	979 (23.5%)	0.00	8,769 (14.8%)	8,728 (14.8%)	0.00
Antidepressants - Other	794 (19.0%)	800 (19.2%)	0.00	4,121 (7.0%)	4,126 (7.0%)	0.00
Antidepressants - SSRI/SNRI	1,742 (41.7%)	1,693 (40.6%)	0.02	11,094 (18.8%)	10,988 (18.6%)	0.00
Antidepressants - Tricyclics	152 (3.6%)	138 (3.3%)	0.02	1,489 (2.5%)	1,496 (2.5%)	0.00
Antipsychotic agents	726 (17.4%)	726 (17.4%)	0.00	1,012 (1.7%)	1,020 (1.7%)	0.00
Anxiolytics (except benzodiazepine)	90 (2.2%)	104 (2.5%)	-0.02	600 (1.0%)	583 (1.0%)	0.00
Anxiolytics - Benzodiazepines	1,039 (24.9%)	1,023 (24.5%)	0.01	10,194 (17.3%)	10,199 (17.3%)	0.00
Bronchodilators	902 (21.6%)	884 (21.2%)	0.01	10,947 (18.5%)	10,994 (18.6%)	0.00
Corticosteroids, inhaled	848 (20.3%)	822 (19.7%)	0.02	13,039 (22.1%)	13,127 (22.2%)	0.00
Corticosteroids, oral	1,221 (29.3%)	1,229 (29.5%)	0.00	18,626 (31.5%)	18,743 (31.7%)	0.00
Dementia drugs	1,995 (47.8%)	1,997 (47.9%)	0.00	1,069 (1.8%)	1,068 (1.8%)	0.00
Diabetes agents - Insulin	471 (11.3%)	470 (11.3%)	0.00	4,076 (6.9%)	4,127 (7.0%)	0.00
Diabetes agents - Metformin	667 (16.0%)	678 (16.2%)	-0.01	9,888 (16.7%)	10,000 (16.9%)	-0.01
Diabetes agents - Other	282 (6.8%)	294 (7.0%)	-0.01	3,238 (5.5%)	3,304 (5.6%)	0.00
Diabetes agents - Sulfonylurea	449 (10.8%)	428 (10.3%)	0.02	5,623 (9.5%)	5,669 (9.6%)	0.00
Estrogen	106 (2.5%)	114 (2.7%)	-0.01	1,946 (3.3%)	1,962 (3.3%)	0.00
GI - H2 blockers	531 (12.7%)	507 (12.1%)	0.02	4,086 (6.9%)	3,984 (6.7%)	0.01
GI - Proton-pump inhibitors	1,588 (38.1%)	1,586 (38.0%)	0.00	17,923 (30.3%)	17,998 (30.5%)	0.00
GI - Sucralfate	114 (2.7%)	107 (2.6%)	0.01	1,093 (1.8%)	1,094 (1.9%)	0.00
Hypnotics	414 (9.9%)	424 (10.2%)	-0.01	5,121 (8.7%)	5,124 (8.7%)	0.00
NSAIDs	621 (14.9%)	630 (15.1%)	-0.01	9,590 (16.2%)	9,715 (16.4%)	-0.01
Opioids	1,706 (40.9%)	1,697 (40.7%)	0.00	22,531 (38.1%)	22,468 (38.0%)	0.00
Parkinsonism drugs	372 (8.9%)	355 (8.5%)	0.01	2,089 (3.5%)	2,080 (3.5%)	0.00
Thyroid hormone replacement	1,097 (26.3%)	1,120 (26.8%)	-0.01	12,452 (21.1%)	12,338 (20.9%)	0.00
**Health care utilization**						
Emergency department visits	2,371 (56.8%)	2,334 (55.9%)	0.02	22,451 (38.0%)	22,442 (38.0%)	0.00
Home Health Day[C]	568 (16.7%)	552 (16.3%)	0.01	2,202 (4.8%)	2,154 (4.7%)	0.00
Home oxygen use	197 (4.7%)	191 (4.6%)	0.01	1,472 (2.5%)	1,508 (2.6%)	0.00
Hospitalizations	2,991 (71.7%)	3,039 (72.8%)	-0.03	23,840 (40.3%)	24,122 (40.8%)	-0.01
**Geographic Region**						
Northeast	935 (22.4%)	914 (21.9%)	0.01	12,936 (21.9%)	12,837 (21.7%)	0.00
Midwest	843 (20.2%)	870 (20.8%)	-0.02	12,760 (21.6%)	12,785 (21.6%)	0.00
South	1,731 (41.5%)	1,704 (40.8%)	0.01	22,948 (38.8%)	22,877 (38.7%)	0.00
West	652 (15.6%)	678 (16.2%)	-0.02	10,385 (17.6%)	10,519 (17.8%)	-0.01
Other	n<11	7 (0.2%)	-	62 (0.1%)	73 (0.1%)	-0.01

Abbreviations: ACE, angiotensin converting enzyme; CCI, combined comorbidity; CFI, claims-based frailty index; CKD, chronic kidney disease; COPD, chronic obstructive pulmonary disease; GI, gastrointestinal; NSAID, non-steroidal anti-inflammatory drug; PVD, peripheral vascular disease; SNRI, serotonin-norepinephrine reuptake inhibitor; SSRI, selective serotonin reuptake inhibitor.

[A] Other race category includes Unknown, Other, Asian, Hispanic, North American Native, and Missing Race Categories in Medicare. Asian, Hispanic, Unknown, and Missing categories are included for race in Optum. Race is unavailable in the MarketScan database.

[B] With both Medicare are Medicaid enrollment eligibility

[C] Home Health services received (days)

© 2023 Lin KJ et al. *JAMA Network Open*.

eTable 21. Selected Characteristics of Propensity Score–Matched Population With Atrial Fibrillation Treated With Dabigatran vs Apixaban by Dementia Diagnosis in the Medicare Population

Characteristics<br>Number of Patients (%)	With Dementia			Without Dementia		
	Apixaban (n=3,393)	Dabigatran (n=3,393)	St. Diff	Apixaban (n=46,202)	Dabigatran (n=46,202)	St. Diff
**Quarter/Year of Cohort Entry Date**						
Q1 2013	20 (0.6%)	20 (0.6%)	0.00	223 (0.5%)	223 (0.5%)	0.00
Q2 2013	126 (3.7%)	140 (4.1%)	-0.02	1,786 (3.9%)	1,910 (4.1%)	-0.01
Q3 2013	163 (4.8%)	145 (4.3%)	0.02	2,476 (5.4%)	2,486 (5.4%)	0.00
Q4 2013	223 (6.6%)	228 (6.7%)	0.00	2,892 (6.3%)	2,897 (6.3%)	0.00
Q1 2014	236 (7.0%)	224 (6.6%)	0.02	3,437 (7.4%)	3,298 (7.1%)	0.01
Q2 2014	247 (7.3%)	250 (7.4%)	0.00	3,107 (6.7%)	3,049 (6.6%)	0.00
Q3 2014	206 (6.1%)	203 (6.0%)	0.00	2,297 (5.0%)	2,320 (5.0%)	0.00
Q4 2014	151 (4.5%)	152 (4.5%)	0.00	2,042 (4.4%)	2,065 (4.5%)	0.00
Q1 2015	181 (5.3%)	164 (4.8%)	0.02	2,272 (4.9%)	2,214 (4.8%)	0.00
Q2 2015	130 (3.8%)	159 (4.7%)	-0.04	1,896 (4.1%)	1,926 (4.2%)	-0.01
Q3 2015	110 (3.2%)	121 (3.6%)	-0.02	1,731 (3.7%)	1,691 (3.7%)	0.00
Q4 2015	136 (4.0%)	140 (4.1%)	-0.01	2,209 (4.8%)	2,240 (4.8%)	0.00
Q1 2016	188 (5.5%)	197 (5.8%)	-0.01	3,042 (6.6%)	3,027 (6.6%)	0.00
Q2 2016	213 (6.3%)	210 (6.2%)	0.00	3,042 (6.6%)	2,987 (6.5%)	0.00
Q3 2016	213 (6.3%)	192 (5.7%)	0.03	2,471 (5.3%)	2,528 (5.5%)	-0.01
Q4 2016	189 (5.6%)	188 (5.5%)	0.00	2,541 (5.5%)	2,545 (5.5%)	0.00
Q1 2017	189 (5.6%)	185 (5.5%)	0.00	2,575 (5.6%)	2,643 (5.7%)	0.00
Q2 2017	176 (5.2%)	174 (5.1%)	0.00	2,219 (4.8%)	2,269 (4.9%)	0.00
Q3 2017	158 (4.7%)	151 (4.5%)	0.01	2,030 (4.4%)	2,030 (4.4%)	0.00
Q4 2017	138 (4.1%)	150 (4.4%)	-0.01	1,914 (4.1%)	1,854 (4.0%)	0.01
**Patient Characteristics**						
Mean age (SD)	81.93 (7.14)	81.94 (6.97)	0.00	76.24 (7.03)	76.29 (6.97)	-0.01
Male	1,371 (40.4%)	1,337 (39.4%)	0.02	23,868 (51.7%)	23,858 (51.6%)	0.00
Female	2,022 (59.6%)	2,056 (60.6%)	-0.02	22,334 (48.3%)	22,344 (48.4%)	0.00
Black	221 (6.5%)	225 (6.6%)	0.00	1,717 (3.7%)	1,738 (3.8%)	-0.01
White	2,955 (87.1%)	2,953 (87.0%)	0.00	42,143 (91.2%)	42,077 (91.1%)	0.00
Other[A]	217 (6.4%)	215 (6.3%)	0.00	2,342 (5.1%)	2,387 (5.2%)	0.00
Dual Status[B]	558 (16.4%)	544 (16.0%)	0.01	1,998 (4.3%)	2,050 (4.4%)	0.00
Mean CHA_2DS_2-VASc score (SD)	5.82 (1.65)	5.81 (1.63)	0.01	4.40 (1.68)	4.40 (1.68)	0.00
Mean HAS-BLED score (SD)	2.67 (0.79)	2.67 (0.79)	0.00	2.20 (0.71)	2.20 (0.71)	0.00
Mean CCI score (SD)	5.79 (2.97)	5.76 (2.98)	0.01	2.65 (2.41)	2.67 (2.44)	-0.01
Mean CFI (SD)	0.31 (0.08)	0.31 (0.08)	0.00	0.19 (0.06)	0.19 (0.06)	0.00
**Medical History**						
Acute renal failure	735 (21.7%)	716 (21.1%)	0.01	3,923 (8.5%)	3,898 (8.4%)	0.00
Acute myocardial infarction	280 (8.3%)	250 (7.4%)	0.03	1,966 (4.3%)	2,024 (4.4%)	0.00
Alcohol abuse or dependence	120 (3.5%)	116 (3.4%)	0.01	749 (1.6%)	766 (1.7%)	-0.01
Anemia	1,424 (42.0%)	1,442 (42.5%)	-0.01	10,903 (23.6%)	10,990 (23.8%)	0.00
Cardio-ablation	16 (0.5%)	18 (0.5%)	0.00	303 (0.7%)	294 (0.6%)	0.01
Cardioversion	108 (3.2%)	95 (2.8%)	0.02	2,865 (6.2%)	2,953 (6.4%)	-0.01
Cerebrovascular disease	1,853 (54.6%)	1,808 (53.3%)	0.03	11,619 (25.1%)	11,514 (24.9%)	0.00
CKD: stage 3, 4 or unspecified	743 (21.9%)	746 (22.0%)	0.00	4,909 (10.6%)	4,936 (10.7%)	0.00
Congestive heart failure (inpatient)	1,191 (35.1%)	1,159 (34.2%)	0.02	6,795 (14.7%)	6,910 (15.0%)	-0.01
Congestive heart failure (outpatient)	1,588 (46.8%)	1,532 (45.2%)	0.03	12,840 (27.8%)	12,897 (27.9%)	0.00
Coronary revascularization	102 (3.0%)	79 (2.3%)	0.04	1,230 (2.7%)	1,257 (2.7%)	0.00
COPD	1,021 (30.1%)	1,000 (29.5%)	0.01	9,163 (19.8%)	9,233 (20.0%)	-0.01
Dementia	3,393 (100.0%)	3,393 (100.0%)	-	0 (0.0%)	0 (0.0%)	-
Diabetes	1,474 (43.4%)	1,484 (43.7%)	-0.01	16,622 (36.0%)	16,673 (36.1%)	0.00
Endoscopy	157 (4.6%)	144 (4.2%)	0.02	740 (1.6%)	738 (1.6%)	0.00
Falls	474 (14.0%)	449 (13.2%)	0.02	1,295 (2.8%)	1,294 (2.8%)	0.00
Fractures	582 (17.2%)	585 (17.2%)	0.00	3,419 (7.4%)	3,371 (7.3%)	0.00
GI bleeding events (inpatient)	402 (11.8%)	379 (11.2%)	0.02	2,224 (4.8%)	2,153 (4.7%)	0.00
GI bleeding events (outpatient)	406 (12.0%)	417 (12.3%)	-0.01	4,911 (10.6%)	4,910 (10.6%)	0.00
Hypertension	3,115 (91.8%)	3,116 (91.8%)	0.00	38,591 (83.5%)	38,614 (83.6%)	0.00
Ischemic Heart Disease	1,901 (56.0%)	1,853 (54.6%)	0.03	19,604 (42.4%)	19,620 (42.5%)	0.00
Liver disease	249 (7.3%)	255 (7.5%)	-0.01	2,595 (5.6%)	2,660 (5.8%)	-0.01
Malignancy	496 (14.6%)	515 (15.2%)	-0.02	8,419 (18.2%)	8,385 (18.1%)	0.00
Obesity	574 (16.9%)	555 (16.4%)	0.01	9,476 (20.5%)	9,437 (20.4%)	0.00
Peptic ulcer	98 (2.9%)	103 (3.0%)	-0.01	747 (1.6%)	782 (1.7%)	-0.01
PVD or PVD Surgery	803 (23.7%)	793 (23.4%)	0.01	5,545 (12.0%)	5,601 (12.1%)	0.00
Smoking	1,157 (34.1%)	1,151 (33.9%)	0.00	14,134 (30.6%)	14,078 (30.5%)	0.00

© 2023 Lin KJ et al. *JAMA Network Open*.

Characteristics<br>Number of Patients (%)	With Dementia			Without Dementia		
	Apixaban (n=3,393)	Dabigatran (n=3,393)	St. Diff	Apixaban (n=46,202)	Dabigatran (n=46,202)	St. Diff
Stroke (inpatient)	707 (20.8%)	708 (20.9%)	0.00	3,230 (7.0%)	3,234 (7.0%)	0.00
Stroke (outpatient)	554 (16.3%)	524 (15.4%)	0.02	3,617 (7.8%)	3,495 (7.6%)	0.01
Syncope	660 (19.5%)	658 (19.4%)	0.00	4,134 (8.9%)	4,147 (9.0%)	0.00
**Medications**						
ACE inhibitors	984 (29.0%)	1,006 (29.6%)	-0.01	12,261 (26.5%)	12,288 (26.6%)	0.00
Angiotensin II receptor blockers	240 (7.1%)	222 (6.5%)	0.02	3,058 (6.6%)	3,022 (6.5%)	0.00
Antiarrhythmic agents	574 (16.9%)	564 (16.6%)	0.01	10,297 (22.3%)	10,302 (22.3%)	0.00
Anticoagulants, injectable	44 (1.3%)	37 (1.1%)	0.02	344 (0.7%)	346 (0.7%)	0.00
Anticonvulsants	809 (23.8%)	818 (24.1%)	-0.01	7,063 (15.3%)	6,994 (15.1%)	0.01
Antidepressants - Other	662 (19.5%)	660 (19.5%)	0.00	3,325 (7.2%)	3,300 (7.1%)	0.00
Antidepressants - SSRI/SNRI	1,457 (42.9%)	1,413 (41.6%)	0.03	8,907 (19.3%)	8,817 (19.1%)	0.01
Antidepressants - Tricyclics	130 (3.8%)	121 (3.6%)	0.01	1,232 (2.7%)	1,228 (2.7%)	0.00
Antiplatelet agents	691 (20.4%)	624 (18.4%)	0.05	6,444 (13.9%)	6,333 (13.7%)	0.01
Antipsychotic agents	623 (18.4%)	615 (18.1%)	0.01	822 (1.8%)	836 (1.8%)	0.00
Anxiolytics - Benzodiazepines	879 (25.9%)	874 (25.8%)	0.00	8,130 (17.6%)	8,169 (17.7%)	0.00
Anxiolytics - Other	82 (2.4%)	94 (2.8%)	-0.03	476 (1.0%)	460 (1.0%)	0.00
Beta-blockers	2,279 (67.2%)	2,258 (66.5%)	0.01	30,142 (65.2%)	30,214 (65.4%)	0.00
Bronchodilators	775 (22.8%)	745 (22.0%)	0.02	8,715 (18.9%)	8,641 (18.7%)	0.01
Calcium channel blockers	87 (2.6%)	87 (2.6%)	0.00	964 (2.1%)	971 (2.1%)	0.00
Corticosteroids, inhaled	722 (21.3%)	700 (20.6%)	0.02	10,508 (22.7%)	10,479 (22.7%)	0.00
Corticosteroids, oral	1,015 (29.9%)	1,027 (30.3%)	-0.01	14,888 (32.2%)	14,960 (32.4%)	0.00
Dementia drugs	1,596 (47.0%)	1,616 (47.6%)	-0.01	867 (1.9%)	861 (1.9%)	0.00
Diabetes agents - Insulin	385 (11.3%)	389 (11.5%)	-0.01	3,140 (6.8%)	3,203 (6.9%)	0.00
Diabetes agents - Metformin	552 (16.3%)	566 (16.7%)	-0.01	7,702 (16.7%)	7,788 (16.9%)	-0.01
Diabetes agents - Other	231 (6.8%)	241 (7.1%)	-0.01	2,531 (5.5%)	2,580 (5.6%)	0.00
Diabetes agents - Sulfonylurea	372 (11.0%)	365 (10.8%)	0.01	4,414 (9.6%)	4,473 (9.7%)	0.00
Diuretics	1,994 (58.8%)	1,976 (58.2%)	0.01	24,710 (53.5%)	24,705 (53.5%)	0.00
Estrogen	84 (2.5%)	100 (2.9%)	-0.02	1,566 (3.4%)	1,569 (3.4%)	0.00
Fibrates	115 (3.4%)	122 (3.6%)	-0.01	2,138 (4.6%)	2,180 (4.7%)	0.00
GI - H2 blockers	471 (13.9%)	441 (13.0%)	0.03	3,355 (7.3%)	3,245 (7.0%)	0.01
GI - Proton-pump inhibitors	1,345 (39.6%)	1,351 (39.8%)	0.00	14,368 (31.1%)	14,439 (31.3%)	0.00
GI - Sucralfate	99 (2.9%)	91 (2.7%)	0.01	894 (1.9%)	904 (2.0%)	-0.01
Hypnotics	361 (10.6%)	372 (11.0%)	-0.01	4,133 (8.9%)	4,138 (9.0%)	0.00
Nitrates	566 (16.7%)	546 (16.1%)	0.02	5,390 (11.7%)	5,354 (11.6%)	0.00
NSAIDs	531 (15.6%)	535 (15.8%)	-0.01	7,722 (16.7%)	7,790 (16.9%)	-0.01
Opioids	1,421 (41.9%)	1,406 (41.4%)	0.01	17,893 (38.7%)	17,791 (38.5%)	0.00
Parkinsonism drugs	321 (9.5%)	302 (8.9%)	0.02	1,679 (3.6%)	1,677 (3.6%)	0.00
Statins	2,285 (67.3%)	2,273 (67.0%)	0.01	29,667 (64.2%)	29,657 (64.2%)	0.00
Thyroid hormone replacement	901 (26.6%)	929 (27.4%)	-0.02	9,928 (21.5%)	9,841 (21.3%)	0.00
**Health care use**						
Emergency department visits	1,820 (53.6%)	1,777 (52.4%)	0.02	16,624 (36.0%)	16,585 (35.9%)	0.00
Home health day[C]	568 (16.7%)	552 (16.3%)	0.01	2,202 (4.8%)	2,154 (4.7%)	0.00
Home oxygen use	165 (4.9%)	156 (4.6%)	0.01	1,223 (2.6%)	1,253 (2.7%)	-0.01
Hospitalizations	2,532 (74.6%)	2,559 (75.4%)	-0.02	19,264 (41.7%)	19,466 (42.1%)	-0.01
**Geographic Region**						
Northeast	791 (23.3%)	778 (22.9%)	0.01	10,186 (22.0%)	10,074 (21.8%)	0.00
Midwest	676 (19.9%)	694 (20.5%)	-0.01	10,032 (21.7%)	9,994 (21.6%)	0.00
South	1,450 (42.7%)	1,410 (41.6%)	0.02	18,183 (39.4%)	18,259 (39.5%)	0.00
West	466 (13.7%)	506 (14.9%)	-0.03	7,764 (16.8%)	7,831 (16.9%)	0.00
Other	<11	<11	-	37 (0.1%)	44 (0.1%)	0.00

Abbreviations: ACE, angiotensin converting enzyme; CCI, combined comorbidity; CFI, claims-based frailty index; CKD, chronic kidney disease; COPD, chronic obstructive pulmonary disease; GI, gastrointestinal; NSAID, non-steroidal anti-inflammatory drug; PVD, peripheral vascular disease; SNRI, serotonin-norepinephrine reuptake inhibitor; SSRI, selective serotonin reuptake inhibitor.

[A] Other race category includes Unknown, Other, Asian, Hispanic, North American Native, and Missing Race Categories in Medicare. Asian, Hispanic, Unknown, and Missing categories are included for race in Optum. Race is unavailable in the MarketScan database.

[B] With both Medicare are Medicaid enrollment eligibility

[C] Home Health services received (days)

© 2023 Lin KJ et al. *JAMA Network Open*.

eTable 22. Selected Characteristics of Propensity Score–Matched Population With Atrial Fibrillation Treated With Dabigatran vs Apixaban by Dementia Diagnosis in the Optum Population

Characteristics<br>Number of Patients (%)	With Dementia			Without Dementia		
	Apixaban (n=450)	Dabigatran (n=450)	St. Diff*	Apixaban (n=6,552)	Dabigatran (n=6,552)	St. Diff
**Quarter/Year of Cohort Entry Date**						
Q1 2013	0 (0.0%)	0 (0.0%)	-	36 (0.5%)	42 (0.6%)	-0.01
Q2 2013	10 (2.2%)	9 (2.0%)	0.01	181 (2.8%)	212 (3.2%)	-0.02
Q3 2013	9 (2.0%)	7 (1.6%)	0.03	233 (3.6%)	229 (3.5%)	0.01
Q4 2013	12 (2.7%)	11 (2.4%)	0.02	338 (5.2%)	328 (5.0%)	0.01
Q1 2014	21 (4.7%)	22 (4.9%)	-0.01	388 (5.9%)	369 (5.6%)	0.01
Q2 2014	21 (4.7%)	16 (3.6%)	0.06	313 (4.8%)	290 (4.4%)	0.02
Q3 2014	12 (2.7%)	13 (2.9%)	-0.01	202 (3.1%)	230 (3.5%)	-0.02
Q4 2014	10 (2.2%)	13 (2.9%)	-0.04	213 (3.3%)	209 (3.2%)	0.01
Q1 2015	23 (5.1%)	20 (4.4%)	0.03	254 (3.9%)	263 (4.0%)	-0.01
Q2 2015	8 (1.8%)	12 (2.7%)	-0.06	181 (2.8%)	200 (3.1%)	-0.02
Q3 2015	10 (2.2%)	12 (2.7%)	-0.03	187 (2.9%)	180 (2.7%)	0.01
Q4 2015	12 (2.7%)	14 (3.1%)	-0.02	227 (3.5%)	213 (3.3%)	0.01
Q1 2016	24 (5.3%)	25 (5.6%)	-0.01	400 (6.1%)	375 (5.7%)	0.02
Q2 2016	17 (3.8%)	16 (3.6%)	0.01	334 (5.1%)	298 (4.5%)	0.03
Q3 2016	18 (4.0%)	20 (4.4%)	-0.02	271 (4.1%)	267 (4.1%)	0.00
Q4 2016	20 (4.4%)	16 (3.6%)	0.04	260 (4.0%)	260 (4.0%)	0.00
Q1 2017	15 (3.3%)	19 (4.2%)	-0.05	243 (3.7%)	280 (4.3%)	-0.03
Q2 2017	13 (2.9%)	13 (2.9%)	0.00	220 (3.4%)	225 (3.4%)	0.00
Q3 2017	15 (3.3%)	17 (3.8%)	-0.03	190 (2.9%)	186 (2.8%)	0.01
Q4 2017	18 (4.0%)	16 (3.6%)	0.02	201 (3.1%)	188 (2.9%)	0.01
Q1 2018	22 (4.9%)	24 (5.3%)	-0.02	293 (4.5%)	300 (4.6%)	0.00
Q2 2018	13 (2.9%)	13 (2.9%)	0.00	204 (3.1%)	202 (3.1%)	0.00
Q3 2018	15 (3.3%)	17 (3.8%)	-0.03	189 (2.9%)	192 (2.9%)	0.00
Q4 2018	15 (3.3%)	12 (2.7%)	0.04	152 (2.3%)	162 (2.5%)	-0.01
Q1 2019	24 (5.3%)	22 (4.9%)	0.02	166 (2.5%)	162 (2.5%)	0.00
Q2 2019	11 (2.4%)	9 (2.0%)	0.03	129 (2.0%)	116 (1.8%)	0.01
Q3 2019	15 (3.3%)	10 (2.2%)	0.07	125 (1.9%)	124 (1.9%)	0.00
Q4 2019	5 (1.1%)	10 (2.2%)	-0.09	114 (1.7%)	110 (1.7%)	0.00
Q1 2020	0 (0.0%)	3 (0.7%)	-0.12	67 (1.0%)	60 (0.9%)	0.01
Q2 2020	8 (1.8%)	8 (1.8%)	0.00	30 (0.5%)	42 (0.6%)	-0.01
Q3 2020	6 (1.3%)	1 (0.2%)	0.13	27 (0.4%)	30 (0.5%)	-0.01
Q4 2020	1 (0.2%)	2 (0.4%)	-0.04	24 (0.4%)	23 (0.4%)	0.00
Q1 2021	19 (4.2%)	25 (5.6%)	-0.06	139 (2.1%)	154 (2.4%)	-0.02
Q2 2021	8 (1.8%)	3 (0.7%)	0.10	21 (0.3%)	31 (0.5%)	-0.03
**Patient Characteristics**						
Mean age (SD)	81.54 (5.75)	81.26 (5.94)	0.05	75.50 (6.38)	75.50 (6.43)	0.00
Male	215 (47.8%)	212 (47.1%)	0.01	3,700 (56.5%)	3,690 (56.3%)	0.00
Female	235 (52.2%)	238 (52.9%)	-0.01	2,852 (43.5%)	2,862 (43.7%)	0.00
Black	29 (6.4%)	39 (8.7%)	-0.09	472 (7.2%)	493 (7.5%)	-0.01
White	329 (73.1%)	334 (74.2%)	-0.02	4,976 (75.9%)	4,923 (75.1%)	0.02
Other[A]	92 (20.4%)	77 (17.1%)	0.08	1,104 (16.8%)	1,136 (17.3%)	-0.01
Mean CHA_2DS_2-VASc score (SD)	5.72 (1.60)	5.67 (1.59)	0.03	4.42 (1.63)	4.44 (1.64)	-0.01
Mean HAS-BLED score (SD)	2.60 (0.71)	2.65 (0.80)	-0.07	2.25 (0.69)	2.25 (0.68)	0.00
Mean CCI score (SD)	6.18 (3.05)	6.42 (3.09)	-0.08	3.09 (2.65)	3.13 (2.62)	-0.02
Mean CFI (SD)	0.29 (0.07)	0.30 (0.08)	-0.13	0.19 (0.06)	0.19 (0.06)	0.00
**Medical History**						
Acute myocardial infarction	98 (21.8%)	95 (21.1%)	0.02	552 (8.4%)	554 (8.5%)	0.00
Acute renal failure	33 (7.3%)	26 (5.8%)	0.06	304 (4.6%)	293 (4.5%)	0.00
Alcohol abuse or dependence	20 (4.4%)	17 (3.8%)	0.03	182 (2.8%)	172 (2.6%)	0.01
Anemia	138 (30.7%)	153 (34.0%)	-0.07	1,449 (22.1%)	1,451 (22.1%)	0.00
Cardio-ablation	1 (0.2%)	2 (0.4%)	-0.04	44 (0.7%)	47 (0.7%)	0.00
Cardioversion	10 (2.2%)	11 (2.4%)	-0.01	470 (7.2%)	459 (7.0%)	0.01
Cerebrovascular disease	222 (49.3%)	219 (48.7%)	0.01	1,446 (22.1%)	1,451 (22.1%)	0.00
CKD: stage 3, 4 or unspecified	122 (27.1%)	139 (30.9%)	-0.08	1,170 (17.9%)	1,173 (17.9%)	0.00

© 2023 Lin KJ et al. *JAMA Network Open*.

Characteristics Number of Patients (%)	With Dementia			Without Dementia		
	Apixaban (n=450)	Dabigatran (n=450)	St. Diff*	Apixaban (n=6,552)	Dabigatran (n=6,552)	St. Diff
Congestive heart failure (inpatient)	94 (20.9%)	101 (22.4%)	-0.04	709 (10.8%)	720 (11.0%)	-0.01
Congestive heart failure (outpatient)	212 (47.1%)	216 (48.0%)	-0.02	2,251 (34.4%)	2,326 (35.5%)	-0.02
Coronary revascularization	5 (1.1%)	5 (1.1%)	0.00	145 (2.2%)	148 (2.3%)	-0.01
COPD	110 (24.4%)	117 (26.0%)	-0.04	1,335 (20.4%)	1,370 (20.9%)	-0.01
Dementia	450 (100.0%)	450 (100.0%)	-	0 (0.0%)	0 (0.0%)	-
Diabetes	202 (44.9%)	195 (43.3%)	0.03	2,424 (37.0%)	2,437 (37.2%)	0.00
Endoscopy	17 (3.8%)	17 (3.8%)	0.00	70 (1.1%)	74 (1.1%)	0.00
Falls	127 (28.2%)	133 (29.6%)	-0.03	564 (8.6%)	582 (8.9%)	-0.01
Fractures	82 (18.2%)	82 (18.2%)	0.00	464 (7.1%)	448 (6.8%)	0.01
GI bleeding events (inpatient)	26 (5.8%)	19 (4.2%)	0.07	141 (2.2%)	147 (2.2%)	0.00
GI bleeding events (outpatient)	121 (26.9%)	121 (26.9%)	0.00	1,621 (24.7%)	1,627 (24.8%)	0.00
Hypertension	429 (95.3%)	428 (95.1%)	0.01	5,783 (88.3%)	5,817 (88.8%)	-0.02
Ischemic Heart Disease	223 (49.6%)	221 (49.1%)	0.01	2,839 (43.3%)	2,821 (43.1%)	0.00
Liver disease	30 (6.7%)	32 (7.1%)	-0.02	448 (6.8%)	435 (6.6%)	0.01
Malignancy	57 (12.7%)	67 (14.9%)	-0.06	1,023 (15.6%)	1,052 (16.1%)	-0.01
Obesity	53 (11.8%)	53 (11.8%)	0.00	1,342 (20.5%)	1,349 (20.6%)	0.00
Peptic ulcer	13 (2.9%)	15 (3.3%)	-0.02	111 (1.7%)	120 (1.8%)	-0.01
PVD or PVD Surgery	96 (21.3%)	100 (22.2%)	-0.02	861 (13.1%)	869 (13.3%)	-0.01
Smoking	122 (27.1%)	113 (25.1%)	0.05	1,596 (24.4%)	1,564 (23.9%)	0.01
Stroke (inpatient)	68 (15.1%)	67 (14.9%)	0.01	319 (4.9%)	347 (5.3%)	-0.02
Stroke (outpatient)	207 (46.0%)	202 (44.9%)	0.02	1,340 (20.5%)	1,348 (20.6%)	0.00
Syncope	82 (18.2%)	90 (20.0%)	-0.05	666 (10.2%)	637 (9.7%)	0.02
**Medications**						
ACE inhibitors	134 (29.8%)	130 (28.9%)	0.02	1,784 (27.2%)	1,761 (26.9%)	0.01
Angiotensin II receptor blockers	25 (5.6%)	27 (6.0%)	-0.02	350 (5.3%)	349 (5.3%)	0.00
Antiarrhythmic agents	67 (14.9%)	67 (14.9%)	0.00	1,381 (21.1%)	1,363 (20.8%)	0.01
Anticoagulants, injectable	9 (2.0%)	7 (1.6%)	0.03	24 (0.4%)	21 (0.3%)	0.02
Anticonvulsants	98 (21.8%)	95 (21.1%)	0.02	921 (14.1%)	930 (14.2%)	0.00
Antidepressants - Other	81 (18.0%)	86 (19.1%)	-0.03	447 (6.8%)	456 (7.0%)	-0.01
Antidepressants - SSRI/SNRI	154 (34.2%)	158 (35.1%)	-0.02	1,141 (17.4%)	1,126 (17.2%)	0.01
Antidepressants - Tricyclics	11 (2.4%)	8 (1.8%)	0.04	118 (1.8%)	130 (2.0%)	-0.01
Antiplatelet agents	54 (12.0%)	57 (12.7%)	-0.02	724 (11.1%)	731 (11.2%)	0.00
Antipsychotic agents	57 (12.7%)	65 (14.4%)	-0.05	100 (1.5%)	91 (1.4%)	0.01
Anxiolytics - Benzodiazepines	86 (19.1%)	87 (19.3%)	-0.01	1,043 (15.9%)	1,023 (15.6%)	0.01
Anxiolytics - Other	1 (0.2%)	3 (0.7%)	-0.07	70 (1.1%)	67 (1.0%)	0.01
Beta-blockers	303 (67.3%)	304 (67.6%)	-0.01	4,113 (62.8%)	4,114 (62.8%)	0.00
Bronchodilators	80 (17.8%)	86 (19.1%)	-0.03	1,200 (18.3%)	1,221 (18.6%)	-0.01
Calcium channel blockers	1 (0.2%)	1 (0.2%)	0.00	5 (0.1%)	7 (0.1%)	0.00
Corticosteroids, inhaled	75 (16.7%)	74 (16.4%)	0.01	1,244 (19.0%)	1,274 (19.4%)	-0.01
Corticosteroids, oral	119 (26.4%)	118 (26.2%)	0.00	1,900 (29.0%)	1,906 (29.1%)	0.00
Dementia drugs	214 (47.6%)	199 (44.2%)	0.07	93 (1.4%)	96 (1.5%)	-0.01
Diabetes agents - Insulin	52 (11.6%)	48 (10.7%)	0.03	475 (7.2%)	468 (7.1%)	0.00
Diabetes agents - Metformin	66 (14.7%)	66 (14.7%)	0.00	1,180 (18.0%)	1,203 (18.4%)	-0.01
Diabetes agents - Other	29 (6.4%)	30 (6.7%)	-0.01	316 (4.8%)	323 (4.9%)	0.00
Diabetes agents - Sulfonylurea	41 (9.1%)	33 (7.3%)	0.07	630 (9.6%)	635 (9.7%)	0.00
Diuretics	227 (50.4%)	233 (51.8%)	-0.03	3,232 (49.3%)	3,244 (49.5%)	0.00
Estrogen	7 (1.6%)	7 (1.6%)	0.00	165 (2.5%)	166 (2.5%)	0.00
Fibrates	20 (4.4%)	21 (4.7%)	-0.01	329 (5.0%)	308 (4.7%)	0.01
GI - H2 blockers	32 (7.1%)	39 (8.7%)	-0.06	409 (6.2%)	388 (5.9%)	0.01
GI - Proton-pump inhibitors	149 (33.1%)	141 (31.3%)	0.04	1,729 (26.4%)	1,760 (26.9%)	-0.01
GI - Sucralfate	5 (1.1%)	7 (1.6%)	-0.04	98 (1.5%)	92 (1.4%)	0.01
Hypnotics	25 (5.6%)	26 (5.8%)	-0.01	486 (7.4%)	460 (7.0%)	0.02
Nitrates	50 (11.1%)	51 (11.3%)	-0.01	562 (8.6%)	615 (9.4%)	-0.03
NSAIDs	55 (12.2%)	58 (12.9%)	-0.02	893 (13.6%)	930 (14.2%)	-0.02
Opioids	161 (35.8%)	169 (37.6%)	-0.04	2,203 (33.6%)	2,235 (34.1%)	-0.01
Parkinsonism drugs	21 (4.7%)	30 (6.7%)	-0.09	192 (2.9%)	201 (3.1%)	-0.01
Statins	293 (65.1%)	285 (63.3%)	0.04	3,943 (60.2%)	3,962 (60.5%)	-0.01
Thyroid hormone replacement	110 (24.4%)	106 (23.6%)	0.02	1,255 (19.2%)	1,248 (19.0%)	0.01
**Health care use**						
Emergency department visits	307 (68.2%)	312 (69.3%)	-0.02	2,995 (45.7%)	2,983 (45.5%)	0.00

© 2023 Lin KJ et al. *JAMA Network Open*.

Characteristics Number of Patients (%)	With Dementia			Without Dementia		
	Apixaban (n=450)	Dabigatran (n=450)	St. Diff*	Apixaban (n=6,552)	Dabigatran (n=6,552)	St. Diff
Home oxygen use	24 (5.3%)	25 (5.6%)	-0.01	182 (2.8%)	177 (2.7%)	0.01
Hospitalizations	258 (57.3%)	272 (60.4%)	-0.06	2,233 (34.1%)	2,221 (33.9%)	0.00
**Geographic Region**						
Northeast	81 (18.0%)	77 (17.1%)	0.02	998 (15.2%)	1,013 (15.5%)	-0.01
Midwest	64 (14.2%)	67 (14.9%)	-0.02	1,125 (17.2%)	1,100 (16.8%)	0.01
South	170 (37.8%)	180 (40.0%)	-0.05	2,672 (40.8%)	2,603 (39.7%)	0.02
West	134 (29.8%)	125 (27.8%)	0.04	1,753 (26.8%)	1,830 (27.9%)	-0.02
Other	1 (0.2%)	1 (0.2%)	0.00	4 (0.1%)	6 (0.1%)	0.00
**Business Type Code (Recategorized)**						
Commercial	21 (4.7%)	25 (5.6%)	-0.04	860 (13.1%)	842 (12.9%)	0.01
Medicare	429 (95.3%)	425 (94.4%)	0.04	5,692 (86.9%)	5,710 (87.1%)	-0.01

Abbreviations: ACE, angiotensin converting enzyme; CCI, combined comorbidity; CFI, claims-based frailty index; CKD, chronic kidney disease; COPD, chronic obstructive pulmonary disease; GI, gastrointestinal; NSAID, non-steroidal anti-inflammatory drug; PVD, peripheral vascular disease; SNRI, serotonin-norepinephrine reuptake inhibitor; SSRI, selective serotonin reuptake inhibitor.

[A] Other race category includes Unknown, Other, Asian, Hispanic, North American Native, and Missing Race Categories in Medicare. Asian, Hispanic, Unknown, and Missing categories are included for race in Optum. Race is unavailable in the MarketScan database.

* For the variables with standardized differences >=0.1, we additional adjust them in the outcome Cox proportional hazards model

© 2023 Lin KJ et al. *JAMA Network Open*.

eTable 23. Selected Characteristics of Propensity Score–Matched Population With Atrial Fibrillation Treated With Dabigatran vs Apixaban by Dementia Diagnosis in the MarketScan Population

Characteristics<br>Number of Patients (%)	With Dementia			Without Dementia		
	Apixaban (n=330)	Dabigatran (n=330)	St. Diff*	Apixaban (n=6,337)	Dabigatran (n=6,337)	St. Diff
**Quarter/Year of Cohort Entry Date**						
Q1 2013	3 (0.9%)	4 (1.2%)	0.00	94 (1.5%)	100 (1.6%)	0.00
Q2 2013	20 (6.1%)	17 (5.2%)	0.01	476 (7.5%)	507 (8.0%)	-0.01
Q3 2013	25 (7.6%)	24 (7.3%)	0.00	636 (10.0%)	632 (10.0%)	0.00
Q4 2013	31 (9.4%)	34 (10.3%)	-0.01	659 (10.4%)	641 (10.1%)	0.00
Q1 2014	16 (4.8%)	20 (6.1%)	-0.01	459 (7.2%)	465 (7.3%)	0.00
Q2 2014	26 (7.9%)	18 (5.5%)	0.02	422 (6.7%)	402 (6.3%)	0.00
Q3 2014	23 (7.0%)	27 (8.2%)	-0.01	350 (5.5%)	359 (5.7%)	0.00
Q4 2014	16 (4.8%)	14 (4.2%)	0.01	271 (4.3%)	289 (4.6%)	0.00
Q1 2015	14 (4.2%)	14 (4.2%)	0.00	210 (3.3%)	233 (3.7%)	0.00
Q2 2015	8 (2.4%)	12 (3.6%)	-0.01	190 (3.0%)	174 (2.7%)	0.00
Q3 2015	13 (3.9%)	9 (2.7%)	0.01	186 (2.9%)	186 (2.9%)	0.00
Q4 2015	14 (4.2%)	15 (4.5%)	0.00	249 (3.9%)	260 (4.1%)	0.00
Q1 2016	26 (7.9%)	21 (6.4%)	0.02	294 (4.6%)	295 (4.7%)	0.00
Q2 2016	14 (4.2%)	16 (4.8%)	-0.01	342 (5.4%)	311 (4.9%)	0.01
Q3 2016	21 (6.4%)	15 (4.5%)	0.02	276 (4.4%)	277 (4.4%)	0.00
Q4 2016	11 (3.3%)	18 (5.5%)	-0.02	279 (4.4%)	259 (4.1%)	0.00
Q1 2017	6 (1.8%)	9 (2.7%)	-0.01	145 (2.3%)	147 (2.3%)	0.00
Q2 2017	13 (3.9%)	8 (2.4%)	0.02	146 (2.3%)	137 (2.2%)	0.00
Q3 2017	7 (2.1%)	6 (1.8%)	0.00	98 (1.5%)	105 (1.7%)	0.00
Q4 2017	3 (0.9%)	7 (2.1%)	-0.01	116 (1.8%)	111 (1.8%)	0.00
Q1 2018	3 (0.9%)	2 (0.6%)	0.00	103 (1.6%)	90 (1.4%)	0.00
Q2 2018	4 (1.2%)	3 (0.9%)	0.00	80 (1.3%)	69 (1.1%)	0.00
Q3 2018	0 (0.0%)	1 (0.3%)	0.00	31 (0.5%)	42 (0.7%)	0.00
Q4 2018	2 (0.6%)	2 (0.6%)	0.00	57 (0.9%)	57 (0.9%)	0.00
Q1 2019	2 (0.6%)	4 (1.2%)	-0.01	33 (0.5%)	31 (0.5%)	0.00
Q2 2019	2 (0.6%)	3 (0.9%)	0.00	27 (0.4%)	36 (0.6%)	0.00
Q3 2019	2 (0.6%)	2 (0.6%)	0.00	35 (0.6%)	43 (0.7%)	0.00
Q4 2019	1 (0.3%)	1 (0.3%)	0.00	20 (0.3%)	23 (0.4%)	0.00
Q1 2020	0 (0.0%)	0 (0.0%)	0.00	17 (0.3%)	17 (0.3%)	0.00
Q2 2020	4 (1.2%)	2 (0.6%)	0.01	7 (0.1%)	9 (0.1%)	0.00
Q3 2020	0 (0.0%)	2 (0.6%)	-0.01	14 (0.2%)	17 (0.3%)	0.00
Q4 2020	0 (0.0%)	0 (0.0%)	0.00	15 (0.2%)	13 (0.2%)	0.00
**Patient Characteristics**						
Mean age (SD)	81.93 (6.54)	82.44 (6.51)	-0.51	76.09 (7.49)	75.96 (7.23)	0.13
Male	163 (49.4%)	158 (47.9%)	0.02	3,688 (58.2%)	3,655 (57.7%)	0.01
Female	167 (50.6%)	172 (52.1%)	-0.02	2,649 (41.8%)	2,682 (42.3%)	-0.01
Mean CHA2DS2-VASc score (SD)	4.68 (1.51)	4.68 (1.53)	0.00	3.63 (1.51)	3.63 (1.50)	0.00
Mean HAS-BLED score (SD)	2.51 (0.79)	2.45 (0.76)	0.06	2.11 (0.65)	2.11 (0.66)	0.00
Mean CCI score (SD)	5.31 (3.19)	5.32 (3.01)	-0.01	2.47 (2.32)	2.45 (2.34)	0.02
Mean CFI (SD)	0.29 (0.07)	0.29 (0.07)	0.00	0.18 (0.05)	0.18 (0.05)	0.00
**Medical History**						
Acute myocardial infarction	22 (6.7%)	19 (5.8%)	0.01	289 (4.6%)	287 (4.5%)	0.00
Acute renal failure	56 (17.0%)	59 (17.9%)	-0.01	389 (6.1%)	403 (6.4%)	0.00
Alcohol abuse or dependence	8 (2.4%)	6 (1.8%)	0.01	103 (1.6%)	103 (1.6%)	0.00
Anemia	109 (33.0%)	97 (29.4%)	0.04	1,064 (16.8%)	1,037 (16.4%)	0.00
Cardio-ablation	0 (0.0%)	0 (0.0%)	0.00	58 (0.9%)	50 (0.8%)	0.00
Cardioversion	13 (3.9%)	12 (3.6%)	0.00	462 (7.3%)	475 (7.5%)	0.00
Cerebrovascular disease	162 (49.1%)	160 (48.5%)	0.01	1,413 (22.3%)	1,383 (21.8%)	0.01
CKD: stage 3, 4 or unspecified	71 (21.5%)	66 (20.0%)	0.02	687 (10.8%)	674 (10.6%)	0.00
Congestive heart failure (inpatient)	78 (23.6%)	84 (25.5%)	-0.02	875 (13.8%)	881 (13.9%)	0.00
Congestive heart failure (outpatient)	126 (38.2%)	132 (40.0%)	-0.02	1,679 (26.5%)	1,660 (26.2%)	0.00
Coronary revascularization	5 (1.5%)	5 (1.5%)	0.00	119 (1.9%)	132 (2.1%)	0.00
COPD	71 (21.5%)	84 (25.5%)	-0.04	1,091 (17.2%)	1,135 (17.9%)	-0.01
Dementia	330 (100.0%)	330 (100.0%)	0.00	0 (0.0%)	0 (0.0%)	0.00

© 2023 Lin KJ et al. *JAMA Network Open*.

Characteristics<br>Number of Patients (%)	With Dementia			Without Dementia		
	Apixaban (n=330)	Dabigatran (n=330)	St. Diff*	Apixaban (n=6,337)	Dabigatran (n=6,337)	St. Diff
Diabetes	139 (42.1%)	135 (40.9%)	0.01	2,020 (31.9%)	2,010 (31.7%)	0.00
Endoscopy	10 (3.0%)	10 (3.0%)	0.00	61 (1.0%)	62 (1.0%)	0.00
Falls	58 (17.6%)	58 (17.6%)	0.00	297 (4.7%)	296 (4.7%)	0.00
Fractures	46 (13.9%)	49 (14.8%)	-0.01	385 (6.1%)	408 (6.4%)	0.00
GI bleeding events (inpatient)	23 (7.0%)	19 (5.8%)	0.01	186 (2.9%)	182 (2.9%)	0.00
GI bleeding events (outpatient)	53 (16.1%)	48 (14.5%)	0.02	891 (14.1%)	897 (14.2%)	0.00
Hypertension	300 (90.9%)	291 (88.2%)	0.03	5,307 (83.7%)	5,324 (84.0%)	0.00
Ischemic Heart Disease	158 (47.9%)	164 (49.7%)	-0.02	2,690 (42.4%)	2,663 (42.0%)	0.00
Liver disease	20 (6.1%)	20 (6.1%)	0.00	310 (4.9%)	323 (5.1%)	0.00
Malignancy	59 (17.9%)	60 (18.2%)	0.00	1,178 (18.6%)	1,185 (18.7%)	0.00
Obesity	29 (8.8%)	25 (7.6%)	0.01	783 (12.4%)	821 (13.0%)	-0.01
Peptic ulcer	4 (1.2%)	4 (1.2%)	0.00	48 (0.8%)	53 (0.8%)	0.00
PVD or PVD Surgery	69 (20.9%)	71 (21.5%)	-0.01	760 (12.0%)	738 (11.6%)	0.00
Smoking	41 (12.4%)	39 (11.8%)	0.01	673 (10.6%)	696 (11.0%)	0.00
Stroke (inpatient)	65 (19.7%)	66 (20.0%)	0.00	415 (6.5%)	417 (6.6%)	0.00
Stroke (outpatient)	134 (40.6%)	129 (39.1%)	0.02	1,157 (18.3%)	1,142 (18.0%)	0.00
Syncope	82 (24.8%)	72 (21.8%)	0.03	567 (8.9%)	577 (9.1%)	0.00
**Medications**						
ACE inhibitors	112 (33.9%)	104 (31.5%)	0.02	1,540 (24.3%)	1,573 (24.8%)	-0.01
Angiotensin II receptor blockers	25 (7.6%)	19 (5.8%)	0.02	460 (7.3%)	445 (7.0%)	0.00
Antiarrhythmic agents	50 (15.2%)	49 (14.8%)	0.00	1,312 (20.7%)	1,320 (20.8%)	0.00
Anticoagulants, injectable	3 (0.9%)	3 (0.9%)	0.00	46 (0.7%)	43 (0.7%)	0.00
Anticonvulsants	72 (21.8%)	66 (20.0%)	0.02	785 (12.4%)	804 (12.7%)	0.00
Antidepressants - Other	51 (15.5%)	54 (16.4%)	-0.01	349 (5.5%)	370 (5.8%)	0.00
Antidepressants - SSRI/SNRI	131 (39.7%)	122 (37.0%)	0.03	1,046 (16.5%)	1,045 (16.5%)	0.00
Antidepressants - Tricyclics	11 (3.3%)	9 (2.7%)	0.01	139 (2.2%)	138 (2.2%)	0.00
Antiplatelet agents	58 (17.6%)	57 (17.3%)	0.00	922 (14.5%)	896 (14.1%)	0.00
Antipsychotic agents	46 (13.9%)	46 (13.9%)	0.00	90 (1.4%)	93 (1.5%)	0.00
Anxiolytics - Benzodiazepines	74 (22.4%)	62 (18.8%)	0.04	1,021 (16.1%)	1,007 (15.9%)	0.00
Anxiolytics - Other	7 (2.1%)	7 (2.1%)	0.00	54 (0.9%)	56 (0.9%)	0.00
Beta-blockers	215 (65.2%)	213 (64.5%)	0.01	4,062 (64.1%)	4,062 (64.1%)	0.00
Bronchodilators	47 (14.2%)	53 (16.1%)	-0.02	1,032 (16.3%)	1,132 (17.9%)	-0.02
Calcium channel blockers	0 (0.0%)	0 (0.0%)	0.00	15 (0.2%)	13 (0.2%)	0.00
Corticosteroids, inhaled	51 (15.5%)	48 (14.5%)	0.01	1,287 (20.3%)	1,374 (21.7%)	-0.01
Corticosteroids, oral	87 (26.4%)	84 (25.5%)	0.01	1,838 (29.0%)	1,877 (29.6%)	-0.01
Dementia drugs	185 (56.1%)	182 (55.2%)	0.01	109 (1.7%)	111 (1.8%)	0.00
Diabetes agents - Insulin	34 (10.3%)	33 (10.0%)	0.00	461 (7.3%)	456 (7.2%)	0.00
Diabetes agents - Metformin	49 (14.8%)	46 (13.9%)	0.01	1,006 (15.9%)	1,009 (15.9%)	0.00
Diabetes agents - Other	22 (6.7%)	23 (7.0%)	0.00	391 (6.2%)	401 (6.3%)	0.00
Diabetes agents - Sulfonylurea	36 (10.9%)	30 (9.1%)	0.02	579 (9.1%)	561 (8.9%)	0.00
Diuretics	170 (51.5%)	178 (53.9%)	-0.02	3,102 (49.0%)	3,114 (49.1%)	0.00
Estrogen	15 (4.5%)	7 (2.1%)	0.02	215 (3.4%)	227 (3.6%)	0.00
Fibrates	19 (5.8%)	13 (3.9%)	0.02	304 (4.8%)	298 (4.7%)	0.00
GI - H2 blockers	28 (8.5%)	27 (8.2%)	0.00	322 (5.1%)	351 (5.5%)	-0.01
GI - Proton-pump inhibitors	94 (28.5%)	94 (28.5%)	0.00	1,826 (28.8%)	1,799 (28.4%)	0.00
GI - Sucralfate	10 (3.0%)	9 (2.7%)	0.00	101 (1.6%)	98 (1.5%)	0.00
Hypnotics	28 (8.5%)	26 (7.9%)	0.01	502 (7.9%)	526 (8.3%)	0.00
Nitrates	35 (10.6%)	33 (10.0%)	0.01	623 (9.8%)	635 (10.0%)	0.00
NSAIDs	35 (10.6%)	37 (11.2%)	-0.01	975 (15.4%)	995 (15.7%)	0.00
Opioids	124 (37.6%)	122 (37.0%)	0.01	2,435 (38.4%)	2,442 (38.5%)	0.00
Parkinsonism drugs	30 (9.1%)	23 (7.0%)	0.02	218 (3.4%)	202 (3.2%)	0.00
Statins	212 (64.2%)	213 (64.5%)	0.00	3,896 (61.5%)	3,934 (62.1%)	-0.01
Thyroid hormone replacement	86 (26.1%)	85 (25.8%)	0.00	1,269 (20.0%)	1,249 (19.7%)	0.00
**Health care use**						
Emergency department visits	244 (73.9%)	245 (74.2%)	0.00	2,832 (44.7%)	2,874 (45.4%)	-0.01
Hospitalizations	201 (60.9%)	208 (63.0%)	-0.02	2,343 (37.0%)	2,435 (38.4%)	-0.02
**Geographic Region**						
Northeast	63 (19.1%)	59 (17.9%)	0.01	1,752 (27.6%)	1,750 (27.6%)	0.00
Midwest	103 (31.2%)	109 (33.0%)	-0.02	1,603 (25.3%)	1,691 (26.7%)	-0.01
South	111 (33.6%)	114 (34.5%)	-0.01	2,093 (33.0%)	2,015 (31.8%)	0.01

© 2023 Lin KJ et al. *JAMA Network Open*.

Characteristics Number of Patients (%)	With Dementia			Without Dementia		
	Apixaban (n=330)	Dabigatran (n=330)	St. Diff*	Apixaban (n=6,337)	Dabigatran (n=6,337)	St. Diff
West	52 (15.8%)	47 (14.2%)	0.02	868 (13.7%)	858 (13.5%)	0.00
Other	1 (0.3%)	1 (0.3%)	0.00	21 (0.3%)	23 (0.4%)	0.00
**Employee Classification (Recategorized), %**						
Salary Non-union	35 (10.6%)	28 (8.5%)	0.02	631 (10.0%)	642 (10.1%)	0.00
Salary Union	15 (4.5%)	8 (2.4%)	0.02	129 (2.0%)	142 (2.2%)	0.00
Salary Other	7 (2.1%)	7 (2.1%)	0.00	132 (2.1%)	143 (2.3%)	0.00
Hourly Non-Union	15 (4.5%)	17 (5.2%)	-0.01	376 (5.9%)	373 (5.9%)	0.00
Hourly Union	108 (32.7%)	126 (38.2%)	-0.06	1,777 (28.0%)	1,772 (28.0%)	0.00
Hourly Other	5 (1.5%)	4 (1.2%)	0.00	60 (0.9%)	64 (1.0%)	0.00
Non-Union	55 (16.7%)	52 (15.8%)	0.01	1,381 (21.8%)	1,344 (21.2%)	0.01
Union	0 (0.0%)	1 (0.3%)	0.00	105 (1.7%)	105 (1.7%)	0.00
Unknown	90 (27.3%)	87 (26.4%)	0.01	1,746 (27.6%)	1,752 (27.6%)	0.00
**Employment Status (Recategorized), %**						
Active Full Time	2 (0.6%)	4 (1.2%)	-0.01	413 (6.5%)	420 (6.6%)	0.00
Active Part Time or Seasonal	0 (0.0%)	0 (0.0%)	0.00	8 (0.1%)	14 (0.2%)	0.00
Early Retiree	0 (0.0%)	0 (0.0%)	0.00	66 (1.0%)	67 (1.1%)	0.00
Medicare Eligible Retiree	238 (72.1%)	231 (70.0%)	0.02	4,488 (70.8%)	4,443 (70.1%)	0.01
Retiree	11 (3.3%)	13 (3.9%)	-0.01	186 (2.9%)	184 (2.9%)	0.00
COBRA Continuee	0 (0.0%)	0 (0.0%)	0.00	6 (0.1%)	4 (0.1%)	0.00
Long Term Disability	0 (0.0%)	0 (0.0%)	0.00	8 (0.1%)	6 (0.1%)	0.00
Surviving Spouse/Dependent	41 (12.4%)	48 (14.5%)	-0.02	429 (6.8%)	450 (7.1%)	0.00
Other/Unknown/Missing	38 (11.5%)	34 (10.3%)	0.01	733 (11.6%)	749 (11.8%)	0.00
**Health Plan Indicator, %**						
Employer	290 (87.9%)	295 (89.4%)	-0.02	5,548 (87.5%)	5,530 (87.3%)	0.00
Health Plan	40 (12.1%)	35 (10.6%)	0.02	789 (12.5%)	807 (12.7%)	0.00
**MHSA Coverage Indicator, %**						
Not Covered/Claims Not Present	10 (3.0%)	11 (3.3%)	0.00	299 (4.7%)	321 (5.1%)	0.00
Covered/Possible MHSA Claims	291 (88.2%)	290 (87.9%)	0.00	5,633 (88.9%)	5,586 (88.1%)	0.01
Missing	29 (8.8%)	29 (8.8%)	0.00	405 (6.4%)	430 (6.8%)	0.00
**Plan Indicator, %**						
Basic/major medical	0 (0.0%)	0 (0.0%)	0.00	0 (0.0%)	0 (0.0%)	0.00
Comprehensive	166 (50.3%)	163 (49.4%)	0.01	2,400 (37.9%)	2,411 (38.0%)	0.00
EPO	0 (0.0%)	0 (0.0%)	0.00	21 (0.3%)	16 (0.3%)	0.00
HMO	18 (5.5%)	19 (5.8%)	0.00	482 (7.6%)	517 (8.2%)	-0.01
POS	9 (2.7%)	14 (4.2%)	-0.02	287 (4.5%)	271 (4.3%)	0.00
PPO	123 (37.3%)	124 (37.6%)	0.00	2,927 (46.2%)	2,916 (46.0%)	0.00
POS with capitation	11 (3.3%)	8 (2.4%)	0.01	78 (1.2%)	83 (1.3%)	0.00
CDHP	0 (0.0%)	0 (0.0%)	0.00	52 (0.8%)	38 (0.6%)	0.00
HDHP	0 (0.0%)	1 (0.3%)	0.00	20 (0.3%)	22 (0.3%)	0.00
Missing	3 (0.9%)	1 (0.3%)	0.01	70 (1.1%)	63 (1.0%)	0.00

Abbreviations: ACE, angiotensin converting enzyme; CCI, combined comorbidity; CFI, claims-based frailty index; CKD, chronic kidney disease; COPD, chronic obstructive pulmonary disease; GI, gastrointestinal; NSAID, non-steroidal anti-inflammatory drug; PVD, peripheral vascular disease; SNRI, serotonin-norepinephrine reuptake inhibitor; SSRI, selective serotonin reuptake inhibitor.

* For the variables with standardized differences >=0.1, we additional adjust them in the outcome Cox proportional hazards model

© 2023 Lin KJ et al. *JAMA Network Open*.

eTable 24. Selected Characteristics of Propensity Score–Matched Population With Atrial Fibrillation Treated With Rivaroxaban vs Apixaban by Dementia Diagnosis Pooled Across Medicare, Optum, and MarketScan Populations

Characteristics Number of Patients (%)	With Dementia			Without Dementia		
	Apixaban (n=19,497)	Rivaroxaban (n=19,497)	St. Diff	Apixaban (n=246,199)	Rivaroxaban (n=246,199)	St. Diff
**Patient Characteristics**						
Mean Age (SD)	82.25 (6.90)	82.25 (6.91)	0.00	76.40 (7.1)	76.44 (7.01)	-0.01
Male	7,643 (39.2%)	7,638 (39.2%)	0.00	126,083 (51.2%)	125,806 (51.1%)	0.00
Female	11,854 (60.8%)	11,859 (60.8%)	0.00	120,116 (48.8%)	120,393 (48.9%)	0.00
Black	1,336 (7.4%)	1,358 (7.5%)	0.00	10,482 (4.7%)	10,487 (4.7%)	0.00
White	15,353 (84.6%)	15,326 (84.5%)	0.00	194,947 (87.8%)	194,881 (87.8%)	0.00
Other[A]	1,459 (8.0%)	1,464 (8.1%)	0.00	16,489 (7.4%)	16,550 (7.5%)	0.00
Dual Status[B]	2,861 (19.2%)	2,849 (19.2%)	0.00	7,829 (4.4%)	7,830 (4.4%)	0.00
Mean CHA2DS2-VASc score (SD)	5.66 (1.61)	5.66 (1.62)	0.00	4.32 (1.6)	4.33 (1.65)	-0.01
Mean HAS-BLED score (SD)	2.64 (0.77)	2.65 (0.77)	0.00	2.20 (0.7)	2.21 (0.69)	-0.01
Mean CCI score (SD)	6.13 (2.98)	6.13 (2.98)	0.00	2.85 (2.5)	2.86 (2.52)	0.00
Mean CFI (SD)	0.31 (0.08)	0.31 (0.08)	0.00	0.19 (0.1)	0.19 (0.06)	0.00
**Cardiovascular conditions, %**						
Acute Myocardial infarction	1,648 (8.5%)	1,709 (8.8%)	-0.01	12,604 (5.1%)	12,858 (5.2%)	0.00
Cardio-ablation	85 (0.4%)	97 (0.5%)	-0.01	1,642 (0.7%)	1,594 (0.6%)	0.00
Cardioversion	624 (3.2%)	598 (3.1%)	0.01	17,079 (6.9%)	17,201 (7.0%)	0.00
Cerebrovascular disease	9,189 (47.1%)	9,224 (47.3%)	0.00	55,294 (22.5%)	55,431 (22.5%)	0.00
Congestive heart failure (inpatient)	6,317 (32.4%)	6,284 (32.2%)	0.00	38,184 (15.5%)	38,622 (15.7%)	0.00
Congestive heart failure (outpatient)	8,683 (44.5%)	8,642 (44.3%)	0.00	68,485 (27.8%)	68,800 (27.9%)	0.00
Coronary revascularization	397 (2.0%)	396 (2.0%)	0.00	6,947 (2.8%)	7,064 (2.9%)	0.00
Hypertension	17,937 (92.0%)	17,918 (91.9%)	0.00	208,359 (84.6%)	208,476 (84.7%)	0.00
Ischemic Heart Disease	10,094 (51.8%)	10,119 (51.9%)	0.00	102,772 (41.7%)	103,205 (41.9%)	0.00
PVD or PVD Surgery	4,689 (24.0%)	4,705 (24.1%)	0.00	30,550 (12.4%)	30,570 (12.4%)	0.00
Stroke (inpatient)	3,448 (17.7%)	3,503 (18.0%)	-0.01	14,890 (6.0%)	15,157 (6.2%)	0.00
Stroke (outpatient)	3,867 (19.8%)	3,893 (20.0%)	0.00	25,476 (10.3%)	25,572 (10.4%)	0.00
Syncope	3,843 (19.7%)	3,796 (19.5%)	0.01	23,669 (9.6%)	23,740 (9.6%)	0.00
**Non-cardiovascular conditions, %**						
Acute renal failure	4,604 (23.6%)	4,695 (24.1%)	-0.01	23,607 (9.6%)	23,995 (9.7%)	-0.01
Alcohol abuse or dependence	657 (3.4%)	649 (3.3%)	0.00	4,977 (2.0%)	4,983 (2.0%)	0.00
Anemia	8,352 (42.8%)	8,379 (43.0%)	0.00	56,618 (23.0%)	56,786 (23.1%)	0.00
CKD: stage 3, 4 or unspecified	4,851 (24.9%)	4,835 (24.8%)	0.00	32,470 (13.2%)	32,630 (13.3%)	0.00
COPD	5,913 (30.3%)	5,865 (30.1%)	0.01	50,804 (20.6%)	50,714 (20.6%)	0.00
Dementia	19,497 (100.0%)	19,497 (100.0%)		0 (0.0%)	0 (0.0%)	
Diabetes	8,211 (42.1%)	8,166 (41.9%)	0.00	84,691 (34.4%)	84,795 (34.4%)	0.00
Endoscopy	708 (3.6%)	696 (3.6%)	0.00	3,706 (1.5%)	3,734 (1.5%)	0.00
Falls	3,518 (18.0%)	3,614 (18.5%)	-0.01	10,972 (4.5%)	11,080 (4.5%)	0.00
Fractures	3,682 (18.9%)	3,726 (19.1%)	-0.01	18,800 (7.6%)	18,823 (7.6%)	0.00
GI bleeding (inpatient)	1,876 (9.6%)	1,877 (9.6%)	0.00	9,926 (4.0%)	10,002 (4.1%)	0.00
GI bleeding (outpatient)	2,735 (14.0%)	2,664 (13.7%)	0.01	30,934 (12.6%)	30,909 (12.6%)	0.00
Liver disease	1,372 (7.0%)	1,334 (6.8%)	0.01	15,092 (6.1%)	15,051 (6.1%)	0.00
Malignancy	2,920 (15.0%)	2,886 (14.8%)	0.00	44,166 (17.9%)	44,173 (17.9%)	0.00
Obesity	3,047 (15.6%)	3,071 (15.8%)	0.00	50,312 (20.4%)	50,194 (20.4%)	0.00
Peptic Ulcer	649 (3.3%)	648 (3.3%)	0.00	4,607 (1.9%)	4,622 (1.9%)	0.00
Smoking	6,591 (33.8%)	6,588 (33.8%)	0.00	74,850 (30.4%)	74,771 (30.4%)	0.00
**Cardiovascular medications**						
ACE inhibitors	5,771 (29.6%)	5,768 (29.6%)	0.00	65,357 (26.5%)	65,368 (26.6%)	0.00
Angiotensin II receptor blockers	1,109 (5.7%)	1,111 (5.7%)	0.00	14,804 (6.0%)	14,772 (6.0%)	0.00
Antiarrhythmic agents	3,085 (15.8%)	3,053 (15.7%)	0.00	48,698 (19.8%)	48,966 (19.9%)	0.00
Anticoagulants, injectable	300 (1.5%)	277 (1.4%)	0.01	1,498 (0.6%)	1,500 (0.6%)	0.00
Antiplatelet agent	3,741 (19.2%)	3,763 (19.3%)	0.00	35,422 (14.4%)	35,650 (14.5%)	0.00
Beta-blockers	12,986 (66.6%)	12,971 (66.5%)	0.00	162,651 (66.1%)	162,739 (66.1%)	0.00
Calcium channel blockers	374 (1.9%)	382 (2.0%)	0.00	3,882 (1.6%)	3,923 (1.6%)	0.00
Diuretics	10,841 (55.6%)	10,832 (55.6%)	0.00	127,020 (51.6%)	127,423 (51.8%)	0.00
Fibrates	694 (3.6%)	674 (3.5%)	0.01	10,944 (4.4%)	10,859 (4.4%)	0.00
Nitrates	2,621 (13.4%)	2,627 (13.5%)	0.00	26,187 (10.6%)	26,411 (10.7%)	0.00

© 2023 Lin KJ et al. *JAMA Network Open*.

Characteristics<br>Number of Patients (%)	With Dementia			Without Dementia		
	Apixaban<br>(n=19,497)	Rivaroxaban<br>(n=19,497)	St. Diff	Apixaban<br>(n=246,199)	Rivaroxaban<br>(n=246,199)	St. Diff
Statins	12,819 (65.7%)	12,826 (65.8%)	0.00	155,233 (63.1%)	155,355 (63.1%)	0.00
**Other medications**						
Anticonvulsants	4,676 (24.0%)	4,683 (24.0%)	0.00	37,412 (15.2%)	37,321 (15.2%)	0.00
Antidepressants - Other	4,168 (21.4%)	4,156 (21.3%)	0.00	17,935 (7.3%)	18,039 (7.3%)	0.00
Antidepressants - SSRI/SNRI	8,007 (41.1%)	7,982 (40.9%)	0.00	45,389 (18.4%)	45,242 (18.4%)	0.00
Antidepressants - Tricyclics	647 (3.3%)	633 (3.2%)	0.00	6,294 (2.6%)	6,325 (2.6%)	0.00
Antipsychotic agents	3,513 (18.0%)	3,512 (18.0%)	0.00	4,372 (1.8%)	4,335 (1.8%)	0.00
Anxiolytics (except benzodiazepine)	580 (3.0%)	557 (2.9%)	0.01	2,732 (1.1%)	2,755 (1.1%)	0.00
Anxiolytics - Benzodiazepines	4,877 (25.0%)	4,842 (24.8%)	0.00	42,624 (17.3%)	42,644 (17.3%)	0.00
Bronchodilators	4,403 (22.6%)	4,394 (22.5%)	0.00	47,683 (19.4%)	47,492 (19.3%)	0.00
Corticosteroids, inhaled	3,954 (20.3%)	4,031 (20.7%)	-0.01	55,187 (22.4%)	55,258 (22.4%)	0.00
Corticosteroids, oral	5,616 (28.8%)	5,625 (28.9%)	0.00	79,558 (32.3%)	79,675 (32.4%)	0.00
Dementia drugs	9,072 (46.5%)	9,088 (46.6%)	0.00	3,724 (1.5%)	3,787 (1.5%)	0.00
Diabetes agents - Insulin	2,147 (11.0%)	2,118 (10.9%)	0.00	15,920 (6.5%)	16,059 (6.5%)	0.00
Diabetes agents - Metformin	2,949 (15.1%)	2,958 (15.2%)	0.00	40,679 (16.5%)	40,660 (16.5%)	0.00
Diabetes agents - Other	1,231 (6.3%)	1,212 (6.2%)	0.00	13,033 (5.3%)	13,147 (5.3%)	0.00
Diabetes agents - Sulfonylurea	1,799 (9.2%)	1,816 (9.3%)	0.00	21,695 (8.8%)	21,769 (8.8%)	0.00
Estrogen	444 (2.3%)	433 (2.2%)	0.00	8,152 (3.3%)	8,231 (3.3%)	0.00
GI - H2 blockers	2,357 (12.1%)	2,335 (12.0%)	0.00	17,342 (7.0%)	17,385 (7.1%)	0.00
GI - Proton-pump inhibitors	7,437 (38.1%)	7,397 (37.9%)	0.00	75,278 (30.6%)	75,444 (30.6%)	0.00
GI - Sucralfate	501 (2.6%)	474 (2.4%)	0.01	4,529 (1.8%)	4,574 (1.9%)	0.00
Hypnotics	1,941 (10.0%)	1,941 (10.0%)	0.00	20,063 (8.1%)	20,141 (8.2%)	0.00
NSAIDs	2,954 (15.2%)	3,015 (15.5%)	-0.01	42,796 (17.4%)	42,877 (17.4%)	0.00
Opioids	7,932 (40.7%)	7,914 (40.6%)	0.00	91,468 (37.2%)	91,537 (37.2%)	0.00
Parkinsonism drugs	1,635 (8.4%)	1,633 (8.4%)	0.00	8,207 (3.3%)	8,218 (3.3%)	0.00
Thyroid hormone replacement	5,168 (26.5%)	5,204 (26.7%)	0.00	51,511 (20.9%)	51,528 (20.9%)	0.00
**Health care utilization**						
Emergency department visits	11,490 (58.9%)	11,524 (59.1%)	0.00	101,920 (41.4%)	102,114 (41.5%)	0.00
Home Health Day[C]	1,449 (9.7%)	1,458 (9.8%)	0.00	5,328 (3.0%)	5,285 (3.0%)	0.00
Home oxygen use	1,024 (5.3%)	1,028 (5.3%)	0.00	6,907 (2.8%)	6,956 (2.8%)	0.00
Hospitalizations	14,768 (75.7%)	14,844 (76.1%)	-0.01	107,651 (43.7%)	108,179 (43.9%)	0.00
**Geographic Region**						
Northeast	3,732 (19.1%)	3,769 (19.3%)	0.00	46,888 (19.0%)	47,138 (19.1%)	0.00
Midwest	4,157 (21.3%)	4,209 (21.6%)	-0.01	55,926 (22.7%)	55,730 (22.6%)	0.00
South	8,279 (42.5%)	8,185 (42.0%)	0.01	94,875 (38.5%)	95,106 (38.6%)	0.00
West	3,306 (17.0%)	3,313 (17.0%)	0.00	48,234 (19.6%)	47,959 (19.5%)	0.00
Other	23 (0.1%)	21 (0.1%)	0.00	276 (0.1%)	266 (0.1%)	0.00

Abbreviations: ACE, angiotensin converting enzyme; CCI, combined comorbidity; CFI, claims-based frailty index; CKD, chronic kidney disease; COPD, chronic obstructive pulmonary disease; GI, gastrointestinal; NSAID, non-steroidal anti-inflammatory drug; PVD, peripheral vascular disease; SNRI, serotonin-norepinephrine reuptake inhibitor; SSRI, selective serotonin reuptake inhibitor.[a] With both Medicare are Medicaid enrollment eligibility

[A] Other race category includes Unknown, Other, Asian, Hispanic, North American Native, and Missing Race Categories in Medicare. Asian, Hispanic, Unknown, and Missing categories are included for race in Optum. Race is unavailable in the MarketScan database.

[B] With both Medicare are Medicaid enrollment eligibility

[C] Home Health services received (days)

© 2023 Lin KJ et al. *JAMA Network Open*.

eTable 25. Selected Characteristics of Propensity Score–Matched Population With Atrial Fibrillation Treated With Rivaroxaban vs Apixaban by Dementia Diagnosis in the Medicare Population

Characteristics<br>Number of Patients (%)	With Dementia			Without Dementia		
	Apixaban (n=14,865)	Rivaroxaban (n=14,865)	St. Diff	Apixaban (n=177,656)	Rivaroxaban (n=177,656)	St. Diff
**Quarter/Year of Cohort Entry Date**						
Q1 2013	20 (0.1%)	20 (0.1%)	0.00	223 (0.1%)	223 (0.1%)	0.00
Q2 2013	126 (0.8%)	146 (1.0%)	-0.02	1,788 (1.0%)	1,973 (1.1%)	-0.01
Q3 2013	167 (1.1%)	155 (1.0%)	0.01	2,624 (1.5%)	2,706 (1.5%)	0.00
Q4 2013	306 (2.1%)	326 (2.2%)	-0.01	4,004 (2.3%)	4,173 (2.3%)	0.00
Q1 2014	511 (3.4%)	510 (3.4%)	0.00	6,381 (3.6%)	6,456 (3.6%)	0.00
Q2 2014	712 (4.8%)	709 (4.8%)	0.00	8,471 (4.8%)	8,392 (4.7%)	0.00
Q3 2014	768 (5.2%)	765 (5.1%)	0.00	8,871 (5.0%)	8,778 (4.9%)	0.00
Q4 2014	901 (6.1%)	873 (5.9%)	0.01	9,950 (5.6%)	9,856 (5.5%)	0.00
Q1 2015	984 (6.6%)	982 (6.6%)	0.00	11,413 (6.4%)	11,248 (6.3%)	0.00
Q2 2015	910 (6.1%)	913 (6.1%)	0.00	11,152 (6.3%)	11,001 (6.2%)	0.00
Q3 2015	867 (5.8%)	851 (5.7%)	0.00	9,918 (5.6%)	9,757 (5.5%)	0.00
Q4 2015	848 (5.7%)	820 (5.5%)	0.01	9,554 (5.4%)	9,547 (5.4%)	0.00
Q1 2016	943 (6.3%)	899 (6.0%)	0.01	10,857 (6.1%)	10,991 (6.2%)	0.00
Q2 2016	889 (6.0%)	928 (6.2%)	-0.01	10,365 (5.8%)	10,512 (5.9%)	0.00
Q3 2016	873 (5.9%)	881 (5.9%)	0.00	9,833 (5.5%)	9,945 (5.6%)	0.00
Q4 2016	829 (5.6%)	865 (5.8%)	-0.01	10,067 (5.7%)	10,241 (5.8%)	0.00
Q1 2017	1,083 (7.3%)	1,083 (7.3%)	0.00	13,643 (7.7%)	13,569 (7.6%)	0.00
Q2 2017	1,079 (7.3%)	1,102 (7.4%)	0.00	13,513 (7.6%)	13,406 (7.5%)	0.00
Q3 2017	1,012 (6.8%)	1,006 (6.8%)	0.00	12,470 (7.0%)	12,411 (7.0%)	0.00
Q4 2017	1,037 (7.0%)	1,031 (6.9%)	0.00	12,559 (7.1%)	12,471 (7.0%)	0.00
**Patient Characteristics**						
Mean age (SD)	82.38 (7.09)	82.39 (7.13)	0.00	76.57 (7.10)	76.62 (7.07)	-0.01
Male	5,522 (37.1%)	5,552 (37.3%)	0.00	88,298 (49.7%)	88,146 (49.6%)	0.00
Female	9,343 (62.9%)	9,313 (62.7%)	0.00	89,358 (50.3%)	89,510 (50.4%)	0.00
Black	1,015 (6.8%)	1,030 (6.9%)	0.00	6,942 (3.9%)	6,956 (3.9%)	0.00
White	12,997 (87.4%)	12,968 (87.2%)	0.01	161,690 (91.0%)	161,612 (91.0%)	0.00
Other[A]	853 (5.7%)	867 (5.8%)	0.00	9,024 (5.1%)	9,088 (5.1%)	0.00
Dual Status[B]	2,861 (19.2%)	2,849 (19.2%)	0.00	7,829 (4.4%)	7,830 (4.4%)	0.00
Mean CHA_2DS_2-VASc score (SD)	5.78 (1.62)	5.78 (1.63)	0.00	4.42 (1.66)	4.43 (1.67)	-0.01
Mean HAS-BLED score (SD)	2.66 (0.77)	2.66 (0.77)	0.00	2.20 (0.70)	2.21 (0.70)	-0.01
Mean CCI score (SD)	5.98 (2.95)	5.98 (2.96)	0.00	2.77 (2.47)	2.78 (2.49)	0.00
Mean CFI (SD)	0.31 (0.08)	0.31 (0.08)	0.00	0.19 (0.06)	0.19 (0.06)	0.00
**Medical History**						
Acute renal failure	3,586 (24.1%)	3,676 (24.7%)	-0.01	17,576 (9.9%)	17,748 (10.0%)	0.00
Acute myocardial infarction	1,246 (8.4%)	1,314 (8.8%)	-0.01	9,080 (5.1%)	9,250 (5.2%)	0.00
Alcohol abuse or dependence	465 (3.1%)	466 (3.1%)	0.00	3,276 (1.8%)	3,305 (1.9%)	-0.01
Anemia	6,635 (44.6%)	6,633 (44.6%)	0.00	42,933 (24.2%)	43,029 (24.2%)	0.00
Cardio-ablation	70 (0.5%)	81 (0.5%)	0.00	1,272 (0.7%)	1,238 (0.7%)	0.00
Cardioversion	442 (3.0%)	423 (2.8%)	0.01	12,062 (6.8%)	12,241 (6.9%)	0.00
Cerebrovascular disease	7,229 (48.6%)	7,244 (48.7%)	0.00	41,330 (23.3%)	41,420 (23.3%)	0.00
CKD: stage 3, 4 or unspecified	3,528 (23.7%)	3,534 (23.8%)	0.00	21,192 (11.9%)	21,299 (12.0%)	0.00
Congestive heart failure (inpatient)	5,183 (34.9%)	5,175 (34.8%)	0.00	29,473 (16.6%)	29,762 (16.8%)	-0.01
Congestive heart failure (outpatient)	6,579 (44.3%)	6,579 (44.3%)	0.00	48,386 (27.2%)	48,687 (27.4%)	0.00
Coronary revascularization	308 (2.1%)	311 (2.1%)	0.00	5,266 (3.0%)	5,312 (3.0%)	0.00
COPD	4,589 (30.9%)	4,550 (30.6%)	0.01	36,737 (20.7%)	36,723 (20.7%)	0.00
Dementia	14,865 (100.0%)	14,865 (100.0%)	-	0 (0.0%)	0 (0.0%)	-
Diabetes	6,330 (42.6%)	6,280 (42.2%)	0.01	61,616 (34.7%)	61,648 (34.7%)	0.00
Endoscopy	590 (4.0%)	589 (4.0%)	0.00	3,001 (1.7%)	3,014 (1.7%)	0.00
Falls	2,139 (14.4%)	2,221 (14.9%)	-0.01	5,341 (3.0%)	5,470 (3.1%)	-0.01
Fractures	2,821 (19.0%)	2,873 (19.3%)	-0.01	13,889 (7.8%)	13,933 (7.8%)	0.00
GI bleeding events (inpatient)	1,618 (10.9%)	1,637 (11.0%)	0.00	8,431 (4.7%)	8,479 (4.8%)	0.00
GI bleeding events (outpatient)	1,640 (11.0%)	1,600 (10.8%)	0.01	18,324 (10.3%)	18,229 (10.3%)	0.00
Hypertension	13,704 (92.2%)	13,683 (92.0%)	0.01	149,338 (84.1%)	149,396 (84.1%)	0.00
Ischemic Heart Disease	7,751 (52.1%)	7,815 (52.6%)	-0.01	74,767 (42.1%)	75,064 (42.3%)	0.00
Liver disease	1,047 (7.0%)	1,010 (6.8%)	0.01	10,703 (6.0%)	10,684 (6.0%)	0.00
Malignancy	2,219 (14.9%)	2,182 (14.7%)	0.01	32,909 (18.5%)	32,824 (18.5%)	0.00
Obesity	2,420 (16.3%)	2,425 (16.3%)	0.00	36,659 (20.6%)	36,416 (20.5%)	0.00
Peptic ulcer	502 (3.4%)	517 (3.5%)	-0.01	3,480 (2.0%)	3,469 (2.0%)	0.00
PVD or PVD Surgery	3,561 (24.0%)	3,562 (24.0%)	0.00	21,550 (12.1%)	21,502 (12.1%)	0.00

© 2023 Lin KJ et al. *JAMA Network Open*.

Characteristics<br>Number of Patients (%)	With Dementia			Without Dementia		
	Apixaban (n=14,865)	Rivaroxaban (n=14,865)	St. Diff	Apixaban (n=177,656)	Rivaroxaban (n=177,656)	St. Diff
Smoking	5,291 (35.6%)	5,323 (35.8%)	0.00	58,866 (33.1%)	58,945 (33.2%)	0.00
Stroke (inpatient)	2,767 (18.6%)	2,814 (18.9%)	-0.01	11,433 (6.4%)	11,629 (6.5%)	0.00
Stroke (outpatient)	2,115 (14.2%)	2,124 (14.3%)	0.00	12,784 (7.2%)	12,832 (7.2%)	0.00
Syncope	2,929 (19.7%)	2,863 (19.3%)	0.01	16,965 (9.5%)	17,053 (9.6%)	0.00
**Medications**						
ACE inhibitors	4,531 (30.5%)	4,525 (30.4%)	0.00	47,682 (26.8%)	47,752 (26.9%)	0.00
Angiotensin II receptor blockers	906 (6.1%)	897 (6.0%)	0.00	10,947 (6.2%)	10,880 (6.1%)	0.00
Antiarrhythmic agents	2,469 (16.6%)	2,437 (16.4%)	0.01	36,525 (20.6%)	36,811 (20.7%)	0.00
Anticoagulants, injectable	256 (1.7%)	231 (1.6%)	0.01	1,159 (0.7%)	1,157 (0.7%)	0.00
Anticonvulsants	3,700 (24.9%)	3,697 (24.9%)	0.00	27,707 (15.6%)	27,643 (15.6%)	0.00
Antidepressants - Other	3,223 (21.7%)	3,222 (21.7%)	0.00	13,016 (7.3%)	13,106 (7.4%)	0.00
Antidepressants - SSRI/SNRI	6,315 (42.5%)	6,273 (42.2%)	0.01	33,934 (19.1%)	33,821 (19.0%)	0.00
Antidepressants - Tricyclics	520 (3.5%)	500 (3.4%)	0.01	4,842 (2.7%)	4,870 (2.7%)	0.00
Antiplatelet agents	2,950 (19.8%)	2,970 (20.0%)	-0.01	26,135 (14.7%)	26,352 (14.8%)	0.00
Antipsychotic agents	2,857 (19.2%)	2,852 (19.2%)	0.00	3,378 (1.9%)	3,356 (1.9%)	0.00
Anxiolytics - Benzodiazepines	3,993 (26.9%)	3,955 (26.6%)	0.01	32,344 (18.2%)	32,428 (18.3%)	0.00
Anxiolytics - Other	443 (3.0%)	443 (3.0%)	0.00	1,965 (1.1%)	2,014 (1.1%)	0.00
Beta-blockers	10,010 (67.3%)	10,002 (67.3%)	0.00	118,393 (66.6%)	118,413 (66.7%)	0.00
Bronchodilators	3,488 (23.5%)	3,467 (23.3%)	0.00	34,737 (19.6%)	34,644 (19.5%)	0.00
Calcium channel blockers	372 (2.5%)	378 (2.5%)	0.00	3,802 (2.1%)	3,841 (2.2%)	-0.01
Corticosteroids, inhaled	3,184 (21.4%)	3,186 (21.4%)	0.00	41,171 (23.2%)	41,291 (23.2%)	0.00
Corticosteroids, oral	4,402 (29.6%)	4,388 (29.5%)	0.00	58,997 (33.2%)	59,269 (33.4%)	0.00
Dementia drugs	6,942 (46.7%)	6,950 (46.8%)	0.00	2,943 (1.7%)	2,960 (1.7%)	0.00
Diabetes agents - Insulin	1,720 (11.6%)	1,693 (11.4%)	0.01	11,716 (6.6%)	11,799 (6.6%)	0.00
Diabetes agents - Metformin	2,221 (14.9%)	2,230 (15.0%)	0.00	29,050 (16.4%)	29,039 (16.3%)	0.00
Diabetes agents - Other	984 (6.6%)	962 (6.5%)	0.00	9,451 (5.3%)	9,513 (5.4%)	0.00
Diabetes agents - Sulfonylurea	1,421 (9.6%)	1,422 (9.6%)	0.00	15,776 (8.9%)	15,807 (8.9%)	0.00
Diuretics	8,510 (57.2%)	8,507 (57.2%)	0.00	93,578 (52.7%)	93,850 (52.8%)	0.00
Estrogen	352 (2.4%)	346 (2.3%)	0.01	6,299 (3.5%)	6,387 (3.6%)	-0.01
Fibrates	545 (3.7%)	532 (3.6%)	0.01	8,052 (4.5%)	8,015 (4.5%)	0.00
GI - H2 blockers	1,899 (12.8%)	1,894 (12.7%)	0.00	13,116 (7.4%)	13,094 (7.4%)	0.00
GI - Proton-pump inhibitors	5,950 (40.0%)	5,937 (39.9%)	0.00	56,438 (31.8%)	56,403 (31.7%)	0.00
GI - Sucralfate	411 (2.8%)	396 (2.7%)	0.01	3,485 (2.0%)	3,537 (2.0%)	0.00
Hypnotics	1,584 (10.7%)	1,597 (10.7%)	0.00	15,503 (8.7%)	15,607 (8.8%)	0.00
Nitrates	2,119 (14.3%)	2,122 (14.3%)	0.00	19,870 (11.2%)	20,179 (11.4%)	-0.01
NSAIDs	2,345 (15.8%)	2,396 (16.1%)	-0.01	31,755 (17.9%)	31,720 (17.9%)	0.00
Opioids	6,314 (42.5%)	6,292 (42.3%)	0.00	68,212 (38.4%)	68,233 (38.4%)	0.00
Parkinsonism drugs	1,319 (8.9%)	1,312 (8.8%)	0.00	6,160 (3.5%)	6,228 (3.5%)	0.00
Statins	9,917 (66.7%)	9,947 (66.9%)	0.00	113,346 (63.8%)	113,366 (63.8%)	0.00
Thyroid hormone replacement	4,103 (27.6%)	4,080 (27.4%)	0.00	38,196 (21.5%)	38,218 (21.5%)	0.00
**Health care use**						
Emergency department visits	8,108 (54.5%)	8,119 (54.6%)	0.00	68,939 (38.8%)	69,030 (38.9%)	0.00
Home health day[C]	1,449 (9.7%)	1,458 (9.8%)	0.00	5,328 (3.0%)	5,285 (3.0%)	0.00
Home oxygen use	765 (5.1%)	781 (5.3%)	-0.01	5,217 (2.9%)	5,234 (2.9%)	0.00
Hospitalizations	11,679 (78.6%)	11,745 (79.0%)	-0.01	82,229 (46.3%)	82,597 (46.5%)	0.00
**Geographic Region**						
Northeast	2,957 (19.9%)	3,002 (20.2%)	-0.01	35,070 (19.7%)	35,147 (19.8%)	0.00
Midwest	3,119 (21.0%)	3,153 (21.2%)	0.00	39,949 (22.5%)	39,790 (22.4%)	0.00
South	6,458 (43.4%)	6,381 (42.9%)	0.01	69,841 (39.3%)	70,184 (39.5%)	0.00
West	2,313 (15.6%)	2,311 (15.5%)	0.00	32,630 (18.4%)	32,372 (18.2%)	0.01
Other	18 (0.1%)	18 (0.1%)	0.00	166 (0.1%)	163 (0.1%)	0.00

Abbreviations: ACE, angiotensin converting enzyme; CCI, combined comorbidity; CFI, claims-based frailty index; CKD, chronic kidney disease; COPD, chronic obstructive pulmonary disease; GI, gastrointestinal; NSAID, non-steroidal anti-inflammatory drug; PVD, peripheral vascular disease; SNRI, serotonin-norepinephrine reuptake inhibitor; SSRI, selective serotonin reuptake inhibitor.[a] With both Medicare are Medicaid enrollment eligibility

[A] Other race category includes Unknown, Other, Asian, Hispanic, North American Native, and Missing Race Categories in Medicare. Asian, Hispanic, Unknown, and Missing categories are included for race in Optum. Race is unavailable in the MarketScan database.

[B] With both Medicare are Medicaid enrollment eligibility

[C] Home Health services received (days)

© 2023 Lin KJ et al. *JAMA Network Open*.

eTable 26. Selected Characteristics of Propensity Score–Matched Population With Atrial Fibrillation Treated With Rivaroxaban vs Apixaban by Dementia Diagnosis in the Optum Population

Characteristics<br>Number of Patients (%)	With Dementia			Without Dementia		
	Apixaban (n=3,283)	Rivaroxaban (n=3,283)	St. Diff	Apixaban (n=44,262)	Rivaroxaban (n=44,262)	St. Diff
**Quarter/Year of Cohort Entry Date**						
Q1 2013	0 (0.0%)	0 (0.0%)	-	36 (0.1%)	37 (0.1%)	0.00
Q2 2013	10 (0.3%)	8 (0.2%)	0.02	182 (0.4%)	221 (0.5%)	-0.01
Q3 2013	12 (0.4%)	14 (0.4%)	0.00	235 (0.5%)	262 (0.6%)	-0.01
Q4 2013	17 (0.5%)	15 (0.5%)	0.00	416 (0.9%)	444 (1.0%)	-0.01
Q1 2014	35 (1.1%)	36 (1.1%)	0.00	635 (1.4%)	679 (1.5%)	-0.01
Q2 2014	51 (1.6%)	68 (2.1%)	-0.04	755 (1.7%)	767 (1.7%)	0.00
Q3 2014	45 (1.4%)	46 (1.4%)	0.00	824 (1.9%)	818 (1.8%)	0.01
Q4 2014	61 (1.9%)	66 (2.0%)	-0.01	893 (2.0%)	865 (2.0%)	0.00
Q1 2015	78 (2.4%)	71 (2.2%)	0.01	1,083 (2.4%)	1,071 (2.4%)	0.00
Q2 2015	70 (2.1%)	74 (2.3%)	-0.01	1,044 (2.4%)	1,015 (2.3%)	0.01
Q3 2015	77 (2.3%)	79 (2.4%)	-0.01	939 (2.1%)	955 (2.2%)	-0.01
Q4 2015	62 (1.9%)	61 (1.9%)	0.00	920 (2.1%)	884 (2.0%)	0.01
Q1 2016	88 (2.7%)	94 (2.9%)	-0.01	1,323 (3.0%)	1,318 (3.0%)	0.00
Q2 2016	72 (2.2%)	80 (2.4%)	-0.01	1,127 (2.5%)	1,094 (2.5%)	0.00
Q3 2016	82 (2.5%)	74 (2.3%)	0.01	1,121 (2.5%)	1,109 (2.5%)	0.00
Q4 2016	62 (1.9%)	69 (2.1%)	-0.01	1,010 (2.3%)	1,014 (2.3%)	0.00
Q1 2017	154 (4.7%)	140 (4.3%)	0.02	1,829 (4.1%)	1,844 (4.2%)	-0.01
Q2 2017	101 (3.1%)	111 (3.4%)	-0.02	1,578 (3.6%)	1,590 (3.6%)	0.00
Q3 2017	119 (3.6%)	115 (3.5%)	0.01	1,605 (3.6%)	1,553 (3.5%)	0.01
Q4 2017	85 (2.6%)	92 (2.8%)	-0.01	1,585 (3.6%)	1,535 (3.5%)	0.01
Q1 2018	191 (5.8%)	185 (5.6%)	0.01	2,552 (5.8%)	2,428 (5.5%)	0.01
Q2 2018	217 (6.6%)	185 (5.6%)	0.04	2,010 (4.5%)	1,970 (4.5%)	0.00
Q3 2018	153 (4.7%)	144 (4.4%)	0.01	1,871 (4.2%)	1,823 (4.1%)	0.01
Q4 2018	143 (4.4%)	124 (3.8%)	0.03	1,648 (3.7%)	1,661 (3.8%)	-0.01
Q1 2019	134 (4.1%)	138 (4.2%)	-0.01	1,935 (4.4%)	1,959 (4.4%)	0.00
Q2 2019	112 (3.4%)	115 (3.5%)	-0.01	1,784 (4.0%)	1,780 (4.0%)	0.00
Q3 2019	141 (4.3%)	133 (4.1%)	0.01	1,603 (3.6%)	1,601 (3.6%)	0.00
Q4 2019	77 (2.3%)	93 (2.8%)	-0.03	1,472 (3.3%)	1,494 (3.4%)	-0.01
Q1 2020	110 (3.4%)	114 (3.5%)	-0.01	1,941 (4.4%)	1,958 (4.4%)	0.00
Q2 2020	113 (3.4%)	110 (3.4%)	0.00	1,364 (3.1%)	1,380 (3.1%)	0.00
Q3 2020	91 (2.8%)	100 (3.0%)	-0.01	1,274 (2.9%)	1,351 (3.1%)	-0.01
Q4 2020	102 (3.1%)	103 (3.1%)	0.00	1,275 (2.9%)	1,333 (3.0%)	-0.01
Q1 2021	288 (8.8%)	281 (8.6%)	0.01	2,770 (6.3%)	2,776 (6.3%)	0.00
Q2 2021	130 (4.0%)	145 (4.4%)	-0.02	1,623 (3.7%)	1,673 (3.8%)	-0.01
**Patient Characteristics**						
Mean age (SD)	81.53 (6.01)	81.50 (5.86)	0.01	75.77 (6.62)	75.77 (6.56)	0.00
Male	1,499 (45.7%)	1,467 (44.7%)	0.02	24,230 (54.7%)	24,115 (54.5%)	0.00
Female	1,784 (54.3%)	1,816 (55.3%)	-0.02	20,032 (45.3%)	20,147 (45.5%)	0.00
Black	321 (9.8%)	328 (10.0%)	-0.01	3,540 (8.0%)	3,531 (8.0%)	0.00
White	2,356 (71.8%)	2,358 (71.8%)	0.00	33,257 (75.1%)	33,269 (75.2%)	0.00
Other[A]	606 (18.5%)	597 (18.2%)	0.01	7,465 (16.9%)	7,462 (16.9%)	0.00
Mean CHA_2DS_2-VASc score (SD)	5.58 (1.61)	5.59 (1.61)	-0.01	4.38 (1.63)	4.39 (1.63)	-0.01
Mean HAS-BLED score (SD)	2.62 (0.80)	2.63 (0.79)	-0.01	2.22 (0.68)	2.23 (0.68)	-0.01
Mean CCI score (SD)	7.01 (3.09)	6.96 (3.07)	0.02	3.28 (2.72)	3.30 (2.71)	-0.01
Mean CFI (SD)	0.30 (0.07)	0.30 (0.07)	0.00	0.18 (0.05)	0.18 (0.05)	0.00
**Medical History**						
Acute myocardial infarction	265 (8.1%)	265 (8.1%)	0.00	2,242 (5.1%)	2,266 (5.1%)	0.00
Acute renal failure	764 (23.3%)	760 (23.1%)	0.00	4,108 (9.3%)	4,268 (9.6%)	-0.01
Alcohol abuse or dependence	155 (4.7%)	149 (4.5%)	0.01	1,268 (2.9%)	1,258 (2.8%)	0.01
Anemia	1,289 (39.3%)	1,304 (39.7%)	-0.01	9,509 (21.5%)	9,526 (21.5%)	0.00
Cardio-ablation	9 (0.3%)	9 (0.3%)	0.00	223 (0.5%)	217 (0.5%)	0.00
Cardioversion	126 (3.8%)	116 (3.5%)	0.02	3,134 (7.1%)	3,079 (7.0%)	0.00
Cerebrovascular disease	1,335 (40.7%)	1,355 (41.3%)	-0.01	8,745 (19.8%)	8,775 (19.8%)	0.00
CKD: stage 3, 4 or unspecified	1,052 (32.0%)	1,036 (31.6%)	0.01	8,502 (19.2%)	8,548 (19.3%)	0.00

© 2023 Lin KJ et al. *JAMA Network Open*.

Characteristics<br>Number of Patients (%)	With Dementia			Without Dementia		
	Apixaban<br>(n=3,283)	Rivaroxaban<br>(n=3,283)	St. Diff	Apixaban<br>(n=44,262)	Rivaroxaban<br>(n=44,262)	St. Diff
Congestive heart failure (inpatient)	729 (22.2%)	726 (22.1%)	0.00	4,945 (11.2%)	5,026 (11.4%)	-0.01
Congestive heart failure (outpatient)	1,588 (48.4%)	1,552 (47.3%)	0.02	14,157 (32.0%)	14,182 (32.0%)	0.00
Coronary revascularization	58 (1.8%)	57 (1.7%)	0.01	1,073 (2.4%)	1,088 (2.5%)	-0.01
COPD	980 (29.9%)	954 (29.1%)	0.02	9,485 (21.4%)	9,488 (21.4%)	0.00
Dementia	3,283 (100.0%)	3,283 (100.0%)	-	0 (0.0%)	0 (0.0%)	-
Diabetes	1,431 (43.6%)	1,410 (42.9%)	0.01	15,542 (35.1%)	15,549 (35.1%)	0.00
Endoscopy	89 (2.7%)	79 (2.4%)	0.02	461 (1.0%)	476 (1.1%)	-0.01
Falls	1,093 (33.3%)	1,093 (33.3%)	0.00	4,226 (9.5%)	4,210 (9.5%)	0.00
Fractures	616 (18.8%)	608 (18.5%)	0.01	3,142 (7.1%)	3,121 (7.1%)	0.00
GI bleeding events (inpatient)	164 (5.0%)	154 (4.7%)	0.01	796 (1.8%)	803 (1.8%)	0.00
GI bleeding events (outpatient)	901 (27.4%)	881 (26.8%)	0.01	9,333 (21.1%)	9,420 (21.3%)	0.00
Hypertension	3,016 (91.9%)	3,024 (92.1%)	-0.01	38,545 (87.1%)	38,562 (87.1%)	0.00
Ischemic Heart Disease	1,677 (51.1%)	1,639 (49.9%)	0.02	17,828 (40.3%)	17,942 (40.5%)	0.00
Liver disease	253 (7.7%)	249 (7.6%)	0.00	3,079 (7.0%)	3,088 (7.0%)	0.00
Malignancy	479 (14.6%)	473 (14.4%)	0.01	6,789 (15.3%)	6,819 (15.4%)	0.00
Obesity	495 (15.1%)	515 (15.7%)	-0.02	10,142 (22.9%)	10,295 (23.3%)	-0.01
Peptic ulcer	122 (3.7%)	106 (3.2%)	0.03	838 (1.9%)	855 (1.9%)	0.00
PVD or PVD Surgery	834 (25.4%)	845 (25.7%)	-0.01	6,327 (14.3%)	6,319 (14.3%)	0.00
Smoking	1,126 (34.3%)	1,089 (33.2%)	0.02	12,723 (28.7%)	12,655 (28.6%)	0.00
Stroke (inpatient)	394 (12.0%)	395 (12.0%)	0.00	1,738 (3.9%)	1,800 (4.1%)	-0.01
Stroke (outpatient)	1,237 (37.7%)	1,257 (38.3%)	-0.01	8,321 (18.8%)	8,341 (18.8%)	0.00
Syncope	621 (18.9%)	641 (19.5%)	-0.02	4,259 (9.6%)	4,205 (9.5%)	0.00
**Medications**						
ACE inhibitors	864 (26.3%)	877 (26.7%)	-0.01	11,573 (26.1%)	11,507 (26.0%)	0.00
Angiotensin II receptor blockers	129 (3.9%)	141 (4.3%)	-0.02	2,276 (5.1%)	2,324 (5.3%)	-0.01
Antiarrhythmic agents	404 (12.3%)	422 (12.9%)	-0.02	7,684 (17.4%)	7,649 (17.3%)	0.00
Anticoagulants, injectable	33 (1.0%)	36 (1.1%)	-0.01	199 (0.4%)	198 (0.4%)	0.00
Anticonvulsants	715 (21.8%)	717 (21.8%)	0.00	6,599 (14.9%)	6,571 (14.8%)	0.00
Antidepressants - Other	682 (20.8%)	667 (20.3%)	0.01	3,393 (7.7%)	3,426 (7.7%)	0.00
Antidepressants - SSRI/SNRI	1,172 (35.7%)	1,173 (35.7%)	0.00	7,459 (16.9%)	7,488 (16.9%)	0.00
Antidepressants - Tricyclics	89 (2.7%)	89 (2.7%)	0.00	896 (2.0%)	888 (2.0%)	0.00
Antiplatelet agents	503 (15.3%)	502 (15.3%)	0.00	5,484 (12.4%)	5,496 (12.4%)	0.00
Antipsychotic agents	464 (14.1%)	458 (14.0%)	0.00	660 (1.5%)	635 (1.4%)	0.01
Anxiolytics - Benzodiazepines	606 (18.5%)	597 (18.2%)	0.01	6,428 (14.5%)	6,373 (14.4%)	0.00
Anxiolytics - Other	98 (3.0%)	83 (2.5%)	0.03	545 (1.2%)	529 (1.2%)	0.00
Beta-blockers	2,096 (63.8%)	2,087 (63.6%)	0.00	28,517 (64.4%)	28,517 (64.4%)	0.00
Bronchodilators	690 (21.0%)	676 (20.6%)	0.01	8,307 (18.8%)	8,315 (18.8%)	0.00
Calcium channel blockers	0 (0.0%)	1 (0.0%)	-	28 (0.1%)	28 (0.1%)	0.00
Corticosteroids, inhaled	557 (17.0%)	600 (18.3%)	-0.03	8,608 (19.4%)	8,566 (19.4%)	0.00
Corticosteroids, oral	860 (26.2%)	865 (26.3%)	0.00	12,951 (29.3%)	12,872 (29.1%)	0.00
Dementia drugs	1,445 (44.0%)	1,465 (44.6%)	-0.01	436 (1.0%)	469 (1.1%)	-0.01
Diabetes agents - Insulin	318 (9.7%)	303 (9.2%)	0.02	2,651 (6.0%)	2,667 (6.0%)	0.00
Diabetes agents - Metformin	560 (17.1%)	545 (16.6%)	0.01	7,804 (17.6%)	7,779 (17.6%)	0.00
Diabetes agents - Other	171 (5.2%)	168 (5.1%)	0.00	2,149 (4.9%)	2,206 (5.0%)	0.00
Diabetes agents - Sulfonylurea	272 (8.3%)	278 (8.5%)	-0.01	3,908 (8.8%)	3,910 (8.8%)	0.00
Diuretics	1,651 (50.3%)	1,647 (50.2%)	0.00	21,538 (48.7%)	21,618 (48.8%)	0.00
Estrogen	53 (1.6%)	52 (1.6%)	0.00	978 (2.2%)	966 (2.2%)	0.00
Fibrates	102 (3.1%)	94 (2.9%)	0.01	1,740 (3.9%)	1,722 (3.9%)	0.00
GI - H2 blockers	338 (10.3%)	324 (9.9%)	0.01	2,796 (6.3%)	2,835 (6.4%)	0.00
GI - Proton-pump inhibitors	1,044 (31.8%)	1,025 (31.2%)	0.01	11,949 (27.0%)	12,128 (27.4%)	-0.01
GI - Sucralfate	60 (1.8%)	54 (1.6%)	0.02	695 (1.6%)	674 (1.5%)	0.01
Hypnotics	241 (7.3%)	224 (6.8%)	0.02	2,724 (6.2%)	2,698 (6.1%)	0.00
Nitrates	325 (9.9%)	325 (9.9%)	0.00	3,821 (8.6%)	3,771 (8.5%)	0.00
NSAIDs	423 (12.9%)	431 (13.1%)	-0.01	7,001 (15.8%)	7,055 (15.9%)	0.00
Opioids	1,096 (33.4%)	1,108 (33.7%)	-0.01	14,140 (31.9%)	14,238 (32.2%)	-0.01
Parkinsonism drugs	218 (6.6%)	210 (6.4%)	0.01	1,326 (3.0%)	1,250 (2.8%)	0.01
Statins	2,074 (63.2%)	2,054 (62.6%)	0.01	26,841 (60.6%)	26,938 (60.9%)	-0.01
Thyroid hormone replacement	764 (23.3%)	787 (24.0%)	-0.02	8,509 (19.2%)	8,509 (19.2%)	0.00
**Health care use**						
Emergency department visits	2,368 (72.1%)	2,372 (72.3%)	0.00	20,808 (47.0%)	20,900 (47.2%)	0.00

Characteristics<br>Number of Patients (%)	With Dementia			Without Dementia		
	Apixaban<br>(n=3,283)	Rivaroxaban<br>(n=3,283)	St. Diff	Apixaban<br>(n=44,262)	Rivaroxaban<br>(n=44,262)	St. Diff
Home oxygen use	212 (6.5%)	200 (6.1%)	0.02	1,395 (3.2%)	1,434 (3.2%)	0.00
Hospitalizations	2,130 (64.9%)	2,134 (65.0%)	0.00	15,329 (34.6%)	15,379 (34.7%)	0.00
**Geographic Region**						
Northeast	506 (15.4%)	491 (15.0%)	0.01	5,652 (12.8%)	5,770 (13.0%)	-0.01
Midwest	539 (16.4%)	559 (17.0%)	-0.02	8,357 (18.9%)	8,260 (18.7%)	0.01
South	1,384 (42.2%)	1,379 (42.0%)	0.00	17,456 (39.4%)	17,478 (39.5%)	0.00
West	852 (26.0%)	853 (26.0%)	0.00	12,762 (28.8%)	12,719 (28.7%)	0.00
Other	2 (0.1%)	1 (0.0%)	0.04	35 (0.1%)	35 (0.1%)	0.00
**Business Type Code (Recategorized)**						
Commercial	130 (4.0%)	122 (3.7%)	0.02	3,859 (8.7%)	3,912 (8.8%)	0.00
Medicare	3,153 (96.0%)	3,161 (96.3%)	-0.02	40,403 (91.3%)	40,350 (91.2%)	0.00

Abbreviations: ACE, angiotensin converting enzyme; CCI, combined comorbidity; CFI, claims-based frailty index; CKD, chronic kidney disease; COPD, chronic obstructive pulmonary disease; GI, gastrointestinal; NSAID, non-steroidal anti-inflammatory drug; PVD, peripheral vascular disease; SNRI, serotonin-norepinephrine reuptake inhibitor; SSRI, selective serotonin reuptake inhibitor

[A] Other race category includes Unknown, Other, Asian, Hispanic, North American Native, and Missing Race Categories in Medicare. Asian, Hispanic, Unknown, and Missing categories are included for race in Optum. Race is unavailable in the MarketScan database.

© 2023 Lin KJ et al. *JAMA Network Open*.

eTable 27. Selected Characteristics of Propensity Score–Matched Population With Atrial Fibrillation Treated With Rivaroxaban vs Apixaban by Dementia Diagnosis in the MarketScan Population

Characteristics<br>Number of Patients (%)	With Dementia			Without Dementia		
	Apixaban (n=1,360)	Warfarin (n=1,360)	St. Diff	Apixaban (n=24,396)	Warfarin (n=24,396)	St. Diff
**Quarter/Year of Cohort Entry Date**						
2013	125 (9.2%)	131 (9.6%)	0.00	2,359 (9.7%)	2,429 (10.0%)	0.00
2014	266 (19.6%)	259 (19.0%)	0.01	5,258 (21.6%)	5,126 (21.0%)	0.01
2015	290 (21.3%)	296 (21.8%)	0.00	4,795 (19.7%)	4,731 (19.4%)	0.00
2016	262 (19.3%)	242 (17.8%)	0.02	4,440 (18.2%)	4,439 (18.2%)	0.00
2017	191 (14.0%)	188 (13.8%)	0.00	3,046 (12.5%)	3,058 (12.5%)	0.00
2018	87 (6.4%)	97 (7.1%)	-0.01	1,775 (7.3%)	1,836 (7.5%)	0.00
2019	88 (6.5%)	94 (6.9%)	0.00	1,728 (7.1%)	1,746 (7.2%)	0.00
2020	51 (3.8%)	53 (3.9%)	0.00	995 (4.1%)	1,031 (4.2%)	0.00
**Patient Characteristics**						
Mean age (SD)	82.71 (6.88)	82.76 (6.66)	-0.05	76.31 (7.57)	76.26 (7.35)	0.05
Male	627 (46.1%)	624 (45.9%)	0.00	13,564 (55.6%)	13,623 (55.8%)	0.00
Female	733 (53.9%)	736 (54.1%)	0.00	10,832 (44.4%)	10,773 (44.2%)	0.00
Mean CHA2DS2-VASc score (SD)	4.51 (1.51)	4.51 (1.56)	0.00	3.50 (1.56)	3.49 (1.57)	0.01
Mean HAS-BLED score (SD)	2.51 (0.77)	2.50 (0.77)	0.01	2.13 (0.65)	2.13 (0.65)	0.00
Mean CCI score (SD)	5.71 (3.06)	5.71 (2.97)	0.00	2.61 (2.36)	2.62 (2.38)	-0.01
Mean CFI (SD)	0.29 (0.07)	0.29 (0.07)	0.00	0.18 (0.05)	0.18 (0.05)	0.00
**Medical History**						
Acute myocardial infarction	132 (9.7%)	129 (9.5%)	0.00	1,328 (5.4%)	1,320 (5.4%)	0.00
Acute renal failure	258 (19.0%)	257 (18.9%)	0.00	1,907 (7.8%)	1,974 (8.1%)	0.00
Alcohol abuse or dependence	38 (2.8%)	32 (2.4%)	0.00	422 (1.7%)	432 (1.8%)	0.00
Anemia	449 (33.0%)	429 (31.5%)	0.02	4,238 (17.4%)	4,254 (17.4%)	0.00
Cardio-ablation	7 (0.5%)	5 (0.4%)	0.00	148 (0.6%)	148 (0.6%)	0.00
Cardioversion	59 (4.3%)	58 (4.3%)	0.00	1,930 (7.9%)	1,900 (7.8%)	0.00
Cerebrovascular disease	621 (45.7%)	630 (46.3%)	-0.01	5,177 (21.2%)	5,249 (21.5%)	0.00
CKD: stage 3, 4 or unspecified	276 (20.3%)	271 (19.9%)	0.00	2,840 (11.6%)	2,796 (11.5%)	0.00
Congestive heart failure (inpatient)	394 (29.0%)	391 (28.7%)	0.00	3,887 (15.9%)	3,853 (15.8%)	0.00
Congestive heart failure (outpatient)	529 (38.9%)	508 (37.4%)	0.02	6,017 (24.7%)	5,983 (24.5%)	0.00
Coronary revascularization	26 (1.9%)	30 (2.2%)	0.00	675 (2.8%)	659 (2.7%)	0.00
COPD	372 (27.4%)	355 (26.1%)	0.01	4,599 (18.9%)	4,520 (18.5%)	0.00
Dementia	1,360 (100.0%)	1,360 (100.0%)	0.00	0 (0.0%)	0 (0.0%)	0.00
Diabetes	459 (33.8%)	471 (34.6%)	-0.01	7,527 (30.9%)	7,590 (31.1%)	0.00
Endoscopy	25 (1.8%)	27 (2.0%)	0.00	247 (1.0%)	244 (1.0%)	0.00
Falls	296 (21.8%)	305 (22.4%)	-0.01	1,376 (5.6%)	1,395 (5.7%)	0.00
Fractures	247 (18.2%)	247 (18.2%)	0.00	1,797 (7.4%)	1,777 (7.3%)	0.00
GI bleeding events (inpatient)	86 (6.3%)	85 (6.3%)	0.00	733 (3.0%)	715 (2.9%)	0.00
GI bleeding events (outpatient)	196 (14.4%)	187 (13.8%)	0.01	3,289 (13.5%)	3,285 (13.5%)	0.00
Hypertension	1,223 (89.9%)	1,222 (89.9%)	0.00	20,583 (84.4%)	20,583 (84.4%)	0.00
Ischemic Heart Disease	684 (50.3%)	681 (50.1%)	0.00	10,192 (41.8%)	10,245 (42.0%)	0.00
Liver disease	69 (5.1%)	77 (5.7%)	-0.01	1,299 (5.3%)	1,313 (5.4%)	0.00
Malignancy	222 (16.3%)	227 (16.7%)	0.00	4,553 (18.7%)	4,561 (18.7%)	0.00
Obesity	123 (9.0%)	136 (10.0%)	-0.01	3,442 (14.1%)	3,508 (14.4%)	0.00
Peptic ulcer	23 (1.7%)	25 (1.8%)	0.00	283 (1.2%)	287 (1.2%)	0.00
PVD or PVD Surgery	311 (22.9%)	314 (23.1%)	0.00	2,746 (11.3%)	2,739 (11.2%)	0.00
Smoking	171 (12.6%)	186 (13.7%)	-0.01	3,124 (12.8%)	3,156 (12.9%)	0.00
Stroke (inpatient)	276 (20.3%)	289 (21.3%)	-0.01	1,711 (7.0%)	1,750 (7.2%)	0.00
Stroke (outpatient)	506 (37.2%)	518 (38.1%)	-0.01	4,329 (17.7%)	4,389 (18.0%)	0.00
Syncope	287 (21.1%)	280 (20.6%)	0.01	2,460 (10.1%)	2,438 (10.0%)	0.00
**Medications**						
ACE inhibitors	360 (26.5%)	371 (27.3%)	-0.01	6,120 (25.1%)	6,116 (25.1%)	0.00
Angiotensin II receptor blockers	70 (5.1%)	78 (5.7%)	-0.01	1,566 (6.4%)	1,562 (6.4%)	0.00
Antiarrhythmic agents	200 (14.7%)	206 (15.1%)	0.00	4,563 (18.7%)	4,523 (18.5%)	0.00
Anticoagulants, injectable	14 (1.0%)	12 (0.9%)	0.00	140 (0.6%)	145 (0.6%)	0.00
Anticonvulsants	269 (19.8%)	273 (20.1%)	0.00	3,152 (12.9%)	3,140 (12.9%)	0.00
Antidepressants - Other	264 (19.4%)	259 (19.0%)	0.00	1,497 (6.1%)	1,494 (6.1%)	0.00

© 2023 Lin KJ et al. *JAMA Network Open*.

Characteristics<br>Number of Patients (%)	With Dementia			Without Dementia		
	Apixaban (n=1,360)	Warfarin (n=1,360)	St. Diff	Apixaban (n=24,396)	Warfarin (n=24,396)	St. Diff
Antidepressants - SSRI/SNRI	537 (39.5%)	549 (40.4%)	-0.01	4,007 (16.4%)	3,955 (16.2%)	0.00
Antidepressants - Tricyclics	38 (2.8%)	40 (2.9%)	0.00	595 (2.4%)	562 (2.3%)	0.00
Antiplatelet agents	289 (21.3%)	288 (21.2%)	0.00	3,907 (16.0%)	3,788 (15.5%)	0.01
Antipsychotic agents	203 (14.9%)	206 (15.1%)	0.00	377 (1.5%)	340 (1.4%)	0.00
Anxiolytics - Benzodiazepines	290 (21.3%)	281 (20.7%)	0.01	3,892 (16.0%)	3,846 (15.8%)	0.00
Anxiolytics - Other	36 (2.6%)	36 (2.6%)	0.00	229 (0.9%)	210 (0.9%)	0.00
Beta-blockers	889 (65.4%)	891 (65.5%)	0.00	15,917 (65.2%)	15,905 (65.2%)	0.00
Bronchodilators	245 (18.0%)	241 (17.7%)	0.00	4,637 (19.0%)	4,535 (18.6%)	0.00
Calcium channel blockers	3 (0.2%)	2 (0.1%)	0.00	56 (0.2%)	48 (0.2%)	0.00
Corticosteroids, inhaled	243 (17.9%)	238 (17.5%)	0.00	5,522 (22.6%)	5,453 (22.4%)	0.00
Corticosteroids, oral	382 (28.1%)	357 (26.3%)	0.02	7,597 (31.1%)	7,584 (31.1%)	0.00
Dementia drugs	689 (50.7%)	691 (50.8%)	0.00	320 (1.3%)	351 (1.4%)	0.00
Diabetes agents - Insulin	123 (9.0%)	124 (9.1%)	0.00	1,564 (6.4%)	1,587 (6.5%)	0.00
Diabetes agents - Metformin	177 (13.0%)	178 (13.1%)	0.00	3,843 (15.8%)	3,854 (15.8%)	0.00
Diabetes agents - Other	78 (5.7%)	80 (5.9%)	0.00	1,450 (5.9%)	1,418 (5.8%)	0.00
Diabetes agents - Sulfonylurea	112 (8.2%)	110 (8.1%)	0.00	2,005 (8.2%)	2,065 (8.5%)	0.00
Diuretics	697 (51.2%)	680 (50.0%)	0.01	12,045 (49.4%)	11,985 (49.1%)	0.00
Estrogen	44 (3.2%)	40 (2.9%)	0.00	865 (3.5%)	886 (3.6%)	0.00
Fibrates	47 (3.5%)	54 (4.0%)	-0.01	1,123 (4.6%)	1,137 (4.7%)	0.00
GI - H2 blockers	110 (8.1%)	113 (8.3%)	0.00	1,459 (6.0%)	1,460 (6.0%)	0.00
GI - Proton-pump inhibitors	447 (32.9%)	439 (32.3%)	0.01	6,973 (28.6%)	6,978 (28.6%)	0.00
GI - Sucralfate	22 (1.6%)	23 (1.7%)	0.00	355 (1.5%)	353 (1.4%)	0.00
Hypnotics	115 (8.5%)	129 (9.5%)	-0.01	1,839 (7.5%)	1,873 (7.7%)	0.00
Nitrates	167 (12.3%)	179 (13.2%)	-0.01	2,469 (10.1%)	2,474 (10.1%)	0.00
NSAIDs	188 (13.8%)	190 (14.0%)	0.00	4,079 (16.7%)	4,096 (16.8%)	0.00
Opioids	523 (38.5%)	517 (38.0%)	0.00	9,118 (37.4%)	9,076 (37.2%)	0.00
Parkinsonism drugs	105 (7.7%)	109 (8.0%)	0.00	708 (2.9%)	742 (3.0%)	0.00
Statins	811 (59.6%)	829 (61.0%)	-0.01	15,055 (61.7%)	15,118 (62.0%)	0.00
Thyroid hormone replacement	348 (25.6%)	337 (24.8%)	0.01	4,867 (19.9%)	4,809 (19.7%)	0.00
**Health care use**						
Emergency department visits	1,030 (75.7%)	1,033 (76.0%)	0.00	12,206 (50.0%)	12,250 (50.2%)	0.00
Hospitalizations	962 (70.7%)	970 (71.3%)	-0.01	10,221 (41.9%)	10,206 (41.8%)	0.00
**Geographic Region**						
Northeast	287 (21.1%)	283 (20.8%)	0.00	6,285 (25.8%)	6,225 (25.5%)	0.00
Midwest	493 (36.3%)	489 (36.0%)	0.00	7,550 (30.9%)	7,765 (31.8%)	-0.01
South	436 (32.1%)	444 (32.6%)	-0.01	7,655 (31.4%)	7,487 (30.7%)	0.01
West	141 (10.4%)	141 (10.4%)	0.00	2,837 (11.6%)	2,849 (11.7%)	0.00
Other	3 (0.2%)	3 (0.2%)	0.00	69 (0.3%)	70 (0.3%)	0.00
**Employee Classification (Recategorized)**						
Salary Non-union	120 (8.8%)	121 (8.9%)	0.00	2,285 (9.4%)	2,313 (9.5%)	0.00
Salary Union	25 (1.8%)	28 (2.1%)	0.00	526 (2.2%)	543 (2.2%)	0.00
Salary Other	19 (1.4%)	20 (1.5%)	0.00	492 (2.0%)	497 (2.0%)	0.00
Hourly Non-Union	64 (4.7%)	57 (4.2%)	0.01	1,288 (5.3%)	1,265 (5.2%)	0.00
Hourly Union	436 (32.1%)	436 (32.1%)	0.00	6,603 (27.1%)	6,560 (26.9%)	0.00
Hourly Other	5 (0.4%)	5 (0.4%)	0.00	169 (0.7%)	170 (0.7%)	0.00
Non-Union	295 (21.7%)	276 (20.3%)	0.01	5,611 (23.0%)	5,526 (22.7%)	0.00
Union	15 (1.1%)	11 (0.8%)	0.00	332 (1.4%)	371 (1.5%)	0.00
Unknown	381 (28.0%)	406 (29.9%)	-0.02	7,090 (29.1%)	7,151 (29.3%)	0.00
**Employment Status (Recategorized)**						
Active Full Time	18 (1.3%)	19 (1.4%)	0.00	1,769 (7.3%)	1,778 (7.3%)	0.00
Active Part Time or Seasonal	0 (0.0%)	0 (0.0%)	0.00	70 (0.3%)	65 (0.3%)	0.00
Early Retiree	5 (0.4%)	4 (0.3%)	0.00	329 (1.3%)	319 (1.3%)	0.00
Medicare Eligible Retiree	957 (70.4%)	952 (70.0%)	0.00	17,141 (70.3%)	17,205 (70.5%)	0.00
Retiree	23 (1.7%)	23 (1.7%)	0.00	333 (1.4%)	343 (1.4%)	0.00
COBRA Continuee	0 (0.0%)	0 (0.0%)	0.00	11 (0.0%)	15 (0.1%)	0.00
Long Term Disability	0 (0.0%)	0 (0.0%)	0.00	24 (0.1%)	28 (0.1%)	0.00
Surviving Spouse/Dependent	201 (14.8%)	199 (14.6%)	0.00	1,926 (7.9%)	1,929 (7.9%)	0.00
Other/Unknown/Missing	156 (11.5%)	163 (12.0%)	-0.01	2,793 (11.4%)	2,714 (11.1%)	0.00
**Health Plan Indicator**						

© 2023 Lin KJ et al. *JAMA Network Open*.

Characteristics Number of Patients (%)	With Dementia			Without Dementia		
	Apixaban (n=1,360)	Warfarin (n=1,360)	St. Diff	Apixaban (n=24,396)	Warfarin (n=24,396)	St. Diff
Employer	1,196 (87.9%)	1,191 (87.6%)	0.00	21,299 (87.3%)	21,415 (87.8%)	-0.01
Health Plan	164 (12.1%)	169 (12.4%)	0.00	3,097 (12.7%)	2,981 (12.2%)	0.01
**MHSA Coverage Indicator**						
Not Covered/Claims Not Present	86 (6.3%)	84 (6.2%)	0.00	1,422 (5.8%)	1,362 (5.6%)	0.00
Covered/Possible MHSA Claims	1,125 (82.7%)	1,118 (82.2%)	0.01	20,456 (83.8%)	20,369 (83.5%)	0.00
Missing	149 (11.0%)	158 (11.6%)	-0.01	2,518 (10.3%)	2,665 (10.9%)	-0.01
**Plan Indicator**						
Basic/major medical	0 (0.0%)	0 (0.0%)	0.00	0 (0.0%)	0 (0.0%)	0.00
Comprehensive	618 (45.4%)	613 (45.1%)	0.00	8,751 (35.9%)	8,686 (35.6%)	0.00
EPO	0 (0.0%)	0 (0.0%)	0.00	71 (0.3%)	77 (0.3%)	0.00
HMO	101 (7.4%)	102 (7.5%)	0.00	2,291 (9.4%)	2,238 (9.2%)	0.00
POS	41 (3.0%)	36 (2.6%)	0.00	880 (3.6%)	894 (3.7%)	0.00
PPO	559 (41.1%)	567 (41.7%)	-0.01	11,454 (47.0%)	11,560 (47.4%)	0.00
POS with capitation	12 (0.9%)	16 (1.2%)	0.00	382 (1.6%)	379 (1.6%)	0.00
CDHP	4 (0.3%)	4 (0.3%)	0.00	207 (0.8%)	205 (0.8%)	0.00
HDHP	2 (0.1%)	1 (0.1%)	0.00	97 (0.4%)	98 (0.4%)	0.00
Missing	23 (1.7%)	21 (1.5%)	0.00	263 (1.1%)	259 (1.1%)	0.00

Abbreviations: ACE, angiotensin converting enzyme; CCI, combined comorbidity; CFI, claims-based frailty index; CKD, chronic kidney disease; COPD, chronic obstructive pulmonary disease; GI, gastrointestinal; NSAID, non-steroidal anti-inflammatory drug; PVD, peripheral vascular disease; SNRI, serotonin-norepinephrine reuptake inhibitor; SSRI, selective serotonin reuptake inhibitor.

© 2023 Lin KJ et al. *JAMA Network Open*.

eTable 28. Dementia and Adverse Outcomes in the US Medicare Population With Atrial Fibrillation Treated With Warfarin vs Apixaban After 1:1 Propensity Score Matching Pooled Across Medicare, Optum, and MarketScan Populations

Outcome	Apixaban (n=250,995)			Warfarin (n=250,995)			Hazard ratio (95% CI)	Rate difference(95% CI)
	PYs	Events	Rate (per 1,000PY)	PYs	Events	Rate (per 1,000PY)		
**Composite event without death**								
Total population	114474	4385	38.16642	113577	6357	53.23802	1.45 (1.37, 1.53)	16.14 (12.32, 19.96)
With dementia	8681	559	64.21884	8510	817	95.69847	1.46 (1.28, 1.67)	29.77 (18.41, 41.13)
Without dementia	105704	3834	36.2602	104897	5530	51.09968	1.45 (1.39, 1.52)	16.03 (13.63, 18.43)
**Ischemic stroke**								
Total population	115105	1362	13.46848	114654	1733	16.95717	1.28 (1.19, 1.37)	3.26 (2.32, 4.21)
With dementia	8748	190	24.96851	8627	228	26.2444	1.22 (1.00, 1.47)	4.89 (0.31, 9.48)
Without dementia	106271	1174	12.73324	105866	1495	16.05065	1.28 (1.18, 1.38)	3.12 (2.02, 4.23)
**Major bleeding**								
Total population	114775	2881	22.70424	113950	4599	35.68511	1.61 (1.54, 1.69)	13.47 (9.81, 17.14)
With dementia	8717	344	36.8403	8554	566	57.22576	1.68 (1.47, 1.91)	24.28 (14.27, 34.30)
Without dementia	105963	2546	21.94152	105231	4026	34.60591	1.59 (1.52, 1.67)	13.09 (10.09, 16.09)
**Major GI bleeding**								
Total population	115033	1620	11.56078	114388	2504	18.33413	1.55 (1.46, 1.65)	7.17 (5.42, 8.91)
With dementia	8751	160	15.48618	8608	266	23.3684	1.69 (1.38, 2.05)	9.39 (0.30, 18.48)
Without dementia	106183	1484	11.82063	105619	2233	18.07034	1.51 (1.41, 1.61)	6.55 (4.70, 8.40)
**IC bleeding**								
Total population	115287	774	6.951371	114826	1290	11.02825	1.54 (1.26, 1.88)	3.81 (2.08, 5.55)
With dementia	8766	126	14.34441	8639	213	24.63461	1.72 (1.38, 2.14)	10.29 (6.13, 14.44)
Without dementia	106438	637	6.363643	106030	1061	9.995074	1.54 (1.24, 1.91)	3.43 (1.82, 5.04)
**Death [A]**								
Total population	88926	10357	116.47	88644	11287	127.33	1.09 (1.06, 1.12)	10.86 (7.61, 14.11)
With dementia	7054	2344	332.25	6936	2663	383.9	1.15 (1.09, 1.22)	51.65 (31.81, 71.49)
Without dementia	81842	7884	96.33	81611	8521	104.41	1.08 (1.05, 1.12)	8.08 (5.01, 11.15)

Abbreviations: CI, Confidence Interval; GI, gastrointestinal; IC, intracranial; PY, person-years.
[A] Based on Medicare data only, as the death ascertainment in Optum and MarketScan is incomplete.

© 2023 Lin KJ et al. *JAMA Network Open*.

eTable 29. Dementia and Adverse Outcomes in the US Medicare Population With Atrial Fibrillation Treated With Warfarin vs Apixaban After 1:1 Propensity Score Matching in the Medicare Population

Outcome	Apixaban (n=193,272)			Warfarin (n=193,272)			Hazard ratio (95% CI)	Rate difference (95% CI)
	PYs	Events	Rate (per 1,000PY)	PYs	Events	Rate (per 1,000PY)		
**Composite event without death**								
Total population	88179	3413	38.71	87479	5020	57.39	1.48 (1.42, 1.55)	18.68 (16.63, 20.73)
With dementia	6968	439	63	6806	664	97.56	1.54 (1.37, 1.74)	34.56 (25.09, 44.04)
Without dementia	81180	2971	36.6	80570	4332	53.77	1.47 (1.40, 1.54)	17.17 (15.10, 19.24)
**Composite event with death** [A]								
Total population	88179	13087	148.41	87479	15266	174.51	1.18 (1.15, 1.20)	26.10 (22.34, 29.86)
With dementia	6968	2653	380.71	6806	3102	455.77	1.19 (1.13, 1.26)	75.05 (53.44, 96.67)
Without dementia	81180	10306	126.95	80570	12058	149.66	1.18 (1.15, 1.21)	22.71 (19.08, 26.33)
**Ischemic stroke**								
Total population	88695	985	11.11	88366	1257	14.22	1.28 (1.18, 1.39)	3.12 (2.07, 4.17)
With dementia	7026	138	19.64	6903	175	25.35	1.29 (1.03, 1.61)	5.71 (0.73, 10.70)
Without dementia	81646	848	10.39	81365	1074	13.2	1.27 (1.16, 1.39)	2.81 (1.76, 3.87)
**Major bleeding**								
Total population	88401	2335	26.41	87746	3748	42.71	1.62 (1.53, 1.70)	16.30 (14.56, 18.04)
With dementia	6995	289	41.31	6839	478	69.89	1.69 (1.46, 1.95)	28.58 (20.71, 36.45)
Without dementia	81371	2040	25.07	80807	3250	40.22	1.60 (1.52, 1.69)	15.15 (13.39, 16.91)
**Major GI bleeding**								
Total population	88601	1358	15.33	88093	2082	23.63	1.54 (1.44, 1.65)	8.31 (7.00, 9.61)
With dementia	7023	139	19.79	6881	231	33.57	1.69 (1.37, 2.09)	13.78 (8.34, 19.21)
Without dementia	81540	1224	15.01	81114	1844	22.73	1.51 (1.41, 1.63)	7.72 (6.39, 9.06)
**IC bleeding**								
Total population	88827	575	6.47	88474	1016	11.48	1.77 (1.60, 1.96)	5.01 (4.13, 5.89)
With dementia	7038	100	14.21	6912	172	24.88	1.75 (1.37, 2.24)	10.68 (6.03, 15.32)
Without dementia	81764	464	5.67	81465	830	10.19	1.79 (1.60, 2.01)	4.51 (3.65, 5.38)
**Death**								
Total population	88926	10357	116.47	88644	11287	127.33	1.09 (1.06, 1.12)	10.86 (7.61, 14.11)
With dementia	7054	2344	332.25	6936	2663	383.9	1.15 (1.09, 1.22)	51.65 (31.81, 71.49)
Without dementia	81842	7884	96.33	81611	8521	104.41	1.08 (1.05, 1.12)	8.08 (5.01, 11.15)

Abbreviations: CI, Confidence Interval; GI, gastrointestinal; IC, intracranial; PY, person-years.
[A] including ischemic stroke, major bleeding, and death

© 2023 Lin KJ et al. *JAMA Network Open*.

eTable 30. Dementia and Adverse Outcomes in the US Medicare Population With Atrial Fibrillation Treated With Warfarin vs Apixaban After 1:1 Propensity Score Matching in the Optum Population

Outcome	Apixaban (n=37,266)			Warfarin (n=37,266)			Hazard ratio (95% CI)	Rate difference (95% CI)
	PYs	Events	Rate (per 1,000PY)	PYs	Events	Rate (per 1,000PY)		
**Composite event without death**								
Total population	17113	633	36.99	16975	893	52.6	1.42 (1.28, 1.57)	15.62 (11.12, 20.11)
With dementia	1128	83	73.53	1123	107	95.24	1.30 (0.97, 1.73)	21.71 (-2.29, 45.71)
Without dementia	15962	566	35.46	15826	787	49.73	1.40 (1.26, 1.56)	14.27 (9.73, 18.81)
**Ischemic stroke**								
Total population	17193	219	12.74	17112	288	16.83	1.32 (1.11, 1.57)	4.09 (1.52, 6.67)
With dementia	1134	34	29.98	1139	33	28.96	0.97 (0.60, 1.56)	-1.02 (-15.13, 13.10)
Without dementia	16038	178	11.1	15946	251	15.74	1.42 (1.17, 1.72)	4.64 (2.10, 7.18)
**Major bleeding**								
Total population	17160	359	20.92	17046	574	33.67	1.61 (1.41, 1.84)	12.75 (9.25, 16.26)
With dementia	1135	35	30.81	1130	64	56.6	1.83 (1.21, 2.77)	25.79 (8.57, 43.01)
Without dementia	16001	347	21.68	15890	514	32.35	1.49 (1.30, 1.71)	10.66 (7.05, 14.27)
**Major GI bleeding**								
Total population	17195	184	10.7	17107	294	17.19	1.60 (1.33, 1.93)	6.48 (3.98, 8.98)
With dementia	1138	14	12.29	1139	29	25.45	2.07 (1.09, 3.91)	13.16 (1.88, 24.44)
Without dementia	16035	186	11.6	15943	264	16.56	1.43 (1.18, 1.72)	4.96 (2.36, 7.56)
**IC bleeding**								
Total population	17219	124	7.2	17152	181	10.55	1.47 (1.17, 1.84)	3.35 (1.36, 5.34)
With dementia	1139	16	14.04	1140	28	24.55	1.75 (0.94, 3.23)	10.51 (-0.89, 21.92)
Without dementia	16059	116	7.22	15987	151	9.44	1.31 (1.03, 1.67)	2.22 (0.22, 4.22)

Abbreviations: CI, Confidence Interval; GI, gastrointestinal; IC, intracranial; PY, person-years.

© 2023 Lin KJ et al. *JAMA Network Open*.

eTable 31. Dementia and Adverse Outcomes in the US Medicare Population With Atrial Fibrillation Treated With Warfarin vs Apixaban After 1:1 Propensity Score Matching in the MarketScan Population

Outcome	Apixaban (n=20,694)			Warfarin (n=20,694)			Hazard ratio (95% CI)	Rate difference (95% CI)
	PYs	Events	Rate (per 1,000PY)	PYs	Events	Rate (per 1,000PY)		
**Composite event without death**								
Total population	9191	332	36.12	9308	475	51.03	1.32 (1.14, 1.52)	11.75 (5.76, 17.75)
With dementia	588	35	59.44	596	49	82.16	1.38 (0.90, 2.13)	22.72 (-7.56, 53.00)
Without dementia	8583	291	33.9	8681	416	47.92	1.39 (1.20, 1.62)	13.66 (7.54, 19.77)
**Ischemic stroke**								
Total population	9225	158	17.13	9367	196	20.92	1.19 (0.97, 1.48)	3.35 (-0.62, 7.31)
With dementia	592	16	26.99	601	21	34.9	1.29 (0.68, 2.48)	7.92 (-12.03, 27.86)
Without dementia	8612	141	16.37	8733	178	20.38	1.15 (0.92, 1.44)	2.64 (-1.44, 6.71)
**Major bleeding**								
Total population	9223	184	19.95	9344	305	32.64	1.49 (1.24, 1.79)	9.95 (5.35, 14.55)
With dementia	591	20	33.8	600	27	45	1.33 (0.75, 2.38)	11.20 (-11.33, 33.72)
Without dementia	8611	165	19.16	8716	262	30.06	1.66 (1.36, 2.02)	12.19 (7.49, 16.89)
**Major GI bleeding**								
Total population	9246	79	8.54	9377	136	14.5	1.65 (1.24, 2.19)	5.49 (2.43, 8.54)
With dementia	594	7	11.77	603	9	14.91	1.27 (0.47, 3.40)	3.13 (-9.94, 16.21)
Without dementia	8632	68	7.88	8745	115	13.15	1.70 (1.27, 2.26)	6.00 (2.78, 9.23)
**IC bleeding**								
Total population	9250	72	7.78	9390	107	11.39	1.25 (0.92, 1.69)	1.99 (-0.76, 4.75)
With dementia	593	10	16.85	603	10	16.57	0.99 (0.41, 2.37)	-0.28 (-14.93, 14.36)
Without dementia	8635	64	7.41	8758	87	9.93	1.41 (1.00, 1.98)	2.71 (0.04, 5.38)

Abbreviations: CI, Confidence Interval; GI, gastrointestinal; IC, intracranial; PY, person-years.

© 2023 Lin KJ et al. *JAMA Network Open*.

eTable 32. Dementia and Adverse Outcomes in the US Medicare Population With Atrial Fibrillation Treated With Warfarin vs Dabigatran After 1:1 Propensity Score Matching Pooled Across Medicare, Optum, and MarketScan Population

Outcome	Apixaban (n=63,359)			Dabigatran (n=63,359)			Hazard ratio (95% CI)	Rate difference (95% CI)
	PYs	Events	Rate (per 1,000PY)	PYs	Events	Rate (per 1,000PY)		
**Composite event without death**								
Total population	29427	976	33.14116	29412	1176	37.49358	1.20 (1.10, 1.31)	6.34 (2.65, 10.03)
With dementia	1831	101	54.88503	1828	156	84.49321	1.55 (1.20, 1.98)	29.60 (11.57, 47.63)
Without dementia	27567	831	29.03575	27546	1021	33.63456	1.21 (1.07, 1.37)	5.77 (1.12, 10.43)
**Ischemic stroke**								
Total population	29572	304	10.46272	29603	361	13.62093	1.18 (1.02, 1.38)	1.83 (0.13, 3.52)
With dementia	1844	22	11.57847	1852	51	34.04786	2.73 (1.11, 6.71)	10.64 (1.28, 19.99)
Without dementia	27687	245	8.847735	27713	309	11.67303	1.26 (1.07, 1.49)	2.32(0.53, 4.11)
**Major bleeding**								
Total population	29490	646	20.79802	29491	799	21.97972	1.11 (0.86, 1.42)	2.21 (-3.66, 8.08)
With dementia	1834	75	39.17351	1837	106	50.95005	1.42 (1.06, 1.91)	18.58 (3.42, 33.73)
Without dementia	27627	552	19.24613	27616	695	20.14472	1.09 (0.81, 1.47)	1.89(-4.44, 8.21)
**Major GI bleeding**								
Total population	29555	346	10.2717	29546	540	13.78916	1.44 (1.12, 1.86)	4.10 (-0.48, 8.68)
With dementia	1842	29	12.84131	1847	61	28.18455	3.81 (0.43, 34.11)	18.07 (7.13, 29.02)
Without dementia	27676	310	9.398365	27661	485	12.86652	1.42 (1.04, 1.95)	3.87 (-0.89, 8.62)
**IC bleeding**								
Total population	29604	182	6.126057	29659	163	5.474064	0.89 (0.72, 1.10)	-0.65 (-1.88, 0.58)
With dementia	1845	32	6.108395	1858	31	12.55449	0.23 (0.03, 2.06)	2.02 (-7.00, 11.03)
Without dementia	27725	150	67.12297	27763	132	4.752617	0.82 (0.59, 1.16)	-1.1063 (-3.10, 0.88)
**Death** [A]								
Total population	23284	1969	84.56	23311	1926	82.62	0.98 (0.92, 1.04)	-1.94 (-7.19, 3.31)
With dementia	1506	434	288.17	1508	431	285.71	0.99 (0.87, 1.13)	-2.46 (-40.70, 35.78)
Without dementia	21738	1503	69.14	21779	1474	67.68	0.98 (0.91, 1.05)	-1.46 (-6.38, 3.45)

Abbreviations: CI, Confidence Interval; GI, gastrointestinal; IC, intracranial; PY, person-years.

[A] Based on Medicare data only, as the death ascertainment in Optum and MarketScan is incomplete.

© 2023 Lin KJ et al. *JAMA Network Open*.

eTable 33. Dementia and Adverse Outcomes in the US Medicare Population With Atrial Fibrillation Treated With Dabigatran vs Apixaban After 1:1 Propensity Score Matching in the Medicare Population

Outcome	Apixaban (n=49,648)			Dabigatran (n=49,648)			Hazard ratio (95% CI)	Rate difference (95% CI)
	PYs	Events	Rate (per 1,000PY)	PYs	Events	Rate (per 1,000PY)		
**Composite event without death**								
Total population	23113	777	33.62	23083	958	41.5	1.23 (1.12, 1.36)	7.88 (4.35, 11.42)
With dementia	1491	81	54.32	1480	124	83.78	1.54 (1.17, 2.04)	29.46 (10.56, 48.37)
Without dementia	21591	666	30.85	21577	841	38.98	1.26 (1.14, 1.40)	8.13 (4.60, 11.65)
**Composite event with death** [A]								
Total population	23113	2612	113.01	23083	2725	118.05	1.04 (0.99, 1.10)	5.04 (-1.16, 11.24)
With dementia	1491	491	329.26	1480	524	354.04	1.08 (0.95, 1.22)	24.78 (-17.25, 66.82)
Without dementia	21591	2052	95.04	21577	2187	101.35	1.07 (1.00, 1.13)	6.31 (0.40, 12.23)
**Ischemic stroke**								
Total population	23234	229	9.86	23249	262	11.27	1.14 (0.96, 1.37)	1.41 (-0.46, 3.28)
With dementia	1502	19	12.64	1501	34	22.65	1.79 (1.02, 3.14)	10.00 (0.50, 19.51)
Without dementia	21690	192	8.85	21724	230	10.59	1.20 (0.99, 1.45)	1.74 (-0.12, 3.59)
**Major bleeding**								
Total population	23161	526	22.71	23141	686	29.64	1.31 (1.16, 1.46)	6.93 (3.99, 9.88)
With dementia	1494	61	40.82	1486	92	61.89	1.52 (1.10, 2.10)	21.08 (4.80, 37.35)
Without dementia	21638	446	20.61	21629	599	27.69	1.34 (1.19, 1.52)	7.08 (4.15, 10.01)
**Major GI bleeding**								
Total population	23213	291	12.54	23186	475	20.49	1.63 (1.41, 1.89)	7.95 (5.61, 10.29)
With dementia	1500	25	16.66	1494	54	36.13	2.17 (1.35, 3.48)	19.47 (7.82, 31.11)
Without dementia	21674	263	12.13	21666	430	19.85	1.64 (1.40, 1.91)	7.71 (5.33, 10.09)
**IC bleeding**								
Total population	23255	140	6.02	23287	132	5.67	0.94 (0.74, 1.19)	-0.35 (-1.74, 1.04)
With dementia	1502	23	15.31	1503	28	18.62	1.22 (0.70, 2.11)	3.32 (-6.00, 12.63)
Without dementia	21721	106	4.88	21759	104	4.78	0.98 (0.75, 1.28)	-0.10 (-1.41, 1.21)
**Death**								
Total population	23284	1969	84.56	23311	1926	82.62	0.98 (0.92, 1.04)	-1.94 (-7.19, 3.31)
With dementia	1506	434	288.17	1508	431	285.71	0.99 (0.87, 1.13)	-2.46 (-40.70, 35.78)
Without dementia	21738	1503	69.14	21779	1474	67.68	0.98 (0.91, 1.05)	-1.46 (-6.38, 3.45)

Abbreviations: CI, Confidence Interval; GI, gastrointestinal; IC, intracranial; PY, person-years.
[A] including ischemic stroke, major bleeding, and death

© 2023 Lin KJ et al. *JAMA Network Open*.

eTable 34. Dementia and Adverse Outcomes in the US Medicare Population With Atrial Fibrillation Treated With Dabigatran vs Apixaban After 1:1 Propensity Score Matching in the Optum Population

Outcome	Apixaban (n=7,023)			Dabigatran (n=7,023)			Hazard ratio (95% CI)	Rate difference (95% CI)
	PYs	Events	Rate (per 1,000PY)	PYs	Events	Rate (per 1,000PY)		
Composite event without death								
Total population	3293	102	30.97	3297	112	33.97	1.10 (0.84, 1.43)	3.00 (-5.70, 11.70)
With dementia	196	10	51.49	202	15	74.8	1.45 (0.65, 3.24)	23.31 (-49.40, 96.02)
Without dementia	3081	95	30.83	3087	95	30.77	1.00 (0.75, 1.33)	-0.05 (-8.81, 8.70)
Ischemic stroke								
Total population	3305	34	10.28	3312	47	14.19	1.38 (0.89, 2.15)	3.91 (-1.42, 9.24)
With dementia	196	2	10.3	204	7	35.1	3.38 (0.70, 16.25)	24.80 (-31.86, 81.45)
Without dementia	3094	28	9.05	3099	40	12.9	1.43 (0.88, 2.31)	3.85 (-1.36, 9.07)
Major bleeding								
Total population	3300	61	18.48	3307	59	17.84	0.97 (0.67, 1.38)	-0.65 (-7.14, 5.85)
With dementia	196	5	259.97	204	7	200.03	1.34 (0.43, 4.24)	8.76 (-40.56, 58.07)
Without dementia	3089	59	19.1	3096	50	16.15	0.85 (0.58, 1.23)	-2.95 (-9.57, 3.67)
Major GI bleeding								
Total population	3306	27	8.17	3312	34	10.26	1.26 (0.76, 2.08)	2.10 (-2.53, 6.72)
With dementia	196	1	5.14	205	4	19.2	3.81 (0.43, 34.11)	14.06 (-29.33, 57.45)
Without dementia	3095	29	9.37	3100	28	9.03	0.96 (0.57, 1.62)	-0.34 (-5.11, 4.44)
IC bleeding								
Total population	3309	24	7.25	3320	16	4.82	0.66 (0.35, 1.25)	-2.43 (-6.17, 1.31)
With dementia	197	4	21.02	206	1	4.94	0.23 (0.03, 2.06)	-16.08 (-61.47, 29.31)
Without dementia	3097	22	7.1	3106	15	4.83	0.68 (0.35, 1.31)	-2.27 (-6.12, 1.57)

Abbreviations: CI, Confidence Interval; GI, gastrointestinal; IC, intracranial; PY, person-years.

© 2023 Lin KJ et al. *JAMA Network Open*.

eTable 35. Dementia and Adverse Outcomes in the US Medicare Population With Atrial Fibrillation Treated With Dabigatran vs Apixaban After 1:1 Propensity Score Matching in the MarketScan Population

Outcome	Apixaban (n=6,688)			Dabigatran (n=6,668)			Hazard ratio (95% CI)	Rate difference (95% CI)
	PYs	Events	Rate (per 1,000PY)	PYs	Events	Rate (per 1,000PY)		
**Composite event**								
Total population	3021	97	32.1	3032	106	34.95	1.09 (0.83, 1.43)	2.85 (-6.37, 12.07)
With dementia	144	10	70.11	146	17	117.57	1.68 (0.77, 3.67)	47.46 (-58.56, 153.47)
Without dementia	2895	70	24.17	2882	85	29.49	1.22 (0.89, 1.67)	5.31 (-3.14, 13.76)
**Ischemic stroke**								
Total population	3033	41	13.51	3042	52	17.09	1.27 (0.84, 1.91)	3.58 (-2.64, 9.80)
With dementia	146	1	6.83	147	10	72.01	10.77 (1.37, 4.47)	65.18 (-79.16, 209.51)
Without dementia	2903	25	8.61	2890	39	13.49	1.57 (0.95, 2.59)	4.88 (-0.53, 10.30)
**Major bleeding**								
Total population	3029	59	19.47	3043	54	17.74	0.91 (0.63, 1.32)	-1.73 (-8.59, 5.13)
With dementia	144	9	61.82	147	7	48.16	0.78 (0.29, 2.10)	-13.66 (-90.82, 63.50)
Without dementia	2900	47	16.2	2891	46	15.91	0.98 (0.65, 1.47)	-0.29 (-6.82, 6.23)
**Major GI bleeding**								
Total population	3036	28	9.22	3048	31	10.17	1.10 (0.66, 1.84)	0.95 (-4.00, 5.90)
With dementia	146	3	21.15	148	3	20.86	0.98 (0.20, 4.86)	-0.28 (-47.66, 47.10)
Without dementia	2907	18	6.19	2895	27	9.33	1.51 (0.83, 2.74)	3.13 (-1.40, 7.67)
**IC bleeding**								
Total population	3040	18	5.92	3052	15	4.91	0.83 (0.42, 1.65)	-1.01 (-4.70, 2.69)
With dementia	146	5	33.27	149	2	13.94	0.42 (0.08, 2.19)	-19.34 (-77.89, 39.22)
Without dementia	2907	22	7.57	2898	13	4.48	0.59 (0.30, 1.18)	-3.08 (-7.08, 0.91)

Abbreviations: CI, Confidence Interval; GI, gastrointestinal; IC, intracranial; PY, person-years.

© 2023 Lin KJ et al. *JAMA Network Open*.

eTable 36. Dementia and Adverse Outcomes in the US Medicare Population With Atrial Fibrillation Treated With Rivaroxaban vs Dabigatran After 1:1 Propensity Score Matching Pooled Across Medicare, Optum, and MarketScan Population

Outcome	Apixaban (n=265,877)			Rivaroxaban (n=265,877)			Hazard ratio (95% CI)	Rate difference (95% CI)
	PYs	Events	Rate (per 1,000PY)	PYs	Events	Rate (per 1,000PY)		
**Composite event without death**								
Total population	119901	4047	32.99871	119022	6106	47.53788	1.46 (1.33, 1.61)	15.05 (9.98, 20.13)
With dementia	8262	550	68.52891	8114	712	87.42522	1.30 (1.12, 1.50)	20.50 (9.85, 31.15)
Without dementia	111561	3416	29.78329	110857	5387	45.16366	1.55 (1.43, 1.68)	15.85 (11.37, 20.34)
**Ischemic stroke**								
Total population	120476	1255	11.75661	120171	1470	13.74028	1.18 (1.09, 1.27)	1.79 (0.95, 2.63)
With dementia	8331	179	24.3652	8233	185	28.53484	1.04 (0.85, 1.28)	0.52 (-3.88, 4.92)
Without dementia	112066	1061	10.73625	111880	1270	12.64134	1.20 (1.11, 1.30)	1.88 (1.04, 2.72)
**Major bleeding**								
Total population	120177	2669	20.11409	119319	4564	33.04205	1.67 (1.53, 1.82)	13.17 (7.89, 18.45)
With dementia	8297	346	40.74936	8148	519	59.83218	1.53 (1.34, 1.75)	20.97 (12.81, 29.13)
Without dementia	111795	2251	17.99159	111118	4051	31.85852	1.81 (1.72, 1.91)	14.14 (9.88, 18.39)
**Major GI bleeding**								
Total population	120427	1442	10.13534	119662	3018	21.19779	2.11 (1.98, 2.24)	11.21 (7.45, 14.97)
With dementia	8330	166	18.78354	8187	301	33.09006	1.84 (1.52, 2.22)	16.53 (11.43, 21.62)
Without dementia	112008	1244	9.28247	111416	2724	20.82389	2.20 (2.06, 2.36)	11.71 (8.52, 14.90)
**IC bleeding**								
Total population	120636	771	6.383051	120340	854	6.933609	1.09 (1.04, 1.15)	5.39 (-0.28, 11.06)
With dementia	8346	124	14.73308	8249	140	16.49513	1.03 (0.85, 1.24)	7.78 (-30.68, 46.25)
Without dementia	112201	632	5.628005	112031	711	6.313199	1.08 (1.04, 1.11)	3.70 (0.66, 6.75)
**Death** [A]								
Total population	87430	8273	94.62	87150	9186	105.4	1.11 (1.08, 1.15)	10.78 (7.81, 13.75)
With dementia	6375	2022	317.13	6290	2274	361.49	1.14 (1.07, 1.21)	44.35 (24.06, 64.64)
Without dementia	80963	6311	77.95	80839	6814	84.29	1.08 (1.04, 1.12)	6.34 (3.57, 9.12)

Abbreviations: CI, Confidence Interval; GI, gastrointestinal; IC, intracranial; PY, person-years.

[A] Based on Medicare data only, as the death ascertainment in Optum and MarketScan is incomplete.

© 2023 Lin KJ et al. *JAMA Network Open*.

eTable 37. Dementia and Adverse Outcomes in the US Medicare Population With Atrial Fibrillation Treated With Rivaroxaban vs Apixaban After 1:1 Propensity Score Matching in the Medicare Population

Outcome	Apixaban (n=192,640)			Rivaroxaban (n=192,640)			Hazard ratio (95% CI)	Rate difference (95% CI)
	PYs	Events	Rate (per 1,000PY)	PYs	Events	Rate (per 1,000PY)		
**Composite event without death**								
Total population	86778	2993	34.49	86018	4651	54.07	1.57 (1.50, 1.64)	19.58 (17.59, 21.56)
With dementia	6296	402	63.85	6173	537	86.98	1.36 (1.19, 1.55)	23.13 (13.48, 32.78)
Without dementia	80396	2528	31.44	79836	4084	51.15	1.63 (1.55, 1.71)	19.71 (17.72, 21.70)
**Composite event with death [A]**								
Total population	86778	10689	123.18	86018	13067	151.91	1.23 (1.20, 1.26)	28.73 (25.24, 32.23)
With dementia	6296	2314	367.53	6173	2641	427.77	1.16 (1.10, 1.23)	60.24 (38.10, 82.39)
Without dementia	80396	8434	104.91	79836	10301	129.03	1.23 (1.19, 1.27)	24.12 (20.77, 27.47)
**Ischemic stroke**								
Total population	87230	842	9.65	86945	987	11.35	1.18 (1.07, 1.29)	1.70 (0.74, 2.66)
With dementia	6351	121	19.05	6269	118	18.82	0.99 (0.77, 1.27)	-0.23 (-5.03, 4.57)
Without dementia	80798	703	8.7	80658	854	10.59	1.22 (1.10, 1.34)	1.89 (0.93, 2.85)
**Major bleeding**								
Total population	86971	2061	23.7	86214	3610	41.87	1.76 (1.67, 1.86)	18.18 (16.47, 19.88)
With dementia	6319	272	43.04	6194	416	67.16	1.56 (1.34, 1.81)	24.12 (15.89, 32.35)
Without dementia	80556	1750	21.72	80009	3176	39.7	1.83 (1.72, 1.94)	17.97 (16.26, 19.69)
**Major GI bleeding**								
Total population	87156	1143	13.11	86480	2407	27.83	2.12 (1.98, 2.27)	14.72 (13.37, 16.07)
With dementia	6346	134	21.11	6225	245	39.36	1.86 (1.50, 2.29)	18.24 (12.16, 24.33)
Without dementia	80714	994	12.31	80238	2154	26.84	2.18 (2.02, 2.35)	14.53 (13.16, 15.90)
**IC bleeding**								
Total population	87335	554	6.34	87041	636	7.31	1.15 (1.03, 1.29)	0.96 (0.19, 1.74)
With dementia	6359	91	14.31	6276	106	16.89	1.18 (0.89, 1.56)	2.58 (-1.78, 6.94)
Without dementia	80882	453	5.6	80745	521	6.45	1.15 (1.02, 1.31)	0.85 (0.09, 1.61)
**Death**								
Total population	87430	8273	94.62	87150	9186	105.4	1.11 (1.08, 1.15)	10.78 (7.81, 13.75)
With dementia	6375	2022	317.13	6290	2274	361.49	1.14 (1.07, 1.21)	44.35 (24.06, 64.64)
Without dementia	80963	6311	77.95	80839	6814	84.29	1.08 (1.04, 1.12)	6.34 (3.57, 9.12)

Abbreviations: CI, Confidence Interval; GI, gastrointestinal; IC, intracranial; PY, person-years.
[A] including ischemic stroke, major bleeding, and death

© 2023 Lin KJ et al. *JAMA Network Open*.

eTable 38. Dementia and Adverse Outcomes in the US Medicare Population With Atrial Fibrillation Treated With Rivaroxaban vs Apixaban After 1:1 Propensity Score Matching in the Optum Population

Outcome	Apixaban (n=47,568)			Rivaroxaban (n=47,568)			Hazard ratio (95% CI)	Rate difference (95% CI)
	PYs	Events	Rate (per 1,000PY)	PYs	Events	Rate (per 1,000PY)		
**Composite event without death**								
Total population	21638	692	31.98	21527	951	44.18	1.38 (1.25, 1.52)	12.20 (8.51, 15.88)
With dementia	1387	105	75.66	1368	113	82.58	1.09 (0.84, 1.42)	6.91 (-14.09, 27.92)
Without dementia	20291	573	28.24	20140	850	42.2	1.49 (1.34, 1.66)	13.97 (10.31, 17.63)
**Ischemic stroke**								
Total population	21728	240	11.05	21683	284	13.1	1.19 (1.00, 1.41)	2.05 (-0.01, 4.12)
With dementia	1396	40	28.64	1384	43	31.05	1.08 (0.70, 1.67)	2.41 (-10.43, 15.25)
Without dementia	20367	208	10.21	20282	242	11.93	1.17 (0.97, 1.41)	1.72 (-0.33, 3.77)
**Major bleeding**								
Total population	21689	405	18.67	21590	637	29.5	1.58 (1.39, 1.79)	10.83 (7.91, 13.76)
With dementia	1396	48	34.38	1376	65	47.24	1.37 (0.95, 1.99)	12.86 (-2.19, 27.91)
Without dementia	20337	329	16.18	20195	583	28.87	1.78 (1.56, 2.04)	12.69 (9.77, 15.61)
**Major GI bleeding**								
Total population	21728	216	9.94	21637	429	19.83	1.99 (1.69, 2.35)	9.89 (7.59, 12.18)
With dementia	1400	20	14.28	1380	41	29.69	2.07 (1.22, 3.54)	15.41 (4.37, 26.44)
Without dementia	20371	177	8.69	20237	397	19.62	2.26 (1.89, 2.69)	10.93 (8.61, 13.24)
**IC bleeding**								
Total population	21756	135	6.21	21723	131	6.03	0.97 (0.76, 1.24)	-0.17 (-1.65, 1.30)
With dementia	1402	21	14.97	1389	18	12.95	0.87 (0.46, 1.63)	-2.02 (-10.78, 6.74)
Without dementia	20393	112	5.49	20317	115	5.66	1.03 (0.79, 1.34)	0.17 (-1.28, 1.62)

Abbreviations: CI, Confidence Interval; GI, gastrointestinal; IC, intracranial; PY, person-years.

© 2023 Lin KJ et al. *JAMA Network Open*.

eTable 39. Dementia and Adverse Outcomes in the US Medicare Population With Atrial Fibrillation Treated With Rivaroxaban vs Apixaban After 1:1 Propensity Score Matching in the MarketScan Population

Outcome	Apixaban (n=25,804)			Rivaroxaban (n=25,804)			Hazard ratio (95% CI)	Rate difference (95% CI)
	PYs	Events	Rate (per 1,000PY)	PYs	Events	Rate (per 1,000PY)		
**Composite event**								
Total population	11485	362	31.52	11477	504	43.91	1.39 (1.22, 1.59)	12.39 (7.37, 17.42)
With dementia	586	31	52.85	578	59	101.93	1.93 (1.25, 2.98)	49.08 (17.10, 81.05)
Without dementia	10874	315	28.97	10881	453	41.63	1.44 (1.24, 1.66)	12.66 (7.67, 17.66)
**Ischemic stroke**								
Total population	11518	173	15.02	11543	199	17.24	1.15 (0.94, 1.41)	2.22 (-1.06, 5.50)
With dementia	589	17	28.86	585	23	39.27	1.36 (0.73, 2.55)	10.41 (-10.70, 31.52)
Without dementia	10901	150	13.76	10940	174	15.9	1.16 (0.93, 1.44)	2.14 (-1.09, 5.37)
**Major bleeding**								
Total population	11517	203	17.63	11515	317	27.53	1.56 (1.31, 1.86)	9.90 (6.02, 13.78)
With dementia	589	16	27.14	584	36	61.62	2.27 (1.26, 4.08)	34.48 (10.35, 58.60)
Without dementia	10902	172	15.78	10914	292	26.75	1.70 (1.40, 2.05)	10.98 (7.11, 14.85)
**Major GI bleeding**								
Total population	11543	83	7.19	11545	182	15.76	2.19 (1.69, 2.84)	8.57 (5.81, 11.34)
With dementia	591	6	10.15	588	14	23.81	2.34 (0.90, 6.09)	13.66 (-1.22, 28.54)
Without dementia	10923	73	6.68	10941	173	15.81	2.37 (1.80, 3.11)	9.13 (6.32, 11.94)
**IC bleeding**								
Total population	11545	82	7.1	11576	87	7.52	1.06 (0.78, 1.43)	0.41 (-1.79, 2.62)
With dementia	591	7	11.83	589	15	25.43	2.15 (0.87, 5.26)	13.60 (-1.98, 29.17)
Without dementia	10926	67	6.13	10969	75	6.84	1.11 (0.80, 1.55)	0.71 (-1.43, 2.84)

Abbreviations: CI, Confidence Interval; GI, gastrointestinal; IC, intracranial; PY, person-years.

© 2023 Lin KJ et al. *JAMA Network Open*.

eTable 40. Sensitivity Analyses for the Primary Outcome in Older Adults With Atrial Fibrillation in the Medicare Population[B]

Outcomes	Primary Analysis	Sensitivity Analysis 1 183-day-ITT with HDPS	Sensitivity Analysis 2 AT (7-day gap[C])	Sensitivity Analysis 3 AT (14-day gap[C])	Sensitivity Analysis 4 AT (30-day gap[C])	Sensitivity Analysis 5 Excluding patients with recent stroke[D]	Sensitivity Analysis 6 Excluding SNF residents	Sensitivity Analysis 7 Excluding patients taking a reduced dose	Sensitivity Analysis 8 183-day-ITT with States in PS Model
**Warfarin vs. Apixaban, Hazard Ratio (95% CI)**									
Total population	1.48 (1.42, 1.55)	1.58 (1.51, 1.65)	1.59 (1.51, 1.67)	1.59 (1.52, 1.66)	1.60 (1.54, 1.66)	1.52 (1.45, 1.60)	1.51 (1.43, 1.58)	1.43 (1.36, 1.50)	1.48 (1.41, 1.54)
With dementia	1.54 (1.37, 1.74)	1.62 (1.42, 1.85)	1.58 (1.38, 1.81)	1.55 (1.38, 1.75)	1.53 (1.38, 1.70)	1.59 (1.40, 1.82)	1.48 (1.23, 1.78)	1.25 (1.08, 1.45)	1.46 (1.29, 1.65)
Without dementia	1.47 (1.40, 1.54)	1.61 (1.53, 1.70)	1.57 (1.49, 1.65)	1.58 (1.50, 1.65)	1.56 (1.50, 1.62)	1.50 (1.43, 1.58)	1.51 (1.43, 1.59)	1.41 (1.34, 1.49)	1.50 (1.43, 1.58)
**Warfarin vs. Apixaban, Rate Difference (95% CI)**									
Total population	18.68 (16.63, 20.73)	23.20 (20.82, 25.58)	21.54 (19.51, 23.57)	20.73 (19.04, 22.43)	19.01 (17.64, 20.39)	17.99 (15.99, 19.99)	17.23 (15.15, 19.32)	15.86 (13.66, 18.05)	18.43 (16.34, 20.51)
With dementia	34.56 (25.09, 44.04)	41.22 (30.03, 52.42)	37.31 (27.29, 47.33)	33.70 (25.43, 41.98)	30.75 (23.83, 37.67)	33.86 (24.29, 43.44)	27.71 (14.72, 40.70)	18.80 (6.87, 30.73)	30.74 (21.00, 40.47)
Without dementia	17.17 (15.10, 19.24)	23.02 (20.58, 25.46)	19.54 (17.50, 21.58)	19.09 (17.40, 20.79)	16.83 (15.46, 18.21)	16.40 (14.38, 18.43)	16.74 (14.64, 18.84)	14.52 (12.31, 16.72)	18.31 (16.19, 20.42)
**Dabigatran vs. Apixaban, Hazard Ratio (95% CI)**									
Total population	1.23 (1.12, 1.36)	1.30 (1.18, 1.43)	1.36 (1.22, 1.52)	1.36 (1.24, 1.50)	1.35 (1.24, 1.47)	1.38 (1.24, 1.53)	1.24 (1.12, 1.38)	1.28 (1.15, 1.44)	1.34 (1.22, 1.48)
With dementia	1.54 (1.17, 2.04)	1.46 (1.11, 1.92)	1.41 (1.04, 1.92)	1.23 (0.94, 1.61)	1.21 (0.96, 1.54)	1.38 (1.01, 1.88)	1.54 (1.05, 2.25)	1.29 (0.93, 1.79)	1.37 (1.04, 1.81)
Without dementia	1.26 (1.14, 1.40)	1.27 (1.14, 1.40)	1.25 (1.11, 1.40)	1.31 (1.18, 1.45)	1.30 (1.19, 1.42)	1.31 (1.17, 1.46)	1.27 (1.13, 1.42)	1.30 (1.16, 1.46)	1.31 (1.18, 1.45)
**Dabigatran vs. Apixaban, Rate Difference (95% CI)**									
Total population	7.88 (4.35, 11.42)	9.59 (6.09, 13.09)	11.49 (8.02, 14.97)	10.77 (7.99, 13.55)	9.65 (7.41, 11.89)	10.36 (7.00, 13.72)	7.21 (3.71, 10.72)	8.50 (4.76, 12.25)	10.64 (7.14, 14.13)
With dementia	29.46 (10.56, 48.37)	26.12 (7.52, 44.73)	27.40 (6.50, 48.30)	15.59 (-0.84, 32.02)	13.52 (0.32, 26.71)	19.40 (0.72, 38.09)	29.15 (3.79, 54.51)	19.36 (-5.71, 44.44)	22.04 (3.04, 41.05)
Without dementia	8.13 (4.60, 11.65)	8.22 (4.70, 11.75)	8.44 (4.96, 11.93)	9.24 (6.45, 12.03)	8.26 (6.00, 10.52)	8.40 (5.00, 11.79)	7.25 (3.81, 10.69)	8.16 (4.49, 11.84)	9.22 (5.72, 12.72)
**Rivaroxaban vs. Apixaban, Hazard Ratio (95% CI)**									
Total population	1.57 (1.50, 1.64)	1.58 (1.51, 1.65)	1.74 (1.65, 1.83)	1.68 (1.61, 1.76)	1.66 (1.59, 1.73)	1.62 (1.55, 1.71)	1.65 (1.57, 1.74)	1.52 (1.44, 1.61)	1.55 (1.48, 1.63)
With dementia	1.36 (1.19, 1.55)	1.41 (1.24, 1.60)	1.60 (1.38, 1.86)	1.49 (1.31, 1.70)	1.44 (1.28, 1.61)	1.37 (1.19, 1.58)	1.31 (1.08, 1.58)	1.37 (1.14, 1.64)	1.36 (1.19, 1.55)
Without dementia	1.63 (1.55, 1.71)	1.59 (1.51, 1.67)	1.75 (1.65, 1.85)	1.69 (1.61, 1.78)	1.67 (1.60, 1.74)	1.63 (1.55, 1.72)	1.68 (1.59, 1.77)	1.55 (1.46, 1.65)	1.60 (1.52, 1.68)
**Rivaroxaban vs. Apixaban, Rate Difference (95% CI)**									
Total population	19.58 (17.59, 21.56)	19.78 (17.79, 21.76)	21.46 (19.55, 23.38)	18.65 (17.10, 20.21)	17.33 (16.03, 18.62)	19.24 (17.31, 21.18)	19.47 (17.49, 21.46)	15.92 (13.75, 18.09)	19.23 (17.24, 21.22)
With dementia	23.13 (13.48, 32.78)	25.76 (16.23, 35.29)	32.86 (23.05, 42.67)	25.89 (17.91, 33.87)	22.90 (16.13, 29.68)	22.08 (12.36, 31.80)	18.51 (5.55, 31.46)	24.10 (10.33, 37.86)	23.26 (13.56, 32.97)
Without dementia	19.71 (17.72, 21.70)	18.90 (16.90, 20.90)	20.14 (18.23, 22.06)	17.61 (16.05, 19.17)	16.50 (15.20, 17.80)	18.36 (16.41, 20.32)	19.59 (17.58, 21.59)	15.69 (13.52, 17.86)	19.18 (17.18, 21.19)

Abbreviations: AT, as-treated analysis; CI, confidence interval; GI, gastrointestinal; HR, hazard ratio; HDPS, high-dimensional propensity score; IC, intracranial; ITT, intention-to-treat; SNF, skilled nursing facility

[A] Composite of Ischemic stroke and major bleeding

[B] These sensitivity analysis results were based on only the Medicare database, so they should not match the results displayed in figures 1-3 which are pooled across the Medicare, Optum, and MarketScan databases.

[C] as -treated analysis using the gap between prescriptions to define discontinuation of the drug of interest.

[D] Excluding patients with stroke in the 60 days prior to cohort entry

© 2023 Lin KJ et al. *JAMA Network Open*.
