# Supplementary material for: Comparative Effectiveness and Safety of Oral Anticoagulants by Dementia Status in Older Patients With Atrial Fibrillation
Source: JAMA Netw Open. 2023 Mar 28;6(3):e234086. doi: 10.1001/jamanetworkopen.2023.4086 (PMC10051113; doi:10.1001/jamanetworkopen.2023.4086)
Supplement: Supplement 2. — Data Sharing Statement [file jamanetwopen-e234086-s002.pdf]

Data Sharing Statement

Lin. Comparative Effectiveness and Safety of Oral Anticoagulants by Dementia Status in Older Patients With Atrial Fibrillation. *JAMA Netw Open*. Published March 28, 2023. doi:10.1001/jamanetworkopen.2023.4086

Data
Data available: No
